# Decoding the Inversion Symmetry Underlying Transcription Factor DNA-Binding Specificity and Functionality in the Genome

**DOI:** 10.1016/j.isci.2019.04.006

**Published:** 2019-04-06

**Authors:** Laurel A. Coons, Adam B. Burkholder, Sylvia C. Hewitt, Donald P. McDonnell, Kenneth S. Korach

**Affiliations:** 1 Receptor Biology Section, Reproductive and Developmental Biology Laboratory, National Institute of Environmental Health Sciences/National Institutes of Health, 111 T.W. Alexander Dr., Research Triangle Park, NC 27709, USA; 2 Department of Pharmacology and Cancer Biology, Duke University School of Medicine, Durham, NC 27710, USA; 3 Integrative Bioinformatics, National Institute of Environmental Health Sciences/National Institutes of Health, Research Triangle Park, NC 27709, USA

**Keywords:** Genetics, Molecular Biology, Evolutionary Biology, Bioinformatics, Omics

## Abstract

Understanding why a transcription factor (TF) binds to a specific DNA element in the genome and whether that binding event affects transcriptional output remains a great challenge. In this study, we demonstrate that TF binding in the genome follows inversion symmetry (IS). In addition, the specific DNA elements where TFs bind in the genome are determined by internal IS within the DNA element. These DNA-binding rules quantitatively define how TFs select the appropriate regulatory targets from a large number of similar DNA elements in the genome to elicit specific transcriptional and cellular responses. Importantly, we also demonstrate that these DNA-binding rules extend to DNA elements that do not support transcriptional activity. That is, the DNA-binding rules are obeyed, but the retention time of the TF at these non-functional DNA elements is not long enough to initiate and/or maintain transcription. We further demonstrate that IS is universal within the genome. Thus, IS is the DNA code that TFs use to interact with the genome and dictates (in conjunction with known DNA sequence constraints) which of those interactions are functionally active.

## Highlights

• Inversion symmetry (IS) is universal within the genome
• Transcription factor binding in the genome follows IS
• DNA elements where transcription factors bind are determined by internal IS
• Functionality is determined by residence time (dictated by IS and DNA sequence constraints)

## Graphical Abstract

**Figure:**
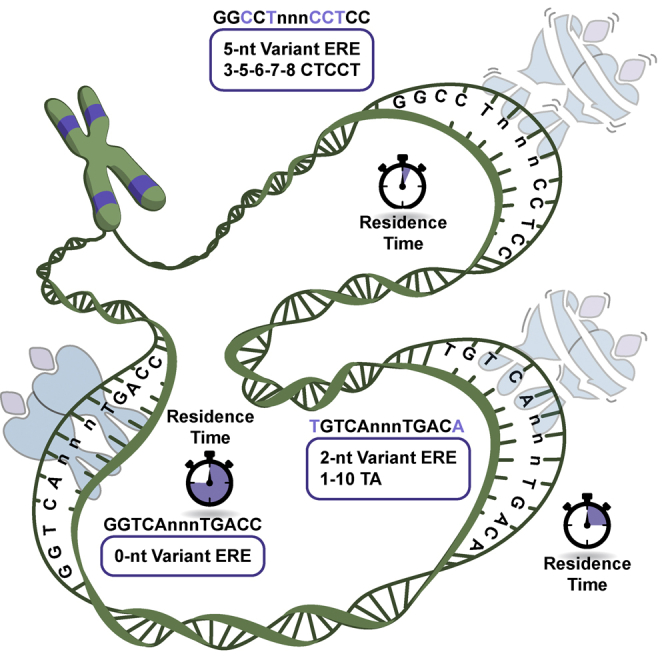

## Introduction

The specificity of gene expression in all organisms depends on the ability of transcription factors (TFs) to interact with *cis*-regulatory DNA elements in the genome. Achievement of specific DNA element recognition is non-trivial as genomes harbor thousands of consensus and near-consensus binding sequences. Understanding (1) why a TF binds to a specific DNA element in the genome and (2) whether that binding event affects transcriptional output remains a great challenge.

In the past decade, there has been a dramatic increase in the use of genome-wide technologies, such as chromatin immunoprecipitation (ChIP) sequencing (ChIPSeq) and ChIP exonuclease (ChIPExo), to identify where a TF physically associates with the genome. These studies have revealed that TF binding is widespread with thousands to tens of thousands of chromatin-interacting events, also known as peaks, in the genome. Although these genomic technologies have provided large-scale snapshots (i.e., point-in-time images) of TF binding in the genome, a full understanding of the mechanistic and quantitative details of specific DNA element recognition by TFs in the context of gene regulation is lacking (Todeschini et al., 2014).

Steroid nuclear receptors (sNRs) are TFs that are activated by the binding of steroid hormones (i.e., ligand-activated TFs) (Tang et al., 2011). The sNR family includes two divisions: the estrogen receptors (ERs) and the ketosteroid receptors (KRs). The ERs are estrogen receptor α (ERα) and estrogen receptor β (ERβ). The KRs are the androgen receptor (AR), glucocorticoid receptor (GR), mineralocorticoid receptor (MR), and progesterone receptor (PR). The sNR family regulate gene expression by interacting with sequence-specific *cis*-regulatory DNA elements in the genome: the estrogen response element (ERE) used by the ERs and the hormone response element (HRE) used by the KRs (Cotnoir-White et al., 2011, Helsen et al., 2012). The evolutionary relationship among the sNRs has been deduced by the high conservation in their DNA-binding domains (DBDs), and not surprisingly they exhibit sequence conservation at the level of their cognate *cis*-regulatory DNA elements (Laudet, 1997).

In a previously published study, we demonstrated that not all TF DNA-binding events result in gene expression (Coons et al., 2017). Specifically, we showed that only a small fraction of all sNR chromatin-interacting events observed in ChIPSeq and ChIPExo experiments is associated with transcriptional output (Li et al., 2011, John et al., 2011, Vockley et al., 2016, Coons et al., 2017), and this functionality is restricted to DNA elements that vary from the consensus palindromic DNA element by one or two nucleotides (nts), named *nuclear receptor functional enhancers* (NRFEs) (Coons et al., 2017). Thus, DNA sequence constraints define which sNR chromatin-interacting events in the genome are functionally active. This raises the question as to the purpose or cause of the remaining non-functional (non-NRFE) chromatin-interacting events observed in ChIPSeq and ChIPExo experiments. Or, assuming a purpose, what information is contained in these structures.

In this study, using publicly available ChIPSeq and ChIPExo experiments, we decode the inversion (reverse-complement) symmetry underlying TF binding to DNA elements in the genome, and elucidate its role in distinguishing functional from non-functional chromatin-interacting events. That is, why TFs bind the specific DNA elements in the genome where they do and whether that binding event ultimately affects gene expression. Specifically, we demonstrate that TF binding in the genome follows inversion symmetry (i.e., the number of TF binding events at a particular DNA element in the genome is equivalent to the number of TF binding events at its reverse-complement DNA element in the genome). In addition, the specific DNA elements where TFs bind in the genome are determined by internal inversion symmetry within the DNA element (i.e., TF DNA-binding is determined by the position of variants within the DNA element and its reverse-complement position). The basis of these DNA-binding rules follows specific algebraic relationships. Thus, these DNA-binding rules quantitatively define how TFs select the appropriate regulatory targets from a large number of similar DNA elements in the genome to elicit specific transcriptional and cellular responses. Importantly, we also demonstrate that these DNA-binding rules extend (i.e., are applicable) to DNA elements that do not support transcriptional activity. That is, the DNA-binding rules are obeyed, but the retention time of the TF at these non-functional DNA elements is not long enough to initiate and/or maintain transcription. Thus, functionality is determined at the individual nucleotide level, and the residence time (or strength of binding) is dictated by the number and position of variants within the DNA element (i.e., inversion symmetry and DNA sequence constraints). We further demonstrate that the population of every DNA element in the single-stranded genome (i.e., 1.4 trillion DNA elements) is equivalent to the population of its reverse-complement DNA element in the single-stranded genome. Therefore, the inversion symmetry observed for TF binding in the genome represents an inherent inversion symmetry structure for all DNA elements in the genome. This property is maintained at the level of each individual chromosome. These findings suggest that the structural mechanisms (by which inversion symmetry ascribes TF DNA-binding and functionality) are universally applicable. Hence, analysis of TF binding in the genome has expanded our understanding as to why the genome is organized in an inversion symmetry structure (i.e., Chargaff's second parity rule). Inversion symmetry is the DNA code that TFs use to interact with the genome, and dictates (in conjunction with known DNA sequence constraints) which of those interactions are functionally active. In addition, we further demonstrate why the inversion symmetry that underlies TF DNA-binding specificity and functionality in the genome, observed in hundreds of ChIPSeq and ChIPExo experiments, has not been detected using current DNA motif identification algorithms.

## Results

### Inversion Symmetry of sNR DNA-Binding at NRFEs in the Genome

Previously, we reported that NRFE EREs and HREs are composed of their consensus palindromic DNA element and those DNA elements that vary from their consensus palindromic DNA element by one or two nts (Coons et al., 2017). In the present study, we evaluated the DNA-binding preference of sNRs at each of the 30 DNA elements that make up the 1-nt variant group (i.e., DNA elements generated by varying the 0-nt variant consensus palindromic DNA element by one nt) in the genome. That is, the 3 alternative nucleotide possibilities (variants) in each of the 10 primary positions of the 13-nt consensus palindromic ERE (5′-GGTCAnnnTGACC-3′) and HRE (5′-GAACAnnnTGTTC-3′) DNA element.

**Figure 1:**
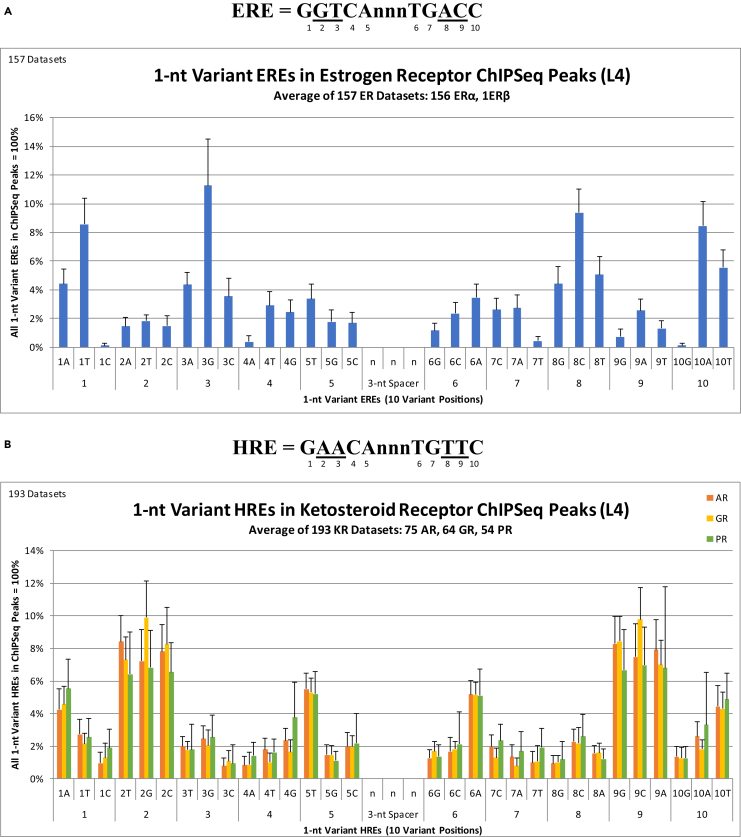
Inversion Symmetry of sNR DNA-Binding at 1-nt Variant DNA Elements in the Genome (A) Average distribution of 1-nt variant ERE DNA elements in ER experiments (156 ERα and 1 ERβ) at the 30 1-nt variant ERE DNA elements in the genome (the total number of 1-nt variant ERE DNA elements contained within an experiment = 100%). (B) Average distribution of 1-nt variant HRE DNA elements in KR experiments (75 AR, 64 GR, 54 PR) at the 30 1-nt variant HRE DNA elements in the genome (the total number of 1-nt variant HRE DNA elements contained within an experiment = 100%). These 30 1-nt variant EREs or HREs are defined by 10 variant positions (positions 1 through 5, followed by their reverse-complements).

First, the absolute number of times each 1-nt variant DNA element occurred in a ChIPSeq or ChIPExo experiment were counted. This analysis was completed by overlapping the location coordinates of every 1-nt variant DNA element in the genome and the location coordinates of the ChIPSeq or ChIPExo peaks in an experiment. The absolute number of times each 1-nt variant DNA element occurred in an experiment was then converted to a proportion or percent of the total number of 1-nt variant DNA elements contained within that ChIPSeq or ChIPExo experiment (i.e., the total number of 1-nt variant DNA elements contained within an experiment = 100%). Taking the average of ER DNA-binding at 1-nt variant ERE DNA elements in the genome from 157 ER experiments, representing a wide variety of mouse tissues and human cell lines, reveals considerable variability in the ability of ER to bind the 30 DNA elements that make up the 1-nt variant ERE group (Figure 1A). Of particular importance, this analysis demonstrates that ER DNA-binding at 1-nt variant ERE DNA elements in the genome follows inversion symmetry (Figure 1A). That is, the number of ER DNA-binding events at a particular 1-nt variant ERE DNA element in the genome is equivalent to the number of ER DNA-binding events at its reverse-complement DNA element in the genome. For example, the number of ER DNA-binding events at the 1T ERE DNA element (i.e., the variant thymidine [T] is in the first [1] position [5′-TGTCAnnnTGACC-3′]) is equivalent to the number of ER DNA-binding events at the 10A ERE DNA element (i.e., the variant adenine [A] is in the tenth [10] position [5′-GGTCAnnnTGACA-3′]) in the genome (Figure 1A). Accordingly, the number of ER DNA-binding events at DNA elements where a variant is in position 2 is equivalent to the number of ER DNA-binding events at DNA elements where a variant is in position 9 (Figure 1A). This equivalency between the number of ER DNA-binding events at a particular DNA element and its reverse-complement DNA element occurs for all five palindromic position pairs: 1-10, 2-9, 3-8, 4-7, 5-6 (Figure 1A). Furthermore, the number of ER DNA-binding events is greatest at 1-nt variant ERE DNA elements that (1) vary position 3 (or its reverse-complement position 8) to any nucleotide, and (2) vary position 1 (or its reverse-complement position 10) to either an A or a T (Figure 1A). The small standard deviation measured across these 157 ER experiments confirms that the inversion symmetry of ER DNA-binding at 1-nt variant ERE DNA elements in the genome is a universal property for all tissues, primary cell lines, and cancer cell lines tested to date (Figure 1A). Note: all detailed data and statistics associated with every figure are compiled in Data S1.

Similarly, taking the average of AR, GR, and PR DNA-binding at 1-nt variant HRE DNA elements in the genome from 193 KR experiments, representing a wide variety of mouse tissues and human cell lines, demonstrates that KR DNA-binding at 1-nt variant HRE DNA elements in the genome also follows inversion symmetry (Figure 1B). Furthermore, this analysis reveals a strikingly similar DNA-binding profile between the different KRs at 1-nt variant HRE DNA elements in the genome, suggesting a shared DNA-binding mechanism between all members of the KR family (Figure 1B). In contrast to ER DNA-binding at 1-nt variant ERE DNA elements, the number of KR DNA-binding events is greatest at 1-nt variant HRE DNA elements that (1) vary position 2 (or its reverse-complement position 9) to any nucleotide, (2) vary position 1 (or its reverse-complement position 10) to a 1A or 10T, and (3) vary position 5 (or its reverse-complement position 6) to a 5T or 6A (Figure 1B). Therefore, from an evolutionary perspective, the increased DNA-binding at 1-nt variant DNA elements in the genome changed from position 3 (or its reverse-complement position 8) in the ERE (by the ER) to position 2 (or its reverse-complement position 9) in the HRE (by the KRs), and the increased DNA-binding of ER when position 1 (or its reverse-complement position 10) is varied to a 1T or 10A in the ERE was transferred to position 5 (or its reverse-complement position 6) in the HRE by the KRs. The small standard deviation measured across these 193 KR experiments confirms that the inversion symmetry of KR DNA-binding at 1-nt variant HRE DNA elements in the genome is a universal property for all tissues, primary cell lines, and cancer cell lines tested to date (Figure 1B).

The inversion symmetry of sNR DNA-binding observed at 1-nt variant DNA elements in the genome (i.e., the number of sNR DNA-binding events at a particular 1-nt variant DNA element is equivalent to the number of sNR DNA-binding events at its reverse-complement DNA element) also occurs for sNR DNA- binding at 2-nt variant DNA elements in the genome (Figure S1). There are 405 DNA elements in the 2-nt variant group (i.e., DNA elements generated by varying the 0-nt variant consensus palindromic DNA element by two nts) (Figure S1). In addition, the increased DNA-binding of ER at DNA elements with variants in palindromic position pair 3-8 and 1-10 of the ERE, and increased DNA-binding of KR at DNA elements with variants in palindromic position pair 2-9 of the HRE also occurs for sNR DNA-binding at 2-nt variant ERE and HRE DNA elements in the genome (Figure S1). Thus, sNR DNA-binding at NRFEs in the genome follows inversion symmetry (i.e., the number of sNR DNA-binding events at a particular DNA element in the genome is equivalent to the number of sNR DNA-binding events at its reverse-complement DNA element in the genome). In addition, sNRs exhibit preferential DNA-binding at specific NRFE DNA elements in the genome; this preference is determined by internal inversion symmetry within the DNA element (i.e., sNR binding in the genome is determined by the position of variants within the DNA element and its reverse-complement position).

### sNR DNA-Binding Is Most Predominant at NRFEs in the Genome

We previously demonstrated that NRFEs (composed of the 0-nt variant consensus palindromic DNA element, 1-nt variant DNA elements, and 2-nt variant DNA elements) constitute ∼45% of the chromatin-interacting events in ER experiments and ∼35% of the chromatin-interacting events in KR experiments (Coons et al., 2017). Thus, ∼55% and ∼65% of the chromatin-interacting events detected in ER and KR ChIPSeq and ChIPExo experiments are non-functional (i.e., non-NRFEs) (Coons et al., 2017). This raises the question as to the purpose or cause of this large amount of non-functional (i.e., non-NRFE) TF interaction with the genome. Or, assuming a purpose, what information is contained in these structures.

In the past, it has been shown that ERE and HRE “palindrome half-sites” (i.e., the left-hand or right-hand side of the symmetrical 0-nt variant consensus palindromic DNA element: GGTCA or TGACC in the ERE, GAACA or TGTTC in the HRE) are an enriched feature observed in sNR ChIPSeq and ChIPExo experiments, and that these sites can support hormone-dependent transcription (Fan et al., 1996, Norris et al., 1995). To further explore the interactions of sNRs with these DNA elements, we obtained the location coordinates of the 81,922 DNA elements that contain 0- to 5-nt variants relative to the 0-nt variant consensus palindromic DNA element in the mouse and human genome (i.e., a 5-nt variant = a half-site) (Table 1). Thus, we are expanding the concept of a “half-site” to include all 5-nt variant DNA elements, not just the “palindrome half-sites.” The 0- to 5-nt variant DNA elements include the 1 0-nt variant consensus palindromic DNA element, 30 1-nt variant DNA elements, 405 2-nt variant DNA elements, 3,240 3-nt variant DNA elements, 17,010 4-nt variant DNA elements, and 61,236 5-nt variant DNA elements, for a total of 81,922 DNA elements (Table 1).

**Table 1:** Number of 0- to 5-nt Variant 13-nt ERE and HRE DNA Elements in the Mouse and Human Genome

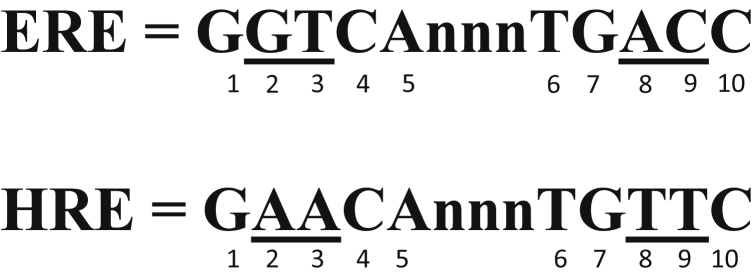									
	DNA Element	Combinatorial Counts				DNA Element Frequency in Genome			
A	B	C	D	E	F	Mouse Genome (mm10)		Human Genome (hg19)	
k	ERE (*n* = 10)	Combinations C = $\left(\begin{array}{c} n\\ k \end{array}\right)$	4-nts D = C x $4^{k}$	Unique E = C x $3^{k}$	Total Unique F = ΣE	Unique	Total Unique	Unique	Total Unique
0	0-nt Variant ERE	1	1	1	1	2,367		2,194	
1	1-nt Variant ERE	10	40	30	31	71,428	73,795	60,313	62,507
2	2-nt Variant ERE	45	720	405	436	898,155	971,950	914,726	977,233
3	3-nt Variant ERE	120	7,680	3,240	3,676	6,750,607	7,722,557	7,516,184	8,493,417
4	4-nt Variant ERE	210	53,760	17,010	20,686	35,508,190	43,230,747	38,222,674	46,716,091
5	5-nt Variant ERE	252	258,048	61,236	81,922	134,732,965	177,963,712	141,280,093	187,996,184

A	B	C	D	E	F	Mouse Genome (mm10)		Human Genome (hg19)	
k	HRE (*n* = 10)	Combinations C = $\left(\begin{array}{c} n\\ k \end{array}\right)$	4-nts D = C x $4^{k}$	Unique E = C x $3^{k}$	Total Unique F = ΣE	Unique	Total Unique	Unique	Total Unique
0	0-nt Variant HRE	1	1	1	1	3,444		3,535	
1	1-nt Variant HRE	10	40	30	31	97,039	100,483	104,767	108,302
2	2-nt Variant HRE	45	720	405	436	1,337,516	1,437,999	1,339,543	1,447,845
3	3-nt Variant HRE	120	7,680	3,240	3,676	10,461,197	11,899,196	10,771,159	12,219,004
4	4-nt Variant HRE	210	53,760	17,010	20,686	49,391,434	61,290,630	54,136,564	66,355,568
5	5-nt Variant HRE	252	258,048	61,236	81,922	169,440,498	230,731,128	187,469,898	253,825,466

A	B	C	D	E	F				
k		Combinations C = $\left(\begin{array}{c} n\\ k \end{array}\right)$	4-nts D = C x $4^{k}$	Unique E = C x $3^{k}$	Total Unique F = ΣE				
0	0-nt Variant	10!/(0! x 10!)	1 x 1	1 x 1	1 + 0				
1	1-nt Variant	10!/(1! x 9!)	10 x 4	10 x 3	30 + 1				
2	2-nt Variant	10!/(2! x 8!)	45 x 16	45 x 9	405 + 31				
3	3-nt Variant	10!/(3! x 7!)	120 x 64	120 x 27	3,240 + 436				
4	4-nt Variant	10!/(4! x 6!)	210 x 256	210 x 81	17,010 + 3,676				
5	5-nt Variant	10!/(5! x 5!)	252 x 1,024	252 x 243	61,236 + 20,686				

The number of 0- to 5-nt variants of the 13-nt ERE and HRE consensus palindromic DNA element on the positive or sense strand in the mouse (mm10) and human (hg19) genome. Here the 13-nt ERE is the estrogen response element (5′-GGTCAnnnTGACC-3′) and the 13-nt HRE is the hormone response element (5′-GAACAnnnTGTTC-3′). The 0- to 5-nt variant 13-nt ERE and HRE DNA elements include the 1 0-nt variant consensus palindromic DNA element, 30 1-nt variant DNA elements (10 variant positions), 405 2-nt variant DNA elements (45 variant positions), 3,240 3-nt variant DNA elements (120 variant positions), 17,010 4-nt variant DNA elements (210 variant positions), and 61,236 5-nt variant DNA elements (252 variant positions), for a total of 81,922 DNA elements. The population count of each of the 81,922 0- to 5-nt variant 13-nt ERE and HRE DNA elements in the mouse (mm10) and human (hg19) genome can be found in Tables S55–S58.

The analysis done in our previously published study counted the number of ChIPSeq or ChIPExo peaks that contained a 0- to 3-nt variant ERE or HRE DNA element, with peak assignment given to the 0-nt variant consensus palindromic DNA element or the DNA element with the least number of variants relative to the 0-nt variant consensus palindromic DNA element (i.e., defining each peak by a single DNA element with the total number of peaks in an experiment = 100%) (Coons et al., 2017). In this study, the absolute number of times a 0- to 5-nt variant DNA element occurred in a ChIPSeq or ChIPExo experiment were counted and used in a signal-to-noise ratio (S/N) analysis. Counting the absolute number of times every 0- to 5-nt variant DNA element occurred in an experiment eliminates any bias toward DNA elements with the least number of variants in individual peaks that contain multiple ERE or HRE DNA elements. That is, the occurrence of all 0- to 5-nt variant DNA elements was counted, because the preset hierarchal peak selection criteria based on the number of variants is eliminated.

The (S/N) is the absolute number of times a 0- to 5-nt variant DNA element occurs in an experiment (defined by the total number of peaks in the experiment and the peak length) compared with the random frequency expectation of that DNA element occurring in the genome (i.e., the probability that any 10-nt DNA element that has a maximum possibility of 4 nts in each position will occur in the genome is once every 1,048,576 nts (4^10^) at random occurrence). (S/N) analysis demonstrates that the number of ER DNA-binding events (i.e., the ER DNA-binding signal) is greatest at the 0-nt variant consensus palindromic ERE DNA element, followed by DNA-binding at 1-nt variant ERE DNA elements, followed by DNA-binding at 2-nt variant ERE DNA elements in the genome (Figure S2). In contrast, there is little to no increase of ER DNA-binding at 3- to 5-nt variant ERE DNA elements above what would be expected with respect to random noise (i.e., random = 0 and 0 = log(1)) (Figure S2). This DNA-binding profile was observed in 157 ER experiments, representing a wide variety of mouse tissues and human cell lines (Figure S2). This DNA-binding profile was also observed across multiple peak selection criteria (L4, L8, L10, L15, L20), where Lx represents an x-fold greater tag density at peaks than in the surrounding 10-kb region (i.e., performing a low-to-high stringency analysis of the ChIPSeq and ChIPExo data) (Figure S2). Likewise, this DNA-binding profile was observed in 194 KR experiments at 0- to 5-nt variant HRE DNA elements in the genome (Figure S3). This suggests that there is little to no increase in sNR DNA-binding at 3- to 5-nt variant ERE or HRE DNA elements in the genome (when analyzed by number of variants) above what would be expected to occur at random. Note: the relative (S/N) values of sNR DNA-binding signals are scale invariant (i.e., the relative ratios between the 0- to 5-nt variant groups are constant) (Figures S2 and S3).

### Sequentially Track sNR DNA-Binding at 0- to 5-nt Variant DNA Elements in the Genome

To evaluate the DNA-binding of sNRs at every DNA element in the 0- to 5-nt variant groups, the absolute number of times each of the 81,922 0- to 5-nt variant DNA elements (Table 1) occurred in an experiment was counted. For display purposes, we have defined a 5-nt variant ERE and HRE DNA element by its five fixed positions, resulting in 252 half-site groups (i.e., categorizing the 61,236 5-nt variant DNA elements into 252 half-site groups, defined by the five positions that are fixed/not varied) (Table 1). Thus, the “palindrome half-sites” make up two of these 252 half-site groups (i.e., positions 1 through 5 or positions 6 through 10 of the 0-nt variant consensus palindromic DNA element are fixed/not varied).

The 252 half-site groups are symmetrically split into a set of 126 groups and their 126 reverse-complements (Table 2). These 126 groups further split into three distinct subgroups (zero vacancies, one vacancy, two vacancies) depending on how many reverse-complement vacancies are in the DNA element (Table 2), that is, the information for which nucleotide occupies each of the 10 primary positions of the 0-nt variant consensus palindromic DNA element is missing/replaced by variants/vacant in the position and its reverse-complement position (i.e., its palindromic position pair). For example, the half-site group 1-2-3-4-5 (variants in positions 6-7-8-9-10) has zero vacancies because the information for which nucleotide occupies each position is present in either the position (positions 1 through 5) or its reverse-complement position (positions 6 through 10) (Table 2). In contrast, the half-site group 1-2-4-5-10 (variants in positions 3-6-7-8-9) has one vacancy because the information for which nucleotide occupies position 3 is missing/replaced by variants/vacant in the position (position 3) and its reverse-complement position (position 8) (Table 2). Of these 126 groups, 16 have zero vacancies, 80 have one vacancy, and 30 have two vacancies (Table 2). The reverse-complement vacancy position ID indicates which of the five palindromic position pairs (i.e., 1-10, 2-9, 3-8, 4-7, 5-6) is missing/replaced by variants/vacant (Table 2).

**Table 2:** The 252 Half-Site Groups Symmetrically Split into 126 Groups and Their Reverse-Complements

	A	B	C	D	E
	Fixed Half-Site Positions	Reverse-Complement Vacancies & Double Occupants	Reverse-Complement Vacancy Position ID	Reverse-Complement Double Occupant Position ID	Positions That Can Be Varied
**Zero Vacancies**					

1	1-2-3-4-5-----	0V			-----6-7-8-9-10
2	1-2--4-5---8--	0V			--3---6-7--9-10
3	-2-3-4-5-----10	0V			1-----6-7-8-9-
4	1-2-3-4--6----	0V			----5--7-8-9-10
5	1-2-3--5--7---	0V			---4--6--8-9-10
6	1--3-4-5----9-	0V			-2----6-7-8--10
7	-2--4-5---8--10	0V			1--3---6-7--9-
8	1-2--4--6--8--	0V			--3--5--7--9-10
9	1-2---5--7-8--	0V			--3-4--6---9-10
10	1---4-5---8-9-	0V			-2-3---6-7---10
11	-2-3-4--6----10	0V			1----5--7-8-9-
12	-2-3--5--7---10	0V			1---4--6--8-9-
13	--3-4-5----9-10	0V			1-2----6-7-8--
14	1-2-3---6-7---	0V			---4-5---8-9-10
15	1--3-4--6---9-	0V			-2---5--7-8--10
16	1--3--5--7--9-	0V			-2--4--6--8--10

**One Vacancy**					

17	1-2--4-5-----10	1V	3	1	--3---6-7-8-9-
18	1-2--4--6----10	1V	3	1	--3--5--7-8-9-
19	1-2---5--7---10	1V	3	1	--3-4--6--8-9-
20	1---4-5----9-10	1V	3	1	-2-3---6-7-8--
21	1-2--4-5-6----	1V	3	5	--3----7-8-9-10
22	-2--4-5-6----10	1V	3	5	1--3----7-8-9-
23	1-2---5-6-7---	1V	3	5	--3-4----8-9-10
24	1---4-5-6---9-	1V	3	5	-2-3----7-8--10
25	1-2--4-5--7---	1V	3	4	--3---6--8-9-10
26	-2--4-5--7---10	1V	3	4	1--3---6--8-9-
27	1-2--4--6-7---	1V	3	4	--3--5---8-9-10
28	1---4-5--7--9-	1V	3	4	-2-3---6--8--10
29	1-2--4-5----9-	1V	3	2	--3---6-7-8--10
30	-2--4-5----9-10	1V	3	2	1--3---6-7-8--
31	1-2--4--6---9-	1V	3	2	--3--5--7-8--10
32	1-2---5--7--9-	1V	3	2	--3-4--6--8--10
33	-2-3-4-5---8--	1V	1	3	1-----6-7--9-10
34	-2-3-4--6--8--	1V	1	3	1----5--7--9-10
35	-2-3--5--7-8--	1V	1	3	1---4--6---9-10
36	--3-4-5---8-9-	1V	1	3	1-2----6-7---10
37	-2-3-4-5-6----	1V	1	5	1------7-8-9-10
38	-2--4-5-6--8--	1V	1	5	1--3----7--9-10
39	-2-3--5-6-7---	1V	1	5	1---4----8-9-10
40	--3-4-5-6---9-	1V	1	5	1-2-----7-8--10
41	-2-3-4-5--7---	1V	1	4	1-----6--8-9-10
42	-2--4-5--7-8--	1V	1	4	1--3---6---9-10
43	-2-3-4--6-7---	1V	1	4	1----5---8-9-10
44	--3-4-5--7--9-	1V	1	4	1-2----6--8--10
45	-2-3-4-5----9-	1V	1	2	1-----6-7-8--10
46	-2--4-5---8-9-	1V	1	2	1--3---6-7---10
47	-2-3-4--6---9-	1V	1	2	1----5--7-8--10
48	-2-3--5--7--9-	1V	1	2	1---4--6--8--10
49	1-2-3-4----8--	1V	5	3	----5-6-7--9-10
50	-2-3-4----8--10	1V	5	3	1----5-6-7--9-
51	1-2-3----7-8--	1V	5	3	---4-5-6---9-10
52	1--3-4----8-9-	1V	5	3	-2---5-6-7---10
53	1-2-3-4------10	1V	5	1	----5-6-7-8-9-
54	1-2--4----8--10	1V	5	1	--3--5-6-7--9-
55	1-2-3----7---10	1V	5	1	---4-5-6--8-9-
56	1--3-4-----9-10	1V	5	1	-2---5-6-7-8--
57	1-2-3-4---7---	1V	5	4	----5-6--8-9-10
58	1-2--4---7-8--	1V	5	4	--3--5-6---9-10
59	-2-3-4---7---10	1V	5	4	1----5-6--8-9-
60	1--3-4---7--9-	1V	5	4	-2---5-6--8--10
61	1-2-3-4-----9-	1V	5	2	----5-6-7-8--10
62	1-2--4----8-9-	1V	5	2	--3--5-6-7---10
63	-2-3-4-----9-10	1V	5	2	1----5-6-7-8--
64	1-2-3----7--9-	1V	5	2	---4-5-6--8--10
65	1-2-3--5---8--	1V	4	3	---4--6-7--9-10
66	-2-3--5---8--10	1V	4	3	1---4--6-7--9-
67	1-2-3---6--8--	1V	4	3	---4-5--7--9-10
68	1--3--5---8-9-	1V	4	3	-2--4--6-7---10
69	1-2-3--5-----10	1V	4	1	---4--6-7-8-9-
70	1-2---5---8--10	1V	4	1	--3-4--6-7--9-
71	1-2-3---6----10	1V	4	1	---4-5--7-8-9-
72	1--3--5----9-10	1V	4	1	-2--4--6-7-8--
73	1-2-3--5-6----	1V	4	5	---4---7-8-9-10
74	1-2---5-6--8--	1V	4	5	--3-4---7--9-10
75	-2-3--5-6----10	1V	4	5	1---4---7-8-9-
76	1--3--5-6---9-	1V	4	5	-2--4---7-8--10
77	1-2-3--5----9-	1V	4	2	---4--6-7-8--10
78	1-2---5---8-9-	1V	4	2	--3-4--6-7---10
79	-2-3--5----9-10	1V	4	2	1---4--6-7-8--
80	1-2-3---6---9-	1V	4	2	---4-5--7-8--10
81	1--3-4-5---8--	1V	2	3	-2----6-7--9-10
82	--3-4-5---8--10	1V	2	3	1-2----6-7--9-
83	1--3-4--6--8--	1V	2	3	-2---5--7--9-10
84	1--3--5--7-8--	1V	2	3	-2--4--6---9-10
85	1--3-4-5-----10	1V	2	1	-2----6-7-8-9-
86	1---4-5---8--10	1V	2	1	-2-3---6-7--9-
87	1--3-4--6----10	1V	2	1	-2---5--7-8-9-
88	1--3--5--7---10	1V	2	1	-2--4--6--8-9-
89	1--3-4-5-6----	1V	2	5	-2-----7-8-9-10
90	1---4-5-6--8--	1V	2	5	-2-3----7--9-10
91	--3-4-5-6----10	1V	2	5	1-2-----7-8-9-
92	1--3--5-6-7---	1V	2	5	-2--4----8-9-10
93	1--3-4-5--7---	1V	2	4	-2----6--8-9-10
94	1---4-5--7-8--	1V	2	4	-2-3---6---9-10
95	--3-4-5--7---10	1V	2	4	1-2----6--8-9-
96	1--3-4--6-7---	1V	2	4	-2---5---8-9-10

**Two Vacancies**					

97	-2--4-5--7--9-	2V	1–3	2–4	1--3---6--8--10
98	-2--4-5-6---9-	2V	1–3	2–5	1--3----7-8--10
99	-2--4-5-6-7---	2V	1–3	4–5	1--3-----8-9-10
100	1-2--4---7--9-	2V	3–5	2–4	--3--5-6--8--10
101	1-2--4-----9-10	2V	3–5	1–2	--3--5-6-7-8--
102	1-2--4---7---10	2V	3–5	1–4	--3--5-6--8-9-
103	1-2---5-6---9-	2V	3–4	2–5	--3-4---7-8--10
104	1-2---5----9-10	2V	3–4	1–2	--3-4--6-7-8--
105	1-2---5-6----10	2V	3–4	1–5	--3-4---7-8-9-
106	1---4-5-6-7---	2V	2–3	4–5	-2-3-----8-9-10
107	1---4-5--7---10	2V	2–3	1–4	-2-3---6--8-9-
108	1---4-5-6----10	2V	2–3	1–5	-2-3----7-8-9-
109	-2-3-4---7--9-	2V	1–5	2–4	1----5-6--8--10
110	-2-3-4----8-9-	2V	1–5	2–3	1----5-6-7---10
111	-2-3-4---7-8--	2V	1–5	3–4	1----5-6---9-10
112	-2-3--5-6---9-	2V	1–4	2–5	1---4---7-8--10
113	-2-3--5---8-9-	2V	1–4	2–3	1---4--6-7---10
114	-2-3--5-6--8--	2V	1–4	3–5	1---4---7--9-10
115	--3-4-5-6-7---	2V	1–2	4–5	1-2------8-9-10
116	--3-4-5--7-8--	2V	1–2	3–4	1-2----6---9-10
117	--3-4-5-6--8--	2V	1–2	3–5	1-2-----7--9-10
118	1-2-3------9-10	2V	4–5	1–2	---4-5-6-7-8--
119	1-2-3-----8-9-	2V	4–5	2–3	---4-5-6-7---10
120	1-2-3-----8--10	2V	4–5	1–3	---4-5-6-7--9-
121	1--3-4---7---10	2V	2–5	1–4	-2---5-6--8-9-
122	1--3-4---7-8--	2V	2–5	3–4	-2---5-6---9-10
123	1--3-4----8--10	2V	2–5	1–3	-2---5-6-7--9-
124	1--3--5-6----10	2V	2–4	1–5	-2--4---7-8-9-
125	1--3--5-6--8--	2V	2–4	3–5	-2--4---7--9-10
126	1--3--5---8—10	2V	2–4	1–3	-2--4--6-7--9-

Column A: Categorizing the 61,236 5-nt variant DNA elements into 252 half-site groups, defined by the five positions that are fixed/not varied. The 252 half-site groups are symmetrically split into a set of 126 groups and their 126 reverse-complements. Column B: These 126 groups further split into three distinct subgroups (zero vacancies, one vacancy, two vacancies) depending on how many reverse-complement vacancies are in the DNA element (i.e., the information for which nucleotide occupies each of the 10 primary positions of the 0-nt variant consensus palindromic DNA element is missing/replaced by variants/vacant in the position and its reverse-complement position [its palindromic position pair]). Of these 126 groups, 16 have zero vacancies, 80 have one vacancy, and 30 have two vacancies. Column C: The reverse-complement vacancy position ID indicates which of the five palindromic position pairs (i.e., 1-10, 2-9, 3-8, 4-7, 5-6) are missing/replaced by variants/vacant. Column B: The number of reverse-complement vacancies equals the number of reverse-complement double occupants in the DNA element (i.e., the information for which nucleotide occupies each of the 10 primary positions of the 0-nt variant consensus palindromic DNA element is occupied in the position and its reverse-complement position [its palindromic position pair]). Column D: The reverse-complement double occupant position ID indicates which of the five palindromic position pairs (i.e., 1-10, 2-9, 3-8, 4-7, 5-6) are doubly occupied. Column E: The positions that are replaced by variants or not fixed for 5-nt variant DNA elements, and may be replaced by variants for 0-nt to 4-nt variant DNA elements.

Furthermore, the number of reverse-complement vacancies equals the number of reverse-complement double occupants (zero double occupants, one double occupant, two double occupants) that occur in the DNA element (Table 2), that is, the information for which nucleotide occupies each of the 10 primary positions of the 0-nt variant consensus palindromic DNA element is occupied in the position and its reverse-complement position. For example, the half-site group 2-4-5-7-9 (variants in positions 1-3-6-8-10) has two reverse-complement vacancies because the information for which nucleotide occupies position 1 and position 3 is missing/replaced by variants/vacant in the position (position 1 and position 3) and its reverse-complement position (position 8 and position 10) (Table 2). This half-site group, 2-4-5-7-9, also has two reverse-complement double occupants because the information for which nucleotide occupies position 2 and position 4 is occupied in the position (position 2 and position 4) and its reverse-complement position (position 7 and position 9) (Table 2). The reverse-complement double occupant position ID indicates which of the five palindromic position pairs (i.e., 1-10, 2-9, 3-8, 4-7, 5-6) is doubly occupied (Table 2).

The remaining 0- to 4-nt variant DNA elements (1 0-nt variant consensus palindromic DNA element, 30 1-nt variant DNA elements, 405 2-nt variant DNA elements, 3,240 3-nt variant DNA elements, and 17,010 4-nt variant DNA elements, for a total of 20,686 DNA elements) can be categorized into these same 252 half-site groups (i.e., five positions are fixed, allowing for *up to* five positions to be varied) (Table 1). For example, the half-site group 1-2-3-4-5 contains all 1-nt variant DNA elements that vary positions 6 through 10 (Table 2). In the case of the ERE, this includes the following 1-nt variant ERE DNA elements: 6A, 6C, 6G, 7A, 7C, 7T, 8C, 8G, 8T, 9A, 9G, 9T, 10A, 10G, 10T (Tables S1 and S3). Thus, each of the 252 half-site groups contain 1 (0-nt variant consensus palindromic DNA element), 15 (1-nt variant DNA elements), 90 (2-nt variant DNA elements), 270 (3-nt variant DNA elements), 405 (4-nt variant DNA elements), and 243 (5-nt variant DNA elements), for a total of 1,024 0- to 5-nt variant DNA elements per half-site group (Tables S1–S4). This allows all 81,922 0-nt to 5-nt variant DNA elements to be categorized into the 252 half-site groups, thus providing the ability to sequentially track sNR DNA-binding at all 81,922 0-nt to 5-nt variant ERE or HRE DNA elements in the genome.

### Inversion Symmetry of sNR DNA-Binding Continues through 5-nt Variant DNA Elements in the Genome

**Figure 2:**
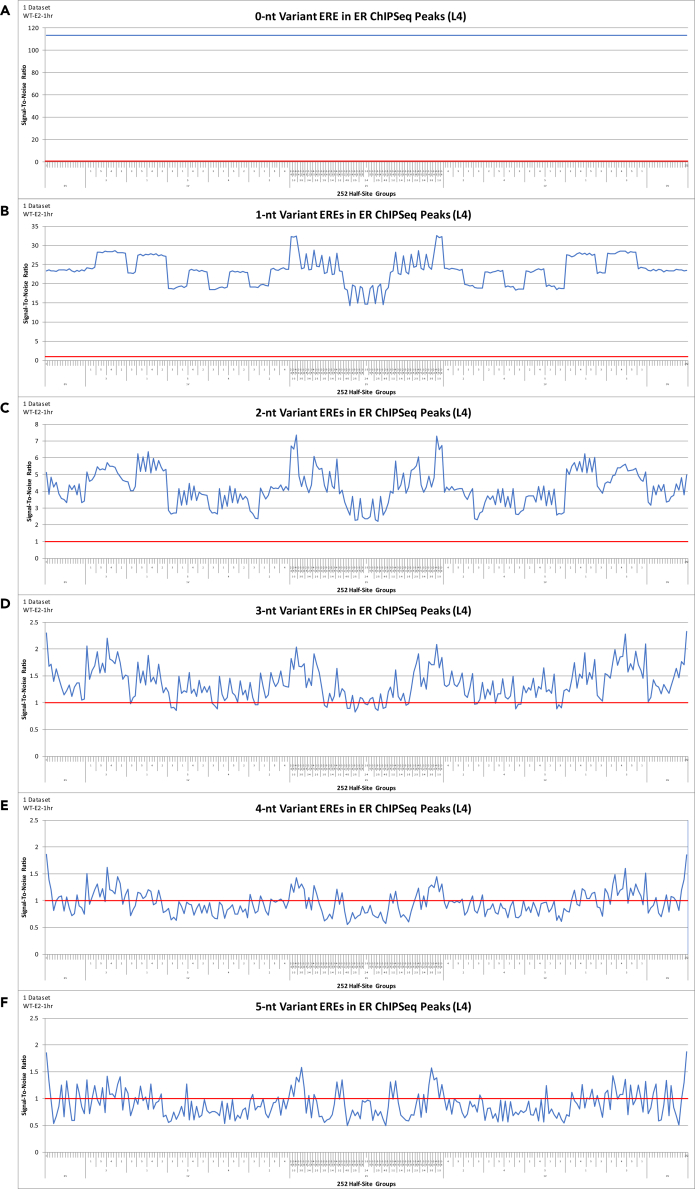
(S/N) Analysis of 0- to 5-nt Variant ERE DNA Elements in ER ChIPSeq Peaks (A–F) (S/N) analysis of 0-nt to 5-nt variant ERE DNA elements (displayed by the 252 half-site groups) in ER (WT-E2-1hr) (76,163 peaks, 146-nt peak length) ChIPSeq peaks. Upper and lower one-tailed Poisson significance thresholds at p < 0.001 (A) 0.19, 2.07; (B) 0.77, 1.25; (C) 0.90, 1.10; (D) 0.94, 1.06; (E) 0.95, 1.05; and (F) 0.94, 1.06. X-axis order = reverse-complement vacancy position ID 3-8 > 1-10 > 5-6 > 4-7 > 2-9. See Table S5 for x-axis details. See Tables S64–S67 for step-by-step instructions of the data analysis from peak selection to (S/N) analysis of ER DNA-binding at 81,922 0-nt to 5-nt variant ERE DNA elements in the genome, displayed by the 252 half-site groups.

**Figure 3:**
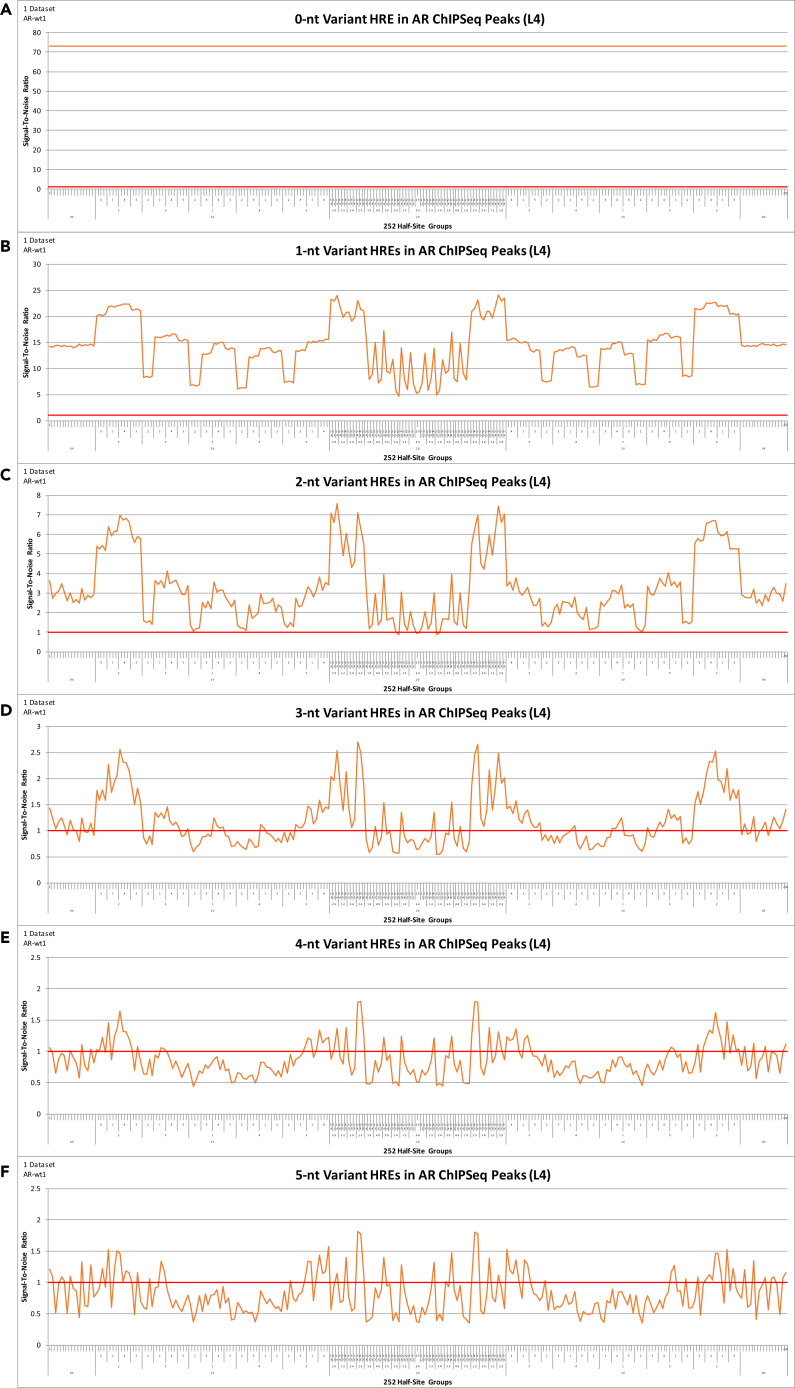
(S/N) Analysis of 0- to 5-nt Variant HRE DNA Elements in AR ChIPSeq Peaks (A–F) (S/N) analysis of 0- to 5-nt variant HRE DNA elements (displayed by the 252 half-site groups) in AR (AR-wt1) (49,859 peaks, 136-nt peak length) ChIPSeq peaks. Upper and lower one-tailed Poisson significance thresholds at p < 0.001 (A) 0.00, 2.47; (B) 0.70, 1.33; (C) 0.87, 1.13; (D) 0.93, 1.08; (E) 0.94, 1.06; and (F) 0.92, 1.08. X-axis order = reverse-complement vacancy position ID 2-9 > 5-6 > 1-10 > 4-7 > 3-8. See Table S6 for x-axis details.

(S/N) analysis of sNR DNA-binding at 0- to 5-nt variant DNA elements in the genome (displayed by the 252 half-site groups) reveals a highly structured symmetrical DNA-binding profile that decays from 0- to 5-nt variants: ER at 0- to 5-nt variant ERE DNA elements (Figure 2) and KR at 0- to 5-nt variant HRE DNA elements (Figures 3, S4, and S5). The x-axis is ordered by the symmetrically split 126 groups (left-to-right: zero vacancies, one vacancy, two vacancies) followed by their 126 reverse-complements. The x-axis is labeled by the reverse-complement vacancy position ID (primary label) and reverse-complement double occupant position ID (secondary label) (Figures 2, 3, S4, and S5). The order of the reverse-complement vacancy position IDs for the ERE is 3-8 > 1-10 > 5-6 > 4-7 > 2-9 (Table S5), whereas that for the HRE is 2-9 > 5-6 > 1-10 > 4-7 > 3-8 (Table S6). Note: this analysis includes upper and lower one-tailed Poisson significance thresholds at p < 0.001; thus the probability of the TF DNA-binding signal occurring outside these boundaries by chance is less than one in a thousand.

This highly structured symmetrical sNR DNA-binding profile (i.e., the left side [126 half-site groups] versus the right side [126 reverse-complements]) demonstrates that there is no sNR DNA-binding bias toward either the 5′ side (GGTCA or GAACA) or the 3′ side (TGACC or TGTTC) of the DNA element at any 1- to 5-nt variant ERE or HRE DNA elements in the genome (Figures 2, 3, S4, and S5). This symmetrical sNR DNA-binding profile also confirms that sNR binding in the genome follows inversion symmetry through 5-nt variant DNA elements (Figures 2, 3, S4, and S5). That is, the number of sNR DNA-binding events at a 1- to 5-nt variant DNA element in the genome is equivalent to the number of sNR DNA-binding events at its reverse-complement DNA element in the genome (Figures 2, 3, S4, and S5).

Furthermore, this highly structured symmetrical sNR DNA-binding profile demonstrates that the specific DNA elements where sNRs bind in the genome is determined by internal inversion symmetry within the DNA element, and this continues through 5-nt variant DNA elements (Figures 2, 3, S4, and S5). That is, sNRs exhibit preferential DNA-binding at particular DNA elements in the genome, and this preference is determined by internal inversion symmetry within the DNA element (i.e., sNR binding in the genome is determined by the position of variants within the DNA element and its reverse-complement position) (Figures 2, 3, S4, and S5). Thus, the specific 0- to 5-nt variant DNA elements that sNRs bind in the genome is determined by the position of variants within the DNA element and its reverse-complement position (Figures 2, 3, S4, and S5).

In addition, sNR DNA-binding at “palindrome half-sites” (half-site group #1 and half-side group #252 on the x-axis) are not particularly distinct compared with the other 250 half-site groups (Figures 2, 3, S4, and S5). Because sNR DNA-binding at 0- to 5-nt variant DNA elements in the genome is a progression that decays from 0- to 5-nt variant DNA elements, this confirms that sNR DNA-binding at 0- to 5-nt variant DNA elements in the genome requires all the information contained within the entire DNA element (i.e., on both sides of the 3-nt spacer) (Figures 2, 3, S4, and S5). Thus, the information is stored in the “complex” as a whole, not in simple physical subsets of the DNA element. This analysis therefore challenges previous suggestions that sNRs bind “palindrome half-sites” in the genome as individual monomers (Figures 2, 3, S4, and S5). The two endpoints (half-site group #1 and half-site group #252 on the x-axis) represent sNR DNA-binding at all DNA elements where variants are confined to the same side of the 3-nt spacer (i.e., the left-hand or right-hand side of the 0-nt variant consensus palindromic DNA element remains fixed) for all 1- to 5-nt variant DNA elements in the genome (Figures 2, 3, S4, and S5).

Moreover, this highly structured symmetrical sNR DNA-binding profile at 0- to 5-nt variant DNA elements in the genome was observed in 157 ER experiments and 194 KR experiments, representing a wide variety of mouse tissues and human cell lines, and across multiple peak selection criteria (L4-L20) (Tables S7 and S8). Of particular importance, there are minimal, if any, peaks in sNR ChIPSeq or ChIPExo experiments that do not contain a 0- to 5-nt variant DNA element, even at the least stringent (L4) peak selection criteria (Tables S7 and S8).

### Quantify the Discrete States of sNR DNA-Binding at 1-nt Variant DNA Elements in the Genome

(S/N) analysis of sNR DNA-binding at 0- to 5-nt variant DNA elements in the genome (displayed by the 252 half-site groups) reveals a highly structured symmetrical DNA-binding profile associated with discrete (S/N) values that decay as the sNR DNA-binding signal decreases from 1- to 5-nt variant DNA elements (Figures 2, 3, S4, and S5). Specifically, (S/N) analysis of sNR DNA-binding at 1-nt variant DNA elements (displayed by the 252 half-site groups) produce a set of symmetrical discrete DNA-binding signals: five plateaus for ER DNA-binding at 1-nt variant ERE DNA elements in the genome (Figure S6) and three plateaus for KR DNA-binding at 1-nt variant HRE DNA elements in the genome (Figure S7). These plateaus characterize the internal inversion symmetry contained within the DNA element, which determines the specific DNA elements that sNRs bind in the genome. These plateaus can be represented as (+2, +1, 0, −1, −2) for ER DNA-binding at 1-nt variant EREs (Figure S6) and (+1, 0, −1) for KR DNA-binding at 1-nt variant HREs in the genome (Figure S7).

ER DNA-binding at 1-nt variant ERE DNA elements in the genome produce five discrete DNA-binding signals across the 252 half-site groups: 6 groups reach the (+2) plateau, 60 groups reach the (+1) plateau, 120 groups reach the (0) plateau, 60 groups reach the (−1) plateau, and 6 groups reach the (−2) plateau (Figure S6). Thus, ER DNA-binding is greatest at the (+2) plateau, which are the six half-site groups that have two reverse-complement vacancies in palindromic position pair 3-8 and 1-10, and two reverse-complement double occupants in palindromic position pair (4-7 and 5-6), (2-9 and 5-6), or (2-9 and 4-7) (Figure S6). ER DNA-binding is least at the (−2) plateau, which are the six half-site groups that have two reverse-complement vacancies in palindromic position pair (4-7 and 5-6), (2-9 and 5-6), or (2-9 and 4-7) and two reverse-complement double occupants in palindromic position pair 3-8 and 1-10 (Figure S6).

In contrast, KR DNA-binding at 1-nt variant HRE DNA elements in the genome produce three discrete DNA-binding signals across the 252 half-site groups: 56 groups reach the (+1) plateau, 140 groups reach the (0) plateau, and 56 groups reach the (−1) plateau (Figure S7) Thus, KR DNA-binding is greatest at the (+1) plateau, which are the 56 half-site groups that have a reverse-complement vacancy in palindromic position pair 2-9 (Figure S7). KR DNA-binding is least at the (−1) plateau, which are the 56 half-site groups that have a reverse-complement double occupant in palindromic position pair 2-9 (Figure S7).

The symbolic representations of the discrete states (+2, +1, 0, −1, −2) for the ERE and (+1, 0, −1) for the HRE can be converted into experimental values (i.e., the (S/N) values) by taking the mean, standard deviation, and difference from the mean of each plateau (Table 3). That is, we replace the symbolic representations with actual (S/N) values determined from ChIPSeq and ChIPExo experiments (Table 3). The exact symmetry is demonstrated by calculating the difference from the mean of each plateau (Table 3). Thus, these calculations quantitatively demonstrate the sNR DNA-binding inversion symmetry of the discrete (S/N) spectrum (Table 3). The five discrete states for the ERE were observed in 157 ER experiments, and the three discrete states for the HRE were observed in 194 KR experiments, representing a wide variety of mouse tissues and human cell lines, and across multiple peak selection criteria (L4-L20) (Tables S9 and S10).

**Table 3:** Quantify the Discrete States of sNR DNA-Binding at 1-nt Variant DNA Elements in the Genome

(A) Signal-To-Noise Ratio (S/N) Analysis of 1-nt Variant ERE DNA Elements in ER (WT-E2-1hr) ChIPSeq Peaks (Figure 2)					
5-State	Algebraic Variable	252 Half-Site Groups	Mean	Standard Deviation	Difference from Mean
+2	+2X	6	32.30	0.23	+8.83
+1	+X	60	27.89	0.42	+4.41
0	0	120	23.47	0.47	0.00
−1	-X	60	19.06	0.42	−4.41
−2	-2X	6	14.64	0.23	−8.83

(B) Signal-To-Noise Ratio (S/N) Analysis of 1-nt Variant HRE DNA Elements in AR (AR-wt1) ChIPSeq Peaks (Figure 3)					
3-State	Algebraic Variable	252 Half-Site Groups	Mean	Standard Deviation	Difference from Mean
+1	+X	56	21.51	1.16	+7.11
0	0	140	14.41	1.19	0.00
−1	-X	56	7.30	1.16	−7.11

(C) Signal-To-Noise Ratio (S/N) Analysis of 1-nt Variant HRE DNA Elements in GR (GR-WT-pred-6am-2) ChIPSeq Peaks (Figure S4)					
3-State	Algebraic Variable	252 Half-Site Groups	Mean	Standard Deviation	Difference from Mean
+1	+X	56	16.30	1.03	+5.00
0	0	140	11.29	1.06	0.00
−1	-X	56	6.29	1.03	−5.00

(D) Signal-To-Noise Ratio (S/N) Analysis of 1-nt Variant HRE DNA Elements in PR (Uterus-PGR-P4) ChIPSeq Peaks (Figure S5)					
3-State	Algebraic Variable	252 Half-Site Groups	Mean	Standard Deviation	Difference from Mean
+1	+X	56	15.38	0.92	+3.46
0	0	140	11.92	0.94	0.00
−1	-X	56	8.46	0.92	−3.46

(S/N) analysis of sNR DNA-binding at 0-nt to 5-nt variant DNA elements in the genome (displayed by the 252 half-site groups) reveals a highly structured symmetrical DNA-binding profile associated with discrete (S/N) values that decay as the sNR DNA-binding signal decreases from 1-nt to 5-nt variant DNA elements (A) ER DNA-binding at 1-nt variant ERE DNA elements in the genome produce five (5) discrete DNA-binding signals across the 252 half-site groups: 6 groups reach the (+2) plateau, 60 groups reach the (+1) plateau, 120 groups reach the (0) plateau, 60 groups reach the (−1) plateau, 6 groups reach the (−2) plateau. (B-D) KR DNA-binding at 1-nt variant HRE DNA elements in the genome produce three (3) discrete DNA-binding signals across the 252 half-site groups: 56 groups reach the (+1) plateau, 140 groups reach the (0) plateau, 56 groups reach the (−1) plateau. The symbolic representations of the discrete states (+2, +1, 0, −1, −2) for the ERE and (+1, 0, −1) for the HRE can be converted into experimental values (i.e., the (S/N) values) by taking the mean, standard deviation, and difference from the mean of each plateau. The exact symmetry is demonstrated by calculating the difference from the mean of each plateau. Thus, these calculations quantitatively demonstrate the sNR DNA-binding inversion symmetry of the discrete (S/N) spectrum.

The symbolic representations of the discrete states (+2, +1, 0, −1, −2) for the ERE and (+1, 0, −1) for the HRE can also be replaced with algebraic variables to formally define the methodology (Table 4; Figures S8 and S9). The 3-state HRE is generated by splitting the nucleotides into a (4,1,0,0,0) grouping (Table 4; Figure S8). That is, palindromic position pair 2-9 (symbolically represented by “A”) versus palindromic position pairs 5-6, 1-10, 4-7, 3-8 (symbolically represented by “B”) (Table 4; Figure S8). Similarly, the 5-state ERE is generated by splitting the nucleotides into a (3,2,0,0,0) grouping (Table 4; Figure S9). That is, palindromic position pair 3-8 and 1-10 (symbolically represented by “A”) versus palindromic position pairs 5-6, 4-7, 2-9 (symbolically represented by “B”) (Table 4; Figure S9). Therefore, if an A is replaced with a B, the impact is (–X) (i.e., the sNR DNA-binding affinity is decreased), whereas if a B is replaced with an A, the impact is exactly the opposite (+X) (i.e., the sNR DNA-binding affinity is increased) to the same magnitude (Table 4; Figures S8 and S9). Thus, this algebraic representation explains why sNR DNA-binding is quantitatively precise and also why there are three discrete DNA-binding states at 1-nt variant HRE DNA elements (by the KRs) and five discrete DNA-binding states at 1-nt variant ERE DNA elements (by the ERs) (Table 4; Figures S8 and S9). The algebraic complexities increase substantially beyond these (4,1,0,0,0) HRE and (3,2,0,0,0) ERE representations. For example, the (3,1,1,0,0) grouping (symbolically represented by “A, B, C”) creates nine discrete states (Table 4, Figure S10). The (2,2,1,0,0) grouping (symbolically represented by “A, B, C”) creates 11 discrete states, the (2,1,1,1,0) grouping (symbolically represented by “A, B, C, D”) creates 25 discrete states, and the (1,1,1,1,1) grouping (symbolically represented by “A, B, C, D, E”) creates 51 discrete states, which are all the remaining possibilities for a 5-nt DNA element in the genome (i.e., a 5-nt DNA element, followed by any arbitrary spacer, followed by its 5-nt reverse-complement DNA element) (Figure S11). Thus, we have provided a mathematical formula that represents the sNR DNA-binding profile observed in 157 ER experiments and 194 KR experiments, representing a wide variety of mouse tissues and human cell lines, and across multiple peak selection criteria (L4-L20) (Tables S9 and S10).

**Table 4:** Algebraic Equations of sNR DNA-Binding at 1-nt Variant DNA Elements in the Genome

DNA Element	Discrete States	Algebraic Variables	Algebraic Equations
HRE	3-State (4,1,0,0,0)	(-X), 0, (+X)	A – B = X B – A = -X
ERE	5-State (3,2,0,0,0)	(-2X), (-X), 0, (+X), (+2X)	A – B = X B – A = -X AA – BB = 2X BB – AA = -2X AB – BB = A – B = X BB – AB = B – A = -X
	9-State (3,1,1,0,0)	(-X), (-Y), (-Z), -(Y + Z), 0, (+X), (+Y), (+Z), (Y + Z)	A – B = X B – C = Y A – C = Z AB – CC = Y + Z AC – BC = A – B = X BC – AC = B – A = -X AC – CC = A – C = Z CC – AC = C – A = -Z BC – CC = B – C = Y CC – BC = C – B = -Y CC – AB = -(Y + Z)

The symbolic representations of the discrete states (+2, +1, 0, −1, −2) for the ERE and (+1, 0, −1) for the HRE can also be replaced with algebraic variables to formally define the methodology. The 3-state HRE is generated by splitting the nucleotides into a (4,1,0,0,0) grouping. That is, palindromic position pair 2-9 (symbolically represented by “A”) versus palindromic position pairs 5-6, 1-10, 4-7, 3-8 (symbolically represented by “B”). The 5-state ERE is generated by splitting the nucleotides into a (3,2,0,0,0) grouping. That is, palindromic position pair 3-8 and 1-10 (symbolically represented by “A”) versus palindromic position pairs 5-6, 4-7, 2-9 (symbolically represented by “B”). Therefore, if an A is replaced with a B, the impact is (–X) (i.e., the sNR DNA-binding affinity is decreased), whereas if a B is replaced with an A, the impact is exactly the opposite (+X) (i.e., the sNR DNA-binding affinity is increased) to the same magnitude. Thus, this algebraic representation explains why sNR DNA-binding is quantitatively precise and also why there are three discrete DNA-binding states at 1-nt variant HRE DNA elements (by the KRs) and five discrete DNA-binding states at 1-nt variant ERE DNA elements (by the ERs). The algebraic complexities increase substantially beyond these (4,1,0,0,0) HRE and (3,2,0,0,0) ERE representations. For example, (3,1,1,0,0) grouping (symbolically represented by “A, B, C”) creates nine discrete states.

### Internal Inversion Symmetry of sNR Binding in the Genome

(S/N) analysis of sNR DNA-binding at each of the 81,922 0- to 5-nt variant DNA elements in the genome (displayed by the 252 half-site groups) clearly demonstrates that sNR binding in the genome follows inversion symmetry (i.e., the number of sNR DNA-binding events at a particular DNA element in the genome is equivalent to the number of sNR DNA-binding events at its reverse-complement DNA element in the genome). The decay of the symmetrical sNR DNA-binding profile from 0- to 5-nt variant DNA elements in the genome confirms that the specific 0- to 5-nt variant DNA elements where sNRs bind in the genome is determined by internal inversion symmetry within the DNA element (i.e., sNR binding in the genome is determined by the position of variants within the DNA element and its reverse-complement position). Because it is difficult to translate the 252 half-site groups to specific DNA elements, the absolute counts of each of the 81,922 0- to 5-nt variant DNA elements in an experiment can also be displayed by the position of the variant within the DNA element, rather than by the 252 half-site groups. That is, the 0- to 5-nt variant DNA elements include the 1 0-nt variant consensus palindromic DNA element, 30 1-nt variant DNA elements (10 variant positions), 405 2-nt variant DNA elements (45 variant positions), 3,240 3-nt variant DNA elements (120 variant positions), 17,010 4-nt variant DNA elements (210 variant positions), and 61,236 5-nt variant DNA elements (252 variant positions), for a total of 81,922 DNA elements (Table 1).

#### Internal Inversion Symmetry of ER DNA-Binding at ERE DNA Elements in the Genome

As previously discussed, ER DNA-binding at 1-nt variant ERE DNA elements in the genome is increased at DNA elements with variants in palindromic position pair 3-8 or 1-10 (Figures 1 and S12). This ER DNA-binding profile at 1-nt variant ERE DNA elements in the genome was observed in 157 ER experiments, representing a wide variety of mouse tissues and human cell lines, and across multiple peak selection criteria (L4-L20) (Table S11).

These 1-nt variant ERE DNA-binding rules (i.e., increased DNA-binding at DNA elements with variants in palindromic position pair 3-8 or 1-10) are also applicable to ER DNA-binding at 2-nt variant ERE DNA elements in the genome (Figure S13). Thus, ER DNA-binding at 2-nt variant ERE DNA elements is increased at DNA elements that have variants in positions 1, 3, 8, or 10 (in any combination with each other) in the genome (i.e., (3-8), (1-10), (1,3)-(8,10), (1,8)-(3,10)) (Figure S13). In addition, ER DNA-binding is also increased at DNA elements that have variants in palindromic position pair (2,3)-(8,9) (i.e., two of the four positions that distinguish the 0-nt variant consensus palindromic ERE DNA element from the 0-nt variant consensus palindromic HRE DNA element) (Figure S13). However, the increased DNA-binding of ER at these DNA elements is reduced (i.e., suppressed) at DNA elements where the variants “crossover” the 3-nt spacer in the genome (i.e., have variants on both sides of the 3-nt spacer) (i.e., (2,8)-(3,9)) (Figure S13). Furthermore, ER DNA-binding is increased at DNA elements with variants in palindromic position pair (3,5)-(6,8), as well as at DNA elements with variants in palindromic position pair (4,5)-(6,7) (Figure S13). Likewise, the increased DNA-binding of ER at these DNA elements is suppressed at DNA elements where the variants crossover the 3-nt spacer (i.e., (3,6)-(5,8), (4,6)-(5,7)) (Figure S13). Thus, the ER DNA-binding rules established at 1-nt variant ERE DNA elements in the genome (i.e., increased DNA-binding at DNA elements with variants in palindromic position pair 3-8 or 1-10) are also applicable to ER DNA-binding at 2-nt variant ERE DNA elements in the genome (i.e., increased DNA-binding at DNA elements with variants in positions 1, 3, 8, or 10 in any combination with each other). That is, the first set of DNA-binding rules (defined by ER DNA-binding at 1-nt variant ERE DNA elements in the genome) is based on which palindromic position pair the variant occupies (i.e., ER DNA-binding is increased at DNA elements with variants in palindromic position pair 3-8 or 1-10). Furthermore, unlike 1-nt variant DNA elements, 2-nt variant DNA elements allow variants to crossover the 3-nt spacer, thus revealing a new DNA-binding pattern not applicable to 1-nt variant DNA elements. That is, the second set of DNA-binding rules (functionally subordinate to and independent of the DNA-binding rules defined by ER DNA-binding at 1-nt variant ERE DNA elements) defined by ER DNA-binding at 2-nt variant ERE DNA elements in the genome, is based on how the position of the variants relate to each other (i.e., ER DNA-binding is increased at DNA elements with variants that do not crossover the 3-nt spacer). To summarize, the second set of ER DNA-binding rules (functionally subordinate to and independent of the DNA-binding rules defined by ER DNA-binding at 1-nt variant DNA elements) defined by ER DNA-binding at 2-nt variant ERE DNA elements in the genome include increased ER DNA-binding at DNA elements with variants in palindromic position pair (2,3)-(8,9), (3,5)-(6,8), and (4,5)-(6,7); this increased DNA-binding of ER is suppressed at DNA elements where these variants crossover the 3-nt spacer (Figure S13). This ER DNA-binding profile at 2-nt variant ERE DNA elements in the genome was observed in 157 ER experiments, representing a wide variety of mouse tissues and human cell lines, and across multiple peak selection criteria (L4-L20) (Table S12).

Analysis of ER DNA-binding at 3-nt variant ERE DNA elements in the genome demonstrates the same properties that were observed in ER DNA-binding at 1-nt variant ERE DNA elements and 2-nt variant ERE DNA elements in the genome (Figure S14). First, ER DNA-binding at 3-nt variant ERE DNA elements is increased at DNA elements with variants in palindromic position pair (1,3)-(8,10), and the third variant is on the same side of the 3-nt spacer (i.e., (1,3,5)-(6,8,10), (1,3,4)-(7,8,10), (1,2,3)-(8,9,10)) (Figure S14). Thus, confirming that the second set of DNA-binding rules, based on how the position of the variants relate to each other (i.e., ER DNA-binding is increased at DNA elements with variants that do not crossover the 3-nt spacer), is exhibited in ER DNA-binding at 3-nt variant ERE DNA elements in the genome. Second, ER DNA-binding is increased at DNA elements with variants in palindromic position pair 3-8 and any other variant (i.e., (1,3,8)-(3,8,10), (3,5,8)-(3,6,8), (3,4,8)-(3,7,8), (2,3,8)-(3,8,9)) (Figure S14). Thus, confirming that the first set of DNA-binding rules, based on which palindromic position pair the variant occupies (i.e., ER DNA-binding is increased at DNA elements with variants in palindromic position pair 3-8 or 1-10), is exhibited in ER DNA-binding at 3-nt variant ERE DNA elements in the genome. However, this increased DNA-binding of ER was less apparent at DNA elements with variants in palindromic position pair 1-10 (i.e., (1,5,10)-(1,6,10), (1,3,10)-(1,8,10), (1,4,10)-(1,7,10), (1,2,10)-(1,9,10)) (Figure S14). Thus, ER prefers to bind 3-nt variant ERE DNA elements with variants in palindromic position pair (1,3)-(8,10) and the third variant being on the same side of the 3-nt spacer, rather than DNA elements with variants in palindromic position pair 1-10 (Figure S14). Furthermore, other than the increased binding of ER at DNA elements with variants in palindromic position pair 3-8, ER DNA-binding was suppressed if the third variant crossed over the 3-nt spacer for all remaining 1, 3, 8, or 10 variant combinations (i.e., (3,4,5)-(6,7,8), (2,3,5)-(6,8,9), (2,3,4)-(7,8,9), (1,4,5)-(6,7,10), (1,2,5)-(6,9,10), (1,2,4)-(7,9,10)) (Figure S14). This demonstrates that the ER DNA-binding rules established at 1-nt variant ERE DNA elements in the genome (i.e., increased DNA-binding at DNA elements with variants in palindromic position pair 3-8 and 1-10), and the distinct and functionally subordinate ER DNA-binding rules established at 2-nt variant ERE DNA elements in the genome (i.e., increased DNA-binding at DNA elements with variants on the same side of the 3-nt spacer) are co-existing and being maintained in ER DNA-binding at 3-nt variant ERE DNA elements in genome (Figure S14). This ER DNA-binding profile at 3-nt variant ERE DNA elements in the genome was observed in 157 ER experiments, representing a wide variety of mouse tissues and human cell lines, and across multiple peak selection criteria (L4-L20) (Table S13).

These ER DNA-binding rules are maintained at 4-nt variant ERE DNA elements (Figure S15, Table S14) and 5-nt variant ERE DNE elements in the genome (Figure S16, Table S15). Thus, ER DNA-binding at 1- to 5-nt variant ERE DNA elements in the genome is determined by two distinct set of rules: (1) a first set established by ER DNA-binding at 1-nt variant ERE DNA elements, which favor binding at DNA elements with variants in palindromic position pair 3-8 and 1-10, and (2) a distinct and functionally subordinate second set, not applicable to 1- variant DNA elements, which favor binding at DNA elements with variants on the same side of the 3-nt spacer. These ER DNA-binding rules are applicable to ER DNA-binding from 1- to 5-nt variant ERE DNA elements in the genome (Figures 2 and S12–S16). Thus, the specific 1- to 5-nt variant ERE DNA elements where ER binds in the genome is determined by internal inversion symmetry within the DNA element (i.e., ER binding in the genome is determined by the position of variants within the DNA element and its reverse-complement position). Increased ER DNA-binding at specific 1- to 5-nt variant ERE DNA elements in the genome is determined by the reverse-complement vacancy in the palindromic position pair by the following hierarchy: 3-8 > 1-10 > 5-6 > 4-7 > 2-9 (Table S5).

#### Internal Inversion Symmetry of KR DNA-Binding at HRE DNA Elements in the Genome

As previously discussed, KR DNA-binding at 1-nt variant HRE DNA elements in the genome is increased at DNA elements with variants in palindromic position pair 2-9 (Figures 1B and S17). This KR DNA-binding profile at 1-nt variant HRE DNA elements in the genome was observed in 194 KR experiments, representing a wide variety of mouse tissues and human cell lines, and across multiple peak selection criteria (L4-L20) (Table S16).

These 1-nt variant HRE DNA-binding rules (i.e., increased DNA-binding at DNA elements with variants in palindromic position pair 2-9) are also applicable to KR DNA-binding at 2-nt variant HRE DNA elements in the genome (Figure S18). Thus, KR DNA-binding at 2-nt variant HRE DNA elements is increased at DNA elements that have variants in palindromic position pair 2-9, followed by 2-5, 1-2, and 2-3 (and their reverse-complements 6-9, 9-10, 8-9) in the genome (Figure S18). However, the increased DNA-binding of KR at these DNA elements is reduced (i.e., suppressed) at DNA elements where the variants crossover the 3-nt spacer in the genome (i.e., 2-6, 1-9, 2-8, and their reverse-complements 5-9, 2-10, 3-9) (Figure S18). Similar to ER, KR DNA-binding is also increased at DNA elements with variants in palindromic position pair (4,5)-(6,7), potentially revealing a pre-evolved sNR DNA-binding mechanism (Figure S18). Thus, the KR DNA-binding rules established at 1-nt variant HRE DNA elements in the genome (i.e., increased DNA-binding at DNA elements with variants in position 2 or 9) are also applicable to KR DNA-binding at 2-nt variant HRE DNA elements in the genome (i.e., increased DNA-binding at DNA elements with variants in palindromic position pair 2-9). That is, the first set of DNA-binding rules (defined by KR DNA-binding at 1-nt variant HRE DNA elements in the genome) is based on which palindromic position pair the variant occupies (i.e., KR DNA-binding is increased at DNA elements with variants in palindromic position pair 2-9). Furthermore, unlike 1-nt variant DNA elements, 2-nt variant DNA elements allow variants to crossover the 3-nt spacer, thus revealing a new DNA-binding pattern not applicable to 1-nt variant DNA elements. That is, the second set of DNA-binding rules (functionally subordinate to and independent of the DNA-binding rules defined by KR DNA-binding at 1-nt variant HRE DNA elements) defined by KR DNA-binding at 2-nt variant HRE DNA elements in the genome, is based on how the position of the variants relate to each other (i.e., KR DNA-binding is increased at DNA elements with variants that do not crossover the 3-nt spacer). To summarize, the second set of KR DNA-binding rules (functionally subordinate to and independent of the DNA-binding rules defined by KR DNA-binding at 1-nt variant HRE DNA elements) defined by KR DNA-binding at 2-nt variant HRE DNA elements in the genome include increased KR DNA-binding at DNA elements with variants in position 2 or 9 and the second variant is on the same side of the 3-nt spacer, and if variants are in palindromic position pair (4,5)-(6,7); this increased DNA-binding of KR is suppressed at DNA elements where these variants crossover the 3-nt spacer (Figure S18). This KR DNA-binding profile at 2-nt variant HRE DNA elements in the genome was observed in 194 KR experiments, representing a wide variety of mouse tissues and human cell lines, and across multiple peak selection criteria (L4-L20) (Table S17).

Analysis of KR DNA-binding at 3-nt variant HRE DNA elements in the genome demonstrates the same properties that were observed in KR DNA-binding at 1-nt variant HRE DNA elements and 2-nt variant HRE DNA elements in the genome (Figure S19). First, KR DNA-binding at 3-nt variant HRE DNA elements is increased at DNA elements with variants in position 2 or 9, and the remaining two variants are on the same side of the 3-nt spacer (i.e., (1,2,5)-(6,9,10), (2,4,5)-(6,7,9), (2,3,5)-(6,8,9), (1,2,3)-(8,9,10)) (Figure S19). Thus, confirming that the second set of DNA-binding rules, based on how the position of the variants relate to each other (i.e., KR DNA-binding is increased at DNA elements with variants that do not crossover the 3-nt spacer), is exhibited in KR DNA-binding at 3-nt variant HRE DNA elements in the genome. Second, KR DNA-binding is increased at DNA elements with variants in palindromic position pair 2-9 with any other variant (i.e., (1,2,9)-(2,9,10), (2,5,9)-(2,6,9), (2,4,9)-(2,7,9), (2,3,9)-(2,8,9)) (Figure S19). Thus, confirming that the first set of DNA-binding rules, based on which palindromic position pair the variant occupies (i.e., KR DNA-binding is increased at DNA elements with variants in palindromic position pair 2-9), is exhibited in KR DNA-binding at 3-nt variant HRE DNA elements in the genome. This demonstrates that the KR DNA-binding rules established at 1-nt variant HRE DNA elements in the genome (i.e., increased DNA-binding at DNA elements with variants in palindromic position pair 2-9) and the distinct and functionally subordinate KR DNA-binding rules established at 2-nt variant HRE DNA elements in the genome (i.e., increased DNA-binding at DNA elements with variants on the same side of the 3-nt spacer) are co-existing and being maintained in KR DNA-binding at 3-nt variant HRE DNA elements in genome (Figure S19). This KR DNA-binding profile at 3-nt variant HRE DNA elements in the genome was observed in 194 KR experiments, representing a wide variety of mouse tissues and human cell lines, and across multiple peak selection criteria (L4-L20) (Table S18).

These KR DNA-binding rules are maintained at 4-nt variant HRE DNA elements (Figure S20, Table S19) and 5-nt variant HRE DNA elements in the genome (Figure S21, Table S20). Thus, KR DNA-binding at 1- to 5-nt variant HRE elements in the genome is determined by two distinct set of rules: (1) a first set established by KR DNA-binding at 1-nt variant HRE DNA elements, which favor binding at DNA elements with variants in palindromic position pair 2-9 and (2) a distinct and functionally subordinate second set, not applicable to 1-nt variant DNA elements, which favor binding at DNA elements with variants on the same side of the 3-nt spacer. These KR DNA-binding rules are applicable to KR DNA-binding from 1-nt to 5-nt variant HRE DNA elements in the genome and for all members of the KR family (Figures 3, S4, S5, and S17–S21). Thus, the specific 1- to 5-nt variant HRE DNA elements where KR binds in the genome is determined by internal inversion symmetry within the DNA element (i.e., KR binding in the genome is determined by the position of variants within the DNA element and its reverse-complement position). Increased KR DNA-binding at specific 1- to 5-nt variant HRE DNA elements in the genome is determined by the reverse-complement vacancy in the palindromic position pair by the following hierarchy: 2-9 > 5-6 > 1-10 > 4-7 > 3-8 (Table S6).

### Distinguishing NRFE (Functional) from Non-NRFE (Non-Functional) DNA Elements in the Genome

In a previously published study, we determined that only a small fraction of all sNR chromatin-interacting events observed in ChIPSeq and ChIPExo experiments is associated with transcriptional activity, and this functionality is restricted to DNA elements that vary from the consensus palindromic DNA element by one or two nts (i.e., NRFEs) (Coons et al., 2017). This finding was observed in a wide variety of models including DBD mutant models, cancerous tumors that were sensitive to hormone deprivation versus tumors that had acquired resistance, constitutively active models, SUMOylation mutant models, etc. (Coons et al., 2017). This functionality was further confirmed via hormone-mediated recruitment of a variety of genomic features that have been correlated with enhancer activation, including enhancer RNA (eRNA) transcription, RNAPII occupancy, and coregulator and TF recruitment (Coons et al., 2017). This study included analysis of over 1300 experiments, representing a wide variety of mouse tissues and human cell lines, and across multiple peak selection criteria (L4-L20) (Coons et al., 2017).

**Figure 4:**
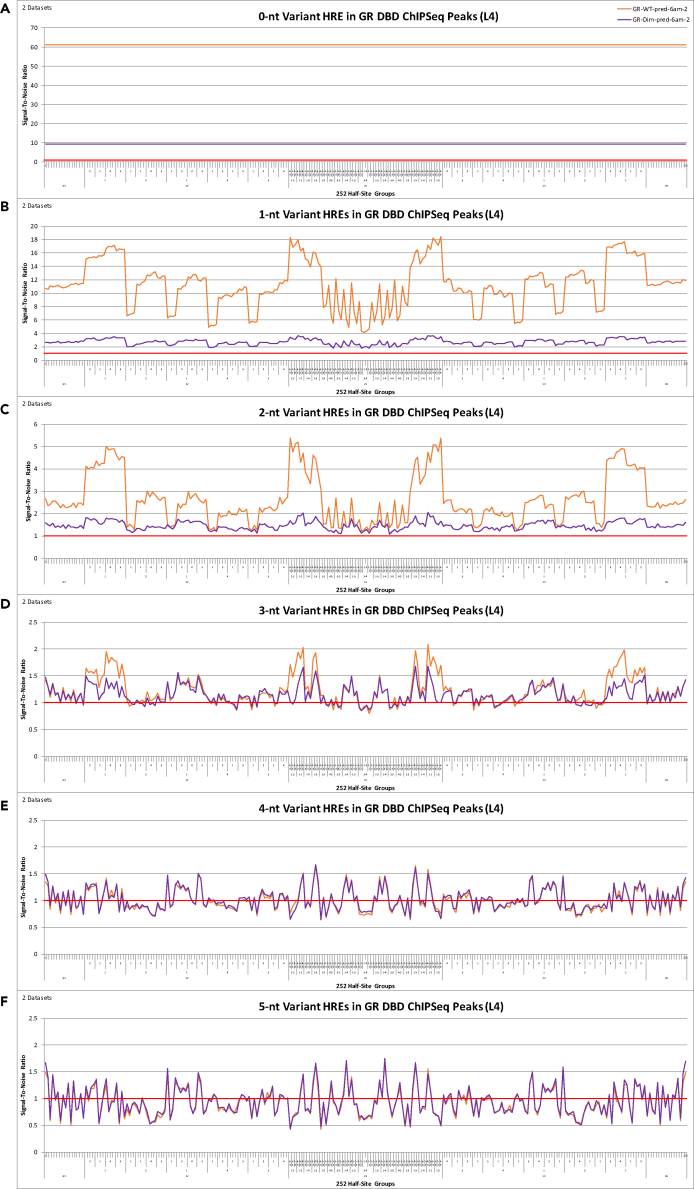
(S/N) Analysis of 0- to 5-nt Variant HRE DNA Elements in GR and GR-Dim ChIPSeq Peaks (A–F) (S/N) analysis of 0- to 5-nt variant HRE DNA elements (displayed by the 252 half-site groups) in GR (GR-WT-pred-6am-2) (23,742 peaks, 142-nt peak length) ChIPSeq peaks and GR-Dim (GR-Dim-pred-6am-2) (34,966 peaks, 148-nt peak length) ChIPSeq peaks. Upper and lower one-tailed Poisson significance thresholds at p < 0.001: (A) 0.00, 3.11 [GR-WT] 0.00, 2.63 [GR-Dim]; (B) 0.58, 1.47 [GR-WT] 0.66, 1.38 [GR-Dim]; (C) 0.82, 1.19 [GR-WT] 0.86, 1.15 [GR-Dim]; (D) 0.90, 1.11 [GR-WT] 0.92, 1.09 [GR-Dim]; (E) 0.92, 1.09 [GR-WT] 0.93, 1.07 [GR-Dim]; and (F) 0.89, 1.11 [GR-WT] 0.91, 1.09 [GR-Dim]. X-axis order = reverse-complement vacancy position ID 2-9 > 5-6 > 1-10 > 4-7 > 3-8. See Table S6 for x-axis details.

One of the models used in our previous study to dissect the relative biological roles of *cis*-regulatory DBD-dependent transcriptional regulation was GR-Dim, a GR DBD mutant mouse model (A458T) (Coons et al., 2017). Tissues derived from GR-Dim are refractory to endogenous glucocorticoids (i.e., no hormone-mediated transcriptional response, neither induction nor repression) (Kleiman et al., 2012, Frijters et al., 2010, Lim et al., 2015, Berg, 1998, Schiller et al., 2014). Using GR-Dim as a specific example from the numerous models analyzed in the previous study, analysis of GR-Dim DNA-binding at 0- to 5-nt variant HRE DNA elements in the genome demonstrates a dramatic reduction in DNA-binding at 0- to 2-nt variant HRE DNA elements in the genome compared to wtGR, consistent with the conclusions observed in our previous study using a different analysis methodology (Coons et al., 2017) (Figures 4 and S22–S24). Other than a slight decrease in DNA-binding at a few specific 3-nt variant HRE DNA elements, the DNA-binding profiles were identical between GR-Dim and wtGR at 3- to 5-nt variant HRE DNA elements in the genome, including at “palindrome half-sites” (i.e., half-site group #1 and half-site group #252 on the x-axis) (Figures 4 and S22–S24). This confirms that GR DNA-binding at “palindrome half-sites” is not functionally active (Figures 4 and S22–S24). The two endpoints (half-site group #1 and half-side group #252 on the x-axis) represent GR DNA-binding at all DNA elements where variants are confined to the same side of the 3-nt spacer (i.e., the left-hand or right-hand side of the 0-nt variant consensus palindromic DNA element remains fixed) for all 1- to 5-nt variant DNA elements in the genome (Figures 4 and S22–S24). Furthermore, although GR-Dim has a significantly reduced DNA-binding signal at NRFE HRE DNA elements, the DNA-binding profiles of GR-Dim and wtGR are highly structured and converge onto the same ((S/N) value = 1) noise profile (Figures 4 and S22–S24). This would not occur if this DNA-binding was random (i.e., two random signals do not map on top of each other), and thus these identical DNA-binding profiles demonstrate inherent structure in DNA-binding through 5-nt variant DNA elements in the genome (Figures 4 and S22–S24). Note that the slight decrease in DNA-binding at a few specific 3-nt variant HRE DNA elements by GR-Dim is made apparent by the DNA-binding enhancement of a particular experiment at 0- to 2-nt variant HRE DNA elements as the relative (S/N) values of sNR DNA-binding signals are scale invariant (i.e., the relative ratios between the 0- to 5-nt variant groups are constant) (Figures S2 and S3).

**Figure 5:**
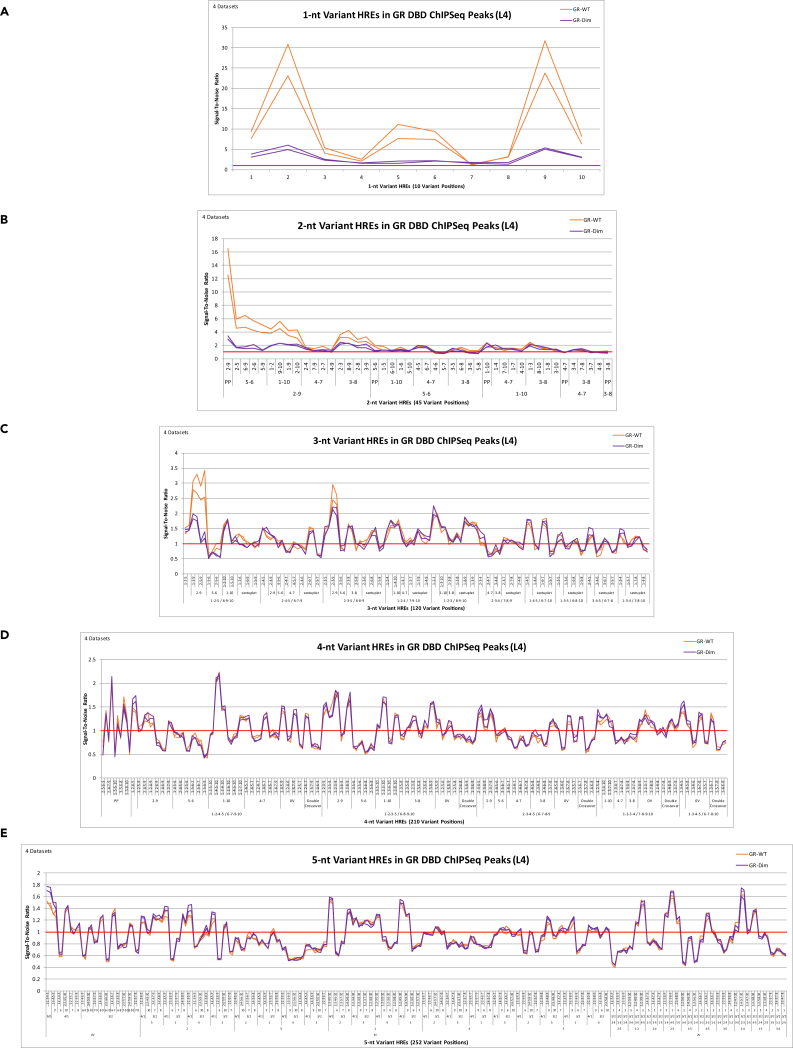
(S/N) Analysis of 1- to 5-nt Variant HRE DNA Elements in GR and GR-Dim ChIPSeq Peaks (A–E) (S/N) analysis of 1- to 5-nt variant HRE DNA elements (displayed by variant position) in GR (GR-WT-pred-6am-1) (34,758 peaks, 151-nt peak length) (GR-WT-pred-6am-2) (23,742 peaks, 142-nt peak length) ChIP-Seq peaks and GR-Dim (GR-Dim-pred-6am-1) (22,130 peaks, 163-nt peak length) (GR-Dim-pred-6am-2) (34,966 peaks, 148-nt peak length) ChIPSeq peaks. Upper and lower one-tailed Poisson significance thresholds at p < 0.001: (A) 0.00, 2.60; 0.00, 3.11 [GR-WT] 0.00, 2.91; 0.00, 2.63 [GR-Dim]; (B) 0.58, 1.49; 0.48, 1.62 [GR-WT] 0.48, 1.61; 0.56, 1.49 [GR-Dim]; (C) 0.75, 1.27; 0.69, 1.35 [GR-WT] 0.70, 1.34; 0.74, 1.28 [GR-Dim]; (D) 0.85, 1.16; 0.81, 1.20 [GR-WT] 0.82, 1.19; 0.85, 1.16 [GR-Dim]; (E) 0.91, 1.09; 0.89, 1.11 [GR-WT] 0.89, 1.11; 0.91, 1.09 [GR-Dim].

Of particular importance, the DNA-binding curve of GR-Dim has a nearly identical structure as wtGR at 0- to 5-nt variant HRE DNA elements in the genome, with significantly reduced amplitude compared with wtGR at 0- to 2-nt variant HRE DNA elements in the genome (Figures 4 and S22–S24). This demonstrates that the effect of the DBD mutation is a loss in GR residence time at the DNA element, rather than the inability to follow the same DNA-binding rules as wtGR (Figures 4 and S22–S24). A loss in residence time of GR-Dim on DNA compared with wtGR has also been identified via reflected light-sheet microscopy (Gebhardt et al., 2013). When we display this same data by the position of the variants, rather than by the 252 half-site groups, the impact of the DBD mutation is most pronounced at DNA elements where wtGR DNA-binding is most increased (Figures 5 and S25). For example, the DNA-binding signal of GR-Dim at 1-nt variant HRE DNA elements is most reduced at DNA elements that vary position 2 (or its reverse-complement position 9), position 5 (or its reverse-complement position 6), and position 1 (or its reverse-complement position 10), the DNA elements where wtGR DNA-binding is most increased (Figures 5 and S25). Likewise, GR-Dim's decreased DNA-binding signal at 2-nt variant HRE DNA elements in the genome occurs specifically at DNA elements where wtGR DNA-binding is most increased (i.e., at DNA elements with variants in palindromic position pair 2-9 followed by 2-5, 1-2 and 2-3 [and their reverse-complements 6-9, 9-10, 8-9]) (Figures 5 and S25). Similarly, GR-Dim's slight decrease in DNA-binding at 3-nt variant HRE DNA elements occurs specifically at DNA elements where wtGR DNA-binding is most increased: at DNA elements with variants in palindromic position pair 2-9 and positions 5, 1, or 3 (i.e., (2,5,9)-(2,6,9), (1,2,9)-(2,9,10), (2,3,9)-(2,8,9)) (Figures 5 and S25). Thus, these are the DNA elements that define functionally active binding sites in chromatin, consistent with the conclusions observed in our previous study using a different analysis methodology (Coons et al., 2017). Collectively, this suggests that the retention time of sNR DNA-binding at 3- to 5-nt variant DNA elements (non-NRFEs), observed in both the wtGR and GR-Dim, is not long enough to initiate and/or maintain transcription, and hence, is non-functional.

To summarize, the DNA-binding of sNRs in the genome is determined by (1) a set of rules defined by sNR DNA-binding at 1-nt variant DNA elements based on which palindromic position pair the variant occupies (i.e., ER DNA-binding is increased at DNA elements with variants in palindromic position pair 3-8 or 1-10 of the ERE, whereas KR DNA-binding is increased at DNA elements with variants in palindromic position pair 2-9 of the HRE) and (2) a second distinct set of rules (functionally subordinate to and independent of the DNA-binding rules defined by sNR DNA-binding at 1-nt variant DNA elements) defined by sNR DNA-binding at 2-nt variant DNA elements based on how the position of the variants relate to each other (i.e., sNR DNA-binding is increased at DNA elements with variants that do not crossover the 3-nt spacer). These DNA-binding rules, dictated by inversion symmetry, continue through 5-nt variant DNA elements in the genome, even though the sNR DNA-binding signal decays as the number of variants increases from 0- to 5-nt variant DNA elements. Furthermore, the sNR DBD mutant mouse model, GR-Dim, confirms that although DNA-binding at NRFEs (i.e., the 0-nt variant consensus palindromic DNA element, 1-nt variant DNA elements, and 2-nt variant DNA elements) are the only functional DNA-binding sites, DNA-binding at non-NRFEs are subject to the same DNA-binding rules, dictated by inversion symmetry, governing NRFE DNA-binding sites in the genome. Thus, the sNR DNA-binding rules extend (i.e., are applicable) to DNA elements that do not support transcriptional activity. That is, the DNA-binding rules are obeyed, but the retention time of the sNR at these non-functional DNA elements is not long enough to initiate and/or maintain transcription. Therefore, functionality is determined at the individual nucleotide level, and the residence time (or strength of binding) is dictated by the number and position of variants within the DNA element (i.e., inversion symmetry and DNA sequence constraints).

Furthermore, the fact that ChIPSeq and ChIPExo analysis of sNR binding in the genome follows inversion symmetry at 0- to 5-nt variant DNA elements for six different sNRs, in hundreds of experiments, and at the least stringent peak selection criteria (L4) demonstrates a previously underappreciated level of accuracy of these technologies. Much of the criticism of these technologies relates to the practice of comparing individual peaks between two experiments (which are highly variable from sample to sample) and the use of position weight matrices (PWMs) for DNA motif identification (which does not detect this level of detail in the sequence analysis) (Coons et al., 2017).

### Inversion Symmetry Detection Methodology

Heuristic-based search engines and expert systems are limited to algorithms that are empirically derived from the currently available information database. For example, scoring DNA sequences against PWMs is a widely adopted method to identify TF *cis*-regulatory DNA elements in ChIPSeq or ChIPExo experiments. PWMs are used to scan a DNA sequence for the presence of DNA sequences that are significantly more similar to the PWMs than to the background (Aerts, 2012). DNA motif identification using PWMs has suggested that ER interacts with many “ERE-like” motifs, including PPAR, TR, and VDR motifs in the genome (Hewitt et al., 2012). These observations have led many to conclude that ER can indiscriminately bind to the sequence-specific *cis*-regulatory DNA element of other nuclear receptors, thus promoting the misconception that there is considerable promiscuity in DNA-binding between members of the NR superfamily. Furthermore, different *cis*-regulatory DNA elements have been delineated for the different members of the KR family, a conclusion not observed in our analysis of KR DNA-binding at 81,922 0- to 5-nt variant HRE DNA elements in the genome from 194 KR experiments (representing a wide variety of mouse tissues and human cell lines, and across multiple peak selection criteria (L4-L20)) (Geserick et al., 2005). To assess the resolution capabilities of PWM-based DNA motif identification, we grouped the ChIPSeq or ChIPExo peaks from each experiment by the number of variants each ERE or HRE DNA element within the peak contained (i.e., peaks that contain a 0-nt variant consensus palindromic DNA element, 1-nt variant DNA element, 2-nt variant DNA element, 3-nt variant DNA element, 4-nt variant DNA element, or 5-nt variant DNA element) and analyzed these grouped peaks using PWMs.

Of 157 ER experiments, on average, PWMs identified the ERE motif (VAGGTCACNSTGACC) in 99%–99% (L4-L20) of the peaks that contain a 0-nt variant consensus palindromic ERE DNA element, 95%–97% (L4-L20) of the peaks that contain a 1-nt variant ERE DNA element, 50%–58% (L4-L20) of the peaks that contain a 2-nt variant ERE DNA element, 26%–38% (L4-L20) of the peaks that contain a 3-nt variant ERE DNA element, 27%–41% (L4-L20) of the peaks that contain a 4-nt variant ERE DNA element, and 26%–41% (L4-L20) of the peaks that contain a 5-nt variant ERE DNA element (Figure S26). Because peaks can contain multiple ERE DNA elements, we then assigned the peaks to the ERE DNA element with the least number of variants relative to the 0-nt consensus palindromic ERE DNA element, thus defining each ChIPSeq or ChIPExo peak by a single ERE DNA element (i.e., unique). This secondary analysis allowed us to determine whether the ERE motif identified (i.e., selected by PWMs in ∼33% of the peaks that contain a 3- to 5-nt variant ERE DNA element) was due to a 0- to 2-nt variant ERE DNA element also occurring in those peaks. Now, PWMs identified the ERE motif in 99%–99% (L4-L20) of the peaks that contain a 0-nt variant consensus palindromic ERE DNA element, 95%–97% (L4-L20) of the peaks that contain a 1-nt variant ERE DNA element, 46%–52% (L4-L20) of the peaks that contain a 2-nt variant ERE DNA element, 3%–4% (L4-L20) of the peaks that contain a 3-nt variant ERE DNA element, 1%–1% (L4-L20) of the peaks that contain a 4-nt variant ERE DNA element, and 1%–1% (L4-L20) of the peaks that contain a 5-nt variant ERE DNA element (Figure S26). Thus, PWMs identified the ERE motif in the majority of peaks that contain a 0- or a 1-nt variant ERE DNA element, about half of the peaks that contain a 2-nt variant ERE DNA element, and did not identify the ERE motif in peaks that contain a 3-nt, 4-nt, or 5-nt variant ERE DNA element (Figure S26). Therefore, PWM analyses would lead one to conclude that the majority of ER DNA-binding events in the genome are being driven by mechanisms other than the ERE sequence, because peaks that contain a 0-nt variant consensus palindromic ERE DNA element or 1-nt variant ERE DNA element constitute, on average, 16%–27% (L4-L20) of all ER DNA-binding events in an experiment, a proportion observed in 157 ER experiments, representing a wide variety of mouse tissues and human cell lines, and across multiple peak selection criteria (L4-L20) (Coons et al., 2017).

Alternatively, of 194 KR experiments, there was a wide distribution in the number of peaks identified by PWMs as containing the ARE motif (RGRACASNSTGTYCYB), GRE motif (NRGVACABNVTGTYCY), GRE 2 motif (VAGRACAKWCTGTYC), PRE motif (VAGRACAKNCTGTBC), or ARE palindrome half-site motif (CCAGGAACAG) (Table 5, Figures S27–S32). For example, of the peaks that contain a 1-nt variant HRE DNA element, on average, 64%–72% (L4-L20) of the peaks were identified by PWMs as containing the GRE motif, whereas 99%–99% (L4-L20) of those exact same peaks were identified by PWMs as containing the PRE motif (Table 5, Figure S27–S32). Or, of the peaks that contain a 2-nt variant HRE DNA element, 31%–39% (L4-L20) of the peaks were identified by PWMs as containing the GRE motif, whereas 86%–90% (L4-L20) of those exact same peaks were identified by PWMs as containing the PRE motif (Table 5, Figure S27–S32). Thus, it is apparent why the use of PWMs for DNA motif identification has led investigators to delineate different *cis*-regulatory DNA elements for the different KRs. However, analysis of KR DNA-binding at 81,922 0- to 5-nt variant HRE DNA elements in the genome quantitatively demonstrates that all members of the KR family follow the same DNA-binding rules and thus bind the same DNA elements (Tables S8 and S16–S20). These DNA-binding rules at 0- to 5-nt variant HRE DNA elements in the genome was observed in 194 KR ChIPSeq experiments, representing a wide variety of mouse tissues and human cell lines and across multiple peak selection criteria (L4-L20) (Tables S8 and S16–S20).

**Table 5:** Inversion Symmetry Detection Methodology

The Number of DNA Motifs Detected by Position Weight Matrices					
All Peaks That Contain 0- to 5-nt Variant HRE DNA Elements					
	DNA Motifs Detected by Position Weight Matrices				
A	B	C	D	E	F
DNA Element	ARE	GRE	GRE 2	PRE	ARE Palindrome Half-Site
	RGRACASNSTGTYCYB	NRGVACABNVTGTYCY	VAGRACAKWCTGTYC	VAGRACAKNCTGTBC	CCAGGAACAG
0-nt Variant HRE	96%–96%	96%–97%	99%–100%	99%–100%	88%–89%
1-nt Variant HRE	76%–81%	64%–72%	84%–88%	99%–99%	77%–80%
2-nt Variant HRE	41%–50%	31%–39%	43%–53%	86%–90%	69%–73%
3-nt Variant HRE	19%–29%	14%–24%	19%–31%	59%–69%	60%–64%
4-nt Variant HRE	18%–29%	14%–24%	19%–31%	50%–64%	57%–63%
5-nt Variant HRE	18%–30%	14%–24%	19%–31%	50%–63%	56%–63%

Unique Peaks That Contain 0- to 5-nt Variant HRE DNA Elements					
	DNA Motifs Detected by Position Weight Matrices				
A	B	C	D	E	F
DNA Element	ARE	GRE	GRE 2	PRE	ARE Palindrome Half-Site
	RGRACASNSTGTYCYB	NRGVACABNVTGTYCY	VAGRACAKWCTGTYC	VAGRACAKNCTGTBC	CCAGGAACAG
0-nt Variant HRE	96%–96%	96%–97%	99%–100%	99%–100%	88%–89%
1-nt Variant HRE	76%–81%	64%–71%	84%–88%	99%–99%	77%–80%
2-nt Variant HRE	39%–47%	29%–37%	42%–51%	85%–89%	68%–72%
3-nt Variant HRE	9%–14%	6%–10%	8%–13%	49%–57%	56%–59%
4-nt Variant HRE	1%–2%	1%–2%	1%–2%	19%–25%	46%–50%
5-nt Variant HRE	1%–2%	1%–1%	1%–1%	6%–8%	33%–39%

Scoring DNA sequences against position weight matrices (PWMs) is a widely adopted method to identify TF *cis*-regulatory DNA elements in ChIPSeq or ChIPExo experiments. To assess the resolution capabilities of PWM-based DNA motif identification, we grouped the peaks from each KR experiment (194 experiments) by the number of variants each HRE DNA element within the peak contained (i.e., peaks that contain a 0-nt variant consensus palindromic DNA element, 1-nt variant DNA element, 2-nt variant DNA element, 3-nt variant DNA element, 4-nt variant DNA element, or 5-nt variant DNA element) and analyzed these grouped peaks using PWMs for detection of the (Column B) ARE motif (RGRACASNSTGTYCYB), (Column C) GRE motif (NRGVACABNVTGTYCY), (Column D) GRE 2 motif (VAGRACAKWCTGTYC), (Column E) PRE motif (VAGRACAKNCTGTBC), and (Column F) ARE palindrome half-site motif (CCAGGAACAG). The data are displayed as the number of peaks identified by PWMs as containing each DNA motif, out of the total number of peaks that contain a 0- to 5-nt variant HRE DNA element. For example, of the peaks that contain a 1-nt variant HRE DNA element, on average, 64%–72% (L4-L20) of the peaks were identified by PWMs as containing the GRE motif, whereas 99%–99% (L4-L20) of those exact same peaks were identified as containing the PRE motif. Because peaks can contain multiple HRE DNA elements, we then assigned the peaks to the HRE DNA element with the least number of variants relative to the 0-nt consensus palindromic HRE DNA element, thus defining each ChIPSeq or ChIPExo peak by a single HRE DNA element (i.e., unique). This secondary analysis allowed us to determine whether the DNA motif identified (i.e., selected by PWMs in peaks that contain a 3- to 5-nt variant HRE DNA element) was due to a 0- to 2-nt variant HRE DNA element also occurring in those peaks.

It is important to recognize the caveats associated with current PWM-based DNA motif identification algorithms, and careful consideration(s) should be given with regard to any conclusions drawn from their output. PWMs are adequate preliminary analysis tools to use when you have a TF with an unknown *cis*-regulatory DNA element, but once this information is identified it is not a useful tool for discriminating between similar DNA elements. Our analysis of the resolution capabilities of PWM-based DNA motif identification explains why the majority of TF DNA-binding events in the genome have previously “appeared” to be driven by mechanisms other than the DNA sequence: the methods being used for DNA motif identification are limited in their detection capabilities. Inversion symmetry needs to be a priority in the design of future DNA motif identification search engines.

### Inversion Symmetry of sNR DNA-Binding at 15-nt DNA Elements in the Genome

Although the core 13-nt ERE and HRE DNA elements for sNRs have been well defined, we are also interested in the contribution of the two nucleotides surrounding the 13-nt DNA elements in the genome, that is, the impact of these two additional nts on sNR binding in the genome (i.e., expanding the 13-nt ERE or HRE DNA element with 10 primary positions to a 15-nt ERE or HRE DNA element with 12 primary positions). Because the analysis of sNR DNA-binding at 13-nt ERE and HRE DNA elements was done by overlapping the location coordinates of each 0- to 5-nt variant ERE or HRE DNA element in the genome and the location coordinates of the ChIPSeq or ChIPExo peaks from each experiment, the counts of all sNR DNA-binding events at 15-nt ERE or HRE DNA elements in the genome are included in the 13-nt ERE and HRE DNA element analysis (i.e., every 13-nt ERE and HRE DNA element in the genome is part of a 15-nt ERE and HRE DNA element). For example, the 0-nt variant consensus palindromic 13-nt ERE DNA element (5′-GGTCAnnnTGACC-3′) occurred 1,202 times in a particular ER experiment (WT-E2-1hr.L4) (Figure S2). Each of those (1,202) 0-nt variant consensus palindromic 13-nt ERE DNA elements is part of a 15-nt ERE DNA element. Thus, analysis of ER DNA-binding at all possible 15-nt ERE DNA elements splits those (1,202) 0-nt variant consensus palindromic 13-nt ERE DNA elements into 16 categories (i.e., 4 nt possibilities in position 1 and 4 nt possibilities in position 12): 109 (1T), 155 (1G), 19 (1C), 13 (12G), 151 (12C), 110 (12A), 11 (1–12 TG), 67 (1–12 TC), 17 (1–12 GG), 305 (1–12 AT), 29 (1–12 TA), 107 (1–12 GC), 5 (1–12 CG), 14 (1–12 CC), 88 (1–12 GA), 2 (1–12 CA), totaling 1,202 (Figure S33). Thus, assessing the role of the flanking nucleotides in a 15-nt ERE or HRE DNA element on sNR binding in the genome requires segregating the counts from the 13-nt ERE and HRE DNA element analysis and displaying them separately.

First, we analyzed the DNA-binding preference of sNRs at the 16 possible 15-nt ERE or HRE DNA elements in the genome (i.e., 4 nt possibilities in position 1 and 4 nt possibilities in position 12) (Figure S33). sNR binding in the genome was most predominant when an adenine (A) was in position 1 and a thymine (T) was in position 12 of the 15-nt ERE DNA element (5′-AGGTCAnnnTGACCT-3′) for ERs and of the 15-nt HRE DNA element (5′-AGAACAnnnTGTTCT-3′) for KRs (Figure S33). This was observed in 154 ER experiments and 194 KR experiments, representing a wide variety of mouse tissues and human cell lines, and across multiple peak selection criteria (L4-L20) (Figure S33). Thus, these 15-nt ERE and HRE DNA elements (i.e., adenine [A] in position 1 and thymine [T] in position 12) represent the 0-nt variant consensus palindromic 15-nt ERE and HRE DNA elements for ERs and KRs (Figure S33).

Next, we repeated the same sNR DNA-binding analysis (as was done for sNR DNA-binding at 0- to 5-nt variant 13-nt ERE and HRE DNA elements in the genome) for the newly identified 0-nt variant consensus palindromic 15-nt ERE and HRE DNA elements and extending through 6-nt variant DNA elements (i.e., a 6-nt variant = a half-site for a 15-nt DNA element with 12 primary positions) in the genome (Figure S34). Analysis of sNR DNA-binding at 13-nt ERE and HRE DNA elements included the 1 0-nt variant consensus palindromic DNA element, 30 1-nt variant DNA elements (10 variant positions), 405 2-nt variant DNA elements (45 variant positions), 3,240 3-nt variant DNA elements (120 variant positions), 17,010 4-nt variant DNA elements (210 variant positions), and 61,236 5-nt variant DNA elements (252 variant positions), for a total of 81,922 DNA elements (Table 1; Figure S34). Analysis of sNR DNA-binding at 15-nt ERE and HRE DNA elements include the 1 0-nt variant consensus palindromic DNA element, 36 1-nt variant DNA elements (12 variant positions), 594 2-nt variant DNA elements (66 variant positions), 5,940 3-nt variant DNA elements (220 variant positions), 40,095 4-nt variant DNA elements (495 variant positions),192,456 5-nt variant DNA elements (792 variant positions), and 673,596 6-nt variant DNA elements (924 variant positions), for a total of 912,718 DNA elements (Figure S34). All data analyses completed for sNR DNA-binding at 0- to 5-nt variant 13-nt ERE and HRE DNA elements in the genome were repeated for sNR DNA-binding at 0- to 6-nt variant 15-nt ERE and HRE DNA elements in the genome (Figures S35–S61; Tables S21–S40). Note: a comparative figure summary between the 13-nt ERE and HRE DNA element analysis and the 15-nt ERE and HRE DNA element analysis can be found in the Supplemental Information.

As demonstrated for the 13-nt NRFE ERE and HRE DNA elements, sNR DNA-binding at 15-nt NRFE ERE and HRE DNA elements in the genome follows inversion symmetry (i.e., the number of sNR DNA-binding events at a particular DNA element in the genome is equivalent to the number of sNR DNA-binding events at its reverse-complement DNA element in the genome) (Figures S35 and S36). In addition, sNRs exhibit preferential DNA-binding at certain NRFE ERE and HRE DNA elements, this preference is determined by internal inversion symmetry within the DNA element (i.e., sNR binding in the genome is determined by the position of variants within the DNA element and its reverse-complement position) (Figures S35 and S36). Also, as with the 13-nt NRFE ERE and HRE DNA elements, sNR DNA-binding is most predominant at 15-nt NRFE ERE and HRE DNA elements versus at non-NRFE DNA elements in the genome (Figures S37 and S38).

Next, to sequentially track sNR DNA-binding at 0-nt to 6-nt variant 15-nt ERE and HRE DNA elements in the genome, we define a 6-nt variant DNA element by its six fixed positions, resulting in 924 half-site groups (i.e., categorizing the 673,596 6-nt variant ERE and HRE DNA elements into 924 half-site groups, defined by the six positions that are fixed/not varied) (Figure S34). The 924 half-site groups are symmetrically split into a set of 452 groups and their 452 reverse-complements, plus 20 groups that are “innate palindromes” (i.e., they are their own reverse-complement) (Figure S39). These 452 groups further split into four distinct subgroups (zero vacancies, one vacancy, two vacancies), plus the 20 innate palindromes that have three vacancies (Figure S39). The remaining 0-nt to 5-nt variant 15-nt DNA elements (1 0-nt variant consensus palindromic DNA element, 36 1-nt variant DNA elements, 594 2-nt variant DNA elements, 5,940 3-nt variant DNA elements, 40,095 4-nt variant DNA elements, and 192,456 5-nt variant DNA elements, for a total of 239,122 DNA elements) can be categorized into these same 924 half-site groups (i.e., six positions are fixed, allowing for *up to* six positions to be varied) (Figure S39). Each of the 924 half-site groups contain 1 (0-nt variant consensus palindromic DNA element), 18 (1-nt variant DNA elements), 135 (2-nt variant DNA elements), 540 (3-nt variant DNA elements), 1,215 (4-nt variant DNA elements), 1,458 (5-nt variant DNA elements), and 729 (6-nt variant DNA elements), for a total of 4,096 0- to 6-nt variant DNA elements per half-site group (Tables S21–S24). This allows all 912,718 0- to 6-nt variant DNA elements to be categorized into the 924 half-site groups, thus providing the ability to sequentially track sNR DNA-binding at 0- to 6-nt variant 15-nt ERE or HRE DNA elements in the genome. The x-axis is ordered by the symmetrically split 452 groups (left-to-right: zero vacancies, one vacancy, two vacancies) followed by their 452 reverse-complements, with the 20 half-site groups that are innate palindromes in the middle (Figures S40–S43; Tables S25 and S26). The x-axis is labeled by the reverse-complement vacancy position ID (primary label) and reverse-complement double occupant position ID (secondary label) (Figures S40–S43; Tables S25 and S26). The order of the reverse-complement vacancy position IDs for the ERE is 4-9 > 1-12 > 2-11 > 6-7 > 5-8 > 3-10 (Table S25), whereas that for the HRE is 3-10 > 1-12 > 6-7 > 2-11 > 5-8 > 4-9 (Table S26).

As expected, (S/N) analysis of sNR DNA-binding at 0- to 6-nt variant 15-nt ERE or HRE DNA elements in the genome (displayed by the 924 half-site groups) reveals a highly structured symmetrical DNA-binding profile that decays from 0- to 6-nt variant DNA elements (Figures S40–S43). This highly structured symmetrical sNR DNA-binding profile demonstrates (1) there is no sNR DNA-binding bias toward either the 5′ side (AGGTCA or AGAACA) or the 3′ side (TGACCT or TGTTCT) of the DNA element at any 1- to 6-nt variant 15-nt ERE or HRE DNA elements in the genome, (2) sNR binding in the genome follows inversion symmetry (i.e., the number of sNR DNA-binding events at a 1- to 6-nt variant DNA element in the genome is equivalent to the number of sNR DNA-binding events at its reverse-complement DNA element in the genome), (3) the specific DNA elements where sNRs bind in the genome is determined by internal inversion symmetry within the DNA element and this continues through 6-nt variant DNA elements (i.e., sNR binding in the genome is determined by the position of variants within the DNA element and its reverse-complement position), (4) sNR DNA-binding at 0- to 6-nt variant DNA elements in the genome requires all the information contained within the entire DNA element (i.e., on both sides of the 3-nt spacer), challenging previous suggestions that sNRs bind “palindrome half-sites” in the genome as individual monomers (Figures S40–S43). Moreover, this highly structured symmetrical sNR DNA-binding profile at 0- to 6-nt variant DNA elements in the genome was observed in 157 ER experiments and 194 KR experiments, representing a wide variety of mouse tissues and human cell lines and across multiple peak selection criteria (L4-L20) (Tables S27 and S28).

Because it is difficult to translate the 924 half-site groups to specific DNA elements, the absolute counts of each of the 912,718 0- to 6-nt variant ERE and HRE DNA element in an experiment can also be displayed by the position of the variant within the DNA element, rather than by the 924 half-site groups (Figures S44–S55). Analysis of sNR DNA-binding at 0- to 6-nt variant 15-nt ERE and HRE DNA elements in the genome shows a predominance of DNA-binding at DNA elements that have variants in these flanking nucleotides (positions 1 and 12) (Figures S44–S55). Thus, having fixed nucleotides in position 1 or position 12 is the least important of the 12 primary positions in a 15-nt ERE or HRE DNA element (Figures S44–S55). This sNR DNA-binding profile was observed in 157 ER experiments and 194 KR experiments, representing a wide variety of mouse tissues and human cell lines, and across multiple peak selection criteria (L4-L20) (Tables S29–S40).

As with the 13-nt DNA element analysis, GR-Dim has a significantly reduced DNA-binding signal at 15-nt NRFE HRE DNA elements, whereas the DNA-binding profiles of GR-Dim and wtGR are highly structured and converge onto the same ((S/N) value = 1) noise profile (demonstrating inherent structure in DNA-binding through 6-nt variant 15-nt HRE DNA elements in the genome) (Figures S56–S61). Likewise, the nearly identical structure of the DNA-binding curve of GR-Dim compared with wtGR at 0- to 6-nt variant HRE DNA elements in the genome (albeit with a significantly reduced amplitude at NRFE HRE DNA elements) demonstrates that the effect of the DBD mutation is a loss in residence time at the DNA element, rather than the inability to follow the same DNA-binding rules as wtGR (Figures S56–S61). Thus, the sNR DNA-binding rules, dictated by inversion symmetry, extend (i.e., are applicable) to DNA elements that do not support transcriptional activity. That is, the DNA-binding rules are obeyed, but the retention time of the sNR at these non-functional DNA elements is not long enough to initiate and/or maintain transcription. Therefore, functionality is determined at the individual nucleotide level, and the residence time (or strength of binding) is dictated by the number and position of variants within the DNA element (i.e., inversion symmetry and DNA sequence constraints).

Of importance, comparison of the sNR DNA-binding profile at the 81,922 0- to 5-nt variant 13-nt ERE or HRE DNA elements in the genome versus at the 912,718 0-nt to 6-nt variant 15-nt ERE or HRE DNA elements in the genome is almost equivalent, confirming that the DNA-binding rules established by the 13-nt DNA element analysis also apply to the 15-nt DNA element analysis (Figures S62–S71). That is, the information content in positions 1 and 12 of the 15-nt ERE and HRE DNA elements is less important than in the other 10 primary positions of the 15-nt ERE and HRE DNA elements, and thus the analysis completed for sNR DNA-binding at 13-nt ERE and HRE DNA elements was adequate to describe the DNA-binding mechanisms underlying sNR binding in the genome (Figures S62–S71). At best, fixing the flanking nucleotides of the 15-nt HRE or ERE DNA elements (i.e., adenine [A] in position 1 and thymine [T] in position 12) causes a few very subtle effects on the relative amplitudes of sNR DNA-binding at certain DNA elements (with variants in specific palindromic position pairs) in the genome (Figures S72–S77; Tables S41–S46).

To summarize, sNR binding in the genome is determined by (1) a set of rules defined by sNR DNA-binding at 1-nt variant ERE and HRE DNA elements based on which palindromic position pair the variant occupies (i.e., ER DNA-binding is increased at DNA elements with variants in palindromic position pair 3-8 or 1-10 [4-9 or 2-11 for the 15-nt DNA elements] of the ERE, whereas KR DNA-binding is increased at DNA elements with variants in palindromic position pair 2-9 [3-10 for the 15-nt DNA elements] of the HRE), and (2) a second distinct set of rules (functionally subordinate to and independent of the DNA-binding rules defined by sNR DNA-binding at 1-nt variant DNA elements) defined by sNR DNA-binding at 2-nt variant DNA elements based on how the position of the variants relate to each other (i.e., sNR DNA-binding is increased at DNA elements with variants that do not crossover the 3-nt spacer). These DNA-binding rules, dictated by inversion symmetry, continue through 5-nt variant DNA elements (6-nt variant DNA elements for the 15-nt DNA elements) in the genome, even though the sNR DNA-binding signal decays as the number the variants increases from 0- to 5-nt variant DNA elements (0- to 6-nt variant DNA elements for the 15-nt DNA elements). These sNR DNA-binding rules were observed in 157 ER experiments and 194 KR experiments, representing a wide variety of mouse tissues and human cell lines, and across multiple peak selection criteria (L4-L20). Of particular importance, there are numerous time course trials in these 351 experiments, confirming that these DNA-binding rules determine sNR binding in the genome for both initial sNR DNA-binding events (i.e., early time points) and subsequent sNR DNA-binding events (i.e., late time points) (Figures S78 and S79). The time course trials further demonstrate that hormone-mediated sNR DNA-binding at NRFE sites in the genome is retained the longest over time, in agreement with previous studies and hormone-mediated eRNA transcription (Figures S78 and S79) (Coons et al., 2017, Coons, 2017).

To further explain the importance of the progression and decay of sNR DNA-binding from 0- to 5-nt variant DNA elements (0-nt to 6-nt variant DNA elements for the 15-nt DNA elements) in the genome, we first looked at (S/N) analysis of sNR DNA-binding at the 0-nt variant consensus palindromic DNA element. (S/N) analysis of sNR DNA-binding at the 0-nt variant consensus palindromic DNA element results in the largest DNA-binding signal, but because this is a single DNA element there is no information to be extracted because all the states are equal. This is the state of equilibrium or maximal entropy. Because this is the state with the largest DNA-binding signal, the DNA-binding signal gives us a relative measure of the integrity of the data with respect to random noise. When a variant is added to the 0-nt variant consensus palindromic DNA element, this introduces an additional variable into the possible DNA element representations. Thus, this introduction of additional variables breaks the symmetry of the equilibrium state, thereby reducing the entropy of the system and the DNA-binding signal as the symmetry of the system is sequentially degraded. The reduction of entropy with each broken symmetry and the introduction of additional variables to the system is interpreted as an equivalent gain in information. Thus, the primary information generating process is the introduction of additional variants in certain palindromic position pairs of the DNA element (i.e., the first set of DNA-binding rules), with the relative positions of these variants playing a secondary, but not insignificant role (i.e., the second set of DNA-binding rules). For example, (S/N) analysis of sNR DNA-binding at 1-nt variant DNA elements in the genome introduces a variable (i.e., the position of the varied nucleotide) that is strictly associated with the position of the variant and its reverse-complement position (i.e., the palindromic position pairs). (S/N) analysis of sNR DNA-binding at 2-nt variant DNA elements in the genome introduces another variable (i.e., the relative position of the two variants) that is associated with how the position of the variants relate to each other. Thus, 2-nt variant DNA elements introduce another variable and additional restrictions (i.e., subrules) that generate an additional set of substructures, which are qualifiers to the superstructure rules defined by sNR DNA-binding at the 0-nt variant consensus palindromic DNA element and at the 1-nt variant DNA elements in the genome. That is, the primary DNA-binding rules are still fully intact and operational at each subsequent level (through 5-/6-nt variant DNA elements); the introduction of another variant at each level introduces additional variables. Thus, every subsequent level provides the opportunity to deduce more subrules that are subordinate to the superstructure rules (defined by sNR DNA-binding at the 0-nt variant consensus palindromic DNA element and at the 1-nt variant DNA elements in the genome). That is, the primary sNR DNA-binding rules, dictated by inversion symmetry, continue through 5-nt variant DNA elements (6-nt variant DNA elements for the 15-nt DNA elements) in the genome. Additional DNA-binding rules, generated by the introduction of additional variables, are subordinate to the primary sNR DNA-binding rules.

### Inversion Symmetry of the Single-Stranded Genome

The observation that sNR binding in the genome follows inversion symmetry was unexpected, prompting us to consider why this was the case. Therefore, we evaluated the number of times every DNA element (1- to 20-nt) occurs in the single-stranded mouse and human genome (Tables 6, S47, and S48). Thus, this includes all DNA elements from the four 1-nt DNA elements to the 1 trillion (1,099,511,627,776) 20-nt DNA elements (Tables 6, S47, and S48). Comparing the population count of every DNA element to the population count of its reverse-complement DNA element revealed a near-perfect inversion symmetry (i.e., 0.999 correlation coefficient) for all 1- to 20-nt DNA elements in the single-stranded mouse and human genome (Tables 6, S47, and S48). This result is Chargaff's second parity rule. This inversion symmetry structure is also maintained at the level of each individual chromosome, except for chromosome M (i.e., the mitochondrial DNA) (Tables S49 and S50).

**Table 6:** Inversion Symmetry of the Single-Stranded Mouse and Human Genome

Mouse Genome (mm10)								
DNA Element	Total DNA Elements in Genome (B = 4^A^)					Population Comparison		
A	B	C	D	E	F	G	H	I
Length (nt)	Expected	Actual	Missing	Actual	Missing	Correlation Coefficient	Slope	Y-Intercept
1	4	4	–	100%	0%	0.999997	0.999985	9,944.94
2	16	16	–	100%	0%	0.999991	0.999991	1,482.34
3	64	64	–	100%	0%	0.999992	0.999992	327.33
4	256	256	–	100%	0%	0.999992	0.999992	85.53
5	1,024	1,024	–	100%	0%	0.999990	0.999990	24.84
6	4,096	4,096	–	100%	0%	0.999986	0.999986	8.80
7	16,384	16,384	–	100%	0%	0.999977	0.999977	3.67
8	65,536	65,536	–	100%	0%	0.999958	0.999958	1.70
9	262,144	262,144	–	100%	0%	0.999927	0.999927	0.74
10	1,048,576	1,048,575	1	100%	0%	0.999891	0.999891	0.28
11	4,194,304	4,192,822	1,482	100%	0%	0.999858	0.999859	0.09
12	16,777,216	16,583,844	193,372	99%	1%	0.999831	0.999832	0.01
13	67,108,864	61,933,424	5,175,440	92%	8%	0.999805	0.999814	(0.07)
14	268,435,456	200,322,339	68,113,117	75%	25%	0.999782	0.999806	(0.20)
15	1,073,741,824	537,352,842	536,388,982	50%	50%	0.999767	0.999803	(0.34)
16	4,294,967,296	1,093,135,359	3,201,831,937	25%	75%	0.999776	0.999824	(0.59)
17	17,179,869,184	1,574,548,194	15,605,320,990	9%	91%	0.999800	0.999848	(0.81)
18	68,719,476,736	1,837,040,301	66,882,436,435	3%	97%	0.999810	0.999856	(0.92)
19	274,877,906,944	1,959,582,013	272,918,324,931	1%	99%	0.999808	0.999853	(0.96)
20	1,099,511,627,776	2,021,349,628	1,097,490,278,148	0%	100%	0.999799	0.999845	(0.97)

Human Genome (hg19)								
DNA Element	Total DNA Elements in Genome (B = 4^A^)					Population Comparison		
A	B	C	D	E	F	G	H	I
Length (nt)	Expected	Actual	Missing	Actual	Missing	Correlation Coefficient	Slope	Y-Intercept
1	4	4	–	100%	0%	0.999994	0.999976	17,191.60
2	16	16	–	100%	0%	0.999984	0.999984	2,908.31
3	64	64	–	100%	0%	0.999986	0.999986	643.10
4	256	256	–	100%	0%	0.999986	0.999986	161.07
5	1,024	1,024	–	100%	0%	0.999985	0.999985	42.31
6	4,096	4,096	–	100%	0%	0.999983	0.999983	12.00
7	16,384	16,384	–	100%	0%	0.999978	0.999978	3.88
8	65,536	65,536	–	100%	0%	0.999967	0.999967	1.46
9	262,144	262,144	–	100%	0%	0.999947	0.999947	0.58
10	1,048,576	1,048,576	–	100%	0%	0.999919	0.999919	0.22
11	4,194,304	4,193,313	991	100%	0%	0.999887	0.999887	0.08
12	16,777,216	16,609,017	168,199	99%	1%	0.999851	0.999852	0.01
13	67,108,864	62,296,994	4,811,870	93%	7%	0.999809	0.999820	(0.07)
14	268,435,456	202,655,673	65,779,783	75%	25%	0.999761	0.999797	(0.19)
15	1,073,741,824	546,364,018	527,377,806	51%	49%	0.999715	0.999780	(0.33)
16	4,294,967,296	1,130,819,799	3,164,147,497	26%	74%	0.999708	0.999807	(0.56)
17	17,179,869,184	1,682,092,708	15,497,776,476	10%	90%	0.999739	0.999854	(0.77)
18	68,719,476,736	2,015,311,971	66,704,164,765	3%	97%	0.999765	0.999885	(0.88)
19	274,877,906,944	2,181,567,011	272,696,339,933	1%	99%	0.999768	0.999898	(0.93)
20	1,099,511,627,776	2,265,704,990	1,097,245,922,786	0%	100%	0.999752	0.999899	(0.95)

Evaluation of the number of times every DNA element (1- to 20-nt) occurs in the single-stranded mouse and human genome. Column B: This includes all DNA elements from the four 1-nt DNA elements to the 1 trillion (1,099,511,627,776) 20-nt DNA elements. Column C and D: All possible 1- to 9-nt DNA elements exist in the mouse and human genome, and only one 10-nt DNA element is missing from the mouse genome. Column E: The absence of multiple DNA elements in the mouse and human genome occurs at 11-nt DNA elements and continues through 20-nt DNA elements, where less than 0.2% of all possible 20-nt DNA elements occur in the genome. Columns G–I: The equivalence of the population count of every DNA element to the population count of its reverse-complement DNA element in the single-stranded mouse and human genome was determined using the correlation coefficient, slope and y-intercept. See Tables S47–S50 for the population count of every 1- to 20-nt DNA elements in the single-stranded mouse and human genome, and at the level of each individual chromosome.

In addition to demonstrating the inherent inversion symmetry structure for all DNA elements in the single-stranded genome, we further demonstrate the absence of analog symmetries between reverse and complement pairs of DNA elements in the single-stranded genome (Tables S47–S50). However, the inversion symmetry structure should be viewed as a parity conservation law. The reverse pairs and the complement pairs are both binary or parity operations. That is, the reverse-of-the-reverse and the complement-of-the-complement leave the DNA element unchanged. Thus, an odd number of operations change the parity, and an even number of operations conserve the parity. For every DNA element, four sets of DNA elements can be generated (i.e., the original DNA element, the reverse-complement DNA element, the reverse DNA element, the complement DNA element), and there are six pairwise comparisons that can be assessed. In both cases where two parity operations are applied (i.e., the original DNA element versus the reverse-complement DNA element and the reverse DNA element versus the complement DNA element) and parity is conserved, the population counts have a correlation coefficient of unity (1) (Tables S47–S50), whereas when the comparisons differ by only a single (1) parity operation (i.e., the original DNA element versus the reverse DNA element, the original DNA element versus the complement DNA element, the reverse DNA element versus the reverse-complement DNA element, and the complement DNA element versus the reverse-complement DNA element) the results are significantly uncorrelated (Tables S47–S50). Yet, all four comparisons are identical numerically (Tables S47–S50). This suggests that the genome is governed by a very general parity conservation law.

This inversion symmetry structure for all DNA elements in the single-stranded genome holds for many species, both eukaryotes and prokaryotes (Shporer et al., 2016, Albrecht-Buehler, 2006). These findings suggest that the structural mechanisms (by which inversion symmetry ascribes TF DNA-binding and functionality) are universally applicable. That is, this analysis accounts for every possible *cis*-regulatory DNA element (for any TF) in the genome with 2- to 40-nt primary positions (i.e., a 20-nt element, followed by any arbitrary spacer, followed by its 20-nt reverse-complement element). For example, the 13-nt ERE and HRE DNA elements are a 5-nt DNA element (5-nt element, 3-nt spacer, 5-nt reverse-complement element), whereas the 15-nt ERE and HRE DNA elements are a 6-nt DNA element (6-nt element, 3-nt spacer, 6-nt reverse-complement element) (Tables S47–S50). Thus, the analysis of TF binding in the genome has expanded our understanding as to why the genome is organized in an inversion symmetry structure (i.e., Chargaff's second parity rule). Inversion symmetry is the DNA code that TFs use to interact with the genome, and dictates (in conjunction with known DNA sequence constraints) which of those interactions are functionally active.

Of particular importance is the observation that all possible 1- to 9-nt DNA elements exist in the mouse and human genome, and only one 10-nt DNA element is missing from the mouse genome (Tables 6, S47, and S48). The absence of multiple DNA elements in the mouse and human genome occurs at 11-nt DNA elements and continues through 20-nt DNA elements (less than 0.2% of all possible 20-nt DNA elements occur in the genome) (Tables 6, S47, and S48). That is, the number of DNA elements that occur in the genome, out of the total number of possible DNA elements, decreases from ∼100% to ∼0% from 11- to 20-nt DNA elements (Tables 6, S47, and S48). This is due to the fact that the sample size is limited to 3 billion nts, the size of the single-stranded genome. Hence, the inversion symmetry would be expected to be universal (beyond 20-nt DNA elements) if the DNA sample size were significantly larger than 3 billion nts.

To further illustrate that the structural mechanisms (by which inversion symmetry ascribes TF DNA-binding and functionality) are universally applicable, we repeated the same DNA-binding analysis as was done with the six sNRs (at ERE and HRE DNA elements) on the unrelated TF p53 (at p53RE DNA elements) in the genome. The tumor suppressor p53 is a TF that controls cellular stress responses (Tonelli et al., 2017). Once activated, p53 binds DNA and regulates gene expression programs that contribute to apoptosis, senescence, or cell-cycle arrest, preventing the dissemination of damaged cells (Tonelli et al., 2017). These processes are involved in tumor suppression, setting the selective pressure for p53 inactivation in tumors (Tonelli et al., 2017). We evaluated the DNA-binding of p53 at 0- to 5-nt variant DNA elements of its 10-nt consensus palindromic DNA element (p53RE) (5′-TGCCCGGGCA-3′) in the genome (Figure S80). Analysis of the 0- to 5-nt p53RE DNA elements include the 1 0-nt variant consensus palindromic DNA element, 30 1-nt variant DNA elements (10 variant positions), 405 2-nt variant DNA elements (45 variant positions), 3,240 3-nt variant DNA elements (120 variant positions), 17,010 4-nt variant DNA elements (210 variant positions), and 61,236 5-nt variant DNA elements (252 variant positions), for a total of 81,922 DNA elements (Figure S80). All 81,922 0- to 5-nt variant p53RE DNA elements can be categorized into the 252 half-site groups, providing the ability to sequentially track p53 DNA-binding at 0- to 5-nt variant p53RE DNA elements in the genome (Tables S51–S52). As expected, (S/N) analysis of p53 DNA-binding at 0- to 5-nt variant p53RE DNA elements in the genome reveals a highly structured symmetrical DNA-binding profile that decays from 0- to 5-nt variant p53RE DNA elements (Figure S81). This highly structured symmetrical p53 DNA-binding profile (i.e., the left side [126 half-site groups] versus the right side [126 reverse-complements]) demonstrates (1) that p53 binding in the genome follows inversion symmetry (i.e., the number of p53 DNA-binding events at a particular 1- to 5-nt variant DNA element in the genome is equivalent to the number of p53 DNA-binding events at its reverse-complement DNA element in the genome), and (2) the specific DNA elements where p53 binds in the genome is determined by internal inversion symmetry within the DNA element (i.e., p53 binding in the genome is determined by the position of variants within the DNA element and its reverse-complement position) (Figure S81). These p53 DNA-binding rules, dictated by inversion symmetry, continue through 5-nt variant p53RE DNA elements in the genome, even though the p53 DNA-binding signal decays as the number of variants increase from 0- to 5-nt variant p53RE DNA elements (Figure S81). Increased p53 DNA-binding at specific 1- to 5-nt variant p53RE DNA elements in the genome is determined by the reverse-complement vacancy in the palindromic position pair by the following hierarchy: 5-6 > 1-10 > 3-8 > 4-7 > 2-9 (Table S53). Moreover, this highly structured symmetrical p53 DNA-binding profile at 0- to 5-nt variant p53RE DNA elements in the genome was observed in 22 p53 experiments and across multiple peak selection criteria (L4-L20) (Table S54).

## Discussion

The information about when and where a gene is going to be expressed resides in DNA elements called *cis*-regulatory elements (Yáñez-Cuna et al., 2013). TFs direct gene expression by binding to *cis*-regulatory elements in the genome. Knowing where TFs bind in the genome and if that binding event will affect transcription is key to understanding gene regulation and biological responses. Despite there being a great deal of interest in understanding how TFs control gene expression, we still do not know how TFs select the appropriate regulatory targets from a large number of near-consensus DNA elements in the genome to elicit specific transcriptional and cellular responses. Knowing precisely how the binding of TFs to the genome turns genes on and off is vital to understand how different cell types function in health and disease.

It is commonly thought that variations in *cis*-regulatory DNA elements are responsible for changes in gene expression and phenotype. A recent study explored whether single nucleotide variants (SNV) in ER DNA-binding sites contribute to ER action through changes in the ER cistrome, thereby affecting disease progression (Bahreini et al., 2016). In this study, analysis of ER ChIPSeq data from MCF7 cells identified an intronic SNV in the IGF1R gene (rs62022087) that increased ER DNA-binding to this DNA element (compared with the wt-allele) (Bahreini et al., 2016). The wt-allele is a 2-nt variant ERE DNA element with variants in positions 7 and 8 (Bahreini et al., 2016). The SNV (rs62022087) is a 1-nt variant ERE DNA element, with a variant in position 8 (the nucleotide in position 7 has been mutated to the original nucleotide in position 7 of the 0-nt variant consensus palindromic ERE DNA element) (Bahreini et al., 2016). The conclusions in our study explain why the identified SNV results in increased ER DNA-binding at that element. The 2-nt variant ERE (wt-allele) that has variants in positions 7 and 8 exhibits a weak ER DNA-binding signal in 157 ER experiments (i.e., an average (S/N) value less than 4) (Figure S13; Table S12). This is because sNR DNA-binding is reduced when variants are in palindromic position pair 4-7 of the ERE or HRE DNA elements; its variation results in a deleterious effect (Tables S7 and S8). Thus, the reduced ER DNA-binding at the wt-allele (as shown in their study) is due to a variant occurring in position 7 of the ERE (Bahreini et al., 2016). In contrast, ER DNA-binding is increased at DNA elements that have a variant in palindromic position pair 3-8 in 157 ER experiments (i.e., an average (S/N) value above 50) (Figure S12; Table S11). Thus, the increased ER DNA-binding at the intronic SNV (rs62022087) (as shown in their study) is due to the 1-nt variant in position 8 of the ERE (Bahreini et al., 2016). In another study, ER^enh588^ has been shown to interact with the promoter region of the CyclinD1 (CCND1) gene, and regulates CCND1 expression by ER binding at a 1-nt variant ERE DNA element (a variant in position 1) in enhancer 588 (Korkmaz et al., 2016). According to the study, mutating this 1-nt variant ERE DNA element in enhancer 588 to a 2-nt variant ERE (inserting a variant in position 10) still allowed for estradiol-mediated activity of CCND1. However, this activity was diminished when the 1-nt variant ERE DNA element in enhancer 588 was mutated to a 5-nt variant ERE DNA element (Korkmaz et al., 2016). These studies demonstrate why decoding the inversion symmetry underlying TF DNA-binding specificity and functionality in the genome is key to understanding how TFs select the appropriate regulatory targets from a large number of similar DNA elements in the genome to elicit specific transcriptional and cellular responses.

In our study, we demonstrate that TF binding in the genome follows inversion symmetry (i.e., the number of TF DNA-binding events at a particular DNA element in the genome is equivalent to the number of TF DNA-binding events at its reverse-complement DNA element in the genome). In addition, the specific DNA elements where TFs bind in the genome is determined by internal inversion symmetry within the DNA element (i.e., TF binding in the genome is determined by the position of variants within the DNA element and its reverse-complement position), hence requiring all the information contained within the entire DNA element (i.e., on both sides of the 3-nt spacer). This confirms that the majority of TF DNA-binding events that contain “nonspecific” (low-affinity) binding sites are in fact DNA sequence dependent and account for the large excess of non-NRFE (non-functional) binding sites observed in ChIPSeq and ChIPExo experiments (∼55% and ∼65%) (Coons et al., 2017). Thus, TF DNA-binding at non-NRFEs reflects more than just “opportunistic” DNA-binding (i.e., interacting in areas of spontaneous accessible chromatin) or artifacts of the ChIPSeq or ChIPExo technology. TF DNA-binding at non-NRFEs is a direct extension of TF DNA-binding at NRFEs, but occurs in a domain that has insufficient binding strength to initiate and/or maintain transcription.

TF binding in the genome is determined by (1) a set of rules defined by TF DNA-binding at 1-nt variant DNA elements based on which palindromic position pair the variant occupies (e.g., ER DNA-binding is increased at DNA elements with variants in palindromic position pair 3-8 or 1-10 of the ERE, whereas KR DNA-binding is increased at DNA elements with variants in palindromic position pair 2-9 of the HRE), and (2) a second distinct set of rules (functionally subordinate to and independent of the DNA-binding rules defined by TF DNA-binding at 1-nt variant DNA elements) defined by TF DNA-binding at 2-nt variant DNA elements based on how the position of the variants relate to each other (i.e., TF DNA-binding is increased at DNA elements with variants that do not crossover the 3-nt spacer). These DNA-binding rules, dictated by inversion symmetry, continue through 5- and 6-nt variant DNA elements in the genome, even though the TF DNA-binding signal decays as the number the variants increases from 0- to 5- and 6-nt variant DNA elements. The basis of these DNA-binding rules follows specific algebraic relationships. Thus, these DNA-binding rules quantitatively define how TFs select the appropriate regulatory targets from a large number of similar DNA elements in the genome to elicit specific transcriptional and cellular responses. Furthermore, the variables that define these algebraic relationships assume specific numerical values for each different or distinct TF, and these algebraic variables can be quantified or measured in laboratory experiments. Thus, this study goes beyond a theoretical analysis and defines a measurable experimental scientific methodology. That is, we have defined a mathematical formula that represents the outcome observed in hundreds of ChIPSeq and ChIPExo experiments.

Historically, the “half-site” has been defined as the two physical nucleotide sequences on the left-hand or right-hand side of the symmetrical 0-nt variant consensus palindromic DNA element (e.g., GGTCA or TGACC in the ERE, GAACA or TGTTC in the HRE), which we call “palindrome half-sites,” that is, a linear string of DNA nucleotides defining the physical entity. Our analysis shows that “half-sites” do not need to be confined to a contiguous set of nucleotides, because replacing any reverse-complement nucleotide from either the left-hand or right-hand half-site results in comparable TF DNA-binding to the “palindrome half-sites.” This generalizes the definition of a “half-site” because we retain the DNA-binding characteristics of the “palindrome half-sites” without the constraint of nucleotide proximity. Thus, specific focus on TF DNA-binding at “palindrome half-sites” is an elementary example of a more complex TF DNA-binding structure, and continued fixation on these anomalous data points limits ones understanding of the processes and properties of TF binding in the genome.

It is perplexing that the inversion symmetry that underlies TF DNA-binding specificity and functionality in the genome, observed in hundreds of ChIPSeq and ChIPExo experiments, has not been detected using current DNA motif identification algorithms. Our analysis reveals that the resolution capability of PWM-based DNA motif identification is limited. An overreliance on these PWM-based algorithms has promoted the misconception that the majority of TF DNA-binding events in the genome are driven by mechanisms other than the DNA sequence. This overreliance has also led to the misconception that sNRs can indiscriminately bind to the *cis*-regulatory DNA element of other nuclear receptors. Furthermore, different *cis*-regulatory DNA elements have been delineated for the different members of the KR family. Analysis of KR DNA-binding at 81,922 0- to 5-nt variant 13-nt HRE DNA elements and at 912,718 0- to 6-nt variant 15-nt HRE DNA elements in the genome (in 194 KR ChIPSeq and ChIPExo experiments, representing a wide variety of mouse tissues and human cell lines, and across multiple peak selection criteria [L4-L20]), quantitatively demonstrates that all members of the KR family follow the same DNA-binding rules and thus bind the same DNA elements. This is further illustrated by the equivalent structure of the DNA-binding curves observed in AR and an AR DBD mouse model (SPARKI), where the second zinc finger in the AR DBD is replaced by that of the GR, at 81,922 0- to 5-nt variant 13-nt HRE DNA elements and at 912,718 0- to 6-nt variant 15-nt HRE DNA elements in the genome (Figures S82–S91). It is important to recognize the caveats associated with current PWM-based DNA motif identification algorithms, and careful considerations should be given with regard to any conclusions drawn from their output. Furthermore, inversion symmetry needs to be a priority in the design of future DNA motif identification search engines.

The concept of sNRs binding to specific so-called “negative response elements” in the genome as a mode of transcriptional repression has been previously described: nHRE is TYACNnnnTGAYC and nERE is TAACAnnnCGTCC (Beato, 1989) (Chen et al., 2001). Both of these negative response elements represent 5-nt variant 13-nt DNA elements: the nHRE has variants in positions 1-2-5-8-9 (positions 3-4-6-7-10 are fixed) and the nERE has variants in positions 1-2-3-6-8 (positions 4-5-7-9-10 are fixed). Thus, there are a total of 243 “negative response element” sequences for the nHRE (Tables S2 and S4) and 243 “negative response element” sequences for the nERE (Tables S1 and S3), that is, the three alternative nucleotide possibilities (variants) at each of those five variant positions. (S/N) analysis of KR DNA-binding in 194 KR experiments, representing a wide variety of mouse tissues and human cell lines, and across multiple peak selection criteria (L4-L20) results in an average KR DNA-binding signal of 0.7 (standard deviation = 0.2) at these 243 nHRE DNA elements in the genome (Table S20). Likewise, (S/N) analysis of ER DNA-binding in 157 ER experiments, representing a wide variety of mouse tissues and human cell lines, and across multiple peak selection criteria (L4-L20) results in an average ER DNA-binding signal of 1.2 (standard deviation = 0.4) at these 243 nERE DNA elements in the genome (Table S15). This confirms that sNR DNA-binding does not occur at “negative response elements” in the genome above (in any statistically significant measure) that which would be expected to occur at random. Furthermore, GR-Dim, a GR DBD mutant mouse model that exhibits neither hormone-mediated gene induction nor repression, has a DNA-binding signal (S/N) of 1.1 (standard deviation = 0.1) at these 243 nHRE DNA elements, whereas wtGR has a DNA-binding signal (S/N) of 0.9 (standard deviation = 0.1) at these 243 nHRE DNA elements (Figure 5). This confirms that sNRs do not bind “negative response elements” in the genome above what would be expected to occur at random, and even if one were to look at the “negative response elements” bound by sNRs that occur at random in the genome, GR-Dim is bound there as well. Thus, these DNA elements are unlikely to be responsible for hormone-mediated gene repression. In addition, in a previous study we showed that all hormone-mediated gene expression, both gene induction and repression, requires sNR DNA-binding to NRFEs (Coons et al., 2017). We further demonstrated that hormone-mediated NRFE binding by sNRs in the genome results directly in gene induction; the coregulators that are recruited to those NRFE sites are being redistributed from sites that are undergoing active transcription (Coons et al., 2017). Thus, this redistribution process consequently results in an effective repression of genes driven by those sites (Coons et al., 2017). This suggests that hormone-mediated sNR DNA-binding at NRFE DNA elements in the genome is a 2-fold mechanistic process. First, it directly causes gene induction, and second, indirectly, it causes gene repression.

Genome-wide ChIP studies provide a snapshot of TF occupancy in the genome. In a previous study we determined that only a small fraction of all TF chromatin-interacting events observed in ChIPSeq and ChIPExo experiments is associated with transcriptional activity, and this functionality is restricted to DNA elements that vary from the consensus palindromic DNA element by one or two nts (i.e., NRFEs) (Coons et al., 2017). sNR DBD mutant mouse models confirm that whereas DNA-binding at NRFE DNA elements (i.e., the 0-nt variant consensus palindromic DNA element, 1-nt variant DNA elements, and 2-nt variant DNA elements) are the only functional DNA-binding sites, DNA-binding at non-NRFE DNA elements in the genome are subject to the same DNA-binding rules (dictated by inversion symmetry) that govern NRFE DNA elements. This DNA-binding structure, observed at non-NRFE DNA elements, is independent of peaks that contain NRFE DNA elements (Tables S67 and S71). Thus, these DNA-binding rules extend (i.e., are applicable) to DNA elements that do not support transcriptional activity. Furthermore, because the DNA-binding curve of sNR DBD mutants maintain the same structure compared with wild-type (albeit with a significantly reduced amplitude at NRFEs), this demonstrates that the effect of the DBD mutation is a loss in residence time on the DNA. That is, the DNA-binding rules are obeyed, but the retention time of the TF at non-NRFE DNA elements is not long enough to initiate and/or maintain transcription. Thus, functional (NRFE) versus non-functional (non-NRFE) DNA-binding is determined at the individual nucleotide level, and the residence time (or strength of binding) is dictated by the number and position of variants within the DNA element (i.e., inversion symmetry and DNA sequence constraints). Collectively, this suggests that the retention time of sNR DNA-binding at non-NRFE DNA elements in the genome is not long enough to initiate and/or maintain transcription, and hence, is non-functional. Thus, although the “non-specific” (low-affinity) non-NRFE DNA elements may not be capable of retaining the TF long enough to ensure activation, they may instead be used to maintain a high concentration of the TF in the vicinity of the functional binding sites (Boeva, 2016). The recent emergence of the CRISPR/Cas9 and CRISPRi genome editing technology to assess the effects of mutating many enhancers simultaneously, while maintaining the native genomic context of the binding site, provides a mechanism to explore this new dimension of TF action.

In addition to decoding the inversion symmetry underlying TF DNA-binding specificity and functionality in the genome, we also demonstrate that the population of every DNA element (1- to 20-nt) in the single-stranded genome is equivalent to the population of its reverse-complement DNA element in the single-stranded genome. Thus, the inversion symmetry observed for TF binding in the genome represents an inherent inversion symmetry structure for all DNA elements in the genome. This property is maintained at the level of each individual chromosome. These findings suggest that the structural mechanisms (by which inversion symmetry ascribes TF DNA-binding and functionality) are universally applicable. To further illustrate the generality of the mechanism, we demonstrate that DNA-binding of the unrelated TF p53 in the genome also follows inversion symmetry (i.e., the number of TF DNA-binding events at a particular DNA element in the genome is equivalent to the number of TF DNA-binding events at its reverse-complement DNA element in the genome), and the specific DNA elements where p53 binds in the genome is determined by internal inversion symmetry within the DNA element (i.e., TF binding in the genome is determined by the position of variants within the DNA element and its reverse-complement position). Thus, analysis of TF binding in the genome has expanded our understanding as to why the genome is organized in an inversion symmetry structure (i.e., Chargaff's second parity rule). Inversion symmetry is the DNA code that TFs use to interact with the genome and dictates (in conjunction with known DNA sequence constraints) which of those interactions are functionally active.

### Conclusion

Gene regulatory programs are encoded in the sequence of the DNA. As in any other language, decoding the instructions between DNA and gene expression is the key for understanding transcriptional regulation. Without understanding the grammar of transcriptional regulation, one cannot tell which DNA sequence changes affect gene expression and how. The generalization of the second Chargaff rule states that the counts of any string of nucleotides on a single chromosomal strand equals the counts of its reverse-complement string of nucleotides on that same single chromosomal strand. This inversion symmetry holds for many species, both eukaryotes and prokaryotes. Our study quantitatively defines how TFs use inversion symmetry to select the appropriate regulatory targets from a large number of similar DNA elements in the genome to elicit specific transcriptional and cellular responses, that is, why a TF binds to the specific DNA elements in the genome where it does and whether that binding event ultimately affects transcriptional output. These data demonstrate that functional versus non-functional DNA-binding is dictated at the individual nucleotide level, and the residence time (or strength of binding) is determined by the number and position of variants within the DNA element (i.e., inversion symmetry and DNA sequence constraints). Furthermore, the inversion symmetry observed for TF binding in the genome represents an inherent inversion symmetry structure for all DNA elements in the genome. These findings suggest that the structural mechanisms (by which inversion symmetry ascribes TF DNA-binding and functionality) are universally applicable. Hence, analysis of TF binding in the genome has expanded our understanding as to why the genome is organized in an inversion symmetry structure (i.e., Chargaff's second parity rule). Inversion symmetry is the DNA code that TFs use to interact with the genome and dictates (in conjunction with known DNA sequence constraints) which of those interactions are functionally active. The use of information theory and techniques in genomic analysis and modeling is therefore vital in future genomic studies.

## Methods

All methods can be found in the accompanying Transparent Methods supplemental file.

## Data and Software Availability

Data available from the following links https://data.mendeley.com/datasets/8ks7pm2jw6/1 and https://archive.org/details/Laurel_Coons_Research_Data_Set_2019_05.

## Supplemental Information

Document S1. Transparent Methods and Figures S1–S91

Tables S1–S20. 13-nt ERE and HRE DNA Element Analysis

Tables S21–S40. 15-nt ERE and HRE DNA Element Analysis

Tables S41–S46. Transform the 13-nt DNA Element Analysis to the 15-nt DNA Element Analysis

Table S47–S50. Inversion Symmetry of the Single-Stranded Mouse and Human Genome

Tables S51–S54. p53 DNA Element Analysis

Tables S55–S63. Number of DNA Elements (ERE, HRE, p53RE) in the Mouse and Human Genome

Tables S64–S71. Data Analysis Example (WT-E2-1hr.L4)

Tables S72–S74. Genome Assembly of the Mouse and Human Genome

Tables S75. Script Codes for Data Analyses

Data S1. All Detailed Data and Statistics Associated with Every Figure
