# Supplementary material for: Decoding the Inversion Symmetry Underlying Transcription Factor DNA-Binding Specificity and Functionality in the Genome
Source: iScience. 2019 Apr 6;15:552–91. doi: 10.1016/j.isci.2019.04.006 (PMC6542189; doi:10.1016/j.isci.2019.04.006)
Supplement: Document S1. Transparent Methods and Figures S1–S91 [file mmc1.pdf]

ISCI, Volume *15*

Supplemental Information

Decoding the Inversion Symmetry Underlying Transcription Factor DNA-Binding Specificity and Functionality in the Genome

Laurel A. Coons, Adam B. Burkholder, Sylvia C. Hewitt, Donald P. McDonnell, and Kenneth S. Korach

Supplemental Information

Decoding the Inversion Symmetry Underlying Transcription Factor DNA-Binding Specificity and Functionality in the Genome

This document contains the following supplemental information:

Contents

Figure S1-S25 Descriptions: 13-nt ERE and HRE DNA Element Analysis ... 7
Figure S1. Inversion Symmetry of sNR DNA-Binding at 2-nt Variant EREs and HREs in the Genome (%) ... 12
Figure S2. (S/N) analysis of 0-nt to 5-nt Variant EREs in ER ChIPSeq Peaks (# of Variants) ... 13
Figure S3. (S/N) analysis of 0-nt to 5-nt Variant HREs in KR ChIPSeq Peaks (# of Variants) ... 14
Figure S4. (S/N) analysis of 0-nt to 5-nt Variant HREs in GR ChIPSeq Peaks (252 Half-Site Groups) ... 15
Figure S5. (S/N) analysis of 0-nt to 5-nt Variant HREs in PR ChIPSeq Peaks (252 Half-Site Groups) ... 16
Figure S6. Quantify the 5 Discrete States of ER DNA-Binding at 1-nt Variant EREs in the Genome (3,2,0,0,0) ... 17
Figure S7. Quantify the 3 Discrete States of KR DNA-Binding at 1-nt Variant HREs in the Genome (4,1,0,0,0) ... 18
Figure S8. Algebraic Equations of the 3-State DNA Element (4,1,0,0,0) ... 19
Figure S9. Algebraic Equations of the 5-State DNA Element (3,2,0,0,0) ... 20
Figure S10. Algebraic Equations of the 9-State DNA Element (3,1,1,0,0) ... 21
Figure S11. Algebraic Equations of the 11-State, 25-State, 51-State DNA Elements (2,2,1,0,0) (2,1,1,1,0) (1,1,1,1,1) ... 22
Figure S12. (S/N) analysis of 1-nt Variant EREs in ER ChIPSeq Peaks (Variant Position) ... 23
Figure S13. (S/N) analysis of 2-nt Variant EREs in ER ChIPSeq Peaks (Variant Position) ... 24
Figure S14. (S/N) analysis of 3-nt Variant EREs in ER ChIPSeq Peaks (Variant Position) ... 25
Figure S15. (S/N) analysis of 4-nt Variant EREs in ER ChIPSeq Peaks (Variant Position) ... 26
Figure S16. (S/N) analysis of 5-nt Variant EREs in ER ChIPSeq Peaks (Variant Position) ... 27
Figure S17. (S/N) analysis of 1-nt Variant HREs in KR ChIPSeq Peaks (Variant Position) ... 28
Figure S18. (S/N) analysis of 2-nt Variant HREs in KR ChIPSeq Peaks (Variant Position) ... 29
Figure S19. (S/N) analysis of 3-nt Variant HREs in KR ChIPSeq Peaks (Variant Position) ... 30
Figure S20. (S/N) analysis of 4-nt Variant HREs in KR ChIPSeq Peaks (Variant Position) ... 31
Figure S21. (S/N) analysis of 5-nt Variant HREs in KR ChIPSeq Peaks (Variant Position) ... 32
Figure S22. (S/N) analysis of 0-nt to 5-nt Variant HREs in GR and GR-Dim ChIPSeq Peaks #2 (252 Half-Site Groups) ... 33
Figure S23. (S/N) analysis of 0-nt to 5-nt Variant HREs in GR and GR-Dim ChIPExo Peaks #1 (252 Half-Site Groups) ... 34
Figure S24. (S/N) analysis of 0-nt to 5-nt Variant HREs in GR and GR-Dim ChIPExo Peaks #2 (252 Half-Site Groups) ... 35
Figure S25. (S/N) analysis of 1-nt to 5-nt Variant HREs in GR and GR-Dim ChIPExo Peaks (Variant Position) ... 36

Figure S26-S32 Descriptions: Inversion Symmetry Detection Methodology (Position Weight Matrices) ... 37
Figure S26. PWMs Detection of the ERE DNA Motif in ER ChIPSeq Peaks ... 40
Figure S27. PWMs Detection of the ARE DNA Motif in KR ChIPSeq Peaks ... 41
Figure S28. PWMs Detection of the GRE DNA Motif in KR ChIPSeq Peaks ... 42
Figure S29. PWMs Detection of the GRE 2 DNA Motif in KR ChIPSeq Peaks ... 43
Figure S30. PWMs Detection of the PRE DNA Motif in KR ChIPSeq Peaks ... 44
Figure S31. PWMs Detection of the ARE Palindrome Half-Site DNA Motif in KR ChIPSeq Peaks ... 45
Figure S32. PWMs Detection of the Different HRE Motifs (ARE, GRE, GRE 2, PRE, ARE Half) in KR ChIPSeq Peaks ... 46

Figure S33-S61 Descriptions: 15-nt ERE and HRE DNA Element Analysis 48
Figure S33. 0-nt Variant 15-nt ERE and HRE Identification in ER and KR ChIPSeq Peaks (16 possibilities) 54
Figure S34. Number of 0-nt to 6-nt Variant 15-nt ERE and HRE DNA Elements in the Mouse and Human Genome 55
Figure S35. Inversion Symmetry of sNR DNA-Binding at 1-nt Variant EREs and HREs in the Genome (%) 56
Figure S36. Inversion Symmetry of sNR DNA-Binding at 2-nt Variant EREs and HREs in the Genome (%) 57
Figure S37. (S/N) analysis of 0-nt to 6-nt Variant EREs in ER ChIPSeq Peaks (# of Variants) 58
Figure S38. (S/N) analysis of 0-nt to 6-nt Variant HREs in KR ChIPSeq Peaks (# of Variants) 59
Figure S39. 924 Half-Site Groups 60
Figure S40. (S/N) analysis of 0-nt to 6-nt Variant EREs in ER ChIPSeq Peaks (924 Half-Site Groups) 66
Figure S41. (S/N) analysis of 0-nt to 6-nt Variant HREs in AR ChIPSeq Peaks (924 Half-Site Groups) 67
Figure S42. (S/N) analysis of 0-nt to 6-nt Variant HREs in GR ChIPSeq Peaks (924 Half-Site Groups) 68
Figure S43. (S/N) analysis of 0-nt to 6-nt Variant HREs in PR ChIPSeq Peaks (924 Half-Site Groups) 69
Figure S44. (S/N) analysis of 1-nt Variant EREs in ER ChIPSeq Peaks (Variant Position) 70
Figure S45. (S/N) analysis of 2-nt Variant EREs in ER ChIPSeq Peaks (Variant Position) 71
Figure S46. (S/N) analysis of 3-nt Variant EREs in ER ChIPSeq Peaks (Variant Position) 72
Figure S47. (S/N) analysis of 4-nt Variant EREs in ER ChIPSeq Peaks (Variant Position) 73
Figure S48. (S/N) analysis of 5-nt Variant EREs in ER ChIPSeq Peaks (Variant Position) 74
Figure S49. (S/N) analysis of 6-nt Variant EREs in ER ChIPSeq Peaks (Variant Position) 75
Figure S50. (S/N) analysis of 1-nt Variant HREs in KR ChIPSeq Peaks (Variant Position) 76
Figure S51. (S/N) analysis of 2-nt Variant HREs in KR ChIPSeq Peaks (Variant Position) 77
Figure S52. (S/N) analysis of 3-nt Variant HREs in KR ChIPSeq Peaks (Variant Position) 78
Figure S53. (S/N) analysis of 4-nt Variant HREs in KR ChIPSeq Peaks (Variant Position) 79
Figure S54. (S/N) analysis of 5-nt Variant HREs in KR ChIPSeq Peaks (Variant Position) 80
Figure S55. (S/N) analysis of 6-nt Variant HREs in KR ChIPSeq Peaks (Variant Position) 81
Figure S56. (S/N) analysis of 0-nt to 6-nt Variant HREs in GR and GR-Dim ChIPSeq Peaks #1 (924 Half-Site Groups) 82
Figure S57. (S/N) analysis of 0-nt to 6-nt Variant HREs in GR and GR-Dim ChIPSeq Peaks #2 (924 Half-Site Groups) 83
Figure S58. (S/N) analysis of 0-nt to 6-nt Variant HREs in GR and GR-Dim ChIPExo Peaks #1 (924 Half-Site Groups) 84
Figure S59. (S/N) analysis of 0-nt to 6-nt Variant HREs in GR and GR-Dim ChIPExo Peaks #2 (924 Half-Site Groups) 85
Figure S60. (S/N) analysis of 1-nt to 6-nt Variant HREs in GR and GR-Dim ChIPSeq Peaks (Variant Position) 86
Figure S61. (S/N) analysis of 1-nt to 6-nt Variant HREs in GR and GR-Dim ChIPExo Peaks (Variant Position) 87

Figure S62-S71 Descriptions: Compare the 13-nt DNA Element Analysis to the 15-nt DNA Element Analysis 88
Comparative Figure Summary: 13-nt DNA Element Analysis vs. 15-nt DNA Element Analysis 88
Figure S62. Compare (S/N) analysis of 1-nt Variant EREs in ER ChIPSeq Peaks (13-nt vs. 15-nt) 91
Figure S63. Compare (S/N) analysis of 2-nt Variant EREs in ER ChIPSeq Peaks (13-nt vs. 15-nt) 92
Figure S64. Compare (S/N) analysis of 3-nt Variant EREs in ER ChIPSeq Peaks (13-nt vs. 15-nt) 93
Figure S65. Compare (S/N) analysis of 4-nt Variant EREs in ER ChIPSeq Peaks (13-nt vs. 15-nt) 94
Figure S66. Compare (S/N) analysis of 5-nt Variant EREs in ER ChIPSeq Peaks (13-nt vs. 15-nt) 95
Figure S67. Compare (S/N) analysis of 1-nt Variant HREs in KR ChIPSeq Peaks (13-nt vs. 15-nt) 96
Figure S68. Compare (S/N) analysis of 2-nt Variant HREs in KR ChIPSeq Peaks (13-nt vs. 15-nt) 97
Figure S69. Compare (S/N) analysis of 3-nt Variant HREs in KR ChIPSeq Peaks (13-nt vs. 15-nt) 98

Figure S70. Compare (S/N) analysis of 4-nt Variant HREs in KR ChIPSeq Peaks (13-nt vs. 15-nt) 99
Figure S71. Compare (S/N) analysis of 5-nt Variant HREs in KR ChIPSeq Peaks (13-nt vs. 15-nt) 100

Figure S72-S77 Descriptions: Transform the 13-nt DNA Element Analysis to the 15-nt DNA Element Analysis 101
Figure S72. Transform the 13-nt DNA Element Analysis to the 15-nt DNA Element Analysis 103
Figure S73. Transform the 13-nt DNA Element Analysis to the 15-nt DNA Element Analysis (1-nt Variant EREs) 104
Figure S74. Transform the 13-nt DNA Element Analysis to the 15-nt DNA Element Analysis (2-nt Variant EREs) 105
Figure S75. Transform the 13-nt DNA Element Analysis to the 15-nt DNA Element Analysis (3-nt Variant EREs) 106
Figure S76. Transform the 13-nt DNA Element Analysis to the 15-nt DNA Element Analysis (4-nt Variant EREs) 107
Figure S77. Transform the 13-nt DNA Element Analysis to the 15-nt DNA Element Analysis (5-nt Variant EREs) 108

Figure S78-S79 Descriptions: sNR DNA-Binding at 13-nt and 15-nt DNA Elements in the Genome (Time-Course) 109
Figure S78. (S/N) analysis of 0-nt to 5-nt Variant EREs in ER (Time-Course) ChIPSeq Peaks (252 Half-Site Groups) 110
Figure S79. (S/N) analysis of 0-nt to 6-nt Variant EREs in ER (Time-Course) ChIPSeq Peaks (924 Half-Site Groups) 111

Figure S80-S81 Descriptions: p53 DNA Element Analysis 112
Figure S80. Number of 0-nt to 5-nt Variant p53RE DNA Elements in the Mouse Genome 113
Figure S81. (S/N) analysis of 0-nt to 5-nt Variant p53REs in p53 ChIPSeq Peaks (252 Half-Site Groups) 114

Figure S82-S91 Descriptions: sNR DNA-Binding at 13-nt and 15-nt DNA Elements in the Genome (AR and AR-SPARKI) 115
Figure S82. (S/N) analysis of 0-nt to 5-nt Variant HREs in AR and AR-SPARKI ChIPSeq Peaks #1 (252 Half-Site Groups) 117
Figure S83. (S/N) analysis of 0-nt to 5-nt Variant HREs in AR and AR-SPARKI ChIPSeq Peaks #2 (252 Half-Site Groups) 118
Figure S84. (S/N) analysis of 0-nt to 5-nt Variant HREs in AR and AR-SPARKI ChIPSeq Peaks #M (252 Half-Site Groups) 119
Figure S85. (S/N) analysis of 0-nt to 6-nt Variant HREs in AR and AR-SPARKI ChIPSeq Peaks #1 (924 Half-Site Groups) 120
Figure S86. (S/N) analysis of 0-nt to 6-nt Variant HREs in AR and AR-SPARKI ChIPSeq Peaks #2 (924 Half-Site Groups) 121
Figure S87. (S/N) analysis of 0-nt to 6-nt Variant HREs in AR and AR-SPARKI ChIPSeq Peaks #M (924 Half-Site Groups) 122
Figure S88. (S/N) analysis of 1-nt to 5-nt Variant HREs in AR and AR-SPARKI ChIPSeq Peaks #1 & #2 (Variant Position) 123
Figure S89. (S/N) analysis of 1-nt to 5-nt Variant HREs in AR and AR-SPARKI ChIPSeq Peaks #M (Variant Position) 124
Figure S90. (S/N) analysis of 1-nt to 6-nt Variant HREs in AR and AR-SPARKI ChIPSeq Peaks #1 & #2 (Variant Position) 125
Figure S91. (S/N) analysis of 1-nt to 6-nt Variant HREs in AR and AR-SPARKI ChIPSeq Peaks #M (Variant Position) 126

Data S1. All Detailed Data and Statistics Associated with Every Figure 127

Table S1-S75 Descriptions 128
Table S1-S20. 13-nt ERE and HRE DNA Element Analysis 128
Table S1. Categorize 0-nt to 5-nt Variant 13-nt EREs into 252 Half-Site Groups (Variant Position) 128
Table S2. Categorize 0-nt to 5-nt Variant 13-nt HREs into 252 Half-Site Groups (Variant Position) 128
Table S3. Categorize 0-nt to 5-nt Variant 13-nt EREs into 252 Half-Site Groups (Sequence) 128
Table S4. Categorize 0-nt to 5-nt Variant 13-nt HREs into 252 Half-Site Groups (Sequence) 128

Table S5. X-Axis Order of 252 ERE Half-Site Groups 128
Table S6. X-Axis Order of 252 HRE Half-Site Groups 128
Table S7. (S/N) analysis of 0-nt to 5-nt Variant EREs in ER ChIPSeq Peaks (252 Half-Site Groups) [157] 129
Table S8. (S/N) analysis of 0-nt to 5-nt Variant HREs in KR ChIPSeq Peaks (252 Half-Site Groups) [194] 129
Table S9. Quantify the 5 Discrete States of ER DNA-Binding at 1-nt Variant EREs in the Genome (3,2,0,0,0) [157] 129
Table S10. Quantify the 3 Discrete States of KR DNA-Binding at 1-nt Variant HREs in the Genome (4,1,0,0,0) [194] 129
Table S11. (S/N) analysis of 1-nt Variant EREs in ER ChIPSeq Peaks (Variant Position) [157] 129
Table S12. (S/N) analysis of 2-nt Variant EREs in ER ChIPSeq Peaks (Variant Position) [157] 129
Table S13. (S/N) analysis of 3-nt Variant EREs in ER ChIPSeq Peaks (Variant Position) [157] 130
Table S14. (S/N) analysis of 4-nt Variant EREs in ER ChIPSeq Peaks (Variant Position) [157] 130
Table S15. (S/N) analysis of 5-nt Variant EREs in ER ChIPSeq Peaks (Variant Position) [157] 130
Table S16. (S/N) analysis of 1-nt Variant HREs in KR ChIPSeq Peaks (Variant Position) [194] 130
Table S17. (S/N) analysis of 2-nt Variant HREs in KR ChIPSeq Peaks (Variant Position) [194] 130
Table S18. (S/N) analysis of 3-nt Variant HREs in KR ChIPSeq Peaks (Variant Position) [194] 131
Table S19. (S/N) analysis of 4-nt Variant HREs in KR ChIPSeq Peaks (Variant Position) [194] 131
Table S20. (S/N) analysis of 5-nt Variant HREs in KR ChIPSeq Peaks (Variant Position) [194] 131

Table S21-S40. 15-nt ERE and HRE DNA Element Analysis 132
Table S21. Categorize 0-nt to 6-nt Variant 15-nt EREs into 924 Half-Site Groups (Variant Position) 132
Table S22. Categorize 0-nt to 6-nt Variant 15-nt HREs into 924 Half-Site Groups (Variant Position) 132
Table S23. Categorize 0-nt to 6-nt Variant 15-nt EREs into 924 Half-Site Groups (Sequence) 132
Table S24. Categorize 0-nt to 6-nt Variant 15-nt HREs into 924 Half-Site Groups (Sequence) 132
Table S25. X-Axis Order of 924 ERE Half-Site Groups 132
Table S26. X-Axis Order of 924 HRE Half-Site Groups 132
Table S27. (S/N) analysis of 0-nt to 6-nt Variant EREs in ER ChIPSeq Peaks (924 Half-Site Groups) [157] 133
Table S28. (S/N) analysis of 0-nt to 6-nt Variant HREs in KR ChIPSeq Peaks (924 Half-Site Groups) [194] 133
Table S29. (S/N) analysis of 1-nt Variant EREs in ER ChIPSeq Peaks (Variant Position) [157] 133
Table S30. (S/N) analysis of 2-nt Variant EREs in ER ChIPSeq Peaks (Variant Position) [157] 133
Table S31. (S/N) analysis of 3-nt Variant EREs in ER ChIPSeq Peaks (Variant Position) [157] 133
Table S32. (S/N) analysis of 4-nt Variant EREs in ER ChIPSeq Peaks (Variant Position) [157] 133
Table S33. (S/N) analysis of 5-nt Variant EREs in ER ChIPSeq Peaks (Variant Position) [157] 134
Table S34. (S/N) analysis of 6-nt Variant EREs in ER ChIPSeq Peaks (Variant Position) [157] 134
Table S35. (S/N) analysis of 1-nt Variant HREs in KR ChIPSeq Peaks (Variant Position) [194] 134
Table S36. (S/N) analysis of 2-nt Variant HREs in KR ChIPSeq Peaks (Variant Position) [194] 134
Table S37. (S/N) analysis of 3-nt Variant HREs in KR ChIPSeq Peaks (Variant Position) [194] 135
Table S38. (S/N) analysis of 4-nt Variant HREs in KR ChIPSeq Peaks (Variant Position) [194] 135
Table S39. (S/N) analysis of 5-nt Variant HREs in KR ChIPSeq Peaks (Variant Position) [194] 135
Table S40. (S/N) analysis of 6-nt Variant HREs in KR ChIPSeq Peaks (Variant Position) [194] 135

Table S41-S46. Transform the 13-nt DNA Element Analysis to the 15-nt DNA Element Analysis 136
Table S41. Transform the 13-nt EREs to the 15-nt EREs (Variant Position) 136

Table S42. Transform the 13-nt HREs to the 15-nt HREs (Variant Position) ... 136
Table S43. Transform the 13-nt EREs to the 15-nt EREs (Sequence) ... 136
Table S44. Transform the 13-nt HREs to the 15-nt HREs (Sequence) ... 137
Table S45. Transform the 15-nt EREs to the 13-nt EREs ... 137
Table S46. Transform the 15-nt HREs to the 13-nt HREs ... 137

Table S47-S50. Inversion Symmetry of the Single-Stranded Mouse and Human Genome ... 138
Table S47. Population Count of 1-nt to 20-nt DNA Elements in the Single-Stranded Mouse Genome [1.4 T] ... 138
Table S48. Population Count of 1-nt to 20-nt DNA Elements in the Single-Stranded Human Genome [1.4 T] ... 138
Table S49. Population Count of 1-nt to 20-nt DNA Elements in the Single-Stranded Mouse Genome (Chromosome) ... 138
Table S50. Population Count of 1-nt to 20-nt DNA Elements in the Single-Stranded Human Genome (Chromosome) ... 138

Table S51-S54. p53 DNA Element Analysis ... 139
Table S51. Categorize 0-nt to 5-nt Variant 10-nt p53REs into 252 Half-Site Groups (Variant Position) ... 139
Table S52. Categorize 0-nt to 5-nt Variant 10-nt p53REs into 252 Half-Site Groups (Sequence) ... 139
Table S53. X-Axis Order of 252 p53RE Half-Site Groups ... 139
Table S54. (S/N) analysis of 0-nt to 5-nt Variant p53REs in p53 ChIPSeq Peaks (252 Half-Site Groups) [22] ... 139

Table S55-S63. Number of DNA Elements (ERE, HRE, p53RE) in the Mouse and Human Genome ... 140
Table S55. Number of 0-nt to 5-nt Variant 13-nt ERE DNA Elements in the Mouse Genome (81,922) ... 140
Table S56. Number of 0-nt to 5-nt Variant 13-nt ERE DNA Elements in the Human Genome (81,922) ... 140
Table S57. Number of 0-nt to 5-nt Variant 13-nt HRE DNA Elements in the Mouse Genome (81,922) ... 140
Table S58. Number of 0-nt to 5-nt Variant 13-nt HRE DNA Elements in the Human Genome (81,922) ... 140
Table S59. Number of 0-nt to 6-nt Variant 15-nt ERE DNA Elements in the Mouse Genome (912,718) ... 141
Table S60. Number of 0-nt to 6-nt Variant 15-nt ERE DNA Elements in the Human Genome (912,718) ... 141
Table S61. Number of 0-nt to 6-nt Variant 15-nt HRE DNA Elements in the Mouse Genome (912,718) ... 141
Table S62. Number of 0-nt to 6-nt Variant 15-nt HRE DNA Elements in the Human Genome (912,718) ... 141
Table S63. Number of 0-nt to 5-nt Variant 10-nt p53RE DNA Elements in the Mouse Genome (81,922) ... 141

Table S64-S71. Data Analysis Example (WT-E2-1hr.L4) ... 142
Table S64. Count 0-nt to 5-nt Variant 13-nt EREs in ER ChIPSeq Peaks (ChIPSeq Peak) ... 142
Table S65. Count 0-nt to 5-nt Variant 13-nt EREs in ER ChIPSeq Peaks (Variant Position) ... 142
Table S66. Categorize 0-nt to 5-nt Variant 13-nt EREs into 252 Half-Site Groups ... 142
Table S67. ER DNA-Binding at Non-NRFE Sites is Independent of NRFE-Containing ChIPSeq Peaks (13-nt ERE) ... 142
Table S68. Count 0-nt to 6-nt Variant 15-nt EREs in ER ChIPSeq Peaks (ChIPSeq Peak) ... 143
Table S69. Count 0-nt to 6-nt Variant 15-nt EREs in ER ChIPSeq Peaks (Variant Position) ... 143
Table S70. Categorize 0-nt to 6-nt Variant 15-nt EREs into 924 Half-Site Groups ... 143
Table S71. ER DNA-Binding at Non-NRFE Sites is Independent of NRFE-Containing ChIPSeq Peaks (15-nt ERE) ... 143

Table S72-S74. Genome Assembly of the Mouse and Human Genome ... 144

Table S72. Genome Assembly (Chromosome Size) of the Mouse and Human Genome 144
Table S73. Genome Assembly (Effective Size) of the Mouse and Human Genome 144
Table S74. Genome Assembly (N Islands) of the Mouse and Human Genome........ 144

Table S75. Script Codes for All Data Analyses........ 145

Datafile S1-S5 Descriptions: Compressed Datafiles........ 146
Datafile S1. ChIPSeq and ChIPExo Experiments 146
Datafile S2. Population Count of 1-nt to 20-nt DNA Elements in the Single-Stranded Mouse Genome [1.4 T]........ 146
Datafile S3. Population Count of 1-nt to 20-nt DNA Elements in the Single-Stranded Human Genome [1.4 T]........ 146
Datafile S4. Population Count of 1-nt to 20-nt DNA Elements in the Single-Stranded Mouse Genome (Chromosome)........ 146
Datafile S5. Population Count of 1-nt to 20-nt DNA Elements in the Single-Stranded Human Genome (Chromosome)........ 146

Transparent Methods 147
Quality Control and Peak Selection Criteria........ 147
Number and Location Coordinates of DNA Elements (ERE, HRE, p53RE) in the Mouse and Human Genome........ 148
Number and Location Coordinates of 0-nt to 5-nt Variant 13-nt ERE and HRE DNA Elements in the Genome 148
Number and Location Coordinates of 0-nt to 6-nt Variant 15-nt ERE and HRE DNA Elements in the Genome 149
Number and Location Coordinates of 0-nt to 5-nt Variant 10-nt p53RE DNA Elements in the Genome 150
Overlap DNA Elements (ERE, HRE, p53RE) in the Genome with ChIPSeq and ChIPExo Experiments........ 151
Convert 0-nt to 5-nt/6-nt Variant DNA Elements to Variant Positions 152
Convert 0-nt to 5-nt Variant 13-nt ERE and HRE DNA Elements to Variant Positions........ 152
Convert 0-nt to 6-nt Variant 15-nt ERE and HRE DNA Elements to Variant Positions........ 153
Convert 0-nt to 5-nt Variant 10-nt p53RE DNA Elements to Variant Positions........ 154
Categorize 0-nt to 5-nt/6-nt Variant DNA Elements into Half-Site Groups........ 155
Categorize 0-nt to 5-nt Variant 13-nt ERE and HRE DNA Elements Into 252 Half-Site Groups 155
Categorize 0-nt to 6-nt Variant 15-nt ERE and HRE DNA Elements Into 924 Half-Site Groups 156
Categorize 0-nt to 5-nt Variant 10-nt p53RE DNA Elements Into 252 Half-Site Groups 157
Signal-To-Noise (S/N) Analysis........ 158
sNR DNA-Binding at 0-nt to 5-nt Variant 13-nt ERE and HRE DNA Elements in the Genome 158
sNR DNA-Binding at 0-nt to 6-nt Variant 15-nt ERE and HRE DNA Elements in the Genome 160
p53 DNA-Binding at 0-nt to 5-nt Variant 10-nt p53RE DNA Elements in the Genome 162
Data Analysis Example (WT-E2-1hr.L4)........ 164
ER DNA-Binding at 0-nt to 5-nt Variant 13-nt ERE DNA Elements in the Genome........ 164
ER DNA-Binding at 0-nt to 6-nt Variant 15-nt ERE DNA Elements in the Genome........ 165
Mouse (mm10) and Human (hg19) Genome Assembly 166
Population Count of 1-nt to 20-nt DNA Elements in the Single-Stranded Mouse and Human Genome 167
Position Weight Matrices (PWMs) for DNA Motif Identification........ 168

Supplemental References........ 169

Figure S1-S25 Descriptions: 13-nt ERE and HRE DNA Element Analysis

****All detailed data and statistics associated with every figure are compiled in Data S1****

The analyses in this section were completed by overlapping the location coordinates of each 0-nt to 5-nt variant 13-nt ERE or HRE DNA element in the genome and the location coordinates of the ChIPSeq or ChIPExo peaks in an experiment. The 0-nt variant consensus palindromic 13-nt ERE DNA element (5′-GGTCAnnnTGACC-3′) and 0-nt variant consensus palindromic 13-nt HRE DNA element (5′-GAACAnnnTGTTC-3′) have ten (10) primary positions. The 0-nt to 5-nt variant ERE or HRE DNA elements include the 1 0-nt variant consensus palindromic DNA element, 30 1-nt variant DNA elements (10 variant positions), 405 2-nt variant DNA elements (45 variant positions), 3,240 3-nt variant DNA elements (120 variant positions), 17,010 4-nt variant DNA elements (210 variant positions), and 61,236 5-nt variant DNA elements (252 variant positions), for a total of 81,922 DNA elements.

Figure S1. Inversion Symmetry of sNR DNA-Binding at 2-nt Variant EREs and HREs in the Genome (%)
(A) Average distribution of 2-nt variant EREs in ER experiments (156 ERα and 1 ERβ) at the four hundred and five (405) 2-nt variant EREs in the genome (the total number of 2-nt variant EREs contained within a ChIPSeq experiment =100%) **(B)** Average distribution of 2-nt variant HREs in 193 KR experiments (75 AR, 64 GR, 54 PR) at the four hundred and five (405) 2-nt variant HREs in the genome (the total number of 2-nt variant HREs contained within a ChIPSeq experiment =100%). These four hundred and five (405) 2-nt variant EREs or HREs are defined by forty-five (45) variant positions. Five (5) of those are when the variants are in the palindromic position pairs (PP): 1-10, 2-9, 3-8, 4-7, 5-6. The remaining forty (40) are symmetrically split into 20 groups and their reverse-complements.

*****

Figure S2. (S/N) analysis of 0-nt to 5-nt Variant EREs in ER ChIPSeq Peaks (displayed by number of variants)
(S/N) analysis of ER DNA-binding at 0-nt to 5-nt variant EREs in 157 ER experiments (156 ERα and 1 ERβ). Experiments are ordered by the experiment with highest (S/N) value at the 0-nt variant consensus palindromic ERE DNA element in the genome. The signal-to-noise ratio (S/N) is the absolute number of times a 0-nt to 5-nt variant DNA element occurs within an experiment [defined by the total number of peaks in the experiment and the peak length] compared to the random frequency expectation of that DNA element occurring in the genome (i.e., the probability that any 10-nt DNA element that has a maximum possibility of 4 nucleotides at each of the 10 primary positions will occur is once every 1,048,576 nucleotides (4^{10}) at random occurrence). Thus, the expected noise is calculated by multiplying the total number of peaks in the experiment by the length of the peaks over 4^{10}. For (S/N) analysis of 0-nt to 5-nt variant DNA elements (displayed by the number of variants in the DNA element), the expected noise is multiplied by the number of DNA elements in each group: 1 0-nt variant consensus palindromic DNA element, 30 1-nt variant DNA elements, 405 2-nt variant DNA elements, 3,240 3-nt variant DNA elements, 17,010 4-nt variant DNA elements, and 61,236 5-nt variant DNA elements, for a total of 81,922 0-nt to 5-nt variant DNA elements. **Note**: the relative enhancement values of the sNR DNA-binding signals are scale invariant (i.e., the relative ratios between the 0-nt to 5-nt variant groups are constant). Y-axis =the natural log of the (S/N) value (i.e., random =0 and 0 =log(1)).

Figure S3. (S/N) analysis of 0-nt to 5-nt Variant HREs in KR ChIPSeq Peaks (displayed by number of variants)
(S/N) analysis of KR DNA-binding at 0-nt to 5-nt variant HREs in 194 KR experiments (75 AR, 64 GR, 1 MR, 54 PR). Experiments are grouped by KR, and then ordered by the experiment with highest (S/N) value at the 0-nt variant consensus palindromic HRE DNA element in the genome. The signal-to-noise ratio (S/N) is the absolute number of times a 0-nt to 5-nt variant DNA element occurs within an experiment [defined by the total number of peaks in the experiment and the peak length] compared to the random frequency expectation of that DNA element occurring in the genome (i.e., the probability that any 10-nt DNA element that has a maximum possibility of 4 nucleotides at each of the 10 primary positions will occur is once every 1,048,576 nucleotides (4^{10}) at random occurrence). Thus, the expected noise is calculated by multiplying the total number of peaks in the experiment by the length of the peaks over 4^{10}. For (S/N) analysis of 0-nt to 5-nt variant DNA elements (displayed by the number of variants in the DNA element), the expected noise is multiplied by the number of DNA elements in each group: 1 0-nt variant consensus palindromic DNA element, 30 1-nt variant DNA elements, 405 2-nt variant DNA elements, 3,240 3-nt variant DNA elements, 17,010 4-nt variant DNA elements, and 61,236 5-nt variant DNA elements, for a total of 81,922 0-nt to 5-nt variant DNA elements. **Note**: the relative enhancement values of the sNR DNA-binding signals are scale invariant (i.e., the relative ratios between the 0-nt to 5-nt variant groups are constant). Y-axis =the natural log of the (S/N) value (i.e., random =0 and 0 =log(1)).

*****

Figure S4. (S/N) analysis of 0-nt to 5-nt Variant HREs in GR ChIPSeq Peaks (displayed by 252 half-site groups)
(S/N) analysis of 0-nt to 5-nt variant HREs (displayed by the 252 half-site groups) in GR (Liver-GR-WT-pred-6am-2) [23,742 peaks, 142-nt peak length] ChIPSeq peaks. X-axis order =Reverse-Complement Vacancy Position ID 2-9 > 5-6 > 1-10 > 4-7 > 3-8. See **Table S6** for x-axis details. This DNA-binding profile was observed in 194 KR experiments, representing a wide variety of mouse tissues and human cell lines, and across multiple peak selection criteria (L4-L20) **(Table S8)**.

Figure S5. (S/N) analysis of 0-nt to 5-nt Variant HREs in PR ChIPSeq Peaks (displayed by 252 half-site groups)

(S/N) analysis of 0-nt to 5-nt variant HREs (displayed by the 252 half-site groups) in PR (Uterus-PGR-P4) [66,545 peaks, 154-nt peak length] ChIPSeq peaks. X-axis order =Reverse-Complement Vacancy Position ID 2-9 > 5-6 > 1-10 > 4-7 > 3-8. See **Table S6** for x-axis details. This DNA-binding profile was observed in 194 KR experiments, representing a wide variety of mouse tissues and human cell lines, and across multiple peak selection criteria (L4-L20) **(Table S8)**.

*****

Figure S6. Quantify the 5 Discrete States of ER DNA-Binding at 1-nt Variant EREs in the Genome (3,2,0,0,0)
(S/N) analysis of 1-nt variant EREs (displayed by the 252 half-site groups) in ER (WT-E2-1hr) [76,163 peaks, 146-nt peak length] ChIPSeq peaks produce five (5) discrete binding signals: 6 groups reach the (+2) plateau, 60 groups reach the (+1) plateau, 120 groups reach the (0) plateau, 60 groups reach the (-1) plateau, 6 groups reach the (-2) plateau. Thus, ER DNA-binding is greatest at the (+2) plateau, which are the 6 half-site groups that have two reverse-complement vacancies in palindromic position pair 3-8 and 1-10 and two reverse-complement double occupants in palindromic position pair (4-7 and 5-6), (2-9 and 5-6), or (2-9 and 4-7). ER DNA-binding is least at the (-2) plateau, which are the 6 half-site groups that have two reverse-complement vacancies in palindromic position pair (4-7 and 5-6), (2-9 and 5-6), or (2-9 and 4-7), and two reverse-complement double occupants in palindromic position pair 3-8 and 1-10. See **Table S5** for x-axis details. This DNA-binding profile was observed in 157 ER experiments, representing a wide variety of mouse tissues and human cell lines, and across multiple peak selection criteria (L4-L20) **(Table S9)**.

Figure S7. Quantify the 3 Discrete States of KR DNA-Binding at 1-nt Variant HREs in the Genome (4,1,0,0,0)
(S/N) analysis of 1-nt variant HREs (displayed by the 252 half-site groups) in AR (AR-wt1) [49,859 peaks, 136-nt peak length] ChIPSeq peaks produce three (3) discrete binding signals: 56 groups reach the (+1) plateau, 140 groups reach the (0) plateau, 56 groups reach the (-1) plateau. Thus, KR DNA-binding is greatest at the (+1) plateau, which are the 56 half-site groups that have a reverse-complement vacancy in palindromic position pair 2-9. KR DNA-binding is least at the (-1) plateau, which are the 56 half-site groups that have a reverse-complement double occupant in palindromic position pair 2-9. See **Table S6** for x-axis details. This DNA-binding profile was observed in 194 KR experiments, representing a wide variety of mouse tissues and human cell lines, and across multiple peak selection criteria (L4-L20) **(Table S10)**.

Figure S8. Algebraic Equations of the 3-State DNA Element (4,1,0,0,0)
The symbolic representations of the discrete states (+1, 0, -1) for the HRE can also be replaced with algebraic variables to formally define the methodology. The 3-state HRE is generated by splitting the nucleotides into a (4,1,0,0,0) grouping. That is, palindromic position pair 2-9 (symbolically represented by “A”) versus palindromic position pairs 5-6, 1-10, 4-7, 3-8 (symbolically represented by “B”). Therefore, if an A is replaced with a B, the impact is (–X) (i.e., the sNR DNA-binding affinity is decreased); while if a B is replaced with an A, the impact is the exactly the opposite (+X) (i.e., the sNR DNA-binding affinity is increased) to the same magnitude. Thus, this algebraic representation explains why sNR DNA-binding is quantitatively precise, and also why there are three (3) discrete DNA-binding states at 1-nt variant HRE DNA elements (by the KRs).

Figure S9. Algebraic Equations of the 5-State DNA Element (3,2,0,0,0)
The symbolic representations of the discrete states (+2, +1, 0, -1, -2) for the ERE can also be replaced with algebraic variables to formally define the methodology. The 5-state ERE is generated by splitting the nucleotides into a (3,2,0,0,0) grouping. That is, palindromic position pair 3-8 and 1-10 (symbolically represented by “A”) versus palindromic position pairs 5-6, 4-7, 2-9 (symbolically represented by “B”). Therefore, if an A is replaced with a B, the impact is (–X) (i.e., the sNR DNA-binding affinity is decreased); while if a B is replaced with an A, the impact is exactly the opposite (+X) (i.e., the sNR DNA-binding affinity is increased) to the same magnitude. Thus, this algebraic representation explains why sNR DNA-binding is quantitatively precise, and also why there are five (5) discrete DNA-binding states at 1-nt variant ERE DNA elements (by the ERs).

Figure S10. Algebraic Equations of the 9-State DNA Element (3,1,1,0,0)
The algebraic complexities increase substantially beyond the (4,1,0,0,0) HRE and (3,2,0,0,0) ERE representations. The (3,1,1,0,0) grouping (symbolically represented by “A, B, C”) creates 9 discrete states.

Figure S11. Algebraic Equations of the 11-State, 25-State, 51-State DNA Elements (2,2,1,0,0) (2,1,1,1,0) (1,1,1,1,1)
The algebraic complexities increase substantially beyond the (4,1,0,0,0) HRE and (3,2,0,0,0) ERE representations. The (2,2,1,0,0) grouping (symbolically represented by “A, B, C”) creates 11 discrete states, the (2,1,1,1,1) grouping (symbolically represented by “A, B, C, D”) creates 25 discrete states, and the (1,1,1,1,1) grouping (symbolically represented by “A, B, C, D, E”) creates 51 discrete states; which are all of the remaining possibilities for a 5-nt DNA element in the genome (i.e., a 5-nt element, followed by any arbitrary spacer, followed by its 5-nt reverse-complement element).

*****

Figure S12. (S/N) analysis of 1-nt Variant EREs in ER ChIPSeq Peaks (displayed by variant position)
(S/N) analysis of 1-nt variant EREs in ER (WT-E2-1hr) ChIPSeq peaks [76,163 peaks, 146-nt peak length].
Displayed by the 10 variant positions of the 30 1-nt variant EREs. This DNA-binding profile was observed in 157 ER experiments, representing a wide variety of mouse tissues and human cell lines, and across multiple peak selection criteria (L4-L20) **(Table S11)**.

Figure S13. (S/N) analysis of 2-nt Variant EREs in ER ChIPSeq Peaks (displayed by variant position)
(S/N) analysis of 2-nt variant EREs in ER (WT-E2-1hr) ChIPSeq peaks [76,163 peaks, 146-nt peak length].
Displayed by the 45 variant positions of the 405 2-nt variant EREs. 5 of the 45 variant positions are the five (5) palindromic position pairs: 1-10, 2-9, 3-8, 4-7, 5-6 (PP, dark red solid bars). 20 of the 45 variant positions are same-side variants (i.e., the variants do not crossover the 3-nt spacer) (blue solid bars). 20 of the 45 variant positions are crossover variants (i.e., the variants do crossover the 3-nt spacer) (blue diagonal stripped bars). Each variant position is immediately followed by its reverse-complement variant position on the x-axis, demonstrating that transcription factor binding in the genome follows inversion symmetry (i.e., the number of transcription factor binding events at a particular DNA element in the genome is equivalent to the number of transcription factor binding events at its reverse-complement DNA element in the genome). This DNA-binding profile was observed in 157 ER experiments, representing a wide variety of mouse tissues and human cell lines, and across multiple peak selection criteria (L4-L20) **(Table S12)**.

Figure S14. (S/N) analysis of 3-nt Variant EREs in ER ChIPSeq Peaks (displayed by variant position)
(S/N) analysis of 3-nt variant EREs in ER (WT-E2-1hr) ChIPSeq peaks [76,163 peaks, 146-nt peak length].
Displayed by the 120 variant positions of the 3,240 3-nt variant EREs. 20 of the 120 variant positions are same-side variants (i.e., the variants do not crossover the 3-nt spacer) (blue solid bars). 100 of the 120 variant positions are crossover variants (i.e., the variants do crossover the 3-nt spacer) (blue diagonal stripped bars). Each variant position is immediately followed by its reverse-complement variant position on the x-axis, demonstrating that transcription factor binding in the genome follows inversion symmetry (i.e., the number of transcription factor binding events at a particular DNA element in the genome is equivalent to the number of transcription factor binding events at its reverse-complement DNA element in the genome). This DNA-binding profile was observed in 157 ER experiments, representing a wide variety of mouse tissues and human cell lines, and across multiple peak selection criteria (L4-L20) **(Table S13)**.

Figure S15. (S/N) analysis of 4-nt Variant EREs in ER ChIPSeq Peaks (displayed by variant position)
(S/N) analysis of 4-nt variant EREs in ER (WT-E2-1hr) ChIPSeq peaks [76,163 peaks, 146-nt peak length].
Displayed by the 210 variant positions of the 17,010 4-nt variant EREs. 10 of the 210 variant positions are the five (5) palindromic position pairs: 1-3-8-10, 3-5-6-8, 3-4-7-8, 2-3-8-9, 1-5-6-10, 1-4-7-10, 1-2-9-10, 4-5-6-7, 2-5-6-9, 2-4-7-9 (PP, dark red solid bars). 10 of the 210 variant positions are same-side variants (i.e., the variants do not crossover the 3-nt spacer) (blue solid bars). 190 of the 45 variant positions are crossover variants (i.e., the variants do crossover the 3-nt spacer) (blue diagonal stripped bars). Each variant position is immediately followed by its reverse-complement variant position on the x-axis, demonstrating that transcription factor binding in the genome follows inversion symmetry (i.e., the number of transcription factor binding events at a particular DNA element in the genome is equivalent to the number of transcription factor binding events at its reverse-complement DNA element in the genome). This DNA-binding profile was observed in 157 ER experiments, representing a wide variety of mouse tissues and human cell lines, and across multiple peak selection criteria (L4-L20) **(Table S14)**.

Figure S16. (S/N) analysis of 5-nt Variant EREs in ER ChIPSeq Peaks (displayed by variant position)
(S/N) analysis of 5-nt variant EREs in ER (WT-E2-1hr) ChIPSeq peaks [76,163 peaks, 146-nt peak length].
Displayed by the 252 variant positions of the 61,236 5-nt variant EREs. 2 of the 252 variant positions are same-side variants (i.e., the variants do not crossover the 3-nt spacer) (blue solid bars). 250 of the 252 variant positions are crossover variants (i.e., the variants do crossover the 3-nt spacer) (blue diagonal stripped bars). Each variant position is immediately followed by its reverse-complement variant position on the x-axis, demonstrating that transcription factor binding in the genome follows inversion symmetry (i.e., the number of transcription factor binding events at a particular DNA element in the genome is equivalent to the number of transcription factor binding events at its reverse-complement DNA element in the genome). This DNA-binding profile was observed in 157 ER experiments, representing a wide variety of mouse tissues and human cell lines, and across multiple peak selection criteria (L4-L20) **(Table S15)**.

*****

Figure S17. (S/N) analysis of 1-nt Variant HREs in KR ChIPSeq Peaks (displayed by variant position)
(S/N) analysis of 1-nt variant HREs in AR (AR-wt1) ChIPSeq peaks [49,859 peaks, 136-nt peak length].
Displayed by the 10 variant positions of the 30 1-nt variant HREs. This DNA-binding profile was observed in 194 KR experiments, representing a wide variety of mouse tissues and human cell lines, and across multiple peak selection criteria (L4-L20) **(Table S16)**.

Figure S18. (S/N) analysis of 2-nt Variant HREs in KR ChIPSeq Peaks (displayed by variant position)
(S/N) analysis of 2-nt variant HREs in AR (AR-wt1) ChIPSeq peaks [49,859 peaks, 136-nt peak length].
Displayed by the 45 variant positions of the 405 2-nt variant HREs. 5 of the 45 variant positions are the five (5) palindromic position pairs: 1-10, 2-9, 3-8, 4-7, 5-6 (PP, dark red solid bars). 20 of the 45 variant positions are same-side variants (i.e., the variants do not crossover the 3-nt spacer) (orange solid bars). 20 of the 45 variant positions are crossover variants (i.e., the variants do crossover the 3-nt spacer) (orange diagonal stripped bars). Each variant position is immediately followed by its reverse-complement variant position on the x-axis, demonstrating that transcription factor binding in the genome follows inversion symmetry (i.e., the number of transcription factor binding events at a particular DNA element in the genome is equivalent to the number of transcription factor binding events at its reverse-complement DNA element in the genome). This DNA-binding profile was observed in 194 KR

experiments, representing a wide variety of mouse tissues and human cell lines, and across multiple peak selection criteria (L4-L20) **(Table S17)**.

Figure S19. (S/N) analysis of 3-nt Variant HREs in KR ChIPSeq Peaks (displayed by variant position)
(S/N) analysis of 3-nt variant HREs in AR (AR-wt1) ChIPSeq peaks [49,859 peaks, 136-nt peak length].
Displayed by the 120 variant positions of the 3,240 3-nt variant HREs. 20 of the 120 variant positions are same-side variants (i.e., the variants do not crossover the 3-nt spacer) (orange solid bars). 100 of the 120 variant positions are crossover variants (i.e., the variants do crossover the 3-nt spacer) (orange diagonal stripped bars). Each variant position is immediately followed by its reverse-complement variant position on the x-axis, demonstrating that transcription factor binding in the genome follows inversion symmetry (i.e., the number of transcription factor binding events at a particular DNA element in the genome is equivalent to the number of transcription factor binding events at its reverse-complement DNA element in the genome). This DNA-binding profile was observed in 194 KR experiments, representing a wide variety of mouse tissues and human cell lines, and across multiple peak selection criteria (L4-L20) **(Table S18)**.

Figure S20. (S/N) analysis of 4-nt Variant HREs in KR ChIPSeq Peaks (displayed by variant position)
(S/N) analysis of 4-nt variant HREs in AR (AR-wt1) ChIPSeq peaks [49,859 peaks, 136-nt peak length].
Displayed by the 210 variant positions of the 17,010 4-nt variant HREs. 10 of the 210 variant positions are the five (5) palindromic position pairs: 2-5-6-9, 1-2-9-10, 2-4-7-9, 2-3-8-9, 1-5-6-10, 4-5-6-7, 3-5-6-8, 1-4-7-10, 1-3-8-10, 3-4-7-8 (PP, dark red solid bars). 10 of the 210 variant positions are same-side variants (i.e., the variants do not crossover the 3-nt spacer) (orange solid bars). 190 of the 45 variant positions are crossover variants (i.e., the variants do crossover the 3-nt spacer) (orange diagonal stripped bars). Each variant position is immediately followed by its reverse-complement variant position on the x-axis, demonstrating that transcription factor binding in the genome follows inversion symmetry (i.e., the number of transcription factor binding events at a particular DNA element in the genome is equivalent to the number of transcription factor binding events at its reverse-complement DNA element in the genome). This DNA-binding profile was observed in 194 KR experiments, representing a wide variety of mouse tissues and human cell lines, and across multiple peak selection criteria (L4-L20) **(Table S19)**.

Figure S21. (S/N) analysis of 5-nt Variant HREs in KR ChIPSeq Peaks (displayed by variant position)
(S/N) analysis of 5-nt variant HREs in AR (AR-wt1) ChIPSeq peaks [49,859 peaks, 136-nt peak length].
Displayed by the 252 variant positions of the 61,236 5-nt variant HREs. 2 of the 252 variant positions are same-side variants (i.e., the variants do not crossover the 3-nt spacer) (orange solid bars). 250 of the 252 variant positions are crossover variants (i.e., the variants do crossover the 3-nt spacer) (orange diagonal stripped bars). Each variant position is immediately followed by its reverse-complement variant position on the x-axis, demonstrating that transcription factor binding in the genome follows inversion symmetry (i.e., the number of transcription factor binding events at a particular DNA element in the genome is equivalent to the number of transcription factor binding events at its reverse-complement DNA element in the genome). This DNA-binding profile was observed in 194 KR experiments, representing a wide variety of mouse tissues and human cell lines, and across multiple peak selection criteria (L4-L20) **(Table S20)**.

*****

Here we show replicate experiments for GR and GR-Dim to illustrate that the slight decrease in DNA-binding at a few specific 3-nt variant HRE DNA elements by GR-Dim is made apparent by the DNA-binding enhancement of a particular experiment at 0-nt to 2-nt variant HRE DNA elements as the relative (S/N) values of sNR DNA-binding signals are scale invariant (i.e., the relative ratios between the 0-nt to 5-nt variant groups are constant) **(Figure S2-S3)**. Thus, the reduced amplitude in DNA-binding at a few specific 3-nt variant HRE DNA elements only occurs in experiments with the highest (S/N) values at 0-nt to 2-nt variant HRE DNA elements.

Figure S22. (S/N) analysis of 0-nt to 5-nt Variant HREs in GR and GR-Dim ChIPSeq Peaks #2 (displayed by 252 half-site groups)
(S/N) analysis of 0-nt to 5-nt variant HREs (displayed by the 252 half-site groups) in GR (Liver-GR-WT-pred-6am-1) [34,758 peaks, 151-nt peak length] ChIPSeq peaks and GR-Dim (Liver-GR-Dim-pred-6am-1) [22,130 peaks, 163-nt peak length] ChIPSeq peaks. X-axis order =Reverse-Complement Vacancy Position ID 2-9 > 5-6 > 1-10 > 4-7 > 3-8. See **Table S6** for x-axis details.

Figure S23. (S/N) analysis of 0-nt to 5-nt Variant HREs in GR and GR-Dim ChIPExo Peaks #1 (displayed by 252 half-site groups)
(S/N) analysis of 0-nt to 5-nt variant HREs (displayed by the 252 half-site groups) in GR (Liver-GR-WT-pred-6am-1) [20,966 peaks, 445-nt peak length] ChIPExo peaks and GR-Dim (Liver-GR-Dim-pred-6am-1) [19,480 peaks, 391-nt peak length] ChIPExo peaks. X-axis order =Reverse-Complement Vacancy Position ID 2-9 > 5-6 > 1-10 > 4-7 > 3-8. See **Table S6** for x-axis details.

Figure S24. (S/N) analysis of 0-nt to 5-nt Variant HREs in GR and GR-Dim ChIPExo Peaks #2 (displayed by 252 half-site groups)
(S/N) analysis of 0-nt to 5-nt variant HREs (displayed by the 252 half-site groups) in GR (Liver-GR-WT-pred-6am-2) [22,129 peaks, 338-nt peak length] ChIPExo peaks and GR-Dim (Liver-GR-Dim-pred-6am-2) [25,670 peaks, 408-nt peak length] ChIPExo peaks. X-axis order =Reverse-Complement Vacancy Position ID 2-9 > 5-6 > 1-10 > 4-7 > 3-8. See **Table S6** for x-axis details.

Figure S25. (S/N) analysis of 1-nt to 5-nt Variant HREs in GR and GR-Dim ChIPExo Peaks (displayed by variant position)
(S/N) analysis of 1-nt to 5-nt variant HREs (displayed by variant position) in GR (Liver-GR-WT-pred-6am-1) [20,966 peaks, 445-nt peak length] (Liver-GR-WT-pred-6am-2) [22,129 peaks, 338-nt peak length] ChIPExo peaks and GR-Dim (Liver-GR-Dim-pred-6am-1) [19,480 peaks, 391-nt peak length] (Liver-GR-Dim-pred-6am-2) [25,670 peaks, 408-nt peak length] ChIPExo peaks.

Figure S1. Inversion Symmetry of sNR DNA-Binding at 2-nt Variant EREs and HREs in the Genome (%)

(A) 2-nt Variant EREs in ER ChIPSeq Peaks (157 Experiments)

ERE = GGTCAnnnTGACC

1 2 3 4 5 6 7 8 9 10

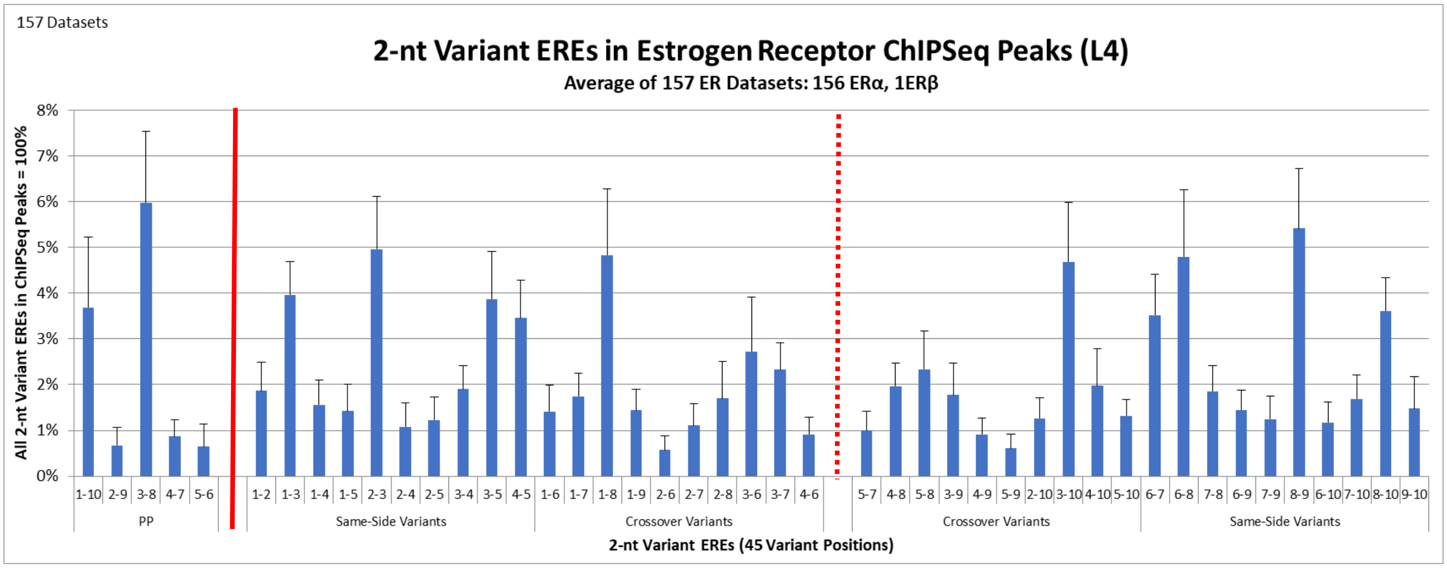

---- Symmetry Divider

(B) 2-nt Variant HREs in KR ChIPSeq Peaks (193 Experiments)

HRE = GAACAnnnTGTTC

1 2 3 4 5 6 7 8 9 10

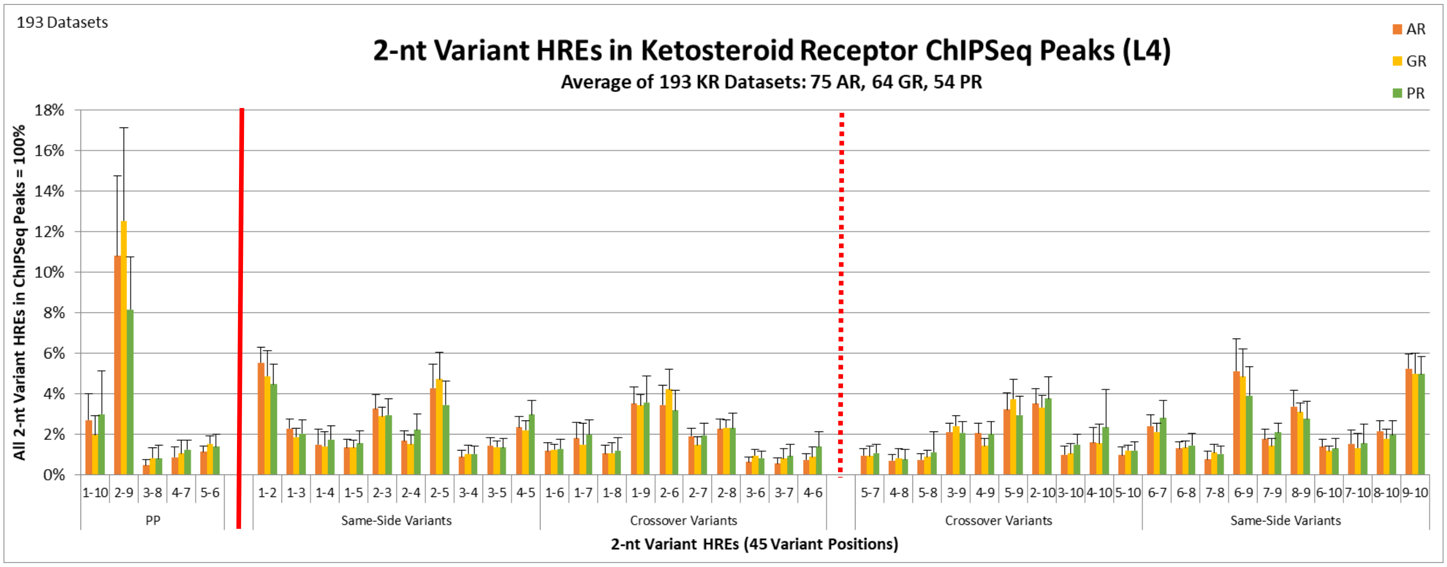

---- Symmetry Divider

Figure S2. (S/N) analysis of 0-nt to 5-nt Variant EREs in ER ChIPSeq Peaks (# of Variants)

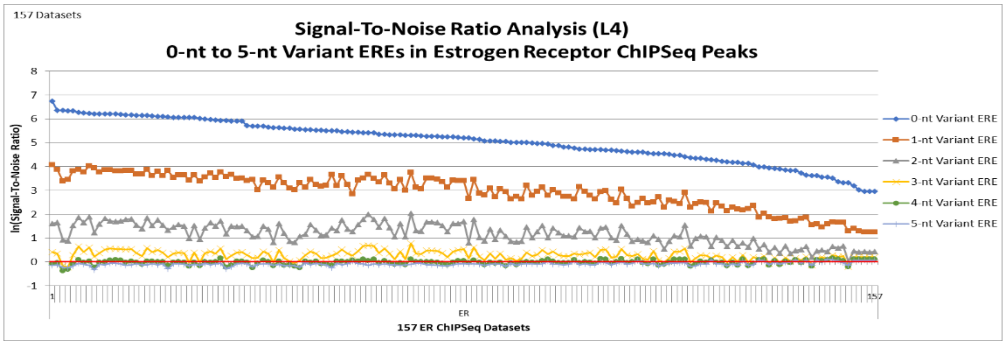

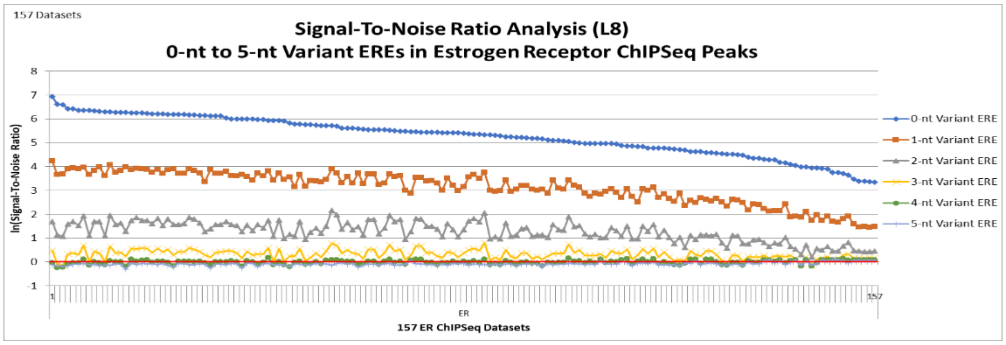

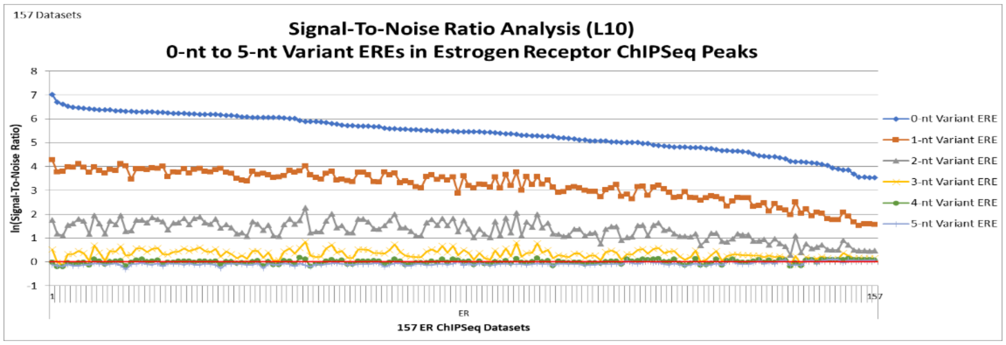

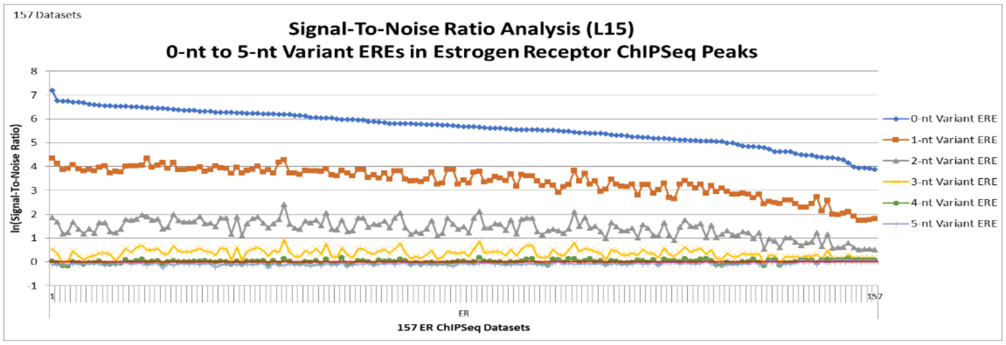

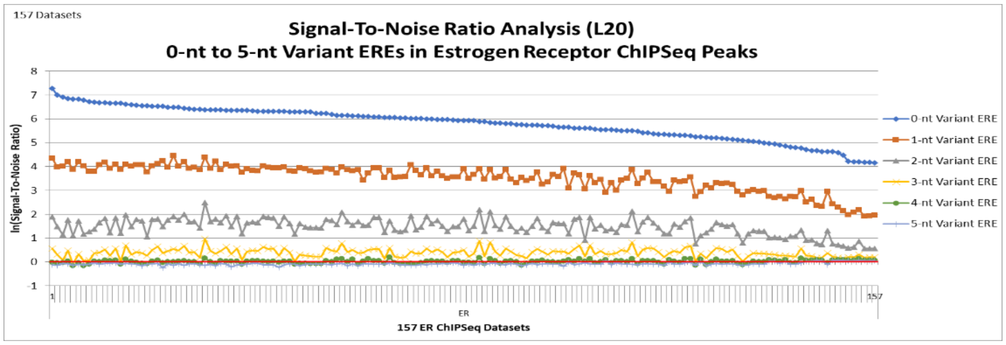

Figure S3. (S/N) analysis of 0-nt to 5-nt Variant HREs in KR ChIPSeq Peaks (# of Variants)

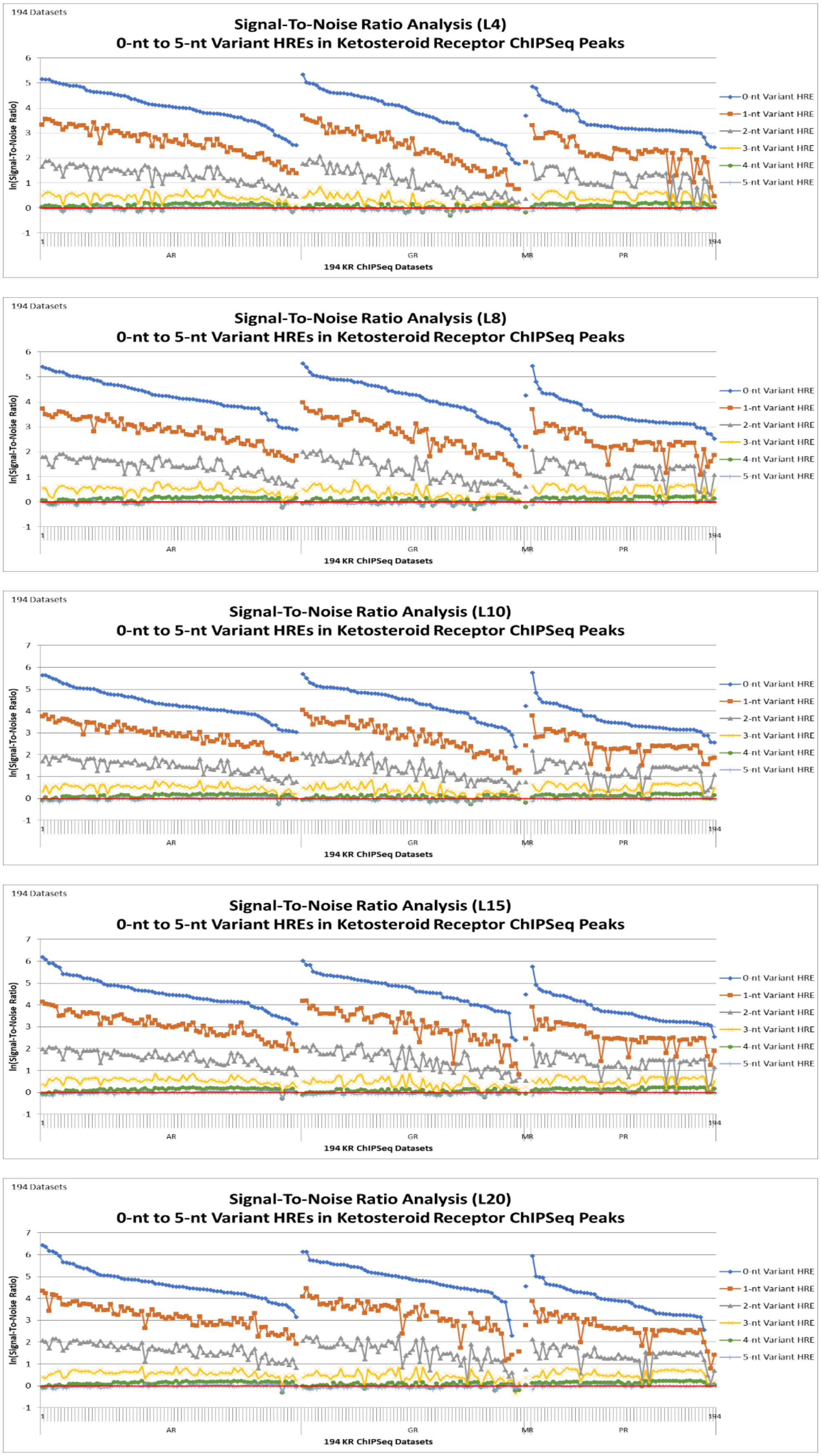

Figure S4. (S/N) analysis of 0-nt to 5-nt Variant HREs in GR ChIPSeq Peaks (252 Half-Site Groups)

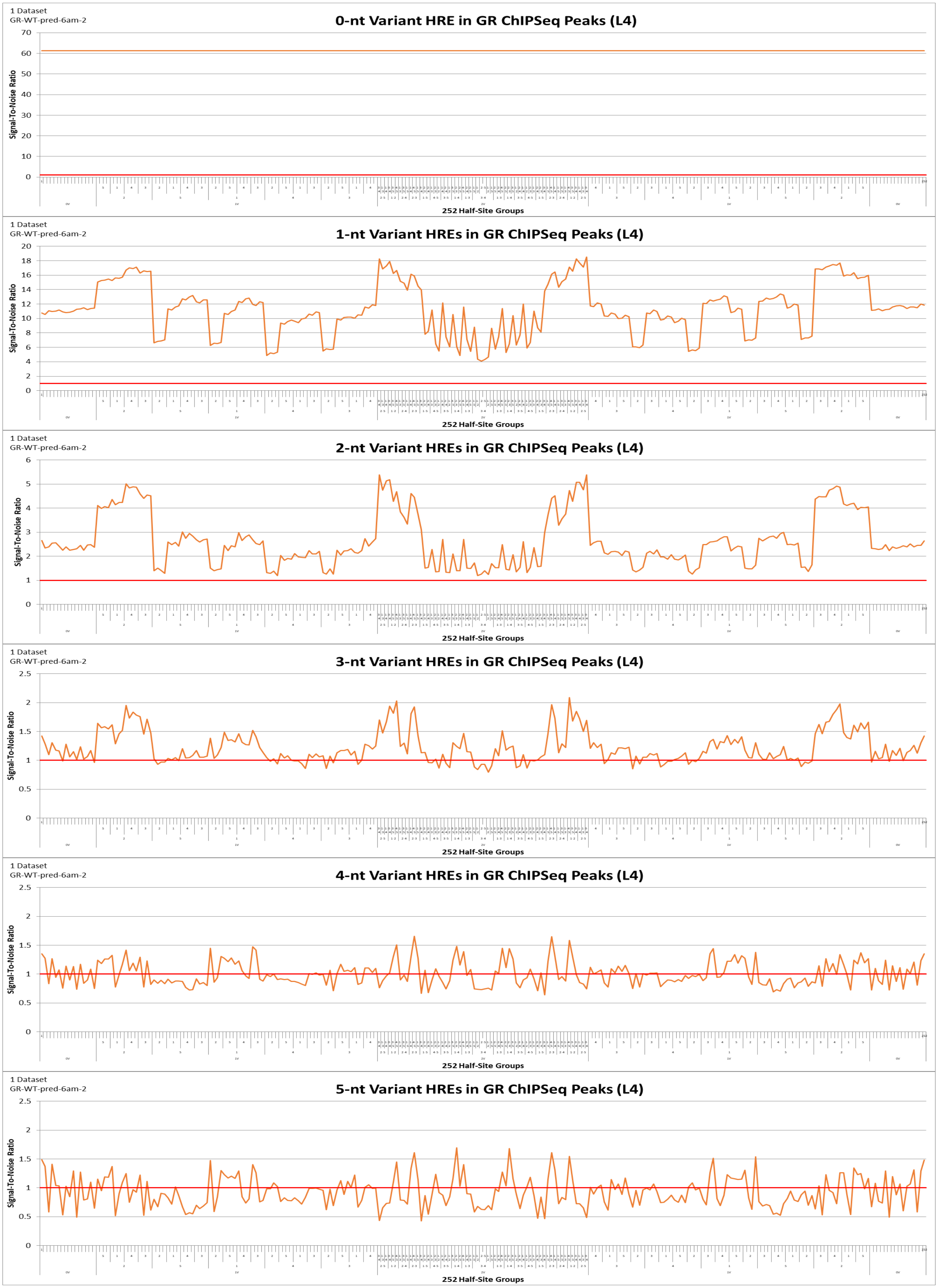

Figure S5. (S/N) analysis of 0-nt to 5-nt Variant HREs in PR ChIPSeq Peaks (252 Half-Site Groups)

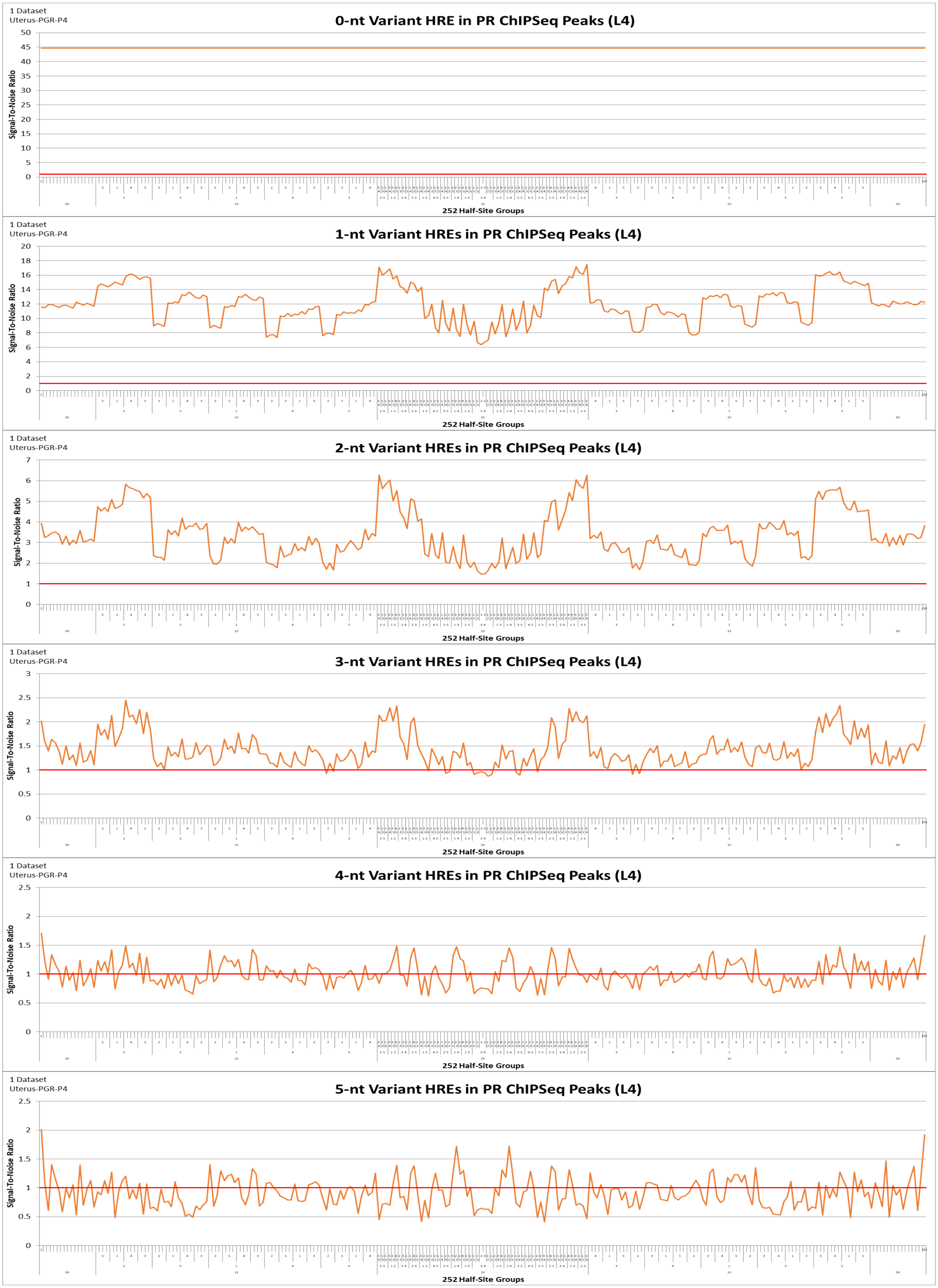

Figure S6. Quantify the 5 Discrete States of ER DNA-Binding at 1-nt Variant EREs in the Genome (3,2,0,0,0)

ERE = GGTCAnnnTGACC

1 2 3 4 5 6 7 8 9 10

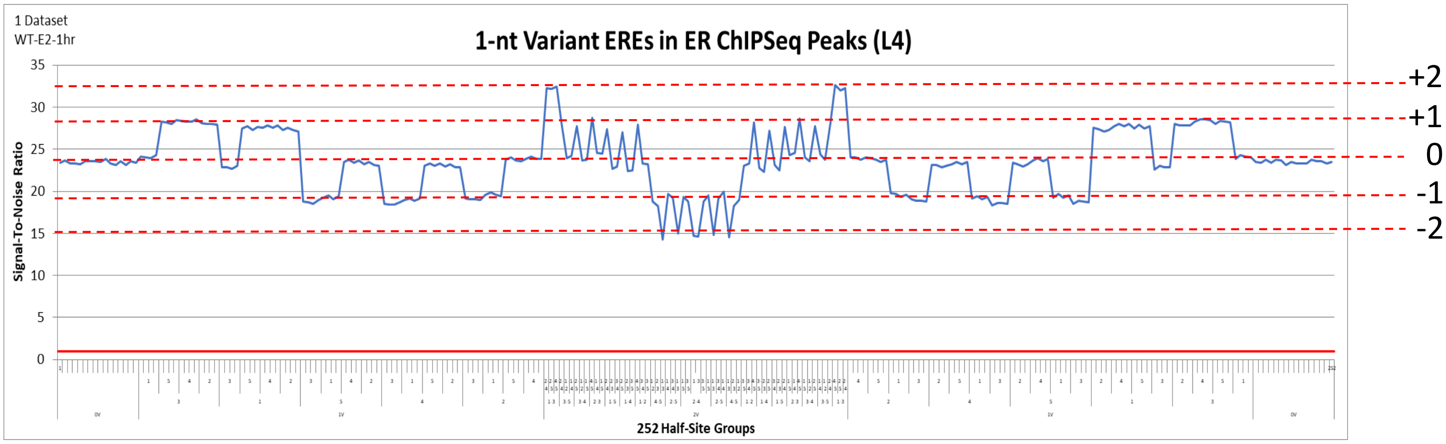

<u>Five (5) Discrete DNA-Binding Plateaus</u>

One Vacancy

RC Double Occupant Position ID	RC Vacancy Position ID					
	ERE	3-8	1-10	5-6	4-7	2-9
	3-8		0	-1	-1	-1
	1-10	0		-1	-1	-1
	5-6	+1	+1		0	0
	4-7	+1	+1	0		0
	2-9	+1	+1	0	0	

(S/N) Value	# of 252 Half-Site Groups
+2	6
+1	60
0	120
-1	60
-2	6

Two Vacancies

RC Double Occupant Position ID	RC Vacancy Position ID										
	ERE	(1,3)-(8,10)	(3,5)-(6,8)	(3,4)-(7,8)	(2,3)-(8,9)	(1,5)-(6,10)	(1,4),(7,10)	(1,2)-(9,10)	(4,5)-(6,7)	(2,5)-(6,9)	(2,4)-(7,9)
	(1,3)-(8,10)								-2	-2	-2
	(3,5)-(6,8)						0	0			-1
	(3,4)-(7,8)					0		0		-1	
	(2,3)-(8,9)					0	0		-1		
	(1,5)-(6,10)			0	0						-1
	(1,4),(7,10)		0		0					-1	
	(1,2)-(9,10)		0	0					-1		
	(4,5)-(6,7)	+2			+1			+1			
	(2,5)-(6,9)	+2		+1			+1				
	(2,4)-(7,9)	+2	+1			+1					

Figure S7. Quantify the 3 Discrete States of KR DNA-Binding at 1-nt Variant HREs in the Genome (4,1,0,0,0)

HRE = GAACAnnnTGTTC

1 2 3 4 5 6 7 8 9 10

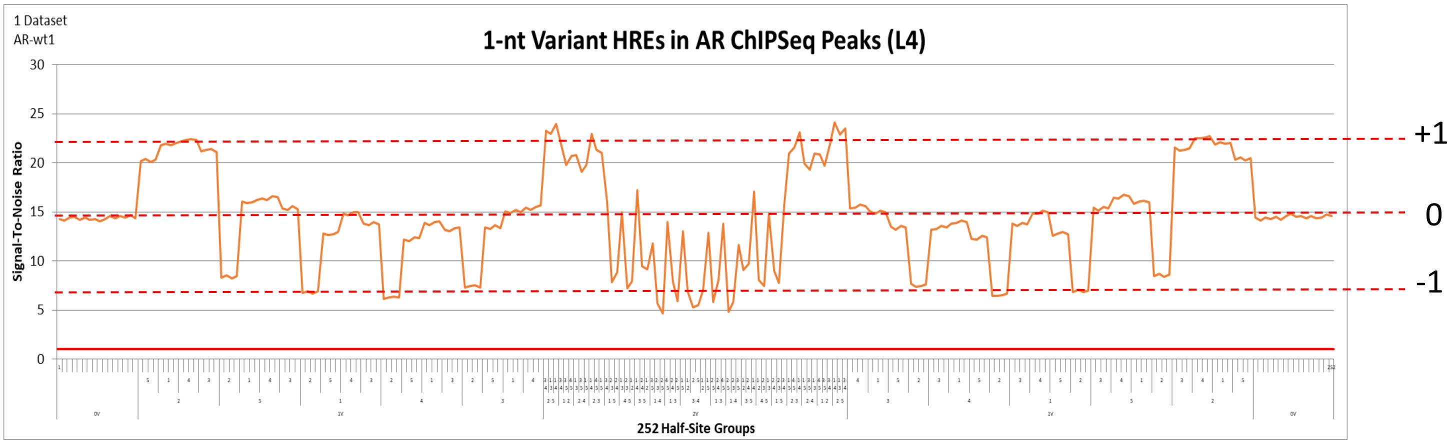

Three (3) Discrete DNA-Binding Plateaus:

One Vacancy

		RC Vacancy Position ID				
	HRE	2-9	5-6	1-10	4-7	3-8
RC Double Occupant Position ID	2-9		-1	-1	-1	-1
	5-6	+1		0	0	0
	1-10	+1	0		0	0
	4-7	+1	0	0		0
	3-8	+1	0	0	0	

(S/N) Value	# of 252 Half-Site Groups
+1	56
0	140
-1	56

Two Vacancies

		RC Vacancy Position ID									
	HRE	(2,5)-(6,9)	(1,2)-(9,10)	(2,4)-(7,9)	(2,3)-(8,9)	(1,5)-(6,10)	(4,5)-(6,7)	(3,5)-(6,8)	(1,4),(7,10)	(1,3)-(8,10)	(3,4)-(7,8)
RC Double Occupant Position ID	(2,5)-(6,9)								-1	-1	-1
	(1,2)-(9,10)						-1	-1			-1
	(2,4)-(7,9)					-1		-1		-1	
	(2,3)-(8,9)					-1	-1		-1		
	(1,5)-(6,10)			+1	+1						0
	(4,5)-(6,7)		+1		+1					0	
	(3,5)-(6,8)		+1	+1					0		
	(1,4),(7,10)	+1			+1			0			
	(1,3)-(8,10)	+1		+1			0				
	(3,4)-(7,8)	+1	+1			0					

Figure S8. Algebraic Equations of the 3-State DNA Element (4,1,0,0,0)

Hormone Response Element (HRE) = 3 Discrete States (4,1,0,0,0)

One Vacancy

			RC Vacancy Position ID				
			N_1	N_2	N_3	N_4	N_5
		Rel Charge	A	B	B	B	B
RC Double Occupant Position ID	N_1	A		-X	-X	-X	-X
	N_2	B	+X		0	0	0
	N_3	B	+X	0		0	0
	N_4	B	+X	0	0		0
	N_5	B	+X	0	0	0	

Two Vacancies

			RC Vacancy Position ID									
			N_1-N_2	N_1-N_3	N_1-N_4	N_1-N_5	N_2-N_3	N_2-N_4	N_2-N_5	N_3-N_4	N_3-N_5	N_4-N_5
		Rel Charge	AB	AB	AB	AB	BB	BB	BB	BB	BB	BB
RC Double Occupant Position ID	N_1-N_2	AB								-X	-X	-X
	N_1-N_3	AB						-X	-X			-X
	N_1-N_4	AB					-X		-X		-X	
	N_1-N_5	AB					-X	-X		-X		
	N_2-N_3	BB			+X	+X						0
	N_2-N_4	BB		+X		+X					0	
	N_2-N_5	BB		+X	+X					0		
	N_3-N_4	BB	+X			+X			0			
	N_3-N_5	BB	+X		+X			0				
	N_4-N_5	BB	+X	+X			0					

Algebraic Equations:

A – B = X

B – A = -X

Figure S9. Algebraic Equations of the 5-State DNA Element (3,2,0,0,0)

Estrogen Response Element (ERE) = 5 Discrete States (3,2,0,0,0)

One Vacancy

			RC Vacancy Position ID				
			N_1	N_2	N_3	N_4	N_5
		Rel Charge	A	A	B	B	B
RC Double Occupant Position ID	N_1	A		0	-X	-X	-X
	N_2	A	0		-X	-X	-X
	N_3	B	+X	+X		0	0
	N_4	B	+X	+X	0		0
	N_5	B	+X	+X	0	0	

Algebraic Equations:

A – B = X

B – A = -X

Two Vacancies

			RC Vacancy Position ID									
			N_1-N_2	N_1-N_3	N_1-N_4	N_1-N_5	N_2-N_3	N_2-N_4	N_2-N_5	N_3-N_4	N_3-N_5	N_4-N_5
		Rel Charge	AA	AB	AB	AB	AB	AB	AB	BB	BB	BB
RC Double Occupant Position ID	N_1-N_2	AA								-2X	-2X	-2X
	N_1-N_3	AB						0	0			-X
	N_1-N_4	AB					0		0		-X	
	N_1-N_5	AB					0	0		-X		
	N_2-N_3	AB			0	0						-X
	N_2-N_4	AB		0		0					-X	
	N_2-N_5	AB		0	0					-X		
	N_3-N_4	BB	+2X			+X			+X			
	N_3-N_5	BB	+2X		+X			+X				
	N_4-N_5	BB	+2X	+X			+X					

Algebraic Equations:

AA – BB = 2X

BB – AA = -2X

AB – BB = A – B = X

BB – AB = B – A = -X

Figure S10. Algebraic Equations of the 9-State DNA Element (3,1,1,0,0)

9 Discrete States (3,1,1,0,0)

One Vacancy

			RC Vacancy Position ID				
			N_1	N_2	N_3	N_4	N_5
		Rel Charge	A	B	C	C	C
RC Double Occupant Position ID	N_1	A		-X	-Z	-Z	-Z
	N_2	B	+X		-Y	-Y	-Y
	N_3	C	+Z	+Y		0	0
	N_4	C	+Z	+Y	0		0
	N_5	C	+Z	+Y	0	0	

Algebraic Equations:

A – B = X

B – C = Y

A – C = Z

Two Vacancies

			RC Vacancy Position ID									
			N_1-N_2	N_1-N_3	N_1-N_4	N_1-N_5	N_2-N_3	N_2-N_4	N_2-N_5	N_3-N_4	N_3-N_5	N_4-N_5
		Rel Charge	AB	AC	AC	AC	BC	BC	BC	CC	CC	CC
RC Double Occupant Position ID	N_1-N_2	AB								-(Y+Z)	-(Y+Z)	-(Y+Z)
	N_1-N_3	AC						-X	-X			-Z
	N_1-N_4	AC					-X		-X		-Z	
	N_1-N_5	AC					-X	-X		-Z		
	N_2-N_3	BC			+X	+X						-Y
	N_2-N_4	BC		+X		+X					-Y	
	N_2-N_5	BC		+X	+X					-Y		
	N_3-N_4	CC	Y+Z			+Z			+Y			
	N_3-N_5	CC	Y+Z		+Z			+Y				
	N_4-N_5	CC	Y+Z	+Z			+Y					

Algebraic Equations:

AB – CC = Y + Z

AC – BC = A – B = X

BC – AC = B – A = -X

AC – CC = A – C = Z

CC – AC = C –A = -Z

BC – CC = B – C = Y

CC – BC = C-B = -Y

CC – AB = -(Y+Z)

Figure S11. Algebraic Equations of the 11-State, 25-State, 51-State DNA Elements (2,2,1,0,0) (2,1,1,1,0) (1,1,1,1,1)

All Possible Discrete States For a 5-nt DNA Element in the Genome
(a 5-nt DNA element, followed by any arbitrary spacer, followed by its 5-nt reverse-complement DNA element)

Independent Variables	DNA Element	Position N_1	Position N_2	Position N_3	Position N_4	Position N_5	Total	Plateaus
1		5	0	0	0	0	5	1
2	HRE	4	1				5	3
	ERE	3	2				5	5
3		3	1	1			5	9
		2	2	1			5	11
4		2	1	1	1		5	25
5		1	1	1	1	1	5	51

Figure S12. (S/N) analysis of 1-nt Variant EREs in ER ChIPSeq Peaks (Variant Position)

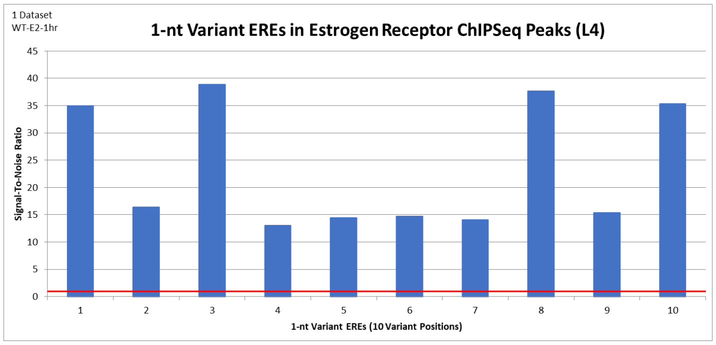

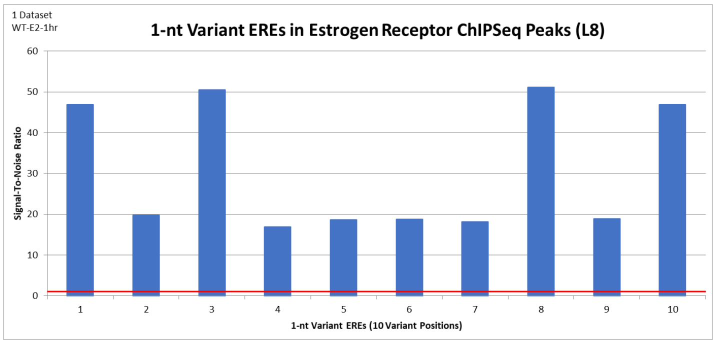

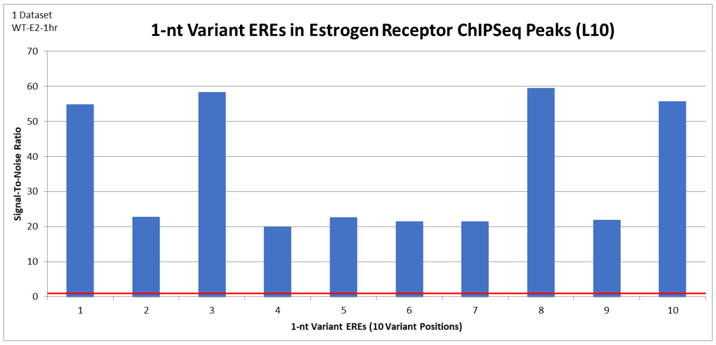

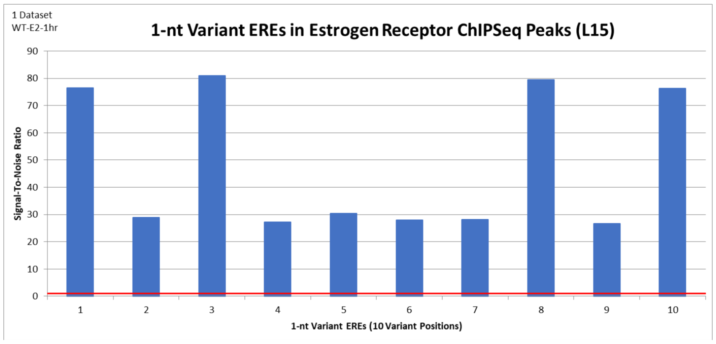

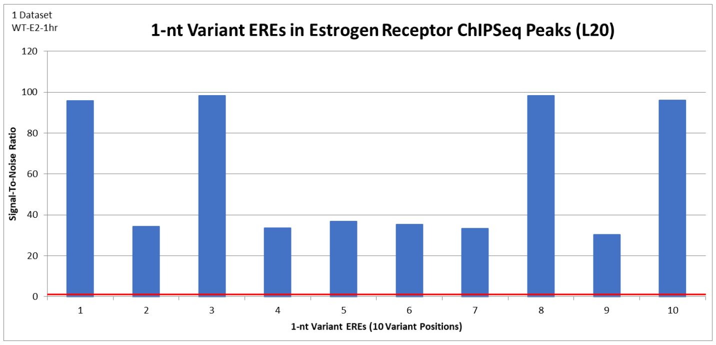

Figure S13. (S/N) analysis of 2-nt Variant EREs in ER ChIPSeq Peaks (Variant Position)

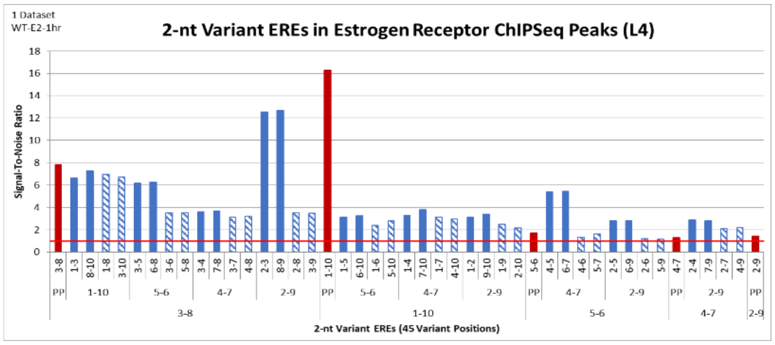

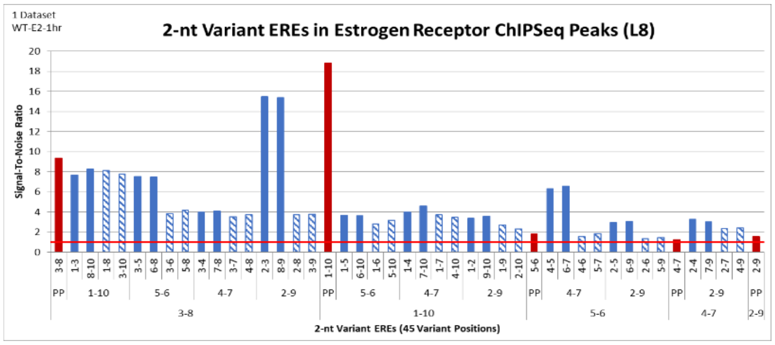

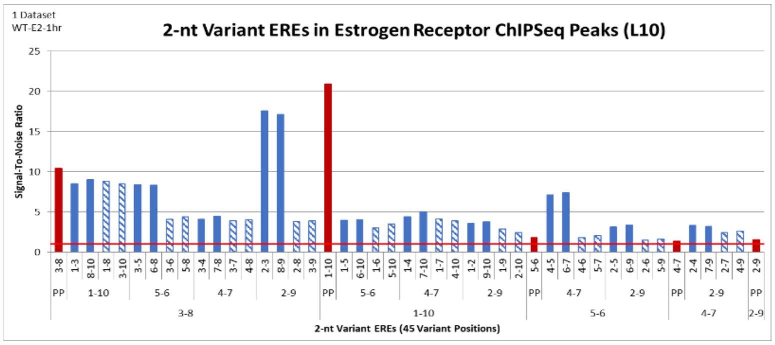

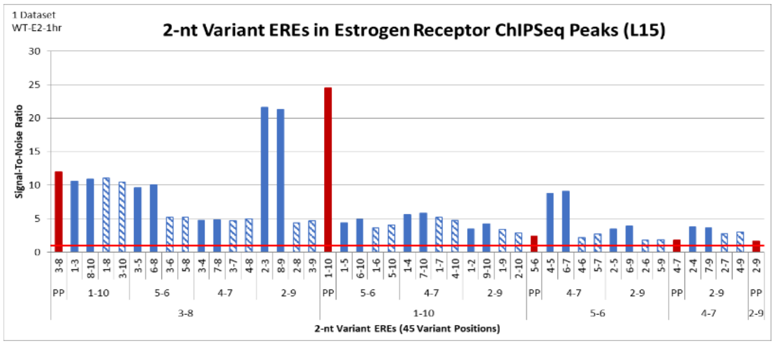

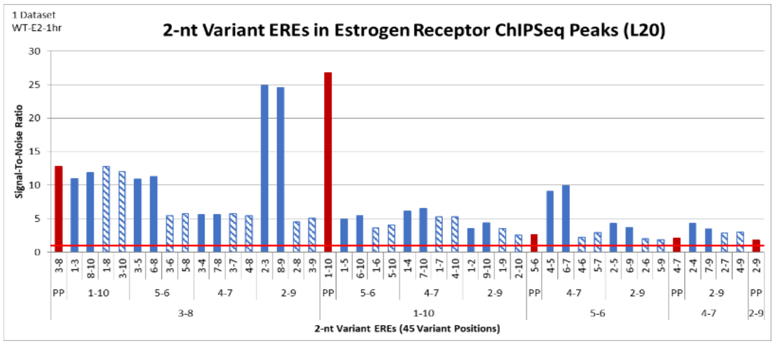

Figure S14. (S/N) analysis of 3-nt Variant EREs in ER ChIPSeq Peaks (Variant Position)

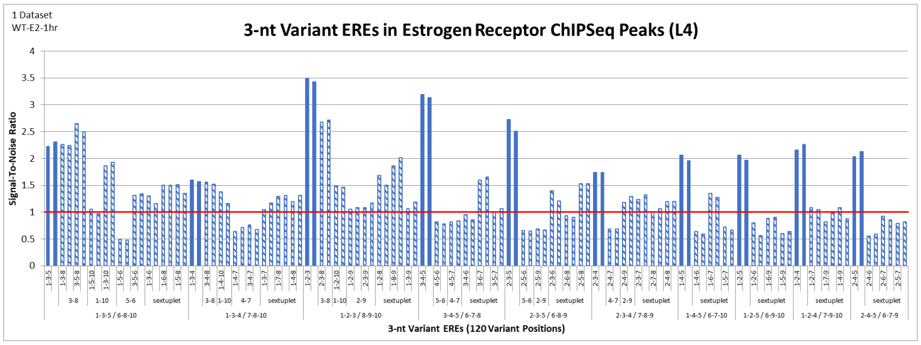

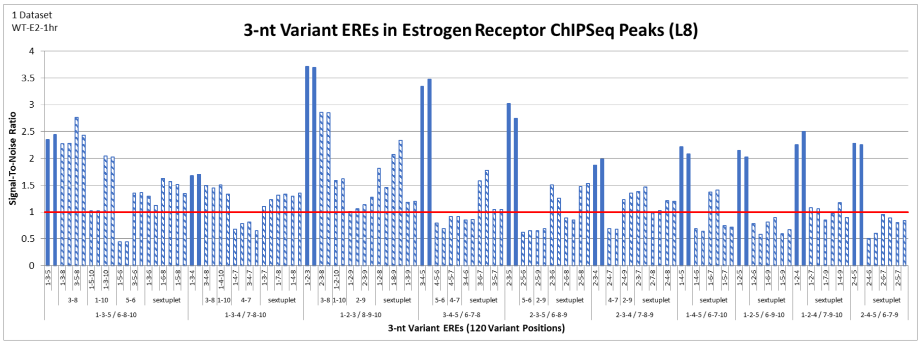

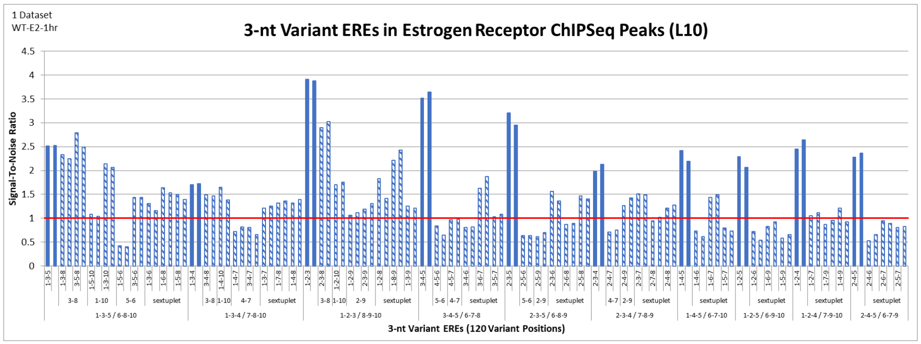

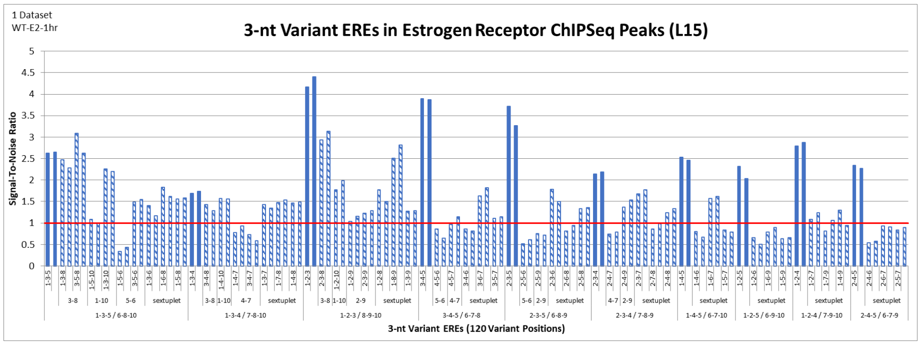

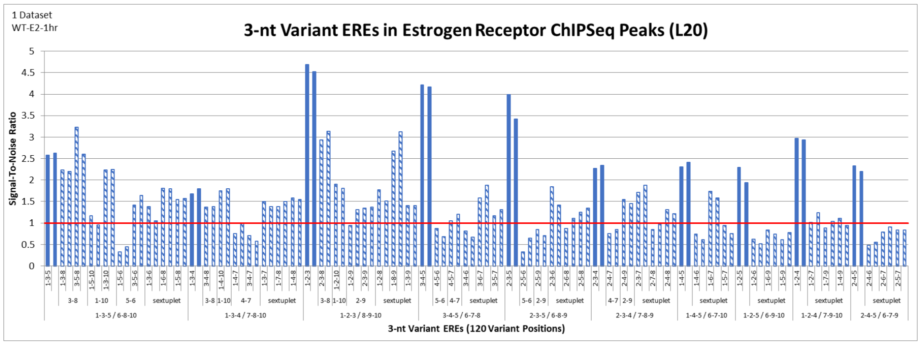

Figure S15. (S/N) analysis of 4-nt Variant EREs in ER ChIPSeq Peaks (Variant Position)

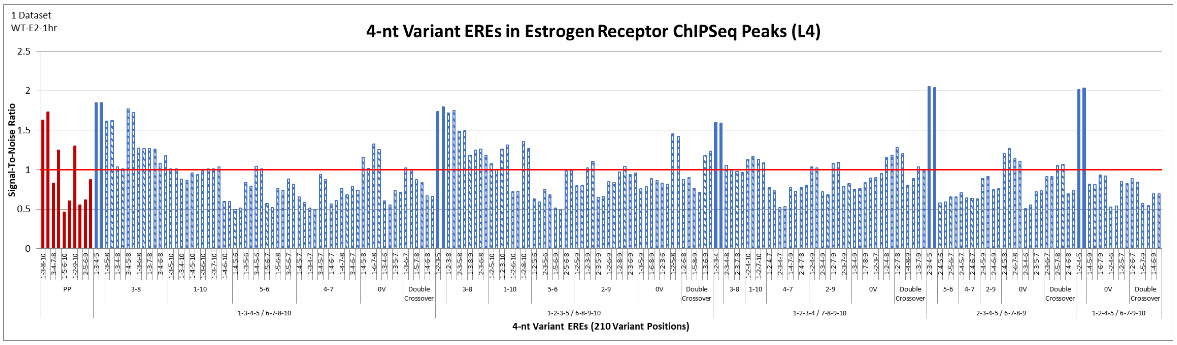

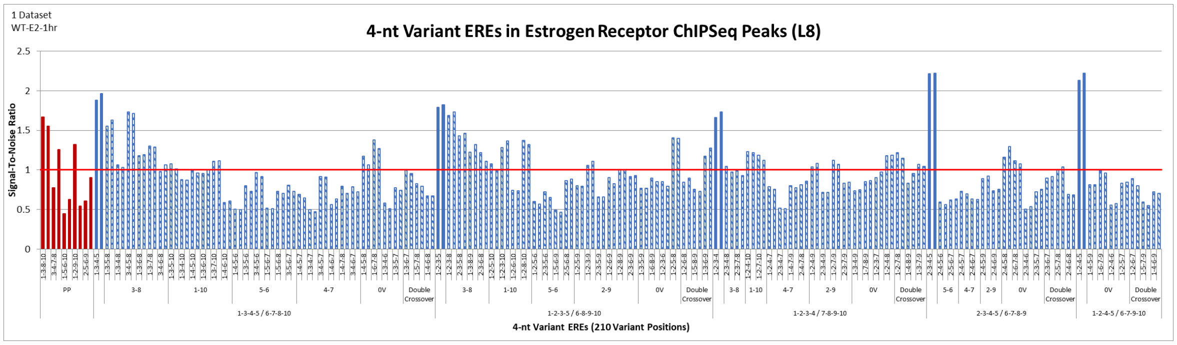

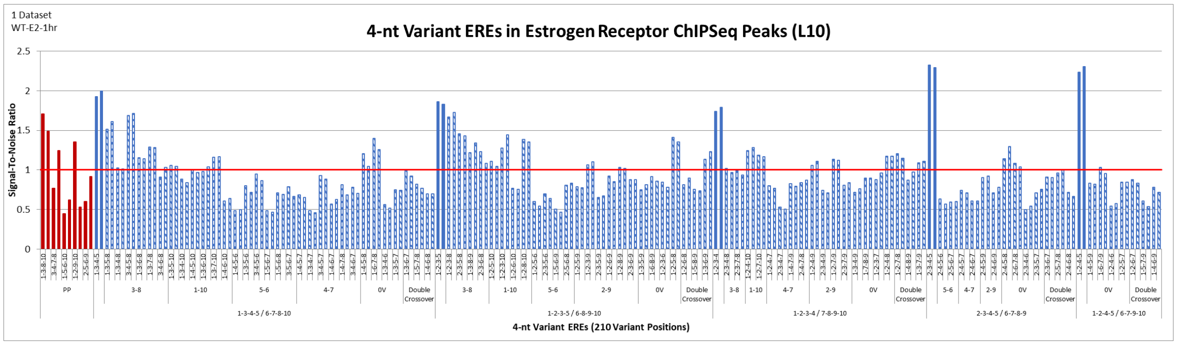

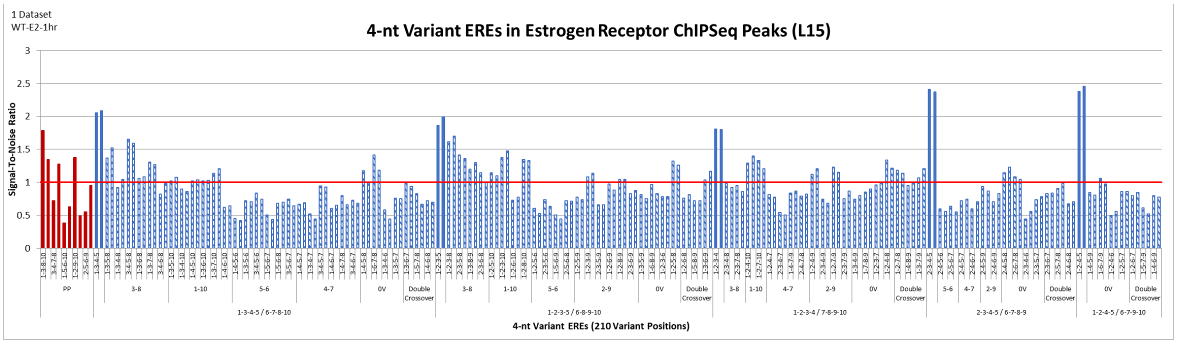

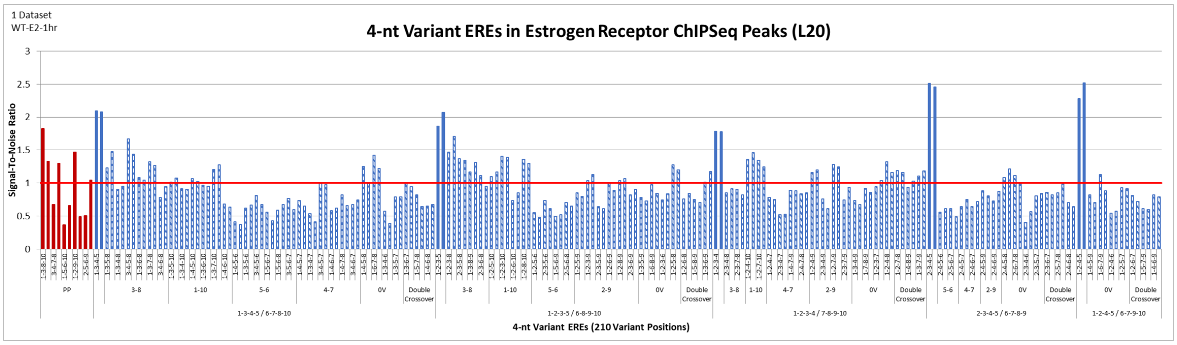

Figure S16. (S/N) analysis of 5-nt Variant EREs in ER ChIPSeq Peaks (Variant Position)

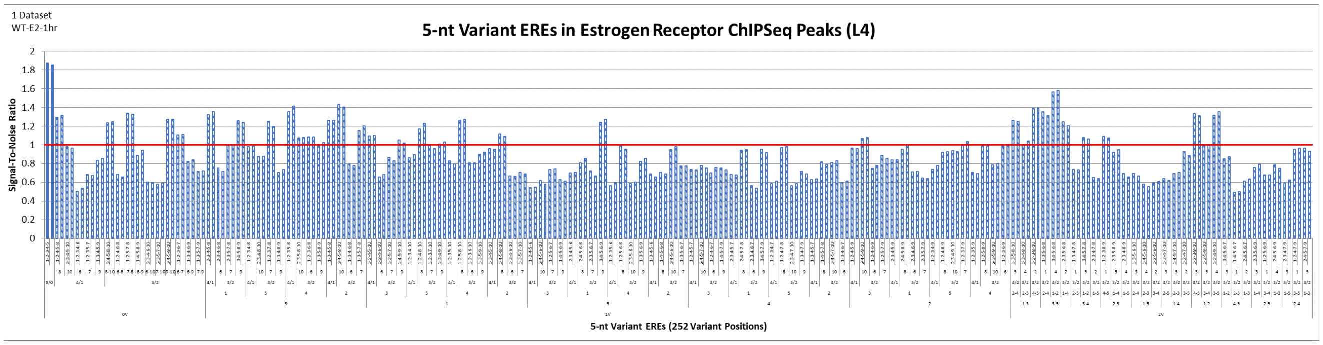

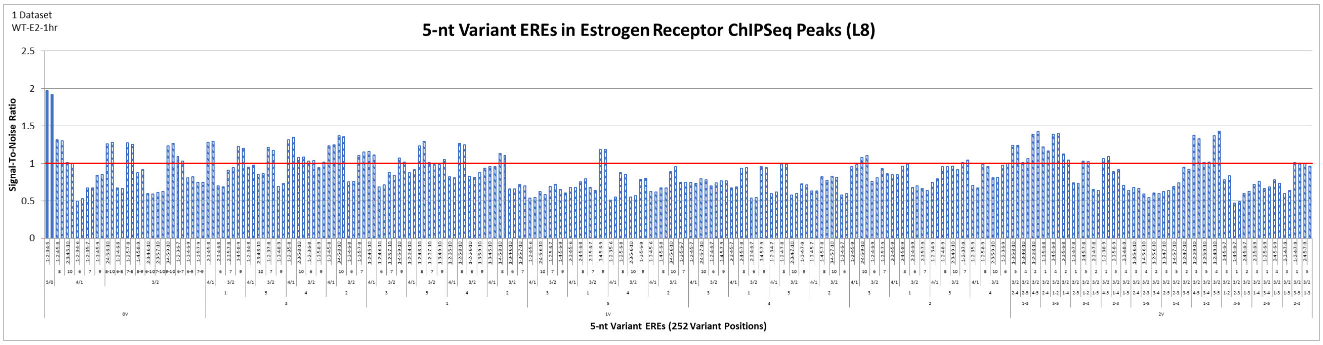

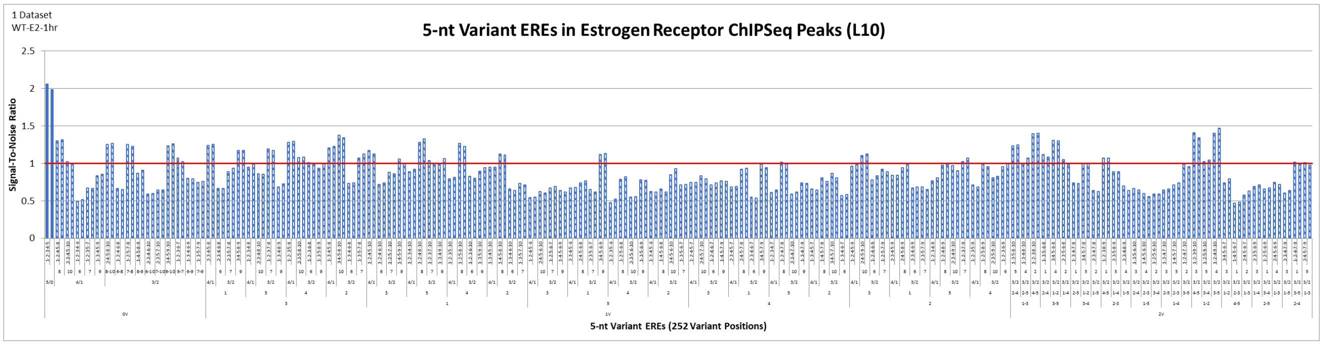

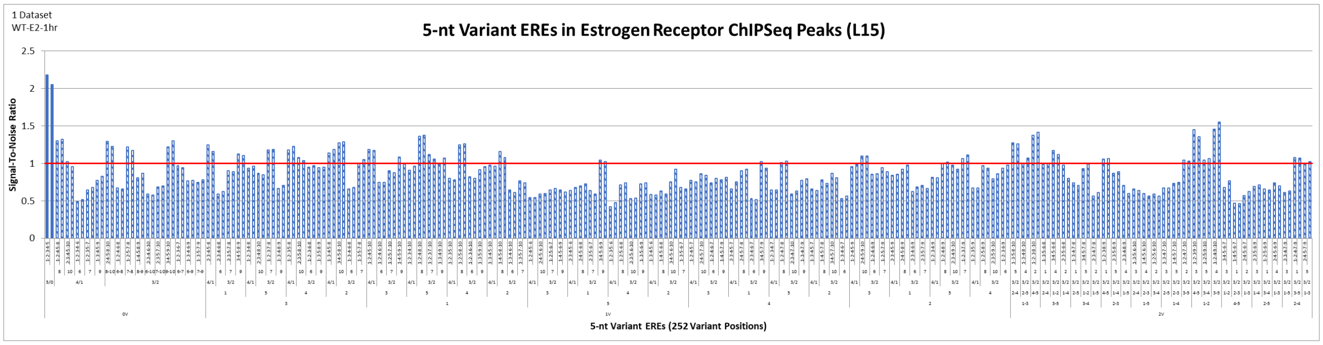

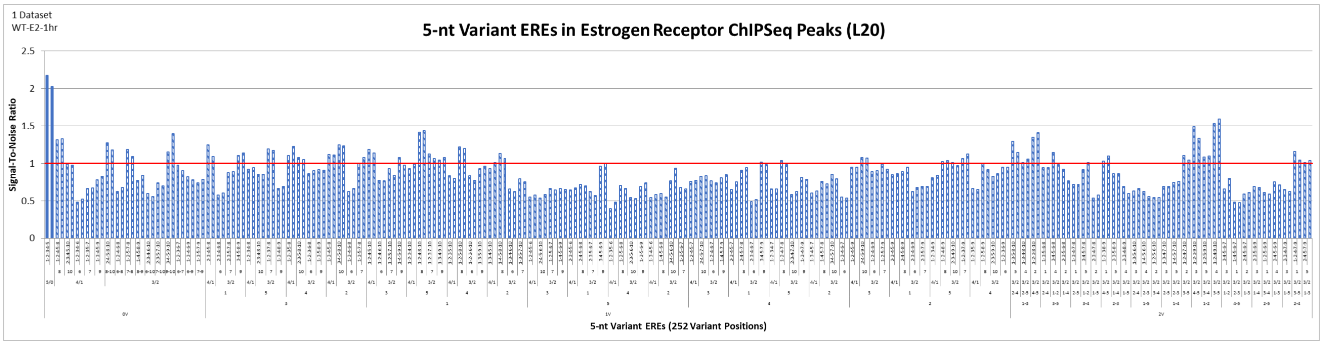

Figure S17. (S/N) analysis of 1-nt Variant HREs in KR ChIPSeq Peaks (Variant Position)

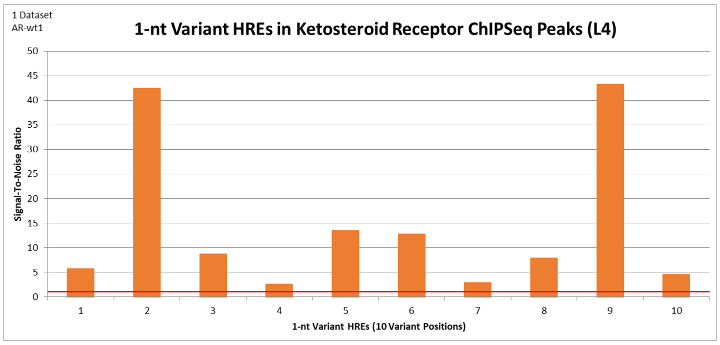

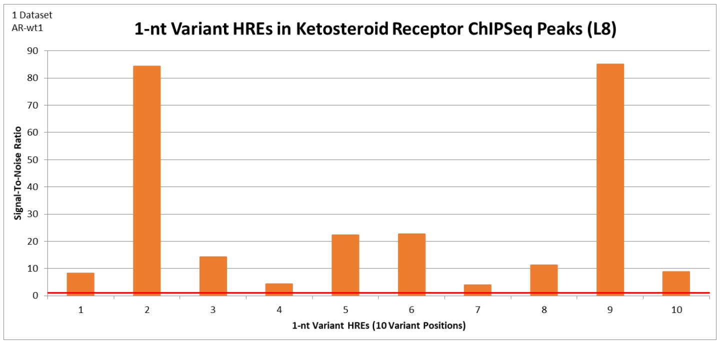

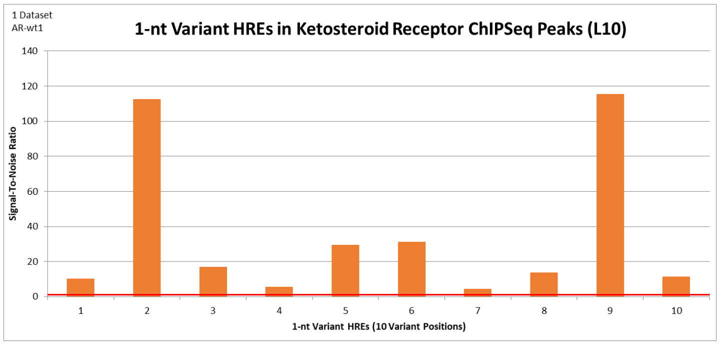

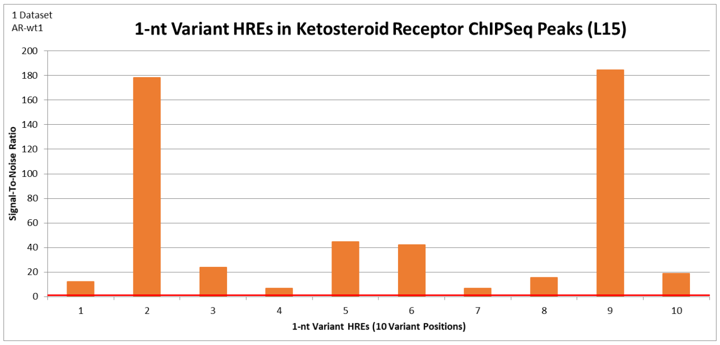

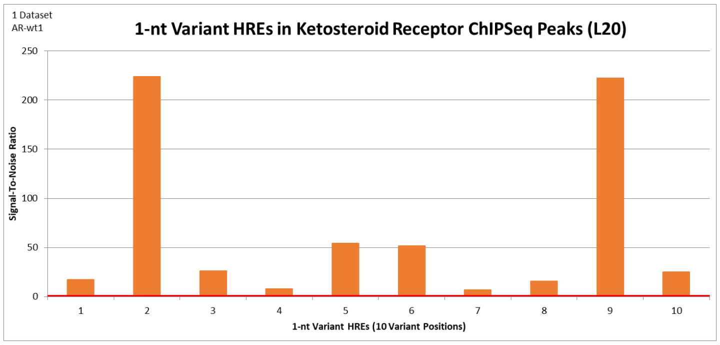

Figure S18. (S/N) analysis of 2-nt Variant HREs in KR ChIPSeq Peaks (Variant Position)

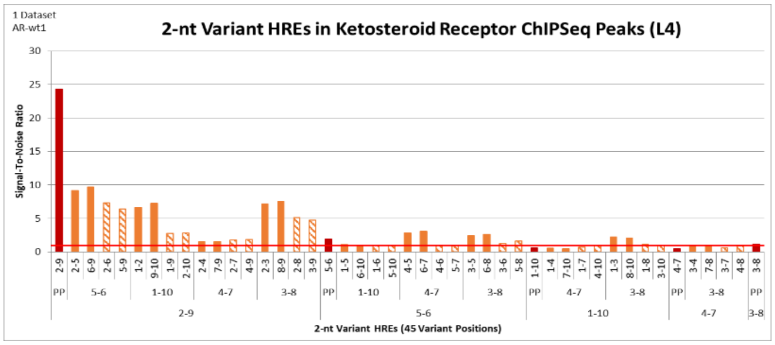

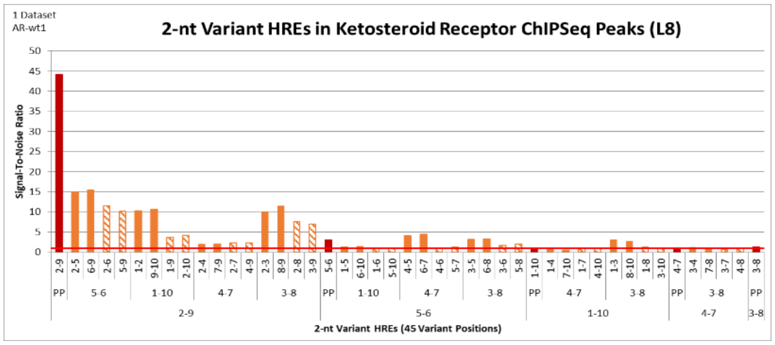

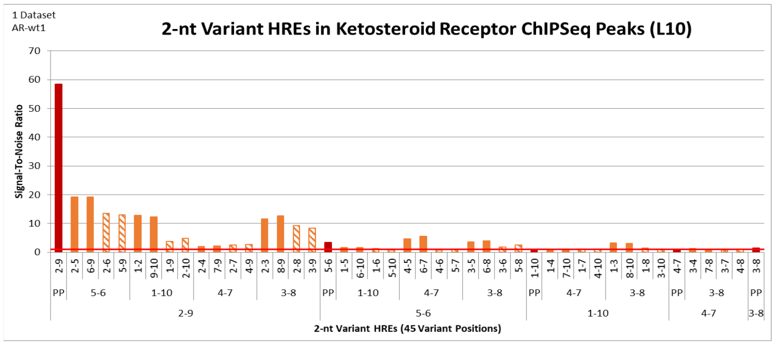

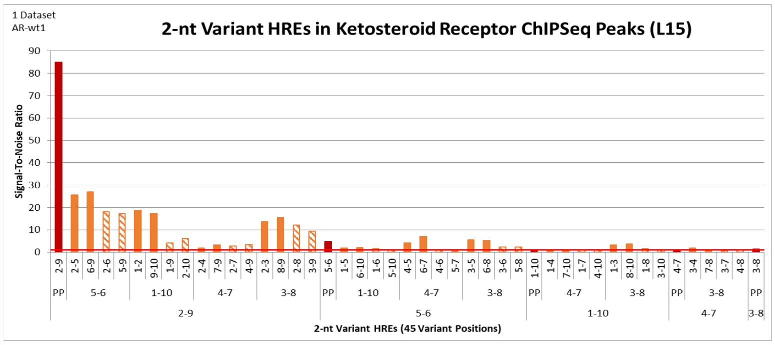

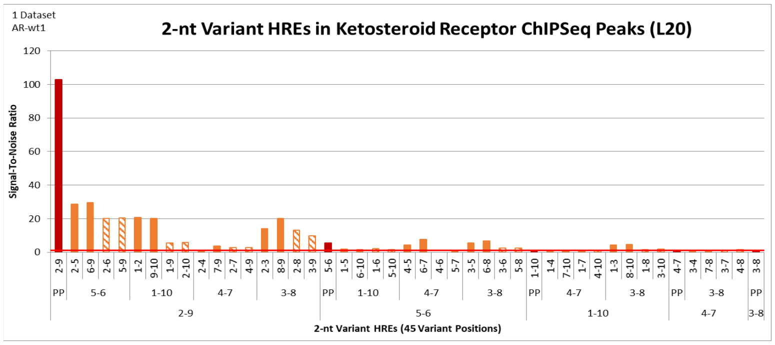

Figure S19. (S/N) analysis of 3-nt Variant HREs in KR ChIPSeq Peaks (Variant Position)

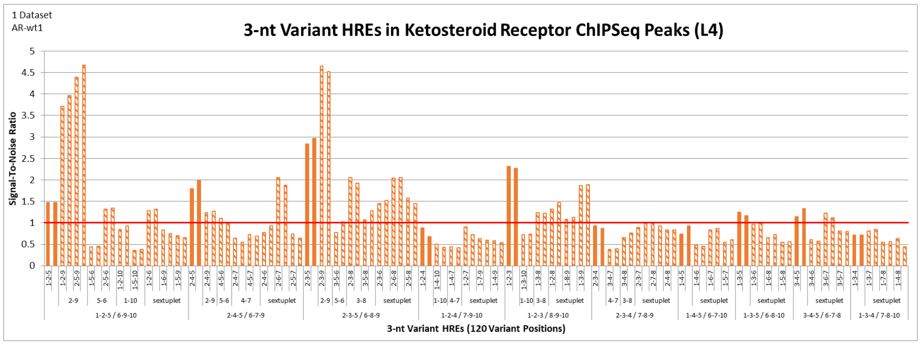

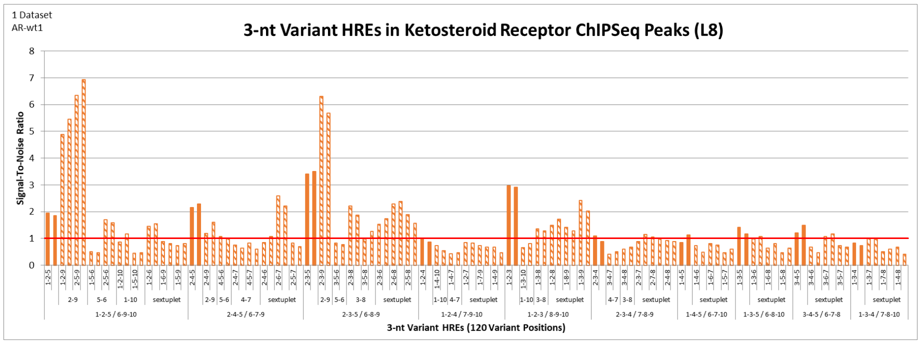

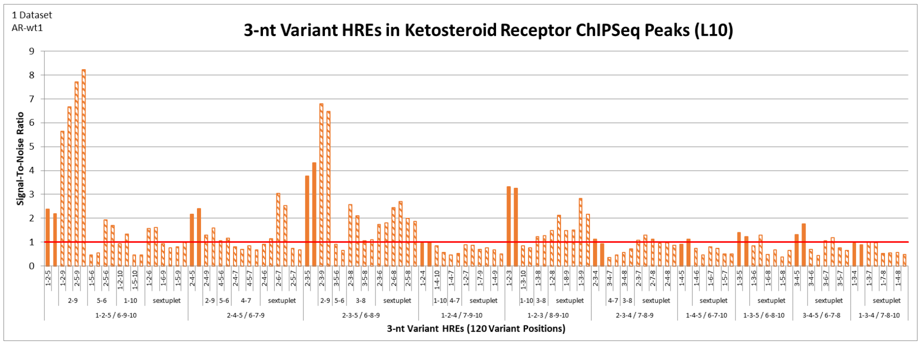

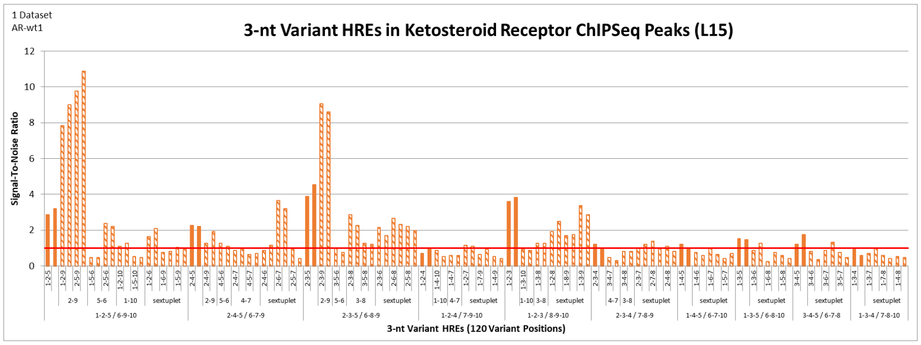

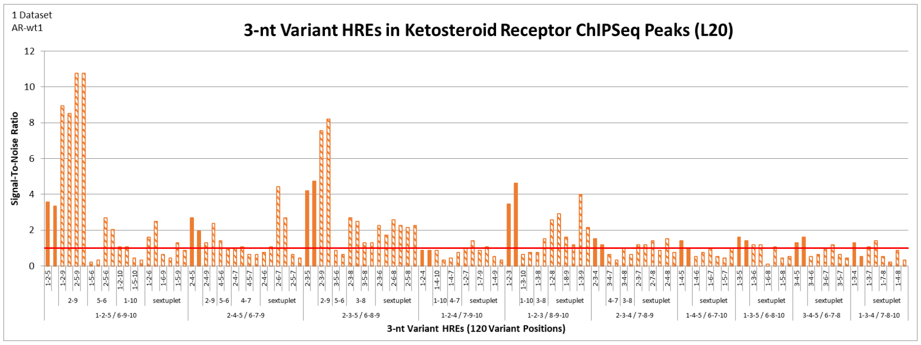

Figure S20. (S/N) analysis of 4-nt Variant HREs in KR ChIPSeq Peaks (Variant Position)

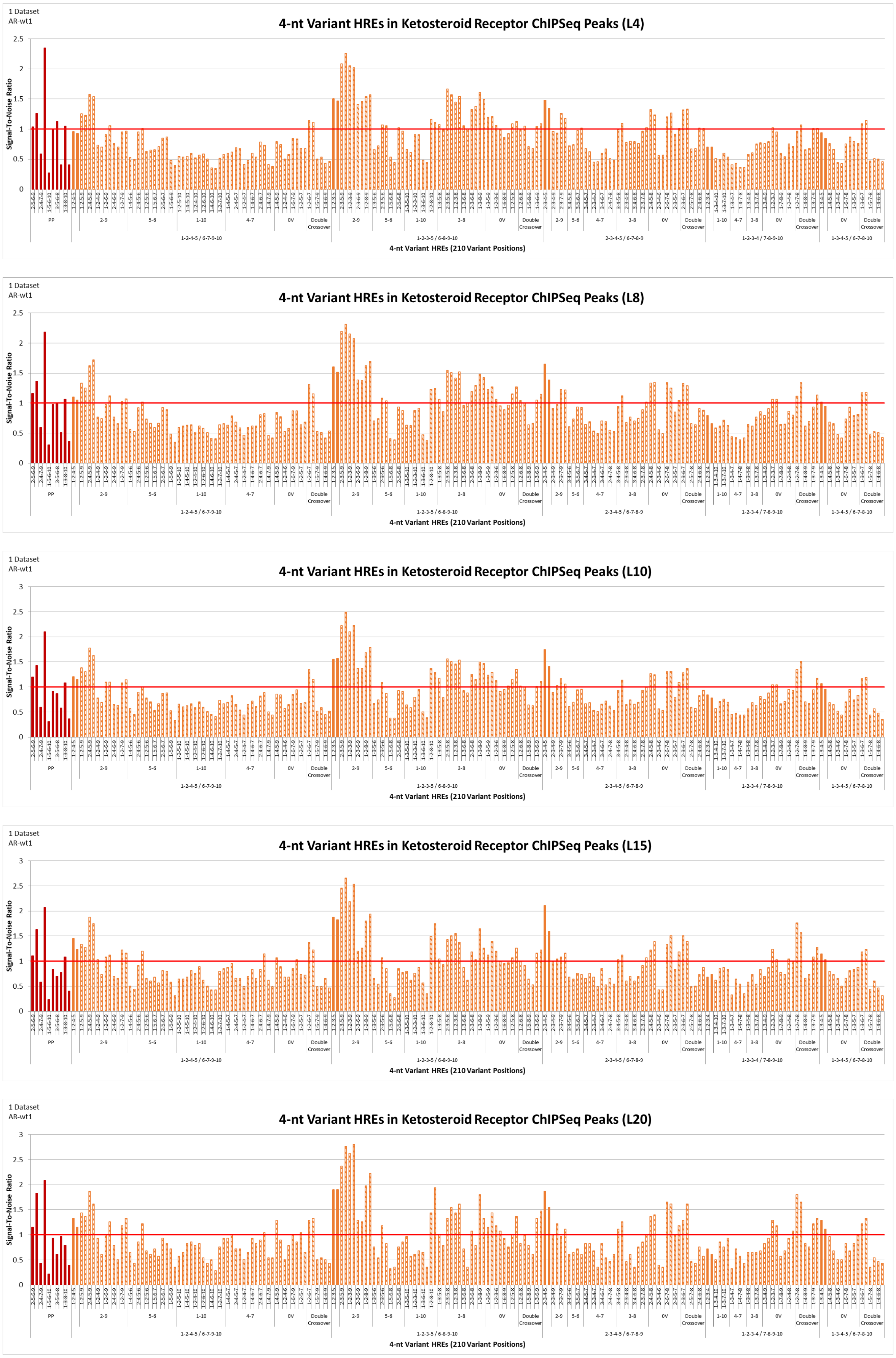

Figure S21. (S/N) analysis of 5-nt Variant HREs in KR ChIPSeq Peaks (Variant Position)

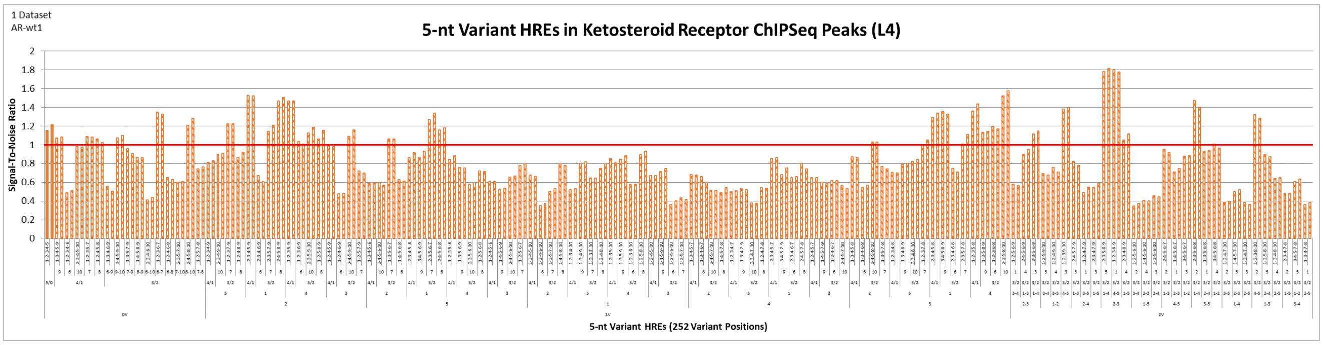

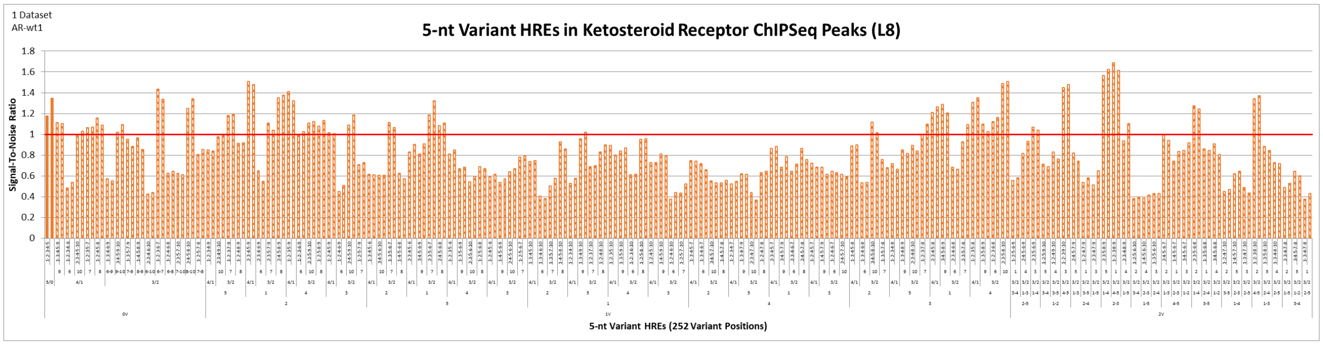

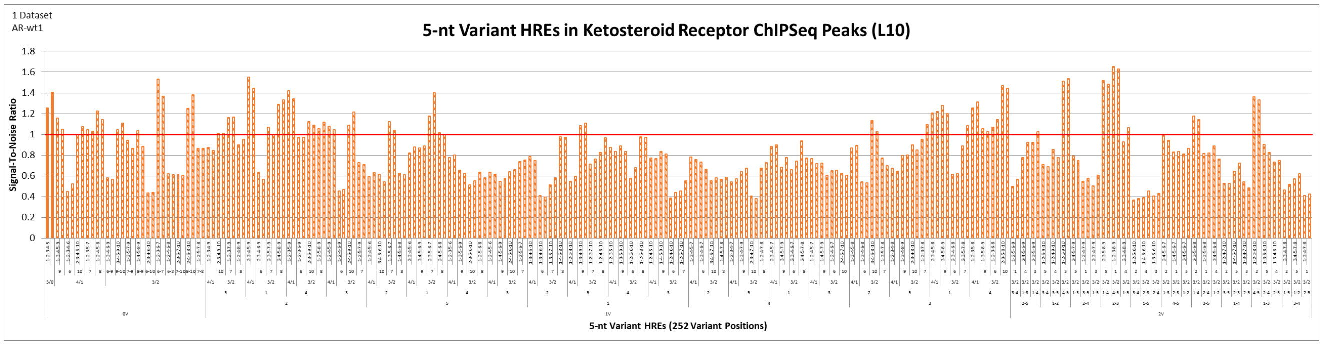

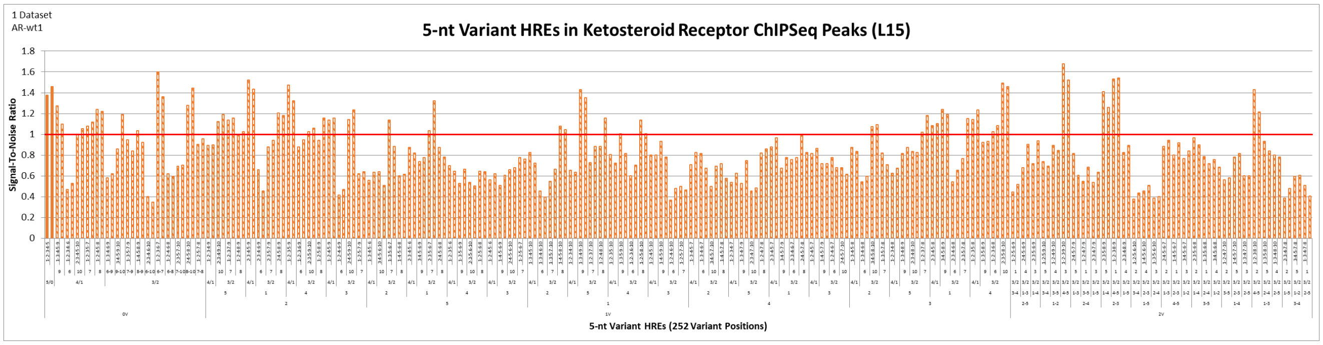

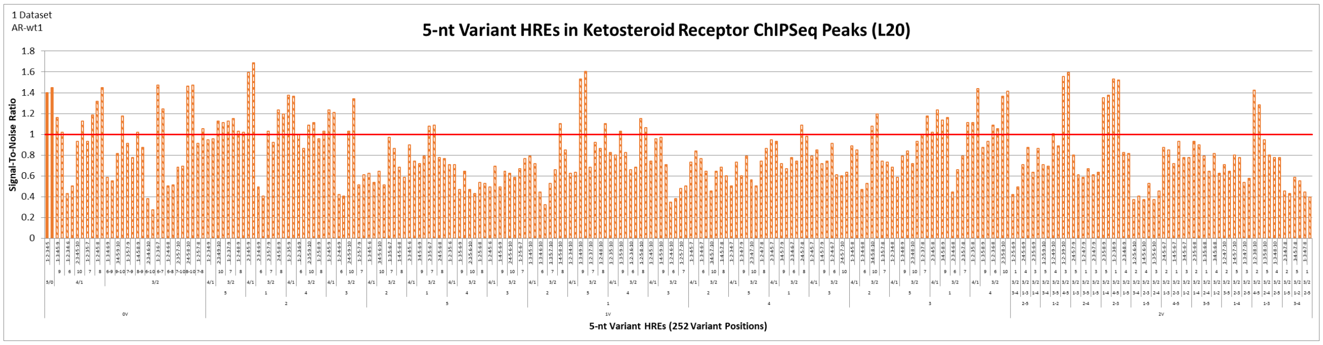

Figure S22. (S/N) analysis of 0-nt to 5-nt Variant HREs in GR and GR-Dim ChIPSeq Peaks #2 (252 Half-Site Groups)

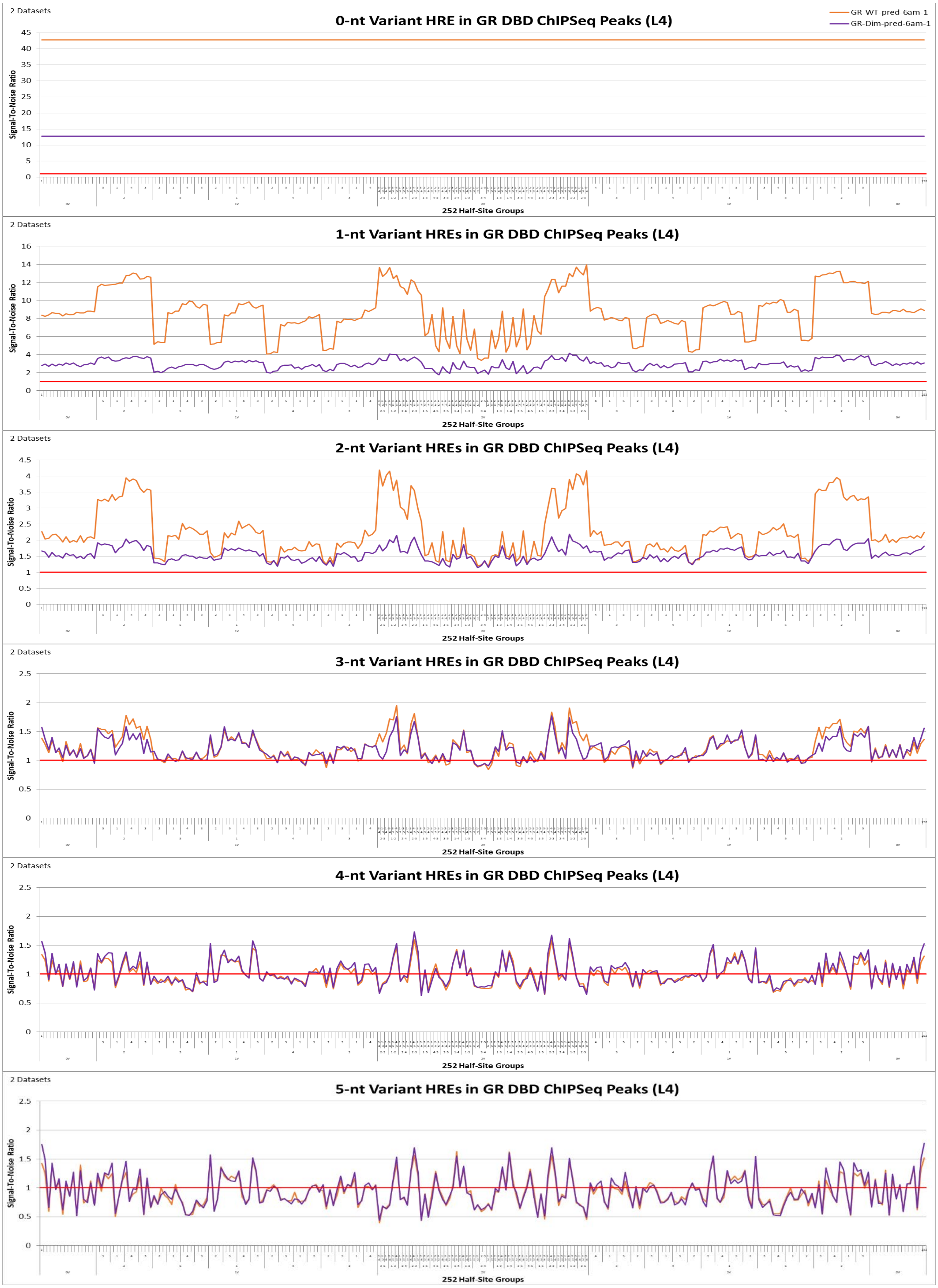

Figure S23. (S/N) analysis of 0-nt to 5-nt Variant HREs in GR and GR-Dim ChIPExo Peaks #1 (252 Half-Site Groups)

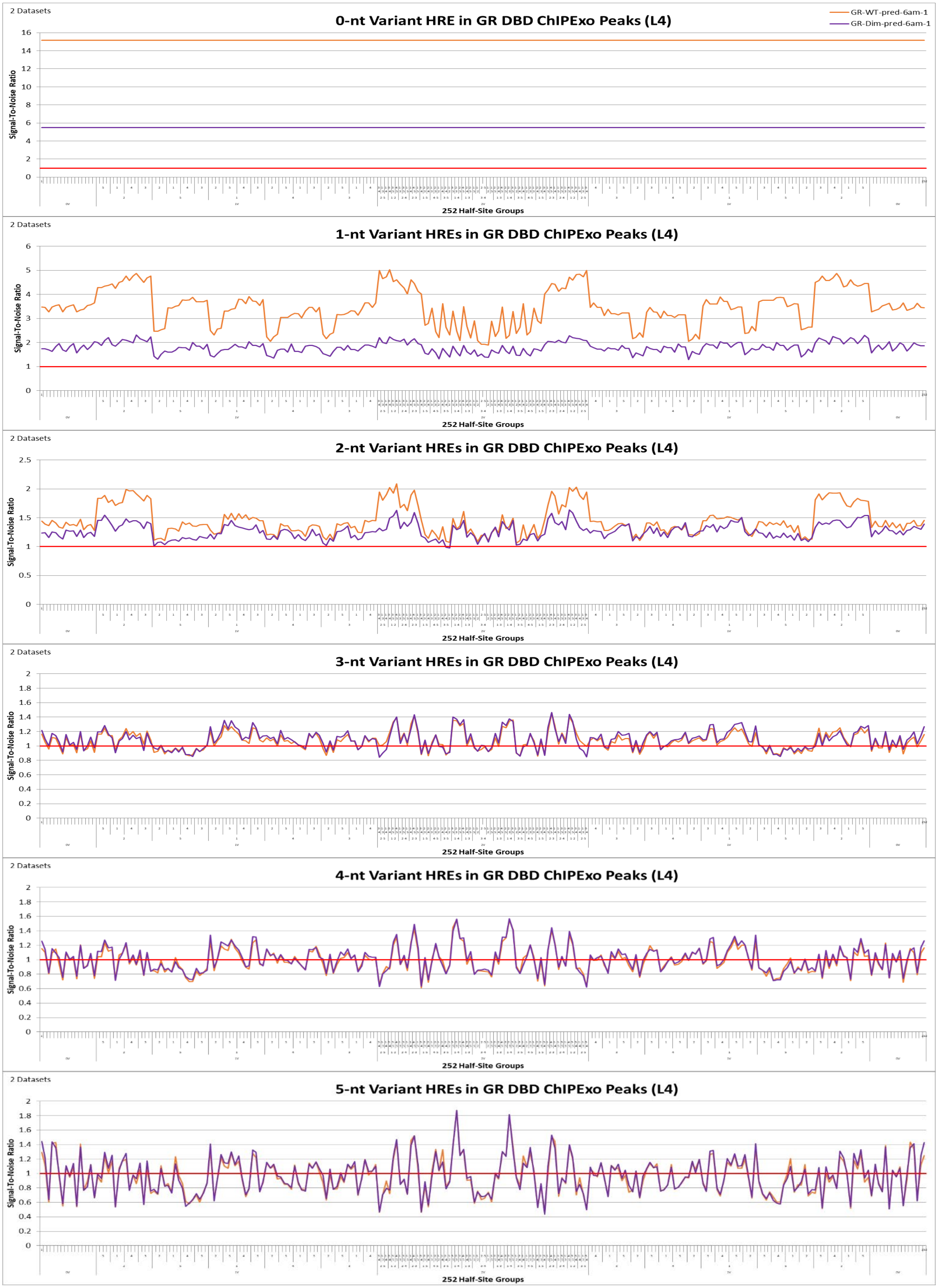

Figure S24. (S/N) analysis of 0-nt to 5-nt Variant HREs in GR and GR-Dim ChIPExo Peaks #2 (252 Half-Site Groups)

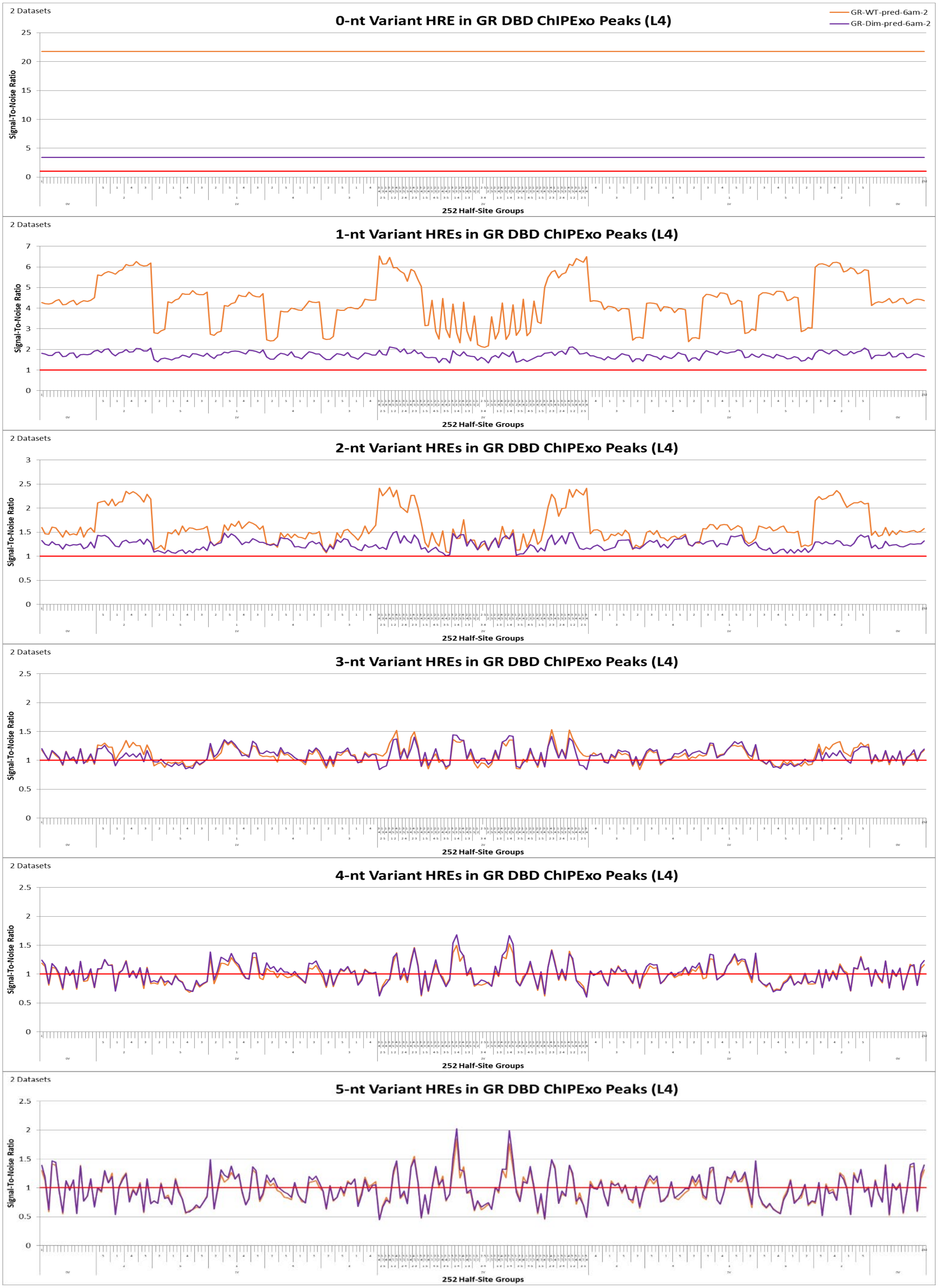

Figure S25. (S/N) analysis of 1-nt to 5-nt Variant HREs in GR and GR-Dim ChIPExo Peaks (Variant Position)

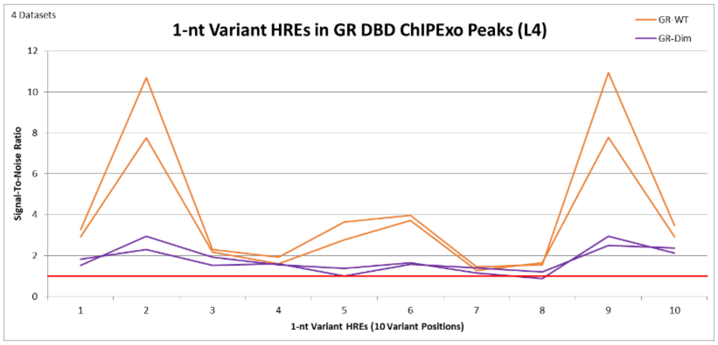

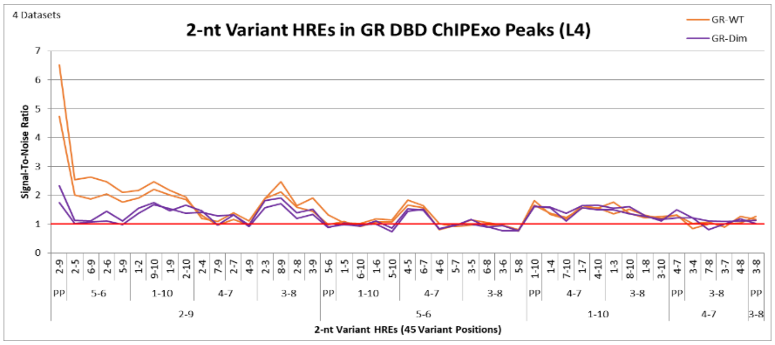

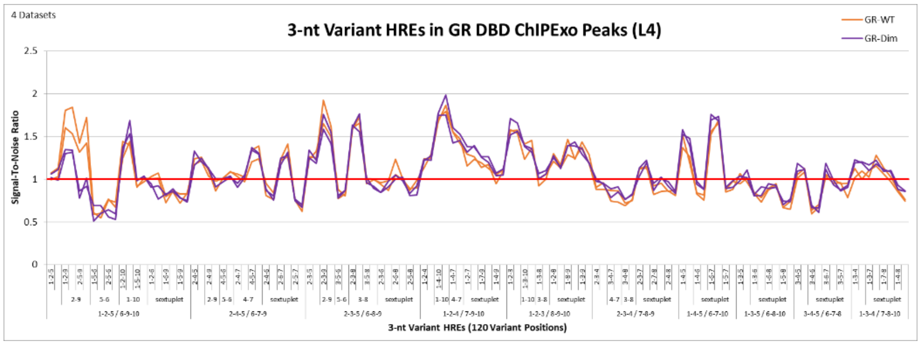

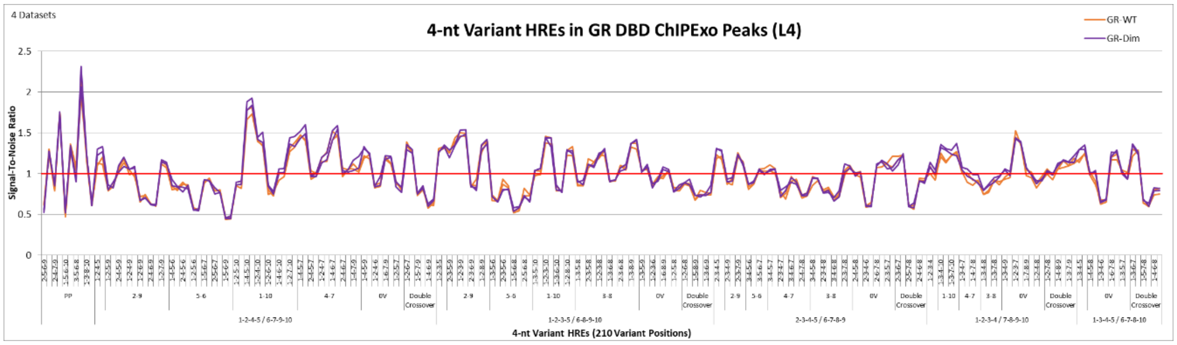

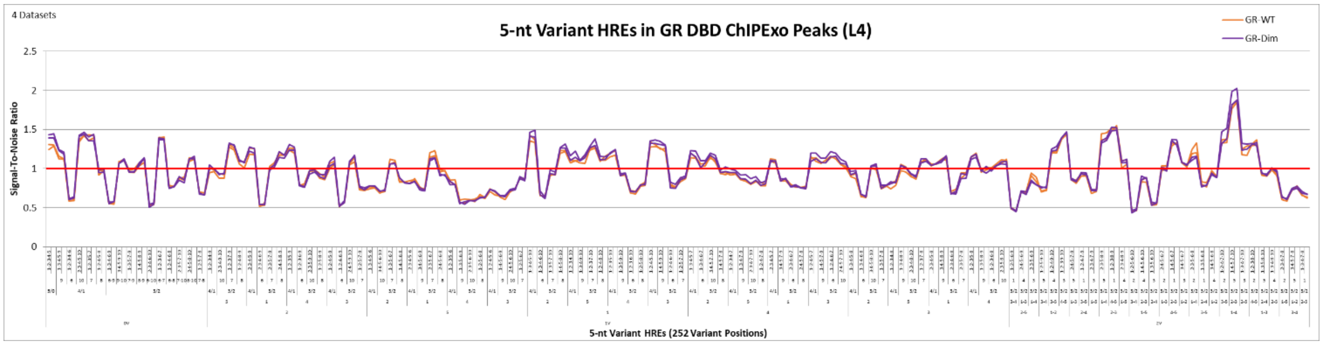

Figure S26-S32 Descriptions: Inversion Symmetry Detection Methodology (Position Weight Matrices)

****All detailed data and statistics associated with every figure are compiled in Data S1****

Scoring DNA sequences against position weight matrices (PWMs) is a widely adopted method to identify TF *cis*-regulatory DNA elements in ChIPSeq or ChIPExo experiments. PWMs are used to scan a DNA sequence for the presence of DNA sequences that are significantly more similar to the PWMs than to the background (1). To assess the resolution capabilities of PWMs-based DNA motif identification, we grouped the ChIPSeq or ChIPExo peaks from each experiment by the number of variants each ERE or HRE DNA element within the peak contained (i.e., peaks that contain a 0-nt variant consensus palindromic DNA element, 1-nt variant DNA element, 2-nt variant DNA element, 3-nt variant DNA element, 4-nt variant DNA element, or 5-nt variant DNA element) and ran these grouped peaks against PWMs. Because peaks can contain multiple ERE or HRE DNA elements, we then assigned the peaks to the ERE or HRE DNA element with the least number of variants relative to the 0-nt consensus palindromic DNA element, thus defining each peak by a single ERE or HRE DNA element (i.e., unique).

Using WT-E2-1hr.L4 [76,163 peaks, 146-nt peak length] as an example experiment, 1,201 peaks contain a 0-nt variant consensus palindromic ERE DNA element, 7,349 peaks contain a 1-nt variant ERE DNA element, 16,368 peaks contain a 2-nt variant ERE DNA element, 34,605 peaks contain a 3-nt variant ERE DNA element, 68,484 peaks contain a 4-nt variant ERE DNA element, and 76,147 peaks contain a 5-nt variant ERE DNA element. PWMs identified the ERE motif (VAGGTCACNSTGACC) in 1,201 (100%) of the peaks that contain 0-nt variant consensus palindromic ERE DNA element, 7,170 (98%) of the peaks that contain a 1-nt variant ERE DNA element, 8,494 (52%) of the peaks that contain a 2-nt variant ERE DNA element, 7,107 (21%) of the peaks that contain a 3-nt variant ERE DNA element, 14,846 (22%) of the peaks that contain a 4-nt variant ERE DNA element, 16,816 (22%) of the peaks that contain a 5-nt variant ERE DNA element, and 16,820 (22%) of all the peaks in the experiment (76,163). We then assigned the peaks to the ERE DNA element with the least number of variants relative to the 0-nt consensus palindromic DNA element, thus defining each peak by a single ERE or HRE DNA element (i.e., unique): 1,201 peaks contain a 0-nt variant consensus palindromic ERE DNA element, 7,332 peaks contain a 1-nt variant ERE DNA element, 15,490 peaks contain a 2-nt variant ERE DNA element, 24,347 peaks contain a 3-nt variant ERE DNA element, 25,194 peaks contain a 4-nt variant ERE DNA element, and 2,599 peaks contains a 5-nt variant ERE DNA element. PWMs identified the ERE motif (VAGGTCACNSTGACC) in 1,201 (100%) of the peaks that contain 0-nt variant consensus palindromic ERE DNA element, 7,153 (98%) of the peaks that contain a 1-nt variant ERE DNA element, 7,631 (49%) of the peaks that contain a 2-nt variant ERE DNA element, 582 (2%) of the peaks that contain a 3-nt variant ERE DNA element, 227 (1%) of the peaks that contain a 4-nt variant ERE DNA element, and 26 (1%) of the peaks that contain a 5-nt variant ERE DNA element. Thus, PWMs identified the ERE motif in the majority of peaks that contain a 0-nt or a 1-nt variant ERE DNA element, about half of the peaks that contain a 2-nt variant ERE DNA element, and did not identify the ERE motif in peaks that contain a 3-nt, 4-nt, or 5-nt variant ERE DNA element. Therefore, PWMs analyses would lead one to conclude that the majority of ER DNA-binding events in the genome are being driven by mechanisms other than the ERE sequence since peaks that contain a 0-nt variant consensus palindromic ERE DNA element or 1-nt variant ERE DNA element constitute 11% of all ER DNA-binding events in the experiment, a proportion observed in 157 ER experiments, representing a wide variety of mouse tissues and human cell lines, and across multiple peak selection criteria (L4-L20) (2).

Figure S26. PWMs Detection of the ERE DNA Motif in ER ChIPSeq Peaks
(A) Of 157 ER experiments, on average, PWMs identified the ERE motif (VAGGTCACNSTGACC) in 99%-99% (L4-L20) of the peaks that contain 0-nt variant consensus palindromic ERE DNA element, 95%-97% (L4-L20) of the peaks that contain a 1-nt variant ERE DNA element, 50%-58% (L4-L20) of the peaks that contain a 2-nt variant ERE DNA element, 26%-38% (L4-L20) of the peaks that contain a 3-nt variant ERE DNA element, 27%-41% (L4-L20) of the peaks that contain a 4-nt variant ERE DNA element, and 26%-41% (L4-L20) of the peaks that contain a 5-nt variant ERE DNA element. **(B)** Because peaks can contain multiple ERE DNA elements, we then assigned peaks to the ERE DNA element with the least number of variants relative to the 0-nt consensus palindromic ERE, thus defining each peak by a single ERE DNA element (i.e., unique). Now, PWMs identified the ERE motif in 99%-99% (L4-L20) of the peaks that contain 0-nt variant consensus palindromic ERE DNA element, 95%-97% (L4-L20) of the peaks that contain a 1-nt variant ERE DNA element, 46%-52% (L4-L20) of the peaks that contain a 2-nt variant ERE DNA element, 3%-4% (L4-L20) of the peaks that contain a 3-nt variant ERE DNA element, 1%-1% (L4-L20) of the peaks that contain a 4-nt variant ERE DNA element, and 1%-1% (L4-L20) of the peaks that contain a 5-nt variant ERE DNA element.

Figure S27. PWMs Detection of the ARE DNA Motif in KR ChIPSeq Peaks
(A) Of 194 KR experiments, on average, PWMs identified the ARE motif (RGRACASNSTGTYCYB) in 96%-96% (L4-L20) of the peaks that contain 0-nt variant consensus palindromic HRE DNA element, 76%-81% (L4-L20) of the peaks that contain a 1-nt variant HRE DNA element, 41%-50% (L4-L20) of the peaks that contain a 2-nt variant HRE DNA element, 19%-29% (L4-L20) of the peaks that contain a 3-nt variant HRE DNA element, 18%-29% (L4-L20) of the peaks that contain a 4-nt variant HRE DNA element, and 18%-30% (L4-L20) of the peaks that contain a 5-nt variant HRE DNA element. **(B)** Because peaks can contain multiple HRE DNA elements, we then assigned peaks to the HRE DNA element with the least number of variants relative to the 0-nt consensus palindromic HRE, thus defining each peak by a single HRE DNA element (i.e., unique). Now, PWMs identified the ARE motif in 96%-96% (L4-L20) of the peaks that contain 0-nt variant consensus palindromic HRE DNA element, 76%-81% (L4-L20) of the peaks that contain a 1-nt variant HRE DNA element, 39%-47% (L4-L20) of the peaks that contain a 2-nt variant HRE DNA element, 9%-

14% (L4-L20) of the peaks that contain a 3-nt variant HRE DNA element, 1%-2% (L4-L20) of the peaks that contain a 4-nt variant HRE DNA element, and 1%-2% (L4-L20) of the peaks that contain a 5-nt variant HRE DNA element.

Figure S28. PWMs Detection of the GRE DNA Motif in KR ChIPSeq Peaks
(A) Of 194 KR experiments, on average, PWMs identified the GRE motif (NRGVACABNVTGTYCY) in 96%-97% (L4-L20) of the peaks that contain 0-nt variant consensus palindromic HRE DNA element, 64%-72% (L4-L20) of the peaks that contain a 1-nt variant HRE DNA element, 31%-39% (L4-L20) of the peaks that contain a 2-nt variant HRE DNA element, 14%-24% (L4-L20) of the peaks that contain a 3-nt variant HRE DNA element, 14%-24% (L4-L20) of the peaks that contain a 4-nt variant HRE DNA element, and 14%-24% (L4-L20) of the peaks that contain a 5-nt variant HRE DNA element. **(B)** Because peaks can contain multiple HRE DNA elements, we then assigned peaks to the HRE DNA element with the least number of variants relative to the 0-nt consensus palindromic HRE, thus defining each peak by a single HRE DNA element (i.e., unique). Now, PWMs identified the GRE motif in 96%-97% (L4-L20) of the peaks that contain 0-nt variant consensus palindromic HRE DNA element, 64%-71% (L4-L20) of the peaks that contain a 1-nt variant HRE DNA element, 29%-37% (L4-L20) of the peaks that contain a 2-nt variant HRE DNA element, 6%-10% (L4-L20) of the peaks that contain a 3-nt variant HRE DNA element, 1%-2% (L4-L20) of the peaks that contain a 4-nt variant HRE DNA element, and 1%-1% (L4-L20) of the peaks that contain a 5-nt variant HRE DNA element.

Figure S29. PWMs Detection of the GRE 2 DNA Motif in KR ChIPSeq Peaks
(A) Of 194 KR experiments, on average, PWMs identified the GRE 2 motif (VAGRACAKWCTGTYC) in 99%-100% (L4-L20) of the peaks that contain 0-nt variant consensus palindromic HRE DNA element, 84%-88% (L4-L20) of the peaks that contain a 1-nt variant HRE DNA element, 43%-53% (L4-L20) of the peaks that contain a 2-nt variant HRE DNA element, 19%-31% (L4-L20) of the peaks that contain a 3-nt variant HRE DNA element, 19%-31% (L4-L20) of the peaks that contain a 4-nt variant HRE DNA element, and 19%-31% (L4-L20) of the peaks that contain a 5-nt variant HRE DNA element. **(B)** Because peaks can contain multiple HRE DNA elements, we then assigned peaks to the HRE DNA element with the least number of variants relative to the 0-nt consensus palindromic HRE, thus defining each peak by a single HRE DNA element (i.e., unique). Now, PWMs identified the GRE 2 motif in 99%-100% (L4-L20) of the peaks that contain 0-nt variant consensus palindromic HRE DNA element, 84%-88% (L4-L20) of the peaks that contain a 1-nt variant HRE DNA element, 42%-51% (L4-L20) of the peaks that contain a 2-nt variant HRE DNA element, 8%-13% (L4-L20) of the peaks that contain a 3-nt variant HRE DNA element, 1%-2% (L4-L20) of the peaks that contain a 4-nt variant HRE DNA element, and 1%-1% (L4-L20) of the peaks that contain a 5-nt variant HRE DNA element.

Figure S30. PWMs Detection of the PRE DNA Motif in KR ChIPSeq Peaks
(A) Of 194 KR experiments, on average, PWMs identified the PRE motif (VAGRACAKNCTGTBC) in 99%-100% (L4-L20) of the peaks that contain 0-nt variant consensus palindromic HRE DNA element, 99%-99% (L4-L20) of the peaks that contain a 1-nt variant HRE DNA element, 86%-90% (L4-L20) of the peaks that contain a 2-nt variant HRE DNA element, 59%-69% (L4-L20) of the peaks that contain a 3-nt variant HRE DNA element, 50%-64% (L4-L20) of the peaks that contain a 4-nt variant HRE DNA element, and 50%-63% (L4-L20) of the peaks that contain a 5-nt variant HRE DNA element. **(B)** Because peaks can contain multiple HRE DNA elements, we then assigned peaks to the HRE DNA element with the least number of variants relative to the 0-nt consensus palindromic HRE, thus defining each peak by a single HRE DNA element (i.e., unique). Now, PWMs identified the PRE motif in 99%-100% (L4-L20) of the peaks that contain 0-nt variant consensus palindromic HRE DNA element, 99%-99% (L4-L20) of the peaks that contain a 1-nt variant HRE DNA element, 85%-89% (L4-L20) of the peaks that contain a 2-nt variant HRE DNA element, 49%-57% (L4-L20) of the peaks that contain a 3-nt variant HRE DNA element, 19%-25% (L4-L20) of the peaks that contain a 4-nt variant HRE DNA element, and 6%-8% (L4-L20) of the peaks that contain a 5-nt variant HRE DNA element.

Figure S31. PWMs Detection of the ARE Palindrome Half-Site DNA Motif in KR ChIPSeq Peaks
(A) Of 194 KR experiments, on average, PWMs identified the ARE Palindrome Half-Site motif (CCAGGAACAG) in 88%-89% (L4-L20) of the peaks that contain 0-nt variant consensus palindromic HRE DNA element, 77%-80% (L4-L20) of the peaks that contain a 1-nt variant HRE DNA element, 69%-73% (L4-L20) of the peaks that contain a 2-nt variant HRE DNA element, 60%-64% (L4-L20) of the peaks that contain a 3-nt variant HRE DNA element, 57%-63% (L4-L20) of the peaks that contain a 4-nt variant HRE DNA element, and 56%-63% (L4-L20) of the peaks that contain a 5-nt variant HRE DNA element. **(B)** Because peaks can contain multiple HRE DNA elements, we then assigned peaks to the HRE DNA element with the least number of variants relative to the 0-nt consensus palindromic HRE, thus defining each peak by a single HRE DNA element (i.e., unique). Now, PWMs identified the ARE Palindrome Half-Site motif in 88%-89% (L4-L20) of the peaks that contain 0-nt variant consensus palindromic HRE DNA element, 77%-80% (L4-L20) of the peaks that contain a 1-nt variant HRE DNA element, 68%-72% (L4-L20) of the peaks that contain a 2-nt variant HRE DNA element, 56%-59% (L4-L20) of the peaks that contain a 3-nt variant HRE DNA element, 46%-50% (L4-L20) of the peaks that contain a 4-nt variant HRE DNA element, and 33%-39% (L4-L20) of the peaks that contain a 5-nt variant HRE DNA element.

Figure S32. PWMs Detection of the Different HRE DNA Motifs (ARE, GRE, GRE 2, PRE, ARE Half) in KR ChIPSeq Peaks
Displaying **Figure S27-S31** side-by-side for comparison purposes. Of 194 KR experiments, there was a wide distribution in the number of peaks identified by PWMs as containing the ARE motif (RGRACASNSTGTYCYB), GRE motif (NRGVACABNVTGTYCY), GRE 2 motif (VAGRACAKWCTGTYC), PRE motif (VAGRACAKNCTGTBC), or ARE palindrome half-site motif (CCAGGAACAG). For example, of the peaks that contain a 1-nt variant HRE DNA element, on average, 64%-72% (L4-L20) of the peaks were identified as containing the GRE motif, whereas 99%-99% (L4-L20) of those exact same peaks were identified as containing the PRE motif. Or, of the peaks that contain a 2-nt variant HRE DNA element, 31%-39% (L4-L20) of the

peaks were identified as containing the GRE motif, whereas 86%-90% (L4-L20) of those exact same peaks were identified as containing the PRE motif. Thus, it is apparent why the use of PWMs for DNA motif identification have led investigators to delineate different *cis*-regulatory DNA elements for the different KRs. However, analysis of KR DNA-binding at 81,922 0-nt to 5-nt variant HRE elements in the genome quantitively demonstrates that all members of the KR family follow the same DNA-binding rules, and thus bind the same DNA elements **(Table S8, Table S16-S20)**. These DNA-binding rules at 0-nt to 5-nt variant HRE DNA elements in the genome was observed in 194 KR ChIPSeq experiments, representing a wide variety of mouse tissues and human cell lines, and across multiple peak selection criteria (L4-L20) **(Table S8, Table S16-S20)**.

Figure S26. PWMs Detection of the <u>ERE DNA Motif</u> in ER ChIPSeq Peaks

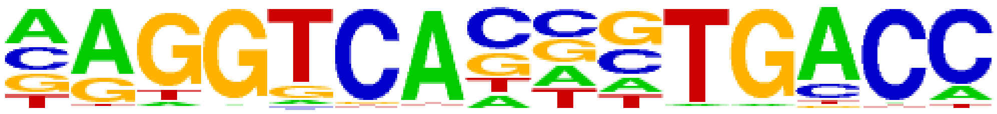

ERE(NR), IR3/MCF7-ERa-ChIPSeq(Unpublished)/Homer

VA<u>GGTCA</u>CNS<u>TGACC</u>

(A) PWMs detection of the <u>ERE DNA motif</u> in ER ChIPSeq Peaks

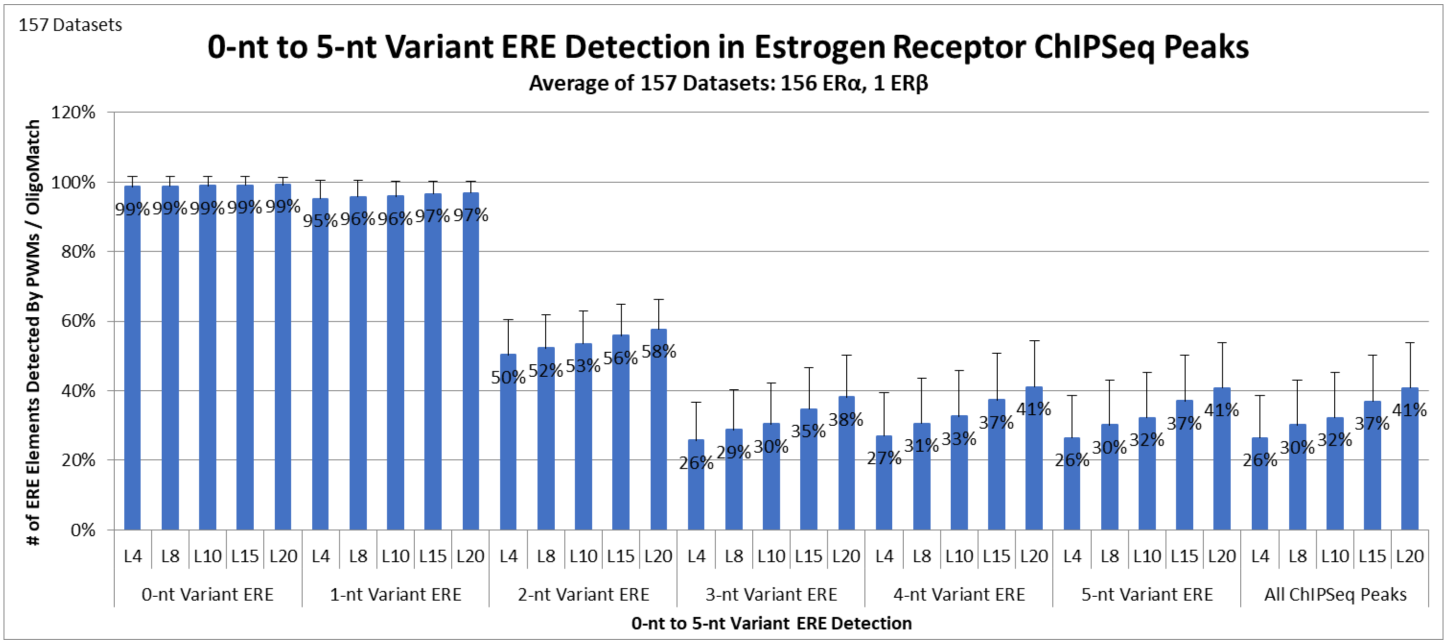

(B) PWMs detection of the <u>ERE DNA motif</u> in ER ChIPSeq Peaks assigned to the ERE DNA element with the least number of variants, defining each peak by a single ERE DNA element (i.e., Unique)

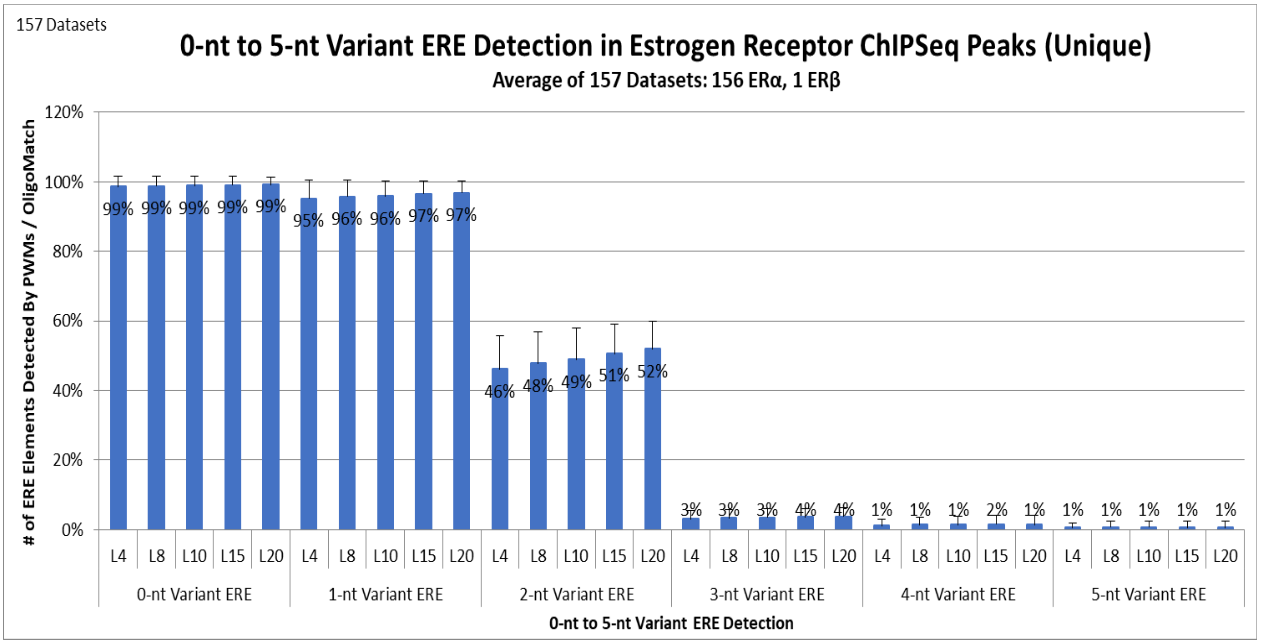

Figure S27. PWMs Detection of the ARE DNA Motif in KR ChIPSeq Peaks

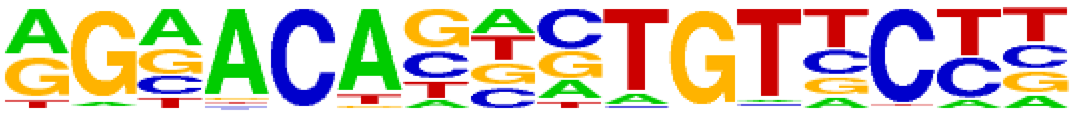

ARE(NR)/LNCAP-AR-ChIPSeq(GSE27824)/Homer

RGRACASNSTGTYCYB

(A) PWMs detection of the ARE DNA motif in KR ChIPSeq Peaks

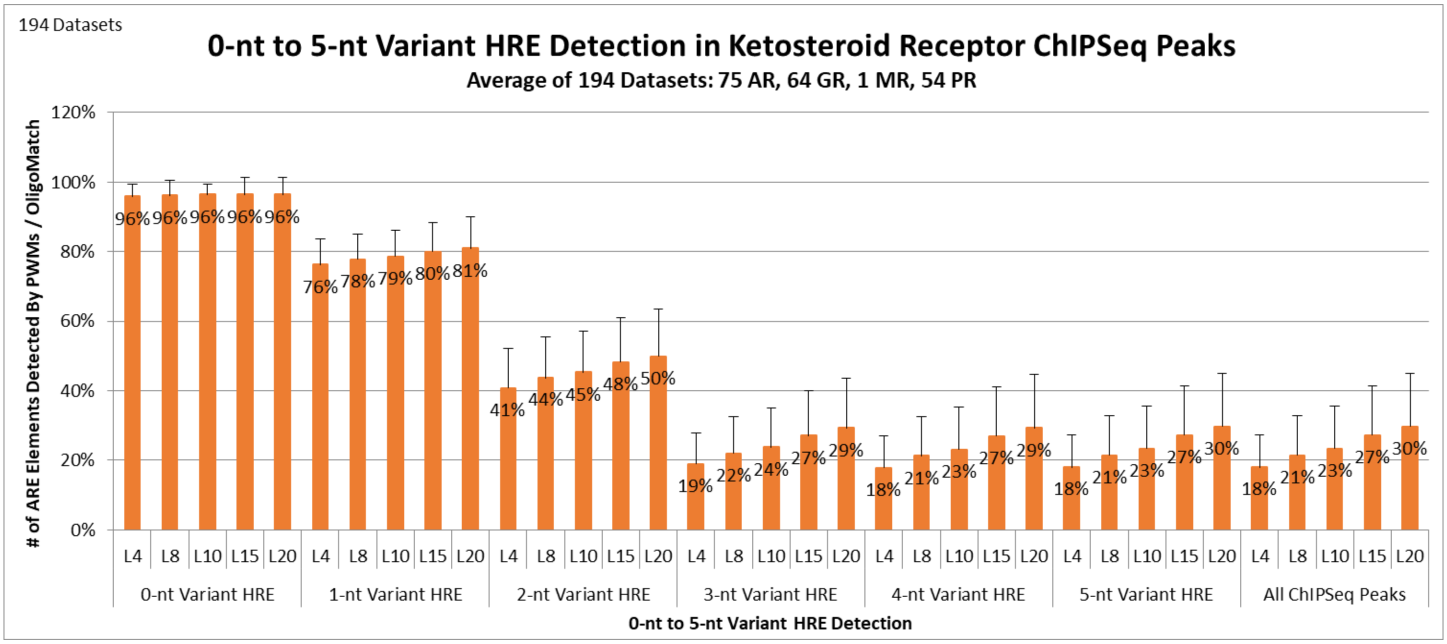

(B) PWMs detection of the ARE DNA motif in KR ChIPSeq Peaks assigned to the HRE DNA element with the least number of variants, defining each peak by a single HRE DNA element (i.e., Unique)

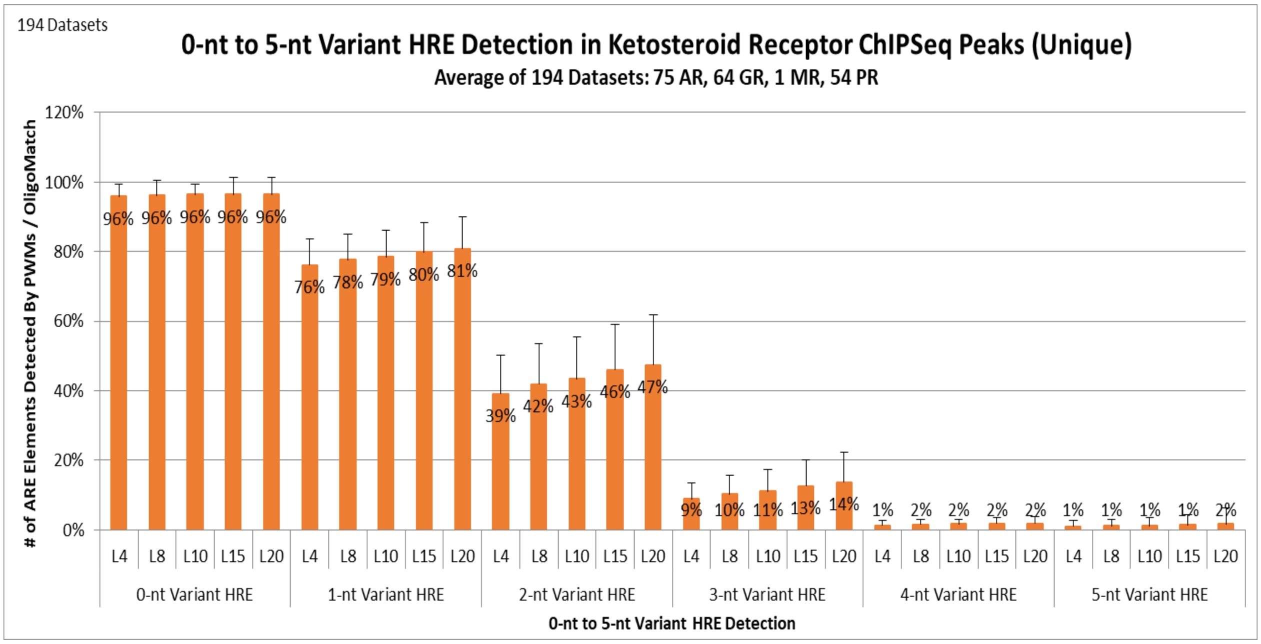

Figure S28. PWMs Detection of the GRE DNA Motif in KR ChIPSeq Peaks

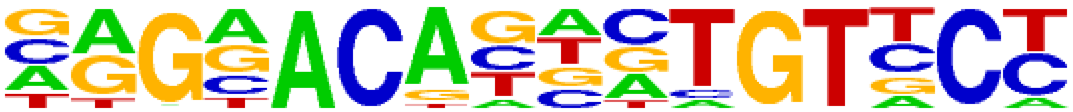

GRE(NR), IR3/A549-GR-ChIPSeq(GSE32465)/Homer

NRGVACABNVTGTYCY

(A) PWMs detection of the GRE DNA motif in KR ChIPSeq Peaks

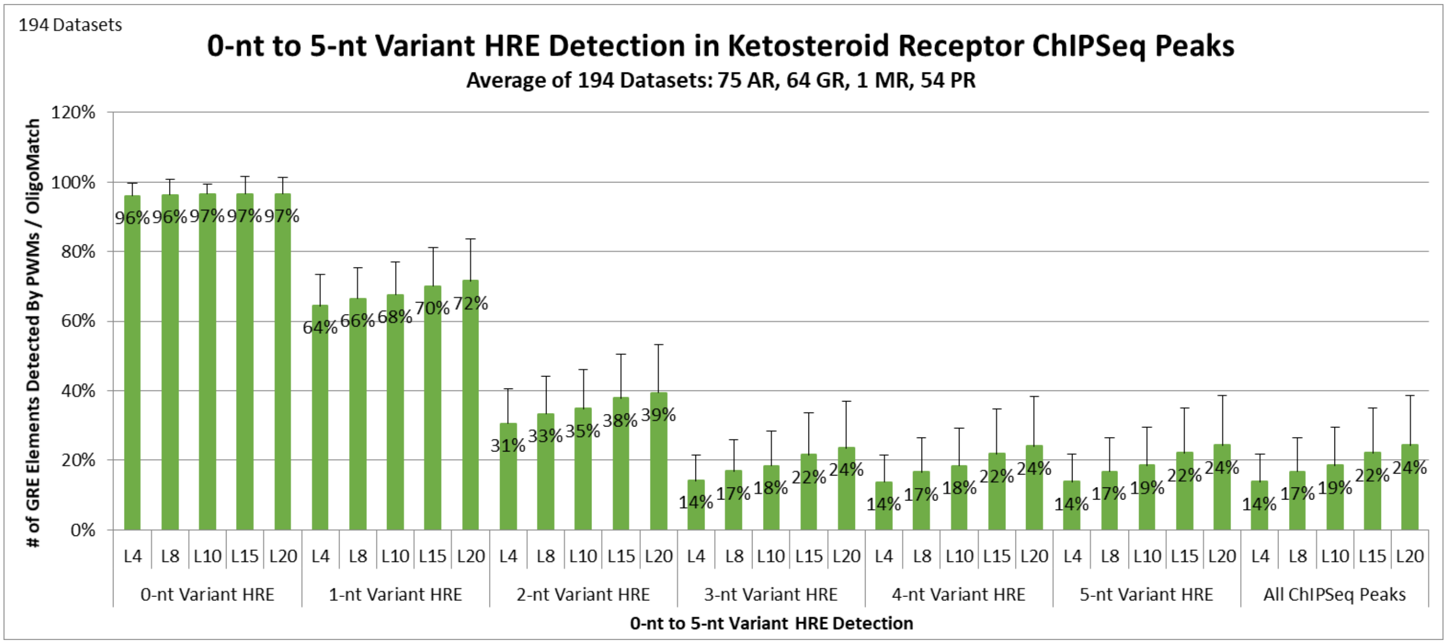

(B) PWMs detection of the GRE DNA motif in KR ChIPSeq Peaks assigned to the HRE DNA element with the least number of variants, defining each peak by a single HRE DNA element (i.e., Unique)

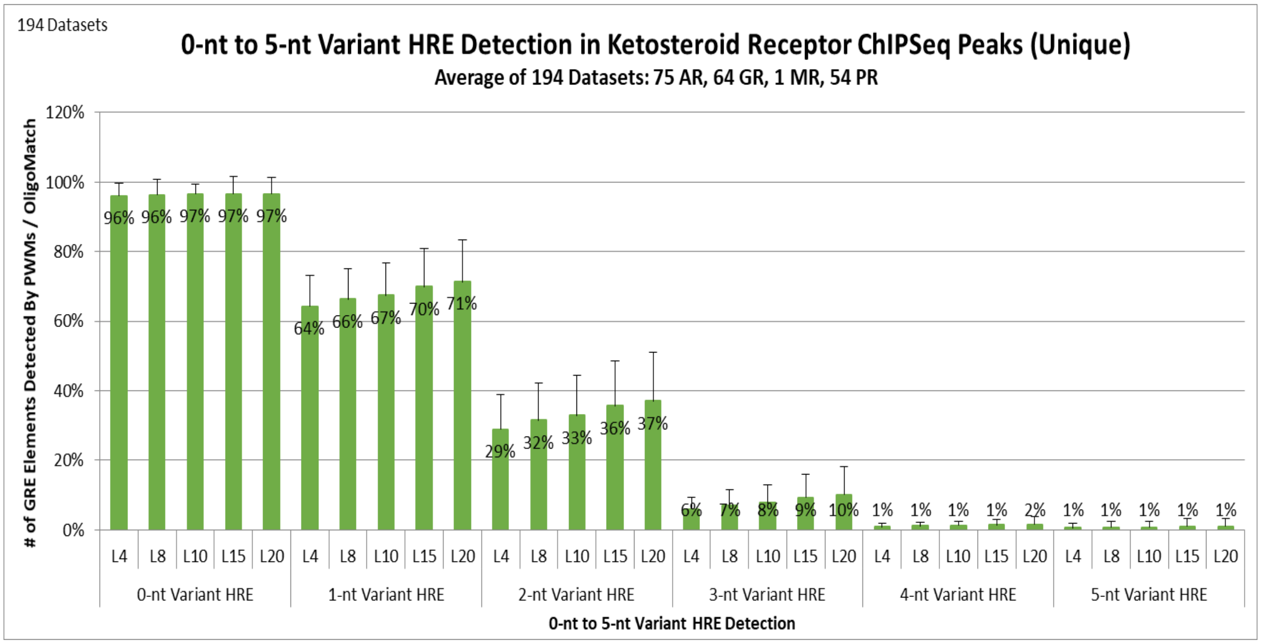

Figure S29. PWMs Detection of the <u>GRE 2 DNA Motif</u> in KR ChIPSeq Peaks

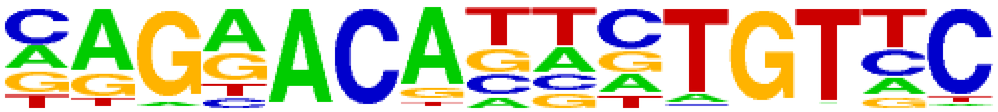

GRE(NR), IR3/RAW264.7-GRE-ChIPSeq(Unpublished)/Homer

VA<u>GRACA</u>KWC<u>TGTYC</u>

(A) PWMs detection of the <u>GRE 2 DNA motif</u> in KR ChIPSeq Peaks

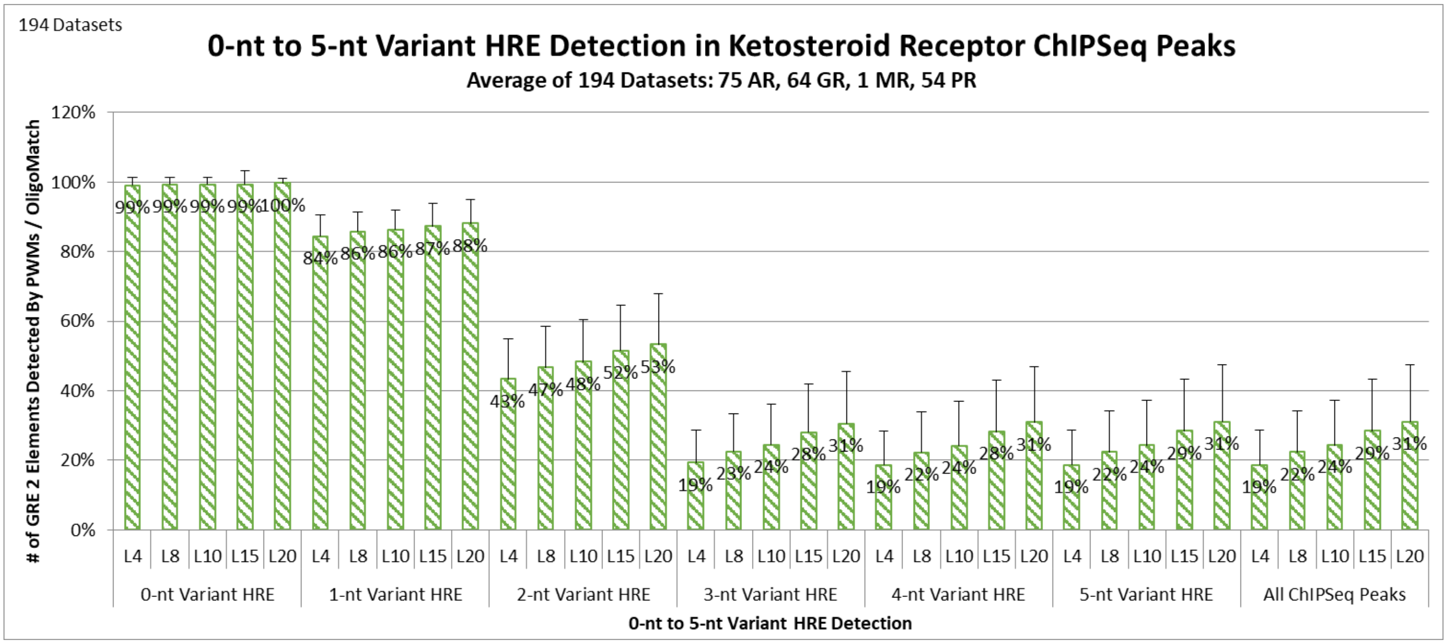

(B) PWMs detection of the <u>GRE 2 DNA motif</u> in KR ChIPSeq Peaks assigned to the HRE DNA element with the least number of variants, defining each peak by a single HRE DNA element (i.e., Unique)

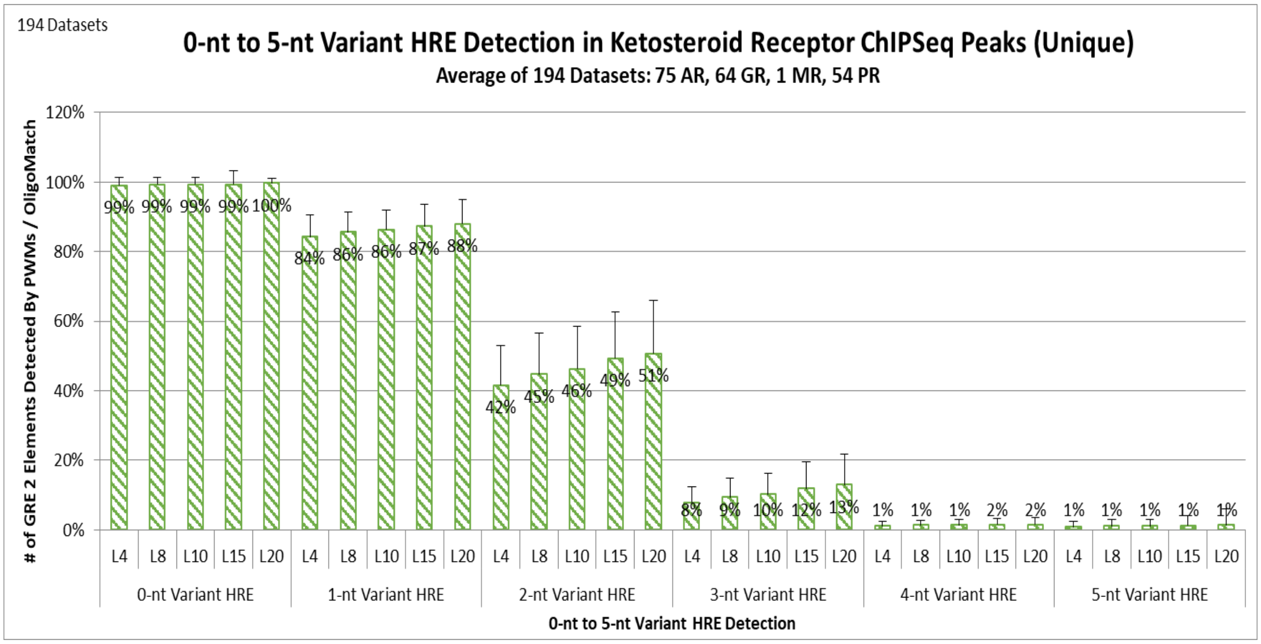

Figure S30. PWMs Detection of the <u>PRE DNA Motif</u> in KR ChIPSeq Peaks

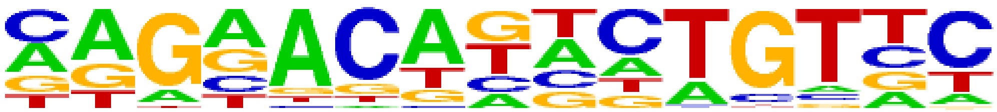

PR(NR)/T47D-PR-ChIPSeq(GSE31130)/Homer

VA<u>GRACA</u>KNC<u>TGTBC</u>

(A) PWMs detection of the <u>PRE DNA motif</u> in KR ChIPSeq Peaks

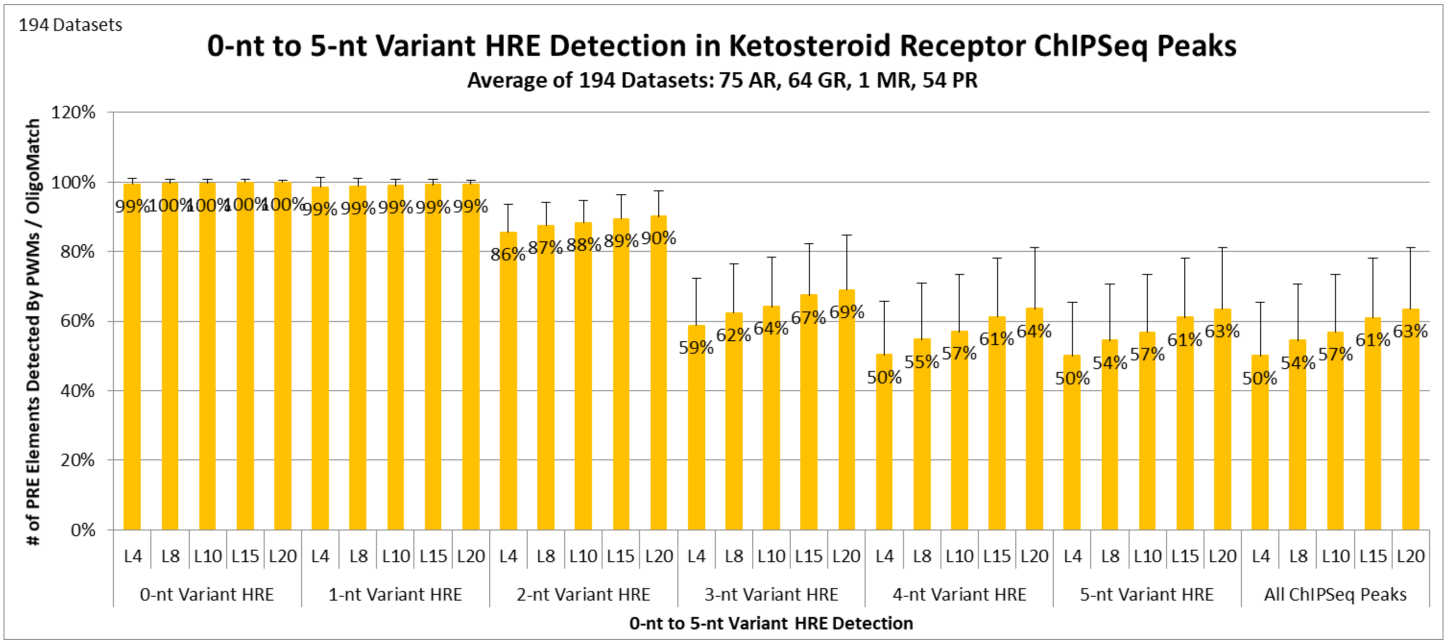

(B) PWMs detection of the <u>PRE DNA motif</u> in KR ChIPSeq Peaks assigned to the HRE DNA element with the least number of variants, defining each peak by a single HRE DNA element (i.e., Unique)

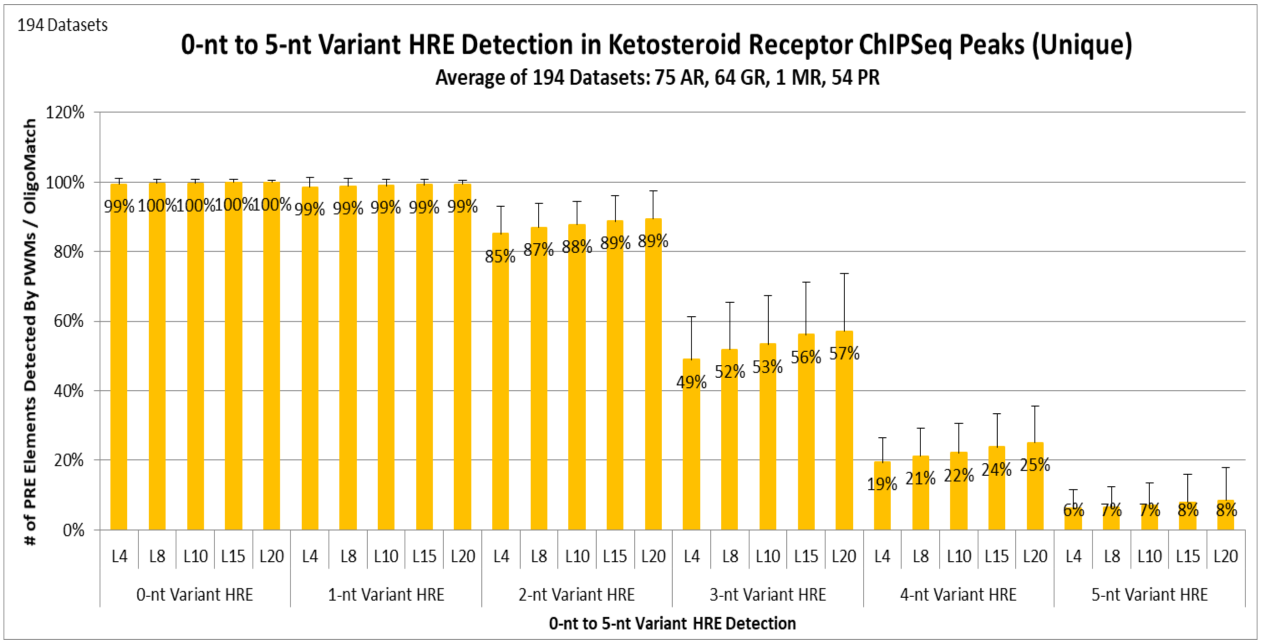

Figure S31. PWMs Detection of the ARE Palindrome Half-Site DNA Motif in KR ChIPSeq Peaks

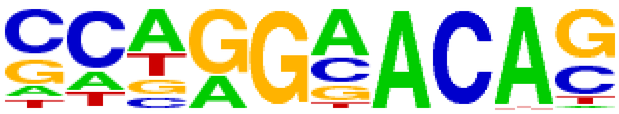

AR-halfsite(NR)/LNCaP-AR-ChIPSeq(GSE27824)/Homer

CCAGGGAACAG

(A) PWMs detection of the ARE Palindrome Half-Site DNA motif in KR ChIPSeq Peaks

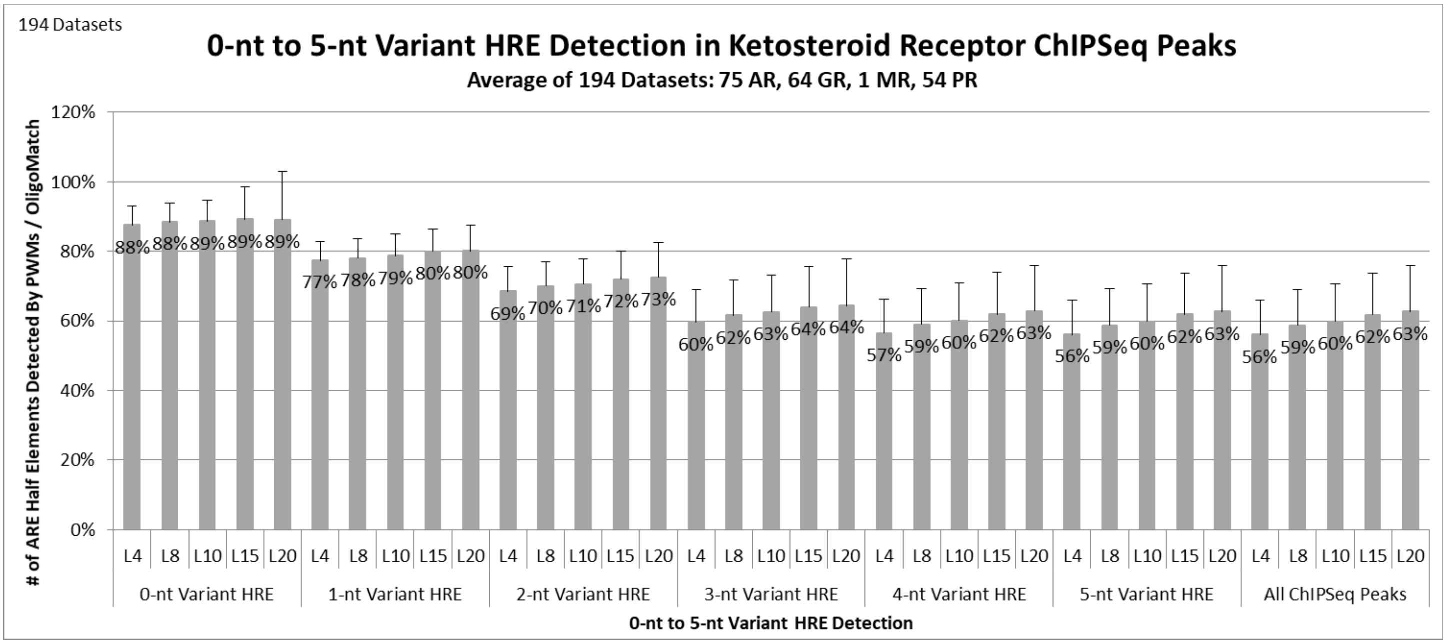

(B) PWMs detection of the ARE Palindrome Half-Site DNA motif in KR ChIPSeq Peaks assigned to the HRE DNA element with the least number of variants, defining each peak by a single HRE DNA element (i.e., Unique)

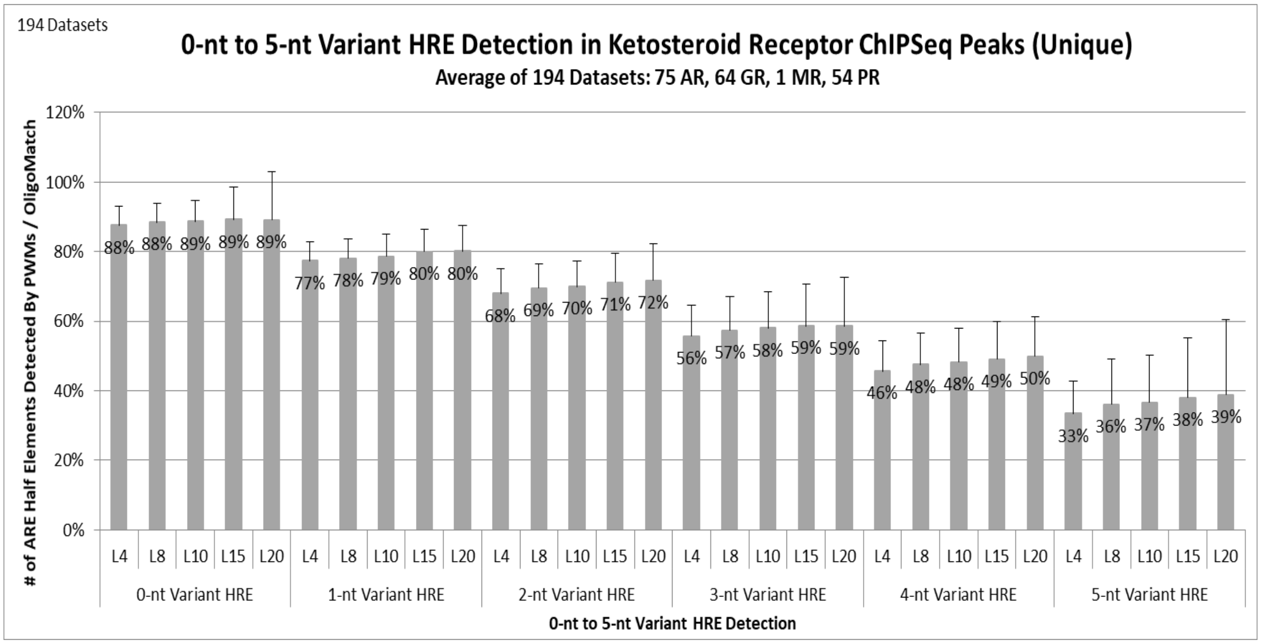

Figure S32. PWMs Detection of the <u>Different HRE Motifs</u> (ARE, GRE, GRE 2, PRE, ARE Half) in KR ChIPSeq Peaks

All Peaks

Unique Peaks

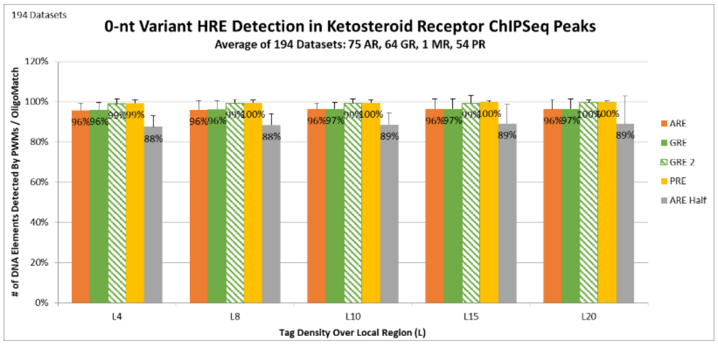

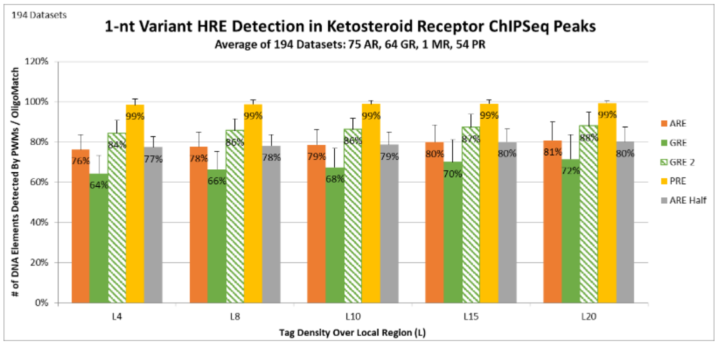

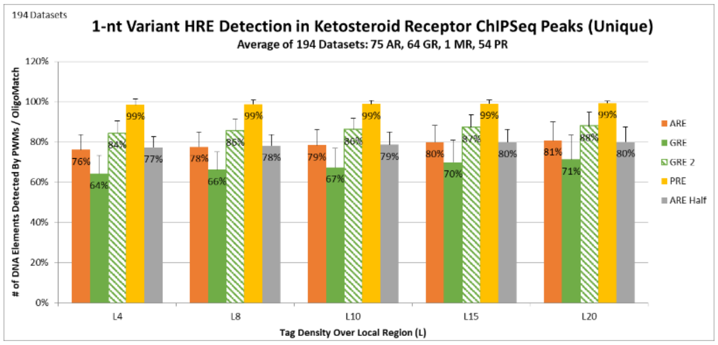

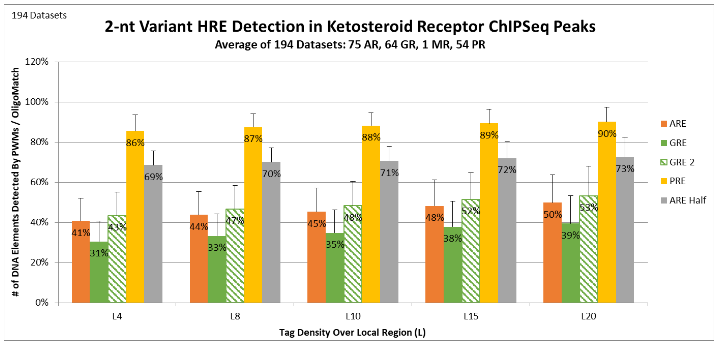

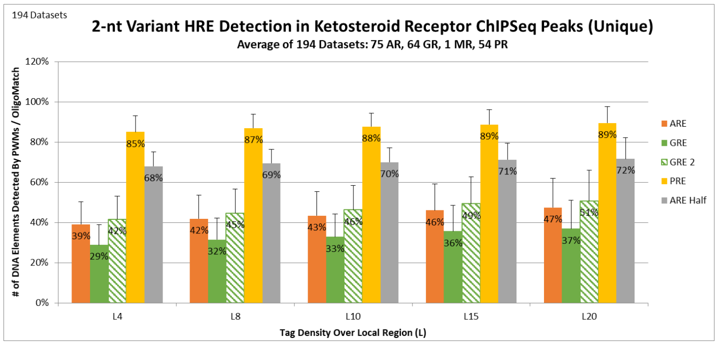

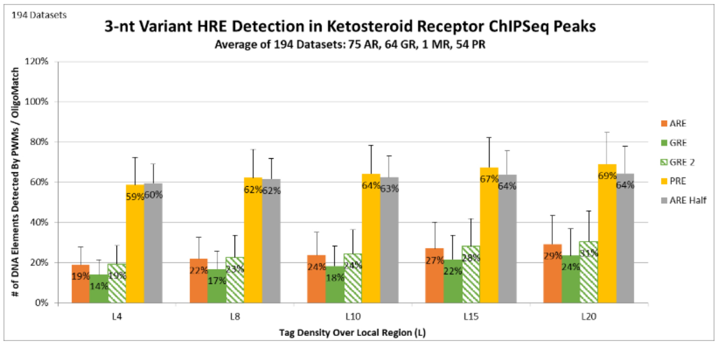

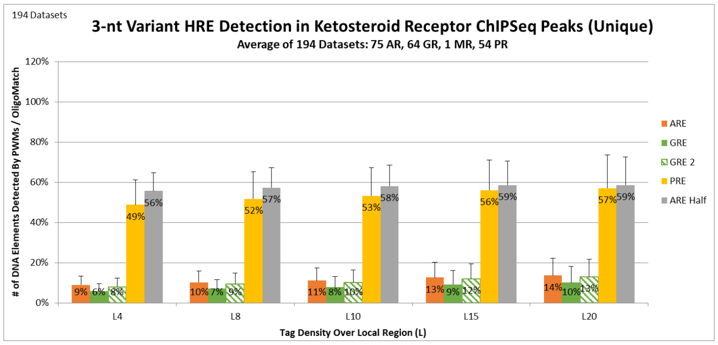

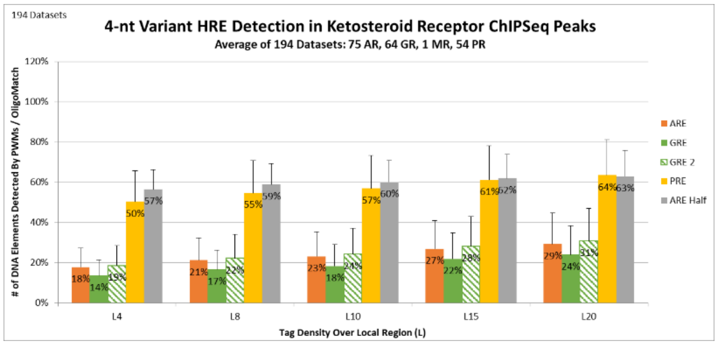

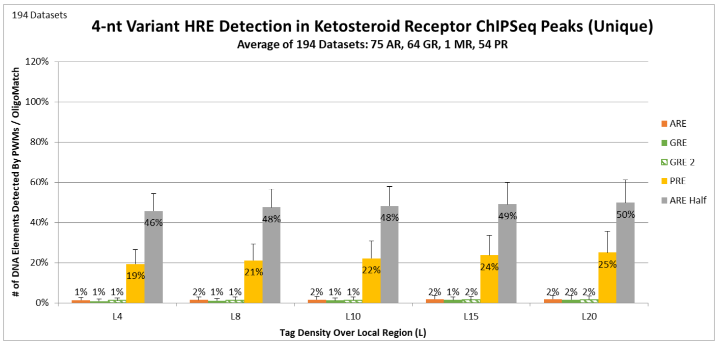

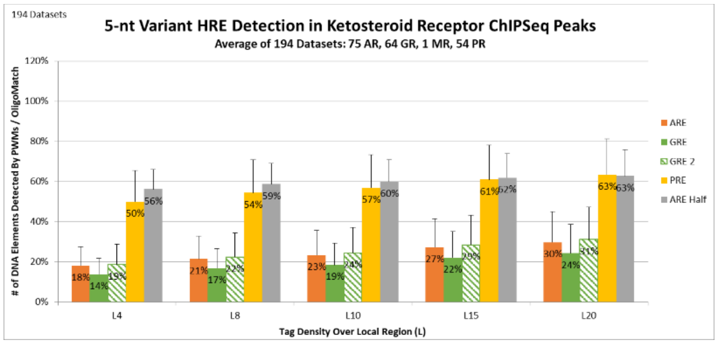
194 Datasets
5-nt Variant HRE Detection in Ketosteroid Receptor ChIPSeq Peaks
Average of 194 Datasets: 75 AR, 64 GR, 1 MR, 54 PR
# of DNA Elements Detected By PWMs / OligoMatch
Tag Density Over Local Region (L)
ARE
GRE
GRE 2
PRE
ARE Half
L4
L8
L10
L15
L20
18% 14% 19% 50% 56%
21% 17% 22% 54% 59%
23% 19% 24% 57% 60%
27% 22% 29% 61% 62%
30% 24% 31% 63% 63%

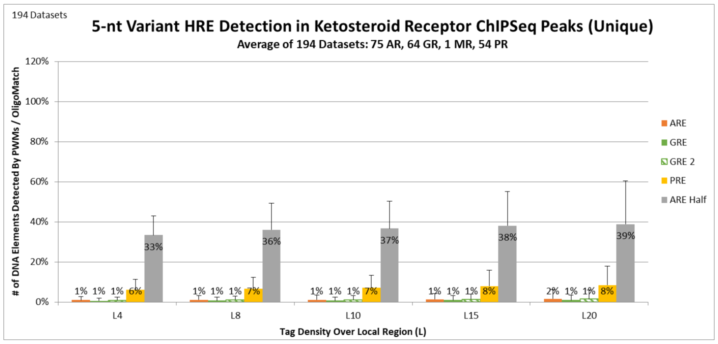
194 Datasets
5-nt Variant HRE Detection in Ketosteroid Receptor ChIPSeq Peaks (Unique)
Average of 194 Datasets: 75 AR, 64 GR, 1 MR, 54 PR
# of DNA Elements Detected By PWMs / OligoMatch
Tag Density Over Local Region (L)
ARE
GRE
GRE 2
PRE
ARE Half
L4
L8
L10
L15
L20
1% 1% 1% 6% 33%
1% 1% 1% 7% 36%
1% 1% 1% 7% 37%
1% 1% 1% 8% 38%
2% 1% 1% 8% 39%

Figure S33-S61 Descriptions: 15-nt ERE and HRE DNA Element Analysis

****All detailed data and statistics associated with every figure are compiled in Data S1****

The analyses in this section were completed by overlapping the location coordinates of each 0-nt to 6-nt variant 15-nt ERE or HRE DNA element in the genome and the location coordinates of the ChIPSeq or ChIPExo peaks in an experiment. The 0-nt variant consensus palindromic 15-nt ERE DNA element (5′-AGGTCAnnnTGACCT-3′) and 0-nt variant consensus palindromic 15-nt HRE DNA element (5′-AGAACAnnnTGTTCT-3′) have twelve (12) primary positions. The 0-nt to 6-nt variant 15-nt ERE or HRE DNA elements include the 1 0-nt variant consensus palindromic DNA element, 36 1-nt variant DNA elements (12 variant positions), 594 2-nt variant DNA elements (66 variant positions), 5,940 3-nt variant DNA elements (220 variant positions), 40,095 4-nt variant DNA elements (495 variant positions), 192,456 5-nt variant DNA elements (792 variant positions), and 673,596 6-nt variant DNA elements (924 variant positions), for a total of 912,718 DNA elements.

Figure S33. 0-nt Variant 15-nt ERE and HRE Identification in ER and KR ChIPSeq Peaks (16 possibilities)
(S/N) analysis of sNR DNA-binding at the 16 possible 15-nt ERE and HRE DNA elements in the genome (i.e., 4-nt possibilities in position 1 and 4-nt possibilities in position 12). sNR DNA-binding was most predominant when an adenine (A) was in position 1 and a thymine (T) was in position 12 of the 15-nt ERE DNA element (5′-AGGTCAnnnTGACCT-3′) for ERs and the 15-nt HRE DNA element (5′-AGAACAnnnTGTTCT-3′) for KRs. This was observed in 157 ER experiments and 194 KR experiments, representing a wide variety of mouse tissues and human cell lines, and across multiple peak selection criteria (L4-L20). Thus, these 15-nt ERE and HRE DNA elements (i.e., adenine (A) in position 1 and thymine (T) in position 12) represent the 0-nt variant consensus palindromic 15-nt ERE and HRE DNA elements for ERs and KRs.

Figure S34. Number of 0-nt to 6-nt Variant 15-nt ERE and HRE DNA Elements in the Mouse and Human Genome
(Top Table) The number of 0-nt to 5-nt variants of the 13-nt ERE and HRE consensus palindromic DNA element on the positive/sense strand in the mouse (mm10) and human (hg19) genome. Here the 13-nt ERE is the estrogen response element (5′-GGTCAnnnTGACC-3′) and the 13-nt HRE is the hormone response element (5′-GAACAnnnTGTTC-3′). The 0-nt to 5-nt variant 13-nt ERE and HRE DNA elements include the 1 0-nt variant consensus palindromic DNA element, 30 1-nt variant DNA elements (10 variant positions), 405 2-nt variant DNA elements (45 variant positions), 3,240 3-nt variant DNA elements (120 variant positions), 17,010 4-nt variant DNA elements (210 variant positions), and 61,236 5-nt variant DNA elements (252 variant positions), for a total of 81,922 DNA elements. The population count of each of the 81,922 0-nt to 5-nt variant 13-nt ERE and HRE DNA elements in the mouse (mm10) and human (hg19) genome can be found in **Table S55-S58**.

(Bottom Table) The number of 0-nt to 6-nt variants of the 15-nt ERE and HRE consensus palindromic DNA element on the positive/sense strand in the mouse (mm10) and human (hg19) genome. Here the 15-nt ERE is the estrogen response element (5′-AGGTCAnnnTGACCT-3′) and the 15-nt HRE is the hormone response element (5′-AGAACAnnnTGTTCT-3′). The 0-nt to 6-nt variant 15-nt ERE and HRE DNA elements include the 1 0-nt variant consensus palindromic DNA element, 36 1-nt variant DNA elements (12 variant positions), 594 2-nt variant DNA elements (66 variant positions), 5,940 3-nt variant DNA elements (220 variant positions), 40,095 4-nt variant DNA elements (495 variant positions), 192,456 5-nt variant DNA elements (792 variant positions), and 673,596 6-nt variant DNA elements (924 variant positions), for a total of 912,718 DNA elements. The population count of each of the 912,718 0-nt to 6-nt variant 15-nt ERE and HRE DNA elements in the mouse (mm10) and human (hg19) genome can be found in **Table S59-S62**.

*****

Figure S35. Inversion Symmetry of sNR DNA-Binding at 1-nt Variant EREs and HREs in the Genome (%)
(A) Average distribution of 1-nt variant EREs in ER experiments (156 ERα and 1 ERβ) at the thirty-six (36) 1-nt variant EREs in the genome (the total number of 1-nt variant EREs contained within a ChIPSeq experiment =100%) **(B)** Average distribution of 1-nt variant HREs in 193 KR experiments (75 AR, 64 GR, 54 PR) at the thirty-six (36) 1-nt variant HREs in the genome (the total number of 1-nt variant HREs contained within a ChIPSeq experiment =100%). These thirty-six (36) 1-nt variant EREs or HREs are defined by twelve (12) variant positions (position 1 through position 6, followed by their reverse-complements).

Figure S36. Inversion Symmetry of sNR DNA-Binding at 2-nt Variant EREs and HREs in the Genome (%)
(A) Average distribution of 2-nt variant EREs in ER experiments (156 ERα and 1 ERβ) at the five hundred and ninety-four (594) 2-nt variant EREs in the genome (the total number of 2-nt variant EREs contained within a ChIPSeq experiment =100%) **(B)** Average distribution of 2-nt variant HREs in 193 KR experiments (75 AR, 64 GR, 54 PR) at the five hundred and ninety-four (594) 2-nt variant HREs in the genome (the total number of 2-nt variant HREs contained within a ChIPSeq experiment =100%). These five hundred and ninety-four (594) 2-nt variant EREs or HREs are defined by sixty-six (66) variant positions. Six (6) of those are when the variants are in the palindromic position pairs (PP): 1-12, 2-11, 3-10, 4-9, 5-8, 6-7. The remaining sixty (60) are symmetrically split into 30 groups and their reverse-complements.

*****

Figure S37. (S/N) analysis of 0-nt to 6-nt Variant EREs in ER ChIPSeq Peaks (displayed by number of variants)
(S/N) analysis of ER DNA-binding at 0-nt to 6-nt variant EREs in 157 ER experiments (156 ERα and 1 ERβ). Experiments are ordered by the experiment with highest (S/N) value at the 0-nt variant consensus palindromic ERE DNA element in the genome. The signal-to-noise ratio (S/N) is the absolute number of times a 0-nt to 6-nt variant DNA element occurs within an experiment [defined by the total number of peaks in the experiment and the peak length] compared to the random frequency expectation of that DNA element occurring in the genome (i.e., the probability that any 12-nt DNA element that has a maximum possibility of 4 nucleotides at each of the 12 primary positions will occur is once every 16,777,216 nucleotides (4^{12}) at random occurrence). Thus, the expected noise is calculated by multiplying the total number of peaks in the experiment by the length of the peaks over 4^{12}. For (S/N) analysis of 0-nt to 6-nt variant DNA elements (displayed by the number of variants in the DNA element), the expected noise is multiplied by the number of DNA elements in each group: 1 0-nt variant consensus palindromic DNA element, 36 1-nt variant DNA elements, 594 2-nt variant DNA elements, 5,940 3-nt variant DNA elements, 40,095 4-nt variant DNA elements, 192,456 5-nt variant DNA elements, and 673,596 6-nt variant DNA elements, for a total of 912,718 0-nt to 6-nt variant DNA elements. **Note**: the relative enhancement values of the sNR DNA-binding signals are scale invariant (i.e., the relative ratios between the 0-nt to 6-nt variant groups are constant). Y-axis =the natural log of the (S/N) value (i.e., random =0 and 0 =log(1)).

Figure S38. (S/N) analysis of 0-nt to 6-nt Variant HREs in KR ChIPSeq Peaks (displayed by number of variants)
(S/N) analysis of ER DNA-binding at 0-nt to 6-nt variant HREs in 194 KR experiments (75 AR, 64 GR, 1 MR, 54 PR). Experiments are grouped by KR, and then ordered by the experiment with highest (S/N) value at the 0-nt variant consensus palindromic HRE DNA element in the genome. The signal-to-noise ratio (S/N) is the absolute number of times a 0-nt to 6-nt variant DNA element occurs within an experiment [defined by the total number of peaks in the experiment and the peak length] compared to the random frequency expectation of that DNA element occurring in the genome (i.e., the probability that any 12-nt DNA element that has a maximum possibility of 4 nucleotides at each of the 12 primary positions will occur is once every 16,777,216 nucleotides (4^{12}) at random occurrence). Thus, the expected noise is calculated by multiplying the total number of peaks in the experiment by the length of the peaks over 4^{12}. For (S/N) analysis of 0-nt to 6-nt variant DNA elements (displayed by the number of variants in the DNA element), the expected noise is multiplied by the number of DNA elements in each group: 1 0-nt variant consensus palindromic DNA element, 36 1-nt variant DNA elements, 594 2-nt variant DNA elements, 5,940 3-nt variant DNA elements, 40,095 4-nt variant DNA elements, 192,456 5-nt variant DNA elements, and 673,596 6-nt variant DNA elements, for a total of 912,718 0-nt to 6-nt variant DNA elements. **Note**: the relative enhancement values of the sNR DNA-binding signals are scale invariant (i.e., the relative ratios between the 0-nt to 6-nt variant groups are constant). Y-axis =the natural log of the (S/N) value (i.e., random =0 and 0 =log(1)).

*****

Figure S39. 924 Half-Site Groups Symmetrically Split Into 452 Groups and Their Reverse-Complements (and 20 Palindromes)
(Column A) Categorizing the 673,596 6-nt variant DNA elements into 924 half-site groups, defined by the six (6) positions that are fixed/not varied. The 924 half-site groups are symmetrically split into a set of 452 groups and their 452 reverse-complements, plus 20 innate palindromes. **(Column B)** These 452 groups further split into three distinct sub-groups (zero vacancies, one vacancy, two vacancies) plus the 20 innate palindromes (three vacancies) depending on how many reverse-complement vacancies are in the DNA element (i.e., the information for which nucleotide occupies each of the twelve (12) primary positions of the 0-nt variant consensus palindromic DNA element is missing/replaced by variants/vacant in the position and its reverse-complement position (its palindromic position pair)). Of these 472 groups, thirty-two (32) have zero vacancies, two hundred and forty (240) have one vacancy, one hundred and eighty (180) have two vacancies, and twenty (20) have three vacancies. **(Column C)** The reverse-complement vacancy position ID indicates which of the six (6) palindromic position pairs (i.e., 1-12, 2-11, 3-10, 4-9, 5-8, 6-7) are missing/replaced by variants/vacant. **(Column B)** The number of reverse-complement vacancies equals the number of reverse-complement double occupants in the DNA element (i.e., the information for which nucleotide occupies each of the twelve (12) primary positions of the 0-nt variant consensus palindromic DNA element is occupied in the position and its reverse-complement position (its palindromic position pair)). **(Column D)** The reverse-complement double occupant position ID indicates which of the six (6) palindromic position pairs (i.e., 1-12, 2-11, 3-10, 4-9, 5-8, 6-7) are doubly occupied. **(Column E)** The positions that are replaced by variants/not fixed for 6-nt variant DNA elements, and may be replaced by variants for 0-nt to 5-nt variant DNA elements.

Figure S40. (S/N) analysis of 0-nt to 6-nt Variant EREs in ER ChIPSeq Peaks (displayed by 924 half-site groups)
(S/N) analysis of 0-nt to 6-nt variant EREs (displayed by the 924 half-site groups) in ER (WT-E2-1hr) [76,163 peaks, 146-nt peak length] ChIPSeq peaks. X-axis order =Reverse-Complement Vacancy Position ID 4-9 > 1-12 > 2-11 > 6-7 > 5-8 > 3-10. See **Table S25** for x-axis details. This DNA-binding profile was observed in 157 ER experiments, representing a wide variety of mouse tissues and human cell lines, and across multiple peak selection criteria (L4-L20) **(Table S27)**. See **Table S68-S71** for step-by-step instructions of the data analysis from peak selection to (S/N) analysis of ER DNA-binding at 912,718 0-nt to 6-nt variant 15-nt ERE DNA elements in the genome, displayed by the 924 half-site groups.

Figure S41. (S/N) analysis of 0-nt to 6-nt Variant HREs in AR ChIPSeq Peaks (displayed by 924 half-site groups)
(S/N) analysis of 0-nt to 6-nt variant HREs (displayed by the 924 half-site groups) in AR (AR-wt1) [49,859 peaks, 136-nt peak length] ChIPSeq peaks. X-axis order =Reverse-Complement Vacancy Position ID 3-10 > 1-12 > 6-7 > 2-11 > 5-8 > 4-9. See **Table S26** for x-axis details. This DNA-binding profile was observed in 194 KR experiments, representing a wide variety of mouse tissues and human cell lines, and across multiple peak selection criteria (L4-L20) **(Table S28)**.

Figure S42. (S/N) analysis of 0-nt to 6-nt Variant HREs in GR ChIPSeq Peaks (displayed by 924 half-site groups)
(S/N) analysis of 0-nt to 6-nt variant HREs (displayed by the 924 half-site groups) in GR (Liver-GR-WT-pred-6am-2) [23,742 peaks, 142-nt peak length] ChIPSeq peaks. X-axis order =Reverse-Complement Vacancy Position ID 3-10 > 1-12 > 6-7 > 2-11 > 5-8 > 4-9. See **Table S26** for x-axis details. This DNA-binding profile was observed in 194 KR experiments, representing a wide variety of mouse tissues and human cell lines, and across multiple peak selection criteria (L4-L20) **(Table S28)**.

Figure S43. (S/N) analysis of 0-nt to 6-nt Variant HREs in PR ChIPSeq Peaks (displayed by 924 half-site groups)
(S/N) analysis of 0-nt to 6-nt variant HREs (displayed by the 924 half-site groups) in PR (Uterus-PGR-P4) [66,545 peaks, 154-nt peak length] ChIPSeq peaks. X-axis order =Reverse-Complement Vacancy Position ID 3-10 > 1-12 > 6-7 > 2-11 > 5-8 > 4-9. See **Table S26** for x-axis details. This DNA-binding profile was observed in 194 KR experiments, representing a wide variety of mouse tissues and human cell lines, and across multiple peak selection criteria (L4-L20) **(Table S28)**.

*****

Figure S44. (S/N) analysis of 1-nt Variant EREs in ER ChIPSeq Peaks (displayed by variant position)
(S/N) analysis of 1-nt variant EREs in ER (WT-E2-1hr) ChIPSeq peaks [76,163 peaks, 146-nt peak length].
Displayed by the 12 variant positions of the 36 1-nt variant EREs. This DNA-binding profile was observed in 157 ER experiments, representing a wide variety of mouse tissues and human cell lines, and across multiple peak selection criteria (L4-L20) **(Table S29)**.

Figure S45. (S/N) analysis of 2-nt Variant EREs in ER ChIPSeq Peaks (displayed by variant position)
(S/N) analysis of 2-nt variant EREs in ER (WT-E2-1hr) ChIPSeq peaks [76,163 peaks, 146-nt peak length].
Displayed by the 66 variant positions of the 594 2-nt variant EREs. 6 of the 66 variant positions are the six (6) palindromic position pairs: 1-12, 2-11, 3-10, 4-9, 5-8, 6-7 (PP, dark red solid bars). 30 of the 66 variant positions are same-side variants (i.e., the variants do not crossover the 3-nt spacer) (blue solid bars). 30 of the 66 variant positions are crossover variants (i.e., the variants do crossover the 3-nt spacer) (blue diagonal stripped bars). Each variant position is immediately followed by its reverse-complement variant position on the x-axis, demonstrating that transcription factor binding in the genome follows inversion symmetry (i.e., the number of transcription factor binding events at a particular DNA element in the genome is equivalent to the number of transcription factor binding events at its reverse-complement DNA element in the genome). This DNA-binding profile was observed in 157 ER experiments, representing a wide variety of mouse tissues and human cell lines, and across multiple peak selection criteria (L4-L20) **(Table S30)**.

Figure S46. (S/N) analysis of 3-nt Variant EREs in ER ChIPSeq Peaks (displayed by variant position)
(S/N) analysis of 3-nt variant EREs in ER (WT-E2-1hr) ChIPSeq peaks [76,163 peaks, 146-nt peak length].
Displayed by the 220 variant positions of the 5,940 3-nt variant EREs. 40 of the 220 variant positions are same-side variants (i.e., the variants do not crossover the 3-nt spacer) (blue solid bars). 180 of the 220 variant positions are crossover variants (i.e., the variants do crossover the 3-nt spacer) (blue diagonal stripped bars). Each variant position is immediately followed by its reverse-complement variant position on the x-axis, demonstrating that transcription factor binding in the genome follows inversion symmetry (i.e., the number of transcription factor binding events at a particular DNA element in the genome is equivalent to the number of transcription factor binding events at its reverse-complement DNA element in the genome). This DNA-binding profile was observed in 157 ER experiments, representing a wide variety of mouse tissues and human cell lines, and across multiple peak selection criteria (L4-L20) **(Table S31)**.

Figure S47. (S/N) analysis of 4-nt Variant EREs in ER ChIPSeq Peaks (displayed by variant position)
(S/N) analysis of 4-nt variant EREs in ER (WT-E2-1hr) ChIPSeq peaks [76,163 peaks, 146-nt peak length].
Displayed by the 495 variant positions of the 40,095 4-nt variant EREs. 15 of the 495 variant positions are the five (5) palindromic position pairs: 2-4-9-11, 4-6-7-9, 4-5-8-9, 3-4-9-10, 2-6-7-11, 2-5-8-11, 2-3-10-11, 5-6-7-8, 3-6-7-10, 3-5-8-10, 1-4-9-12, 1-2-11-12, 1-6-7-12, 1-5-8-12, 1-3-10-12 (PP, dark red solid bars). 30 of the 495 variant positions are same-side variants (i.e., the variants do not crossover the 3-nt spacer) (blue solid bars). 450 of the 495 variant positions are crossover variants (i.e., the variants do crossover the 3-nt spacer) (blue diagonal stripped bars). Each variant position is immediately followed by its reverse-complement variant position on the x-axis, demonstrating that transcription factor binding in the genome follows inversion symmetry (i.e., the number of transcription factor binding events at a particular DNA element in the genome is equivalent to the number of transcription factor binding events at its reverse-complement DNA element in the genome). This DNA-binding profile was observed in 157 ER experiments, representing a wide variety of mouse tissues and human cell lines, and across multiple peak selection criteria (L4-L20) **(Table S32)**.

Figure S48. (S/N) analysis of 5-nt Variant EREs in ER ChIPSeq Peaks (displayed by variant position)
(S/N) analysis of 5-nt variant EREs in ER (WT-E2-1hr) ChIPSeq peaks [76,163 peaks, 146-nt peak length].
Displayed by the 792 variant positions of the 192,456 5-nt variant EREs. 12 of the 792 variant positions are same-side variants (i.e., the variants do not crossover the 3-nt spacer) (blue solid bars). 780 of the 792 variant positions are crossover variants (i.e., the variants do crossover the 3-nt spacer) (blue diagonal stripped bars). Each variant position is immediately followed by its reverse-complement variant position on the x-axis, demonstrating that transcription factor binding in the genome follows inversion symmetry (i.e., the

number of transcription factor binding events at a particular DNA element in the genome is equivalent to the number of transcription factor binding events at its reverse-complement DNA element in the genome). This DNA-binding profile was observed in 157 ER experiments, representing a wide variety of mouse tissues and human cell lines, and across multiple peak selection criteria (L4-L20) **(Table S33)**.

Figure S49. (S/N) analysis of 6-nt Variant EREs in ER ChIPSeq Peaks (displayed by variant position)
(S/N) analysis of 6-nt variant EREs in ER (WT-E2-1hr) ChIPSeq peaks [76,163 peaks, 146-nt peak length].
Displayed by the 924 variant positions of the 673,596 6-nt variant EREs. 20 of the 924 variant positions are the five (5) palindromic position pairs: 1-4-6-7-9-12, 2-3-4-9-10-11, 1-3-4-9-10-12, 1-2-4-9-11-12, 2-4-6-7-9-11, 1-4-5-8-9-12, 2-4-5-8-9-11, 3-4-6-7-9-10, 1-2-6-7-11-12, 4-5-6-7-8-9, 1-2-5-8-11-12, 1-3-6-7-10-12, 1-2-3-10-11-12, 3-4-5-8-9-10, 1-5-6-7-8-12, 2-3-5-8-10-11, 2-3-6-7-10-11, 1-3-5-8-10-12, 3-5-6-7-8-10, 2-5-6-7-8-11 (PP, dark red solid bars). 2 of the 924 variant positions are same-side variants (i.e., the variants do not crossover the 3-nt spacer) (blue solid bars). 922 of the 924 variant positions are crossover variants (i.e., the variants do crossover the 3-nt spacer) (blue diagonal stripped bars). Each variant position is immediately followed by its reverse-complement variant position on the x-axis, demonstrating that transcription factor binding in the genome follows inversion symmetry (i.e., the number of transcription factor binding events at a particular DNA element in the genome is equivalent to the number of transcription factor binding events at its reverse-complement DNA element in the genome). This DNA-binding profile was observed in 157 ER experiments, representing a wide variety of mouse tissues and human cell lines, and across multiple peak selection criteria (L4-L20) **(Table S34)**.

*****

Figure S50. (S/N) analysis of 1-nt Variant HREs in KR ChIPSeq Peaks (displayed by variant position)
(S/N) analysis of 1-nt variant HREs in AR (AR-wt1) ChIPSeq peaks [49,859 peaks, 136-nt peak length].
Displayed by the 12 variant positions of the 36 1-nt variant HREs. This DNA-binding profile was observed in 194 KR experiments, representing a wide variety of mouse tissues and human cell lines, and across multiple peak selection criteria (L4-L20) **(Table S35)**.

Figure S51. (S/N) analysis of 2-nt Variant HREs in KR ChIPSeq Peaks (displayed by variant position)
(S/N) analysis of 2-nt variant HREs in AR (AR-wt1) ChIPSeq peaks [49,859 peaks, 136-nt peak length].
Displayed by the 66 variant positions of the 594 2-nt variant HREs. 6 of the 66 variant positions are the six (6) palindromic position pairs: 1-12, 2-11, 3-10, 4-9, 5-8, 6-7 (PP, dark red solid bars). 30 of the 66 variant positions are same-side variants (i.e., the variants do not crossover the 3-nt spacer) (orange solid bars). 30 of the 66 variant positions are crossover variants (i.e., the variants do crossover the 3-nt spacer) (orange diagonal stripped bars). Each variant position is immediately followed by its reverse-complement variant position on the x-axis, demonstrating that transcription factor binding in the genome follows inversion symmetry (i.e., the number of transcription factor binding events at a particular DNA element in the genome is equivalent to the number of transcription factor binding events at its reverse-complement DNA element in the genome). This DNA-binding profile was observed in 194 KR experiments, representing a wide variety of mouse tissues and human cell lines, and across multiple peak selection criteria (L4-L20) **(Table S36)**.

Figure S52. (S/N) analysis of 3-nt Variant HREs in KR ChIPSeq Peaks (displayed by variant position)
(S/N) analysis of 3-nt variant HREs in AR (AR-wt1) ChIPSeq peaks [49,859 peaks, 136-nt peak length].
Displayed by the 220 variant positions of the 5,940 3-nt variant HREs. 40 of the 220 variant positions are same-side variants (i.e., the variants do not crossover the 3-nt spacer) (orange solid bars). 180 of the 220 variant positions are crossover variants (i.e., the variants do crossover the 3-nt spacer) (orange diagonal stripped bars). Each variant position is immediately followed by its reverse-complement variant position on the x-axis, demonstrating that transcription factor binding in the genome follows inversion symmetry (i.e., the number of transcription factor binding events at a particular DNA element in the genome is equivalent to the number of transcription factor binding events at its reverse-complement DNA element in the genome). This DNA-binding profile was observed in 194 KR experiments, representing a wide variety of mouse tissues and human cell lines, and across multiple peak selection criteria (L4-L20) **(Table S37)**.

Figure S53. (S/N) analysis of 4-nt Variant HREs in KR ChIPSeq Peaks (displayed by variant position)
(S/N) analysis of 4-nt variant HREs in AR (AR-wt1) ChIPSeq peaks [49,859 peaks, 136-nt peak length].
Displayed by the 495 variant positions of the 40,095 4-nt variant HREs. 15 of the 495 variant positions are the five (5) palindromic position pairs: 3-6-7-10, 2-3-10-11, 3-5-8-10, 3-4-9-10, 2-6-7-11, 5-6-7-8, 4-6-7-9, 2-5-8-11, 2-4-9-11, 4-5-8-9, 1-3-10-12, 1-6-7-12, 1-2-11-12, 1-5-8-12, 1-4-9-12 (PP, dark red solid bars). 30 of the 495 variant positions are same-side variants (i.e., the variants do not crossover the 3-nt spacer) (orange solid bars). 450 of the 495 variant positions are crossover variants (i.e., the variants do crossover the 3-nt spacer) (orange diagonal stripped bars). Each variant position is immediately followed by its reverse-complement variant position on the x-axis, demonstrating that transcription factor binding in the genome follows inversion symmetry (i.e., the number of transcription factor binding events at a particular DNA element in the genome is equivalent to the number of transcription factor binding events at its reverse-complement DNA element in the genome). This DNA-binding profile was observed in 194 KR experiments, representing a wide variety of mouse tissues and human cell lines, and across multiple peak selection criteria (L4-L20) **(Table S38)**.

Figure S54. (S/N) analysis of 5-nt Variant HREs in KR ChIPSeq Peaks (displayed by variant position)
(S/N) analysis of 5-nt variant HREs in AR (AR-wt1) ChIPSeq peaks [49,859 peaks, 136-nt peak length].
Displayed by the 792 variant positions of the 192,456 5-nt variant HREs. 12 of the 792 variant positions are same-side variants (i.e., the variants do not crossover the 3-nt spacer) (orange solid bars). 780 of the 792 variant positions are crossover variants (i.e., the variants do crossover the 3-nt spacer) (orange diagonal stripped bars). Each variant position is immediately followed by its reverse-complement variant position on the x-axis, demonstrating that transcription factor binding in the genome follows inversion symmetry (i.e., the number of transcription factor binding events at a particular DNA element in the genome is equivalent to the number of transcription factor binding events at its reverse-complement DNA element in the genome). This DNA-binding profile was observed in 194 KR experiments, representing a wide variety of mouse tissues and human cell lines, and across multiple peak selection criteria (L4-L20) **(Table S39)**.

Figure S55. (S/N) analysis of 6-nt Variant HREs in KR ChIPSeq Peaks (displayed by variant position)
(S/N) analysis of 6-nt variant HREs in AR (AR-wt1) ChIPSeq peaks [49,859 peaks, 136-nt peak length].
Displayed by the 924 variant positions of the 673,596 6-nt variant HREs. 20 of the 924 variant positions are the five (5) palindromic position pairs: 1-2-3-10-11-12, 3-4-5-8-9-10, 1-3-4-9-10-12, 1-2-4-9-11-12, 2-3-6-7-10-11, 1-3-5-8-10-12, 3-5-6-7-8-10, 2-5-6-7-8-11, 1-2-6-7-11-12, 4-5-6-7-8-9, 1-5-6-7-8-12, 2-3-5-8-10-11, 1-4-6-7-9-12, 2-3-4-9-10-11, 1-2-5-8-11-12, 1-3-6-7-10-12, 2-4-6-7-9-11, 1-4-5-8-9-12, 2-4-5-8-9-11, 3-4-6-7-9-10 (PP, dark red solid bars). 2 of the 924 variant positions are same-side variants (i.e., the variants do not crossover the 3-nt spacer) (orange solid bars). 922 of the 924 variant positions are crossover variants (i.e., the variants do crossover the 3-nt spacer) (orange diagonal stripped bars). Each variant position is immediately followed by its reverse-complement variant position on the x-axis, demonstrating that transcription factor binding in the genome follows inversion symmetry (i.e., the number of transcription factor binding events at a particular DNA element in the genome is equivalent to the number of transcription factor binding events at its reverse-complement DNA element in the genome). This DNA-binding profile was observed in 194 KR experiments, representing a wide variety of mouse tissues and human cell lines, and across multiple peak selection criteria (L4-L20) **(Table S40)**.

*****

Here we show replicate experiments for GR and GR-Dim to illustrate that the slight decrease in DNA-binding at a few specific 3-nt variant HRE DNA elements by GR-Dim is made apparent by the DNA-binding enhancement of a particular experiment at 0-nt to 2-nt variant HRE DNA elements as the relative (S/N) values of sNR DNA-binding signals are scale invariant (i.e., the relative ratios between the 0-nt to 5-nt variant groups are constant) **(Figure S37-S38)**. Thus, the reduced amplitude in DNA-binding at a few specific 3-nt variant HRE DNA elements only occurs in experiments with the highest (S/N) values at 0-nt to 2-nt variant HRE DNA elements. **Note**: see section *"Transform the 13-nt DNA Element Analysis to the 15-nt DNA Element Analysis"* to understand how the 13-nt ERE and HRE DNA elements split into the 15-nt ERE and HRE DNA elements (e.g., explaining why some DNA elements are a 2-nt variant DNA element in the 13-nt analysis and a 4-nt variant DNA element in the 15-nt analysis).

Figure S56. (S/N) analysis of 0-nt to 6-nt Variant HREs in GR and GR-Dim ChIPSeq Peaks #1 (displayed by 924 half-site groups)
(S/N) analysis of 0-nt to 6-nt variant HREs (displayed by the 924 half-site groups) in GR (Liver-GR-WT-pred-6am-2) [23,742 peaks, 142-nt peak length] ChIPSeq peaks and GR-Dim (Liver-GR-Dim-pred-6am-2) [34,966 peaks, 148-nt peak length] ChIPSeq peaks. X-axis order =Reverse-Complement Vacancy Position ID 3-10 > 1-12 > 6-7 > 2-11 > 5-8 > 4-9. See **Table S26** for x-axis details.

Figure S57. (S/N) analysis of 0-nt to 6-nt variant HREs in GR and GR-Dim ChIPSeq Peaks #2 (displayed by 924 half-site groups)
(S/N) analysis of 0-nt to 6-nt variant HREs (displayed by the 924 half-site groups) in GR (Liver-GR-WT-pred-6am-1) [34,758 peaks, 151-nt peak length] ChIPSeq peaks and GR-Dim (Liver-GR-Dim-pred-6am-1) [22,130 peaks, 163-nt peak length] ChIPSeq peaks. X-axis order =Reverse-Complement Vacancy Position ID 3-10 > 1-12 > 6-7 > 2-11 > 5-8 > 4-9. See **Table S26** for x-axis details.

Figure S58. (S/N) analysis of 0-nt to 6-nt variant HREs in GR and GR-Dim ChIPExo Peaks #1 (displayed by 924 half-site groups)
(S/N) analysis of 0-nt to 6-nt variant HREs (displayed by the 924 half-site groups) in GR (Liver-GR-WT-pred-6am-1) [20,966 peaks, 445-nt peak length] ChIPExo peaks and GR-Dim (Liver-GR-Dim-pred-6am-1) [19,480 peaks, 391-nt peak length] ChIPExo peaks. X-axis order =Reverse-Complement Vacancy Position ID 3-10 > 1-12 > 6-7 > 2-11 > 5-8 > 4-9. See **Table S26** for x-axis details.

Figure S59. (S/N) analysis of 0-nt to 6-nt variant HREs in GR and GR-Dim ChIPExo Peaks #2 (displayed by 924 half-site groups)
(S/N) analysis of 0-nt to 6-nt variant HREs (displayed by the 924 half-site groups) in GR (Liver-GR-WT-pred-6am-2) [22,129 peaks, 338-nt peak length] ChIPExo peaks and GR-Dim (Liver-GR-Dim-pred-6am-2) [25,670 peaks, 408-nt peak length] ChIPExo peaks. X-axis order =Reverse-Complement Vacancy Position ID 3-10 > 1-12 > 6-7 > 2-11 > 5-8 > 4-9. See **Table S26** for x-axis details.

Figure S60. (S/N) analysis of 1-nt to 6-nt variant HREs in GR and GR-Dim ChIPSeq Peaks (displayed by variant position)

(S/N) analysis of 1-nt to 6-nt variant HREs (displayed by variant position) in GR (Liver-GR-WT-pred-6am-1) [23,742 peaks, 142-nt peak length] (Liver-GR-WT-pred-6am-2) [34,758 peaks, 151-nt peak length] ChIPSeq peaks and GR-Dim (Liver-GR-Dim-pred-6am-1) [34,966 peaks, 148-nt peak length] (Liver-GR-Dim-pred-6am-2) [22,130 peaks, 163-nt peak length] ChIPSeq peaks.

Figure S61. (S/N) analysis of 1-nt to 6-nt variant HREs in GR and GR-Dim ChIPExo Peaks (displayed by variant position)
(S/N) analysis of 1-nt to 6-nt variant HREs (displayed by variant position) in GR (Liver-GR-WT-pred-6am-1) [20,966 peaks, 445-nt peak length] (Liver-GR-WT-pred-6am-2) [22,129 peaks, 338-nt peak length] ChIPExo peaks and GR-Dim (Liver-GR-Dim-pred-6am-1) [19,480 peaks, 391-nt peak length] (Liver-GR-Dim-pred-6am-2) [25,670 peaks, 408-nt peak length] ChIPExo peaks.

Figure S33. 0-nt Variant 15-nt ERE and HRE Identification in ER and KR ChIPSeq Peaks (16 possibilities)

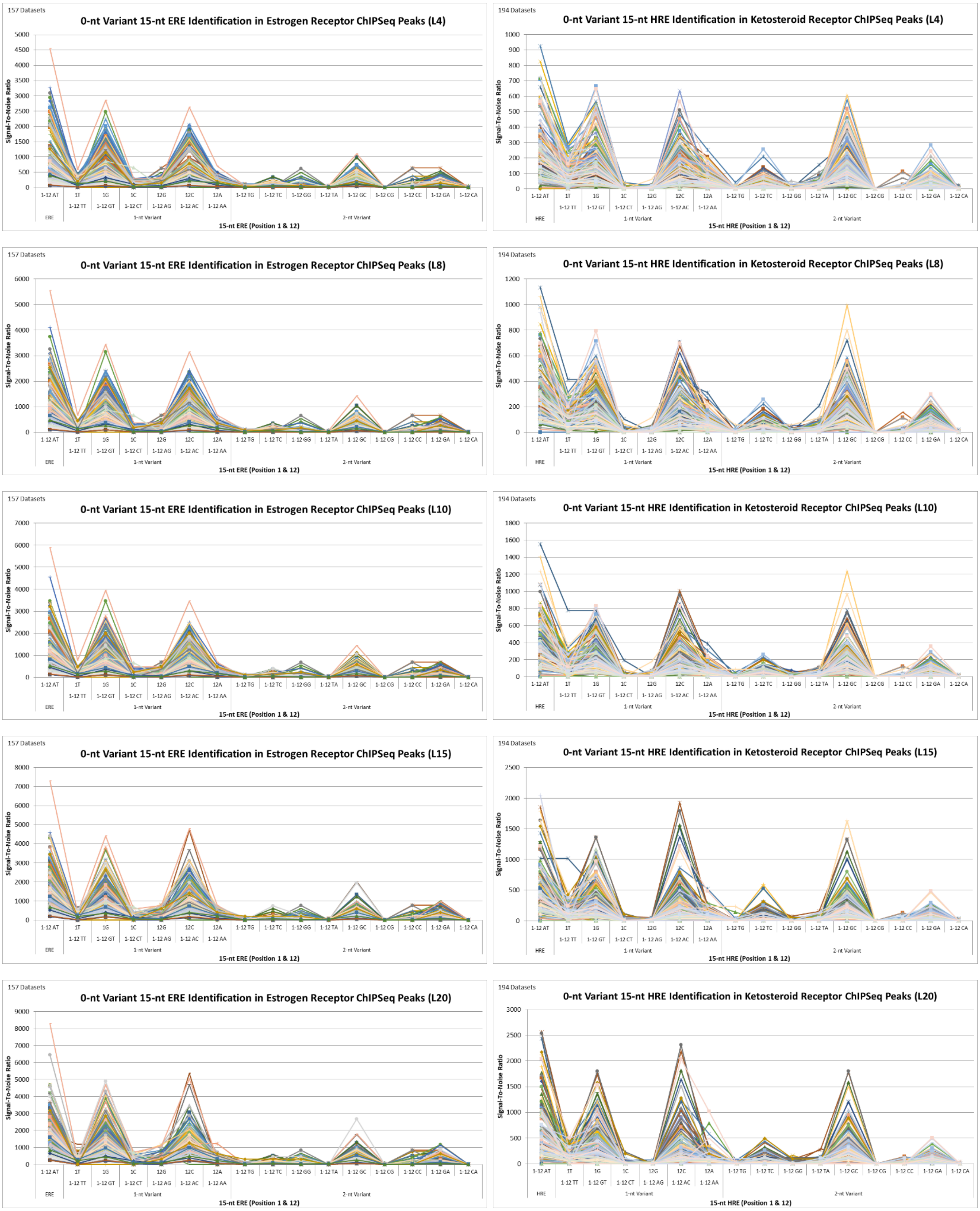

Figure S34. Number of 0-nt to 6-nt Variant 15-nt ERE and HRE DNA Elements in the Mouse and Human Genome

13-nt ERE and HRE DNA Elements (*n*=10)

	DNA Element	Combinatorial Counts				DNA Element Frequency in Genome			
A	B	C	D	E	F	Mouse Genome (mm10)		Human Genome (hg19)	
k	**13-nt ERE** GGTCAnnnTGACC	Combinations C = $\binom{n}{k}$	4-nts D = C x 4^k	Unique E = C x 3^k	Total Unique F = ΣE	Unique	Total Unique	Unique	Total Unique
0	0-nt Variant ERE	1	1	1	1	2,367		2,194	
1	1-nt Variant ERE	10	40	30	31	71,428	73,795	60,313	62,507
2	2-nt Variant ERE	45	720	405	436	898,155	971,950	914,726	977,233
3	3-nt Variant ERE	120	7,680	3,240	3,676	6,750,607	7,722,557	7,516,184	8,493,417
4	4-nt Variant ERE	210	53,760	17,010	20,686	35,508,190	43,230,747	38,222,674	46,716,091
5	5-nt Variant ERE	252	258,048	61,236	81,922	134,732,965	177,963,712	141,280,093	187,996,184

A	B	C	D	E	F	Mouse Genome (mm10)		Human Genome (hg19)	
k	**13-nt HRE** GAACAnnnTGTTC	Combinations C = $\binom{n}{k}$	4-nts D = C x 4^k	Unique E = C x 3^k	Total Unique F = ΣE	Unique	Total Unique	Unique	Total Unique
0	0-nt Variant HRE	1	1	1	1	3,444		3,535	
1	1-nt Variant HRE	10	40	30	31	97,039	100,483	104,767	108,302
2	2-nt Variant HRE	45	720	405	436	1,337,516	1,437,999	1,339,543	1,447,845
3	3-nt Variant HRE	120	7,680	3,240	3,676	10,461,197	11,899,196	10,771,159	12,219,004
4	4-nt Variant HRE	210	53,760	17,010	20,686	49,391,434	61,290,630	54,136,564	66,355,568
5	5-nt Variant HRE	252	258,048	61,236	81,922	169,440,498	230,731,128	187,469,898	253,825,466

A	B	C	D	E	F
k		Combinations C = $\binom{n}{k}$	4-nts D = C x 4^k	Unique E = C x 3^k	Total Unique F = ΣE
0	0-nt Variant	10!/(0! x 10!)	1 x 1	1 x 1	1 + 0
1	1-nt Variant	10!/(1! x 9!)	10 x 4	10 x 3	30 + 1
2	2-nt Variant	10!/(2! x 8!)	45 x 16	45 x 9	405 + 31
3	3-nt Variant	10!/(3! x 7!)	120 x 64	120 x 27	3,240 + 436
4	4-nt Variant	10!/(4! x 6!)	210 x 256	210 x 81	17,010 +3,676
5	5-nt Variant	10!/(5! x 5!)	252 x 1,024	252 x 243	61,236 + 20,686

15-nt ERE and HRE DNA Elements (*n*=12)

	DNA Element	Combinatorial Counts				DNA Element Frequency in Genome			
A	B	C	D	E	F	Mouse Genome (mm10)		Human Genome (hg19)	
k	**15-nt ERE** AGGTCAnnnTGACCT	Combinations C = $\binom{n}{k}$	4-nts D = C x 4^k	Unique E = C x 3^k	Total Unique F = ΣE	Unique	Total Unique	Unique	Total Unique
0	0-nt Variant ERE	1	1	1	1	404		342	
1	1-nt Variant ERE	12	48	36	37	10,964	11,368	9,223	9,565
2	2-nt Variant ERE	66	1056	594	631	152,140	163,508	118,354	127,919
3	3-nt Variant ERE	220	14,080	5,940	6,571	1,256,647	1,420,155	1,213,963	1,341,882
4	4-nt Variant ERE	495	126,720	40,095	46,666	6,916,002	8,336,157	7,349,740	8,691,622
5	5-nt Variant ERE	792	811,008	192,456	239,122	30,575,777	38,911,934	33,052,166	41,743,788
6	6-nt Variant ERE	924	3,784,704	673,596	912,718	104,588,671	143,500,605	113,051,144	154,794,932

A	B	C	D	E	F	Mouse Genome (mm10)		Human Genome (hg19)	
k	**15-nt HRE** AGAACAnnnTGTTCT	Combinations C = $\binom{n}{k}$	4-nts D = C x 4^k	Unique E = C x 3^k	Total Unique F = ΣE	Unique	Total Unique	Unique	Total Unique
0	0-nt Variant HRE	1	1	1	1	639		565	
1	1-nt Variant HRE	12	48	36	37	17,847	18,486	14,639	15,204
2	2-nt Variant HRE	66	1056	594	631	256,540	275,026	206,983	222,187
3	3-nt Variant HRE	220	14,080	5,940	6,571	2,188,277	2,463,303	1,978,470	2,200,657
4	4-nt Variant HRE	495	126,720	40,095	46,666	11,128,133	13,591,436	11,661,975	13,862,632
5	5-nt Variant HRE	792	811,008	192,456	239,122	43,400,607	56,992,043	48,017,520	61,880,152
6	6-nt Variant HRE	924	3,784,704	673,596	912,718	134,064,927	191,056,970	151,083,618	212,963,770

A	B	C	D	E	F
k		Combinations C = $\binom{n}{k}$	4-nts D = C x 4^k	Unique E = C x 3^k	Total Unique F = ΣE
0	0-nt Variant	12!/(0! x 12!)	1 x 1	1 x 1	1 + 0
1	1-nt Variant	12!/(1! x 11!)	12 x 4	12 x 3	36 + 1
2	2-nt Variant	12!/(2! x 10!)	66 x 16	66 x 9	594 + 37
3	3-nt Variant	12!/(3! x 9!)	220 x 64	220 x 27	5,940 + 631
4	4-nt Variant	12!/(4! x 8!)	495 x 256	495 x 81	40,095 + 6,571
5	5-nt Variant	12!/(5! x 7!)	792 x 1,024	792 x 243	192,456 + 46,666
6	6-nt Variant	12!/(6! x 6!)	924 x 4,096	924 x 729	673,596 + 239,122

Figure S35. Inversion Symmetry of sNR DNA-Binding at 1-nt Variant EREs and HREs in the Genome (%)

(A) 1-nt Variant EREs in ER ChIPSeq Peaks (157 Experiments)

ERE = AG<u>GT</u>CAnnnTG<u>AC</u>CT

1 2 3 4 5 6 7 8 9 10 11 12

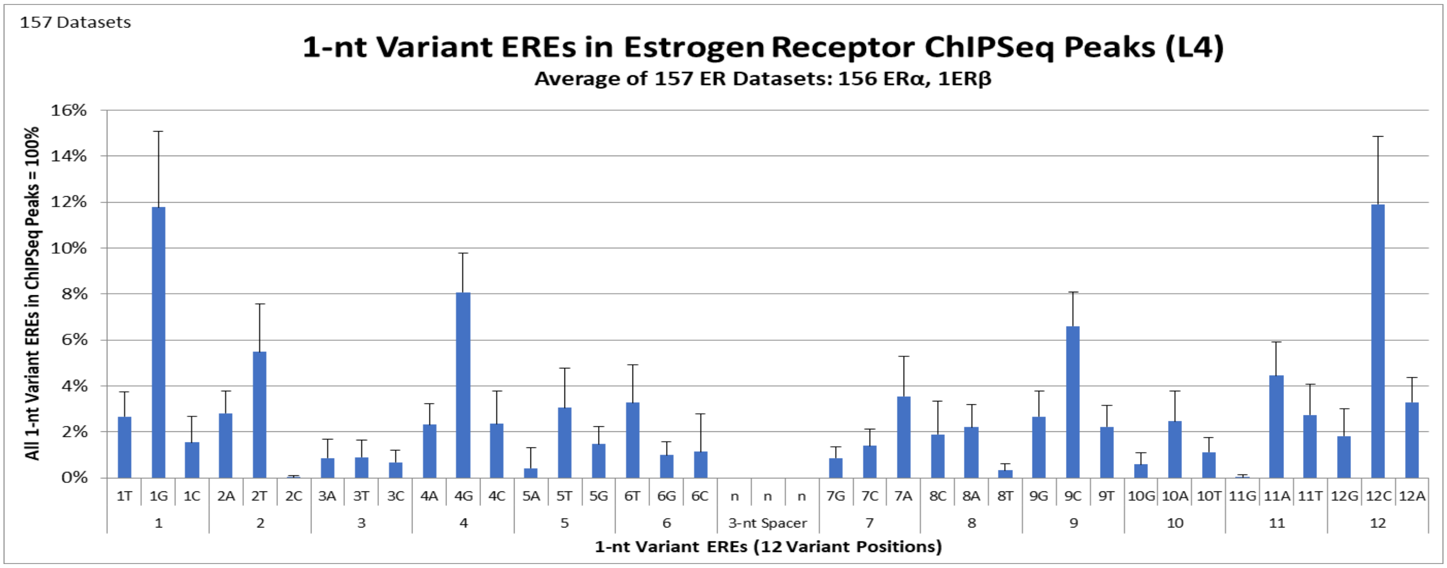

(B) 1-nt Variant HREs in KR ChIPSeq Peaks (193 Experiments)

HRE = AG<u>AA</u>CAnnnTG<u>TT</u>CT

1 2 3 4 5 6 7 8 9 10 11 12

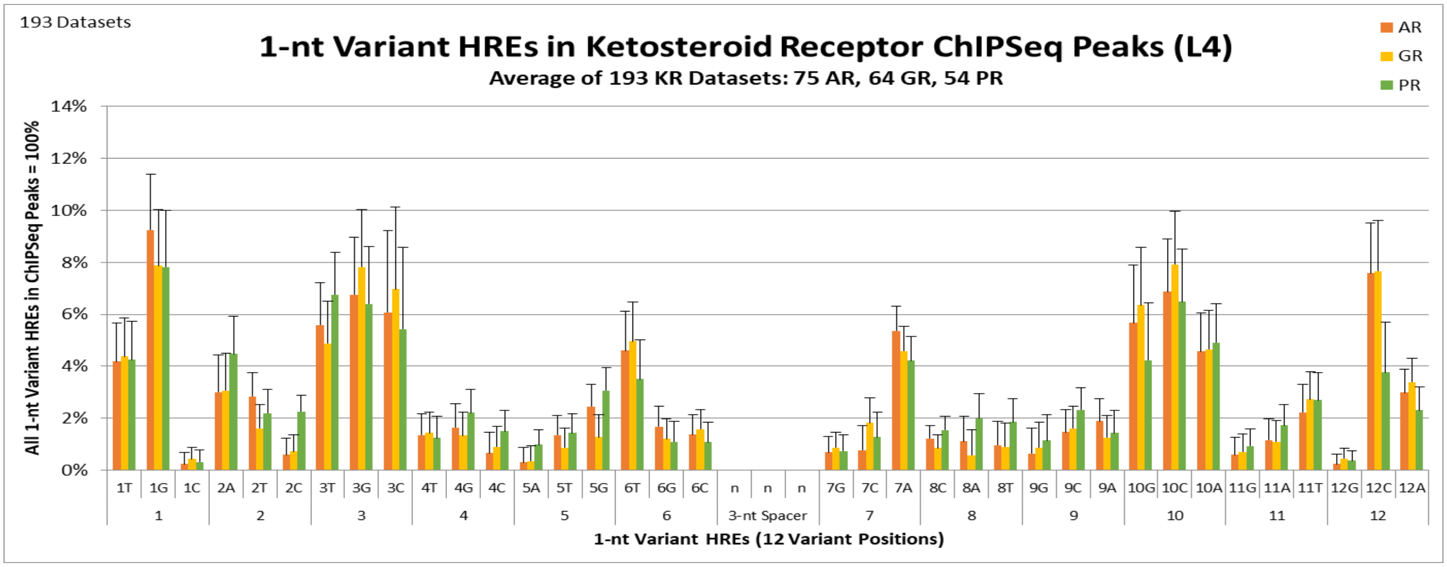

Figure S36. Inversion Symmetry of sNR DNA-Binding at 2-nt Variant EREs and HREs in the Genome (%)

(A) 2-nt Variant EREs in ER ChIPSeq Peaks (157 Experiments)

ERE = AGGTCAnnnTGACCT
1 2 3 4 5 6 7 8 9 10 11 12

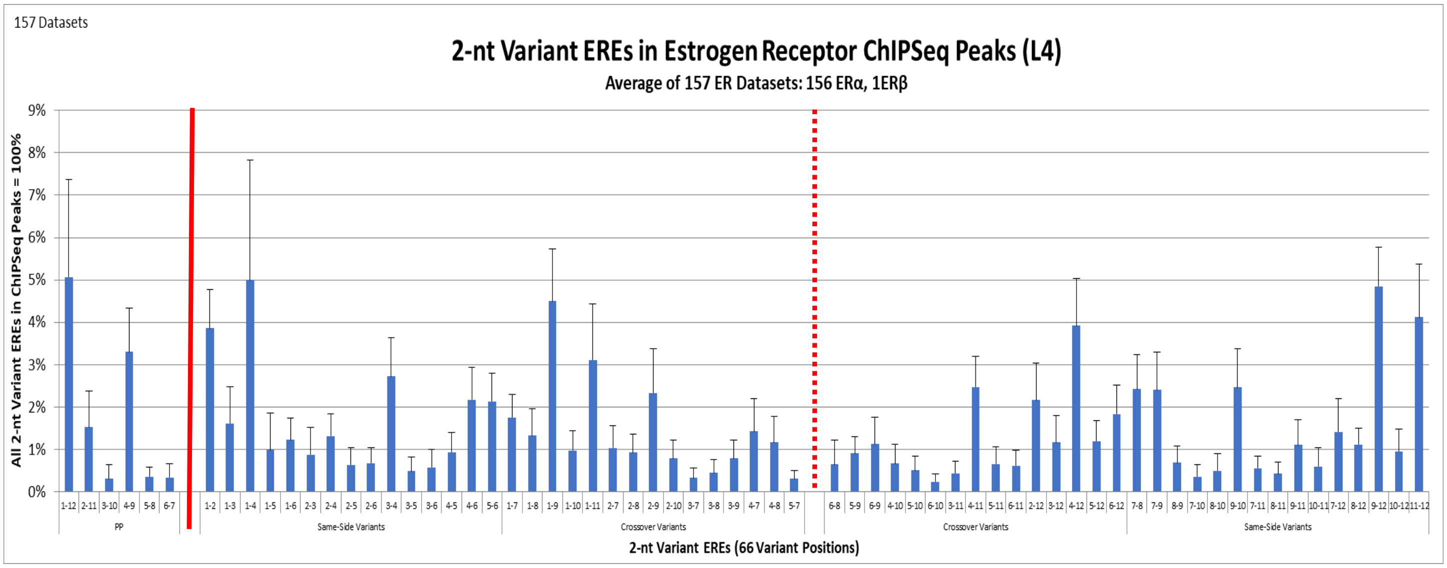

---- **Symmetry Divider**

(B) 2-nt Variant HREs in KR ChIPSeq Peaks (193 Experiments)

HRE = AGAACAnnnTGTTCT
1 2 3 4 5 6 7 8 9 10 11 12

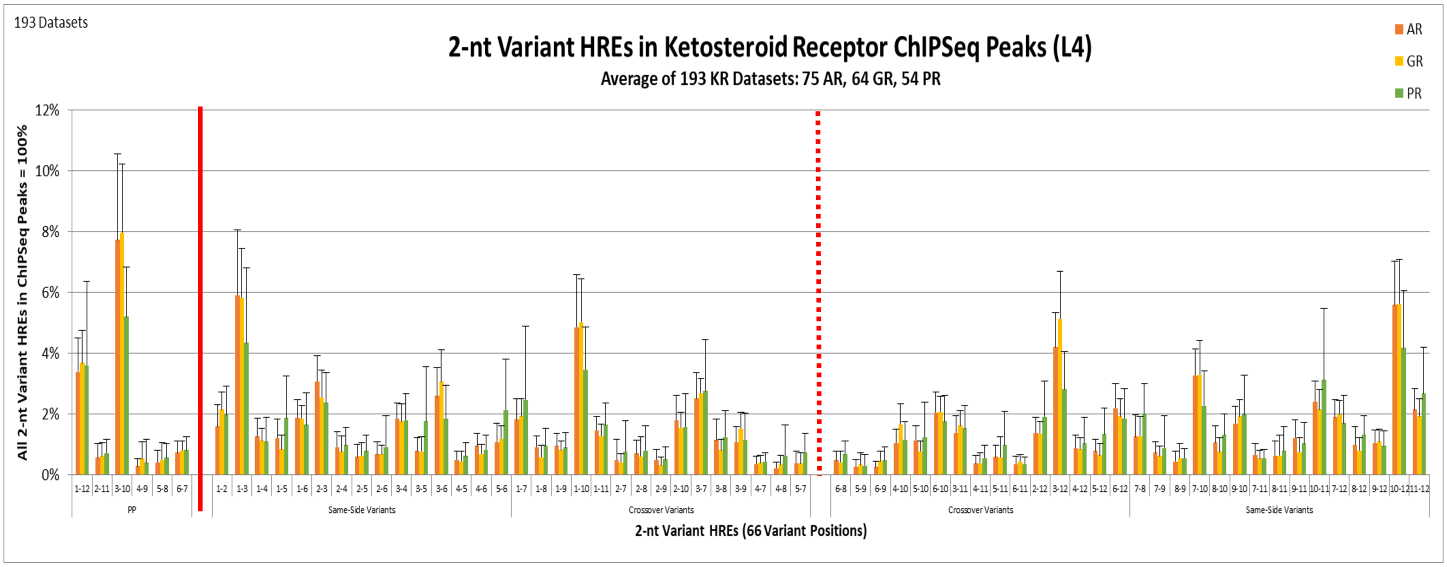

---- **Symmetry Divider**

Figure S37. (S/N) analysis of 0-nt to 6-nt Variant EREs in ER ChIPSeq Peaks (# of Variants)

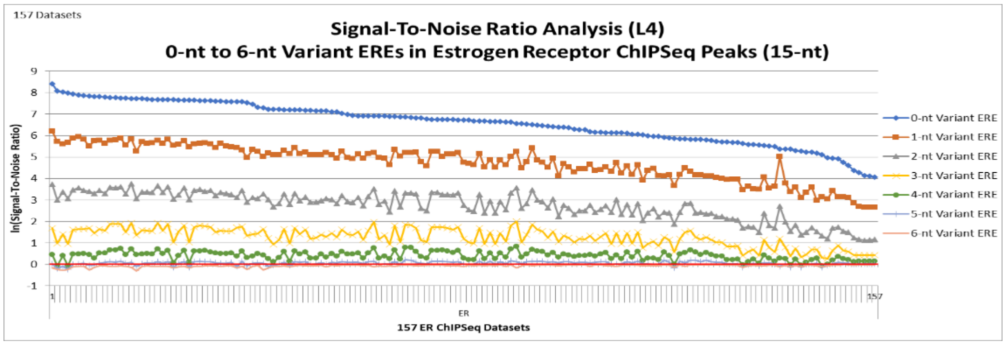

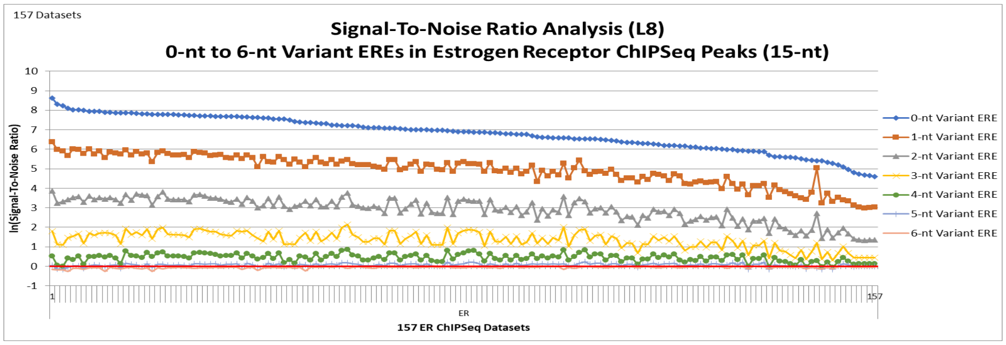

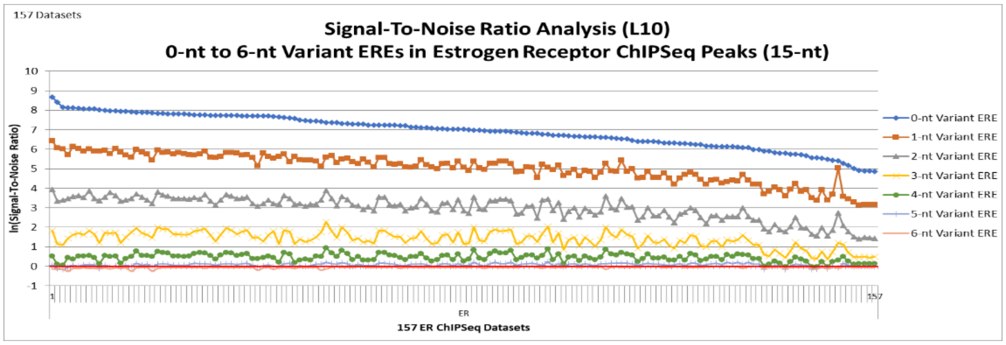

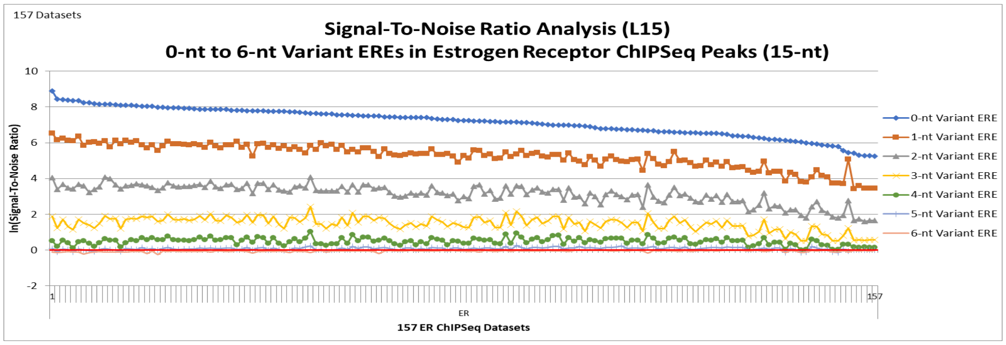

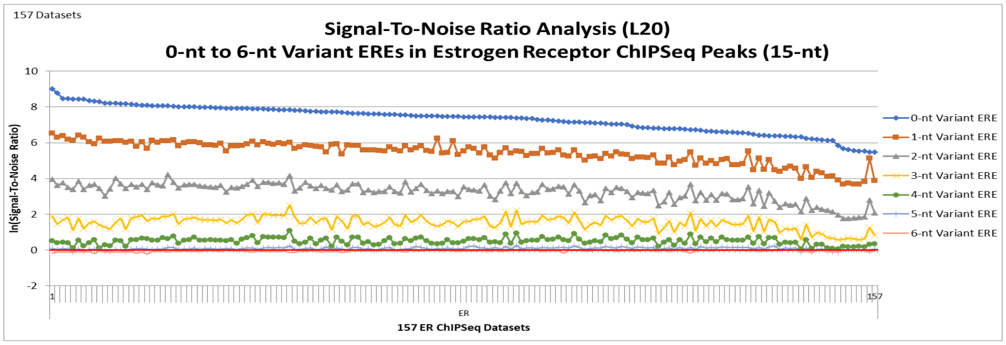

Figure S38. (S/N) analysis of 0-nt to 6-nt Variant HREs in KR ChIPSeq Peaks (# of Variants)

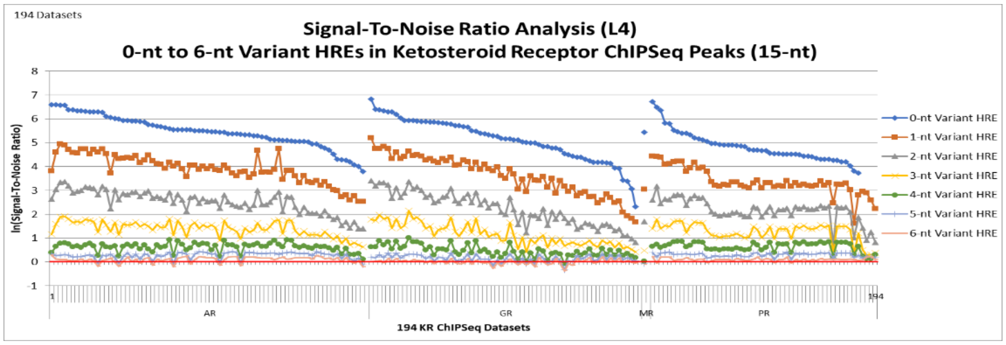

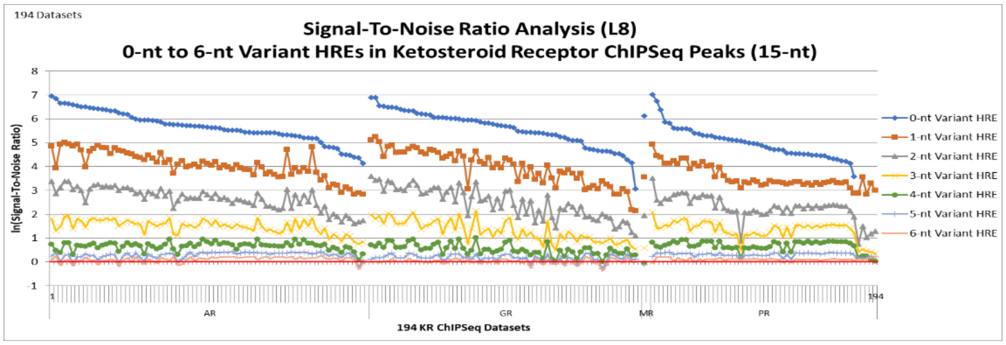

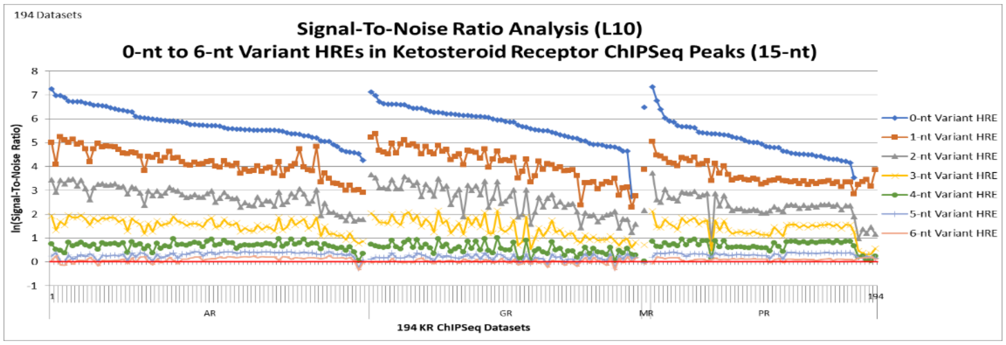

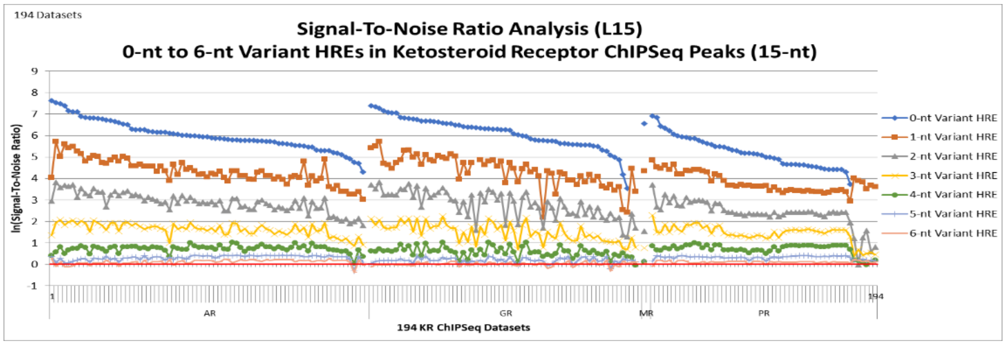

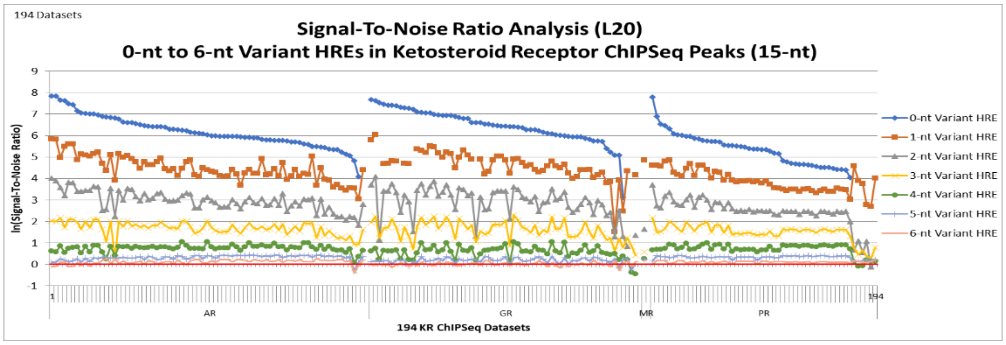

Figure S39. 924 Half-Site Groups

	A	B	C	D	E
	**Fixed Half-Site Positions**	**Reverse-Complement Vacancies & Double Occupants**	**Reverse-Complement Vacancy Position ID**	**Reverse-Complement Double Occupant Position ID**	**Positions That Can Be Varied**
			**Zero Vacancies**		
1	1-2-3-4-5-6------	0V			------7-8-9-10-11-12
2	1-2-3--5-6---9---	0V			---4---7-8--10-11-12
3	-2-3-4-5-6------12	0V			1------7-8-9-10-11-
4	1--3-4-5-6-----11-	0V			-2-----7-8-9-10--12
5	1-2-3-4-5--7-----	0V			-----6--8-9-10-11-12
6	1-2-3-4--6--8----	0V			----5--7--9-10-11-12
7	1-2--4-5-6----10--	0V			--3----7-8-9--11-12
8	-2-3--5-6---9---12	0V			1---4---7-8--10-11-
9	1--3--5-6---9--11-	0V			-2--4---7-8--10--12
10	1-2-3--5--7--9---	0V			---4--6--8--10-11-12
11	1-2-3---6--8-9---	0V			---4-5--7---10-11-12
12	1-2---5-6---9-10--	0V			--3-4---7-8---11-12
13	--3-4-5-6-----11-12	0V			1-2-----7-8-9-10--
14	-2-3-4-5--7-----12	0V			1-----6--8-9-10-11-
15	-2-3-4--6--8----12	0V			1----5--7--9-10-11-
16	-2--4-5-6----10--12	0V			1--3----7-8-9--11-
17	1--3-4-5--7----11-	0V			-2----6--8-9-10--12
18	1--3-4--6--8---11-	0V			-2---5--7--9-10--12
19	1---4-5-6----10-11-	0V			-2-3----7-8-9---12
20	1-2-3-4---7-8----	0V			----5-6---9-10-11-12
21	1-2--4-5--7---10--	0V			--3---6--8-9--11-12
22	1-2--4--6--8--10--	0V			--3--5--7--9--11-12
23	-2-3--5--7--9---12	0V			1---4--6--8--10-11-
24	1--3--5--7--9--11-	0V			-2--4--6--8--10--12
25	1-2-3----7-8-9---	0V			---4-5-6----10-11-12
26	1-2---5--7--9-10--	0V			--3-4--6--8---11-12
27	--3-4-5--7----11-12	0V			1-2----6--8-9-10--
28	-2-3-4---7-8----12	0V			1----5-6---9-10-11-
29	-2--4-5--7---10--12	0V			1--3---6--8-9--11-
30	1--3-4---7-8---11-	0V			-2---5-6---9-10--12
31	1---4-5--7---10-11-	0V			-2-3---6--8-9---12
32	1-2--4---7-8--10--	0V			--3--5-6---9--11-12
			**One Vacancy**		
33	1-2-3--5--7-----12	1V	4	1	---4--6--8-9-10-11-
34	1--3--5--7----11-12	1V	4	1	-2--4--6--8-9-10--
35	1-2-3----7-8----12	1V	4	1	---4-5-6---9-10-11-
36	1-2---5--7---10--12	1V	4	1	--3-4--6--8-9--11-
37	1--3----7-8---11-12	1V	4	1	-2--4-5-6---9-10--
38	1----5--7---10-11-12	1V	4	1	-2-3-4--6--8-9---
39	1-2-----7-8--10--12	1V	4	1	--3-4-5-6---9--11-
40	1------7-8--10-11-12	1V	4	1	-2-3-4-5-6---9---
41	1-2-3--5--7----11-	1V	4	2	---4--6--8-9-10--12
42	-2-3--5--7----11-12	1V	4	2	1---4--6--8-9-10--
43	1-2-3----7-8---11-	1V	4	2	---4-5-6---9-10--12
44	1-2---5--7---10-11-	1V	4	2	--3-4--6--8-9---12
45	-2-3----7-8---11-12	1V	4	2	1---4-5-6---9-10--
46	-2---5--7---10-11-12	1V	4	2	1--3-4--6--8-9---
47	1-2-----7-8--10-11-	1V	4	2	--3-4-5-6---9---12
48	-2-----7-8--10-11-12	1V	4	2	1--3-4-5-6---9---
49	1-2-3---6-7-8----	1V	4	6	---4-5----9-10-11-12
50	-2-3---6-7-8----12	1V	4	6	1---4-5----9-10-11-
51	1--3---6-7-8---11-	1V	4	6	-2--4-5----9-10--12
52	1-2----6-7-8--10--	1V	4	6	--3-4-5----9--11-12
53	--3---6-7-8---11-12	1V	4	6	1-2--4-5----9-10--
54	-2----6-7-8--10--12	1V	4	6	1--3-4-5----9--11-
55	1-----6-7-8--10-11-	1V	4	6	-2-3-4-5----9---12
56	-----6-7-8--10-11-12	1V	4	6	1-2-3-4-5----9---
57	1-2-3--5--7-8----	1V	4	5	---4--6---9-10-11-12
58	-2-3--5--7-8----12	1V	4	5	1---4--6---9-10-11-
59	1--3--5--7-8---11-	1V	4	5	-2--4--6---9-10--12
60	1-2---5--7-8--10--	1V	4	5	--3-4--6---9--11-12
61	--3--5--7-8---11-12	1V	4	5	1-2--4--6---9-10--
62	-2---5--7-8--10--12	1V	4	5	1--3-4--6---9--11-
63	1----5--7-8--10-11-	1V	4	5	-2-3-4--6---9---12
64	----5--7-8--10-11-12	1V	4	5	1-2-3-4--6---9---
65	1-2-3--5--7---10--	1V	4	3	---4--6--8-9--11-12
66	-2-3--5--7---10--12	1V	4	3	1---4--6--8-9--11-
67	1--3--5--7---10-11-	1V	4	3	-2--4--6--8-9---12
68	1-2-3----7-8--10--	1V	4	3	---4-5-6---9--11-12
69	--3--5--7---10-11-12	1V	4	3	1-2--4--6--8-9---
70	-2-3----7-8--10--12	1V	4	3	1---4-5-6---9--11-
71	1--3----7-8--10-11-	1V	4	3	-2--4-5-6---9---12
72	--3----7-8--10-11-12	1V	4	3	1-2--4-5-6---9---
73	-2-3-4-5--7--9---	1V	1	4	1-----6--8--10-11-12

74	--3-4-5--7--9--11-	1V	1	4	1-2----6--8--10--12
75	-2-3-4---7-8-9---	1V	1	4	1----5-6----10-11-12
76	-2--4-5--7--9-10--	1V	1	4	1--3---6--8---11-12
77	--3-4---7-8-9--11-	1V	1	4	1-2---5-6----10--12
78	---4-5--7--9-10-11-	1V	1	4	1-2-3---6--8----12
79	-2--4---7-8-9-10--	1V	1	4	1--3--5-6-----11-12
80	---4---7-8-9-10-11-	1V	1	4	1-2-3--5-6------12
81	-2-3-4-5--7----11-	1V	1	2	1-----6--8-9-10--12
82	-2-3--5--7--9--11-	1V	1	2	1---4--6--8--10--12
83	-2-3-4---7-8---11-	1V	1	2	1----5-6---9-10--12
84	-2--4-5--7---10-11-	1V	1	2	1--3---6--8-9---12
85	-2-3----7-8-9--11-	1V	1	2	1---4-5-6----10--12
86	-2---5--7--9-10-11-	1V	1	2	1--3-4--6--8----12
87	-2--4---7-8--10-11-	1V	1	2	1--3--5-6---9---12
88	-2-----7-8-9-10-11-	1V	1	2	1--3-4-5-6------12
89	-2-3-4--6-7-8----	1V	1	6	1----5----9-10-11-12
90	-2-3---6-7-8-9---	1V	1	6	1---4-5-----10-11-12
91	--3-4--6-7-8---11-	1V	1	6	1-2---5----9-10--12
92	-2--4--6-7-8--10--	1V	1	6	1--3--5----9--11-12
93	--3---6-7-8-9--11-	1V	1	6	1-2--4-5-----10--12
94	-2----6-7-8-9-10--	1V	1	6	1--3-4-5------11-12
95	---4--6-7-8--10-11-	1V	1	6	1-2-3--5----9---12
96	-----6-7-8-9-10-11-	1V	1	6	1-2-3-4-5-------12
97	-2-3-4-5--7-8----	1V	1	5	1-----6---9-10-11-12
98	-2-3--5--7-8-9---	1V	1	5	1---4--6----10-11-12
99	--3-4-5--7-8---11-	1V	1	5	1-2----6---9-10--12
100	-2--4-5--7-8--10--	1V	1	5	1--3---6---9--11-12
101	--3--5--7-8-9--11-	1V	1	5	1-2--4--6----10--12
102	-2---5--7-8-9-10--	1V	1	5	1--3-4--6-----11-12
103	---4-5--7-8--10-11-	1V	1	5	1-2-3---6---9---12
104	----5--7-8-9-10-11-	1V	1	5	1-2-3-4--6------12
105	-2-3-4-5--7---10--	1V	1	3	1-----6--8-9--11-12
106	-2-3--5--7--9-10--	1V	1	3	1---4--6--8---11-12
107	--3-4-5--7---10-11-	1V	1	3	1-2----6--8-9---12
108	-2-3-4---7-8--10--	1V	1	3	1----5-6---9--11-12
109	--3--5--7--9-10-11-	1V	1	3	1-2--4--6--8----12
110	-2-3----7-8-9-10--	1V	1	3	1---4-5-6-----11-12
111	--3-4---7-8--10-11-	1V	1	3	1-2---5-6---9---12
112	--3----7-8-9-10-11-	1V	1	3	1-2--4-5-6------12
113	1--3-4-5--7--9---	1V	2	4	-2----6--8--10-11-12
114	--3-4-5--7--9---12	1V	2	4	1-2----6--8--10-11-
115	1--3-4---7-8-9---	1V	2	4	-2---5-6----10-11-12
116	1---4-5--7--9-10--	1V	2	4	-2-3---6--8---11-12
117	--3-4---7-8-9---12	1V	2	4	1-2---5-6----10-11-
118	---4-5--7--9-10--12	1V	2	4	1-2-3---6--8---11-
119	1---4---7-8-9-10--	1V	2	4	-2-3--5-6-----11-12
120	---4---7-8-9-10--12	1V	2	4	1-2-3--5-6-----11-
121	1--3-4-5--7-----12	1V	2	1	-2----6--8-9-10-11-
122	1--3--5--7--9---12	1V	2	1	-2--4--6--8--10-11-
123	1--3-4---7-8----12	1V	2	1	-2---5-6---9-10-11-
124	1---4-5--7---10--12	1V	2	1	-2-3---6--8-9---11-
125	1--3----7-8-9---12	1V	2	1	-2--4-5-6----10-11-
126	1----5--7--9-10--12	1V	2	1	-2-3-4--6--8---11-
127	1---4---7-8--10--12	1V	2	1	-2-3--5-6---9---11-
128	1------7-8-9-10--12	1V	2	1	-2-3-4-5-6-----11-
129	1--3-4--6-7-8----	1V	2	6	-2---5----9-10-11-12
130	1--3---6-7-8-9---	1V	2	6	-2--4-5-----10-11-12
131	--3-4--6-7-8----12	1V	2	6	1-2---5----9-10-11-
132	1---4--6-7-8--10--	1V	2	6	-2-3--5----9--11-12
133	--3---6-7-8-9---12	1V	2	6	1-2--4-5-----10-11-
134	1-----6-7-8-9-10--	1V	2	6	-2-3-4-5------11-12
135	---4--6-7-8--10--12	1V	2	6	1-2-3--5----9---11-
136	-----6-7-8-9-10--12	1V	2	6	1-2-3-4-5------11-
137	1--3-4-5--7-8----	1V	2	5	-2----6---9-10-11-12
138	1--3--5--7-8-9---	1V	2	5	-2--4--6----10-11-12
139	--3-4-5--7-8----12	1V	2	5	1-2----6---9-10-11-
140	1---4-5--7-8--10--	1V	2	5	-2-3---6---9--11-12
141	--3--5--7-8-9---12	1V	2	5	1-2--4--6----10-11-
142	1----5--7-8-9-10--	1V	2	5	-2-3-4--6-----11-12
143	---4-5--7-8--10--12	1V	2	5	1-2-3---6---9---11-
144	----5--7-8-9-10--12	1V	2	5	1-2-3-4--6-----11-
145	1--3-4-5--7---10--	1V	2	3	-2----6--8-9--11-12
146	1--3--5--7--9-10--	1V	2	3	-2--4--6--8---11-12
147	--3-4-5--7---10--12	1V	2	3	1-2----6--8-9---11-
148	1--3-4---7-8--10--	1V	2	3	-2---5-6---9--11-12
149	--3--5--7--9-10--12	1V	2	3	1-2--4--6--8---11-
150	1--3----7-8-9-10--	1V	2	3	-2--4-5-6-----11-12
151	--3-4---7-8--10--12	1V	2	3	1-2---5-6---9---11-
152	--3----7-8-9-10--12	1V	2	3	1-2--4-5-6-----11-
153	1-2-3-4----8-9---	1V	6	4	----5-6-7---10-11-12
154	-2-3-4----8-9---12	1V	6	4	1----5-6-7---10-11-
155	1--3-4----8-9--11-	1V	6	4	-2---5-6-7---10--12
156	1-2--4----8-9-10--	1V	6	4	--3--5-6-7----11-12

157	--3-4----8-9--11-12	1V	6	4	1-2---5-6-7---10--
158	-2--4----8-9-10--12	1V	6	4	1--3--5-6-7----11-
159	1---4----8-9-10-11-	1V	6	4	-2-3--5-6-7-----12
160	---4----8-9-10-11-12	1V	6	4	1-2-3--5-6-7-----
161	1-2-3-4----8----12	1V	6	1	----5-6-7--9-10-11-
162	1-2-3-----8-9---12	1V	6	1	---4-5-6-7---10-11-
163	1--3-4----8---11-12	1V	6	1	-2---5-6-7--9-10--
164	1-2--4----8--10--12	1V	6	1	--3--5-6-7--9--11-
165	1--3-----8-9--11-12	1V	6	1	-2--4-5-6-7---10--
166	1-2------8-9-10--12	1V	6	1	--3-4-5-6-7----11-
167	1---4----8--10-11-12	1V	6	1	-2-3--5-6-7--9---
168	1-------8-9-10-11-12	1V	6	1	-2-3-4-5-6-7-----
169	1-2-3-4----8---11-	1V	6	2	----5-6-7--9-10--12
170	1-2-3-----8-9--11-	1V	6	2	---4-5-6-7---10--12
171	-2-3-4----8---11-12	1V	6	2	1----5-6-7--9-10--
172	1-2--4----8--10-11-	1V	6	2	--3--5-6-7--9---12
173	-2-3-----8-9--11-12	1V	6	2	1---4-5-6-7---10--
174	1-2------8-9-10-11-	1V	6	2	--3-4-5-6-7-----12
175	-2--4----8--10-11-12	1V	6	2	1--3--5-6-7--9---
176	-2------8-9-10-11-12	1V	6	2	1--3-4-5-6-7-----
177	1-2-3--5---8-9---	1V	6	5	---4--6-7---10-11-12
178	-2-3--5---8-9---12	1V	6	5	1---4--6-7---10-11-
179	1--3--5---8-9--11-	1V	6	5	-2--4--6-7---10--12
180	1-2---5---8-9-10--	1V	6	5	--3-4--6-7----11-12
181	--3--5---8-9--11-12	1V	6	5	1-2--4--6-7---10--
182	-2---5---8-9-10--12	1V	6	5	1--3-4--6-7----11-
183	1----5---8-9-10-11-	1V	6	5	-2-3-4--6-7-----12
184	----5---8-9-10-11-12	1V	6	5	1-2-3-4--6-7-----
185	1-2-3-4----8--10--	1V	6	3	----5-6-7--9--11-12
186	1-2-3-----8-9-10--	1V	6	3	---4-5-6-7----11-12
187	-2-3-4----8--10--12	1V	6	3	1----5-6-7--9--11-
188	1--3-4----8--10-11-	1V	6	3	-2---5-6-7--9---12
189	-2-3-----8-9-10--12	1V	6	3	1---4-5-6-7----11-
190	1--3-----8-9-10-11-	1V	6	3	-2--4-5-6-7-----12
191	--3-4----8--10-11-12	1V	6	3	1-2---5-6-7--9---
192	--3-----8-9-10-11-12	1V	6	3	1-2--4-5-6-7-----
193	1-2-3-4---7--9---	1V	5	4	----5-6--8--10-11-12
194	-2-3-4---7--9---12	1V	5	4	1----5-6--8--10-11-
195	1--3-4---7--9--11-	1V	5	4	-2---5-6--8--10--12
196	1-2--4---7--9-10--	1V	5	4	--3--5-6--8---11-12
197	--3-4---7--9--11-12	1V	5	4	1-2---5-6--8--10--
198	-2--4---7--9-10--12	1V	5	4	1--3--5-6--8---11-
199	1---4---7--9-10-11-	1V	5	4	-2-3--5-6--8----12
200	---4---7--9-10-11-12	1V	5	4	1-2-3--5-6--8----
201	1-2-3-4---7-----12	1V	5	1	----5-6--8-9-10-11-
202	1-2-3----7--9---12	1V	5	1	---4-5-6--8--10-11-
203	1--3-4---7----11-12	1V	5	1	-2---5-6--8-9-10--
204	1-2--4---7---10--12	1V	5	1	--3--5-6--8-9--11-
205	1--3----7--9--11-12	1V	5	1	-2--4-5-6--8--10--
206	1-2-----7--9-10--12	1V	5	1	--3-4-5-6--8---11-
207	1---4---7---10-11-12	1V	5	1	-2-3--5-6--8-9---
208	1------7--9-10-11-12	1V	5	1	-2-3-4-5-6--8----
209	1-2-3-4---7----11-	1V	5	2	----5-6--8-9-10--12
210	1-2-3----7--9--11-	1V	5	2	---4-5-6--8--10--12
211	-2-3-4---7----11-12	1V	5	2	1----5-6--8-9-10--
212	1-2--4---7---10-11-	1V	5	2	--3--5-6--8-9---12
213	-2-3----7--9--11-12	1V	5	2	1---4-5-6--8--10--
214	1-2-----7--9-10-11-	1V	5	2	--3-4-5-6--8----12
215	-2--4---7---10-11-12	1V	5	2	1--3--5-6--8-9---
216	-2-----7--9-10-11-12	1V	5	2	1--3-4-5-6--8----
217	1-2-3---6-7--9---	1V	5	6	---4-5---8--10-11-12
218	-2-3---6-7--9---12	1V	5	6	1---4-5---8--10-11-
219	1--3---6-7--9--11-	1V	5	6	-2--4-5---8--10--12
220	1-2----6-7--9-10--	1V	5	6	--3-4-5---8---11-12
221	--3---6-7--9--11-12	1V	5	6	1-2--4-5---8--10--
222	-2----6-7--9-10--12	1V	5	6	1--3-4-5---8---11-
223	1-----6-7--9-10-11-	1V	5	6	-2-3-4-5---8----12
224	-----6-7--9-10-11-12	1V	5	6	1-2-3-4-5---8----
225	1-2-3-4---7---10--	1V	5	3	----5-6--8-9--11-12
226	1-2-3----7--9-10--	1V	5	3	---4-5-6--8---11-12
227	-2-3-4---7---10--12	1V	5	3	1----5-6--8-9--11-
228	1--3-4---7---10-11-	1V	5	3	-2---5-6--8-9---12
229	-2-3----7--9-10--12	1V	5	3	1---4-5-6--8---11-
230	1--3----7--9-10-11-	1V	5	3	-2--4-5-6--8----12
231	--3-4---7---10-11-12	1V	5	3	1-2---5-6--8-9---
232	--3----7--9-10-11-12	1V	5	3	1-2--4-5-6--8----
233	1-2--4-5--7--9---	1V	3	4	--3---6--8--10-11-12
234	-2--4-5--7--9---12	1V	3	4	1--3---6--8--10-11-
235	1---4-5--7--9--11-	1V	3	4	-2-3---6--8--10--12
236	1-2--4---7-8-9---	1V	3	4	--3--5-6----10-11-12
237	---4-5--7--9--11-12	1V	3	4	1-2-3---6--8--10--
238	-2--4---7-8-9---12	1V	3	4	1--3--5-6----10-11-
239	1---4---7-8-9--11-	1V	3	4	-2-3--5-6----10--12

240	---4---7-8-9--11-12	1V	3	4	1-2-3--5-6----10--
241	1-2--4-5--7-----12	1V	3	1	--3---6--8-9-10-11-
242	1-2---5--7--9---12	1V	3	1	--3-4--6--8--10-11-
243	1---4-5--7----11-12	1V	3	1	-2-3---6--8-9-10--
244	1-2--4---7-8----12	1V	3	1	--3--5-6---9-10-11-
245	1----5--7--9--11-12	1V	3	1	-2-3-4--6--8--10--
246	1-2-----7-8-9---12	1V	3	1	--3-4-5-6----10-11-
247	1---4---7-8---11-12	1V	3	1	-2-3--5-6---9-10--
248	1------7-8-9--11-12	1V	3	1	-2-3-4-5-6----10--
249	1-2--4-5--7----11-	1V	3	2	--3---6--8-9-10--12
250	1-2---5--7--9--11-	1V	3	2	--3-4--6--8--10--12
251	-2--4-5--7----11-12	1V	3	2	1--3---6--8-9-10--
252	1-2--4---7-8---11-	1V	3	2	--3--5-6---9-10--12
253	-2---5--7--9--11-12	1V	3	2	1--3-4--6--8--10--
254	1-2-----7-8-9--11-	1V	3	2	--3-4-5-6----10--12
255	-2--4---7-8---11-12	1V	3	2	1--3--5-6---9-10--
256	-2-----7-8-9--11-12	1V	3	2	1--3-4-5-6----10--
257	1-2--4--6-7-8----	1V	3	6	--3--5----9-10-11-12
258	1-2----6-7-8-9---	1V	3	6	--3-4-5-----10-11-12
259	-2--4--6-7-8----12	1V	3	6	1--3--5----9-10-11-
260	1---4--6-7-8---11-	1V	3	6	-2-3--5----9-10--12
261	-2----6-7-8-9---12	1V	3	6	1--3-4-5-----10-11-
262	1-----6-7-8-9--11-	1V	3	6	-2-3-4-5-----10--12
263	---4--6-7-8---11-12	1V	3	6	1-2-3--5----9-10--
264	-----6-7-8-9--11-12	1V	3	6	1-2-3-4-5-----10--
265	1-2--4-5--7-8----	1V	3	5	--3---6---9-10-11-12
266	1-2---5--7-8-9---	1V	3	5	--3-4--6----10-11-12
267	-2--4-5--7-8----12	1V	3	5	1--3---6---9-10-11-
268	1---4-5--7-8---11-	1V	3	5	-2-3---6---9-10--12
269	-2---5--7-8-9---12	1V	3	5	1--3-4--6----10-11-
270	1----5--7-8-9--11-	1V	3	5	-2-3-4--6----10--12
271	---4-5--7-8---11-12	1V	3	5	1-2-3---6---9-10--
272	----5--7-8-9--11-12	1V	3	5	1-2-3-4--6----10--
**Two Vacancies**					
273	-2-3---6-7-8---11-	2V	1-4	2-6	1---4-5----9-10--12
274	-2----6-7-8--10-11-	2V	1-4	2-6	1--3-4-5----9---12
275	-2-3--5--7-8---11-	2V	1-4	2-5	1---4--6---9-10--12
276	-2---5--7-8--10-11-	2V	1-4	2-5	1--3-4--6---9---12
277	-2-3--5--7---10-11-	2V	1-4	2-3	1---4--6--8-9---12
278	-2-3----7-8--10-11-	2V	1-4	2-3	1---4-5-6---9---12
279	-2-3--5-6-7-8----	2V	1-4	5-6	1---4-----9-10-11-12
280	--3--5-6-7-8---11-	2V	1-4	5-6	1-2--4-----9-10--12
281	-2-3---6-7-8--10--	2V	1-4	3-6	1---4-5----9--11-12
282	--3---6-7-8--10-11-	2V	1-4	3-6	1-2--4-5----9---12
283	-2-3--5--7-8--10--	2V	1-4	3-5	1---4--6---9--11-12
284	--3--5--7-8--10-11-	2V	1-4	3-5	1-2--4--6---9---12
285	1--3---6-7-8----12	2V	2-4	1-6	-2--4-5----9-10-11-
286	1-----6-7-8--10--12	2V	2-4	1-6	-2-3-4-5----9--11-
287	1--3--5--7-8----12	2V	2-4	1-5	-2--4--6---9-10-11-
288	1----5--7-8--10--12	2V	2-4	1-5	-2-3-4--6---9--11-
289	1--3--5--7---10--12	2V	2-4	1-3	-2--4--6--8-9--11-
290	1--3----7-8--10--12	2V	2-4	1-3	-2--4-5-6---9--11-
291	1--3--5-6-7-8----	2V	2-4	5-6	-2--4-----9-10-11-12
292	--3--5-6-7-8----12	2V	2-4	5-6	1-2--4-----9-10-11-
293	1--3---6-7-8--10--	2V	2-4	3-6	-2--4-5----9--11-12
294	--3---6-7-8--10--12	2V	2-4	3-6	1-2--4-5----9--11-
295	1--3--5--7-8--10--	2V	2-4	3-5	-2--4--6---9--11-12
296	--3--5--7-8--10--12	2V	2-4	3-5	1-2--4--6---9--11-
297	1-2-3-----8---11-12	2V	4-6	1-2	---4-5-6-7--9-10--
298	1-2------8--10-11-12	2V	4-6	1-2	--3-4-5-6-7--9---
299	1-2-3--5---8----12	2V	4-6	1-5	---4--6-7--9-10-11-
300	1--3--5---8---11-12	2V	4-6	1-5	-2--4--6-7--9-10--
301	1-2-3-----8--10--12	2V	4-6	1-3	---4-5-6-7--9--11-
302	1--3-----8--10-11-12	2V	4-6	1-3	-2--4-5-6-7--9---
303	1-2-3--5---8---11-	2V	4-6	2-5	---4--6-7--9-10--12
304	-2-3--5---8---11-12	2V	4-6	2-5	1---4--6-7--9-10--
305	1-2-3-----8--10-11-	2V	4-6	2-3	---4-5-6-7--9---12
306	-2-3-----8--10-11-12	2V	4-6	2-3	1---4-5-6-7--9---
307	1-2-3--5---8--10--	2V	4-6	3-5	---4--6-7--9--11-12
308	-2-3--5---8--10--12	2V	4-6	3-5	1---4--6-7--9--11-
309	1-2-3----7----11-12	2V	4-5	1-2	---4-5-6--8-9-10--
310	1-2-----7---10-11-12	2V	4-5	1-2	--3-4-5-6--8-9---
311	1-2-3---6-7-----12	2V	4-5	1-6	---4-5---8-9-10-11-
312	1--3---6-7----11-12	2V	4-5	1-6	-2--4-5---8-9-10--
313	1-2-3----7---10--12	2V	4-5	1-3	---4-5-6--8-9--11-
314	1--3----7---10-11-12	2V	4-5	1-3	-2--4-5-6--8-9---
315	1-2-3---6-7----11-	2V	4-5	2-6	---4-5---8-9-10--12
316	-2-3---6-7----11-12	2V	4-5	2-6	1---4-5---8-9-10--
317	1-2-3----7---10-11-	2V	4-5	2-3	---4-5-6--8-9---12
318	-2-3----7---10-11-12	2V	4-5	2-3	1---4-5-6--8-9---
319	1-2-3---6-7---10--	2V	4-5	3-6	---4-5---8-9--11-12
320	-2-3---6-7---10--12	2V	4-5	3-6	1---4-5---8-9--11-
321	1-2---5--7----11-12	2V	3-4	1-2	--3-4--6--8-9-10--

322	1-2-----7-8---11-12	2V	3-4	1-2	--3-4-5-6---9-10--
323	1-2----6-7-8----12	2V	3-4	1-6	--3-4-5----9-10-11-
324	1-----6-7-8---11-12	2V	3-4	1-6	-2-3-4-5----9-10--
325	1-2---5--7-8----12	2V	3-4	1-5	--3-4--6---9-10-11-
326	1----5--7-8---11-12	2V	3-4	1-5	-2-3-4--6---9-10--
327	1-2----6-7-8---11-	2V	3-4	2-6	--3-4-5----9-10--12
328	-2----6-7-8---11-12	2V	3-4	2-6	1--3-4-5----9-10--
329	1-2---5--7-8---11-	2V	3-4	2-5	--3-4--6---9-10--12
330	-2---5--7-8---11-12	2V	3-4	2-5	1--3-4--6---9-10--
331	1-2---5-6-7-8----	2V	3-4	5-6	--3-4-----9-10-11-12
332	-2---5-6-7-8----12	2V	3-4	5-6	1--3-4-----9-10-11-
333	--3-4--6-7-8-9---	2V	1-2	4-6	1-2---5-----10-11-12
334	---4--6-7-8-9-10--	2V	1-2	4-6	1-2-3--5------11-12
335	--3-4-5--7-8-9---	2V	1-2	4-5	1-2----6----10-11-12
336	---4-5--7-8-9-10--	2V	1-2	4-5	1-2-3---6-----11-12
337	--3-4-5--7--9-10--	2V	1-2	3-4	1-2----6--8---11-12
338	--3-4---7-8-9-10--	2V	1-2	3-4	1-2---5-6-----11-12
339	--3--5-6-7-8-9---	2V	1-2	5-6	1-2--4------10-11-12
340	----5-6-7-8-9-10--	2V	1-2	5-6	1-2-3-4-------11-12
341	--3-4--6-7-8--10--	2V	1-2	3-6	1-2---5----9--11-12
342	--3---6-7-8-9-10--	2V	1-2	3-6	1-2--4-5------11-12
343	--3-4-5--7-8--10--	2V	1-2	3-5	1-2----6---9--11-12
344	--3--5--7-8-9-10--	2V	1-2	3-5	1-2--4--6-----11-12
345	-2-3-4----8-9--11-	2V	1-6	2-4	1----5-6-7---10--12
346	-2--4----8-9-10-11-	2V	1-6	2-4	1--3--5-6-7-----12
347	-2-3-4-5---8-9---	2V	1-6	4-5	1-----6-7---10-11-12
348	--3-4-5---8-9--11-	2V	1-6	4-5	1-2----6-7---10--12
349	-2-3-4----8-9-10--	2V	1-6	3-4	1----5-6-7----11-12
350	--3-4----8-9-10-11-	2V	1-6	3-4	1-2---5-6-7-----12
351	-2-3--5---8-9--11-	2V	1-6	2-5	1---4--6-7---10--12
352	-2---5---8-9-10-11-	2V	1-6	2-5	1--3-4--6-7-----12
353	-2-3-4----8--10-11-	2V	1-6	2-3	1----5-6-7--9---12
354	-2-3-----8-9-10-11-	2V	1-6	2-3	1---4-5-6-7-----12
355	-2-3--5---8-9-10--	2V	1-6	3-5	1---4--6-7----11-12
356	--3--5---8-9-10-11-	2V	1-6	3-5	1-2--4--6-7-----12
357	-2-3-4---7--9--11-	2V	1-5	2-4	1----5-6--8--10--12
358	-2--4---7--9-10-11-	2V	1-5	2-4	1--3--5-6--8----12
359	-2-3-4--6-7--9---	2V	1-5	4-6	1----5---8--10-11-12
360	--3-4--6-7--9--11-	2V	1-5	4-6	1-2---5---8--10--12
361	-2-3-4---7--9-10--	2V	1-5	3-4	1----5-6--8---11-12
362	--3-4---7--9-10-11-	2V	1-5	3-4	1-2---5-6--8----12
363	-2-3---6-7--9--11-	2V	1-5	2-6	1---4-5---8--10--12
364	-2----6-7--9-10-11-	2V	1-5	2-6	1--3-4-5---8----12
365	-2-3-4---7---10-11-	2V	1-5	2-3	1----5-6--8-9---12
366	-2-3----7--9-10-11-	2V	1-5	2-3	1---4-5-6--8----12
367	-2-3---6-7--9-10--	2V	1-5	3-6	1---4-5---8---11-12
368	--3---6-7--9-10-11-	2V	1-5	3-6	1-2--4-5---8----12
369	-2--4-5--7--9--11-	2V	1-3	2-4	1--3---6--8--10--12
370	-2--4---7-8-9--11-	2V	1-3	2-4	1--3--5-6----10--12
371	-2--4--6-7-8-9---	2V	1-3	4-6	1--3--5-----10-11-12
372	---4--6-7-8-9--11-	2V	1-3	4-6	1-2-3--5-----10--12
373	-2--4-5--7-8-9---	2V	1-3	4-5	1--3---6----10-11-12
374	---4-5--7-8-9--11-	2V	1-3	4-5	1-2-3---6----10--12
375	-2--4--6-7-8---11-	2V	1-3	2-6	1--3--5----9-10--12
376	-2----6-7-8-9--11-	2V	1-3	2-6	1--3-4-5-----10--12
377	-2--4-5--7-8---11-	2V	1-3	2-5	1--3---6---9-10--12
378	-2---5--7-8-9--11-	2V	1-3	2-5	1--3-4--6----10--12
379	-2---5-6-7-8-9---	2V	1-3	5-6	1--3-4------10-11-12
380	----5-6-7-8-9--11-	2V	1-3	5-6	1-2-3-4------10--12
381	1--3-4----8-9---12	2V	2-6	1-4	-2---5-6-7---10-11-
382	1---4----8-9-10--12	2V	2-6	1-4	-2-3--5-6-7----11-
383	1--3-4-5---8-9---	2V	2-6	4-5	-2----6-7---10-11-12
384	--3-4-5---8-9---12	2V	2-6	4-5	1-2----6-7---10-11-
385	1--3-4----8-9-10--	2V	2-6	3-4	-2---5-6-7----11-12
386	--3-4----8-9-10--12	2V	2-6	3-4	1-2---5-6-7----11-
387	1--3--5---8-9---12	2V	2-6	1-5	-2--4--6-7---10-11-
388	1----5---8-9-10--12	2V	2-6	1-5	-2-3-4--6-7----11-
389	1--3-4----8--10--12	2V	2-6	1-3	-2---5-6-7--9--11-
390	1--3-----8-9-10--12	2V	2-6	1-3	-2--4-5-6-7----11-
391	1--3--5---8-9-10--	2V	2-6	3-5	-2--4--6-7----11-12
392	--3--5---8-9-10--12	2V	2-6	3-5	1-2--4--6-7----11-
393	1--3-4---7--9---12	2V	2-5	1-4	-2---5-6--8--10-11-
394	1---4---7--9-10--12	2V	2-5	1-4	-2-3--5-6--8---11-
395	1--3-4--6-7--9---	2V	2-5	4-6	-2---5---8--10-11-12
396	--3-4--6-7--9---12	2V	2-5	4-6	1-2---5---8--10-11-
397	1--3-4---7--9-10--	2V	2-5	3-4	-2---5-6--8---11-12
398	--3-4---7--9-10--12	2V	2-5	3-4	1-2---5-6--8---11-
399	1--3---6-7--9---12	2V	2-5	1-6	-2--4-5---8--10-11-
400	1-----6-7--9-10--12	2V	2-5	1-6	-2-3-4-5---8---11-
401	1--3-4---7---10--12	2V	2-5	1-3	-2---5-6--8-9--11-
402	1--3----7--9-10--12	2V	2-5	1-3	-2--4-5-6--8---11-
403	1--3---6-7--9-10--	2V	2-5	3-6	-2--4-5---8---11-12
404	--3---6-7--9-10--12	2V	2-5	3-6	1-2--4-5---8---11-

405	1---4-5--7--9---12	2V	2-3	1-4	-2-3---6--8--10-11-
406	1---4---7-8-9---12	2V	2-3	1-4	-2-3--5-6----10-11-
407	1---4--6-7-8-9---	2V	2-3	4-6	-2-3--5-----10-11-12
408	---4--6-7-8-9---12	2V	2-3	4-6	1-2-3--5-----10-11-
409	1---4-5--7-8-9---	2V	2-3	4-5	-2-3---6----10-11-12
410	---4-5--7-8-9---12	2V	2-3	4-5	1-2-3---6----10-11-
411	1---4--6-7-8----12	2V	2-3	1-6	-2-3--5----9-10-11-
412	1-----6-7-8-9---12	2V	2-3	1-6	-2-3-4-5-----10-11-
413	1---4-5--7-8----12	2V	2-3	1-5	-2-3---6---9-10-11-
414	1----5--7-8-9---12	2V	2-3	1-5	-2-3-4--6----10-11-
415	1----5-6-7-8-9---	2V	2-3	5-6	-2-3-4------10-11-12
416	----5-6-7-8-9---12	2V	2-3	5-6	1-2-3-4------10-11-
417	1-2-3-4-----9---12	2V	5-6	1-4	----5-6-7-8--10-11-
418	1--3-4-----9--11-12	2V	5-6	1-4	-2---5-6-7-8--10--
419	1-2-3-4-----9--11-	2V	5-6	2-4	----5-6-7-8--10--12
420	-2-3-4-----9--11-12	2V	5-6	2-4	1----5-6-7-8--10--
421	1-2-3-4-----9-10--	2V	5-6	3-4	----5-6-7-8---11-12
422	-2-3-4-----9-10--12	2V	5-6	3-4	1----5-6-7-8---11-
423	1-2-3------9--11-12	2V	5-6	1-2	---4-5-6-7-8--10--
424	1-2-------9-10-11-12	2V	5-6	1-2	--3-4-5-6-7-8----
425	1-2-3------9-10--12	2V	5-6	1-3	---4-5-6-7-8---11-
426	1--3------9-10-11-12	2V	5-6	1-3	-2--4-5-6-7-8----
427	1-2-3------9-10-11-	2V	5-6	2-3	---4-5-6-7-8----12
428	-2-3------9-10-11-12	2V	5-6	2-3	1---4-5-6-7-8----
429	1-2--4----8-9---12	2V	3-6	1-4	--3--5-6-7---10-11-
430	1---4----8-9--11-12	2V	3-6	1-4	-2-3--5-6-7---10--
431	1-2--4----8-9--11-	2V	3-6	2-4	--3--5-6-7---10--12
432	-2--4----8-9--11-12	2V	3-6	2-4	1--3--5-6-7---10--
433	1-2--4-5---8-9---	2V	3-6	4-5	--3---6-7---10-11-12
434	-2--4-5---8-9---12	2V	3-6	4-5	1--3---6-7---10-11-
435	1-2--4----8---11-12	2V	3-6	1-2	--3--5-6-7--9-10--
436	1-2------8-9--11-12	2V	3-6	1-2	--3-4-5-6-7---10--
437	1-2---5---8-9---12	2V	3-6	1-5	--3-4--6-7---10-11-
438	1----5---8-9--11-12	2V	3-6	1-5	-2-3-4--6-7---10--
439	1-2---5---8-9--11-	2V	3-6	2-5	--3-4--6-7---10--12
440	-2---5---8-9--11-12	2V	3-6	2-5	1--3-4--6-7---10--
441	1-2--4---7--9---12	2V	3-5	1-4	--3--5-6--8--10-11-
442	1---4---7--9--11-12	2V	3-5	1-4	-2-3--5-6--8--10--
443	1-2--4---7--9--11-	2V	3-5	2-4	--3--5-6--8--10--12
444	-2--4---7--9--11-12	2V	3-5	2-4	1--3--5-6--8--10--
445	1-2--4--6-7--9---	2V	3-5	4-6	--3--5---8--10-11-12
446	-2--4--6-7--9---12	2V	3-5	4-6	1--3--5---8--10-11-
447	1-2--4---7----11-12	2V	3-5	1-2	--3--5-6--8-9-10--
448	1-2-----7--9--11-12	2V	3-5	1-2	--3-4-5-6--8--10--
449	1-2----6-7--9---12	2V	3-5	1-6	--3-4-5---8--10-11-
450	1-----6-7--9--11-12	2V	3-5	1-6	-2-3-4-5---8--10--
451	1-2----6-7--9--11-	2V	3-5	2-6	--3-4-5---8--10--12
452	-2----6-7--9--11-12	2V	3-5	2-6	1--3-4-5---8--10--
**Three Vacancies**					
453	1----5-6-7-8----12	3V	2-3-4	1-5-6	-2-3-4-----9-10-11-
454	--3--5-6-7-8--10--	3V	1-2-4	3-5-6	1-2--4-----9--11-12
455	-2-3---6-7---10-11-	3V	1-4-5	2-3-6	1---4-5---8-9---12
456	1-2---5---8---11-12	3V	3-4-6	1-2-5	--3-4--6-7--9-10--
457	1-2-3-------10-11-12	3V	4-5-6	1-2-3	---4-5-6-7-8-9---
458	-2--4-5---8-9--11-	3V	1-3-6	2-4-5	1--3---6-7---10--12
459	1-2----6-7----11-12	3V	3-4-5	1-2-6	--3-4-5---8-9-10--
460	1---4--6-7--9---12	3V	2-3-5	1-4-6	-2-3--5---8--10-11-
461	-2--4--6-7--9--11-	3V	1-3-5	2-4-6	1--3--5---8--10--12
462	1--3-4-----9-10--12	3V	2-5-6	1-3-4	-2---5-6-7-8---11-
463	1-2--4-----9--11-12	3V	3-5-6	1-2-4	--3--5-6-7-8--10--
464	1---4-5---8-9---12	3V	2-3-6	1-4-5	-2-3---6-7---10-11-
465	-2-3-4-----9-10-11-	3V	1-5-6	2-3-4	1----5-6-7-8----12
466	---4-5-6-7-8-9---	3V	1-2-3	4-5-6	1-2-3-------10-11-12
467	--3-4--6-7--9-10--	3V	1-2-5	3-4-6	1-2---5---8---11-12
468	--3-4-5---8-9-10--	3V	1-2-6	3-4-5	1-2----6-7----11-12
469	1--3---6-7---10--12	3V	2-4-5	1-3-6	-2--4-5---8-9--11-
470	1--3--5---8--10--12	3V	2-4-6	1-3-5	-2--4--6-7--9--11-
471	-2---5-6-7-8---11-	3V	1-3-4	2-5-6	1--3-4-----9-10--12
472	-2-3--5---8--10-11-	3V	1-4-6	2-3-5	1---4--6-7--9---12

Figure S40. (S/N) analysis of 0-nt to 6-nt Variant EREs in ER ChIPSeq Peaks (924 Half-Site Groups)

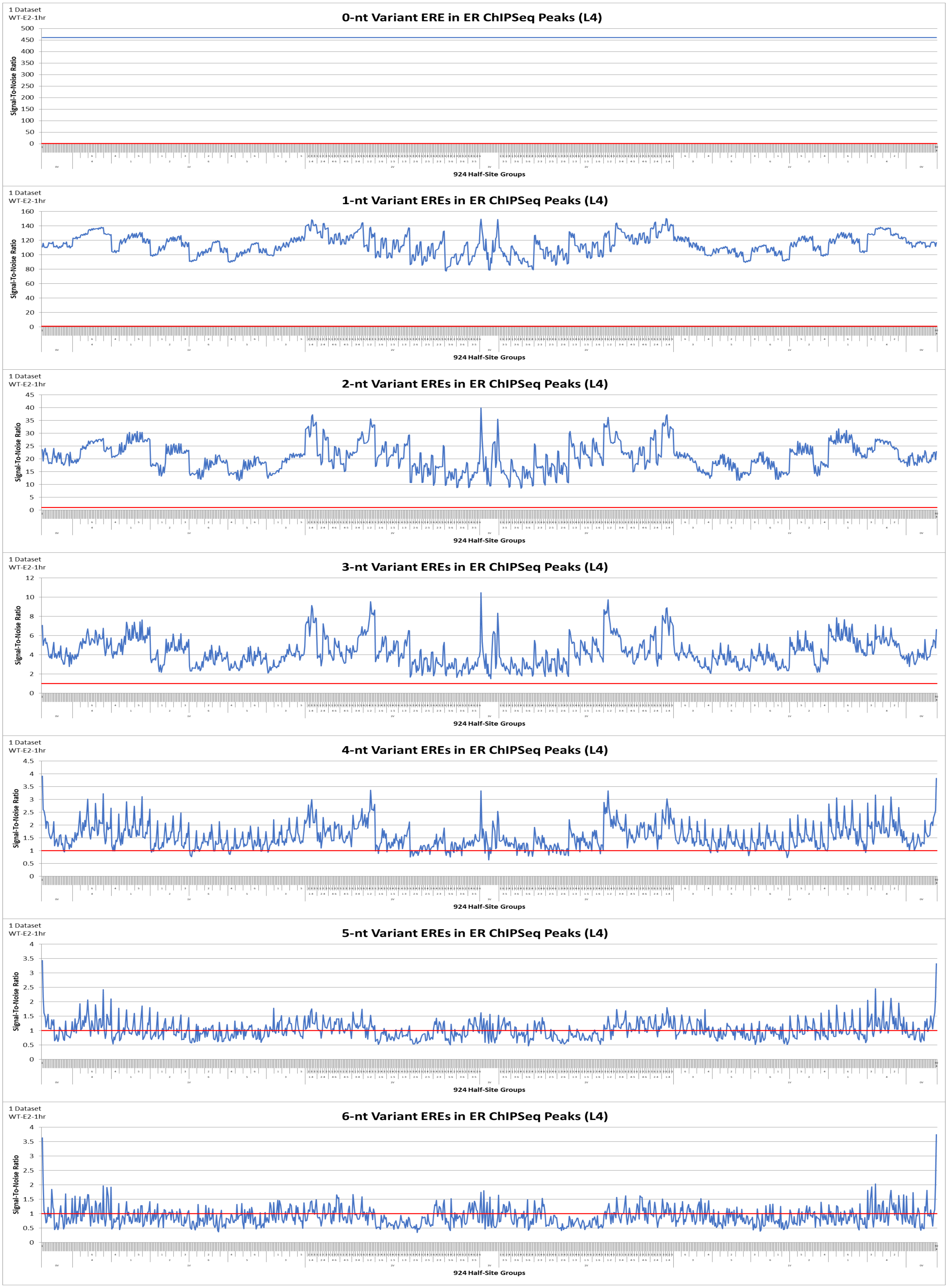

Figure S41. (S/N) analysis of 0-nt to 6-nt Variant HREs in AR ChIPSeq Peaks (924 Half-Site Groups)

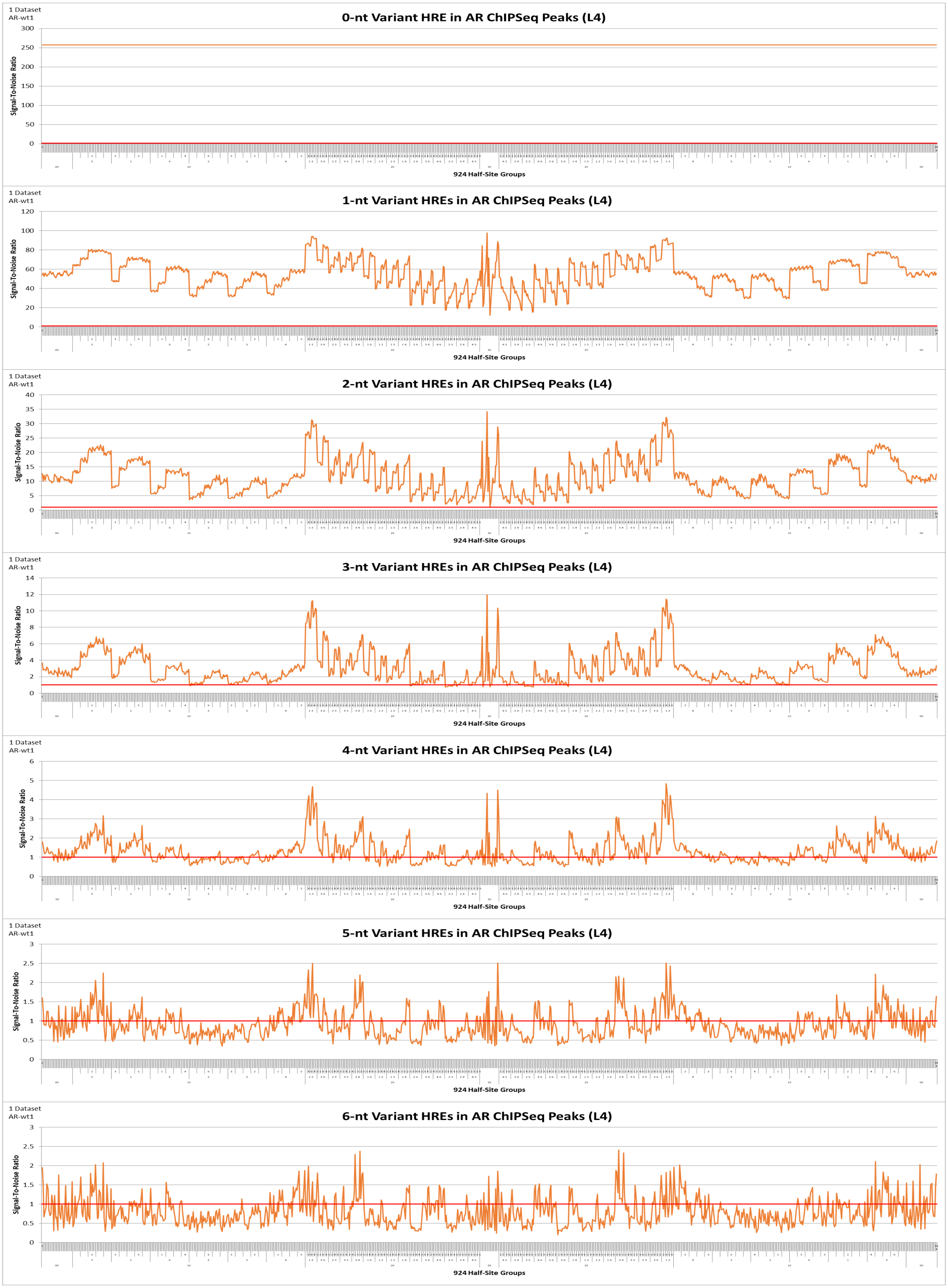

Figure S42. (S/N) analysis of 0-nt to 6-nt Variant HREs in GR ChIPSeq Peaks (924 Half-Site Groups)

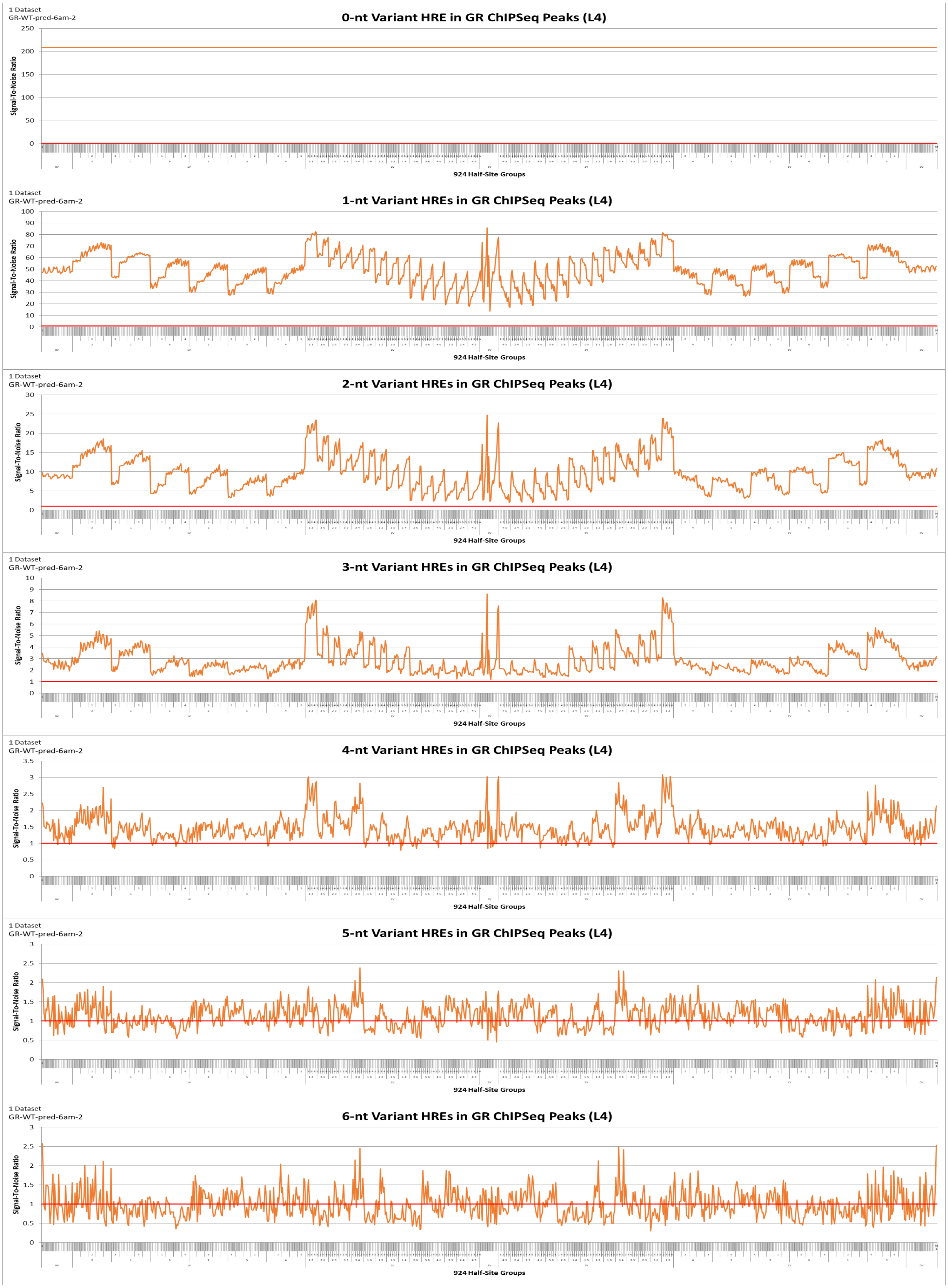

Figure S43. (S/N) analysis of 0-nt to 6-nt Variant HREs in PR ChIPSeq Peaks (924 Half-Site Groups)

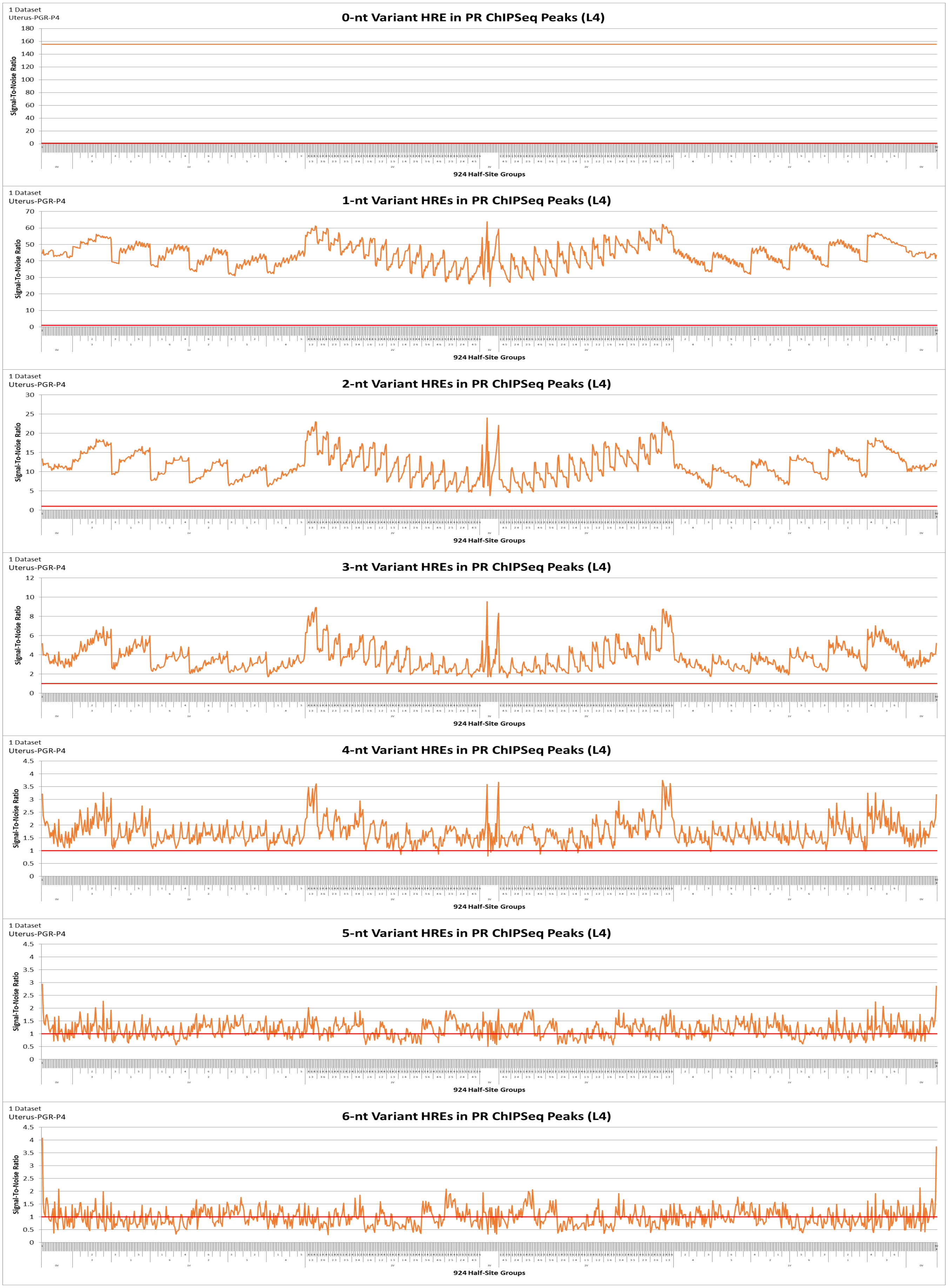

Figure S44. (S/N) analysis of 1-nt Variant EREs in ER ChIPSeq Peaks (Variant Position)

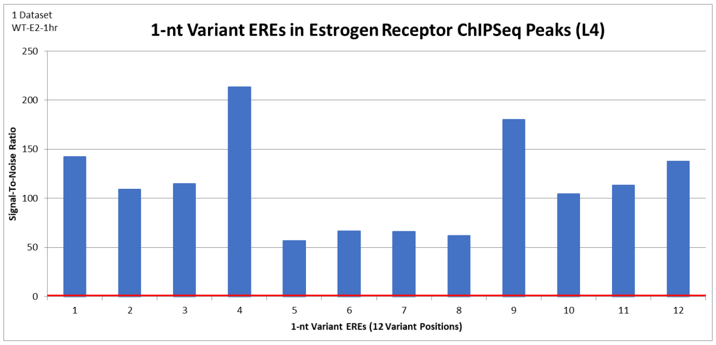

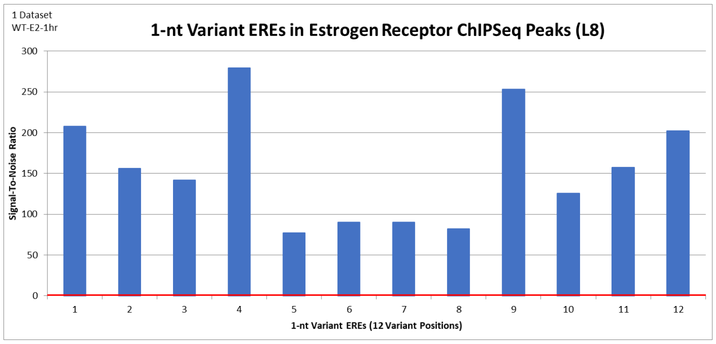

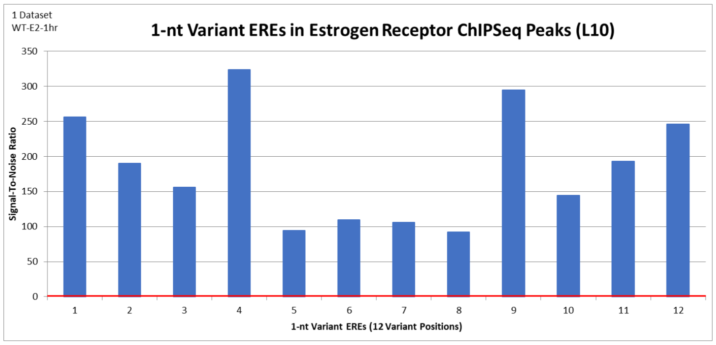

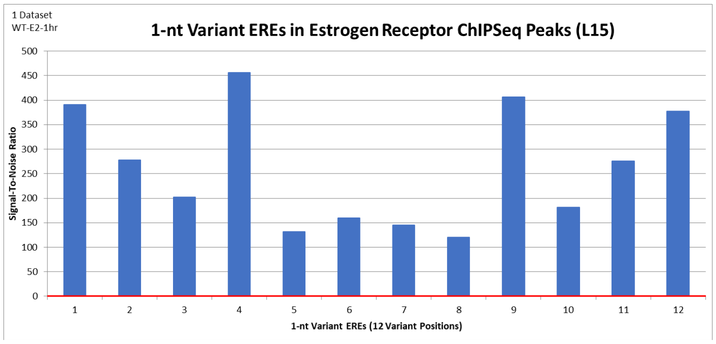

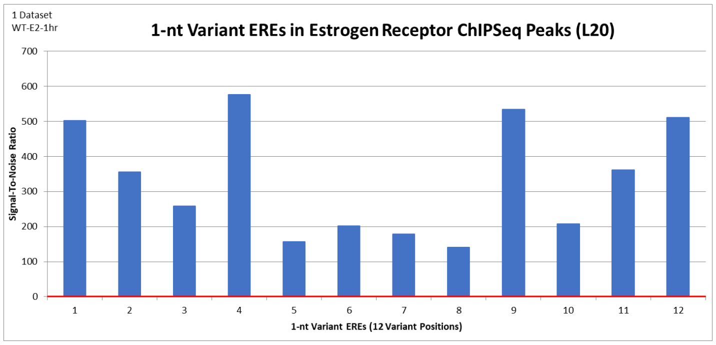

Figure S45. (S/N) analysis of 2-nt Variant EREs in ER ChIPSeq Peaks (Variant Position)

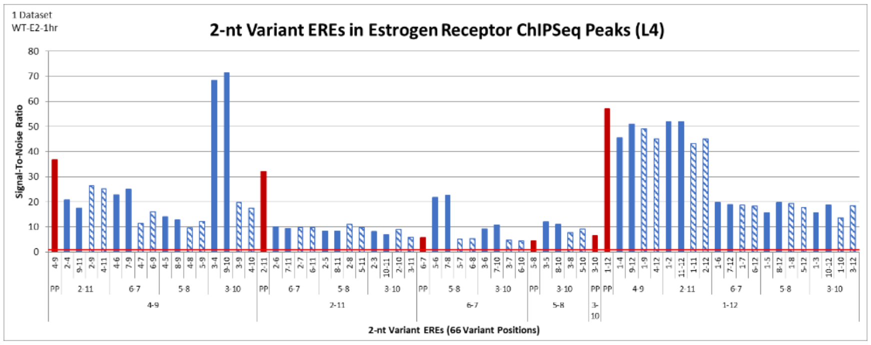

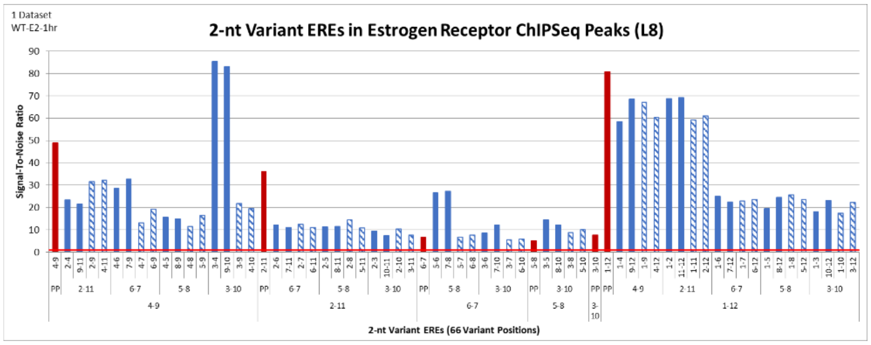

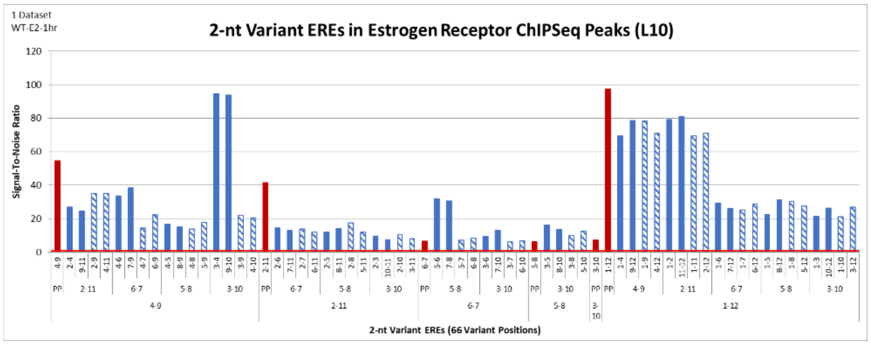

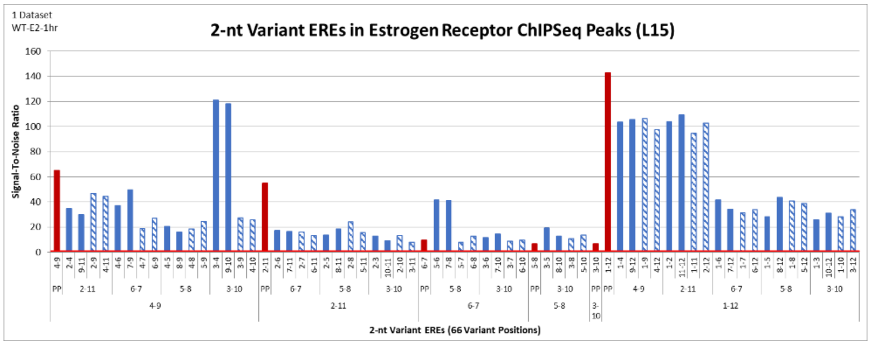

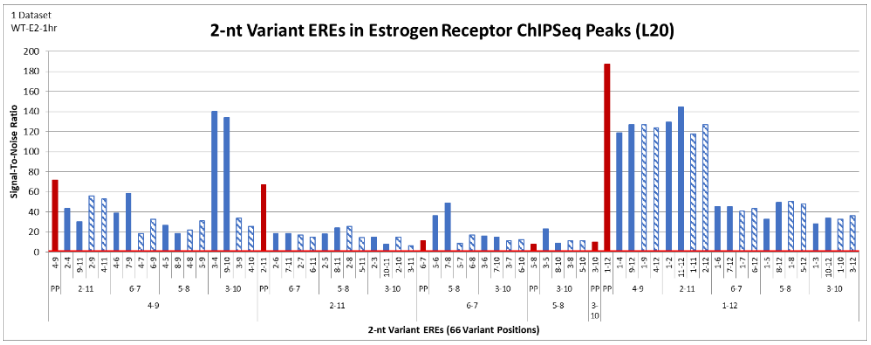

Figure S46. (S/N) analysis of 3-nt Variant EREs in ER ChIPSeq Peaks (Variant Position)

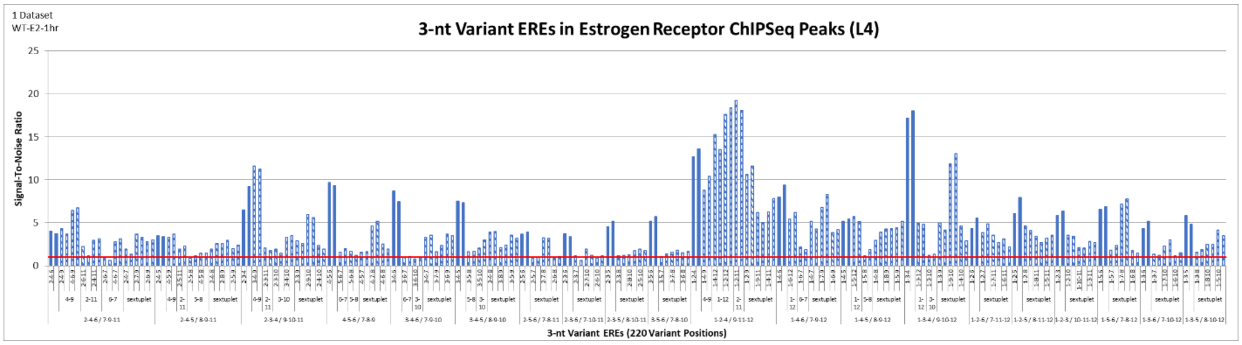

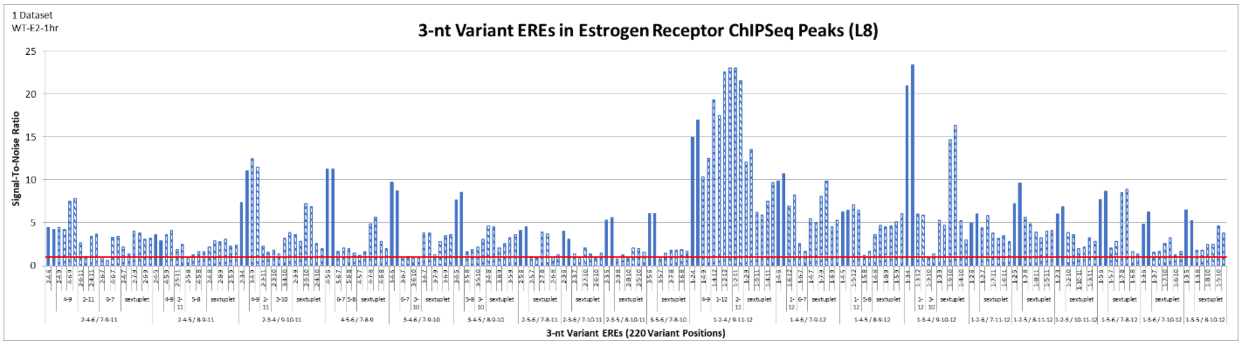

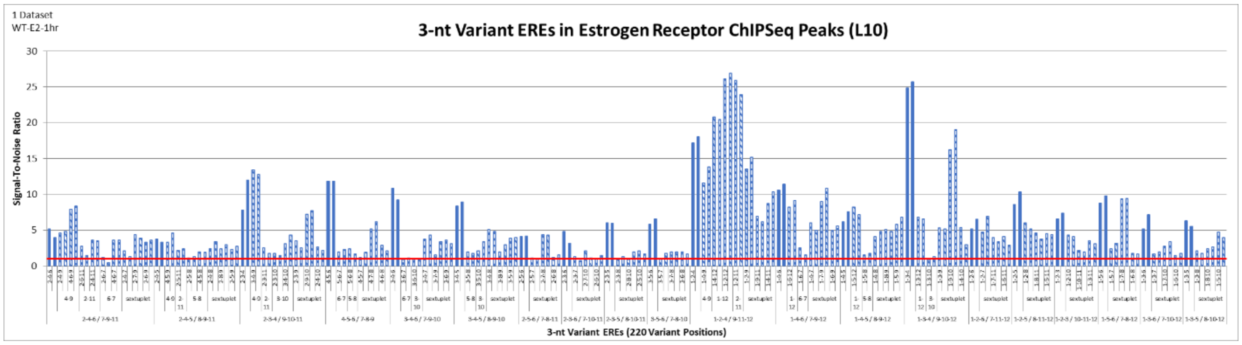

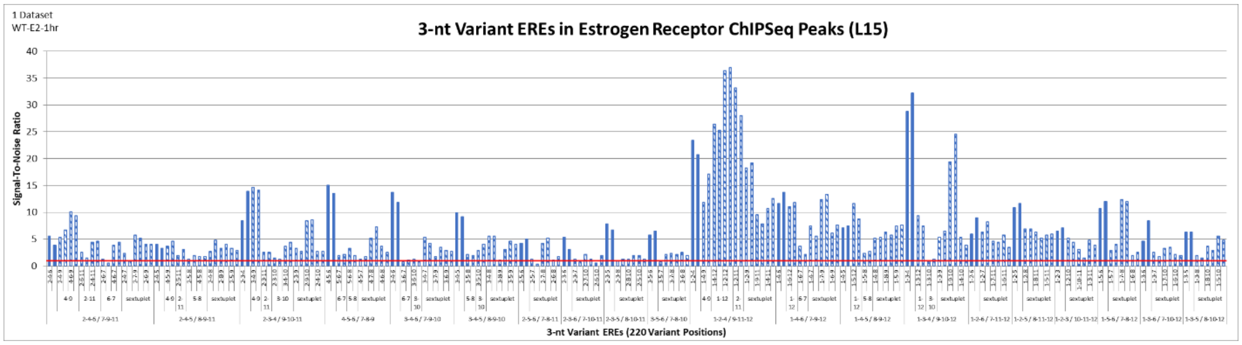

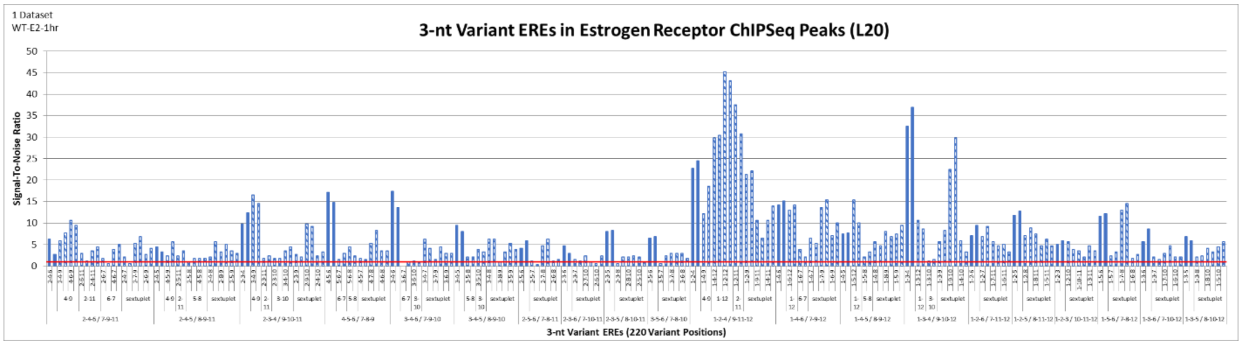

Figure S47. (S/N) analysis of 4-nt Variant EREs in ER ChIPSeq Peaks (Variant Position)

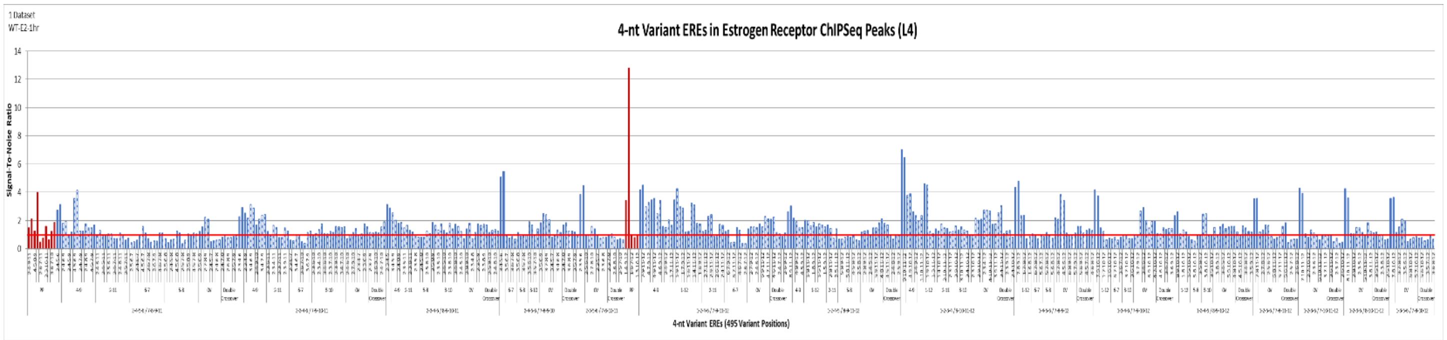

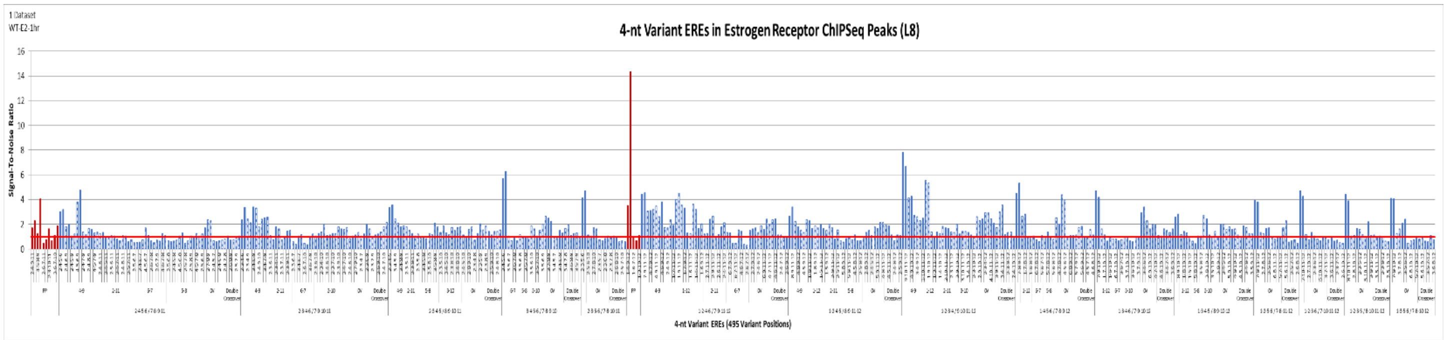

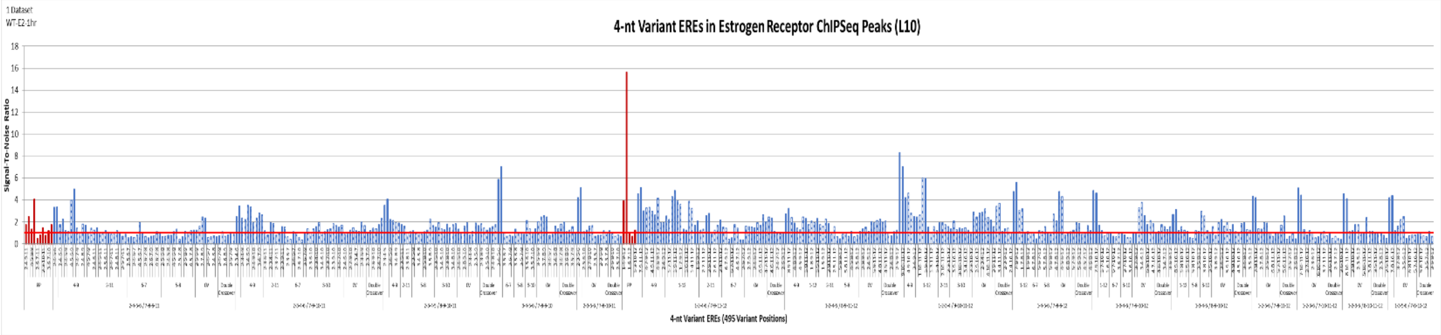

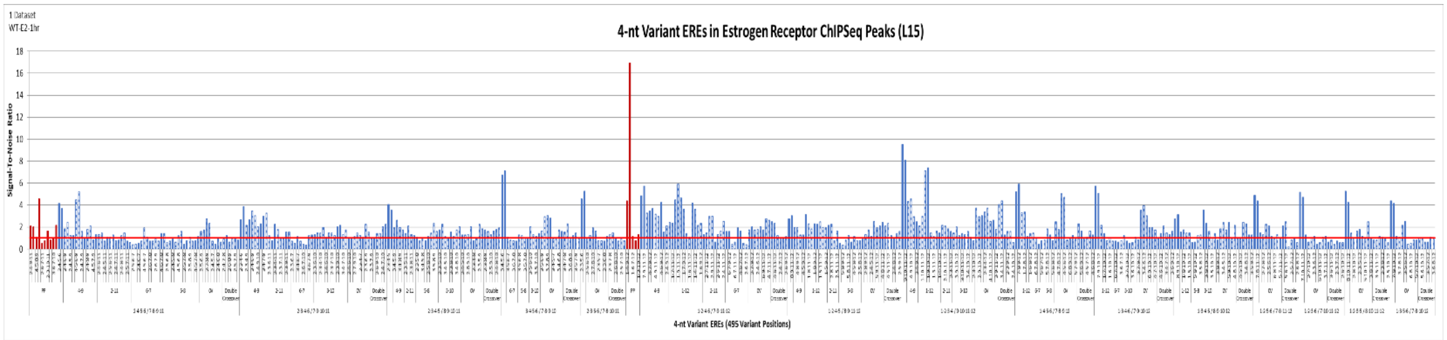

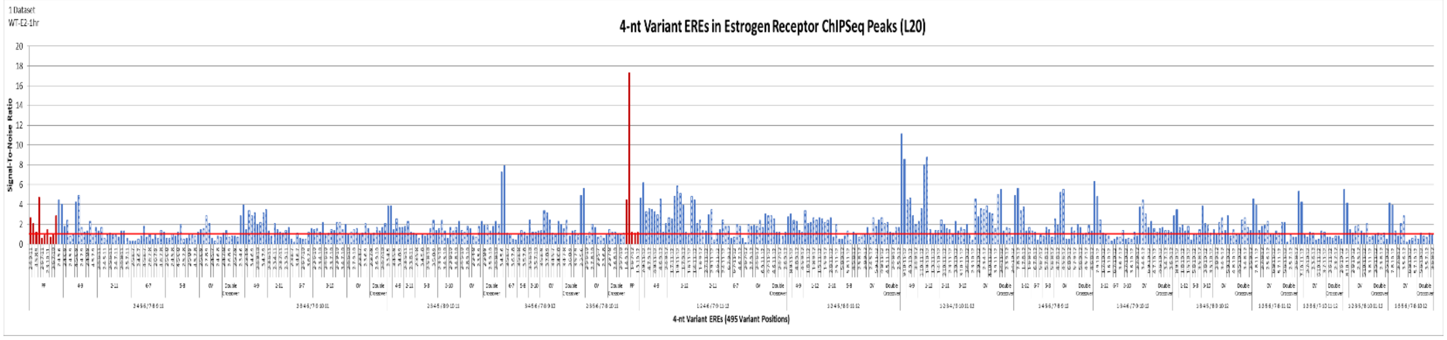

Figure S48. (S/N) analysis of 5-nt Variant EREs in ER ChIPSeq Peaks (Variant Position)

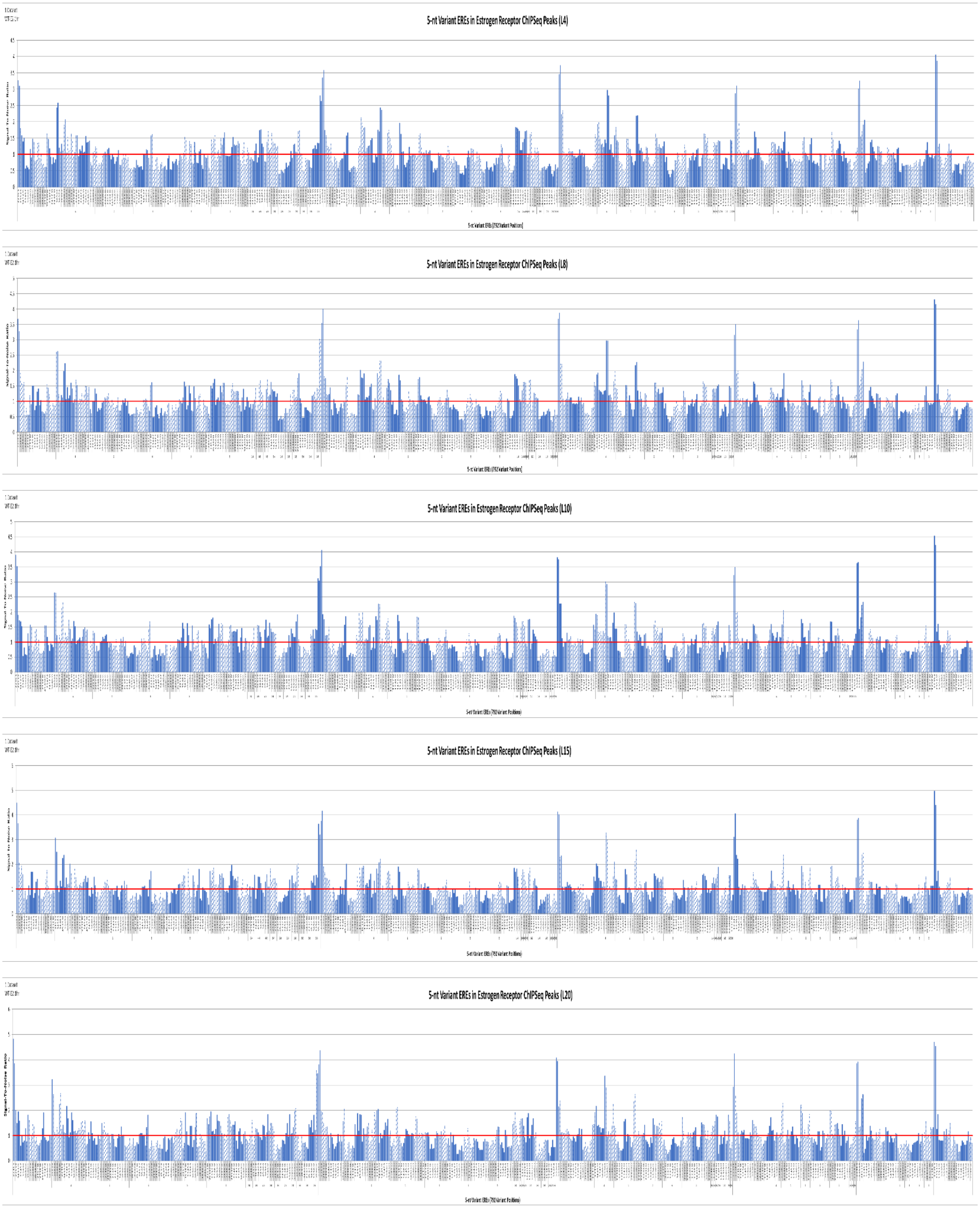

Figure S49. (S/N) analysis of 6-nt Variant EREs in ER ChIPSeq Peaks (Variant Position)

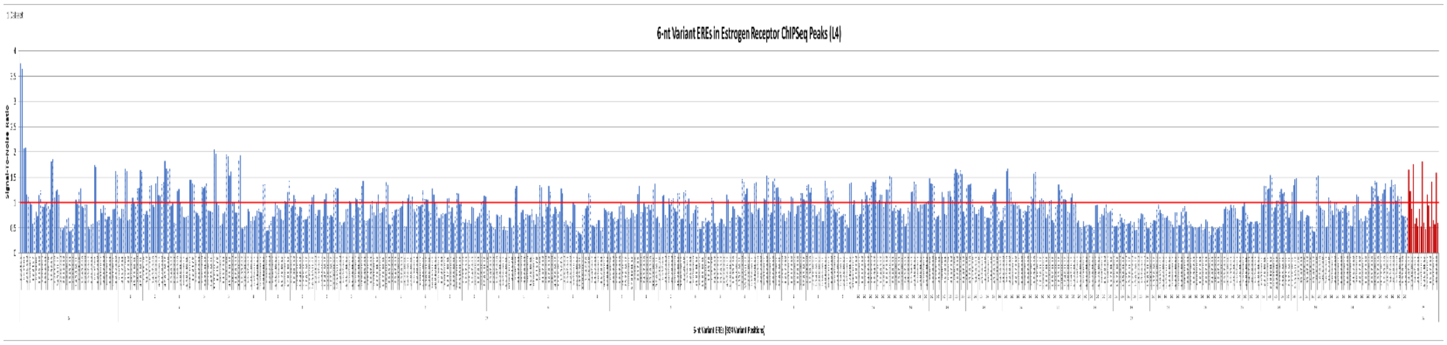

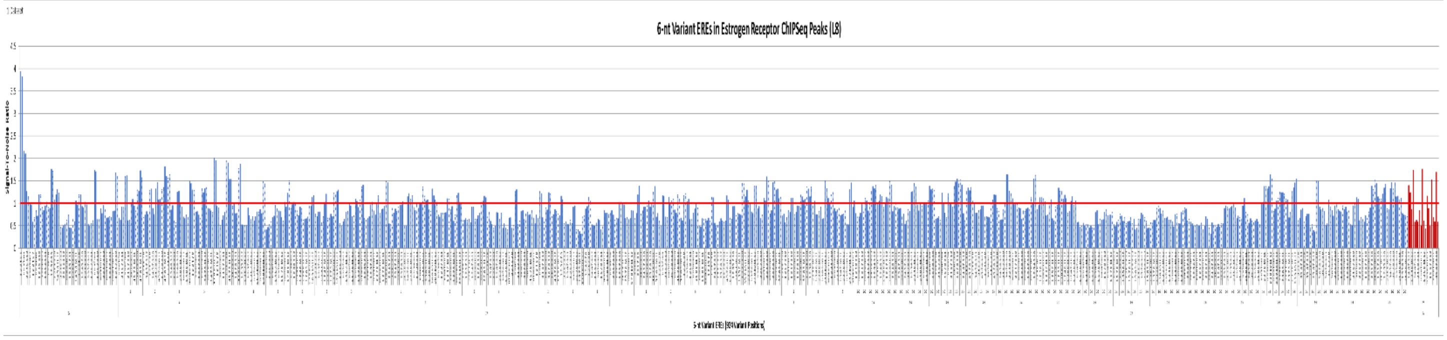

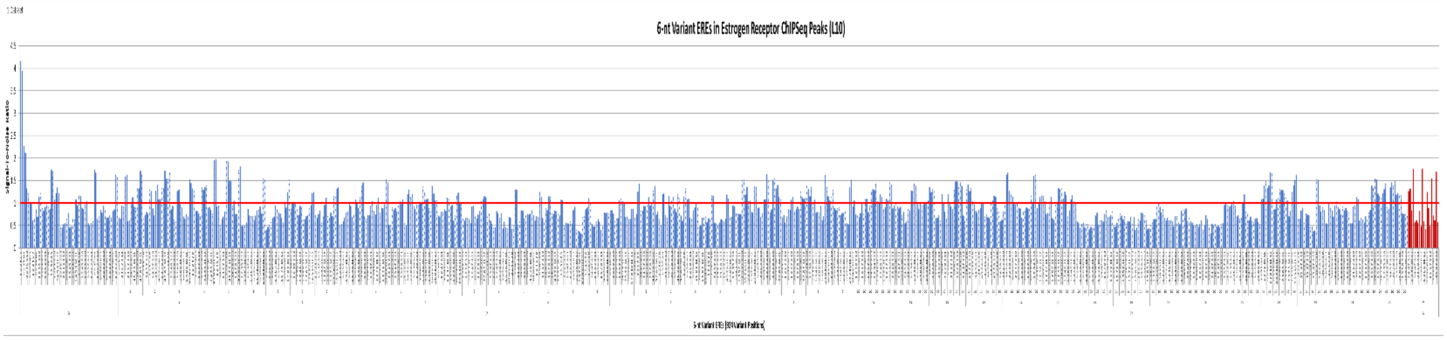

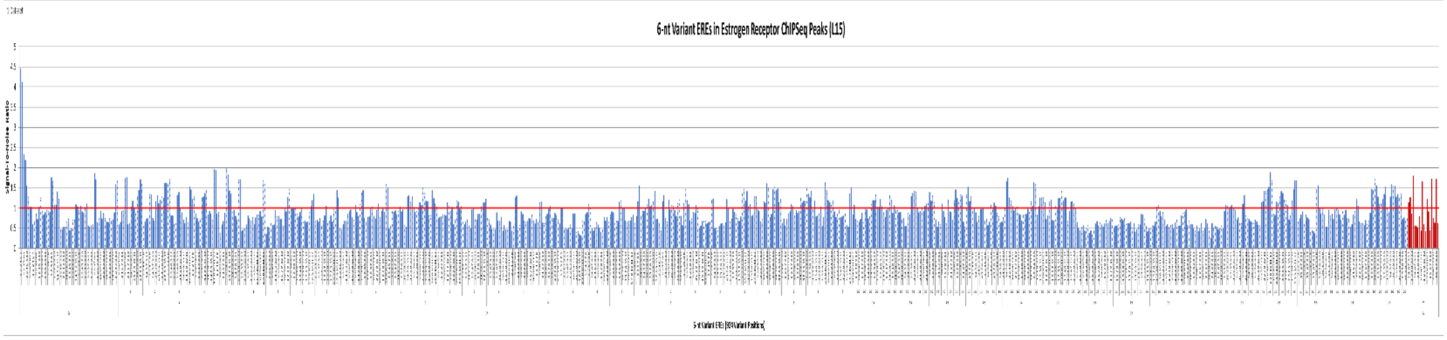

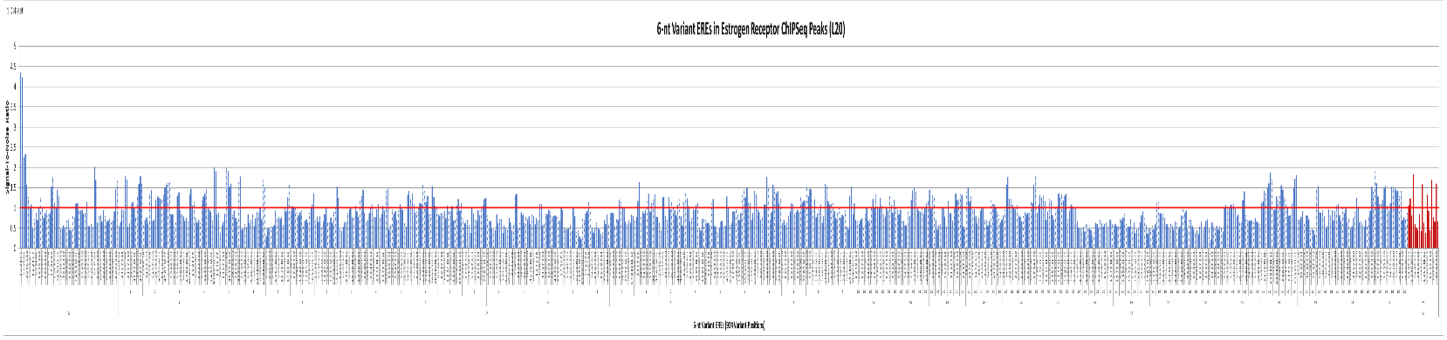

Figure S50. (S/N) analysis of 1-nt Variant HREs in KR ChIPSeq Peaks (Variant Position)

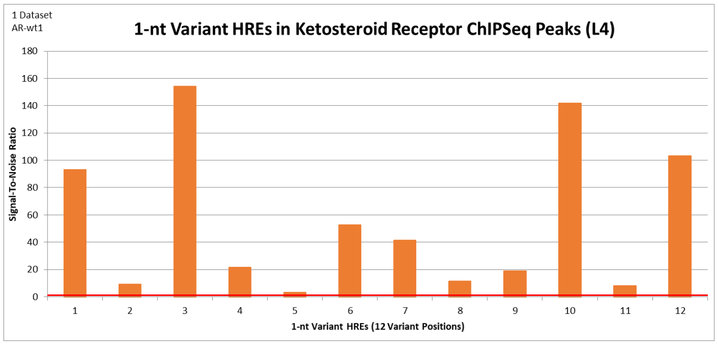

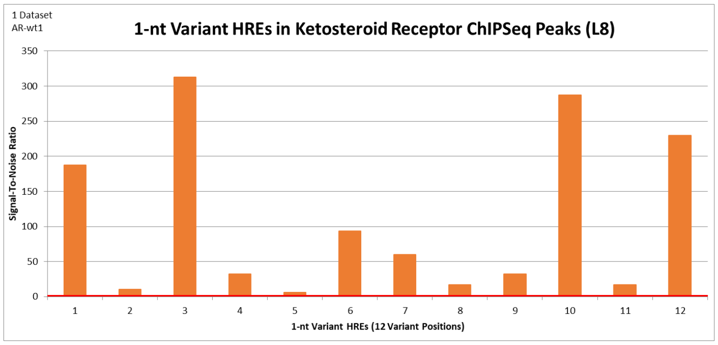

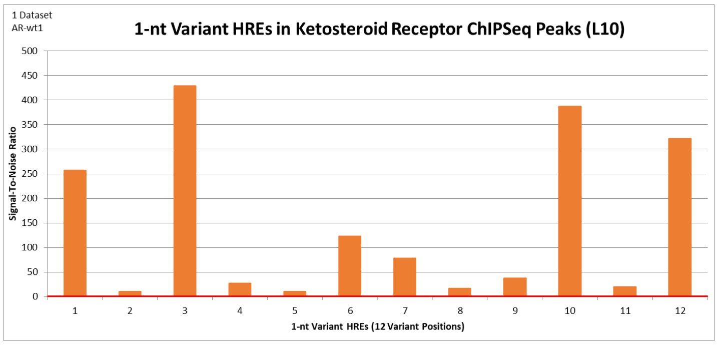

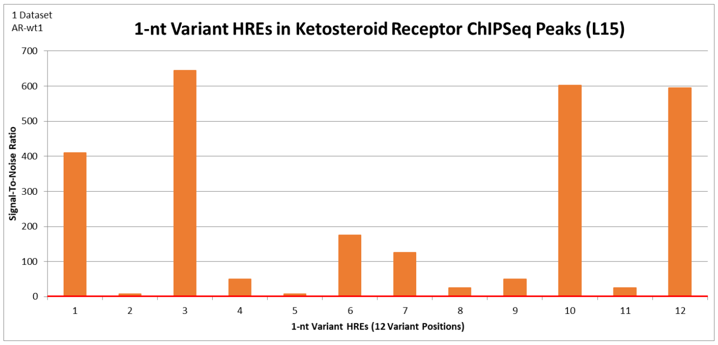

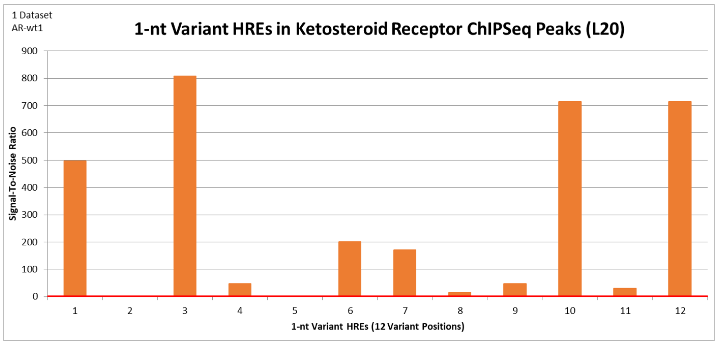

Figure S51. (S/N) analysis of 2-nt Variant HREs in KR ChIPSeq Peaks (Variant Position)

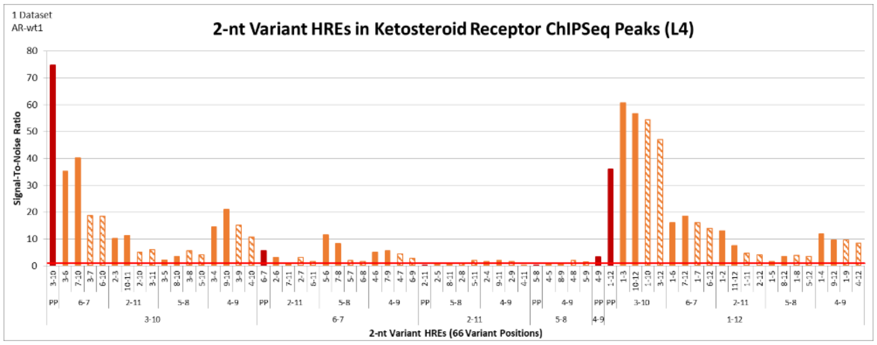

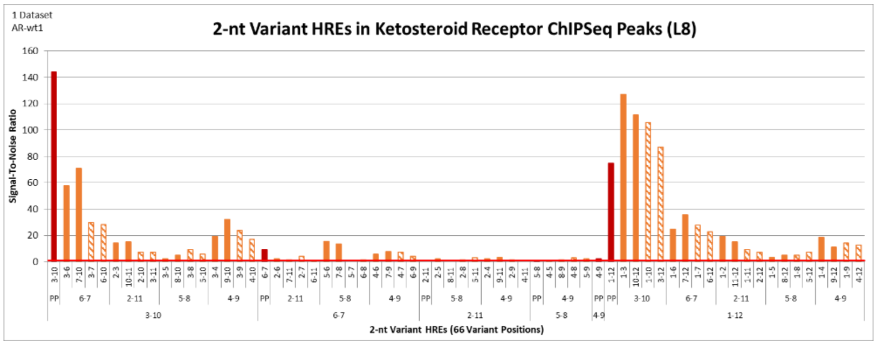

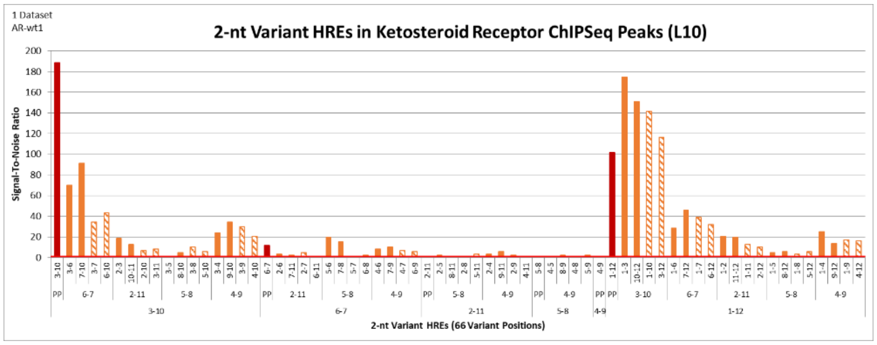

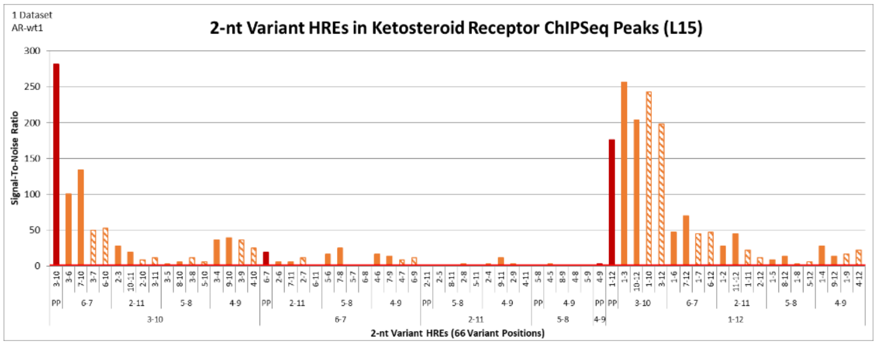

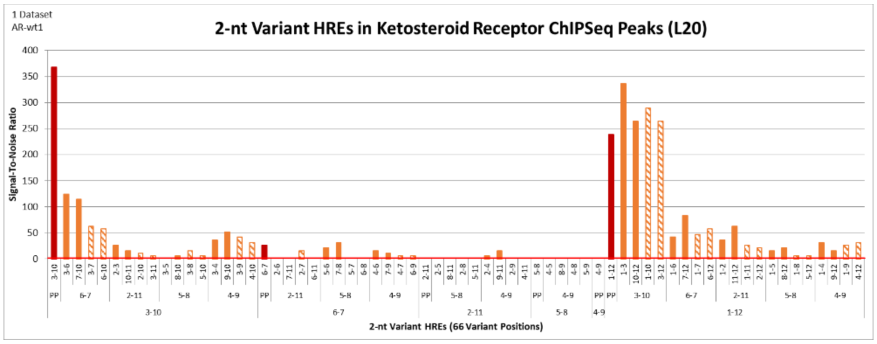

Figure S52. (S/N) analysis of 3-nt Variant HREs in KR ChIPSeq Peaks (Variant Position)

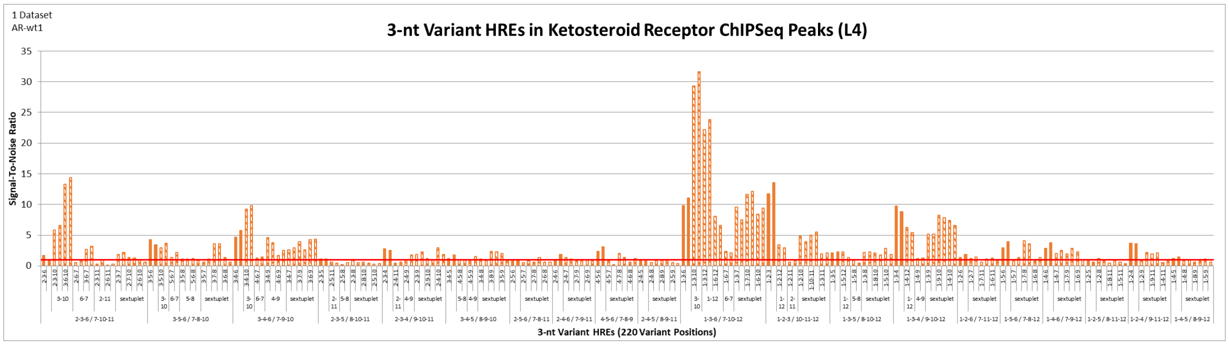

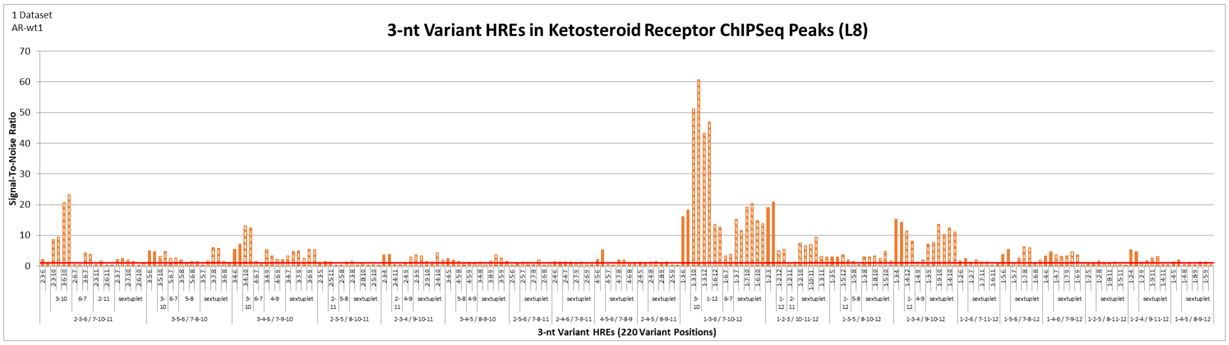

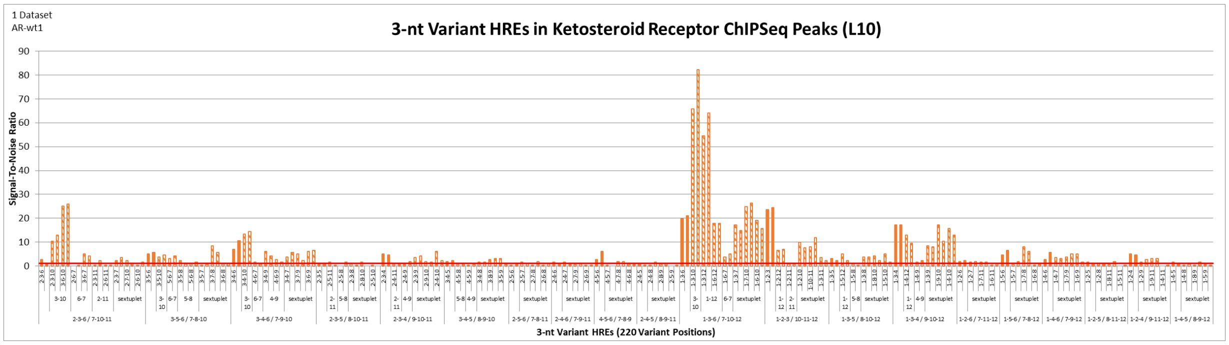

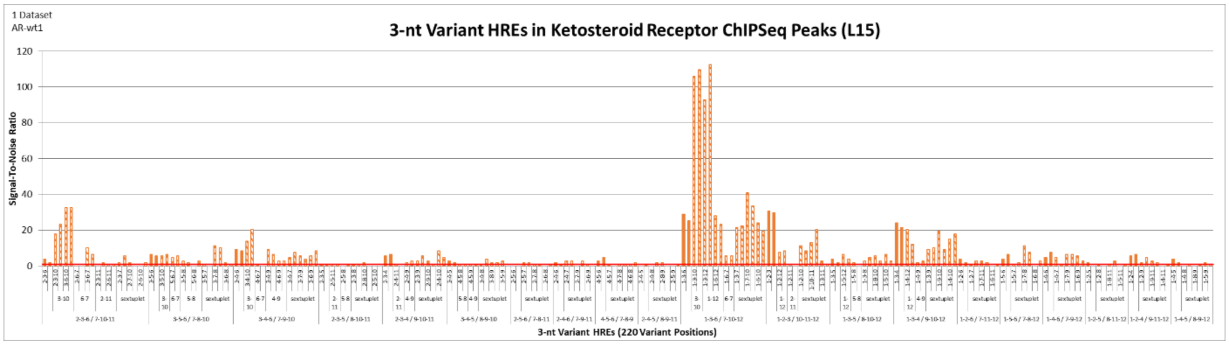

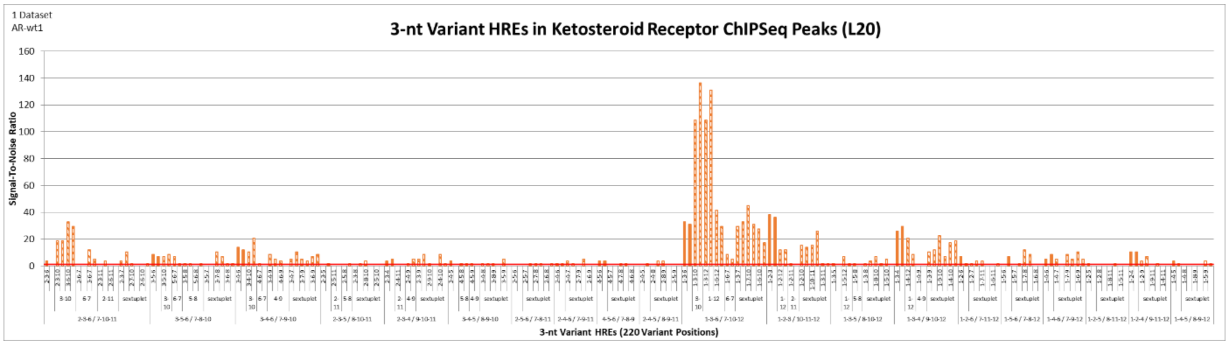

Figure S53. (S/N) analysis of 4-nt Variant HREs in KR ChIPSeq Peaks (Variant Position)

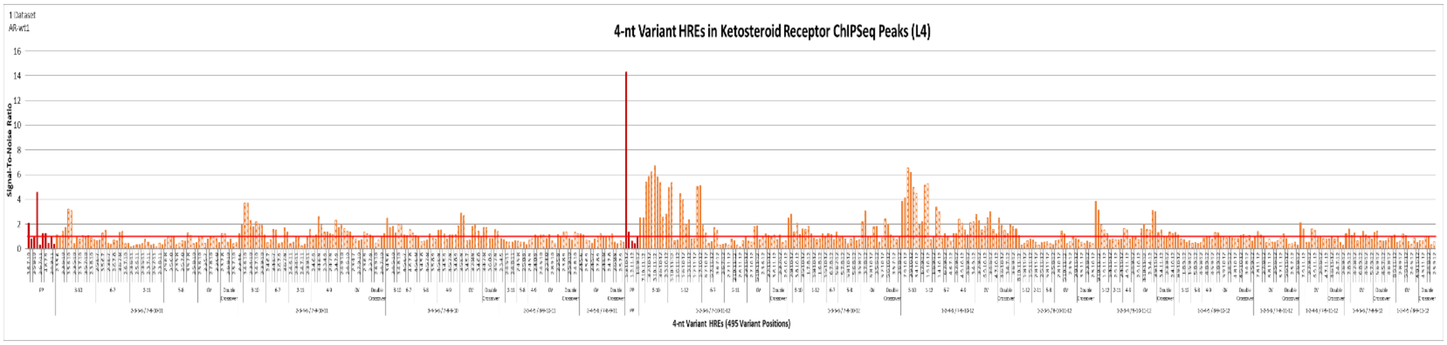

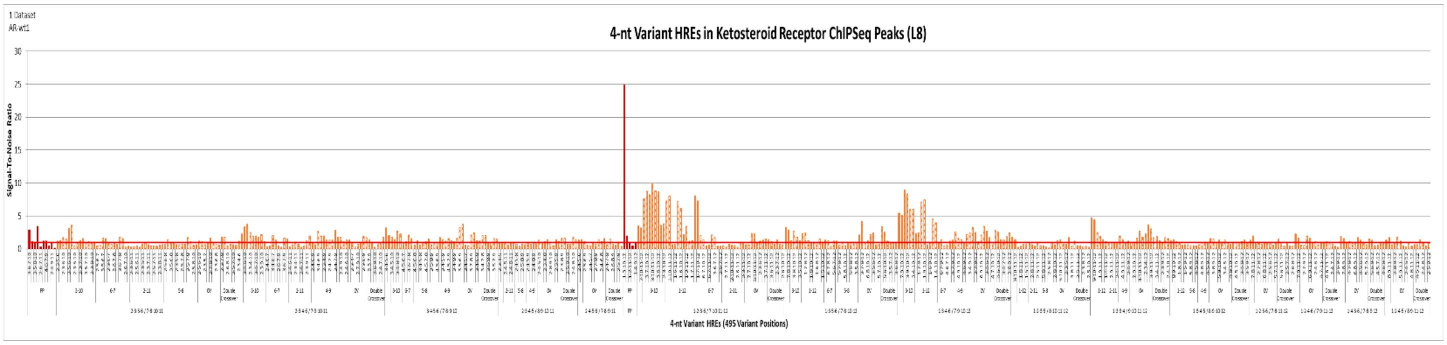

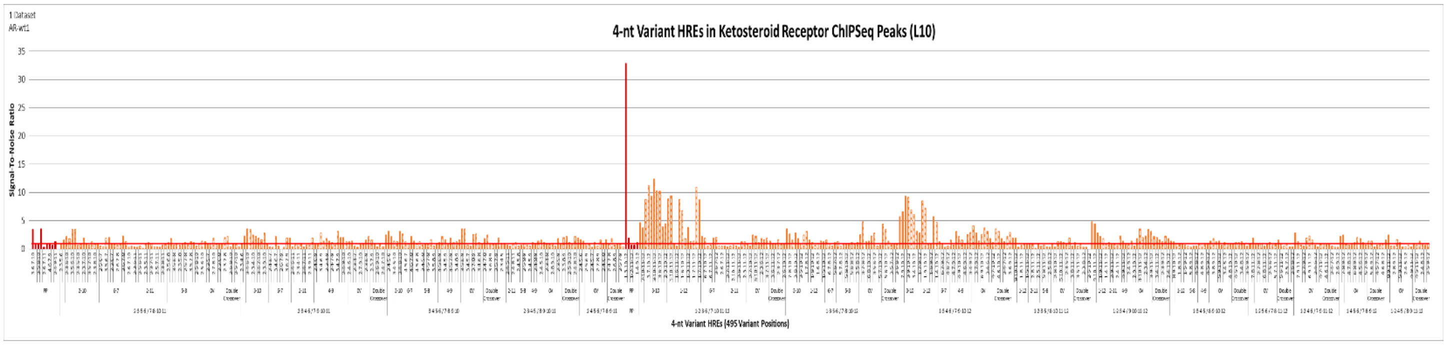

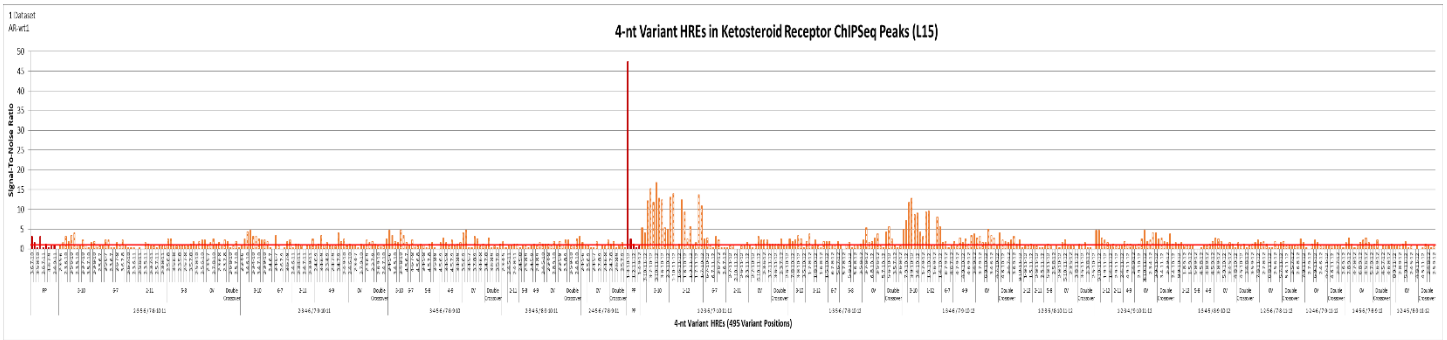

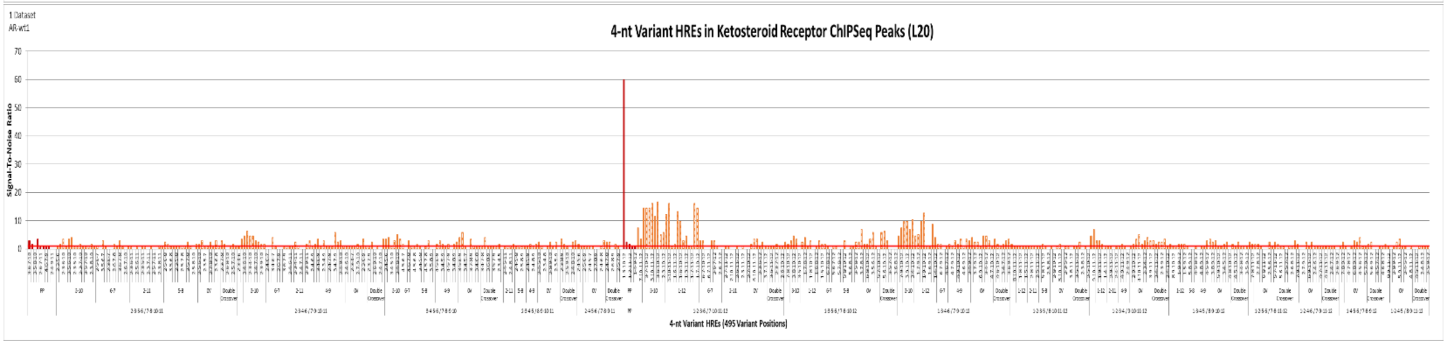

Figure S54. (S/N) analysis of 5-nt Variant HREs in KR ChIPSeq Peaks (Variant Position)

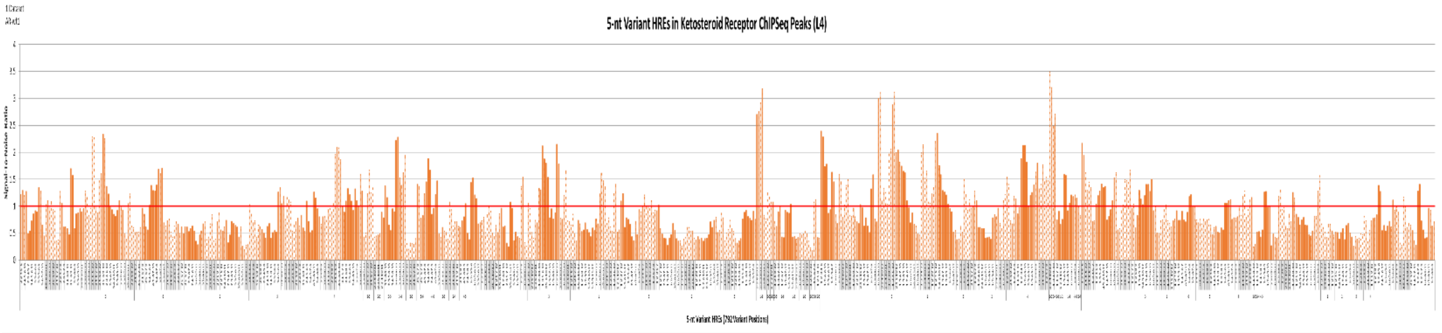

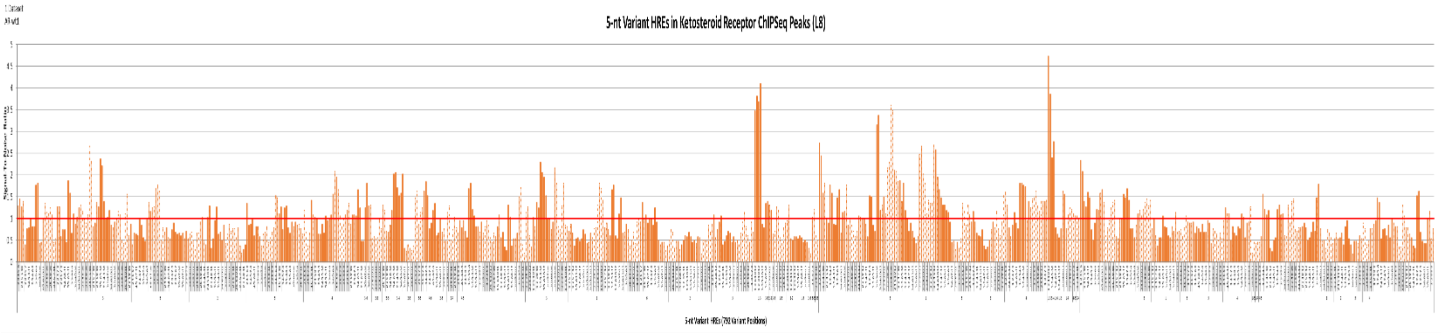

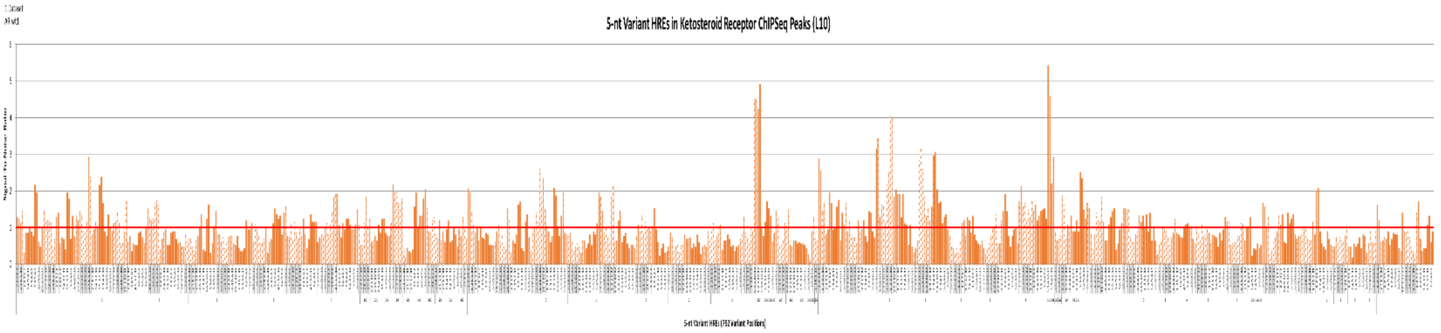

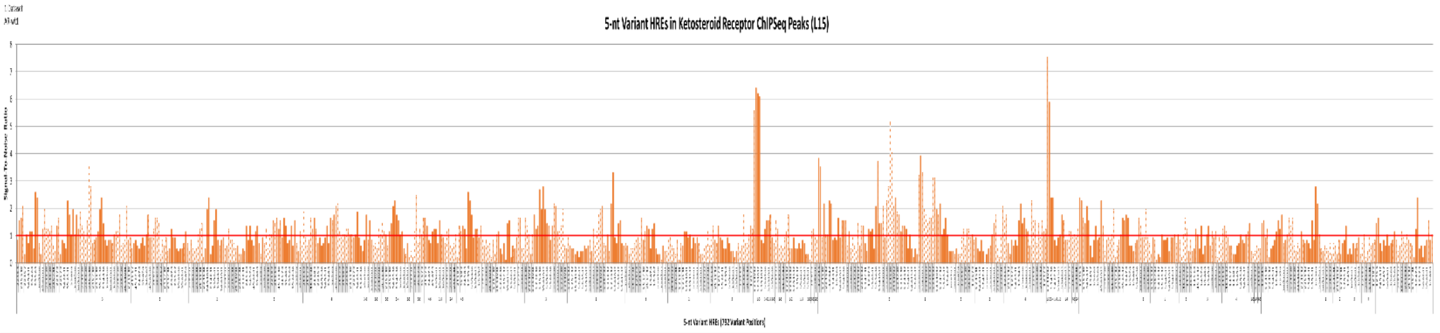

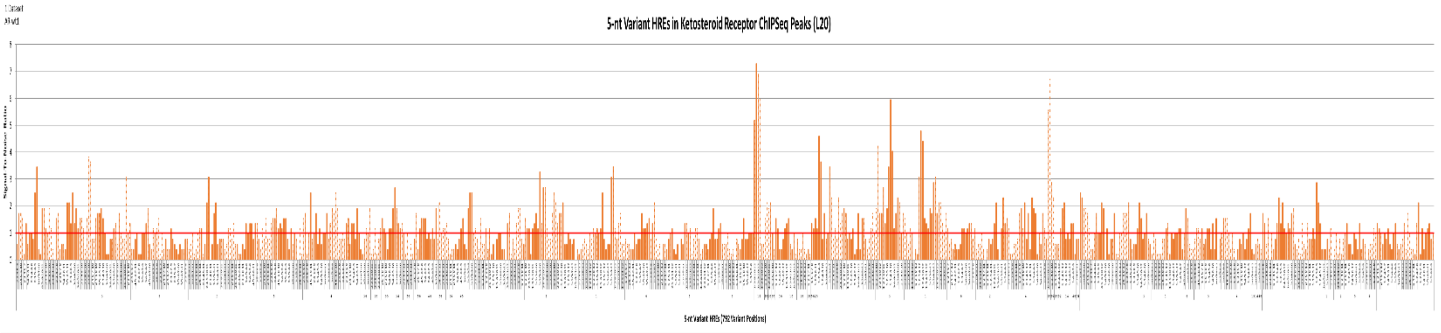

Figure S55. (S/N) analysis of 6-nt Variant HREs in KR ChIPSeq Peaks (Variant Position)

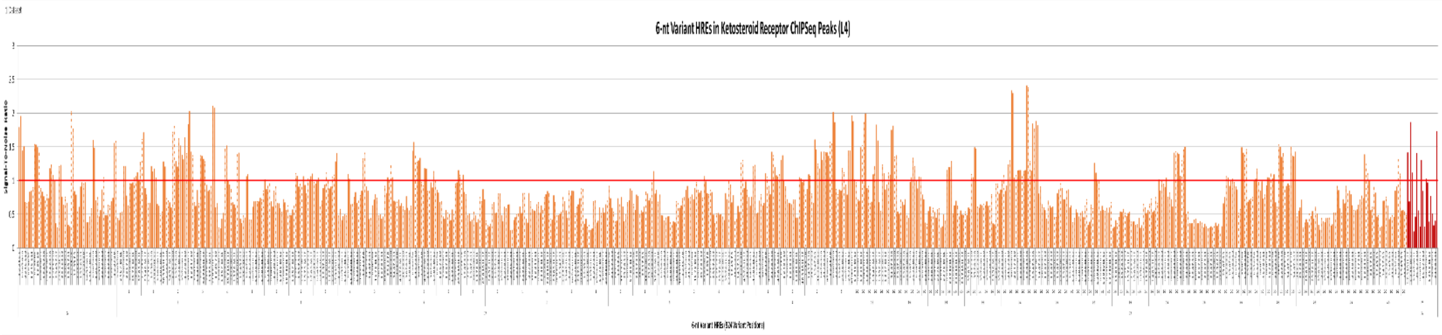

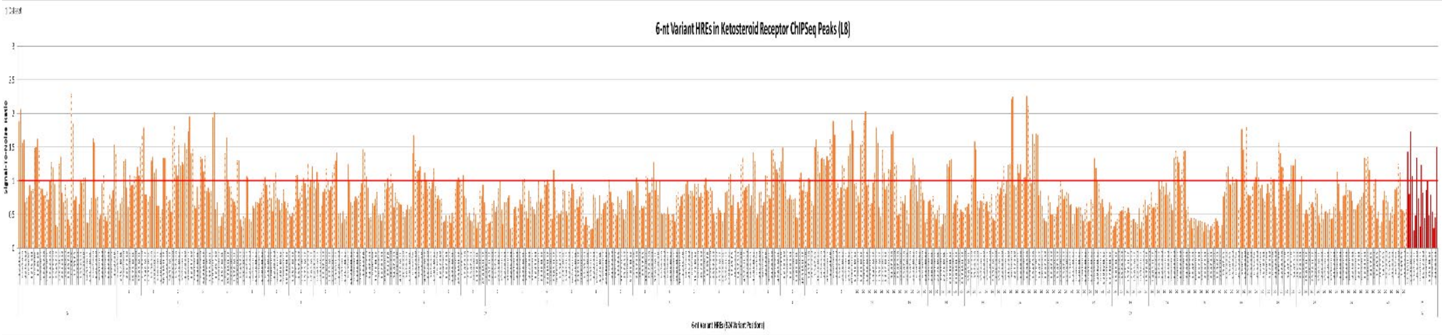

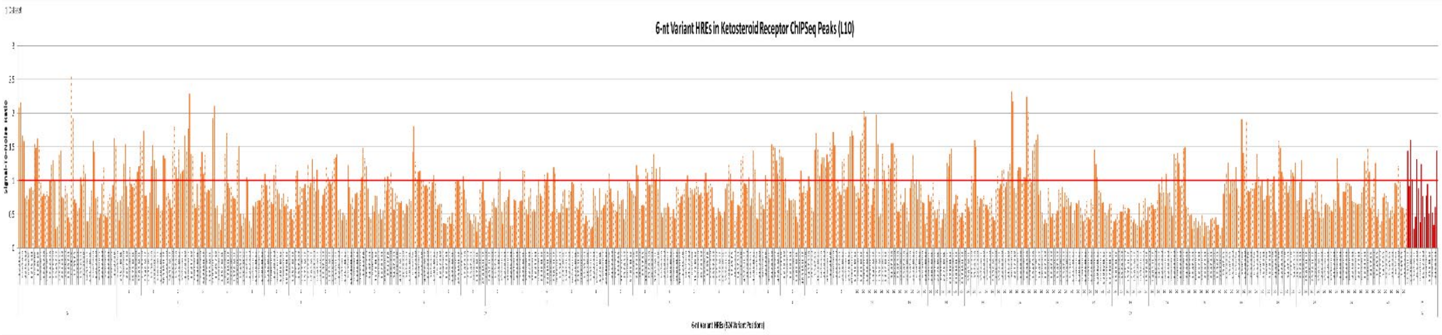

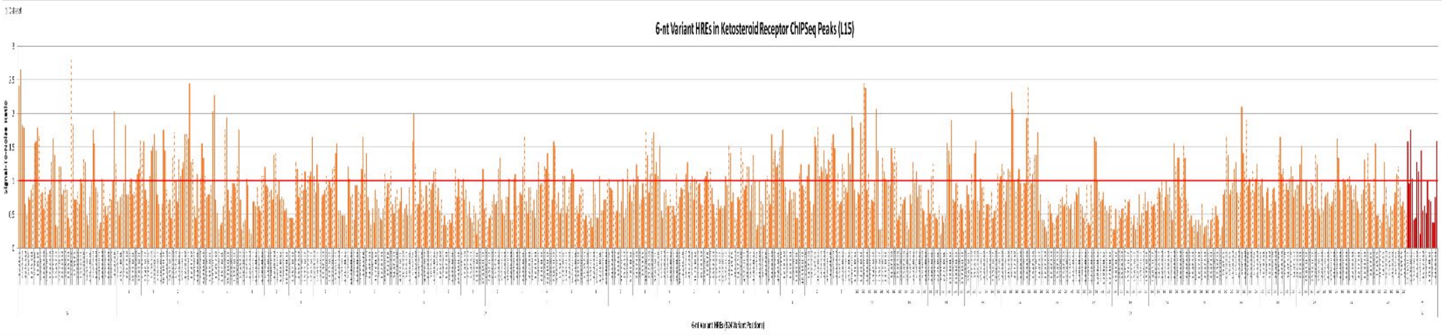

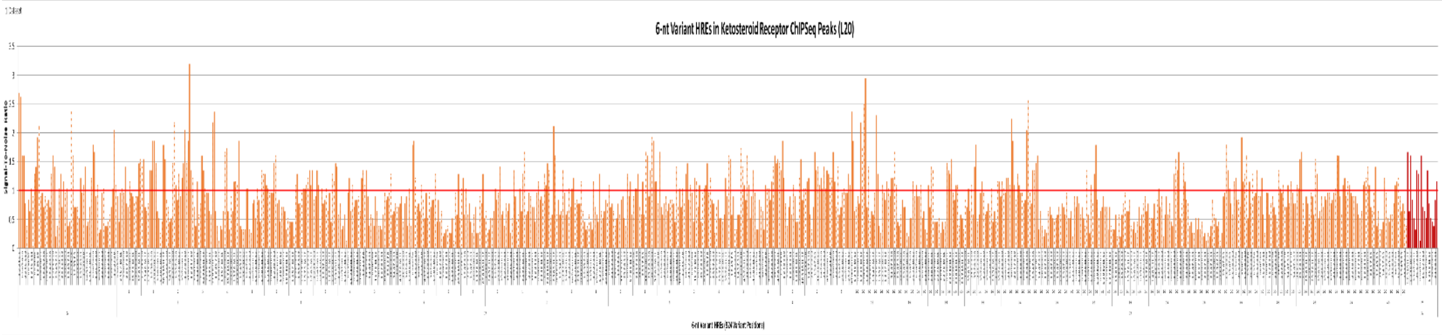

Figure S56. (S/N) analysis of 0-nt to 6-nt Variant HREs in GR and GR-Dim ChIPSeq Peaks #1 (924 Half-Site Groups)

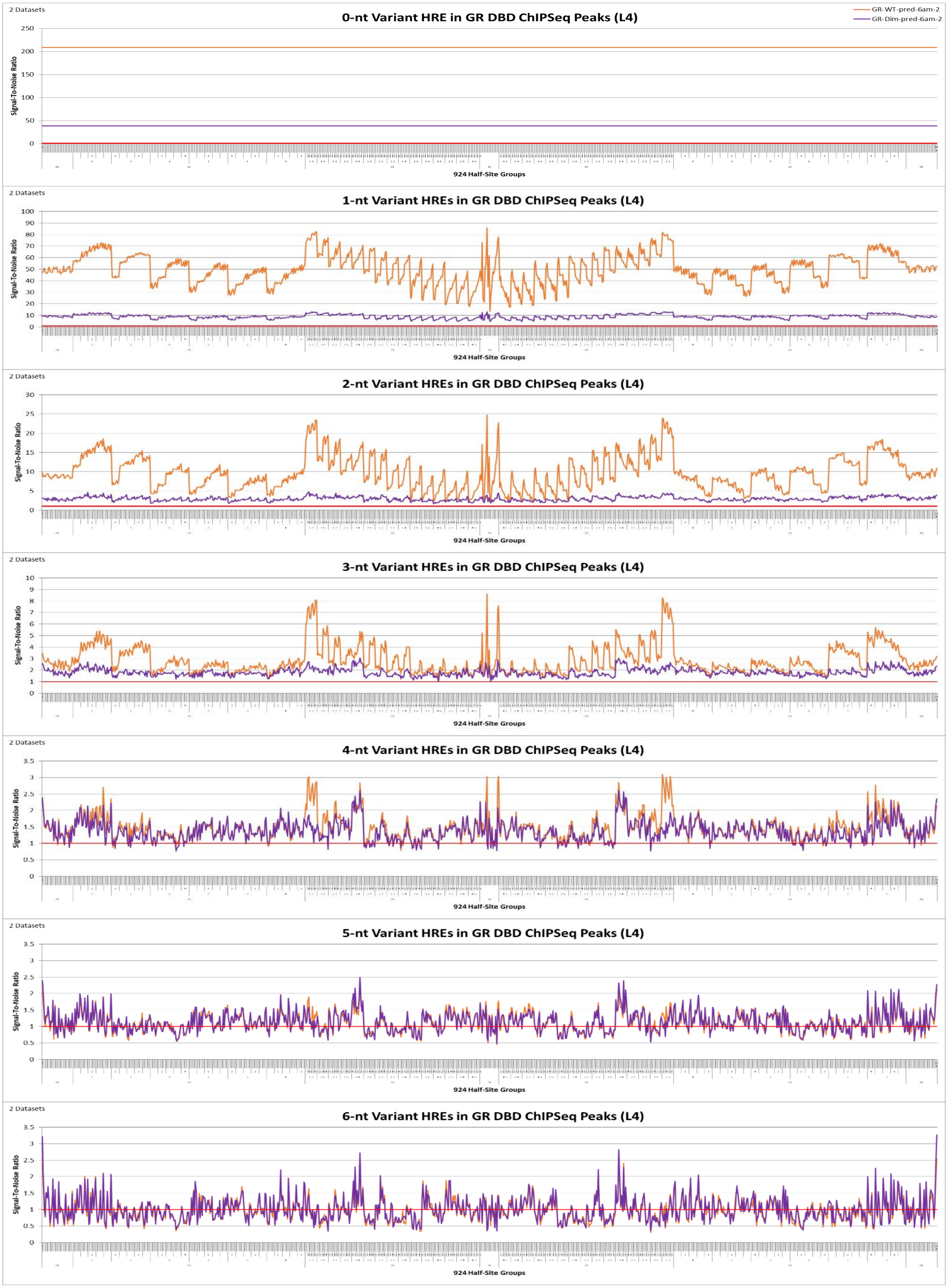

Figure S57. (S/N) analysis of 0-nt to 6-nt Variant HREs in GR and GR-Dim ChIPSeq Peaks #2 (924 Half-Site Groups)

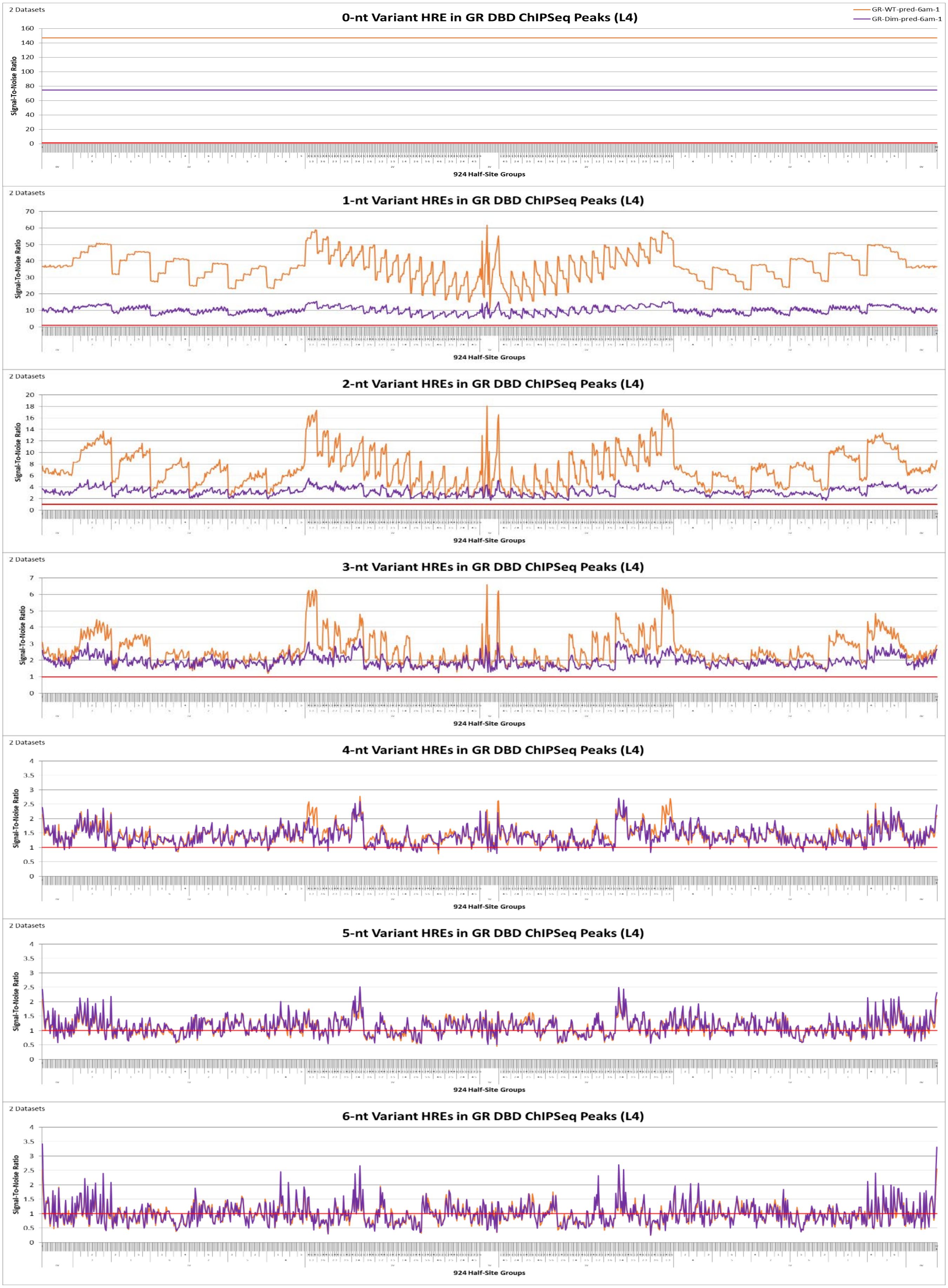

Figure S58. (S/N) analysis of 0-nt to 6-nt Variant HREs in GR and GR-Dim ChIPExo Peaks #1 (924 Half-Site Groups)

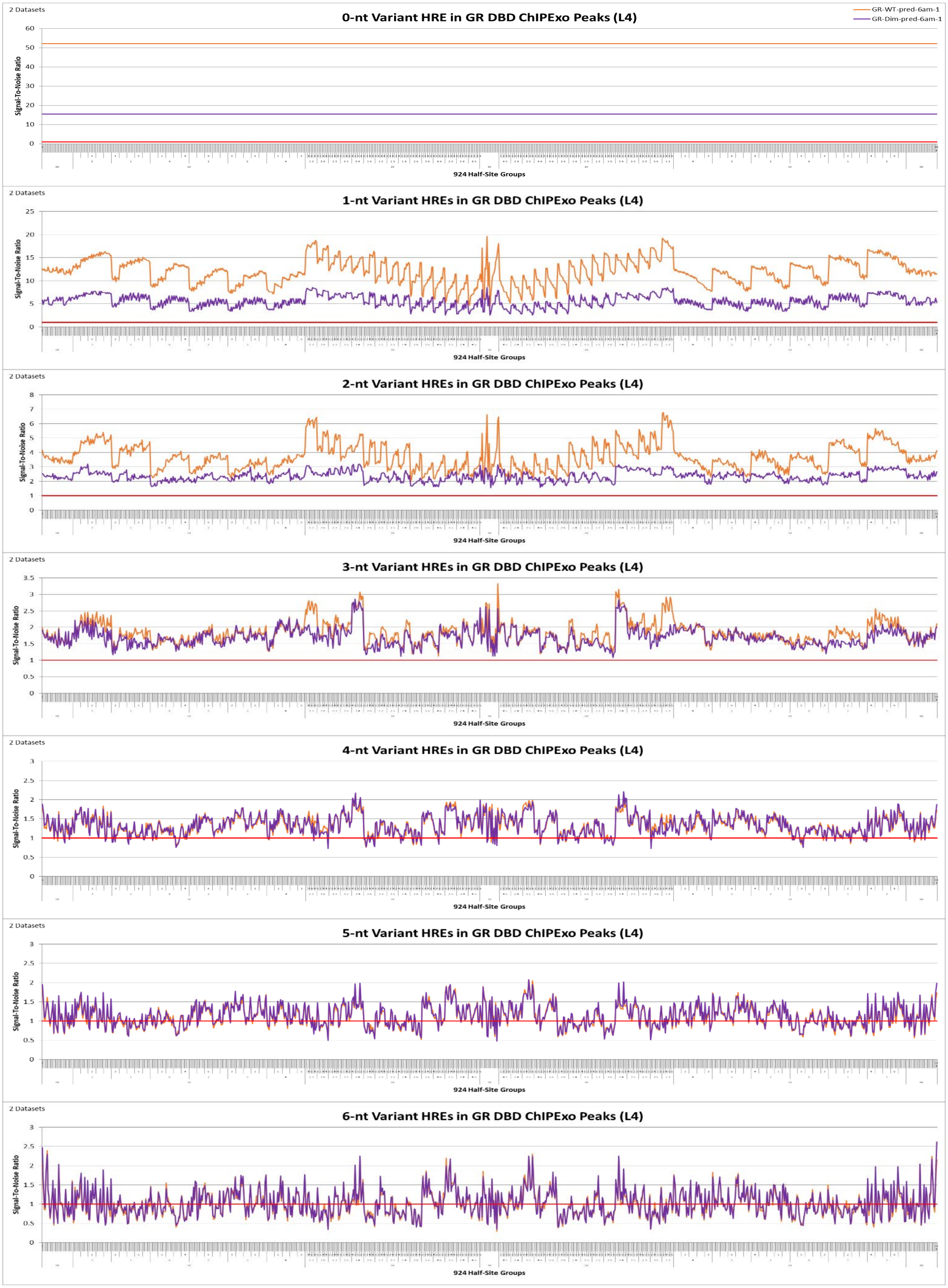

Figure S59. (S/N) analysis of 0-nt to 6-nt Variant HREs in GR and GR-Dim ChIPExo Peaks #2 (924 Half-Site Groups)

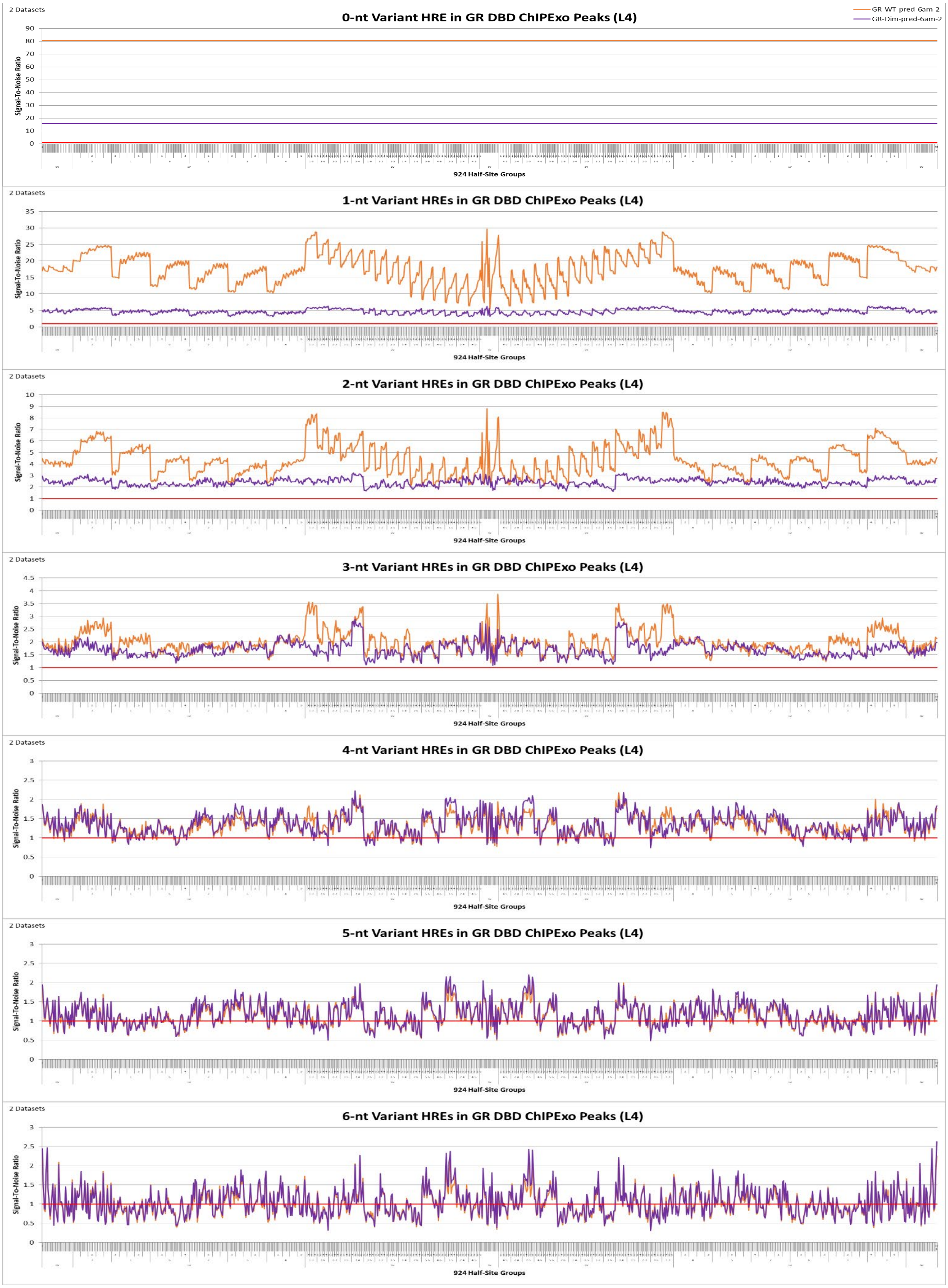

Figure S60. (S/N) analysis of 1-nt to 6-nt Variant HREs in GR and GR-Dim ChIPSeq Peaks (Variant Position)

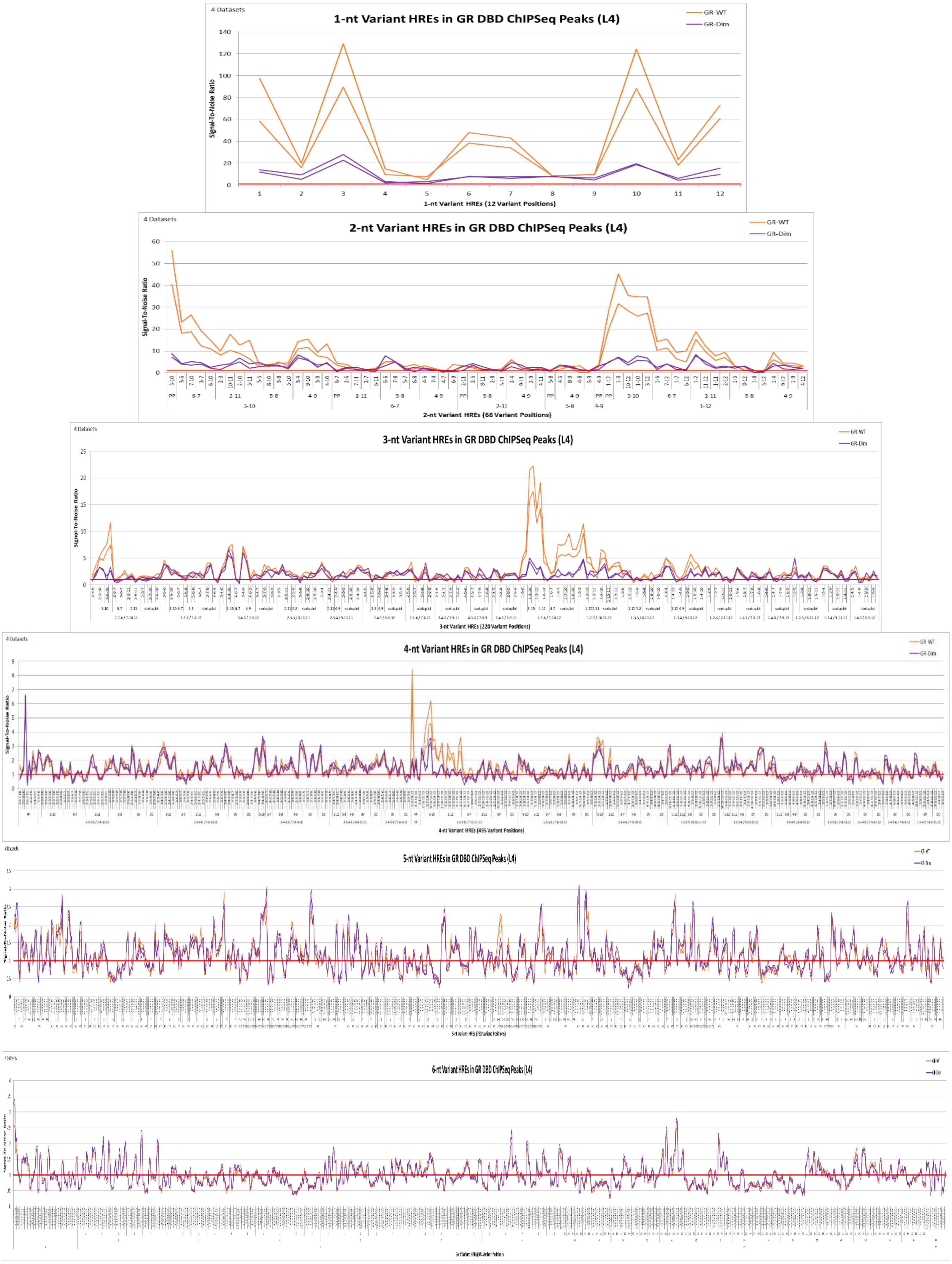

Figure S61. (S/N) analysis of 1-nt to 6-nt Variant HREs in GR and GR-Dim ChIPExo Peaks (Variant Position)

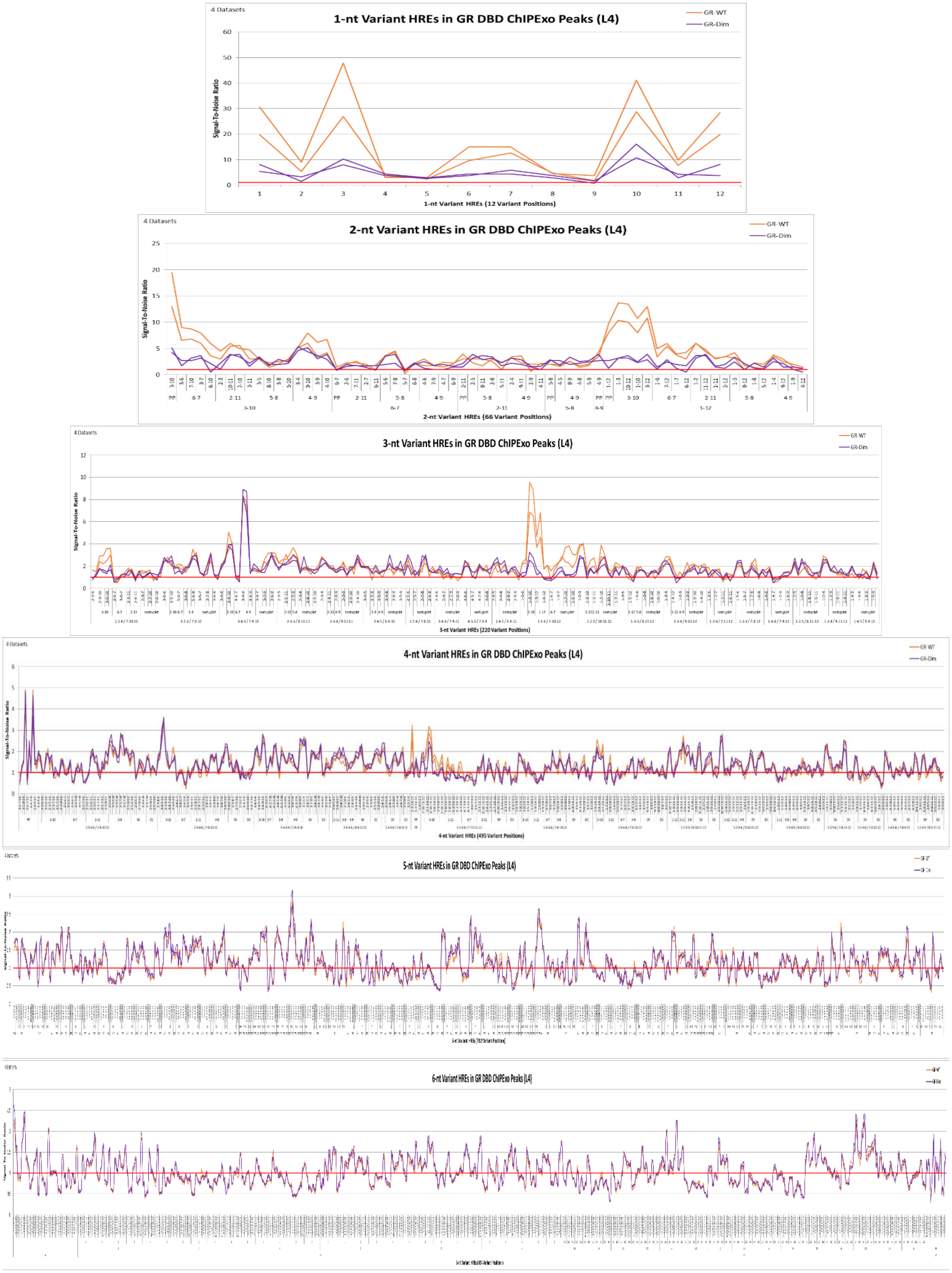

Figure S62-S71 Descriptions: Compare the 13-nt DNA Element Analysis to the 15-nt DNA Element Analysis

Comparative Figure Summary: 13-nt DNA Element Analysis vs. 15-nt DNA Element Analysis

We repeated the same sNR DNA-binding analysis (as was done for sNR DNA-binding at 0-nt to 5-nt variant 13-nt ERE and HRE DNA elements in the genome) for the newly identified 0-nt variant consensus palindromic 15-nt ERE and HRE DNA elements and extending through 6-nt variant DNA elements (i.e., a 6-nt variant =a half-site for a 15-nt DNA element with 12 primary positions) in the genome. Analysis of sNR DNA-binding at 13-nt ERE and HRE DNA elements included the 1 0-nt variant consensus palindromic DNA element, 30 1-nt variant DNA elements (10 variant positions), 405 2-nt variant DNA elements (45 variant positions), 3,240 3-nt variant DNA elements (120 variant positions), 17,010 4-nt variant DNA elements (210 variant positions), and 61,236 5-nt variant DNA elements (252 variant positions), for a total of 81,922 DNA elements. Analysis of sNR DNA-binding at 15-nt ERE and HRE DNA elements include the 1 0-nt variant consensus palindromic DNA element, 36 1-nt variant DNA elements (12 variant positions), 594 2-nt variant DNA elements (66 variant positions), 5,940 3-nt variant DNA elements (220 variant positions), 40,095 4-nt variant DNA elements (495 variant positions), 192,456 5-nt variant DNA elements (792 variant positions), and 673,596 6-nt variant DNA elements (924 variant positions), for a total of 912,718 DNA elements. All data analyses completed for sNR DNA-binding at 0-nt to 5-nt variant 13-nt ERE and HRE DNA elements in the genome was repeated for sNR DNA-binding at 0-nt to 6-nt variant 15-nt ERE and HRE DNA elements in the genome.

Comparative Figure			
13-nt DNA Element Analysis vs. 15-nt DNA Element Analysis			
	Figure Title	13-nt DNA Element	15-nt DNA Element
1	Number of ERE and HRE DNA Elements in the Mouse and Human Genome	**Table 1**	Figure S34
2	Inversion Symmetry of sNR DNA-Binding at 1-nt Variant DNA Elements in the Genome (%)	**Figure 1**	Figure S35
3	Inversion Symmetry of sNR DNA-Binding at 2-nt Variant DNA Elements in the Genome (%)	Figure S1	Figure S36
4	(S/N) analysis of 0-nt to 5-nt/6-nt Variant EREs in ER ChIPSeq Peaks (# of Variants)	Figure S2	Figure S37
5	(S/N) analysis of 0-nt to 5-nt/6-nt Variant HREs in KR ChIPSeq Peaks (# of Variants)	Figure S3	Figure S38
6	Half-Site Groups [252] [924]	**Table 2**	Figure S39
7	(S/N) analysis of 0-nt to 5-nt/6-nt Variant EREs in ER ChIPSeq Peaks (Half-Site Groups)	**Figure 2**	Figure S40
8	(S/N) analysis of 0-nt to 5-nt/6-nt Variant HREs in AR ChIPSeq Peaks (Half-Site Groups)	**Figure 3**	Figure S41
9	(S/N) analysis of 0-nt to 5-nt/6-nt Variant HREs in GR ChIPSeq Peaks (Half-Site Groups)	Figure S4	Figure S42
10	(S/N) analysis of 0-nt to 5-nt/6-nt Variant HREs in PR ChIPSeq Peaks (Half-Site Groups)	Figure S5	Figure S43
11	(S/N) analysis of 1-nt Variant EREs in ER ChIPSeq Peaks (Variant Position) [10] [12]	Figure S12	Figure S44
12	(S/N) analysis of 2-nt Variant EREs in ER ChIPSeq Peaks (Variant Position) [45] [66]	Figure S13	Figure S45
13	(S/N) analysis of 3-nt Variant EREs in ER ChIPSeq Peaks (Variant Position) [120] [220]	Figure S14	Figure S46
14	(S/N) analysis of 4-nt Variant EREs in ER ChIPSeq Peaks (Variant Position) [210] [495]	Figure S15	Figure S47
15	(S/N) analysis of 5-nt Variant EREs in ER ChIPSeq Peaks (Variant Position) [252] [792]	Figure S16	Figure S48
16	(S/N) analysis of 1-nt Variant HREs in KR ChIPSeq Peaks (Variant Position) [10] [12]	Figure S17	Figure S50
17	(S/N) analysis of 2-nt Variant HREs in KR ChIPSeq Peaks (Variant Position) [45] [66]	Figure S18	Figure S51
18	(S/N) analysis of 3-nt Variant HREs in KR ChIPSeq Peaks (Variant Position) [120] [220]	Figure S19	Figure S52
19	(S/N) analysis of 4-nt Variant HREs in KR ChIPSeq Peaks (Variant Position) [210] [495]	Figure S20	Figure S53
20	(S/N) analysis of 5-nt Variant HREs in KR ChIPSeq Peaks (Variant Position) [252] [792]	Figure S21	Figure S54
21	(S/N) analysis of 0-nt to 5-nt/6-nt Variant HREs in GR and GR-Dim ChIPSeq Peaks #1 (Half-Site Groups)	**Figure 4**	Figure S56
22	(S/N) analysis of 0-nt to 5-nt/6-nt Variant HREs in GR and GR-Dim ChIPSeq Peaks #2 (Half-Site Groups)	Figure S22	Figure S57
23	(S/N) analysis of 0-nt to 5-nt/6-nt Variant HREs in GR and GR-Dim ChIPExo Peaks #1 (Half-Site Groups)	Figure S23	Figure S58
24	(S/N) analysis of 0-nt to 5-nt/6-nt Variant HREs in GR and GR-Dim ChIPExo Peaks #2 (Half-Site Groups)	Figure S24	Figure S59
25	(S/N) analysis of 0-nt to 5-nt/6-nt Variant HREs in GR and GR-Dim ChIPSeq Peaks (Variant Position)	**Figure 5**	Figure S60
26	(S/N) analysis of 0-nt to 5-nt/6-nt Variant HREs in GR and GR-Dim ChIPExo Peaks (Variant Position)	Figure S25	Figure S61

*****

****All detailed data and statistics associated with every figure are compiled in Data S1****

To allow for a more direct comparison between the sNR DNA-binding profile at the 81,922 0-nt to 5-nt variant 13-nt ERE or HRE DNA elements in the genome versus at the 912,718 0-nt to 6-nt variant 15-nt ERE or HRE DNA elements in the genome, in this section we have removed the bars associated with sNR DNA-binding at DNA elements that have a variant in position 1 or position 12 from the plots of the 15-nt ERE and HRE DNA element analysis. For example, looking at **Figure S62**, the plots on the left-hand side of the page show ER DNA-binding at the 30 1-nt variant 13-nt ERE DNA elements (10 variant positions) in the genome (5′-GGTCAnnnTGACC-3′), while the plots on the right-hand side of the page show ER DNA-binding at the 36 1-nt variant 15-nt ERE DNA elements (12 variant positions) in the genome (5′-AGGTCAnnnTGACCT-3′). We have removed the bars that represent ER DNA-binding at position 1 and position 12 from the plots on the right-hand side of the page (i.e., the 15-nt ERE DNA element analysis), as ER DNA-binding at those sites is expanded information from the 13-nt ERE DNA element analysis. Thus, the 15-nt ERE and HRE DNA element analysis plots (i.e., on the right-hand side of the page) are the same plots from **Figure S44-S55**, except we have removed this expanded information (i.e., bars that represent sNR DNA-binding at DNA elements that have a variant in position 1 or position 12 of the 15-nt ERE and HRE DNA elements). The DNA-binding profile at the 13-nt DNA elements in the genome versus at the 15-nt DNA elements in the genome are almost equivalent, confirming that the DNA-binding rules established by the 13-nt DNA element analysis also apply to the 15-nt DNA element analysis. **Note**: see section *"Transform the 13-nt DNA Element Analysis to the 15-nt DNA Element Analysis"* to understand the minor differences in the relative amplitudes of the sNR DNA-binding profile at the 13-nt DNA elements in the genome versus at the 15-nt DNA elements in the genome.

Figure S62. Compare (S/N) analysis of 1-nt Variant EREs in ER ChIPSeq Peaks (13-nt vs. 15-nt)
(S/N) analysis of 1-nt variant EREs in ER (WT-E2-1hr) ChIPSeq peaks [76,163 peaks, 146-nt peak length].
Displayed by the 10 variant positions of the 30 1-nt variant EREs [13-nt ERE] versus the 10 of 12 variant positions of the 36 1-nt variant EREs [15-nt ERE].

Figure S63. Compare (S/N) analysis of 2-nt Variant EREs in ER ChIPSeq Peaks (13-nt vs. 15-nt)
(S/N) analysis of 2-nt variant EREs in ER (WT-E2-1hr) ChIPSeq peaks [76,163 peaks, 146-nt peak length].
Displayed by the 45 variant positions of the 405 2-nt variant EREs [13-nt ERE] versus the 45 of 66 variant positions of the 594 2-nt variant EREs [15-nt ERE]. 5 of the 45 variant positions are the five (5) palindromic position pairs: 1-10, 2-9, 3-8, 4-7, 5-6 (PP, dark red solid bars). 20 of the 45 variant positions are same-side variants (i.e., the variants do not crossover the 3-nt spacer) (blue solid bars). 20 of the 45 variant positions are crossover variants (i.e., the variants do crossover the 3-nt spacer) (blue diagonal stripped bars). Each variant position is immediately followed by its reverse-complement variant position on the x-axis, demonstrating that transcription factor binding in the genome follows inversion symmetry (i.e., the number of transcription factor binding events at a particular DNA element in the genome is equivalent to the number of transcription factor binding events at its reverse-complement DNA element in the genome).

Figure S64. Compare (S/N) analysis of 3-nt Variant EREs in ER ChIPSeq Peaks (13-nt vs. 15-nt)
(S/N) analysis of 3-nt variant EREs in ER (WT-E2-1hr) ChIPSeq peaks [76,163 peaks, 146-nt peak length].
Displayed by the 120 variant positions of the 3,240 3-nt variant EREs [13-nt ERE] versus the 120 of 220 variant positions of the 5,940 3-nt variant EREs [15-nt ERE]. 20 of the 120 variant positions are same-side variants (i.e., the variants do not crossover the 3-nt spacer) (blue solid bars). 100 of the 120 variant positions are crossover variants (i.e., the variants do crossover the 3-nt spacer) (blue diagonal stripped bars). Each variant position is immediately followed by its reverse-complement variant position on the x-axis, demonstrating that transcription factor binding in the genome follows inversion symmetry (i.e., the number of transcription factor binding events at a particular DNA element in the genome is equivalent to the number of transcription factor binding events at its reverse-complement DNA element in the genome).

Figure S65. Compare (S/N) analysis of 4-nt Variant EREs in ER ChIPSeq Peaks (13-nt vs. 15-nt)
(S/N) analysis of 4-nt variant EREs in ER (WT-E2-1hr) ChIPSeq peaks [76,163 peaks, 146-nt peak length].
Displayed by the 210 variant positions of the 17,010 4-nt variant EREs [13-nt ERE] versus the 210 of 495 variant positions of the 40,095 4-nt variant EREs [15-nt ERE]. 10 of the 210 variant positions are the five (5) palindromic position pairs: 1-3-8-10, 3-5-6-8, 3-4-7-8, 2-3-8-9, 1-5-6-10, 1-4-7-10, 1-2-9-10, 4-5-6-7, 2-5-6-9, 2-4-7-9 (PP, dark red solid bars). 10 of the 210 variant positions are same-side variants (i.e., the variants do not crossover the 3-nt spacer) (blue solid bars). 190 of the 45 variant positions are crossover variants (i.e., the variants do crossover the 3-nt spacer) (blue diagonal stripped bars). Each variant position is immediately followed by its reverse-complement variant position on the x-axis, demonstrating that transcription factor binding in the genome follows inversion symmetry (i.e., the number of transcription factor binding events at a particular DNA element in the genome is equivalent to the number of transcription factor binding events at its reverse-complement DNA element in the genome).

Figure S66. Compare (S/N) analysis of 5-nt Variant EREs in ER ChIPSeq Peaks (13-nt vs. 15-nt)
(S/N) analysis of 5-nt variant EREs in ER (WT-E2-1hr) ChIPSeq peaks [76,163 peaks, 146-nt peak length].
Displayed by the 252 variant positions of the 61,236 5-nt variant EREs [13-nt ERE] versus the 252 of 792 variant positions of the 192,456 5-nt variant EREs [15-nt ERE]. 2 of the 252 variant positions are same-side variants (i.e., the variants do not crossover the 3-nt spacer) (blue solid bars). 250 of the 252 variant positions are crossover variants (i.e., the variants do crossover the 3-nt spacer) (blue diagonal stripped bars). Each variant position is immediately followed by its reverse-complement variant position on the x-axis, demonstrating that transcription factor binding in the genome follows inversion symmetry (i.e., the number of transcription factor binding events at a particular DNA element in the genome is equivalent to the number of transcription factor binding events at its reverse-complement DNA element in the genome).

*****

Figure S67. Compare (S/N) analysis of 1-nt Variant HREs in KR ChIPSeq Peaks (13-nt vs. 15-nt)
(S/N) analysis of 1-nt variant HREs in AR (AR-wt1) ChIPSeq peaks [49,859 peaks, 136-nt peak length].
Displayed by the 10 variant positions of the 30 1-nt variant HREs [13-nt HRE] versus the 10 of 12 variant positions of the 36 1-nt variant HREs [15-nt HRE].

Figure S68. Compare (S/N) analysis of 2-nt Variant HREs in KR ChIPSeq Peaks (13-nt vs. 15-nt)
(S/N) analysis of 2-nt variant HREs in AR (AR-wt1) ChIPSeq peaks [49,859 peaks, 136-nt peak length].
Displayed by the 45 variant positions of the 405 2-nt variant HREs [13-nt HRE] versus the 45 of 66 variant positions of the 594 2-nt variant HREs [15-nt HRE]. 5 of the 45 variant positions are the five (5) palindromic position pairs: 1-10, 2-9, 3-8, 4-7, 5-6 (PP, dark red solid bars). 20 of the 45 variant positions are same-side variants (i.e., the variants do not crossover the 3-nt spacer) (orange solid bars). 20 of the 45 variant positions are crossover variants (i.e., the variants do crossover the 3-nt spacer) (orange diagonal stripped bars). Each variant position is immediately followed by its reverse-complement variant position on the x-axis, demonstrating that transcription factor binding in the genome follows inversion symmetry (i.e., the number of transcription factor binding events at a

particular DNA element in the genome is equivalent to the number of transcription factor binding events at its reverse-complement DNA element in the genome).

Figure S69. Compare (S/N) analysis of 3-nt Variant HREs in KR ChIPSeq Peaks (13-nt vs. 15-nt)
(S/N) analysis of 3-nt variant HREs in AR (AR-wt1) ChIPSeq peaks [49,859 peaks, 136-nt peak length].
Displayed by the 120 variant positions of the 3,240 3-nt variant HREs [13-nt HRE] versus the 120 of 220 variant positions of the 5,940 3-nt variant HREs [15-nt HRE]. 20 of the 120 variant positions are same-side variants (i.e., the variants do not crossover the 3-nt spacer) (orange solid bars). 100 of the 120 variant positions are crossover variants (i.e., the variants do crossover the 3-nt spacer) (orange diagonal stripped bars). Each variant position is immediately followed by its reverse-complement variant position on the x-axis, demonstrating that transcription factor binding in the genome follows inversion symmetry (i.e., the number of transcription factor binding events at a particular DNA element in the genome is equivalent to the number of transcription factor binding events at its reverse-complement DNA element in the genome).

Figure S70. Compare (S/N) analysis of 4-nt Variant HREs in KR ChIPSeq Peaks (13-nt vs. 15-nt)
(S/N) analysis of 4-nt variant HREs in AR (AR-wt1) ChIPSeq peaks [49,859 peaks, 136-nt peak length].
Displayed by the 210 variant positions of the 17,010 4-nt variant HREs [13-nt HRE] versus the 210 of 495 variant positions of the 40,095 4-nt variant HREs [15-nt HRE]. 10 of the 210 variant positions are the five (5) palindromic position pairs: 2-5-6-9, 1-2-9-10, 2-4-7-9, 2-3-8-9, 1-5-6-10, 4-5-6-7, 3-5-6-8, 1-4-7-10, 1-3-8-10, 3-4-7-8 (PP, dark red solid bars). 10 of the 210 variant positions are same-side variants (i.e., the variants do not crossover the 3-nt spacer) (orange solid bars). 190 of the 45 variant positions are crossover variants (i.e., the variants do crossover the 3-nt spacer) (orange diagonal stripped bars). Each variant position is immediately followed by its reverse-complement variant position on the x-axis, demonstrating that transcription factor binding in the genome follows inversion symmetry (i.e., the number of transcription factor binding events at a particular DNA element in the genome is equivalent to the number of transcription factor binding events at its reverse-complement DNA element in the genome).

Figure S71. Compare (S/N) analysis of 5-nt Variant HREs in KR ChIPSeq Peaks (13-nt vs. 15-nt)
(S/N) analysis of 5-nt variant HREs in AR (AR-wt1) ChIPSeq peaks [49,859 peaks, 136-nt peak length].
Displayed by the 252 variant positions of the 61,236 5-nt variant HREs [13-nt HRE] versus the 252 of 792 variant positions of the 192,456 5-nt variant HREs [15-nt HRE]. 2 of the 252 variant positions are same-side variants (i.e., the variants do not crossover the 3-nt spacer) (orange solid bars). 250 of the 252 variant positions are crossover variants (i.e., the variants do crossover the 3-nt spacer) (orange diagonal stripped bars). Each variant position is immediately followed by its reverse-complement variant position on the x-axis, demonstrating that transcription factor binding in the genome follows inversion symmetry (i.e., the number of transcription factor binding events at a particular DNA element in the genome is equivalent to the number of transcription factor binding events at its reverse-complement DNA element in the genome).

Figure S62. Compare (S/N) analysis of 1-nt Variant EREs in ER ChIPSeq Peaks (13-nt vs. 15-nt)

1-nt Variant 13-nt ERE DNA Elements | **1-nt Variant 15-nt ERE DNA Elements**

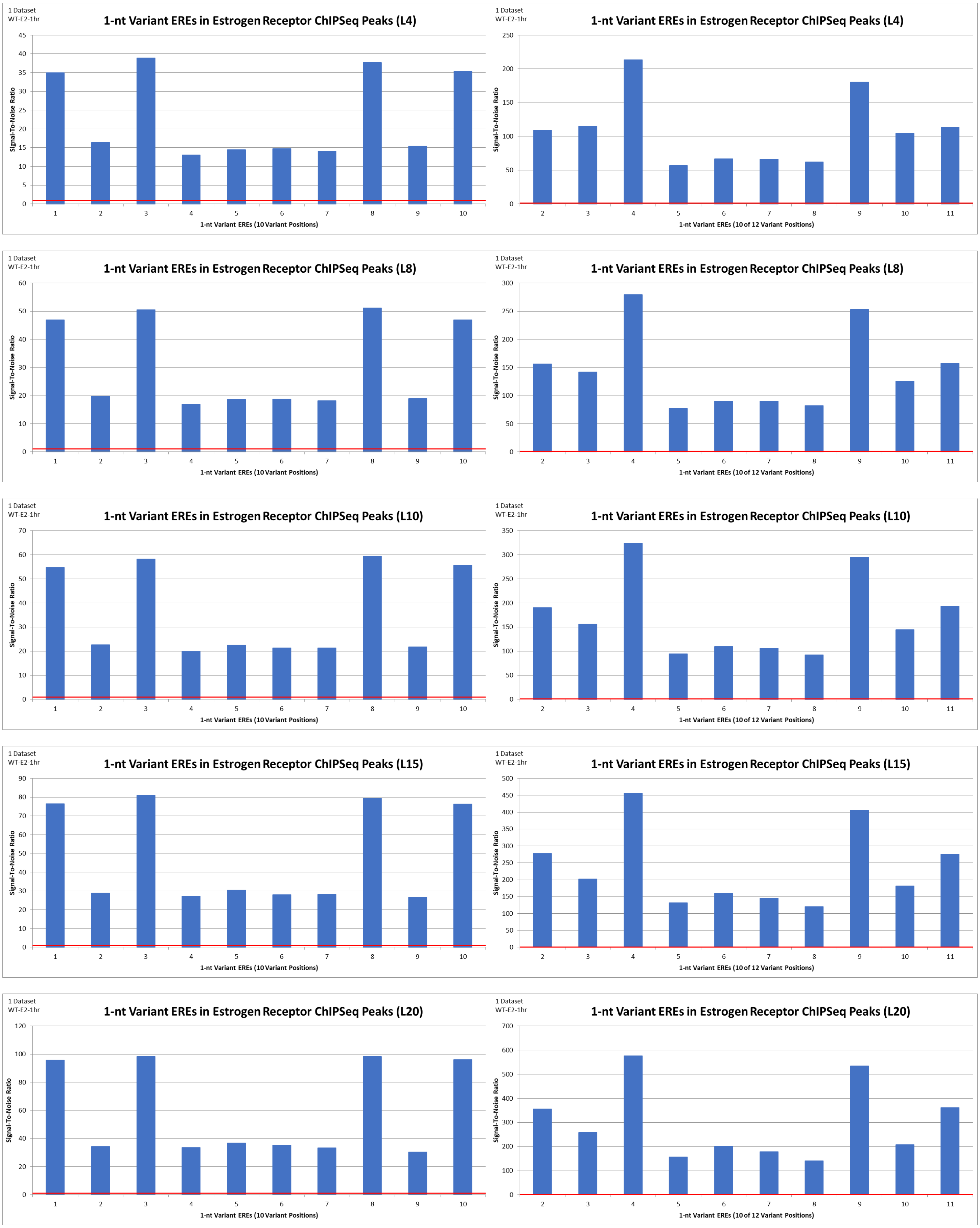

Figure S63. Compare (S/N) analysis of 2-nt Variant EREs in ER ChIPSeq Peaks (13-nt vs. 15-nt)

2-nt Variant 13-nt ERE DNA Elements | **2-nt Variant 15-nt ERE DNA Elements**

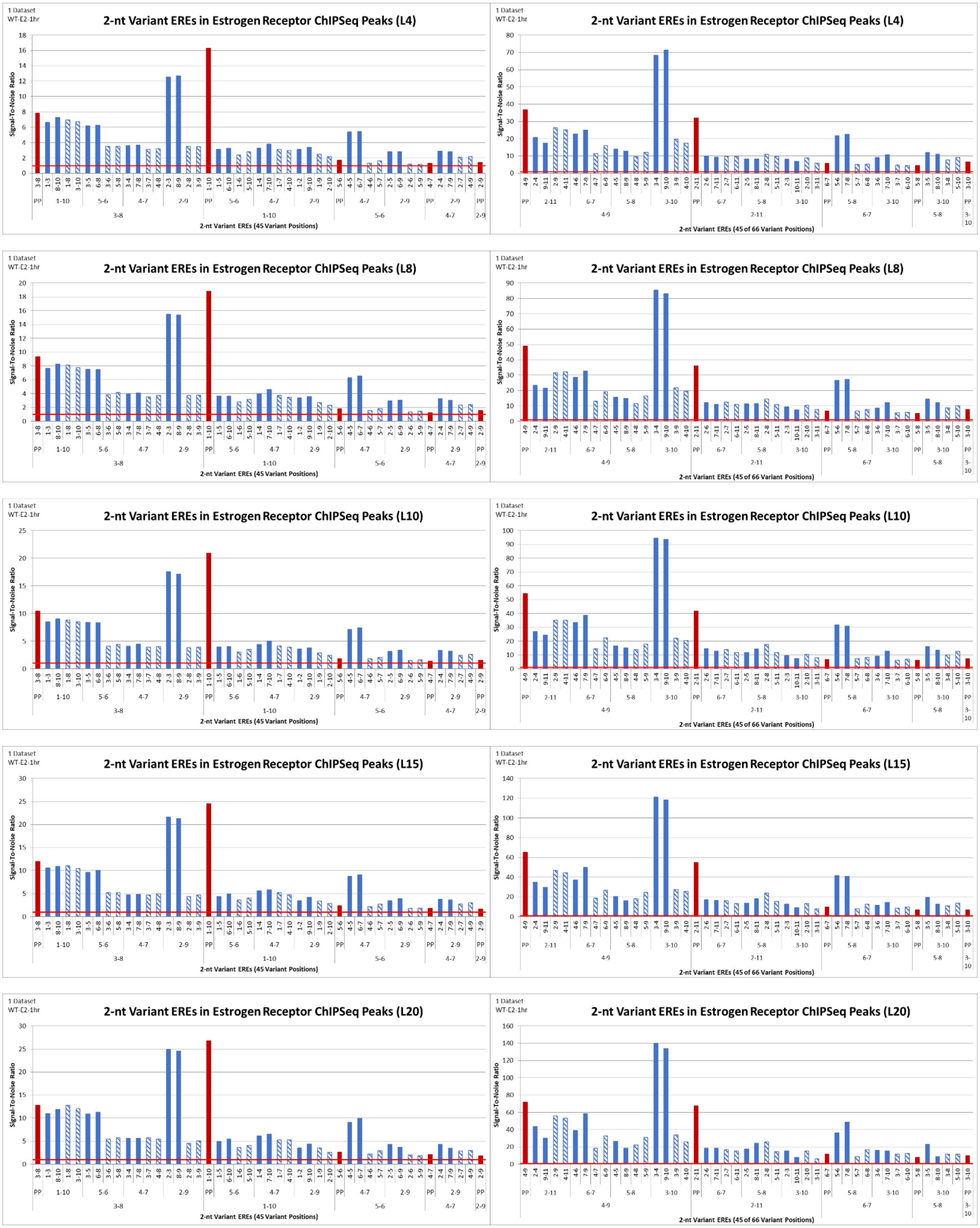

Figure S64. Compare (S/N) analysis of 3-nt Variant EREs in ER ChIPSeq Peaks (13-nt vs. 15-nt)

3-nt Variant 13-nt ERE DNA Elements

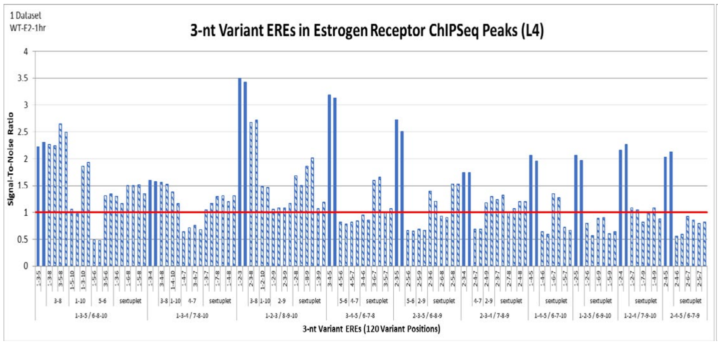

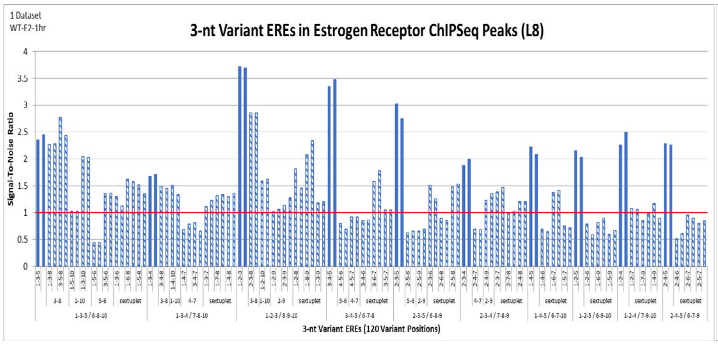

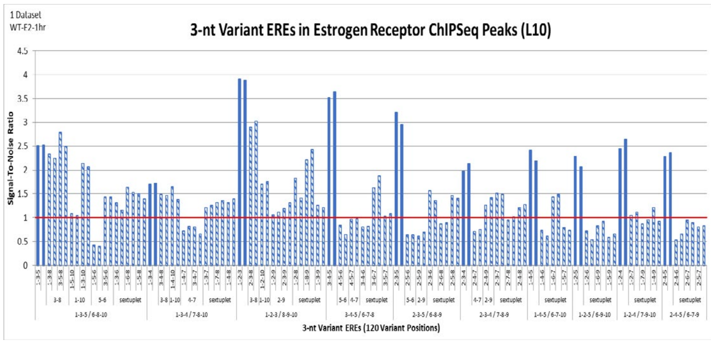

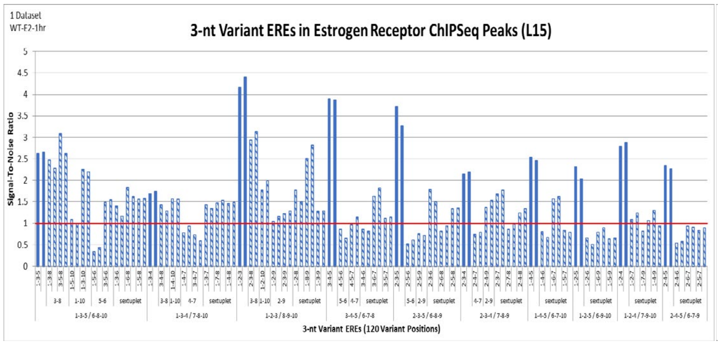

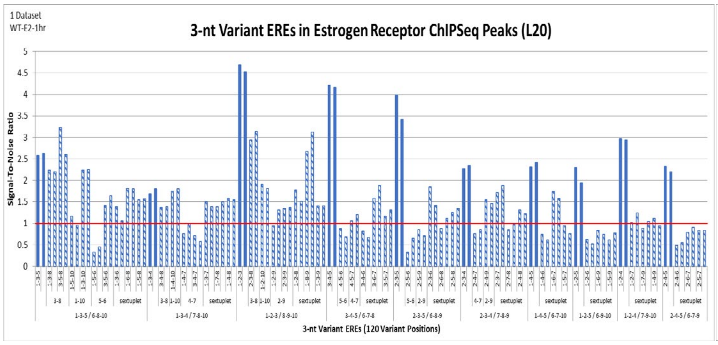

3-nt Variant 15-nt ERE DNA Elements

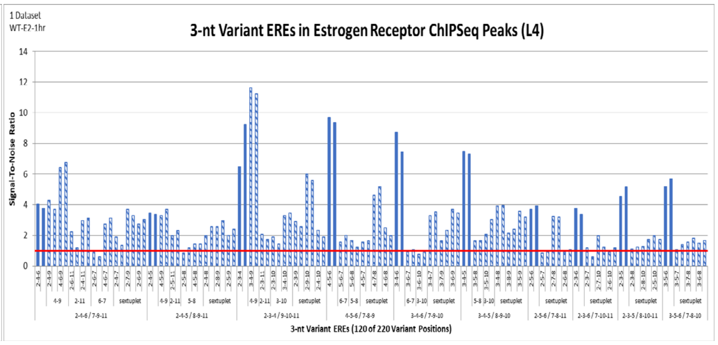

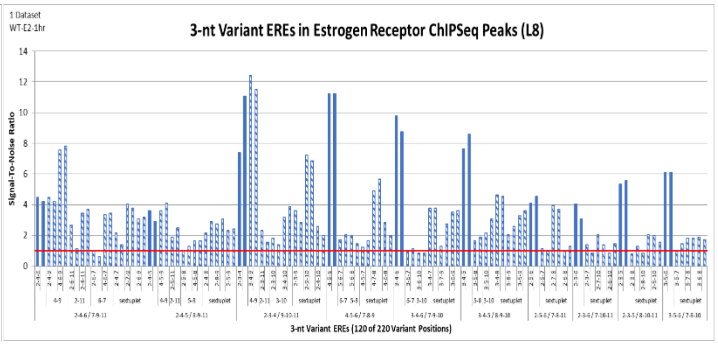

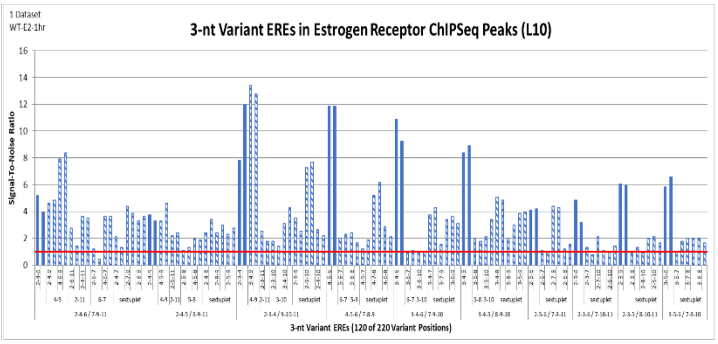

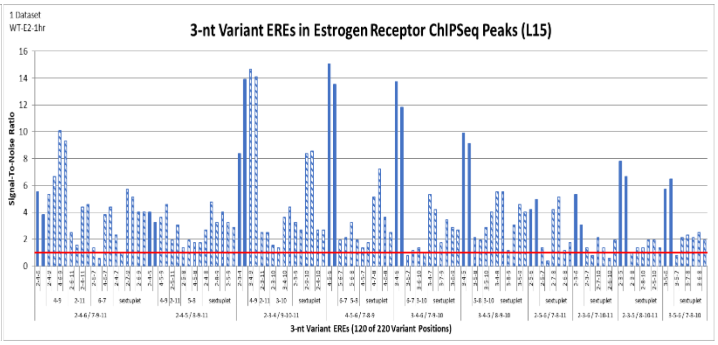

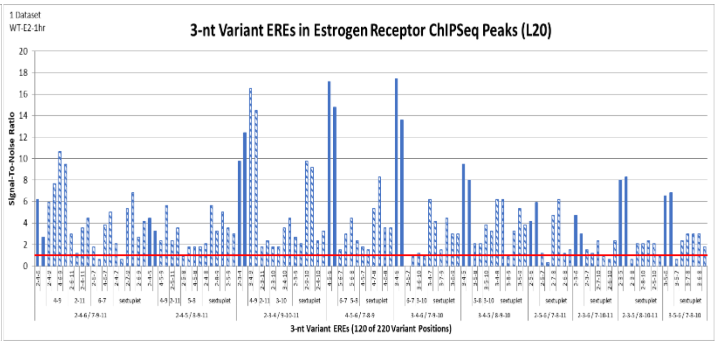

Figure S65. Compare (S/N) analysis of 4-nt Variant EREs in ER ChIPSeq Peaks (13-nt vs. 15-nt)

4-nt Variant 13-nt ERE DNA Elements

4-nt Variant 15-nt ERE DNA Elements

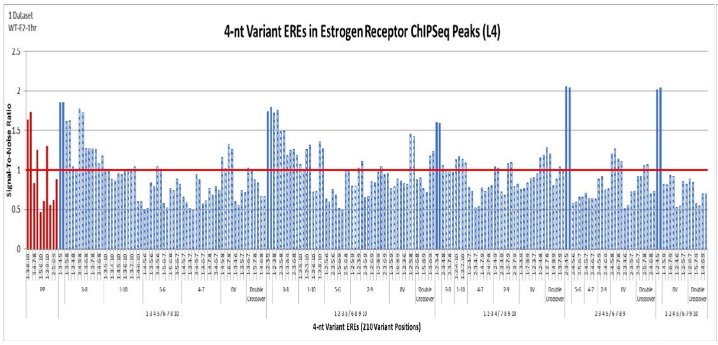

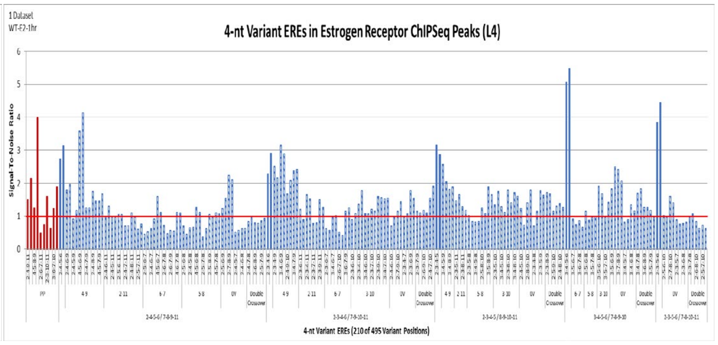

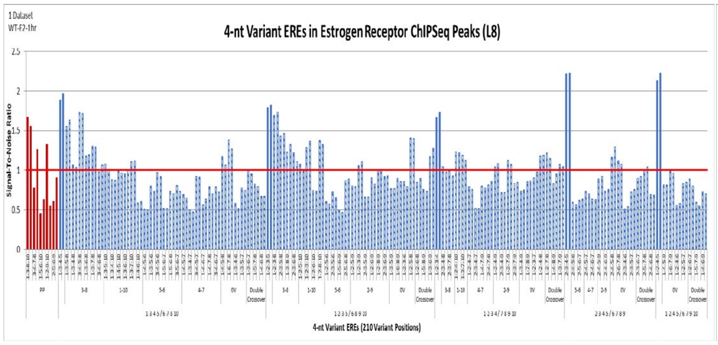

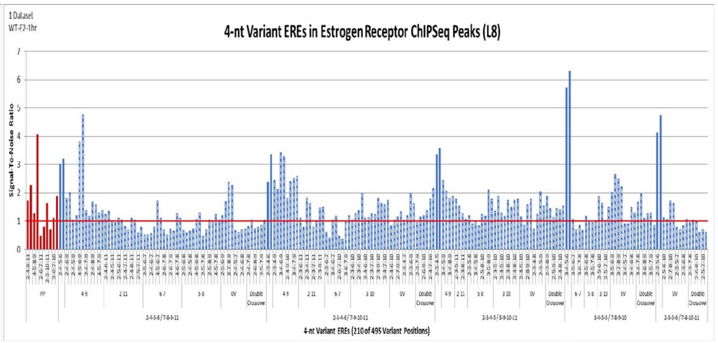

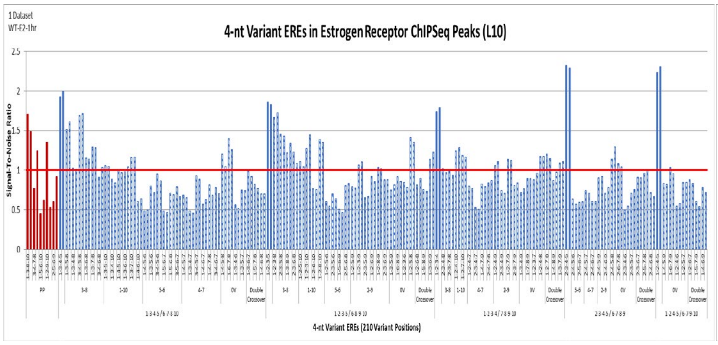

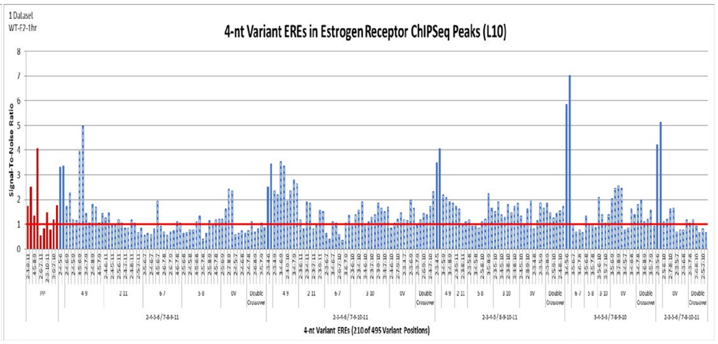

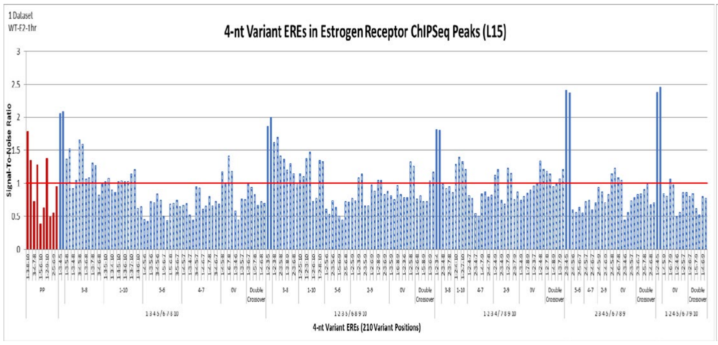

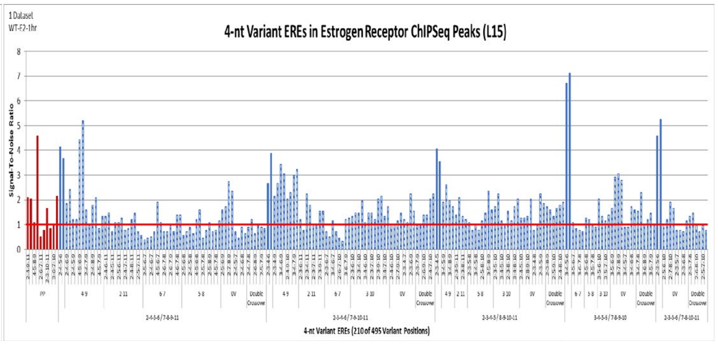

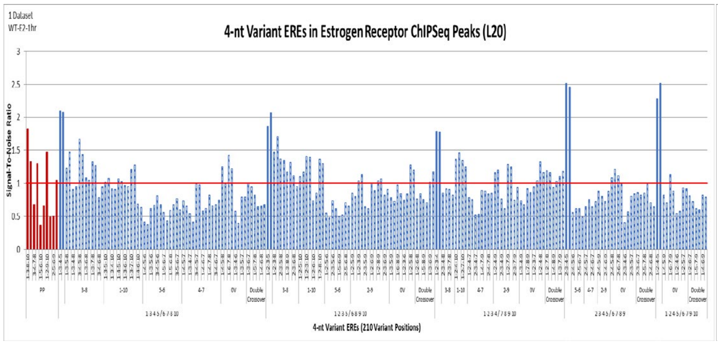

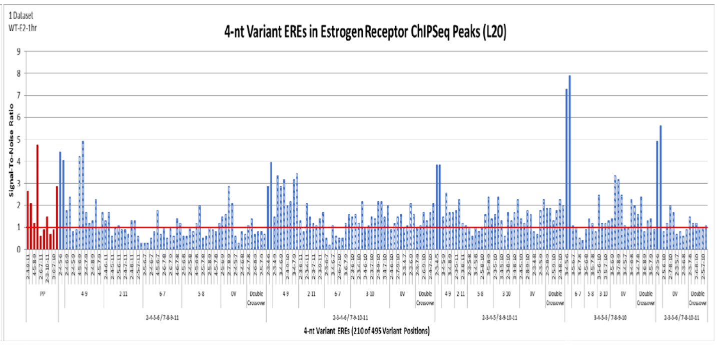

Figure S66. Compare (S/N) analysis of 5-nt Variant EREs in ER ChIPSeq Peaks (13-nt vs. 15-nt)

5-nt Variant 13-nt ERE DNA Elements **5-nt Variant 15-nt ERE DNA Elements**

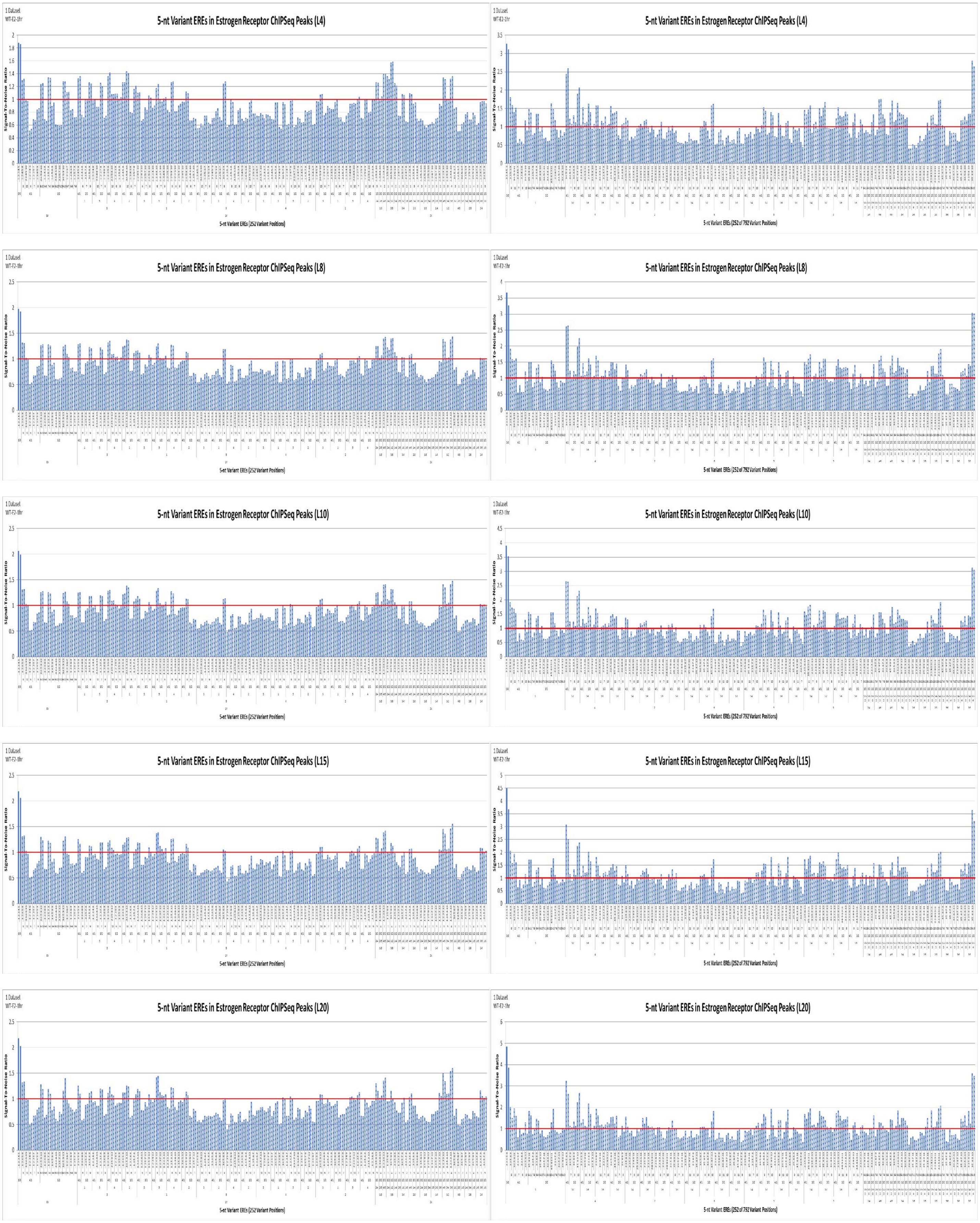

Figure S67. Compare (S/N) analysis of 1-nt Variant HREs in KR ChIPSeq Peaks (13-nt vs. 15-nt)

1-nt Variant 13-nt HRE DNA Elements

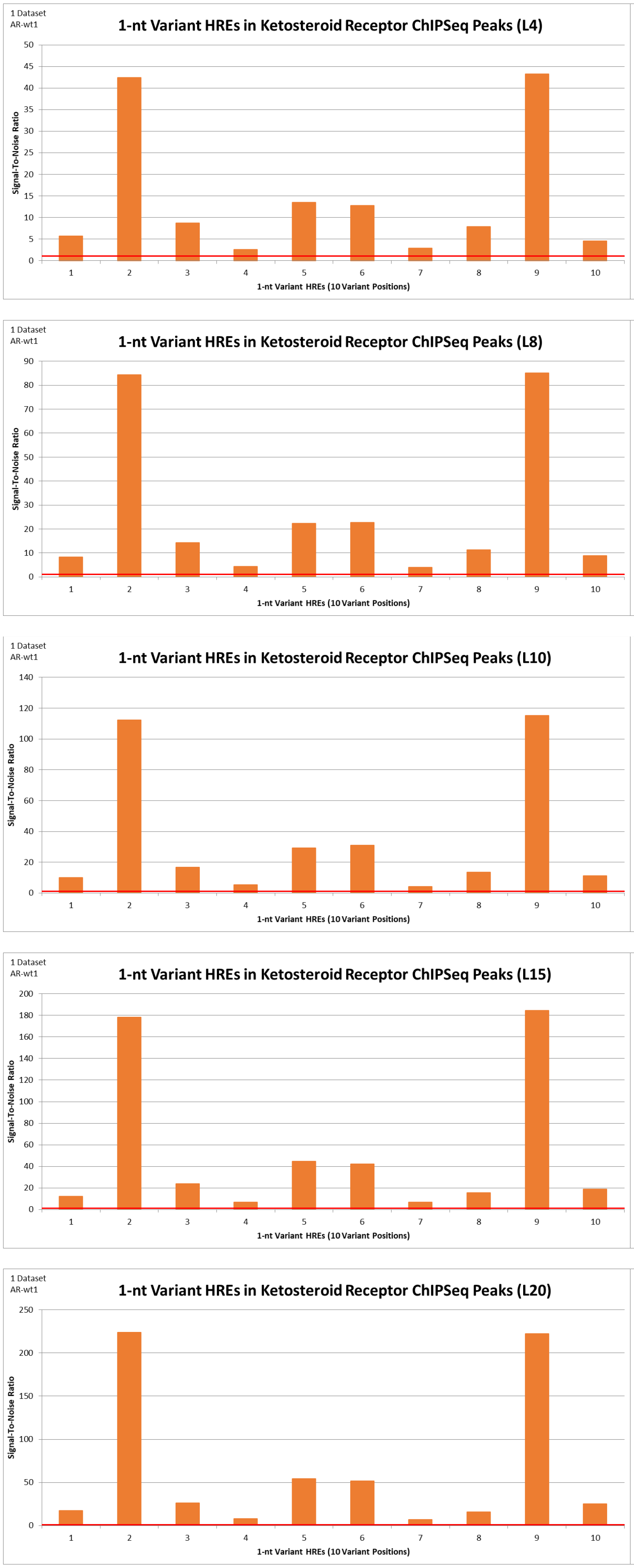

1-nt Variant 15-nt HRE DNA Elements

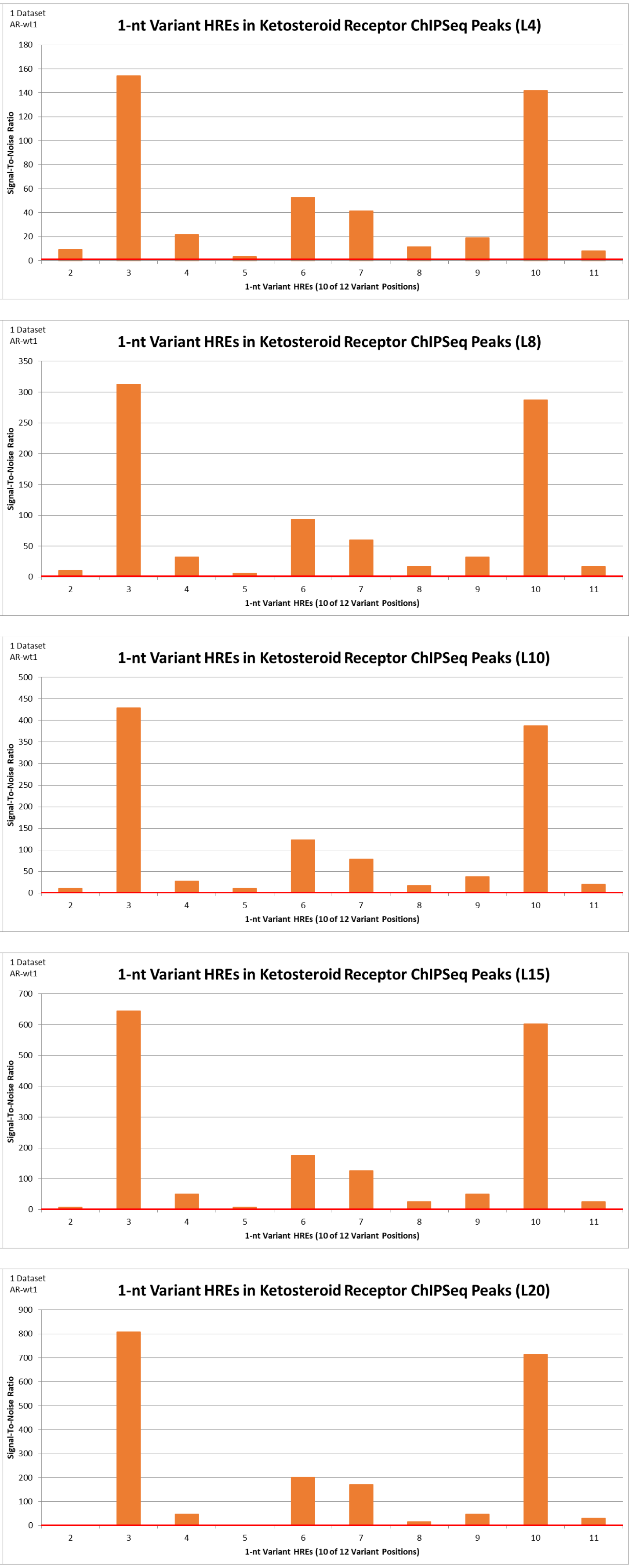

Figure S68. Compare (S/N) analysis of 2-nt Variant HREs in KR ChIPSeq Peaks (13-nt vs. 15-nt)

2-nt Variant 13-nt HRE DNA Elements

2-nt Variant 15-nt HRE DNA Elements

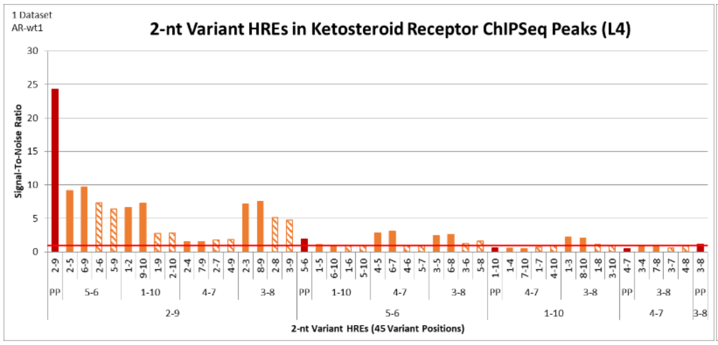

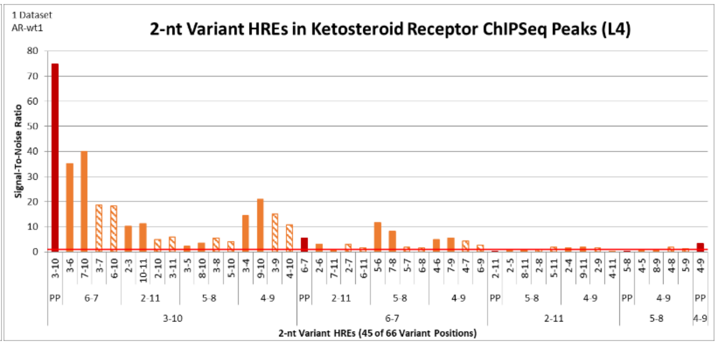

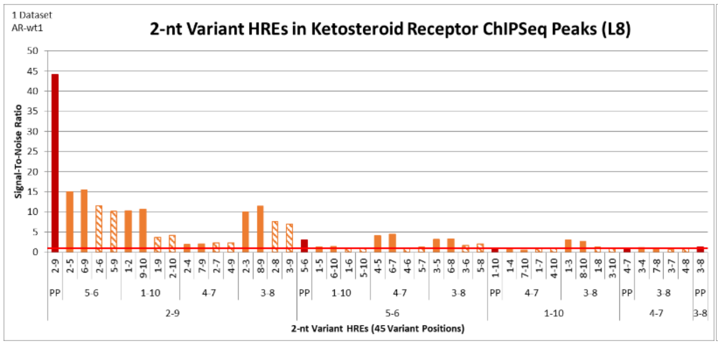

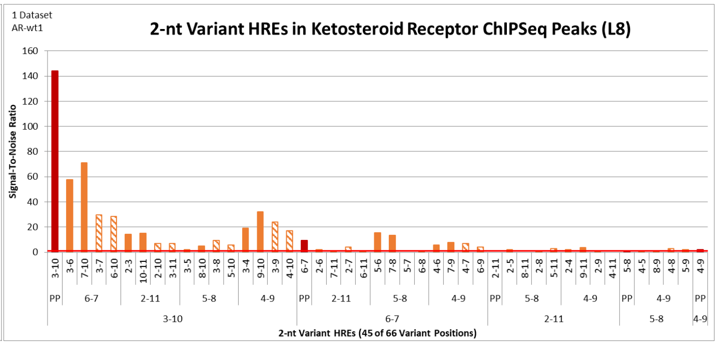

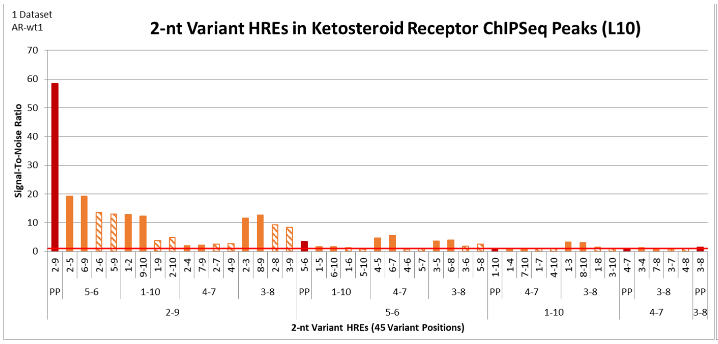

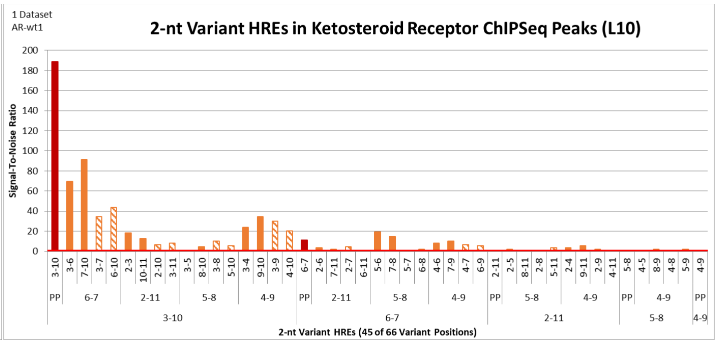

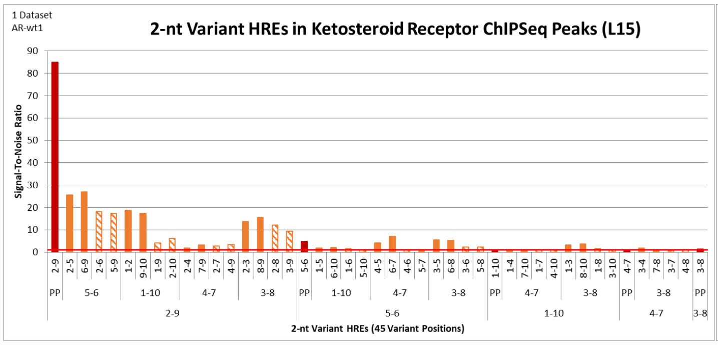

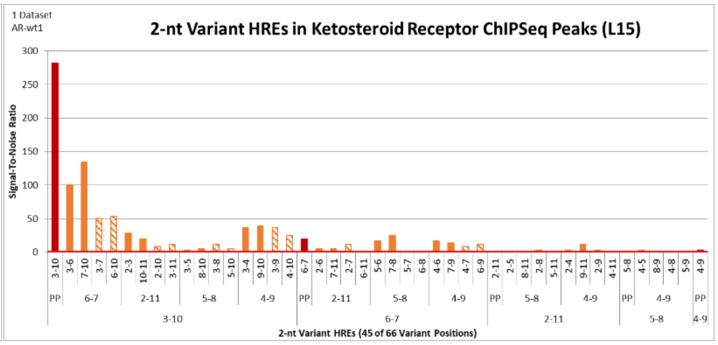

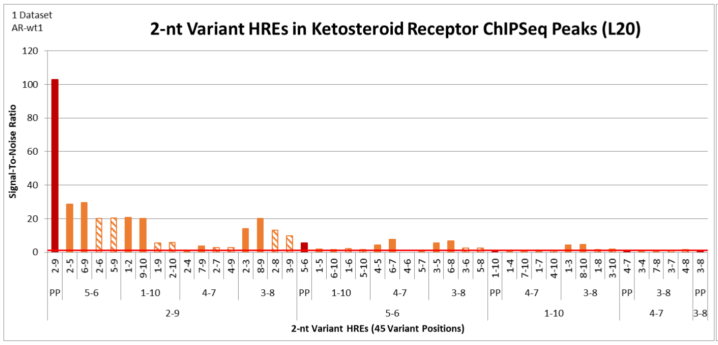

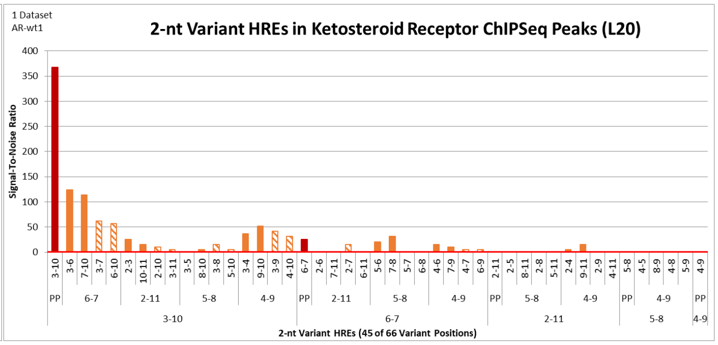

Figure S69. Compare (S/N) analysis of 3-nt Variant HREs in KR ChIPSeq Peaks (13-nt vs. 15-nt)

3-nt Variant 13-nt HRE DNA Elements

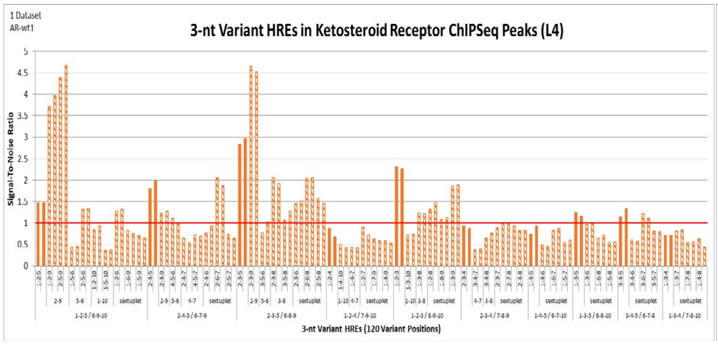

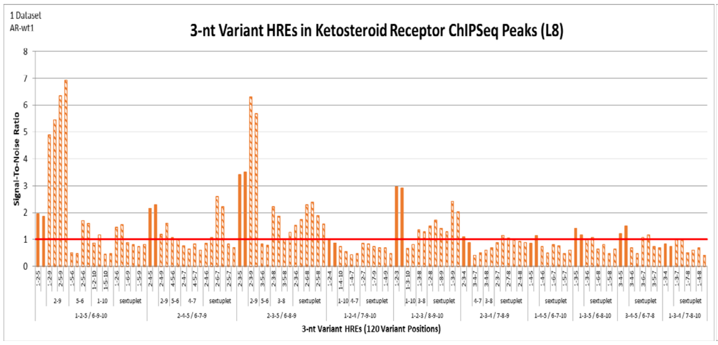

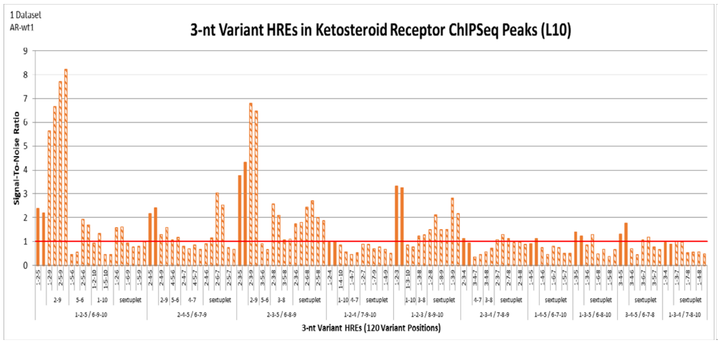

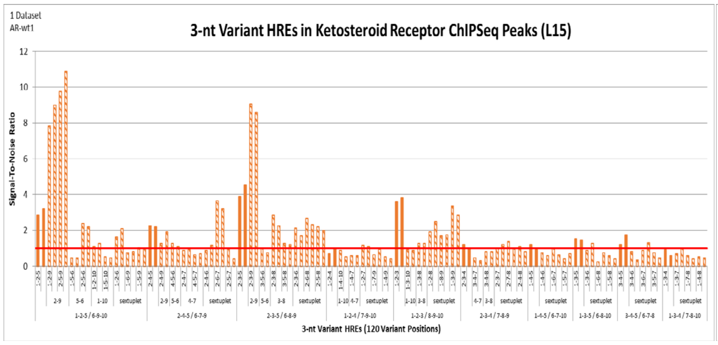

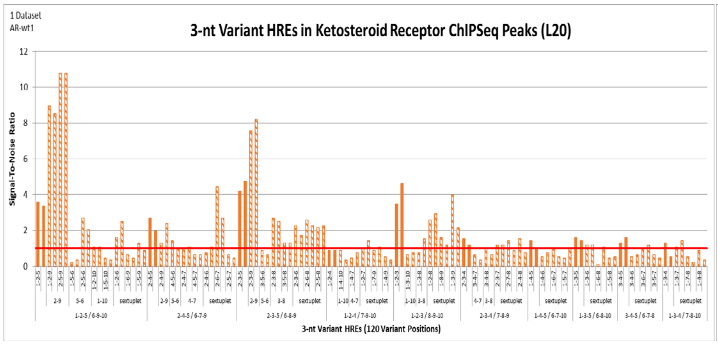

3-nt Variant 15-nt HRE DNA Elements

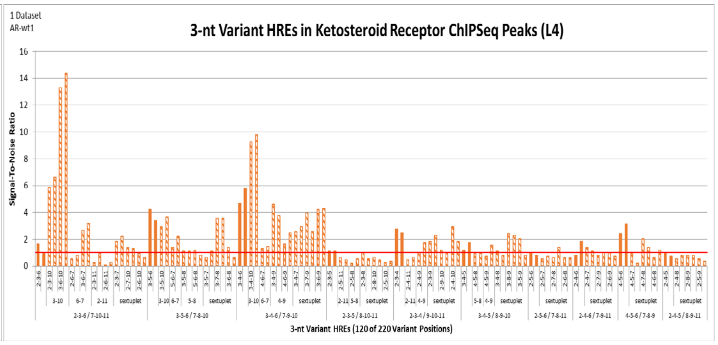

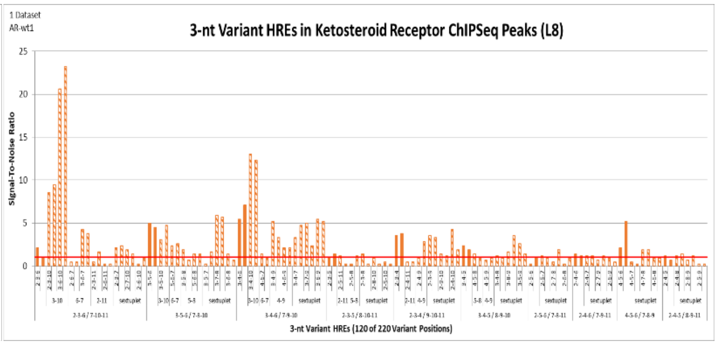

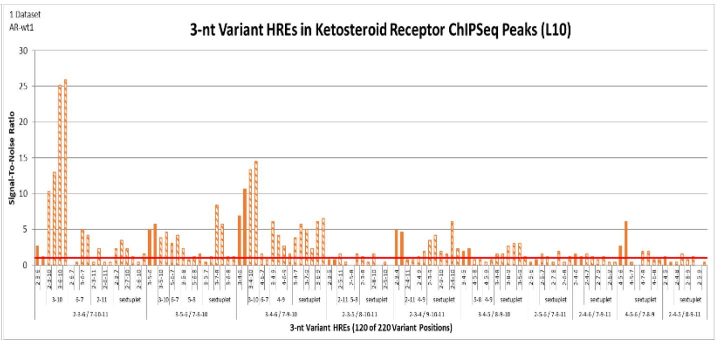

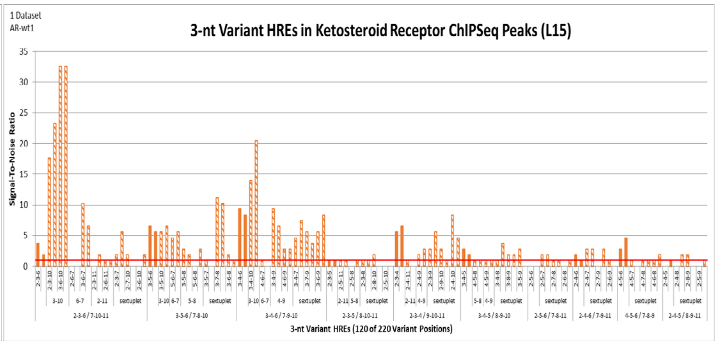

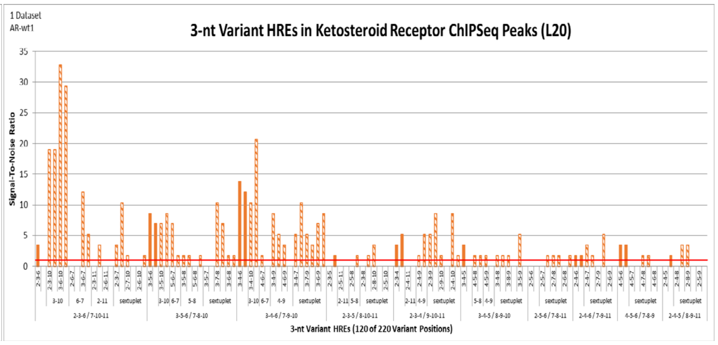

Figure S70. Compare (S/N) analysis of 4-nt Variant HREs in KR ChIPSeq Peaks (13-nt vs. 15-nt)

4-nt Variant 13-nt HRE DNA Elements

4-nt Variant 15-nt HRE DNA Elements

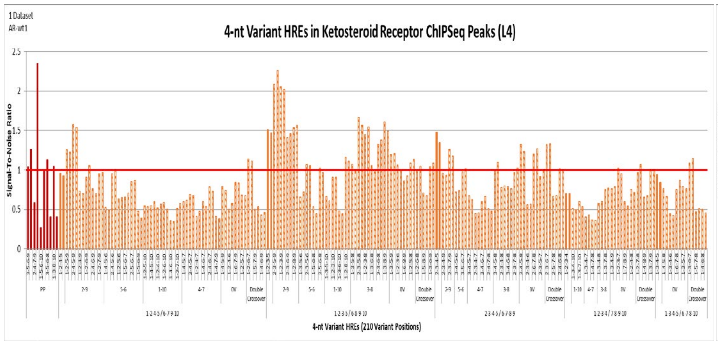

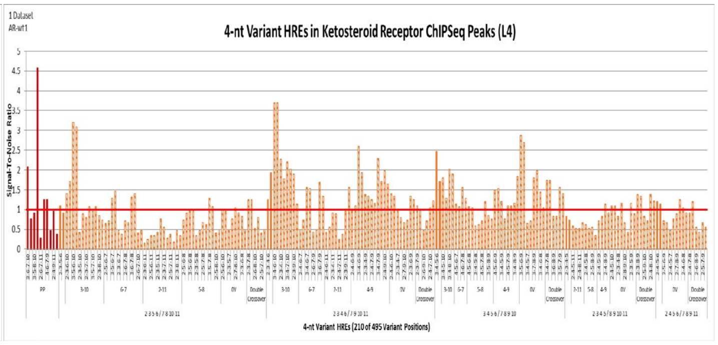

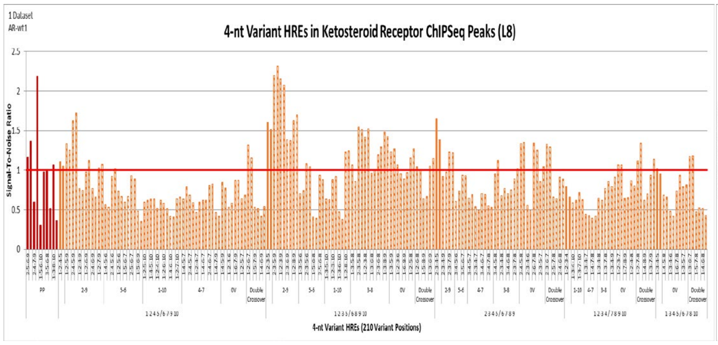

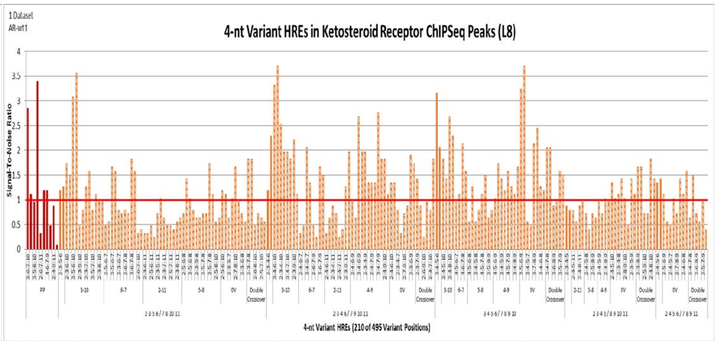

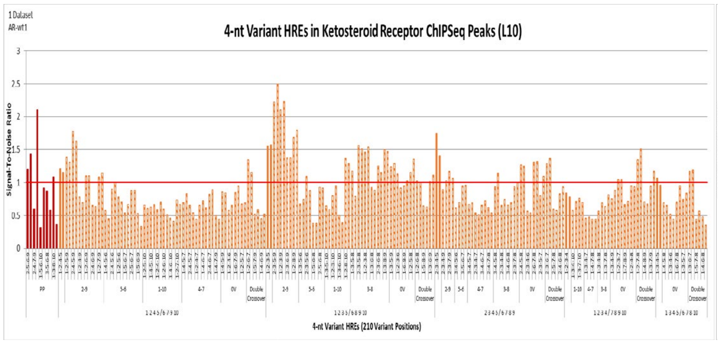

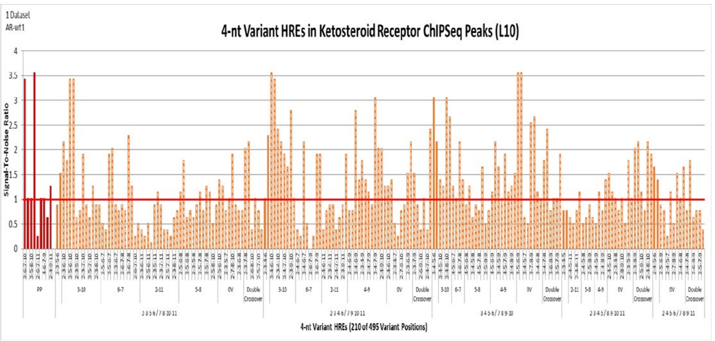

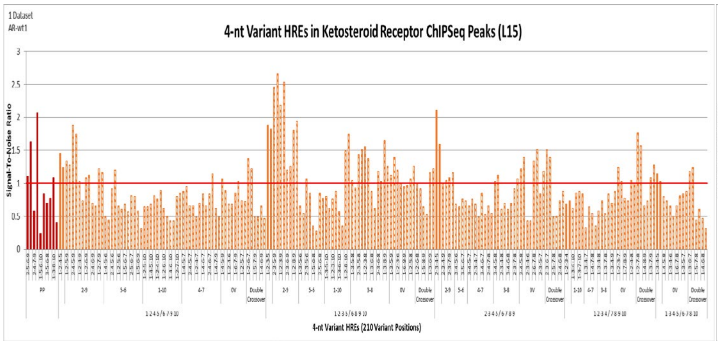

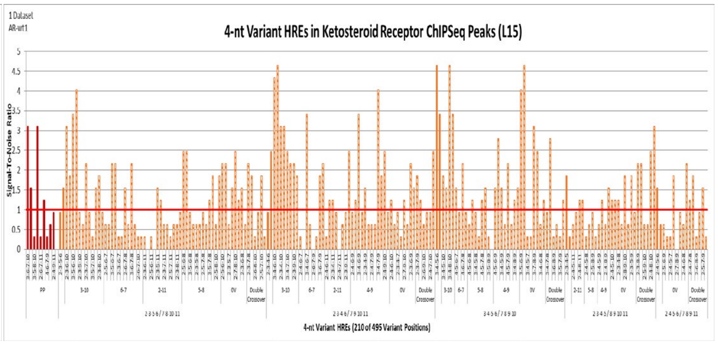

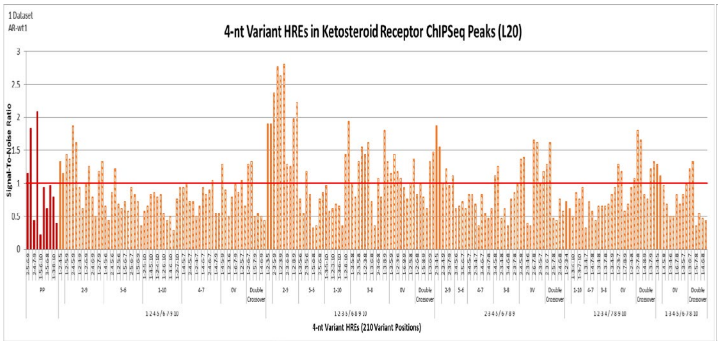

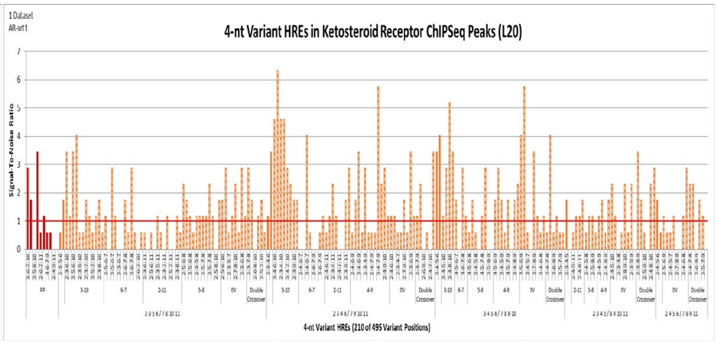

Figure S71. Compare (S/N) analysis of 5-nt Variant HREs in KR ChIPSeq Peaks (13-nt vs. 15-nt)

5-nt Variant 13-nt HRE DNA Elements

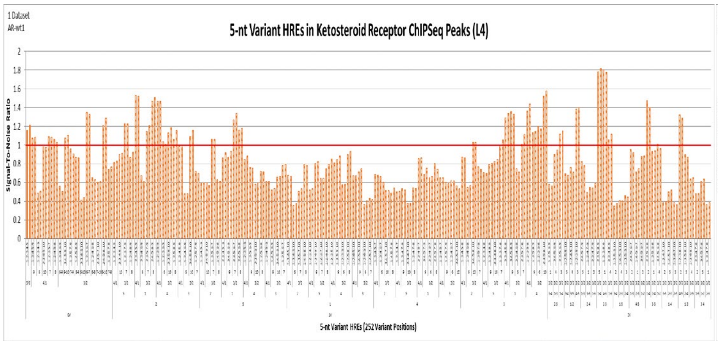

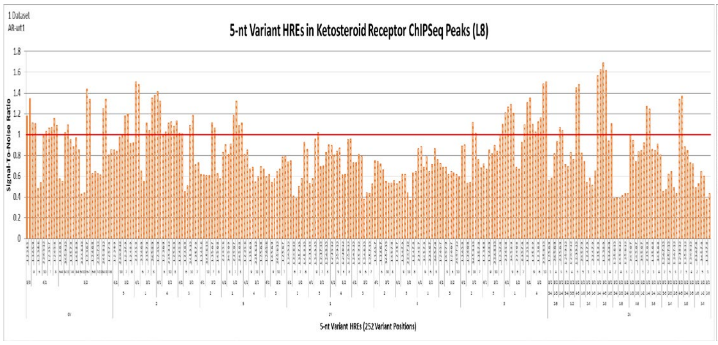

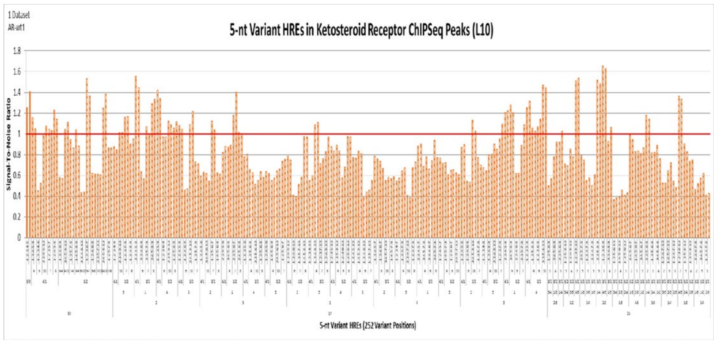

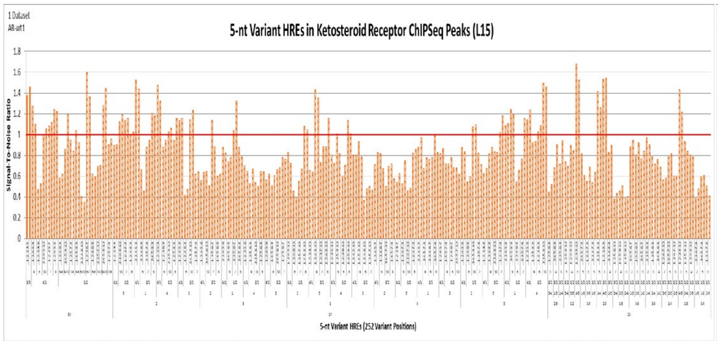

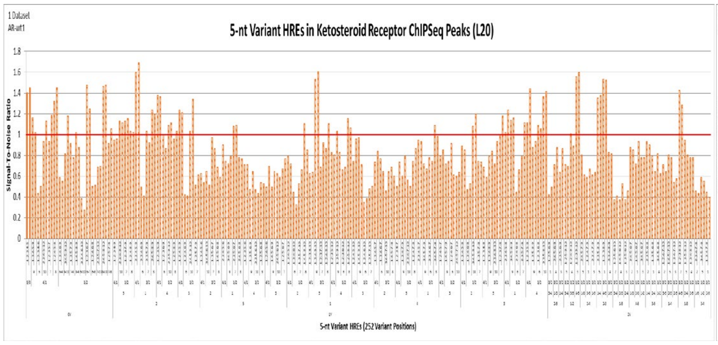

5-nt Variant 15-nt HRE DNA Elements

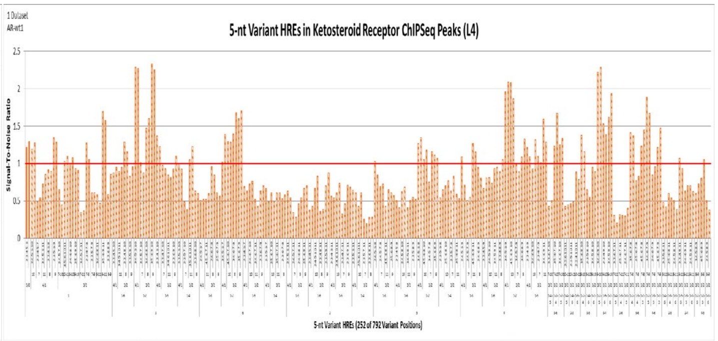

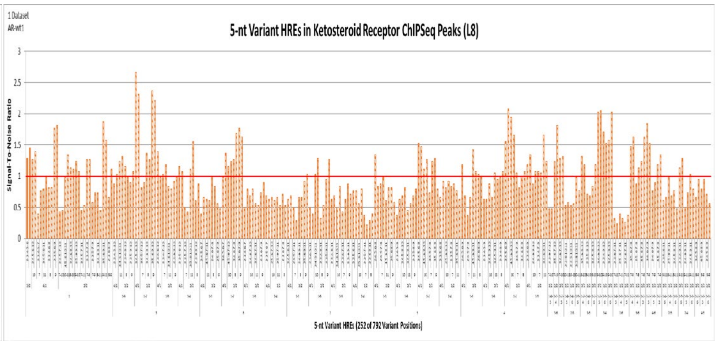

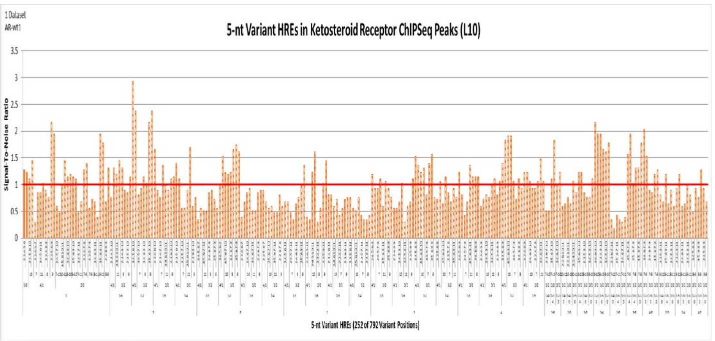

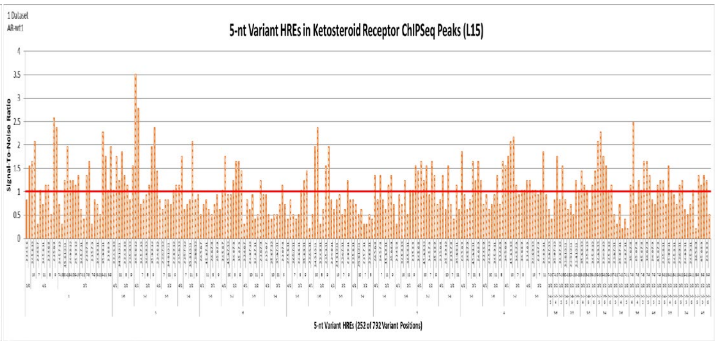

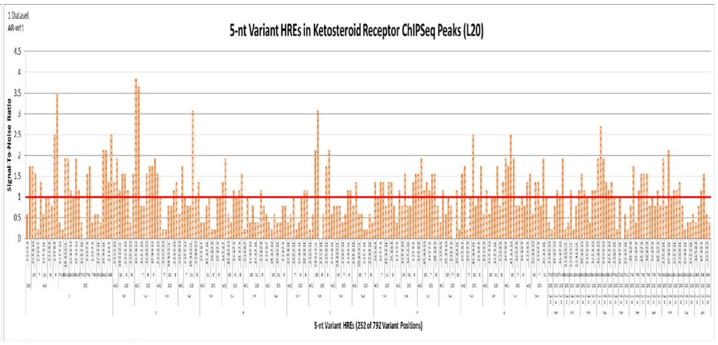

Figure S72-S77 Descriptions: Transform the 13-nt DNA Element Analysis to the 15-nt DNA Element Analysis

****All detailed data and statistics associated with every figure are compiled in Data S1****

Because the analysis of sNR DNA-binding at 13-nt ERE and HRE DNA elements was done by overlapping the location coordinates of each 0-nt to 5-nt variant ERE or HRE DNA element in the genome and the location coordinates of the ChIPSeq or ChIPExo peaks from each experiment, the counts of all sNR DNA-binding events at 15-nt ERE or HRE DNA elements in the genome are included in the 13-nt ERE and HRE DNA element analysis (i.e., every 13-nt ERE and HRE DNA element in the genome is part of a 15-nt ERE and HRE DNA element). Thus, assessing the role of the flanking nucleotides in a 15-nt ERE or HRE DNA element on sNR DNA-binding in the genome requires segregating the counts from the 13-nt ERE and HRE DNA element analysis and displaying them separately. Therefore, the 13-nt ERE or HRE DNA element with 10 primary positions is converted to a 15-nt ERE or HRE DNA element with 12 primary positions.

Analysis of sNR DNA-binding at 13-nt ERE and HRE DNA elements included the 1 0-nt variant consensus palindromic DNA element, 30 1-nt variant DNA elements (10 variant positions), 405 2-nt variant DNA elements (45 variant positions), 3,240 3-nt variant DNA elements (120 variant positions), 17,010 4-nt variant DNA elements (210 variant positions), and 61,236 5-nt variant DNA elements (252 variant positions), for a total of 81,922 DNA elements. Analysis of sNR DNA-binding at 15-nt ERE and HRE DNA elements include the 1 0-nt variant consensus palindromic DNA element, 36 1-nt variant DNA elements (12 variant positions), 594 2-nt variant DNA elements (66 variant positions), 5,940 3-nt variant DNA elements (220 variant positions), 40,095 4-nt variant DNA elements (495 variant positions), 192,456 5-nt variant DNA elements (792 variant positions), and 673,596 6-nt variant DNA elements (924 variant positions), for a total of 912,718 DNA elements.

Figure S72. Transform the 13-nt DNA Element Analysis to the 15-nt DNA Element Analysis
Each of the 81,922 0-nt to 5-nt variant 13-nt DNA elements splits into 16 categories of the 15-nt DNA elements. When an adenine (A) is in position 1 and a thymine (T) is in position 12 of the 15-nt DNA element, this is the 0-nt variant difference DNA element [=1 DNA element]. When one of the three (3) alternative nucleotide possibilities are in position 1 (C, G, T) or position 12 (A, C, G), these are 1-nt variant difference DNA elements [=6 DNA elements]. When one of the three (3) alternative nucleotide possibilities are in position 1 and position 12, these are 2-nt variant difference DNA elements [=9 DNA elements].

Using WT-E2-1hr.L4 [76,163 peaks, 146-nt peak length] as an example experiment, the 0-nt variant consensus palindromic 13-nt ERE DNA element (5′-GGTCAnnnTGACC-3′) occurred 1,202 times in a particular ER experiment (WT-E2-1hr.L4) (Figure S2). Each of those 1,202 0-nt variant consensus palindromic 13-nt ERE DNA elements are part of a 15-nt ERE DNA element. Thus, analysis of ER DNA-binding at all possible 15-nt ERE DNA elements splits those 1,202 0-nt variant consensus palindromic 13-nt ERE DNA elements into 16 categories (i.e., 4-nt possibilities in position 1 and 4-nt possibilities in position 12): 109 (1T), 155 (1G), 19 (1C), 13 (12G), 151 (12C), 110 (12A), 11 (1-12 TG), 67 (1-12 TC), 17 (1-12 GG), 305 (1-12 AT), 29 (1-12 TA), 107 (1-12 GC), 5 (1-12 CG), 14 (1-12 CC), 88 (1-12 GA), 2 (1-12 CA), totaling 1,202 **(Figure S33).**

However, there are some DNA elements that are unique to each list (i.e., the 81,922 0-nt to 5-nt variant 13-nt DNA elements and the 912,718 0-nt to 6-nt variant 15-nt DNA elements). The 81,922 0-nt to 5-nt variant 13-nt DNA element list includes 551,124 DNA elements that are not on the 912,718 0-nt to 6-nt variant 15-nt DNA element list (these represent a 7-nt variant of the 15-nt DNA elements). The 912,718 0-nt to 6-nt variant 15-nt DNA element list includes 153,090 DNA elements that are not on the 81,922 0-nt to 5-nt variant 13-nt DNA element list (these represent a 6-nt variant of the 13-nt DNA elements). Thus, of the 81,922 0-nt to 5-nt variant 13-nt DNA element list and the 912,718 0-nt to 6-nt variant 15-nt DNA element list, 759,628 DNA elements correspond to each other. The splitting of the 81,922 0-nt to 5-nt variant 13-nt ERE and HRE DNA elements into the 16 categories of the 15-nt ERE and HRE DNA elements can be found in **Table S41-S46**.

Using WT-E2-1hr.L4 [76,163 peaks, 146-nt peak length] as an example experiment, there are 827,919 0-nt to 5-nt variant 13-nt EREs identified by overlapping the location coordinates of the 81,922 0-nt to 5-nt variant 13-nt EREs in the genome and the location coordinates of the ChIPSeq peaks in this experiment. There are 605,166 0-nt to 6-nt variant 15-nt EREs identified by overlapping the location coordinates of the 912,718 0-nt to 6-nt variant 15-nt EREs in the genome and the location coordinates of the ChIPSeq peaks in this experiment. Of the 605,166 0-nt to 6-nt variant 15-nt EREs identified in the experiment, 95,296 of those are the 153,090 DNA elements that are unique to the 15-nt ERE list (i.e., they are 6-nt variant 15-nt variant EREs that categorize to 6-nt variant 13-nt variant EREs) (=509,870). Of the 827,919 0-nt to 5-nt variant 13-nt EREs identified, 314,956 of those are the 551,124 DNA elements that are unique to the 13-nt ERE list (i.e., they are 5-nt variant 13-nt variant EREs that categorize to 7-nt variant 15-nt variant EREs) (=512,963). Of those 512,963 0-nt to 5-nt variant 13-nt EREs, 3093 of them were missing from the 15-nt ERE analysis due to their location being at the ends of the ChIPSeq peaks (i.e., the entire 13-nt DNA element was required to be within the peak boundaries for the 13-nt ERE analysis and the entire 15-nt DNA element was required to be within the peak boundaries for the 15-nt ERE analysis) (=509,870). Thus, in the WT-E2-1hr.L4 experiment, 509,870 are legitimately represented in both the 13-nt DNA element analysis and 15-nt DNA element analysis.

Figure S73. Transform the 13-nt DNA Element Analysis to the 15-nt DNA Element Analysis (1-nt Variant EREs)
Splitting (S/N) analysis of 1-nt variant 13-nt EREs in ER (WT-E2-1hr) [76,163 peaks, 146-nt peak length] ChIPSeq peaks into the 16 categories of the 15-nt ERE DNA elements (i.e., 1 stays in the 0-nt variant difference category, 6 move to the 1-nt variant difference category, 9 move to the 2-nt variant difference category). The distribution ratio into each of the three categories should stay constant if the two flanking nucleotides of the 15-nt DNA element are not playing any role. Therefore, although the DNA-binding profile at the 13-nt DNA elements in the genome versus at the 15-nt DNA elements in the genome are almost equivalent (confirming that the DNA-binding rules established by the 13-nt DNA element analysis also apply to the 15-nt DNA element analysis), there are minor effects. For example, by fixing the flanking nucleotides of the 15-nt ERE (adenine (A) is in position 1 and thymine (T) is in position 12), ER DNA-binding is reduced at 15-nt EREs that have variants in position 2 or position 11.

Figure S74. Transform the 13-nt DNA Element Analysis to the 15-nt DNA Element Analysis (2-nt Variant EREs)
Splitting (S/N) analysis of 2-nt variant 13-nt EREs in ER (WT-E2-1hr) [76,163 peaks, 146-nt peak length] ChIPSeq peaks into the 16 categories of the 15-nt ERE DNA elements (i.e., 1 stays in the 0-nt variant difference category, 6 move to the 1-nt variant difference category, 9 move to the 2-nt variant difference category). The distribution ratio into each of the three categories should stay constant if the two flanking nucleotides of the 15-nt DNA element are not playing any role. Therefore, although the DNA-binding profile at the 13-nt DNA elements in the genome versus at the 15-nt DNA elements in the genome are almost equivalent (confirming that the DNA-binding rules established by the 13-nt DNA element analysis also apply to the 15-nt DNA element analysis), there are minor effects.

Figure S75. Transform the 13-nt DNA Element Analysis to the 15-nt DNA Element Analysis (3-nt Variant EREs)
Splitting (S/N) analysis of 3-nt variant 13-nt EREs in ER (WT-E2-1hr) [76,163 peaks, 146-nt peak length] ChIPSeq peaks into the 16 categories of the 15-nt ERE DNA elements (i.e., 1 stays in the 0-nt variant difference category, 6 move to the 1-nt variant difference category, 9 move to the 2-nt variant difference category). The distribution ratio into each of the three categories should stay constant if the two flanking nucleotides of the 15-nt DNA element are not playing any role. Therefore, although the DNA-binding profile at the 13-nt DNA elements in the genome versus at the 15-nt DNA elements in the genome are almost equivalent (confirming that the DNA-binding rules established by the 13-nt DNA element analysis also apply to the 15-nt DNA element analysis), there are minor effects.

Figure S76. Transform the 13-nt DNA Element Analysis to the 15-nt DNA Element Analysis (4-nt Variant EREs)
Splitting (S/N) analysis of 4-nt variant 13-nt EREs in ER (WT-E2-1hr) [76,163 peaks, 146-nt peak length] ChIPSeq peaks into the 16 categories of the 15-nt ERE DNA elements (i.e., 1 stays in the 0-nt variant difference category, 6 move to the 1-nt variant difference category, 9 move to the 2-nt variant difference category). The distribution ratio into each of the three categories should stay constant if the two flanking nucleotides of the 15-nt DNA element are not playing any role. Therefore, although the DNA-binding profile at the 13-nt DNA elements in the genome versus at the 15-nt DNA elements in the genome are almost equivalent (confirming that the DNA-binding rules established by the 13-nt DNA element analysis also apply to the 15-nt DNA element analysis), there are minor effects.

Figure S77. Transform the 13-nt DNA Element Analysis to the 15-nt DNA Element Analysis (5-nt Variant EREs)
Splitting (S/N) analysis of 5-nt variant 13-nt EREs in ER (WT-E2-1hr) [76,163 peaks, 146-nt peak length] ChIPSeq peaks into the 16 categories of the 15-nt ERE DNA elements (i.e., 1 stays in the 0-nt variant difference category, 6 move to the 1-nt variant difference category, 9 move to the 2-nt variant difference category). The distribution ratio into each of the three categories should stay constant if the two flanking nucleotides of the 15-nt DNA element are not playing any role. Therefore, although the DNA-binding profile at the 13-nt DNA elements in the genome versus at the 15-nt DNA elements in the genome are almost equivalent (confirming that the DNA-binding rules established by the 13-nt DNA element analysis also apply to the 15-nt DNA element analysis), there are minor effects. The green stripped bars represent the 314,956 counts that are 5-nt variant 13-nt variant EREs that represent 7-nt variant 15-nt variant EREs (i.e., the 551,124 DNA elements that are unique to the 13-nt ERE list).

Figure S72. Transform the 13-nt DNA Element Analysis to the 15-nt DNA Element Analysis

			15-nt DNA Element Analysis								
			**1**	**36**	**594**	**5,940**	**40,095**	**192,456**	**673,596**	**1,732,104**	
			**0-nt Variant**	**1-nt Variant**	**2-nt Variant**	**3-nt Variant**	**4-nt Variant**	**5-nt Variant**	**6-nt Variant**	***7-nt Variant***	
**13-nt DNA Element Analysis**	**1**	**0-nt Variant**	1	6	9						**16**
	**30**	**1-nt Variant**		30	180	270					**480**
	**405**	**2-nt Variant**			405	2,430	3,645				**6,480**
	**3,240**	**3-nt Variant**				3,240	19,440	29,160			**51,840**
	**17,010**	**4-nt Variant**					17,010	102,060	153,090		**272,160**
	**61,236**	**5-nt Variant**						61,236	367,416	551,124	**428,652**
	153,090	***6-nt Variant***							153,090		
											**759,628**

Figure S73. Transform the 13-nt DNA Element Analysis to the 15-nt DNA Element Analysis (1-nt Variant EREs)

1-nt Variant 13-nt ERE DNA Elements

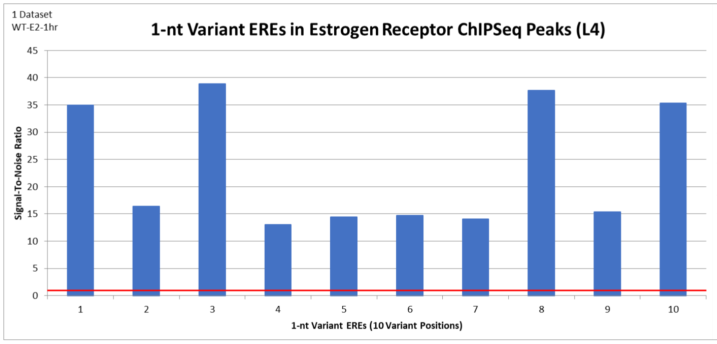

1-nt Variant 15-nt ERE DNA Elements

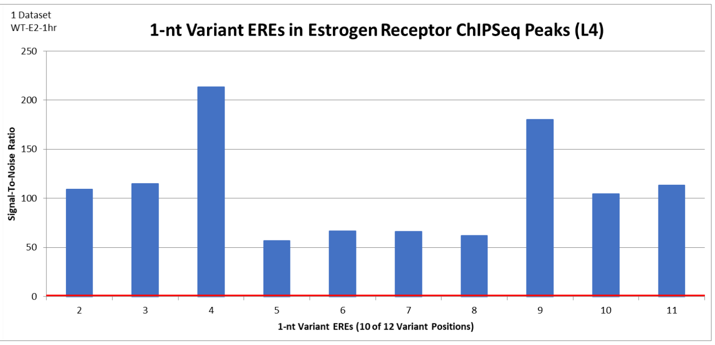

Categorize (S/N) Analysis of ER DNA-Binding at 1-nt Variant 13-nt EREs Into Three Categories of 15-nt EREs

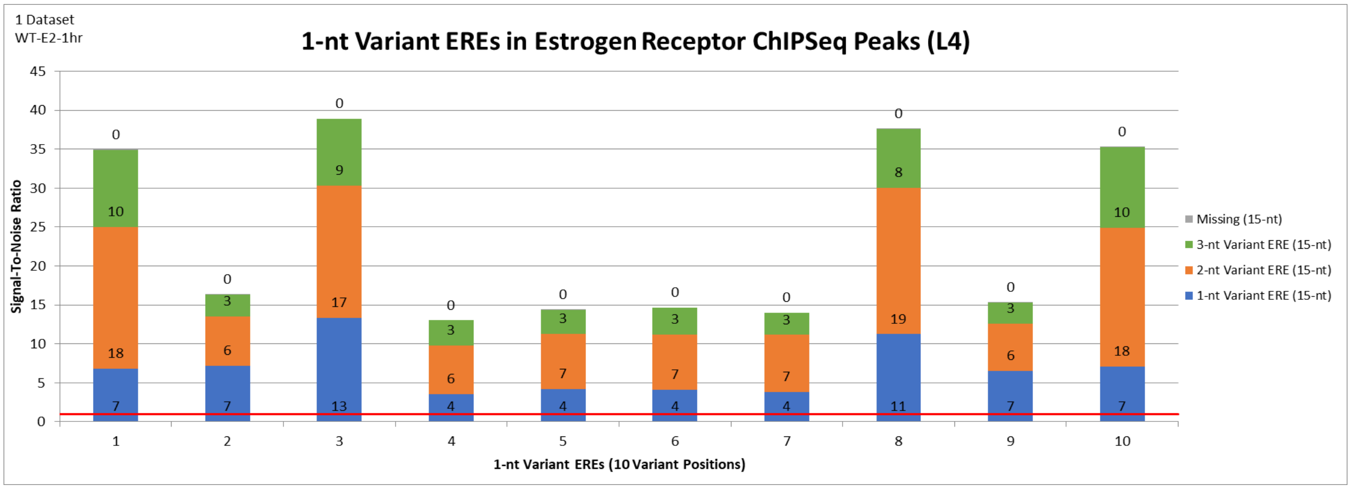

Categorize (S/N) Analysis of ER DNA-Binding at 1-nt Variant 13-nt EREs Into Three Categories of 15-nt EREs (% of (S/N))

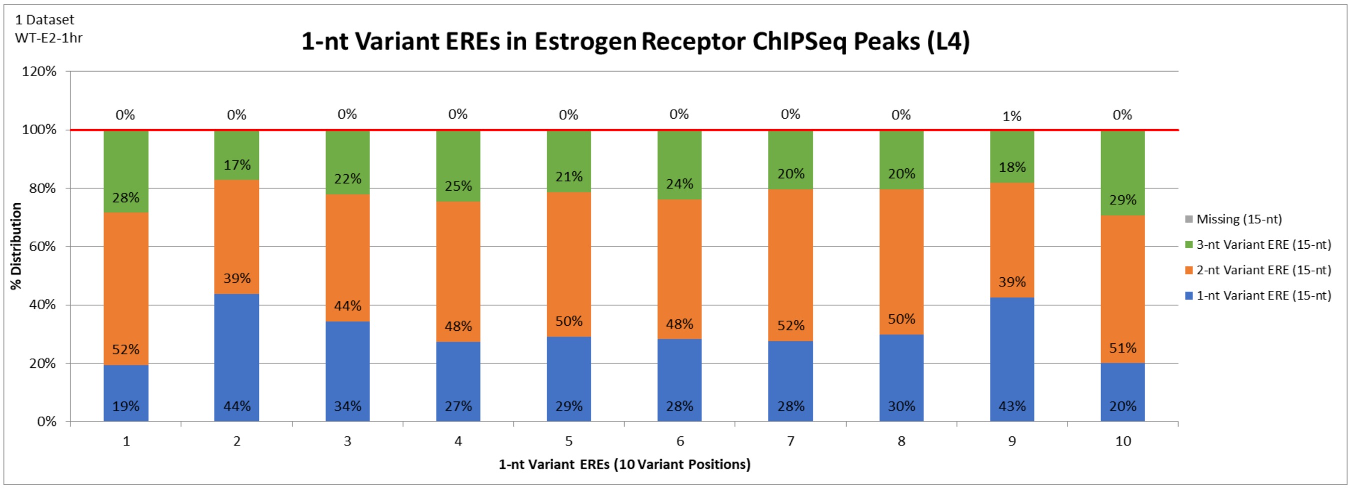

Figure S74. Transform the 13-nt DNA Element Analysis to the 15-nt DNA Element Analysis (2-nt Variant EREs)

2-nt Variant 13-nt ERE DNA Elements

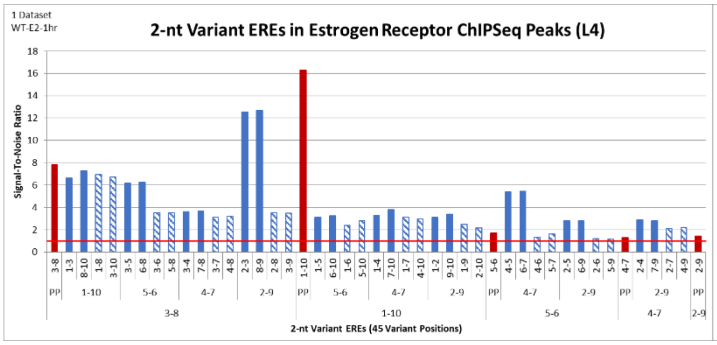

2-nt Variant 15-nt ERE DNA Elements

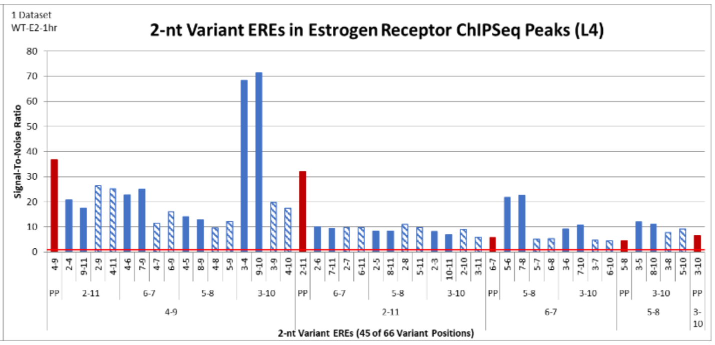

Categorize (S/N) Analysis of ER DNA-Binding at 2-nt Variant 13-nt EREs Into Three Categories of 15-nt EREs

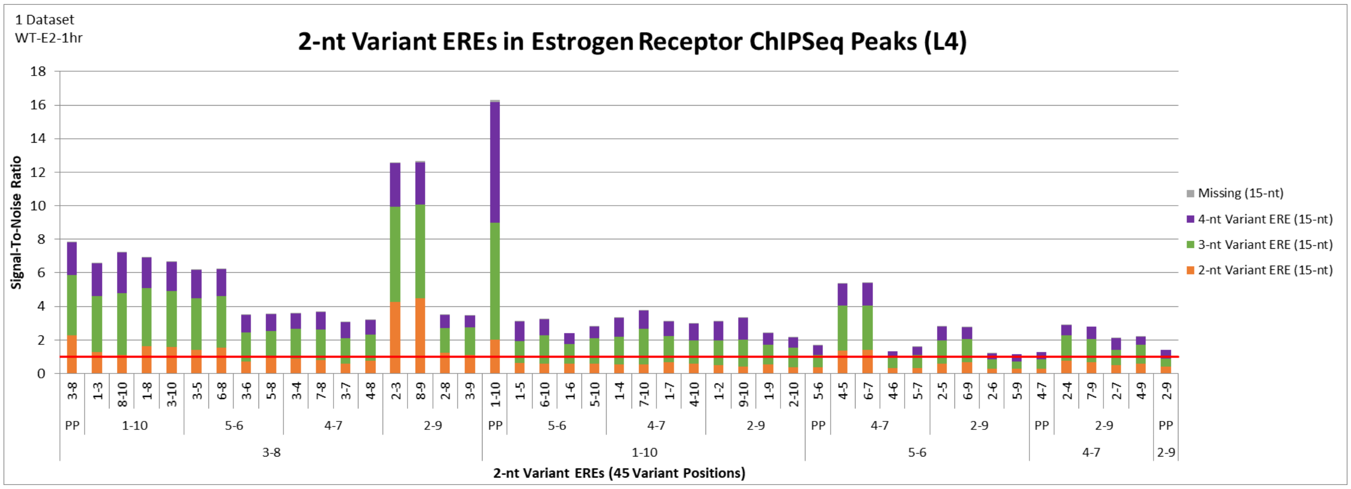

Categorize (S/N) Analysis of ER DNA-Binding at 2-nt Variant 13-nt EREs Into Three Categories of 15-nt EREs (% of (S/N))

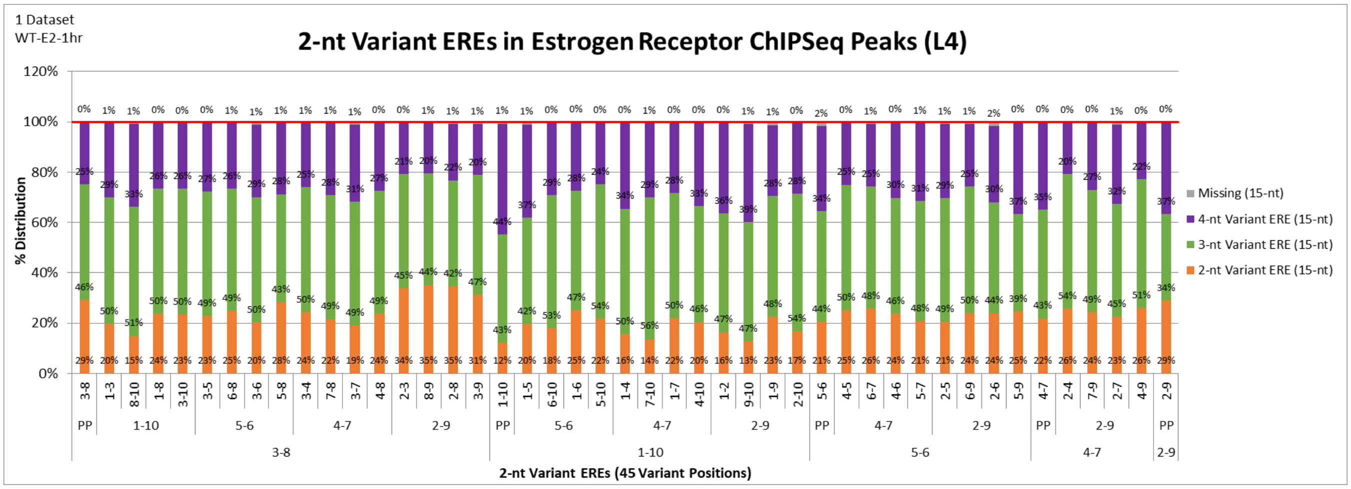

Figure S75. Transform the 13-nt DNA Element Analysis to the 15-nt DNA Element Analysis (3-nt Variant EREs)

3-nt Variant 13-nt ERE DNA Elements

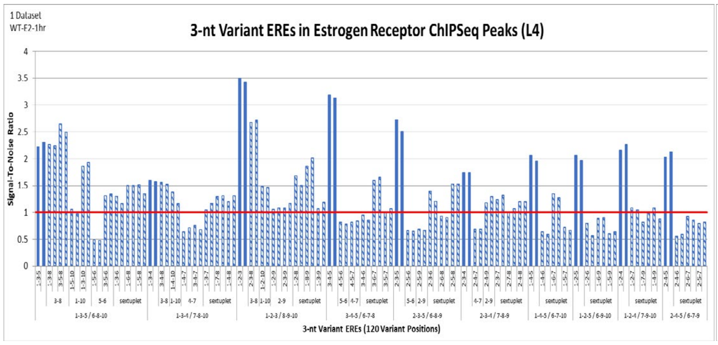

3-nt Variant 15-nt ERE DNA Elements

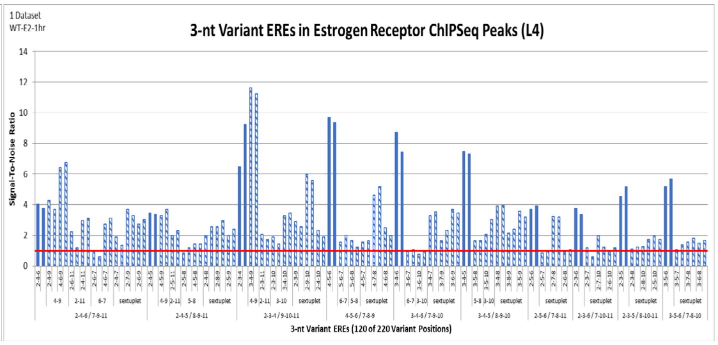

Categorize (S/N) Analysis of ER DNA-Binding at 3-nt Variant 13-nt EREs Into Three Categories of 15-nt EREs

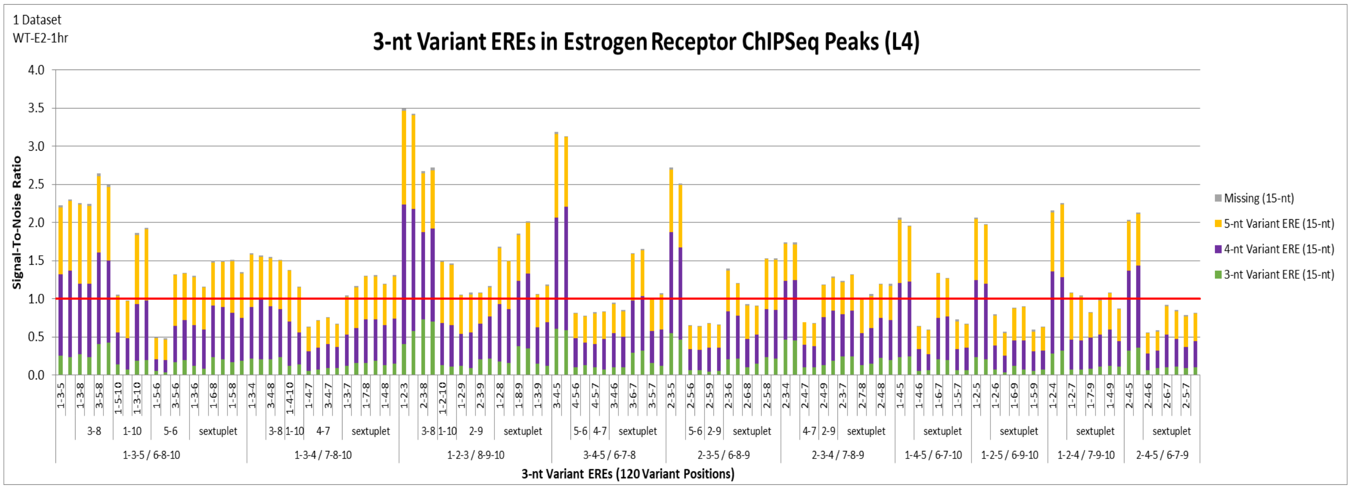

Categorize (S/N) Analysis of ER DNA-Binding at 3-nt Variant 13-nt EREs Into Three Categories of 15-nt EREs (% of (S/N))

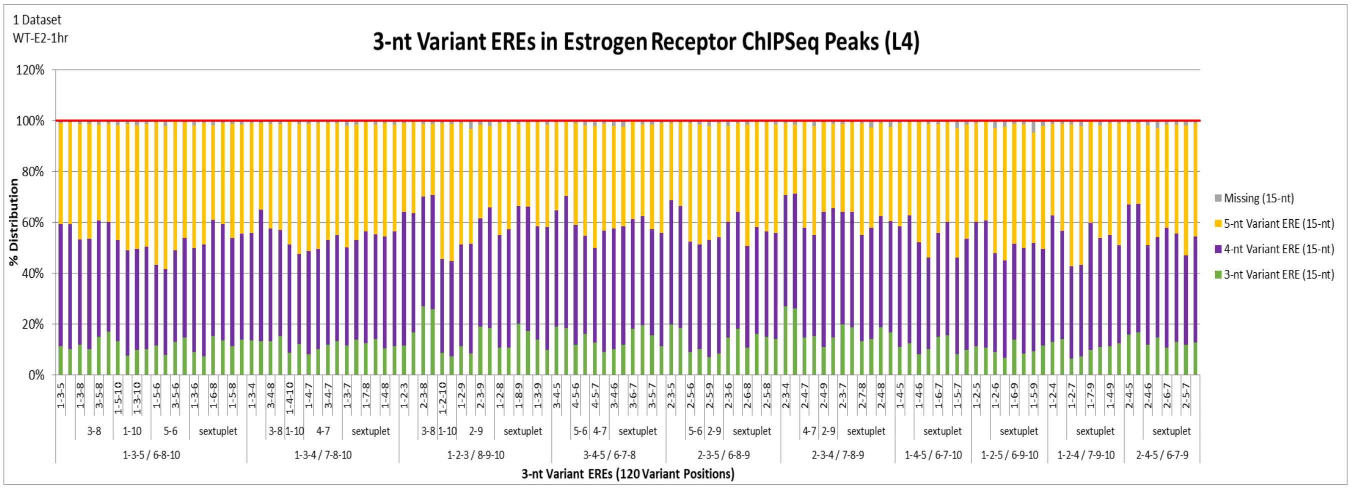

Figure S76. Transform the 13-nt DNA Element Analysis to the 15-nt DNA Element Analysis (4-nt Variant EREs)

4-nt Variant 13-nt ERE DNA Elements

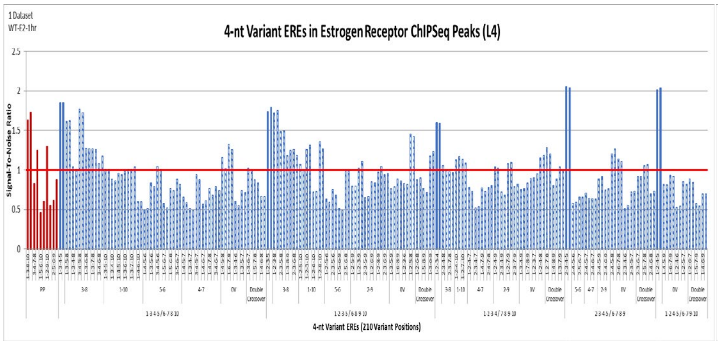

4-nt Variant 15-nt ERE DNA Elements

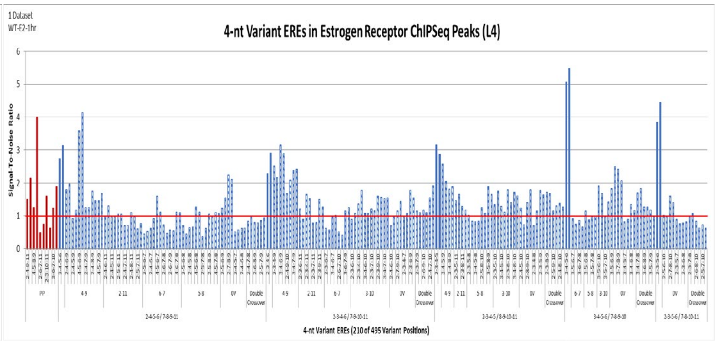

Categorize (S/N) Analysis of ER DNA-Binding at 4-nt Variant 13-nt EREs Into Three Categories of 15-nt EREs

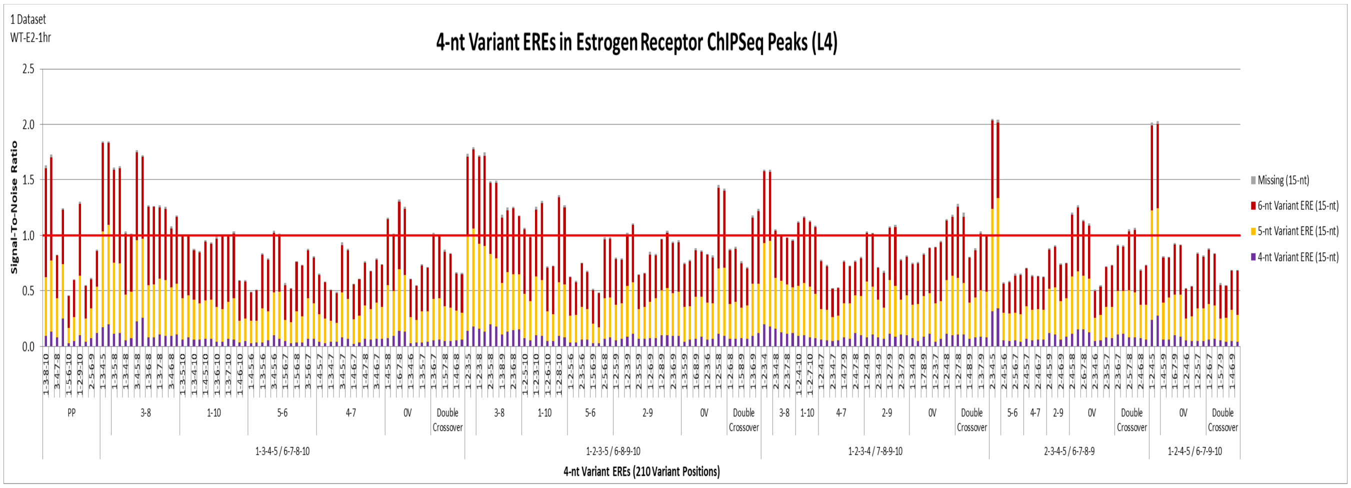

Categorize (S/N) Analysis of ER DNA-Binding at 4-nt Variant 13-nt EREs Into Three Categories of 15-nt EREs (% of (S/N))

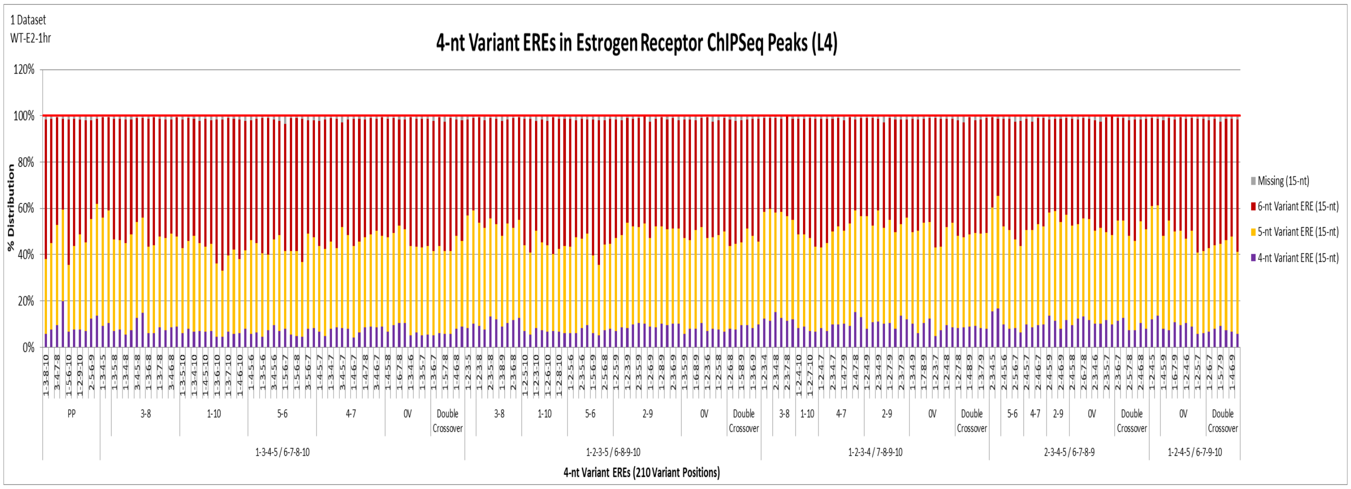

Figure S77. Transform the 13-nt DNA Element Analysis to the 15-nt DNA Element Analysis (5-nt Variant EREs)

5-nt Variant 13-nt ERE DNA Elements

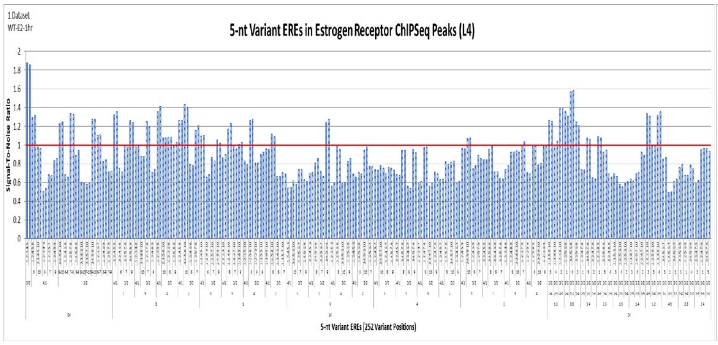

5-nt Variant 15-nt ERE DNA Elements

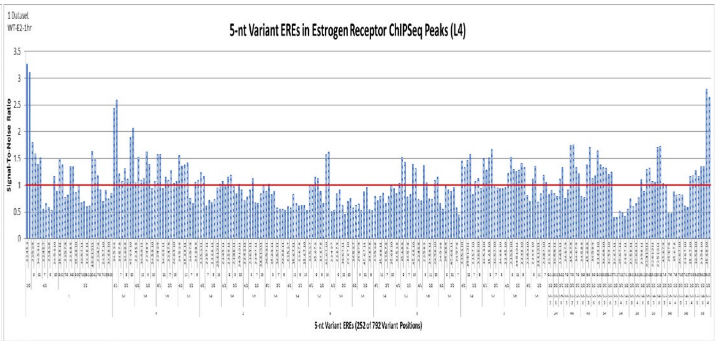

Categorize (S/N) Analysis of ER DNA-Binding at 5-nt Variant 13-nt EREs Into Three Categories of 15-nt EREs

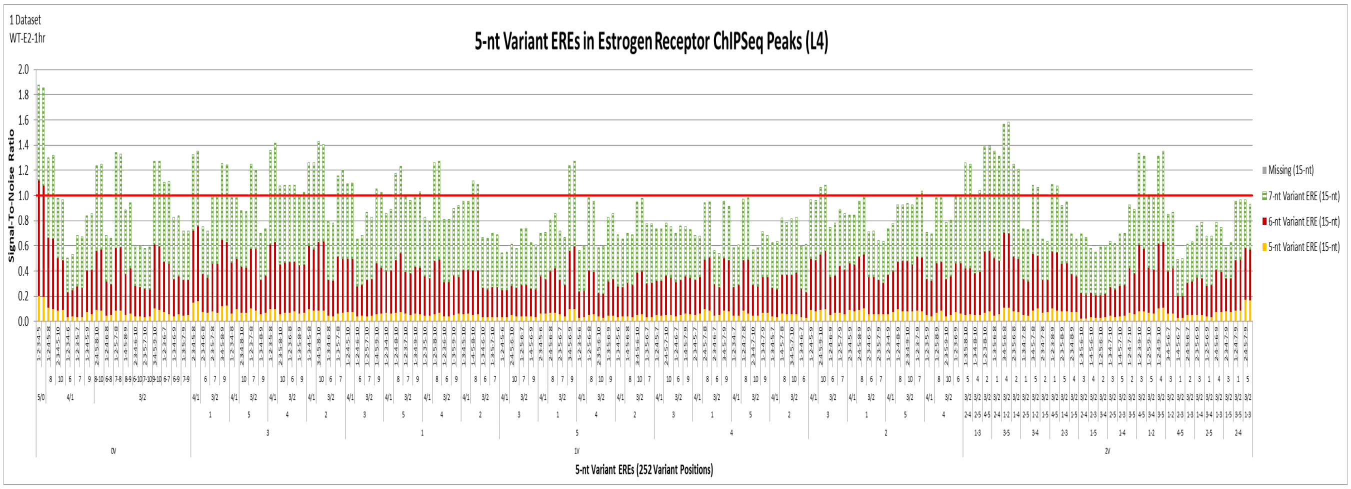

Categorize (S/N) Analysis of ER DNA-Binding at 5-nt Variant 13-nt EREs Into Three Categories of 15-nt EREs (% of (S/N))

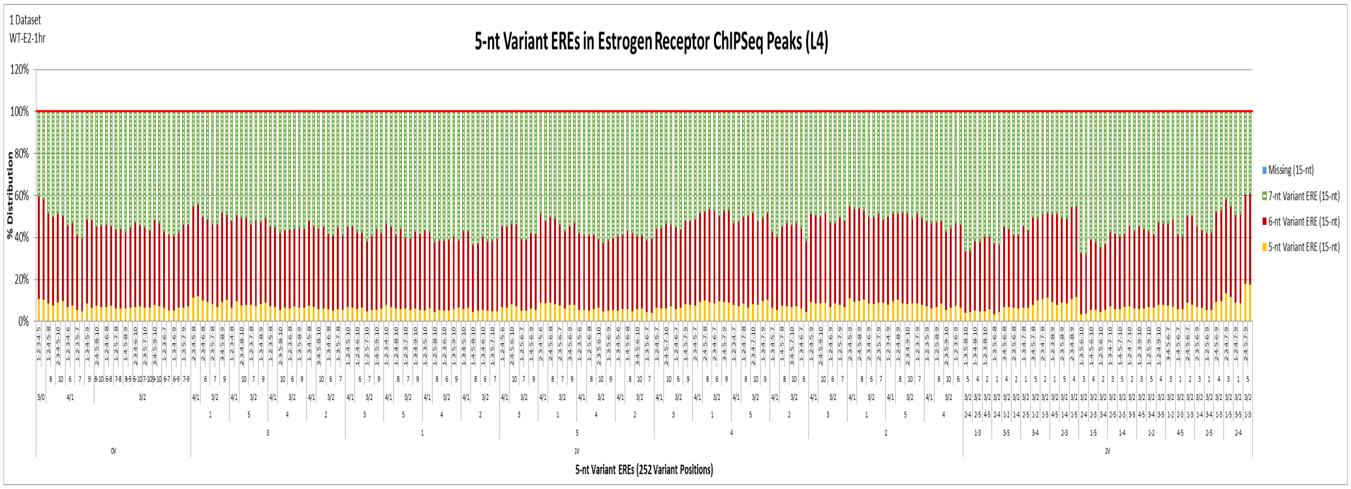

Figure S78-S79 Descriptions: sNR DNA-Binding at 13-nt and 15-nt DNA Elements in the Genome (Time-Course)

****All detailed data and statistics associated with every figure are compiled in Data S1****

Figure S78. (S/N) analysis of 0-nt to 5-nt Variant 13-nt EREs in ER (Time-Course) ChIPSeq Peaks (displayed by 252 half-site groups)

(S/N) analysis of 0-nt to 5-nt variant EREs (displayed by the 252 half-site groups) in ER (MCF7-ERa-E2-2min) [8,476 peaks, 157-nt peak length] (MCF7-ERa-E2-5min) [8,967 peaks, 157-nt peak length] (MCF7-ERa-E2-10min-A) [13,125 peaks, 163-nt peak length] (MCF7-ERa-E2-10min-B) [15,386 peaks, 168-nt peak length] (MCF7-ERa-E2-40min-A) [11,408 peaks, 176-nt peak length] (MCF7-ERa-E2-40min-B) [7,855 peaks, 208-nt peak length] (MCF7-ERa-E2-160min) [3,159 peaks, 164-nt peak length] ChIPSeq peaks. X-axis order =Reverse-Complement Vacancy Position ID 3-8 > 1-10 > 5-6 > 4-7 > 2-9. See **Table S5** for x-axis details.

Figure S79. (S/N) analysis of 0-nt to 6-nt Variant 15-nt EREs in ER (Time-Course) ChIPSeq Peaks (displayed by 924 half-site groups)

(S/N) analysis of 0-nt to 6-nt variant EREs (displayed by the 924 half-site groups) in ER (MCF7-ERa-E2-2min) [8,476 peaks, 157-nt peak length] (MCF7-ERa-E2-5min) [8,967 peaks, 157-nt peak length] (MCF7-ERa-E2-10min-A) [13,125 peaks, 163-nt peak length] (MCF7-ERa-E2-10min-B) [15,386 peaks, 168-nt peak length] (MCF7-ERa-E2-40min-A) [11,408 peaks, 176-nt peak length] (MCF7-ERa-E2-40min-B) [7,855 peaks, 208-nt peak length] (MCF7-ERa-E2-160min) [3,159 peaks, 164-nt peak length] ChIPSeq peaks. X-axis order =Reverse-Complement Vacancy Position ID 4-9 > 1-12 > 2-11 > 6-7 > 5-8 > 3-10. See **Table S25** for x-axis details.

Figure S78. (S/N) analysis of 0-nt to 5-nt Variant EREs in ER (Time-Course) ChIPSeq Peaks (252 Half-Site Groups)

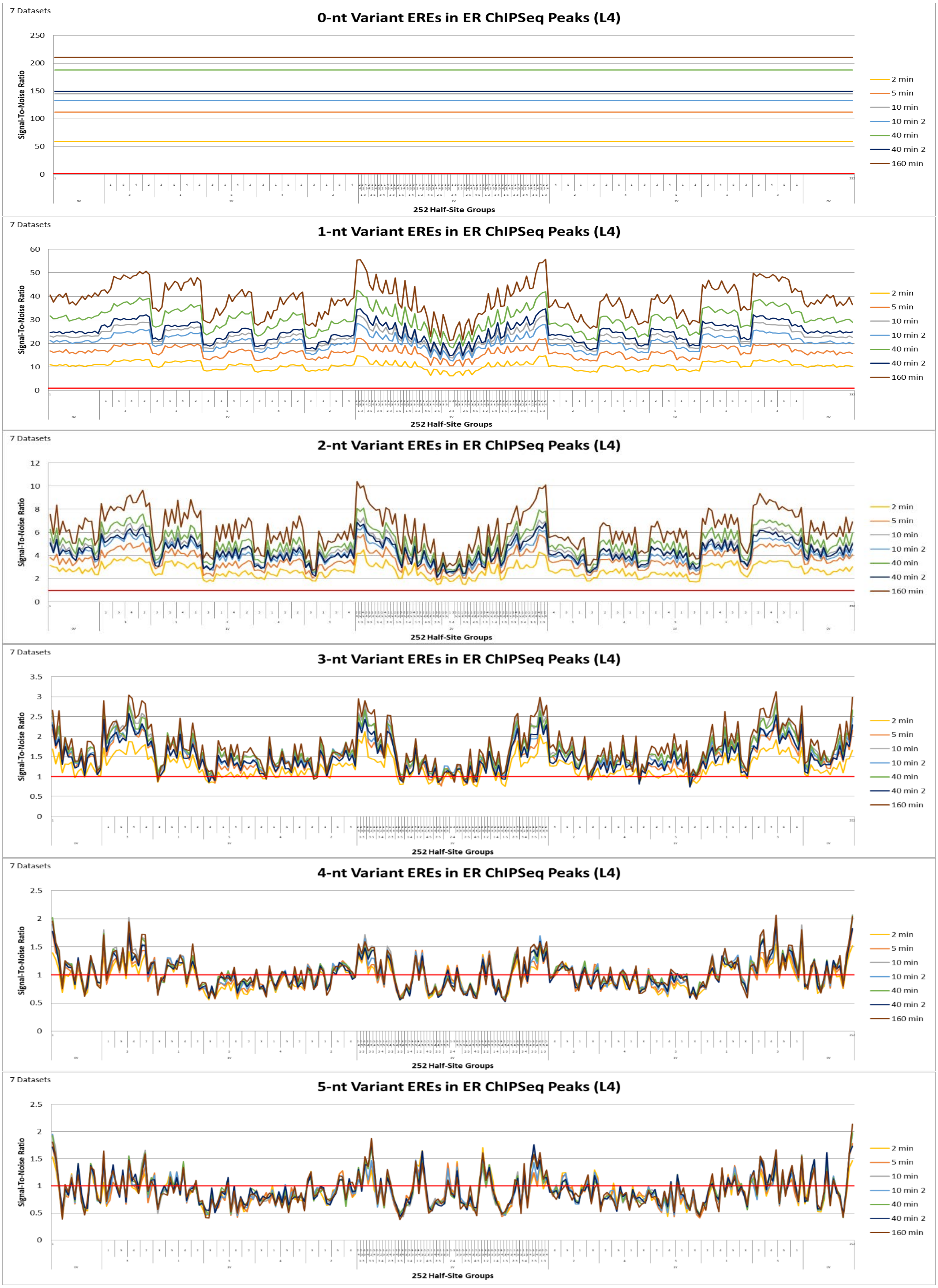

Figure S79. (S/N) analysis of 0-nt to 6-nt Variant EREs in ER (Time-Course) ChIPSeq Peaks (924 Half-Site Groups)

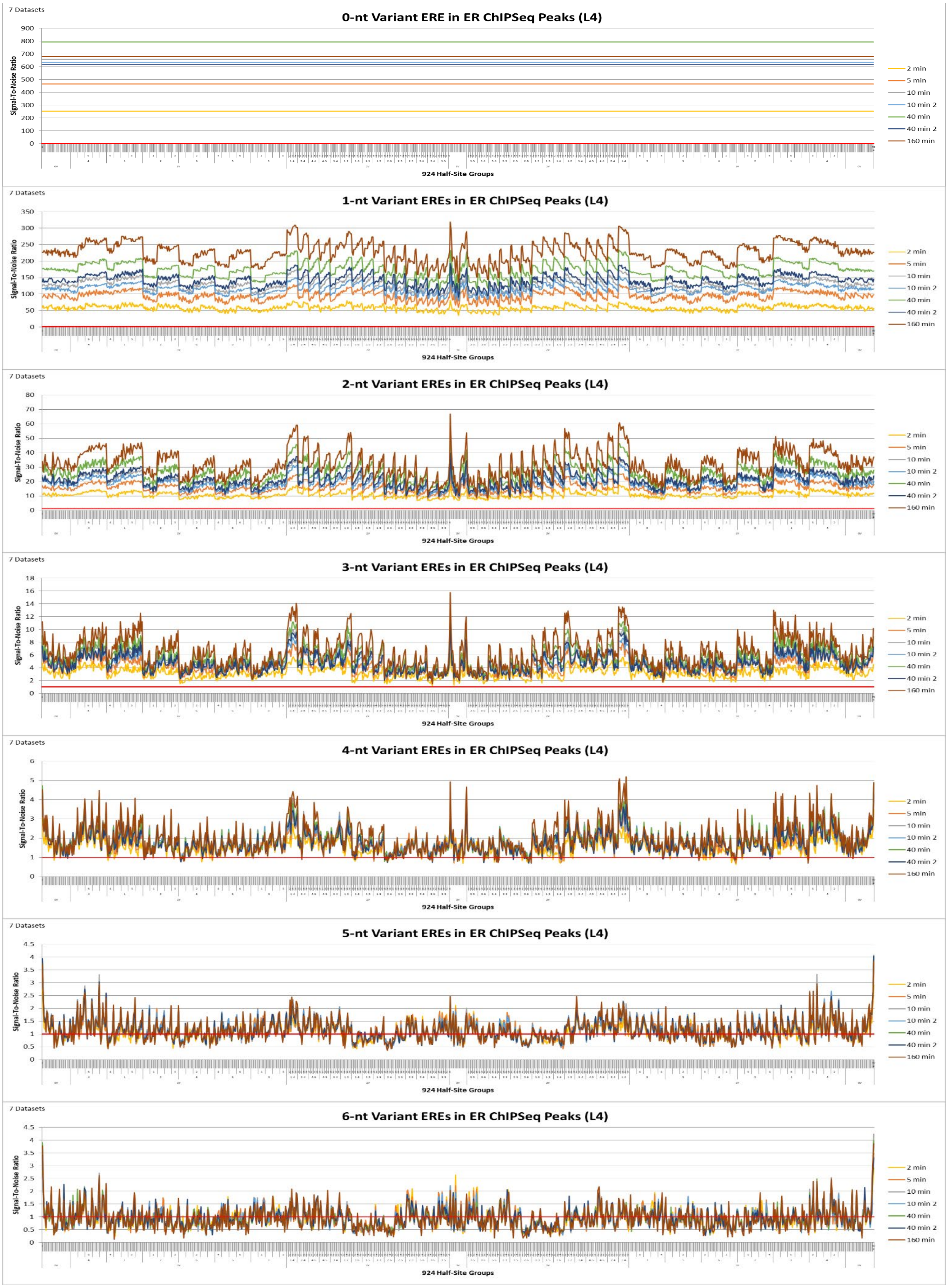

Figure S80-S81 Descriptions: p53 DNA Element Analysis

****All detailed data and statistics associated with every figure are compiled in Data S1****

To further illustrate that the structural mechanisms (by which inversion symmetry ascribes TF DNA-binding and functionality) are universally applicable, we repeated the same DNA-binding analysis as was done with the six (6) sNRs (at ERE and HRE DNA elements) on the unrelated TF p53 (at p53RE DNA elements) in the genome. The tumor suppressor p53 is a TF that controls cellular stress responses (3). Once activated, p53 binds DNA and regulates gene expression programs that contribute to apoptosis, senescence or cell cycle arrest, preventing the dissemination of damaged cells (3). These processes are involved in tumor suppression, setting the selective pressure for p53 inactivation in tumors (3).

Figure S80. Number of 0-nt to 5-nt Variant p53RE DNA Elements in the Mouse Genome
The number of 0-nt to 5-nt variants of the 10-nt p53RE consensus palindromic DNA element on the positive/sense strand in the mouse (mm10) genome. Here the 10-nt p53RE is the p53 response element (5′-TGCCCGGGCA-3′). The 0-nt to 5-nt variant p53RE DNA elements include the 1 0-nt variant consensus palindromic DNA element, 30 1-nt variant DNA elements (10 variant positions), 405 2-nt variant DNA elements (45 variant positions), 3,240 3-nt variant DNA elements (120 variant positions), 17,010 4-nt variant DNA elements (210 variant positions), and 61,236 5-nt variant DNA elements (252 variant positions), for a total of 81,922 DNA elements. The population count of each of the 81,922 0-nt to 5-nt variant 10-nt p53RE DNA elements in the mouse (mm10) genome can be found in **Table S63**.

Figure S81. (S/N) analysis of 0-nt to 5-nt Variant p53REs in p53 ChIPSeq Peaks (displayed by 252 half-site groups)
(S/N) analysis of 0-nt to 5-nt variant p53REs (displayed by the 252 half-site groups) in p53 (p53-T5-3hrs-DMSO) [4,489 peaks, 307-nt peak length] ChIPSeq peaks. X-axis order =Reverse-Complement Vacancy Position ID 5-6 > 1-10 > 3-8 > 4-7 > 2-9. See **Table S53** for x-axis details. This DNA-binding profile was observed in 22 p53 experiments, and across multiple peak selection criteria (L4-L20) **(Table S54)**.

Figure S80. Number of 0-nt to 5-nt Variant p53RE DNA Elements in the Mouse Genome

p53RE = TGCCCGGGCA

1 2 3 4 5 6 7 8 9 10

	DNA Element	Combinatorial Counts				DNA Element Frequency in Genome	
**A**	**B**	**C**	**D**	**E**	**F**	**Mouse Genome (mm10)**	
k	**P53RE** (***n*=10)**	**Combinations** $C = \binom{n}{k}$	**4-nts** $D = C \times 4^k$	**Unique** $E = C \times 3^k$	**Total Unique** $F = \Sigma E$	**Unique**	**Total Unique**
**0**	0-nt Variant p53RE	1	1	1	1	1,078	
**1**	1-nt Variant p53RE	10	40	30	31	33,992	35,070
**2**	2-nt Variant p53RE	45	720	405	436	641,422	676,492
**3**	3-nt Variant p53RE	120	7,680	3,240	3,676	5,380,457	6,056,949
**4**	4-nt Variant p53RE	210	53,760	17,010	20,686	29,257,379	35,314,328
**5**	5-nt Variant p53RE	252	258,048	61,236	81,922	113,933,936	149,248,264

**A**	**B**	**C**	**D**	**E**	**F**
k		**Combinations** $C = \binom{n}{k}$	**4-nts** $D = C \times 4^k$	**Unique** $E = C \times 3^k$	**Total Unique** $F = \Sigma E$
**0**	0-nt Variant	10!/(0! x 10!)	1 x 1	1 x 1	1 + 0
**1**	1-nt Variant	10!/(1! x 9!)	10 x 4	10 x 3	30 + 1
**2**	2-nt Variant	10!/(2! x 8!)	45 x 16	45 x 9	405 + 31
**3**	3-nt Variant	10!/(3! x 7!)	120 x 64	120 x 27	3,240 + 436
**4**	4-nt Variant	10!/(4! x 6!)	210 x 256	210 x 81	17,010 +3,676
**5**	5-nt Variant	10!/(5! x 5!)	252 x 1,024	252 x 243	61,236 + 20,686

Figure S81. (S/N) analysis of 0-nt to 5-nt Variant p53REs in p53 ChIPSeq Peaks (252 Half-Site Groups)

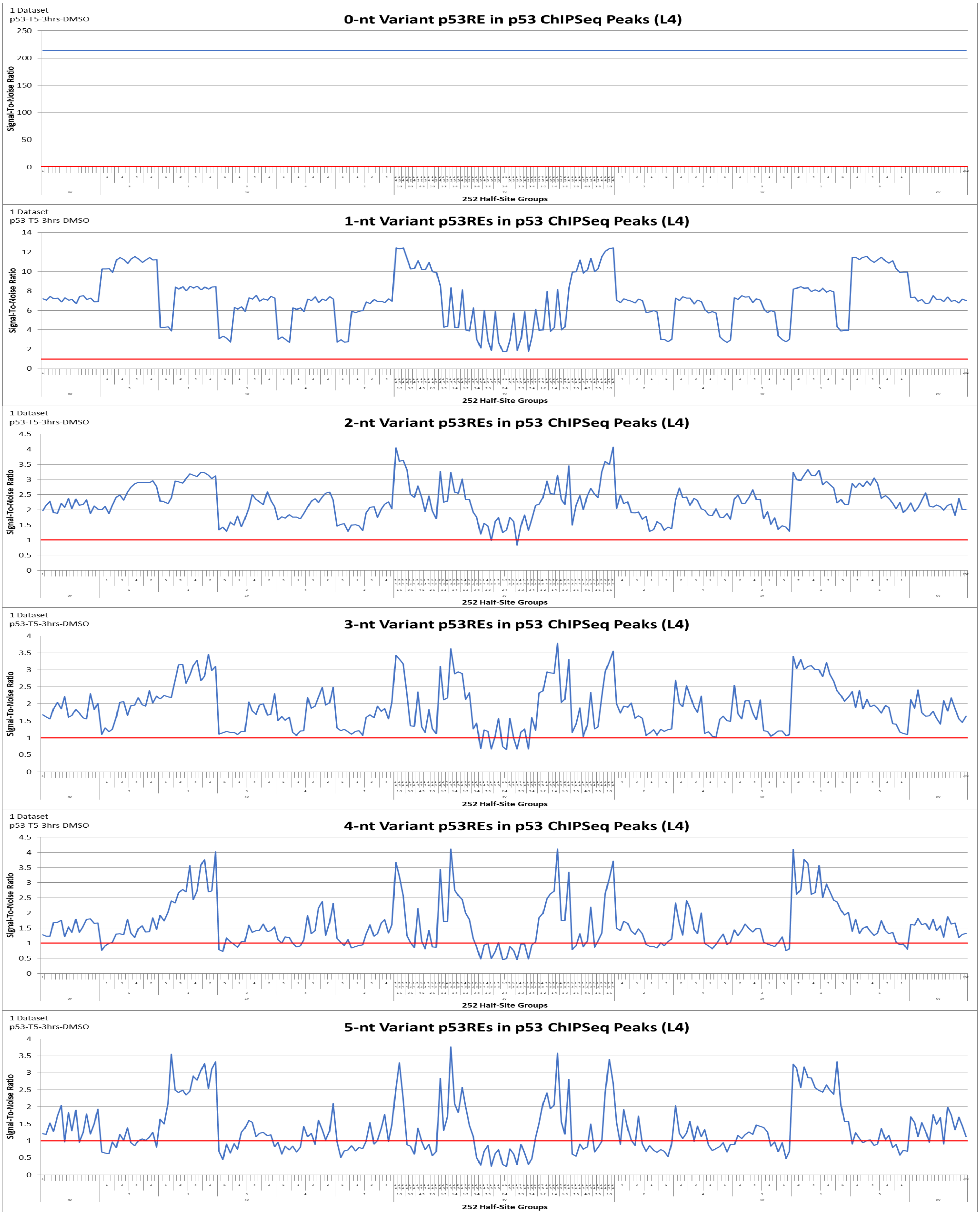

Figure S82-S91 Descriptions: sNR DNA-Binding at 13-nt and 15-nt DNA Elements in the Genome (AR and AR-SPARKI)

****All detailed data and statistics associated with every figure are compiled in Data S1****

It's perplexing that the inversion symmetry that underlies TF DNA-binding specificity and functionality in the genome, observed in hundreds of ChIPSeq and ChIPExo experiments, has not been detected using current DNA motif identification algorithms. Our analysis reveals that the resolution capability of PWMs-based DNA motif identification is limited. An overreliance on these PWMs-based algorithms has promoted the misconception that the majority of TF DNA-binding events in the genome are driven by mechanisms other than the DNA sequence. This overreliance has also led to the misconception that sNRs can indiscriminately bind to the *cis*-regulatory DNA element of other nuclear receptors. Furthermore, different *cis*-regulatory DNA elements have been delineated for the different members of the KR family.

Analysis of KR DNA-binding at 81,922 0-nt to 5-nt variant 13-nt HRE DNA elements and at 912,718 0-nt to 6-nt variant 15-nt HRE DNA elements in the genome (in 194 KR ChIPSeq and ChIPExo experiments, representing a wide variety of mouse tissues and human cell lines, and across multiple peak selection criteria (L4-L20)) quantitively demonstrates that all members of the KR family are following the same DNA-binding rules, and thus bind the same DNA elements. This is further illustrated by the equivalent structure of the DNA-binding curves observed in AR and an AR DBD mouse model (SPARKI), where the second zinc finger in the AR DBD is replaced by that of the GR, at 81,922 0-nt to 5-nt variant 13-nt HRE DNA elements and at 912,718 0-nt to 6-nt variant 15-nt HRE DNA elements in the genome. Two biological replicates (#1 and #2) from intact AR wild-type and AR-SPARKI mice were sequenced and used in peak calling (4). To increase the depth of the analysis, replicate ChIPSeq samples were merged (#M) and the concatenated samples were used in peak calling (4).

Figure S82. (S/N) analysis of 0-nt to 5-nt Variant 13-nt HREs in AR and AR-SPARKI ChIPSeq Peaks #1 (displayed by 252 half-site groups)
(S/N) analysis of <u>0-nt to 5-nt variant HREs</u> (displayed by the 252 half-site groups) in AR (AR-wt1) [49,859 peaks, 136-nt peak length] ChIPSeq peaks and AR-SPARKI (AR-SPARKI1) [41,315 peaks, 141-nt peak length] ChIPSeq peaks. X-axis order =Reverse-Complement Vacancy Position ID 2-9 > 5-6 > 1-10 > 4-7 > 3-8. See **Table S6** for x-axis details.

Figure S83. (S/N) analysis of 0-nt to 5-nt Variant 13-nt HREs in AR and AR-SPARKI ChIPSeq Peaks #2 (displayed by 252 half-site groups)
(S/N) analysis of <u>0-nt to 5-nt variant HREs</u> (displayed by the 252 half-site groups) in AR (AR-wt2) [47,112 peaks, 142-nt peak length] ChIPSeq peaks and AR-SPARKI (AR-SPARKI2) [21,735 peaks, 130-nt peak length] ChIPSeq peaks. X-axis order =Reverse-Complement Vacancy Position ID 2-9 > 5-6 > 1-10 > 4-7 > 3-8. See **Table S6** for x-axis details.

Figure S84. (S/N) analysis of 0-nt to 5-nt Variant 13-nt HREs in AR and AR-SPARKI ChIPSeq Peaks #M (displayed by 252 half-site groups)
(S/N) analysis of <u>0-nt to 5-nt variant HREs</u> (displayed by the 252 half-site groups) in AR (Pro-AR-wt1) [40,737 peaks, 147-nt peak length] ChIPSeq peaks and AR-SPARKI (Pro-AR-SPARKI1) [35,846 peaks, 147-nt peak length] ChIPSeq peaks. X-axis order =Reverse-Complement Vacancy Position ID 2-9 > 5-6 > 1-10 > 4-7 > 3-8. See **Table S6** for x-axis details.

*****

Figure S85. (S/N) analysis of 0-nt to 6-nt Variant 15-nt HREs in AR and AR-SPARKI ChIPSeq Peaks #1 (displayed by 252 half-site groups)
(S/N) analysis of <u>0-nt to 6-nt variant HREs</u> (displayed by the 924 half-site groups) in AR (AR-wt1) [49,859 peaks, 136-nt peak length] ChIPSeq peaks and AR-SPARKI (AR-SPARKI1) [41,315 peaks, 141-nt peak length] ChIPSeq peaks. X-axis order =Reverse-Complement Vacancy Position ID 3-10 > 1-12 > 6-7 > 2-11 > 5-8 > 4-9. See **Table S26** for x-axis details.

Figure S86. (S/N) analysis of 0-nt to 6-nt Variant 15-nt HREs in AR and AR-SPARKI ChIPSeq Peaks #2 (displayed by 252 half-site groups)
(S/N) analysis of <u>0-nt to 6-nt variant HREs</u> (displayed by the 924 half-site groups) in AR (AR-wt2) [47,112 peaks, 142-nt peak length] ChIPSeq peaks and AR-SPARKI (AR-SPARKI2) [21,735 peaks, 130-nt peak length] ChIPSeq peaks. X-axis order =Reverse-Complement Vacancy Position ID 3-10 > 1-12 > 6-7 > 2-11 > 5-8 > 4-9. See **Table S26** for x-axis details.

Figure S87. (S/N) analysis of 0-nt to 6-nt Variant 15-nt HREs in AR and AR-SPARKI ChIPSeq Peaks #M (displayed by 252 half-site groups)
(S/N) analysis of <u>0-nt to 6-nt variant HREs</u> (displayed by the 924 half-site groups) in AR (Pro-AR-wt1) [40,737 peaks, 147-nt peak length] ChIPSeq peaks and AR-SPARKI (Pro-AR-SPARKI1) [35,846 peaks, 147-nt peak length] ChIPSeq peaks. X-axis order =Reverse-Complement Vacancy Position ID 3-10 > 1-12 > 6-7 > 2-11 > 5-8 > 4-9. See **Table S26** for x-axis details.

*****

Figure S88. (S/N) analysis of 1-nt to 5-nt Variant 13-nt HREs in AR and AR-SPARKI ChIPSeq Peaks #1 #2 (displayed by variant position)
(S/N) analysis of 1-nt to 5-nt variant HREs (displayed by variant position) in AR (AR-wt1 and -2) [49,859 peaks, 136-nt peak length] [47,112 peaks, 142-nt peak length] ChIPSeq peaks and AR-SPARKI (AR-SPARKI1 and -2) [41,315 peaks, 141-nt peak length] [21,735 peaks, 130-nt peak length] ChIPSeq peaks.

Figure S89. (S/N) analysis of 1-nt to 5-nt Variant 13-nt HREs in AR and AR-SPARKI ChIPSeq Peaks #M (displayed by variant position)
(S/N) analysis of 1-nt to 5-nt variant HREs (displayed by variant position) in AR (Pro-AR-wt1) [40,737 peaks, 147-nt peak length] ChIPSeq peaks and AR-SPARKI (Pro-AR-SPARKI1) [35,846 peaks, 147-nt peak length] ChIPSeq peaks.

*****

Figure S90. (S/N) analysis of 1-nt to 6-nt Variant 15-nt HREs in AR and AR-SPARKI ChIPSeq Peaks #1 #2 (displayed by variant position)
(S/N) analysis of 1-nt to 6-nt variant HREs (displayed by variant position) in AR (AR-wt1 and -2) [49,859 peaks, 136-nt peak length] [47,112 peaks, 142-nt peak length] ChIPSeq peaks and AR-SPARKI (AR-SPARKI1 and -2) [41,315 peaks, 141-nt peak length] [21,735 peaks, 130-nt peak length] ChIPSeq peaks.

Figure S91. (S/N) analysis of 1-nt to 6-nt Variant 15-nt HREs in AR and AR-SPARKI ChIPSeq Peaks #M (displayed by variant position)
(S/N) analysis of 1-nt to 6-nt variant HREs (displayed by variant position) in AR (Pro-AR-wt1) [40,737 peaks, 147-nt peak length] ChIPSeq peaks and AR-SPARKI (Pro-AR-SPARKI1) [35,846 peaks, 147-nt peak length] ChIPSeq peaks.

Figure S82. (S/N) analysis of 0-nt to 5-nt Variant HREs in AR and AR-SPARKI ChIPSeq Peaks #1 (252 Half-Site Groups)

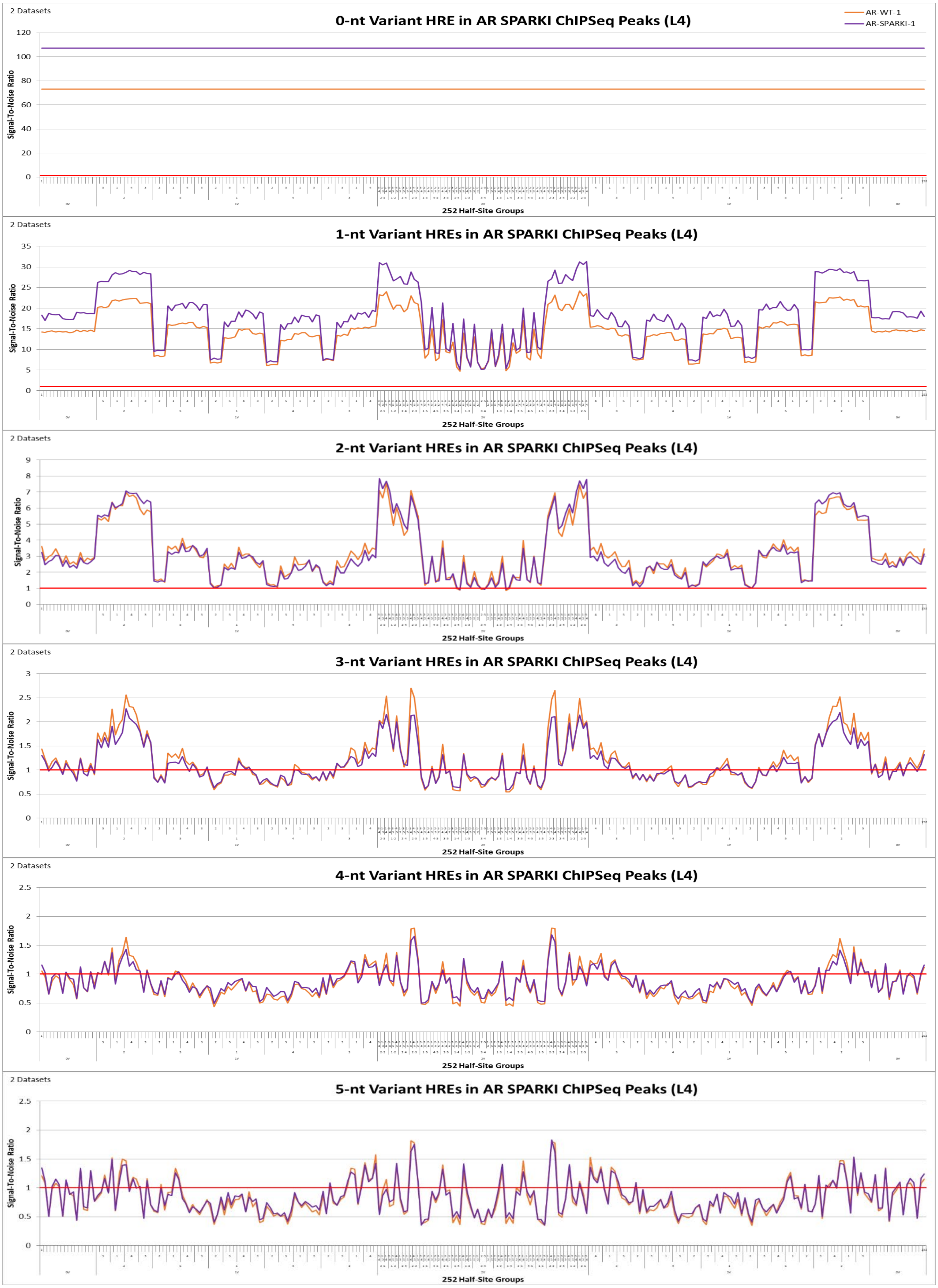

Figure S83. (S/N) analysis of 0-nt to 5-nt Variant HREs in AR and AR-SPARKI ChIPSeq Peaks #2 (252 Half-Site Groups)

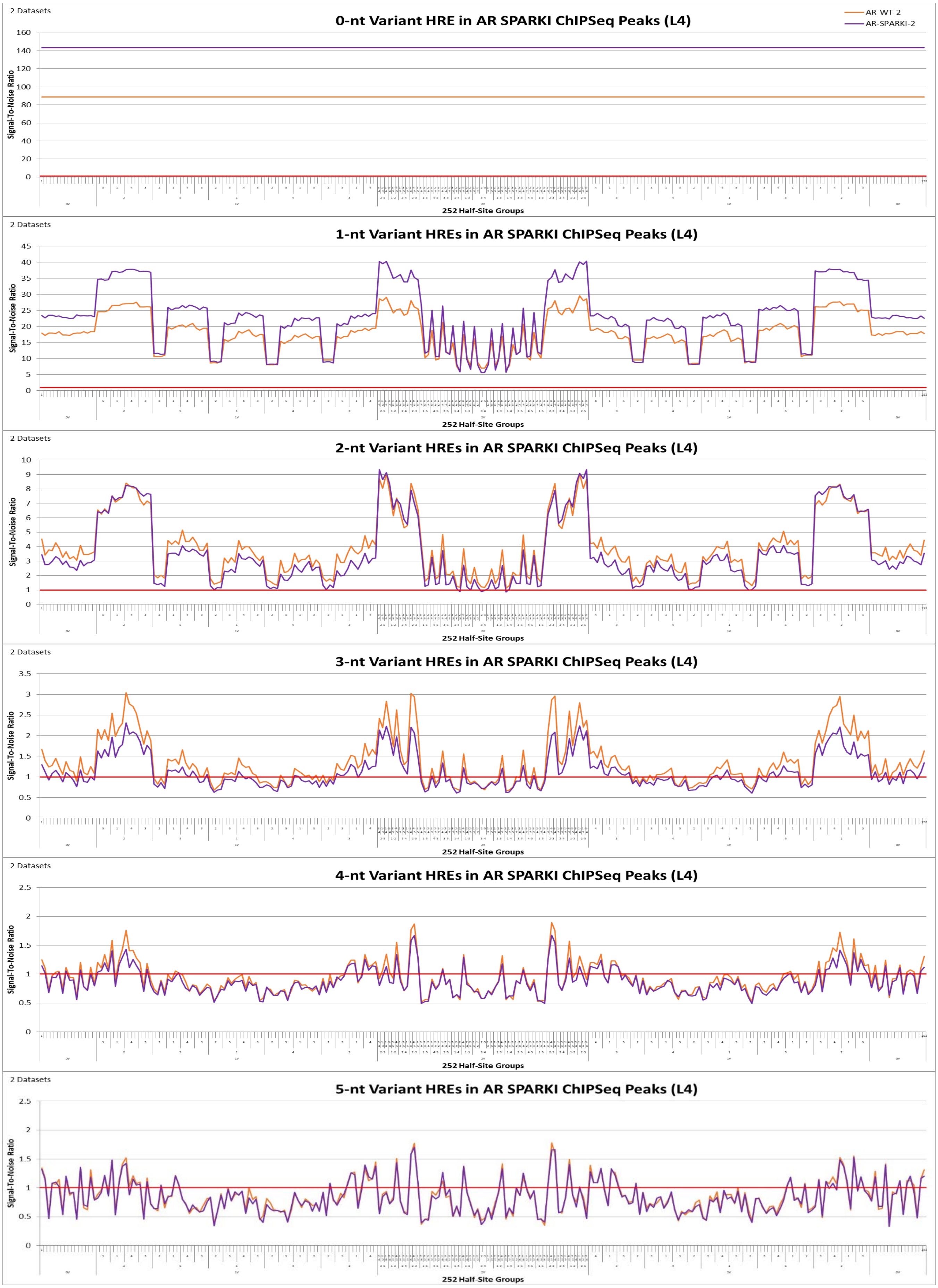

Figure S84. (S/N) analysis of 0-nt to 5-nt Variant HREs in AR and AR-SPARKI ChIPSeq Peaks #M (252 Half-Site Groups)

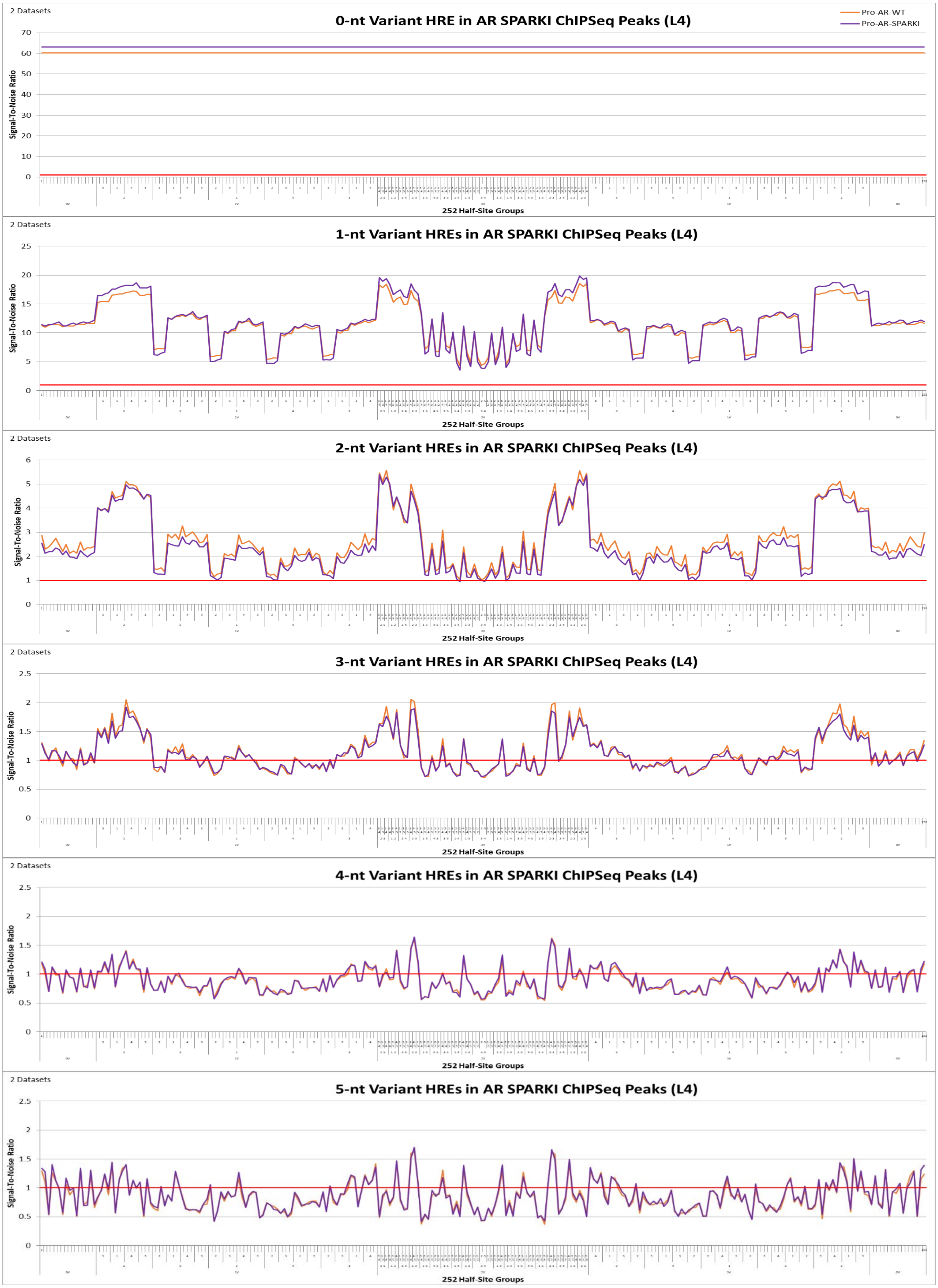

Figure S85. (S/N) analysis of 0-nt to 6-nt Variant HREs in AR and AR-SPARKI ChIPSeq Peaks #1 (924 Half-Site Groups)

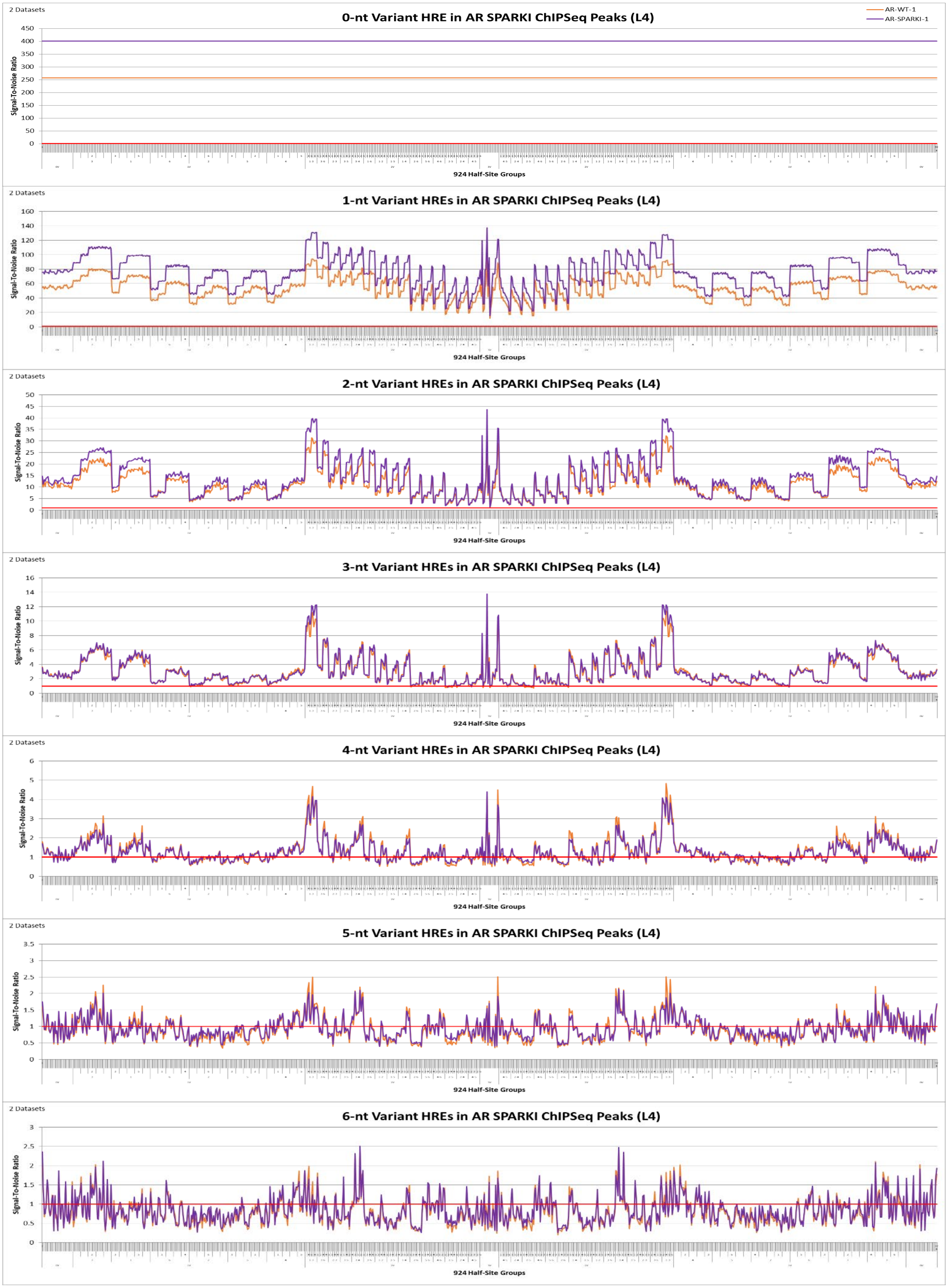

Figure S86. (S/N) analysis of 0-nt to 6-nt Variant HREs in AR and AR-SPARKI ChIPSeq Peaks #2 (924 Half-Site Groups)

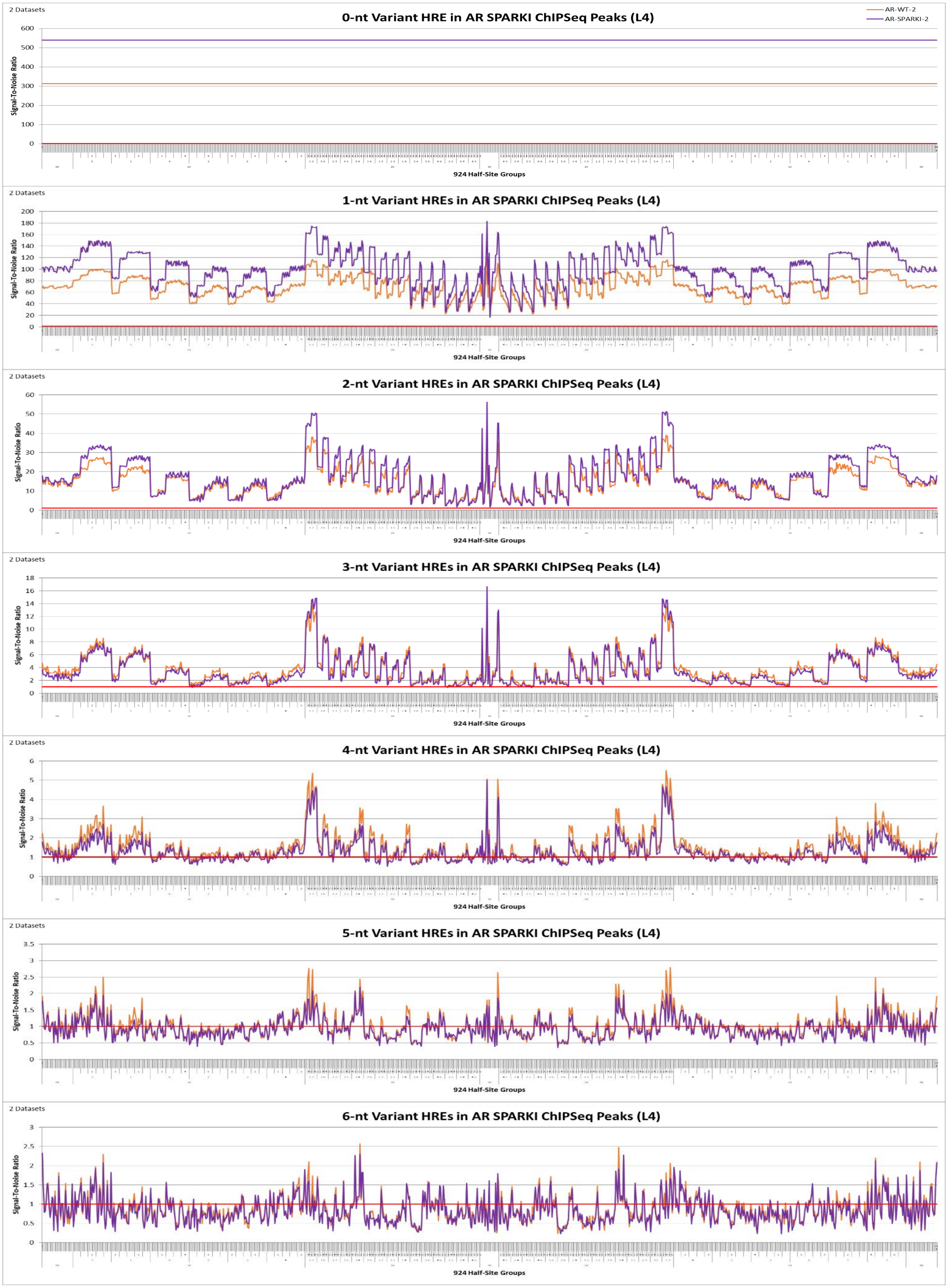

Figure S87. (S/N) analysis of 0-nt to 6-nt Variant HREs in AR and AR-SPARKI ChIPSeq Peaks #M (924 Half-Site Groups)

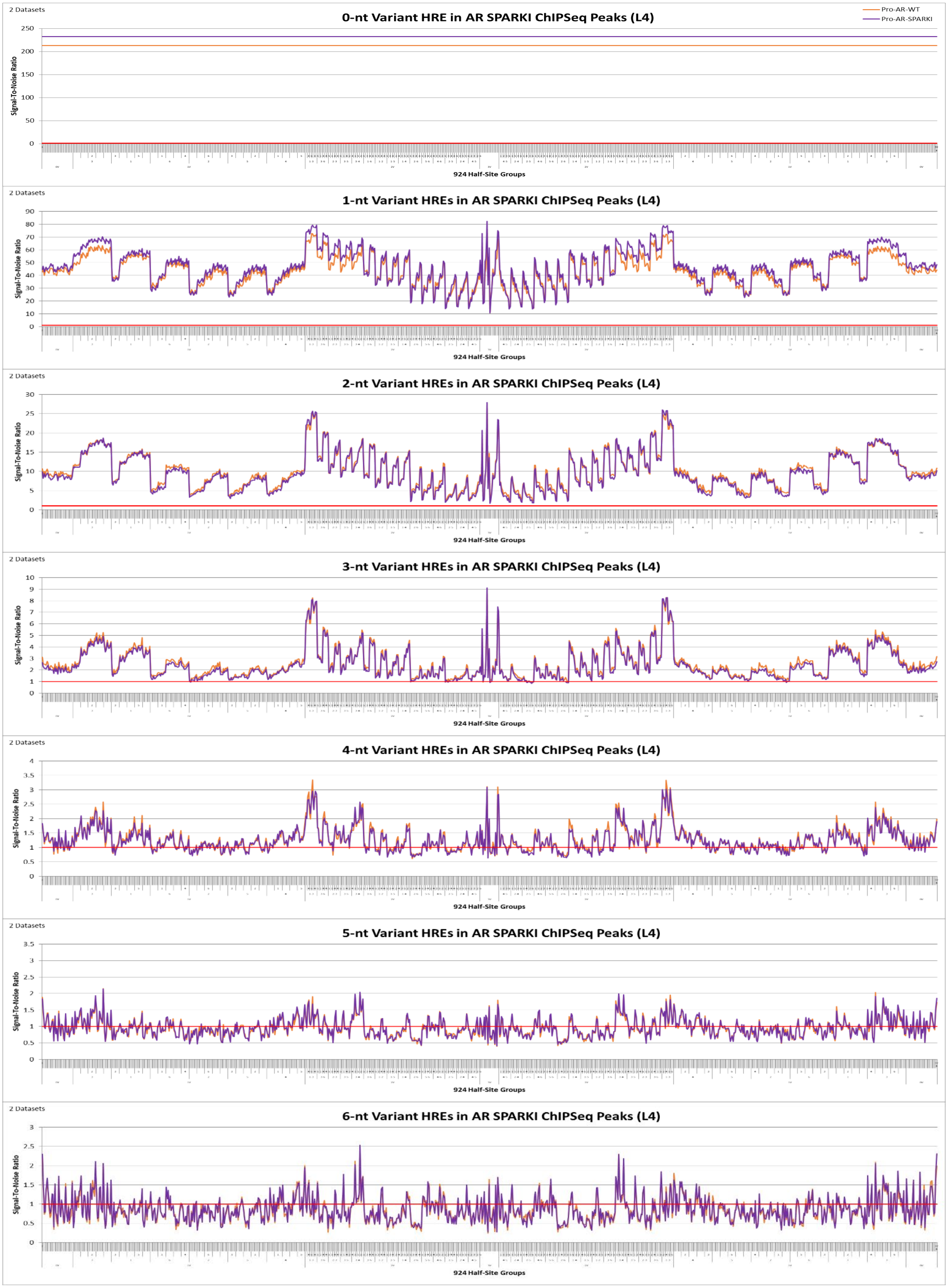

Figure S88. (S/N) analysis of 1-nt to 5-nt Variant HREs in AR and AR-SPARKI ChIPSeq Peaks #1 & #2 (Variant Position)

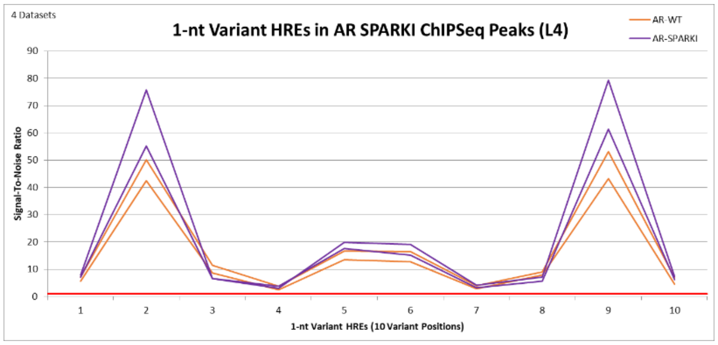

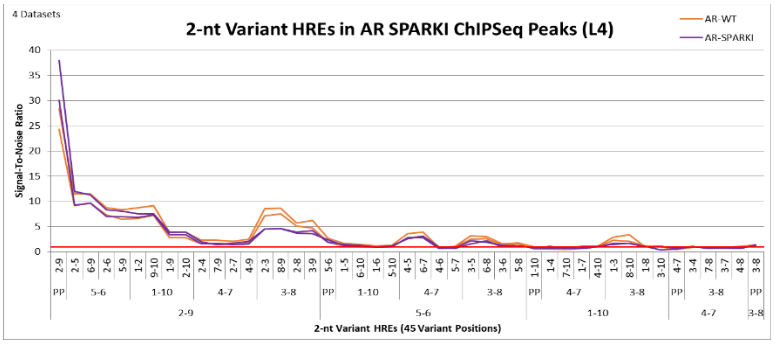

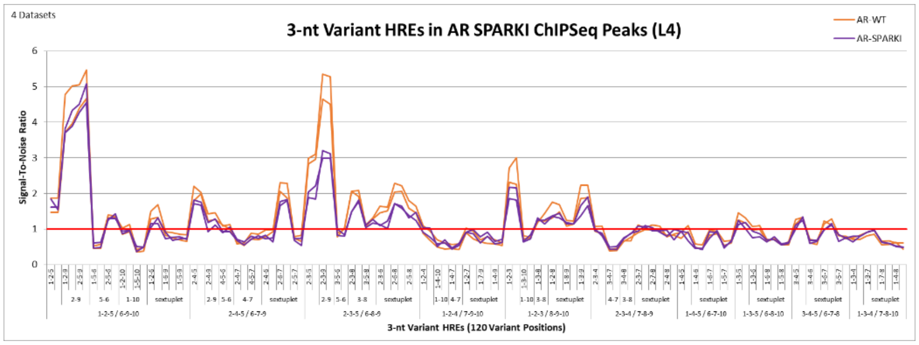

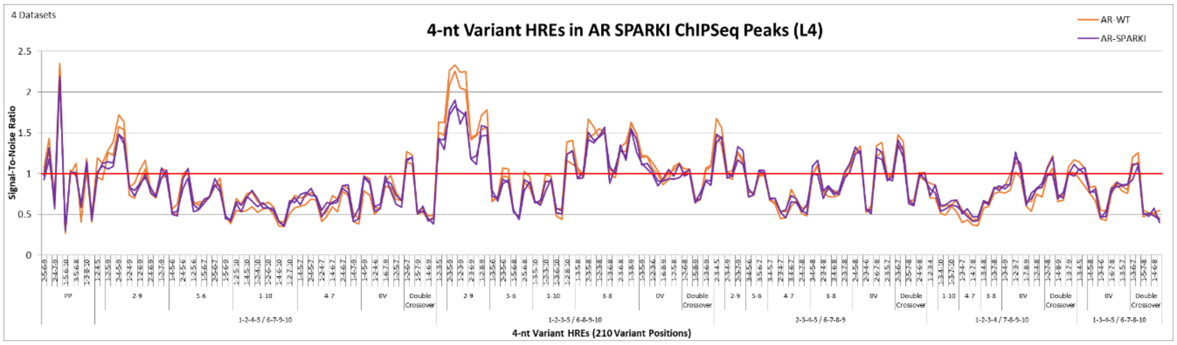

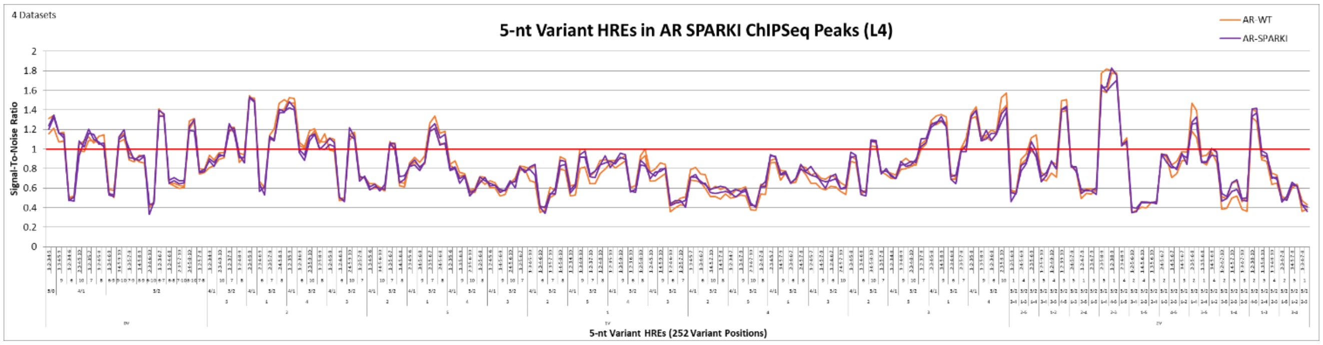

Figure S89. (S/N) analysis of 1-nt to 5-nt Variant HREs in AR and AR-SPARKI ChIPSeq Peaks #M (Variant Position)

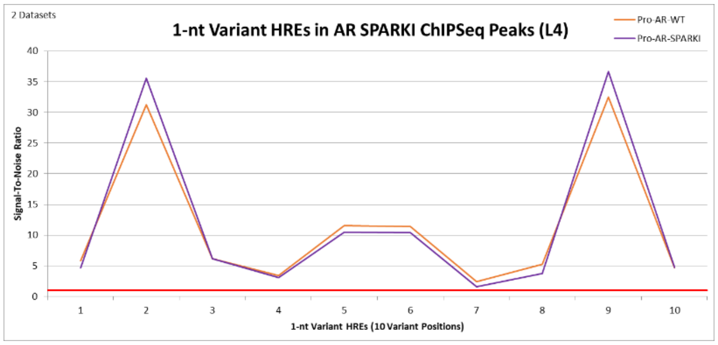

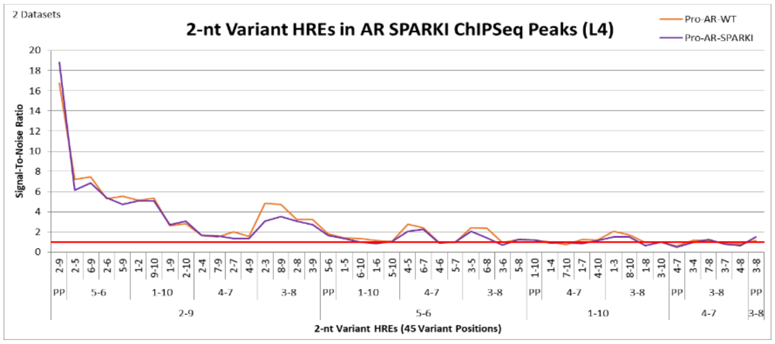

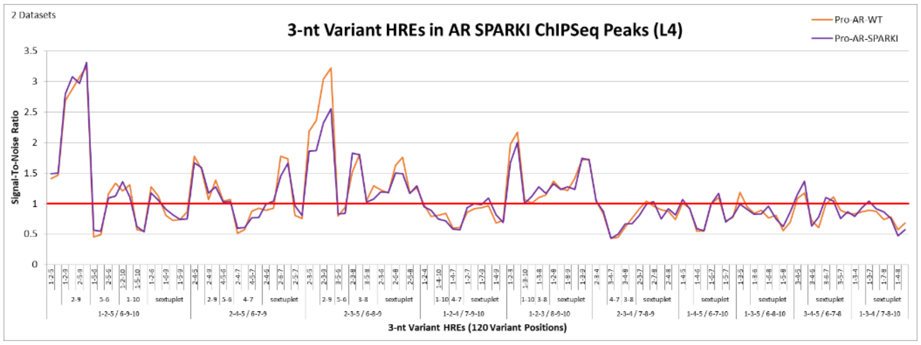

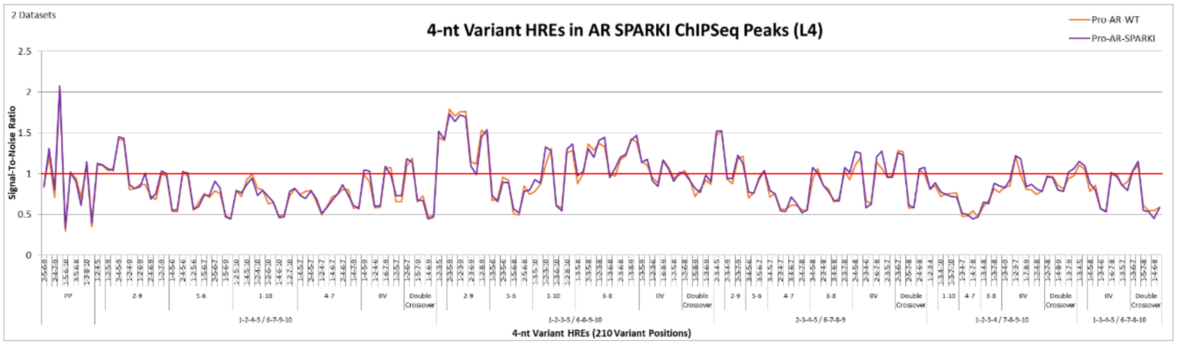

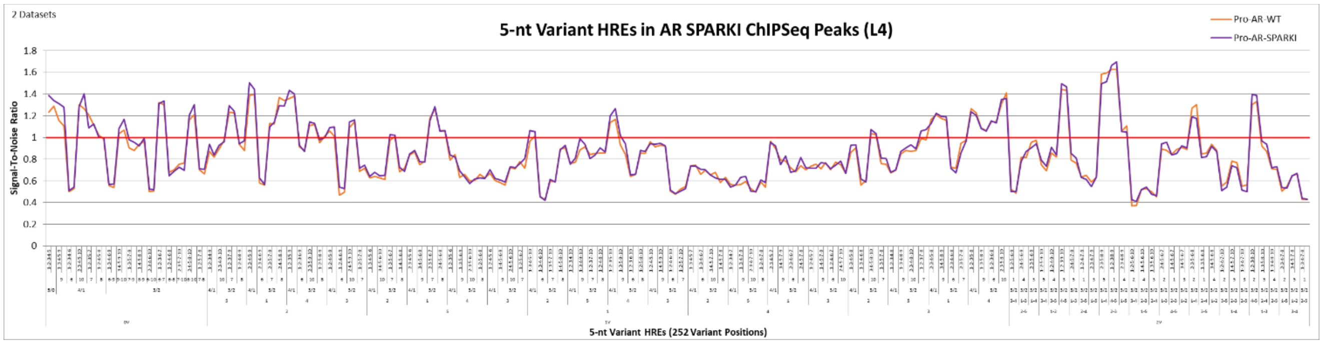

Figure S90. (S/N) analysis of 1-nt to 6-nt Variant HREs in AR and AR-SPARKI ChIPSeq Peaks #1 & #2 (Variant Position)

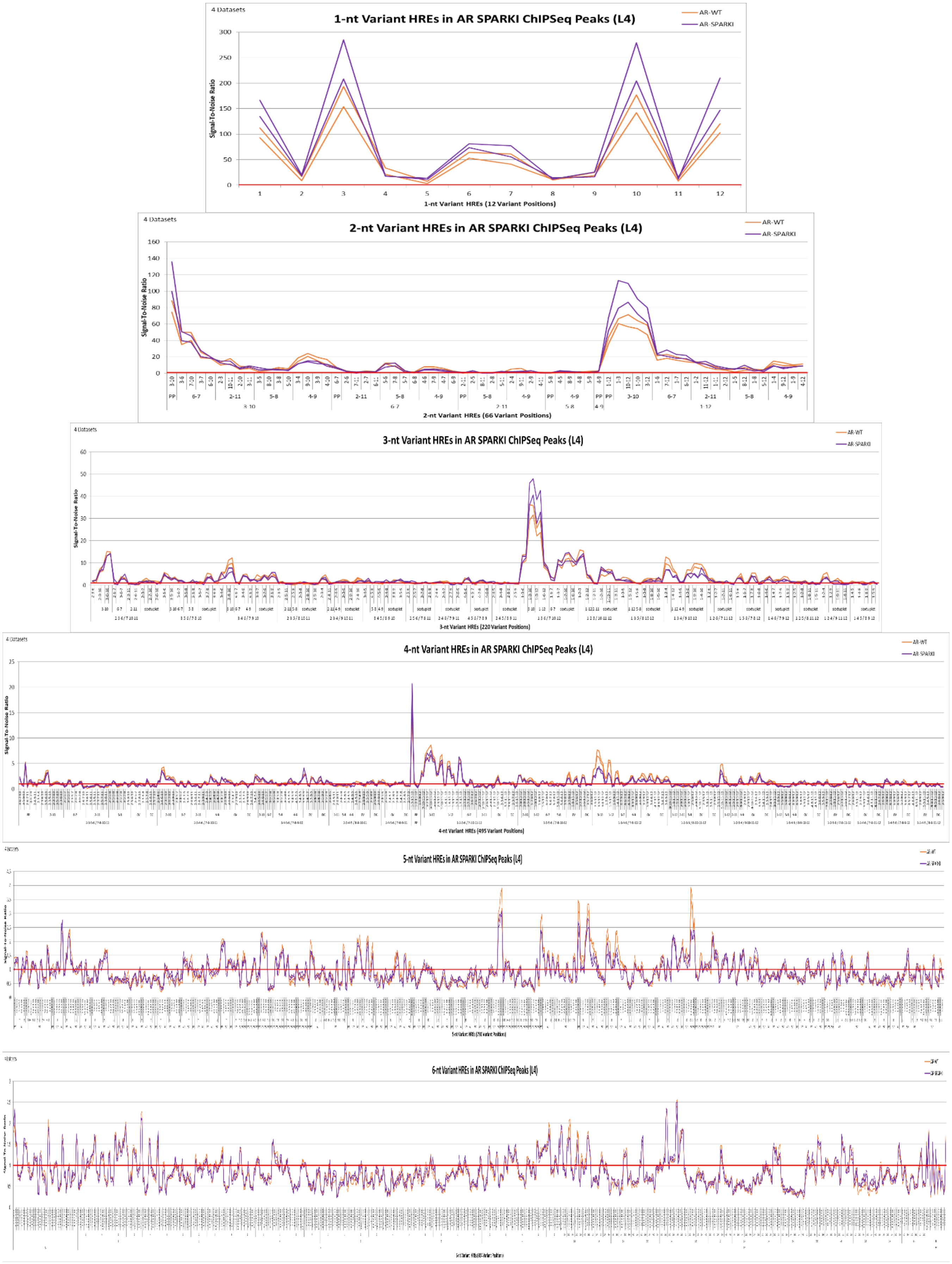

Figure S91. (S/N) analysis of 1-nt to 6-nt Variant HREs in AR and AR-SPARKI ChIPSeq Peaks #M (Variant Position)

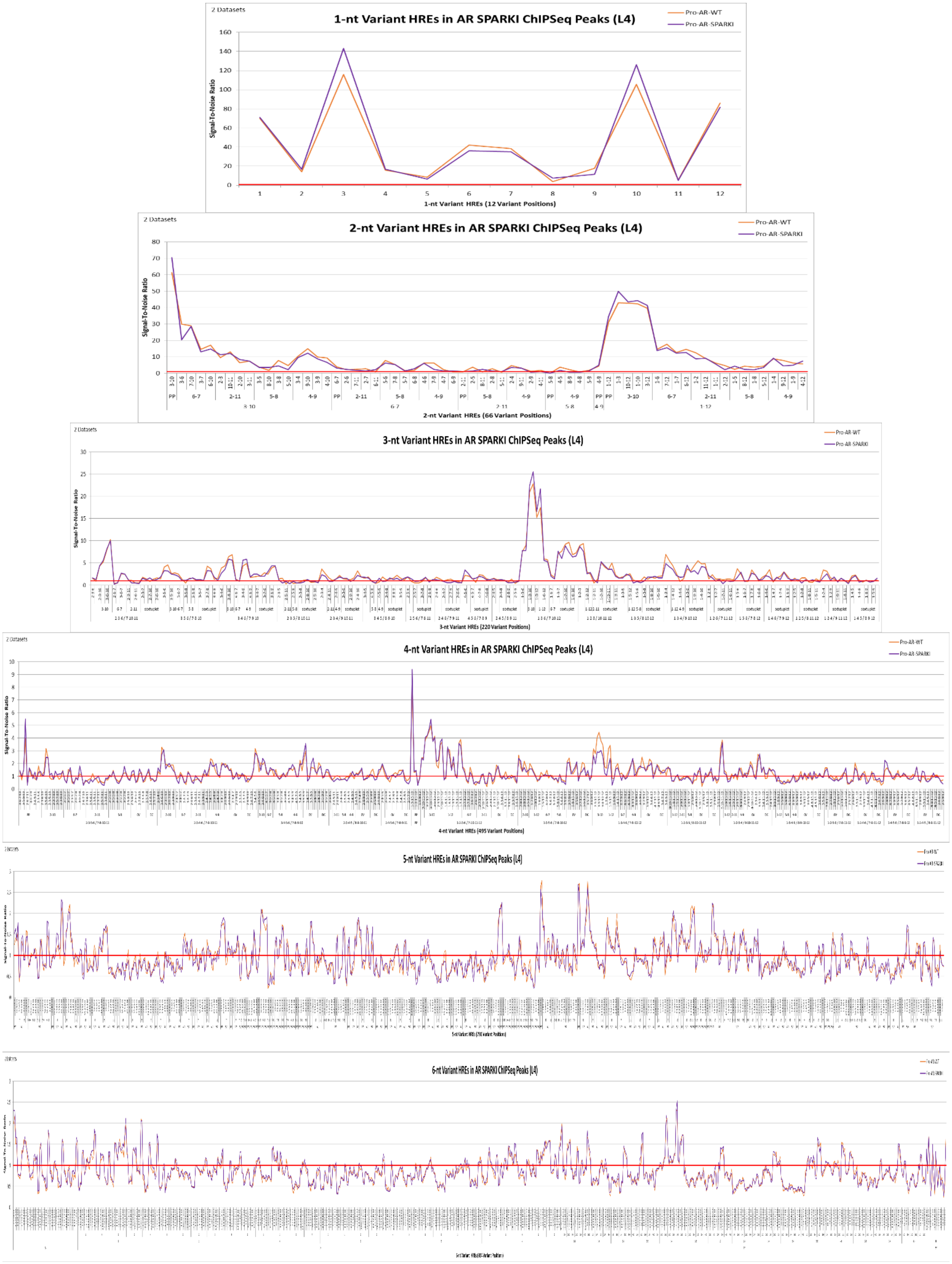

Data S1. All Detailed Data and Statistics Associated with Every Figure

- Figure 1 – Figure 5
- Table 3
- Table 5
- Figure S1 – Figure S91
- Upper and lower one-tailed Poisson significance thresholds at $p<0.001$, thus the probability of the sNR DNA-binding signal occurring outside these boundaries by chance is less than one in a thousand

Table S1-S75 Descriptions

Table S1-S20. 13-nt ERE and HRE DNA Element Analysis

The analyses in this section were completed by overlapping the location coordinates of each 0-nt to 5-nt variant 13-nt ERE or HRE DNA element in the genome and the location coordinates of the ChIPSeq or ChIPExo peaks in an experiment. The 0-nt variant consensus palindromic 13-nt ERE DNA element (5′-GGTCAnnnTGACC-3′) and 0-nt variant consensus palindromic 13-nt HRE DNA element (5′-GAACAnnnTGTTC-3′) have ten (10) primary positions. The 0-nt to 5-nt variant ERE or HRE DNA elements include the 1 0-nt variant consensus palindromic DNA element, 30 1-nt variant DNA elements (10 variant positions), 405 2-nt variant DNA elements (45 variant positions), 3,240 3-nt variant DNA elements (120 variant positions), 17,010 4-nt variant DNA elements (210 variant positions), and 61,236 5-nt variant DNA elements (252 variant positions), for a total of 81,922 DNA elements.

Table S1. Categorize 0-nt to 5-nt Variant 13-nt EREs into 252 Half-Site Groups (Variant Position)

- Categorize the 81,922 0-nt to 5-nt variant 13-nt ERE DNA elements into 252 half-site groups (5 positions are fixed, allowing for *up to* 5 positions to be varied)
- Each of the 252 ERE half-site groups contain: 1 0-nt variant consensus palindromic ERE DNA element, 15 1-nt variant ERE DNA elements, 90 2-nt variant ERE DNA elements, 270 3-nt variant ERE DNA elements, 405 4-nt variant ERE DNA elements, 243 5-nt variant ERE DNA elements, for a total of 1,024 ERE DNA elements
- 252 half-site groups x 1,024 DNA elements per half-site group =258,048 DNA elements (81,922 unique)

Table S2. Categorize 0-nt to 5-nt Variant 13-nt HREs into 252 Half-Site Groups (Variant Position)

- Categorize the 81,922 0-nt to 5-nt variant 13-nt HRE DNA elements into 252 half-site groups (5 positions are fixed, allowing for *up to* 5 positions to be varied)
- Each of the 252 HRE half-site groups contain: 1 0-nt variant consensus palindromic HRE DNA element, 15 1-nt variant HRE DNA elements, 90 2-nt variant HRE DNA elements, 270 3-nt variant HRE DNA elements, 405 4-nt variant HRE DNA elements, 243 5-nt variant HRE DNA elements, for a total of 1,024 HRE DNA elements
- 252 half-site groups x 1,024 DNA elements per half-site group =258,048 DNA elements (81,922 unique)

Table S3. Categorize 0-nt to 5-nt Variant 13-nt EREs into 252 Half-Site Groups (Sequence)

- Categorize the 81,922 0-nt to 5-nt variant 13-nt ERE DNA elements into 252 half-site groups (5 positions are fixed, allowing for *up to* 5 positions to be varied)
- Each of the 252 ERE half-site groups contain: 1 0-nt variant consensus palindromic ERE DNA element, 15 1-nt variant ERE DNA elements, 90 2-nt variant ERE DNA elements, 270 3-nt variant ERE DNA elements, 405 4-nt variant ERE DNA elements, 243 5-nt variant ERE DNA elements, for a total of 1,024 ERE DNA elements
- 252 half-site groups x 1,024 DNA elements per half-site group =258,048 DNA elements (81,922 unique)

Table S4. Categorize 0-nt to 5-nt Variant 13-nt HREs into 252 Half-Site Groups (Sequence)

- Categorize the 81,922 0-nt to 5-nt variant 13-nt HRE DNA elements into 252 half-site groups (5 positions are fixed, allowing for *up to* 5 positions to be varied)
- Each of the 252 HRE half-site groups contain: 1 0-nt variant consensus palindromic HRE DNA element, 15 1-nt variant HRE DNA elements, 90 2-nt variant HRE DNA elements, 270 3-nt variant HRE DNA elements, 405 4-nt variant HRE DNA elements, 243 5-nt variant HRE DNA elements, for a total of 1,024 HRE DNA elements
- 252 half-site groups x 1,024 DNA elements per half-site group =258,048 DNA elements (81,922 unique)

Table S5. X-Axis Order of 252 ERE Half-Site Groups

- (S/N) analysis of 0-nt to 5-nt variant EREs in ER ChIPSeq or ChIPExo peaks (displayed by 252 ERE half-site groups)
- X-axis order
  - 126 half-site groups [left-to-right: 0 vacancies, 1 vacancy, 2 vacancies] followed by their 126 reverse-complements
  - Of these 252 half-site groups, 32 have zero vacancies, 160 have one vacancy, 60 have two vacancies
  - The x-axis is labeled by the reverse-complement vacancy position ID (primary label) and reverse-complement double occupant position ID (secondary label)
  - The order of the reverse-complement vacancy position IDs for ERE is 3-8 > 1-10 > 5-6 > 4-7 > 2-9

Table S6. X-Axis Order of 252 HRE Half-Site Groups

- (S/N) analysis of 0-nt to 5-nt variant HREs in KR ChIPSeq or ChIPExo peaks (displayed by 252 HRE half-site groups)
- X-axis order
  - 126 half-site groups [left-to-right: 0 vacancies, 1 vacancy, 2 vacancies] followed by their 126 reverse-complements
  - Of these 252 half-site groups, 32 have zero vacancies, 160 have one vacancy, 60 have two vacancies

- The x-axis is labeled by the reverse-complement vacancy position ID (primary label) and reverse-complement double occupant position ID (secondary label)
- The order of the reverse-complement vacancy position IDs for HRE is 2/9 > 5/6 > 1/10 > 4/7 > 3/8

Table S7. (S/N) analysis of 0-nt to 5-nt Variant EREs in ER ChIPSeq Peaks (252 Half-Site Groups) [157]

- (S/N) analysis of <u>0-nt to 5-nt variant EREs</u> in ER ChIPSeq or ChIPExo peaks (displayed by 252 <u>ERE</u> half-site groups)
- 157 ER experiments
- Peak selection criteria =L4-L20
- Categorize the counts of ER DNA-binding at 0-nt to 5-nt variant EREs into 252 ERE half-site groups (bottom table)
- Convert these counts to (S/N) values (top table)

Table S8. (S/N) analysis of 0-nt to 5-nt Variant HREs in KR ChIPSeq Peaks (252 Half-Site Groups) [194]

- (S/N) analysis of <u>0-nt to 5-nt variant HREs</u> in KR ChIPSeq or ChIPExo peaks (displayed by 252 <u>HRE</u> half-site groups)
- 194 KR experiments
- Peak selection criteria =L4-L20
- Categorize the counts of KR DNA-binding at 0-nt to 5-nt variant HREs into 252 HRE half-site groups (bottom table)
- Convert these counts to (S/N) values (top table)

*****

Table S9. Quantify the 5 Discrete States of ER DNA-Binding at 1-nt Variant EREs in the Genome (3,2,0,0,0) [157]

- (S/N) analysis of <u>1-nt variant EREs</u> in ER ChIPSeq or ChIPExo peaks (displayed by 252 <u>ERE</u> half-site groups)
- 157 ER experiments
- Peak selection criteria =L4-L20
- Categorize the counts of ER DNA-binding at 1-nt variant EREs into 252 ERE half-site groups (bottom table)
- Convert these counts to (S/N) values (top table)
- Calculate the mean value, standard deviation, and difference from the mean of the <u>6</u> groups that reach the (+2) plateau, <u>60</u> groups reach the (+1) plateau, <u>120</u> groups reach the (0) plateau, <u>60</u> groups reach the (-1) plateau, <u>6</u> groups reach the (-2) plateau, for a total of 252 half-site groups (right-hand side)

Table S10. Quantify the 3 Discrete States of KR DNA-Binding at 1-nt Variant HREs in the Genome (4,1,0,0,0) [194]

- (S/N) analysis of <u>1-nt variant HREs</u> in KR ChIPSeq or ChIPExo peaks (displayed by 252 <u>HRE</u> half-site groups)
- 194 KR experiments
- Peak selection criteria =L4-L20
- Categorize the counts of KR DNA-binding at 1-nt variant HREs into 252 HRE half-site groups (bottom table)
- Convert these counts to (S/N) values (top table)
- Calculate the mean value, standard deviation, and difference from the mean of the <u>56</u> groups that reach the (+1) plateau, <u>140</u> groups reach the (0) plateau, <u>56</u> groups reach the (-1) plateau, for a total of 252 half-site groups (right-hand side)

*****

Table S11. (S/N) analysis of 1-nt Variant EREs in ER ChIPSeq Peaks (Variant Position) [157]

- (S/N) analysis of <u>1-nt variant EREs</u> in ER ChIPSeq or ChIPExo peaks (displayed by variant position)
- Displayed by the <u>10</u> variant positions of the <u>30</u> 1-nt variant EREs
- 157 ER experiments
- Peak selection criteria =L4-L20
- Categorize the counts of ER DNA-binding at 1-nt variant EREs by variant position (bottom table)
- Convert these counts to (S/N) values (top table)

Table S12. (S/N) analysis of 2-nt Variant EREs in ER ChIPSeq Peaks (Variant Position) [157]

- (S/N) analysis of <u>2-nt variant EREs</u> in ER ChIPSeq or ChIPExo peaks (displayed by variant position)
- Displayed by the <u>45</u> variant positions of the <u>405</u> 2-nt variant EREs
  - 5 of the 45 variant positions are the five (5) palindromic position pairs: 1-10, 2-9, 3-8, 4-7, 5-6
  - 20 of the 45 variant positions are same-side variants (i.e., the variants do not crossover the 3-nt spacer)
  - 20 of the 45 variant positions are crossover variants (i.e., the variants do crossover the 3-nt spacer)
  - Each variant position is immediately followed by its reverse-complement variant position
- 157 ER experiments
- Peak selection criteria =L4-L20
- Categorize the counts of ER DNA-binding at 2-nt variant EREs by variant position (bottom table)

- Convert these counts to (S/N) values (top table)

Table S13. (S/N) analysis of 3-nt Variant EREs in ER ChIPSeq Peaks (Variant Position) [157]

- (S/N) analysis of 3-nt variant EREs in ER ChIPSeq or ChIPExo peaks (displayed by variant position)
- Displayed by the 120 variant positions of the 3,240 3-nt variant EREs
    - 20 of the 120 variant positions are same-side variants (i.e., the variants do not crossover the 3-nt spacer)
    - 100 of the 120 variant positions are crossover variants (i.e., the variants do crossover the 3-nt spacer)
    - Each variant position is immediately followed by its reverse-complement variant position
- 157 ER experiments
- Peak selection criteria =L4-L20
- Categorize the counts of ER DNA-binding at 3-nt variant EREs by variant position (bottom table)
- Convert these counts to (S/N) values (top table)

Table S14. (S/N) analysis of 4-nt Variant EREs in ER ChIPSeq Peaks (Variant Position) [157]

- (S/N) analysis of 4-nt variant EREs in ER ChIPSeq or ChIPExo peaks (displayed by variant position)
- Displayed by the 210 variant positions of the 17,010 4-nt variant EREs
    - 10 of the 210 variant positions are the five (5) palindromic position pairs: 1-3-8-10, 3-5-6-8, 3-4-7-8, 2-3-8-9, 1-5-6-10, 1-4-7-10, 1-2-9-10, 4-5-6-7, 2-5-6-9, 2-4-7-9
    - 10 of the 210 variant positions are same-side variants (i.e., the variants do not crossover the 3-nt spacer)
    - 190 of the 45 variant positions are crossover variants (i.e., the variants do crossover the 3-nt spacer)
    - Each variant position is immediately followed by its reverse-complement variant position
- 157 ER experiments
- Peak selection criteria =L4-L20
- Categorize the counts of ER DNA-binding at 4-nt variant EREs by variant position (bottom table)
- Convert these counts to (S/N) values (top table)

Table S15. (S/N) analysis of 5-nt Variant EREs in ER ChIPSeq Peaks (Variant Position) [157]

- (S/N) analysis of 5-nt variant EREs in ER ChIPSeq or ChIPExo peaks (displayed by variant position)
- Displayed by the 252 variant positions of the 61,236 5-nt variant EREs
    - 2 of the 252 variant positions are same-side variants (i.e., the variants do not crossover the 3-nt spacer)
    - 250 of the 252 variant positions are crossover variants (i.e., the variants do crossover the 3-nt spacer)
    - Each variant position is immediately followed by its reverse-complement variant position
- 157 ER experiments
- Peak selection criteria =L4-L20
- Categorize the counts of ER DNA-binding at 5-nt variant EREs by variant position (bottom table)
- Convert these counts to (S/N) values (top table)

*****

Table S16. (S/N) analysis of 1-nt Variant HREs in KR ChIPSeq Peaks (Variant Position) [194]

- (S/N) analysis of 1-nt variant HREs in KR ChIPSeq or ChIPExo peaks (displayed by variant position)
- Displayed by the 10 variant positions of the 30 1-nt variant HREs
- 194 KR experiments
- Peak selection criteria =L4-L20
- Categorize the counts of KR DNA-binding at 1-nt variant HREs by variant position (bottom table)
- Convert these counts to (S/N) values (top table)

Table S17. (S/N) analysis of 2-nt Variant HREs in KR ChIPSeq Peaks (Variant Position) [194]

- (S/N) analysis of 2-nt variant HREs in KR ChIPSeq or ChIPExo peaks (displayed by variant position)
- Displayed by the 45 variant positions of the 405 2-nt variant HREs
    - 5 of the 45 variant positions are the five (5) palindromic position pairs: 1-10, 2-9, 3-8, 4-7, 5-6
    - 20 of the 45 variant positions are same-side variants (i.e., the variants do not crossover the 3-nt spacer)
    - 20 of the 45 variant positions are crossover variants (i.e., the variants do crossover the 3-nt spacer)
    - Each variant position is immediately followed by its reverse-complement variant position
- 194 KR experiments
- Peak selection criteria =L4-L20
- Categorize the counts of KR DNA-binding at 2-nt variant HREs by variant position (bottom table)
- Convert these counts to (S/N) values (top table)

Table S18. (S/N) analysis of 3-nt Variant HREs in KR ChIPSeq Peaks (Variant Position) [194]

- (S/N) analysis of <u>3-nt variant HREs</u> in KR ChIPSeq or ChIPExo peaks (displayed by variant position)
- Displayed by the <u>120</u> variant positions of the <u>3,240</u> 3-nt variant HREs
  - 20 of the 120 variant positions are same-side variants (i.e., the variants do not crossover the 3-nt spacer)
  - 100 of the 120 variant positions are crossover variants (i.e., the variants do crossover the 3-nt spacer)
  - Each variant position is immediately followed by its reverse-complement variant position
- 194 KR experiments
- Peak selection criteria =L4-L20
- Categorize the counts of KR DNA-binding at 3-nt variant HREs by variant position (bottom table)
- Convert these counts to (S/N) values (top table)

Table S19. (S/N) analysis of 4-nt Variant HREs in KR ChIPSeq Peaks (Variant Position) [194]

- (S/N) analysis of <u>4-nt variant HREs</u> in KR ChIPSeq or ChIPExo peaks (displayed by variant position)
- Displayed by the <u>210</u> variant positions of the <u>17,010</u> 4-nt variant HREs
  - 10 of the 210 variant positions are the five (5) palindromic position pairs: 2-5-6-9, 1-2-9-10, 2-4-7-9, 2-3-8-9, 1-5-6-10, 4-5-6-7, 3-5-6-8, 1-4-7-10, 1-3-8-10, 3-4-7-8
  - 10 of the 210 variant positions are same-side variants (i.e., the variants do not crossover the 3-nt spacer)
  - 190 of the 45 variant positions are crossover variants (i.e., the variants do crossover the 3-nt spacer)
  - Each variant position is immediately followed by its reverse-complement variant position
- 194 KR experiments
- Peak selection criteria =L4-L20
- Categorize the counts of KR DNA-binding at 4-nt variant HREs by variant position (bottom table)
- Convert these counts to (S/N) values (top table)

Table S20. (S/N) analysis of 5-nt Variant HREs in KR ChIPSeq Peaks (Variant Position) [194]

- (S/N) analysis of <u>5-nt variant HREs</u> in KR ChIPSeq or ChIPExo peaks (displayed by variant position)
- Displayed by the <u>252</u> variant positions of the <u>61,236</u> 5-nt variant HREs
  - 2 of the 252 variant positions are same-side variants (i.e., the variants do not crossover the 3-nt spacer)
  - 250 of the 252 variant positions are crossover variants (i.e., the variants do crossover the 3-nt spacer)
  - Each variant position is immediately followed by its reverse-complement variant position
- 194 KR experiments
- Peak selection criteria =L4-L20
- Categorize the counts of KR DNA-binding at 5-nt variant HREs by variant position (bottom table)
- Convert these counts to (S/N) values (top table)

Table S21-S40. 15-nt ERE and HRE DNA Element Analysis

The analyses in this section were completed by overlapping the location coordinates of each 0-nt to 6-nt variant 15-nt ERE or HRE DNA element in the genome and the location coordinates of the ChIPSeq or ChIPExo peaks in an experiment. The 0-nt variant consensus palindromic 15-nt ERE DNA element (5′-AGGTCAnnnTGACCT-3′) and 0-nt variant consensus palindromic 15-nt HRE DNA element (5′-AGAACAnnnTGTTCT-3′) have twelve (12) primary positions. The 0-nt to 6-nt variant 15-nt ERE or HRE DNA elements include the 1 0-nt variant consensus palindromic DNA element, 36 1-nt variant DNA elements (12 variant positions), 594 2-nt variant DNA elements (66 variant positions), 5,940 3-nt variant DNA elements (220 variant positions), 40,095 4-nt variant DNA elements (495 variant positions), 192,456 5-nt variant DNA elements (792 variant positions), and 673,596 6-nt variant DNA elements (924 variant positions), for a total of 912,718 DNA elements.

Table S21. Categorize 0-nt to 6-nt Variant 15-nt EREs into 924 Half-Site Groups (Variant Position)

- Categorize the 912,718 0-nt to 6-nt variant 15-nt ERE DNA elements into 924 half-site groups (6 positions are fixed, allowing for *up to* 6 positions to be varied)
- Each of the 924 ERE half-site groups contain: 1 0-nt variant consensus palindromic ERE DNA element, 18 1-nt variant ERE DNA elements, 135 2-nt variant ERE DNA elements, 540 3-nt variant ERE DNA elements, 1215 4-nt variant ERE DNA elements, 1458 5-nt variant ERE DNA elements, 729 6-nt variant ERE DNA elements, for a total of 4,096 ERE DNA elements
- 924 half-site groups x 4,096 DNA elements per half-site group =3,784,704 DNA elements (912,718 unique)

Table S22. Categorize 0-nt to 6-nt Variant 15-nt HREs into 924 Half-Site Groups (Variant Position)

- Categorize the 912,718 0-nt to 6-nt variant 15-nt HRE DNA elements into 924 half-site groups (6 positions are fixed, allowing for *up to* 6 positions to be varied)
- Each of the 924 HRE half-site groups contain: 1 0-nt variant consensus palindromic HRE DNA element, 18 1-nt variant HRE DNA elements, 135 2-nt variant HRE DNA elements, 540 3-nt variant HRE DNA elements, 1215 4-nt variant HRE DNA elements, 1458 5-nt variant HRE DNA elements, 729 6-nt variant HRE DNA elements, for a total of 4,096 HRE DNA elements
- 924 half-site groups x 4,096 DNA elements per half-site group =3,784,704 DNA elements (912,718 unique)

Table S23. Categorize 0-nt to 6-nt Variant 15-nt EREs into 924 Half-Site Groups (Sequence)

- Categorize the 912,718 0-nt to 6-nt variant 15-nt ERE DNA elements into 924 half-site groups (6 positions are fixed, allowing for *up to* 6 positions to be varied)
- Each of the 924 ERE half-site groups contain: 1 0-nt variant consensus palindromic ERE DNA element, 18 1-nt variant ERE DNA elements, 135 2-nt variant ERE DNA elements, 540 3-nt variant ERE DNA elements, 1215 4-nt variant ERE DNA elements, 1458 5-nt variant ERE DNA elements, 729 6-nt variant ERE DNA elements, for a total of 4,096 ERE DNA elements
- 924 half-site groups x 4,096 DNA elements per half-site group =3,784,704 DNA elements (912,718 unique)

Table S24. Categorize 0-nt to 6-nt Variant 15-nt HREs into 924 Half-Site Groups (Sequence)

- Categorize the 912,718 0-nt to 6-nt variant 15-nt HRE DNA elements into 924 half-site groups (6 positions are fixed, allowing for *up to* 6 positions to be varied)
- Each of the 924 HRE half-site groups contain: 1 0-nt variant consensus palindromic HRE DNA element, 18 1-nt variant HRE DNA elements, 135 2-nt variant HRE DNA elements, 540 3-nt variant HRE DNA elements, 1215 4-nt variant HRE DNA elements, 1458 5-nt variant HRE DNA elements, 729 6-nt variant HRE DNA elements, for a total of 4,096 HRE DNA elements
- 924 half-site groups x 4,096 DNA elements per half-site group =3,784,704 DNA elements (912,718 unique)

Table S25. X-Axis Order of 924 ERE Half-Site Groups

- (S/N) analysis of 0-nt to 6-nt variant EREs in ER ChIPSeq or ChIPExo peaks (displayed by 924 ERE half-site groups)
- X-axis order
  - 452 half-site groups [left-to-right: 0 vacancies, 1 vacancy, 2 vacancies], 20 half-site groups [3 vacancies], followed by their 452 reverse-complements
  - Of these 924 half-site groups, 64 have zero vacancies, 480 have one vacancy, 360 have two vacancies, 20 have three vacancies
  - The x-axis is labeled by the reverse-complement vacancy position ID (primary label) and reverse-complement double occupant position ID (secondary label)
  - The order of the reverse-complement vacancy position IDs for ERE is 4-9 > 1-12 > 2-11 > 6-7 > 5-8 > 3-10

Table S26. X-Axis Order of 924 HRE Half-Site Groups

- (S/N) analysis of 0-nt to 6-nt variant HREs in KR ChIPSeq or ChIPExo peaks (displayed by 924 HRE half-site groups)

- X-axis order
  - 452 half-site groups [left-to-right: 0 vacancies, 1 vacancy, 2 vacancies], 20 half-site groups [3 vacancies], followed by their 452 reverse-complements
  - Of these 924 half-site groups, 64 have zero vacancies, 480 have one vacancy, 360 have two vacancies, 20 have three vacancies
  - The x-axis is labeled by the reverse-complement vacancy position ID (primary label) and reverse-complement double occupant position ID (secondary label)
  - The order of the reverse-complement vacancy position IDs for ERE is 3-10 > 1-12 > 6-7 > 2-11 > 5-8 > 4-9

Table S27. (S/N) analysis of 0-nt to 6-nt Variant EREs in ER ChIPSeq Peaks (924 Half-Site Groups) [157]

- (S/N) analysis of <u>0-nt to 6-nt variant EREs</u> in ER ChIPSeq or ChIPExo peaks (displayed by 924 <u>ERE</u> half-site groups)
- 157 ER experiments
- Peak selection criteria =L4-L20
- Categorize the counts of ER DNA-binding at 0-nt to 6-nt variant EREs into 924 ERE half-site groups (bottom table)
- Convert these counts to (S/N) values (top table)

Table S28. (S/N) analysis of 0-nt to 6-nt Variant HREs in KR ChIPSeq Peaks (924 Half-Site Groups) [194]

- (S/N) analysis of <u>0-nt to 6-nt variant HREs</u> in KR ChIPSeq or ChIPExo peaks (displayed by 924 <u>HRE</u> half-site groups)
- 194 KR experiments
- Peak selection criteria =L4-L20
- Categorize the counts of KR DNA-binding at 0-nt to 6-nt variant HREs into 924 HRE half-site groups (bottom table)
- Convert these counts to (S/N) values (top table)

*****

Table S29. (S/N) analysis of 1-nt Variant EREs in ER ChIPSeq Peaks (Variant Position) [157]

- (S/N) analysis of <u>1-nt variant EREs</u> in ER ChIPSeq or ChIPExo peaks (displayed by variant position)
- Displayed by the <u>12</u> variant positions of the <u>36</u> 1-nt variant EREs
- 157 ER experiments
- Peak selection criteria =L4-L20
- Categorize the counts of ER DNA-binding at 1-nt variant EREs by variant position (bottom table)
- Convert these counts to (S/N) values (top table)

Table S30. (S/N) analysis of 2-nt Variant EREs in ER ChIPSeq Peaks (Variant Position) [157]

- (S/N) analysis of <u>2-nt variant EREs</u> in ER ChIPSeq or ChIPExo peaks (displayed by variant position)
- Displayed by the <u>66</u> variant positions of the <u>594</u> 2-nt variant EREs
  - 6 of the 66 variant positions are the six (6) palindromic position pairs: 1-12, 2-11, 3-10, 4-9, 5-8, 6-7
  - 30 of the 66 variant positions are same-side variants (i.e., the variants do not crossover the 3-nt spacer)
  - 30 of the 66 variant positions are crossover variants (i.e., the variants do crossover the 3-nt spacer)
  - Each variant position is immediately followed by its reverse-complement variant position
- 157 ER experiments
- Peak selection criteria =L4-L20
- Categorize the counts of ER DNA-binding at 2-nt variant EREs by variant position (bottom table)
- Convert these counts to (S/N) values (top table)

Table S31. (S/N) analysis of 3-nt Variant EREs in ER ChIPSeq Peaks (Variant Position) [157]

- (S/N) analysis of <u>3-nt variant EREs</u> in ER ChIPSeq or ChIPExo peaks (displayed by variant position)
- Displayed by the <u>220</u> variant positions of the <u>5,940</u> 3-nt variant EREs
  - 40 of the 220 variant positions are same-side variants (i.e., the variants do not crossover the 3-nt spacer)
  - 180 of the 220 variant positions are crossover variants (i.e., the variants do crossover the 3-nt spacer)
  - Each variant position is immediately followed by its reverse-complement variant position
- 157 ER experiments
- Peak selection criteria =L4-L20
- Categorize the counts of ER DNA-binding at 3-nt variant EREs by variant position (bottom table)
- Convert these counts to (S/N) values (top table)

Table S32. (S/N) analysis of 4-nt Variant EREs in ER ChIPSeq Peaks (Variant Position) [157]

- (S/N) analysis of <u>4-nt variant EREs</u> in ER ChIPSeq or ChIPExo peaks (displayed by variant position)

- Displayed by the 495 variant positions of the 40,095 4-nt variant EREs
  - 15 of the 495 variant positions are the five (5) palindromic position pairs: 2-4-9-11, 4-6-7-9, 4-5-8-9, 3-4-9-10, 2-6-7-11, 2-5-8-11, 2-3-10-11, 5-6-7-8, 3-6-7-10, 3-5-8-10, 1-4-9-12, 1-2-11-12, 1-6-7-12, 1-5-8-12, 1-3-10-12
  - 30 of the 495 variant positions are same-side variants (i.e., the variants do not crossover the 3-nt spacer)
  - 450 of the 495 variant positions are crossover variants (i.e., the variants do crossover the 3-nt spacer)
  - Each variant position is immediately followed by its reverse-complement variant position
- 157 ER experiments
- Peak selection criteria =L4-L20
- Categorize the counts of ER DNA-binding at 4-nt variant EREs by variant position (bottom table)
- Convert these counts to (S/N) values (top table)

Table S33. (S/N) analysis of 5-nt Variant EREs in ER ChIPSeq Peaks (Variant Position) [157]

- (S/N) analysis of 5-nt variant EREs in ER ChIPSeq or ChIPExo peaks (displayed by variant position)
- Displayed by the 792 variant positions of the 192,456 5-nt variant EREs
  - 12 of the 792 variant positions are same-side variants (i.e., the variants do not crossover the 3-nt spacer)
  - 780 of the 792 variant positions are crossover variants (i.e., the variants do crossover the 3-nt spacer)
  - Each variant position is immediately followed by its reverse-complement variant position
- 157 ER experiments
- Peak selection criteria =L4-L20
- Categorize the counts of ER DNA-binding at 5-nt variant EREs by variant position (bottom table)
- Convert these counts to (S/N) values (top table)

Table S34. (S/N) analysis of 6-nt Variant EREs in ER ChIPSeq Peaks (Variant Position) [157]

- (S/N) analysis of 6-nt variant EREs in ER ChIPSeq or ChIPExo peaks (displayed by variant position)
- Displayed by the 924 variant positions of the 673,596 6-nt variant EREs
  - 20 of the 924 variant positions are the five (5) palindromic position pairs: 1-4-6-7-9-12, 2-3-4-9-10-11, 1-3-4-9-10-12, 1-2-4-9-11-12, 2-4-6-7-9-11, 1-4-5-8-9-12, 2-4-5-8-9-11, 3-4-6-7-9-10, 1-2-6-7-11-12, 4-5-6-7-8-9, 1-2-5-8-11-12, 1-3-6-7-10-12, 1-2-3-10-11-12, 3-4-5-8-9-10, 1-5-6-7-8-12, 2-3-5-8-10-11, 2-3-6-7-10-11, 1-3-5-8-10-12, 3-5-6-7-8-10, 2-5-6-7-8-11
  - 2 of the 924 variant positions are same-side variants (i.e., the variants do not crossover the 3-nt spacer)
  - 922 of the 924 variant positions are crossover variants (i.e., the variants do crossover the 3-nt spacer)
  - Each variant position is immediately followed by its reverse-complement variant position
- 157 ER experiments
- Peak selection criteria =L4-L20
- Categorize the counts of ER DNA-binding at 6-nt variant EREs by variant position (bottom table)
- Convert these counts to (S/N) values (top table)

*****

Table S35. (S/N) analysis of 1-nt Variant HREs in KR ChIPSeq Peaks (Variant Position) [194]

- (S/N) analysis of 1-nt variant HREs in KR ChIPSeq or ChIPExo peaks (displayed by variant position)
- Displayed by the 12 variant positions of the 36 1-nt variant HREs
- 194 KR experiments
- Peak selection criteria =L4-L20
- Categorize the counts of KR DNA-binding at 1-nt variant HREs by variant position (bottom table)
- Convert these counts to (S/N) values (top table)

Table S36. (S/N) analysis of 2-nt Variant HREs in KR ChIPSeq Peaks (Variant Position) [194]

- (S/N) analysis of 2-nt variant HREs in KR ChIPSeq or ChIPExo peaks (displayed by variant position)
- Displayed by the 66 variant positions of the 594 2-nt variant HREs
  - 6 of the 66 variant positions are the six (6) palindromic position pairs: 1-12, 2-11, 3-10, 4-9, 5-8, 6-7
  - 30 of the 66 variant positions are same-side variants (i.e., the variants do not crossover the 3-nt spacer)
  - 30 of the 66 variant positions are crossover variants (i.e., the variants do crossover the 3-nt spacer)
  - Each variant position is immediately followed by its reverse-complement variant position
- 194 KR experiments
- Peak selection criteria =L4-L20
- Categorize the counts of KR DNA-binding at 2-nt variant HREs by variant position (bottom table)
- Convert these counts to (S/N) values (top table)

Table S37. (S/N) analysis of 3-nt Variant HREs in KR ChIPSeq Peaks (Variant Position) [194]

- (S/N) analysis of 3-nt variant HREs in KR ChIPSeq or ChIPExo peaks (displayed by variant position)
- Displayed by the 220 variant positions of the 5,940 3-nt variant HREs
    - 40 of the 220 variant positions are same-side variants (i.e., the variants do not crossover the 3-nt spacer)
    - 180 of the 220 variant positions are crossover variants (i.e., the variants do crossover the 3-nt spacer)
    - Each variant position is immediately followed by its reverse-complement variant position
- 194 KR experiments
- Peak selection criteria =L4-L20
- Categorize the counts of KR DNA-binding at 3-nt variant HREs by variant position (bottom table)
- Convert these counts to (S/N) values (top table)

Table S38. (S/N) analysis of 4-nt Variant HREs in KR ChIPSeq Peaks (Variant Position) [194]

- (S/N) analysis of 4-nt variant HREs in KR ChIPSeq or ChIPExo peaks (displayed by variant position)
- Displayed by the 495 variant positions of the 40,095 4-nt variant HREs
    - 15 of the 495 variant positions are the five (5) palindromic position pairs: 3-6-7-10, 2-3-10-11, 3-5-8-10, 3-4-9-10, 2-6-7-11, 5-6-7-8, 4-6-7-9, 2-5-8-11, 2-4-9-11, 4-5-8-9, 1-3-10-12, 1-6-7-12, 1-2-11-12, 1-5-8-12, 1-4-9-12
    - 30 of the 495 variant positions are same-side variants (i.e., the variants do not crossover the 3-nt spacer)
    - 450 of the 495 variant positions are crossover variants (i.e., the variants do crossover the 3-nt spacer)
    - Each variant position is immediately followed by its reverse-complement variant position
- 194 KR experiments
- Peak selection criteria =L4-L20
- Categorize the counts of KR DNA-binding at 4-nt variant HREs by variant position (bottom table)
- Convert these counts to (S/N) values (top table)

Table S39. (S/N) analysis of 5-nt Variant HREs in KR ChIPSeq Peaks (Variant Position) [194]

- (S/N) analysis of 5-nt variant HREs in KR ChIPSeq or ChIPExo peaks (displayed by variant position)
- Displayed by the 792 variant positions of the 192,456 5-nt variant HREs
    - 12 of the 792 variant positions are same-side variants (i.e., the variants do not crossover the 3-nt spacer)
    - 780 of the 792 variant positions are crossover variants (i.e., the variants do crossover the 3-nt spacer)
    - Each variant position is immediately followed by its reverse-complement variant position
- 194 KR experiments
- Peak selection criteria =L4-L20
- Categorize the counts of KR DNA-binding at 5-nt variant HREs by variant position (bottom table)
- Convert these counts to (S/N) values (top table)

Table S40. (S/N) analysis of 6-nt Variant HREs in KR ChIPSeq Peaks (Variant Position) [194]

- (S/N) analysis of 6-nt variant HREs in KR ChIPSeq or ChIPExo peaks (displayed by variant position)
- Displayed by the 924 variant positions of the 673,596 6-nt variant HREs
    - 20 of the 924 variant positions are the five (5) palindromic position pairs: 1-2-3-10-11-12, 3-4-5-8-9-10, 1-3-4-9-10-12, 1-2-4-9-11-12, 2-3-6-7-10-11, 1-3-5-8-10-12, 3-5-6-7-8-10, 2-5-6-7-8-11, 1-2-6-7-11-12, 4-5-6-7-8-9, 1-5-6-7-8-12, 2-3-5-8-10-11, 1-4-6-7-9-12, 2-3-4-9-10-11, 1-2-5-8-11-12, 1-3-6-7-10-12, 2-4-6-7-9-11, 1-4-5-8-9-12, 2-4-5-8-9-11, 3-4-6-7-9-10
    - 2 of the 924 variant positions are same-side variants (i.e., the variants do not crossover the 3-nt spacer)
    - 922 of the 924 variant positions are crossover variants (i.e., the variants do crossover the 3-nt spacer)
    - Each variant position is immediately followed by its reverse-complement variant position
- 194 KR experiments
- Peak selection criteria =L4-L20
- Categorize the counts of KR DNA-binding at 6-nt variant HREs by variant position (bottom table)
- Convert these counts to (S/N) values (top table)

Table S41-S46. Transform the 13-nt DNA Element Analysis to the 15-nt DNA Element Analysis

Analysis of the 13-nt ERE and HRE DNA elements include the 1 0-nt variant consensus palindromic DNA element, 30 1-nt variant DNA elements (10 variant positions), 405 2-nt variant DNA elements (45 variant positions), 3,240 3-nt variant DNA elements (120 variant positions), 17,010 4-nt variant DNA elements (210 variant positions), and 61,236 5-nt variant DNA elements (252 variant positions), for a total of 81,922 DNA elements. Analysis of the 15-nt ERE and HRE DNA elements include the 1 0-nt variant consensus palindromic DNA element, 36 1-nt variant DNA elements (12 variant positions), 594 2-nt variant DNA elements (66 variant positions), 5,940 3-nt variant DNA elements (220 variant positions), 40,095 4-nt variant DNA elements (495 variant positions), 192,456 5-nt variant DNA elements (792 variant positions), and 673,596 6-nt variant DNA elements (924 variant positions), for a total of 912,718 DNA elements.

Table S41. Transform the 13-nt EREs to the 15-nt EREs (Variant Position)

- Each of the 81,922 0-nt to 5-nt variant 13-nt EREs splits into 16 categories of the 15-nt EREs
- The 15-nt EREs are displayed by their variant position
- 16 categories:
  - 0-nt variant difference: an adenine (A) is in position 1 and a thymine (T) is in position 12 of the 15-nt DNA element [=1 DNA element]
  - 1-nt variant difference: one of the three (3) alternative nucleotide possibilities are in position 1 or position 12 [=6 DNA elements]
  - 2-nt variant difference: one of the three (3) alternative nucleotide possibilities are in position 1 and position 12 [=9 DNA elements]
- 81,922 0-nt to 5-nt variant 13-nt EREs x 16 categories =1,310,752 15-nt EREs
- The 81,922 0-nt to 5-nt variant 13-nt EREs include 551,124 EREs that are not part of the 912,718 0-nt to 6-nt variant 15-nt EREs (these represent a 7-nt variant of the 15-nt ERE)
- Thus, of the 81,922 0-nt to 5-nt variant 13-nt EREs and the 912,718 0-nt to 6-nt variant 15-nt EREs, 759,628 EREs correspond to each other

Table S42. Transform the 13-nt HREs to the 15-nt HREs (Variant Position)

- Each of the 81,922 0-nt to 5-nt variant 13-nt HREs splits into 16 categories of the 15-nt HREs
- The 15-nt HREs are displayed by their variant position
- 16 categories:
  - 0-nt variant difference: an adenine (A) is in position 1 and a thymine (T) is in position 12 of the 15-nt DNA element [=1 DNA element]
  - 1-nt variant difference: one of the three (3) alternative nucleotide possibilities are in position 1 or position 12 [=6 DNA elements]
  - 2-nt variant difference: one of the three (3) alternative nucleotide possibilities are in position 1 and position 12 [=9 DNA elements]
- 81,922 0-nt to 5-nt variant 13-nt HREs x 16 categories =1,310,752 15-nt HREs
- The 81,922 0-nt to 5-nt variant 13-nt HREs include 551,124 HREs that are not part of the 912,718 0-nt to 6-nt variant 15-nt HREs (these represent a 7-nt variant of the 15-nt HRE)
- Thus, of the 81,922 0-nt to 5-nt variant 13-nt HREs and the 912,718 0-nt to 6-nt variant 15-nt HREs, 759,628 HREs correspond to each other

Table S43. Transform the 13-nt EREs to the 15-nt EREs (Sequence)

- Each of the 81,922 0-nt to 5-nt variant 13-nt EREs splits into 16 categories of the 15-nt EREs
- The 15-nt EREs are displayed by their DNA sequence
- 16 categories:
  - 0-nt variant difference: an adenine (A) is in position 1 and a thymine (T) is in position 12 of the 15-nt DNA element [=1 DNA element]
  - 1-nt variant difference: one of the three (3) alternative nucleotide possibilities are in position 1 or position 12 [=6 DNA elements]
  - 2-nt variant difference: one of the three (3) alternative nucleotide possibilities are in position 1 and position 12 [=9 DNA elements]
- 81,922 0-nt to 5-nt variant 13-nt EREs x 16 categories =1,310,752 15-nt EREs

- The 81,922 0-nt to 5-nt variant 13-nt EREs include 551,124 EREs that are not part of the 912,718 0-nt to 6-nt variant 15-nt EREs (these represent a 7-nt variant of the 15-nt ERE)
- Thus, of the 81,922 0-nt to 5-nt variant 13-nt EREs and the 912,718 0-nt to 6-nt variant 15-nt EREs, 759,628 EREs correspond to each other

Table S44. Transform the 13-nt HREs to the 15-nt HREs (Sequence)

- Each of the 81,922 0-nt to 5-nt variant 13-nt HREs splits into 16 categories of the 15-nt HREs
- The 15-nt HREs are displayed by their DNA sequence
- 16 categories:
    - 0-nt variant difference: an adenine (A) is in position 1 and a thymine (T) is in position 12 of the 15-nt DNA element [=1 DNA element]
    - 1-nt variant difference: one of the three (3) alternative nucleotide possibilities are in position 1 or position 12 [=6 DNA elements]
    - 2-nt variant difference: one of the three (3) alternative nucleotide possibilities are in position 1 and position 12 [=9 DNA elements]
- 81,922 0-nt to 5-nt variant 13-nt HREs x 16 categories =1,310,752 15-nt HREs
- The 81,922 0-nt to 5-nt variant 13-nt HREs include 551,124 HREs that are not part of the 912,718 0-nt to 6-nt variant 15-nt HREs (these represent a 7-nt variant of the 15-nt HRE)
- Thus, of the 81,922 0-nt to 5-nt variant 13-nt HREs and the 912,718 0-nt to 6-nt variant 15-nt HREs, 759,628 HREs correspond to each other

Table S45. Transform the 15-nt EREs to the 13-nt EREs

- The 912,718 0-nt to 6-nt variant 15-nt EREs and their corresponding 13-nt EREs
- The 912,718 0-nt to 6-nt variant 15-nt EREs include 153,090 EREs that are not part of the 81,922 0-nt to 5-nt variant 13-nt EREs (these represent a 6-nt variant of the 13-nt ERE)
- Thus, of the 81,922 0-nt to 5-nt variant 13-nt EREs and the 912,718 0-nt to 6-nt variant 15-nt EREs, 759,628 EREs correspond to each other
- Example Experiment (WT-E2-1hr.L4) [76,163 peaks, 146-nt peak length]
    - The 0-nt variant consensus palindromic 13-nt ERE (5′-GGTCAnnnTGACC-3′) occurred 1,202 times in an ER experiment
    - Each of those 1,202 EREs are part of a 15-nt ERE
    - Analysis of ER DNA-binding at all possible 15-nt EREs splits those 1,202 13-nt EREs into 16 categories (i.e., 4-nt possibilities in position 1 and 4-nt possibilities in position 12): 109 (1T), 155 (1G), 19 (1C), 13 (12G), 151 (12C), 110 (12A), 11 (1-12 TG), 67 (1-12 TC), 17 (1-12 GG), 305 (1-12 AT), 29 (1-12 TA), 107 (1-12 GC), 5 (1-12 CG), 14 (1-12 CC), 88 (1-12 GA), 2 (1-12 CA), totaling 1,202 **(Figure S33)**

Table S46. Transform the 15-nt HREs to the 13-nt HREs

- The 912,718 0-nt to 6-nt variant 15-nt HREs and their corresponding 13-nt HREs
- The 912,718 0-nt to 6-nt variant 15-nt HREs include 153,090 HREs that are not part of the 81,922 0-nt to 5-nt variant 13-nt HREs (these represent a 6-nt variant of the 13-nt HRE)
- Thus, of the 81,922 0-nt to 5-nt variant 13-nt HREs and the 912,718 0-nt to 6-nt variant 15-nt HREs, 759,628 HREs correspond to each other

Table S47-S50. Inversion Symmetry of the Single-Stranded Mouse and Human Genome

Evaluation of the number of times every DNA element (1-nt to 20-nt) occurs in the single-stranded mouse (mm10) and human (hg19) genome. This includes all DNA elements from the four (4) 1-nt DNA elements to the 1 trillion (1,099,511,627,776) 20-nt DNA elements. Comparing the population count of every DNA element to the population count of its reverse-complement DNA element revealed a near perfect inversion symmetry for all 1-nt to 20-nt DNA elements in the single-stranded mouse and human genome. This property is also maintained at the level of each individual chromosome, except for chromosome M (i.e., the mitochondrial DNA). In addition to demonstrating the inherent inversion symmetry structure for all DNA elements in the single-stranded genome, we further demonstrate the absence of analog symmetries between reverse pairs and complement pairs of DNA elements in the single-stranded genome.

Table S47. Population Count of 1-nt to 20-nt DNA Elements in the Single-Stranded Mouse Genome [1.4 T]

- Correlation coefficient, slope, y-intercept, and general error fraction (GEF) (ratio of average error to average length) between the population count of every DNA element (1-nt to 20-nt) and its reverse DNA element, its complement DNA element, and its reverse-complement DNA element (=6 comparisons) in the single-stranded mouse (mm10) genome

Table S48. Population Count of 1-nt to 20-nt DNA Elements in the Single-Stranded Human Genome [1.4 T]

- Correlation coefficient, slope, y-intercept, and general error fraction (GEF) (ratio of average error to average length) between the population count of every DNA element (1-nt to 20-nt) and its reverse DNA element, its complement DNA element, and its reverse-complement DNA element (=6 comparisons) in the single-stranded human (hg19) genome

Table S49. Population Count of 1-nt to 20-nt DNA Elements in the Single-Stranded Mouse Genome (Chromosome)

- Correlation coefficient, slope, y-intercept, and general error fraction (GEF) (ratio of average error to average length) between the population count of every DNA element (1-nt to 20-nt) and its reverse DNA element, its complement DNA element, and its reverse-complement DNA element (=6 comparisons) in the single-stranded mouse (mm10) genome

Table S50. Population Count of 1-nt to 20-nt DNA Elements in the Single-Stranded Human Genome (Chromosome)

- Correlation coefficient, slope, y-intercept, and general error fraction (GEF) (ratio of average error to average length) between the population count of every DNA element (1-nt to 20-nt) and its reverse DNA element, its complement DNA element, and its reverse-complement DNA element (=6 comparisons) in the single-stranded human (hg19) genome

Table S51-S54. p53 DNA Element Analysis

The analyses in this section were completed by overlapping the location coordinates of each 0-nt to 5-nt variant 10-nt p53RE DNA element in the genome and the location coordinates of the ChIPSeq or ChIPExo peaks in an experiment. The 0-nt variant consensus palindromic 10-nt p53RE DNA element (5′-TGCCCGGGCA-3′) has ten (10) primary positions. The 0-nt to 5-nt variant p53RE DNA elements include the 1 0-nt variant consensus palindromic DNA element, 30 1-nt variant DNA elements (10 variant positions), 405 2-nt variant DNA elements (45 variant positions), 3,240 3-nt variant DNA elements (120 variant positions), 17,010 4-nt variant DNA elements (210 variant positions), and 61,236 5-nt variant DNA elements (252 variant positions), for a total of 81,922 DNA elements.

Table S51. Categorize 0-nt to 5-nt Variant 10-nt p53REs into 252 Half-Site Groups (Variant Position)

- Categorize the 81,922 0-nt to 5-nt variant 10-nt p53RE DNA elements into 252 half-site groups (5 positions are fixed, allowing for *up to* 5 positions to be varied)
- Each of the 252 p53RE half-site groups contain: 1 0-nt variant consensus palindromic p53RE DNA element, 15 1-nt variant p53RE DNA elements, 90 2-nt variant p53RE DNA elements, 270 3-nt variant p53RE DNA elements, 405 4-nt variant p53RE DNA elements, 243 5-nt variant p53RE DNA elements, for a total of 1,024 p53RE DNA elements
- 252 half-site groups x 1,024 DNA elements per half-site group =258,048 DNA elements (81,922 unique)

Table S52. Categorize 0-nt to 5-nt Variant 10-nt p53REs into 252 Half-Site Groups (Sequence)

- Categorize the 81,922 0-nt to 5-nt variant 10-nt p53RE DNA elements into 252 half-site groups (5 positions are fixed, allowing for *up to* 5 positions to be varied)
- Each of the 252 p53RE half-site groups contain: 1 0-nt variant consensus palindromic p53RE DNA element, 15 1-nt variant p53RE DNA elements, 90 2-nt variant p53RE DNA elements, 270 3-nt variant p53RE DNA elements, 405 4-nt variant p53RE DNA elements, 243 5-nt variant p53RE DNA elements, for a total of 1,024 p53RE DNA elements
- 252 half-site groups x 1,024 DNA elements per half-site group =258,048 DNA elements (81,922 unique)

Table S53. X-Axis Order of 252 p53RE Half-Site Groups

- (S/N) analysis of 0-nt to 5-nt variant p53REs in p53 ChIPSeq or ChIPExo peaks (displayed by 252 p53RE half-site groups)
- X-axis order
    - 126 half-site groups [left-to-right: 0 vacancies, 1 vacancy, 2 vacancies] followed by their 126 reverse-complements
    - Of these 252 half-site groups, 32 have zero vacancies, 160 have one vacancy, 60 have two vacancies
    - The x-axis is labeled by the reverse-complement vacancy position ID (primary label) and reverse-complement double occupant position ID (secondary label)
    - The order of the reverse-complement vacancy position IDs for p53RE is 5-6 > 1-10 > 3-8 > 4-7 > 2-9

Table S54. (S/N) analysis of 0-nt to 5-nt Variant p53REs in p53 ChIPSeq Peaks (252 Half-Site Groups) [22]

- (S/N) analysis of 0-nt to 5-nt variant p53REs in p53 ChIPSeq or ChIPExo peaks (displayed by 252 p53RE half-site groups)
- 22 p53 experiments
- Peak selection criteria =L4-L20
- Categorize the counts of p53 DNA-binding at 0-nt to 5-nt variant p53REs into 252 p53RE half-site groups (bottom table)
- Convert these counts to (S/N) values (top table)

Table S55-S63. Number of DNA Elements (ERE, HRE, p53RE) in the Mouse and Human Genome

The number of 0-nt to 5-nt variants of the 13-nt ERE and HRE consensus palindromic DNA element on the positive/sense strand in the mouse (mm10) and human (hg19) genome. Here the 13-nt ERE is the estrogen response element (5′-GGTCAnnnTGACC-3′) and the 13-nt HRE is the hormone response element (5′-GAACAnnnTGTTC-3′). The 0-nt to 5-nt variant ERE or HRE DNA elements include the 1 0-nt variant consensus palindromic DNA element, 30 1-nt variant DNA elements (10 variant positions), 405 2-nt variant DNA elements (45 variant positions), 3,240 3-nt variant DNA elements (120 variant positions), 17,010 4-nt variant DNA elements (210 variant positions), and 61,236 5-nt variant DNA elements (252 variant positions), for a total of 81,922 DNA elements.

The number of 0-nt to 6-nt variants of the 15-nt ERE and HRE consensus palindromic DNA element on the positive/sense strand in the mouse (mm10) and human (hg19) genome. Here the 15-nt ERE is the estrogen response element (5′-AGGTCAnnnTGACCT-3′) and the 15-nt HRE is the hormone response element (5′-AGAACAnnnTGTTCT-3′). The 0-nt to 6-nt variant 15-nt ERE or HRE DNA elements include the 1 0-nt variant consensus palindromic DNA element, 36 1-nt variant DNA elements (12 variant positions), 594 2-nt variant DNA elements (66 variant positions), 5,940 3-nt variant DNA elements (220 variant positions), 40,095 4-nt variant DNA elements (495 variant positions), 192,456 5-nt variant DNA elements (792 variant positions), and 673,596 6-nt variant DNA elements (924 variant positions), for a total of 912,718 DNA elements.

The number of 0-nt to 5-nt variants of the 10-nt p53RE consensus palindromic DNA element on the positive/sense strand in the mouse (mm10) genome. Here the 10-nt p53RE is the p53 response element (5′-TGCCCGGGCA-3′). The 0-nt to 5-nt variant p53RE DNA elements include the 1 0-nt variant consensus palindromic DNA element, 30 1-nt variant DNA elements (10 variant positions), 405 2-nt variant DNA elements (45 variant positions), 3,240 3-nt variant DNA elements (120 variant positions), 17,010 4-nt variant DNA elements (210 variant positions), and 61,236 5-nt variant DNA elements (252 variant positions), for a total of 81,922 DNA elements.

Table S55. Number of 0-nt to 5-nt Variant 13-nt ERE DNA Elements in the Mouse Genome (81,922)

- Population count of the 81,922 0-nt to 5-nt variant 13-nt EREs in the mouse (mm10) genome [=177,963,712]
- The 0-nt to 5-nt variant 13-nt EREs include:
  - 1 0-nt variant consensus palindromic ERE
  - 30 1-nt variant EREs (10 variant positions)
  - 405 2-nt variant EREs (45 variant positions)
  - 3,240 3-nt variant EREs (120 variant positions)
  - 17,010 4-nt variant EREs (210 variant positions)
  - 61,236 5-nt variant EREs (252 variant positions)

Table S56. Number of 0-nt to 5-nt Variant 13-nt ERE DNA Elements in the Human Genome (81,922)

- Population count of the 81,922 0-nt to 5-nt variant 13-nt EREs in the human (hg19) genome [=187,996,184]
- The 0-nt to 5-nt variant 13-nt EREs include:
  - 1 0-nt variant consensus palindromic ERE
  - 30 1-nt variant EREs (10 variant positions)
  - 405 2-nt variant EREs (45 variant positions)
  - 3,240 3-nt variant EREs (120 variant positions)
  - 17,010 4-nt variant EREs (210 variant positions)
  - 61,236 5-nt variant EREs (252 variant positions)

Table S57. Number of 0-nt to 5-nt Variant 13-nt HRE DNA Elements in the Mouse Genome (81,922)

- Population count of the 81,922 0-nt to 5-nt variant 13-nt HREs in the mouse (mm10) genome [=230,731,128]
- The 0-nt to 5-nt variant 13-nt HREs include:
  - 1 0-nt variant consensus palindromic HRE
  - 30 1-nt variant HREs (10 variant positions)
  - 405 2-nt variant HREs (45 variant positions)
  - 3,240 3-nt variant HREs (120 variant positions)
  - 17,010 4-nt variant HREs (210 variant positions)
  - 61,236 5-nt variant HREs (252 variant positions)

Table S58. Number of 0-nt to 5-nt Variant 13-nt HRE DNA Elements in the Human Genome (81,922)

- Population count of the 81,922 0-nt to 5-nt variant 13-nt HREs in the human (hg19) genome [=253,825,466]
- The 0-nt to 5-nt variant 13-nt HREs include:
  - 1 0-nt variant consensus palindromic HRE
  - 30 1-nt variant HREs (10 variant positions)
  - 405 2-nt variant HREs (45 variant positions)
  - 3,240 3-nt variant HREs (120 variant positions)
  - 17,010 4-nt variant HREs (210 variant positions)

- 61,236 5-nt variant HREs (252 variant positions)

*****

Table S59. Number of 0-nt to 6-nt Variant 15-nt ERE DNA Elements in the Mouse Genome (912,718)

- Population count of the 912,718 0-nt to 6-nt variant 15-nt EREs in the mouse (mm10) genome [=143,500,605]
- The 0-nt to 6-nt variant 15-nt EREs include:
    - 1 0-nt variant consensus palindromic ERE
    - 36 1-nt variant EREs (12 variant positions)
    - 594 2-nt variant EREs (66 variant positions)
    - 5,940 3-nt variant EREs (220 variant positions)
    - 40,095 4-nt variant EREs (495 variant positions)
    - 192,456 5-nt variant EREs (792 variant positions)
    - 673,596 6-nt variant EREs (924 variant positions)

Table S60. Number of 0-nt to 6-nt Variant 15-nt ERE DNA Elements in the Human Genome (912,718)

- Population count of the 912,718 0-nt to 6-nt variant 15-nt EREs in the human (hg19) genome [=154,794,932]
- The 0-nt to 6-nt variant 15-nt EREs include:
    - 1 0-nt variant consensus palindromic ERE
    - 36 1-nt variant EREs (12 variant positions)
    - 594 2-nt variant EREs (66 variant positions)
    - 5,940 3-nt variant EREs (220 variant positions)
    - 40,095 4-nt variant EREs (495 variant positions)
    - 192,456 5-nt variant EREs (792 variant positions)
    - 673,596 6-nt variant EREs (924 variant positions)

Table S61. Number of 0-nt to 6-nt Variant 15-nt HRE DNA Elements in the Mouse Genome (912,718)

- Population count of the 912,718 0-nt to 6-nt variant 15-nt HREs in the mouse (mm10) genome [=191,056,970]
- The 0-nt to 6-nt variant 15-nt HREs include:
    - 1 0-nt variant consensus palindromic HRE
    - 36 1-nt variant HREs (12 variant positions)
    - 594 2-nt variant HREs (66 variant positions)
    - 5,940 3-nt variant HREs (220 variant positions)
    - 40,095 4-nt variant HREs (495 variant positions)
    - 192,456 5-nt variant HREs (792 variant positions)
    - 673,596 6-nt variant HREs (924 variant positions)

Table S62. Number of 0-nt to 6-nt Variant 15-nt HRE DNA Elements in the Human Genome (912,718)

- Population count of the 912,718 0-nt to 6-nt variant 15-nt HREs in the human (hg19) genome [=212,963,770]
- The 0-nt to 6-nt variant 15-nt HREs include:
    - 1 0-nt variant consensus palindromic HRE
    - 36 1-nt variant HREs (12 variant positions)
    - 594 2-nt variant HREs (66 variant positions)
    - 5,940 3-nt variant HREs (220 variant positions)
    - 40,095 4-nt variant HREs (495 variant positions)
    - 192,456 5-nt variant HREs (792 variant positions)
    - 673,596 6-nt variant HREs (924 variant positions)

*****

Table S63. Number of 0-nt to 5-nt Variant 10-nt p53RE DNA Elements in the Mouse Genome (81,922)

- Population count of the 81,922 0-nt to 5-nt variant p53REs in the mouse (mm10) genome [=149,248,264]
- The 0-nt to 5-nt variant 10-nt p53REs include:
    - 1 0-nt variant consensus palindromic p53RE
    - 30 1-nt variant p53REs (10 variant positions)
    - 405 2-nt variant p53REs (45 variant positions)
    - 3,240 3-nt variant p53REs (120 variant positions)
    - 17,010 4-nt variant p53REs (210 variant positions)
    - 61,236 5-nt variant p53REs (252 variant positions)

Table S64-S71. Data Analysis Example (WT-E2-1hr.L4)

Step-by-step instructions of the data analysis from peak selection to (S/N) analysis of ER DNA-binding at 81,922 0-nt to 5-nt variant 13-nt EREs and at 912,718 0-nt to 6-nt variant 15-nt EREs in the genome (displayed by 252 half-site groups and 924 half-site groups)

Table S64. Count 0-nt to 5-nt Variant 13-nt EREs in ER ChIPSeq Peaks (ChIPSeq Peak)

- The experiment WT-E2-1hr.L4 contains 76,163 ChIPSeq peaks (146-nt peak length)
  - 1,201 peaks contain a 0-nt variant consensus palindromic ERE
  - 7,349 peaks contain a 1-nt variant ERE
  - 16,368 peaks contain a 2-nt variant ERE
  - 34,605 peaks contain a 3-nt variant ERE
  - 68,484 peaks contain a 4-nt variant ERE
  - 76,147 peaks contain a 5-nt variant ERE
- These 76,163 peaks contain 827,919 0-nt to 5-nt variant 13-nt EREs
- Listed by ChIPSeq peak

Table S65. Count 0-nt to 5-nt Variant 13-nt EREs in ER ChIPSeq Peaks (Variant Position)

- The experiment WT-E2-1hr.L4 contains 76,163 ChIPSeq peaks (146-nt peak length)
- These 76,163 peaks contain 827,919 0-nt to 5-nt variant 13-nt EREs
- Listed by variant position

Table S66. Categorize 0-nt to 5-nt Variant 13-nt EREs into 252 Half-Site Groups

- Categorize the 827,919 0-nt to 5-nt variant 13-nt EREs into the 252 half-site groups
- For example, the half-site group 1-2-3-4-5 contains all 1-nt variant EREs that vary positions 6 through 10 (i.e., 6A, 6C, 6G, 7A, 7C, 7T, 8C, 8G, 8T, 9A, 9G, 9T, 10A, 10G, 10T), totaling 3,724
- To convert this count to a (S/N) value, multiply the 76,163 peaks by the 146-nt peak length (=11,119,798 nucleotides), and divide 11,119,798 nucleotides by 4^{10} (i.e., the probability that any 10-nt DNA element that has a maximum possibility of 4 nucleotides in each position will occur in the genome is once every 1,048,576 nucleotides (4^{10}) at random occurrence), which yields an expected noise signal of 10.60
- There are 15 (1-nt variant DNA elements) in each 252 half-site group, thus 10.60 x 15 =159.07 expected noise signal
- The DNA element occurrence (3,724), divided by the expected noise (159.07), gives a (S/N) value of 23.41, which is displayed on the 252 half-site plots

Table S67. ER DNA-Binding at Non-NRFE Sites is Independent of NRFE-Containing ChIPSeq Peaks (13-nt ERE)

- The DNA-binding structure observed at non-NRFE DNA elements in the genome is independent of the binding peaks that contain an NRFE

- (S/N) value for all 827,919 0-nt to 5-nt variant EREs:
  - ER DNA-binding at the 0-nt variant consensus palindromic ERE =113.35
  - ER DNA-binding at 1-nt variant EREs =23.47
  - ER DNA-binding at 2-nt variant EREs =4.20
  - ER DNA-binding at 3-nt variant EREs =1.36
  - ER DNA-binding at 4-nt variant EREs =0.96
  - ER DNA-binding at 5-nt variant EREs =0.90

- (S/N) value for all 827,919 0-nt to 5-nt variant EREs (excluding peaks that have already been assigned to an ERE DNA element with less variants):
  - 1,201 peaks contain a 0-nt variant consensus palindromic ERE =7188.01
  - 7,332 peaks contain a 1-nt variant ERE =243.22
  - 15,490 peaks contain a 2-nt variant ERE =19.53
  - 24,374 peaks contain a 3-nt variant ERE =3.02
  - 25,194 peaks contain a 4-nt variant ERE =1.08
  - 2,599 peaks contain a 5-nt variant ERE =0.96

- (S/N) value for all other ERE DNA elements found within these groups
  - Of the 1,201 peaks that contain a 0-nt variant consensus palindromic ERE =7188.01
    - 17 of them also contain a 1-nt variant ERE =3.59
    - 88 of them also contain a 2-nt variant ERE =1.34
    - 419 of them also contain a 3-nt variant ERE =0.98
    - 1,055 of them also contain a 4-nt variant ERE =0.83
    - 1,201 of them also contain a 5-nt variant ERE =0.85

Table S68. Count 0-nt to 6-nt Variant 15-nt EREs in ER ChIPSeq Peaks (ChIPSeq Peak)

- The experiment WT-E2-1hr.L4 contains 76,163 ChIPSeq peaks (146-nt peak length)
  - 304 peaks contain a 0-nt variant consensus palindromic ERE
  - 2,707 peaks contain a 1-nt variant ERE
  - 7,912 peaks contain a 2-nt variant ERE
  - 15,311 peaks contain a 3-nt variant ERE
  - 31,661 peaks contain a 4-nt variant ERE
  - 62,903 peaks contain a 5-nt variant ERE
  - 75,891 peaks contain a 6-nt variant ERE
- These 76,163 peaks contain 605,166 0-nt to 6-nt variant 15-nt EREs
- Listed by ChIPSeq peak

Table S69. Count 0-nt to 6-nt Variant 15-nt EREs in ER ChIPSeq Peaks (Variant Position)

- The experiment WT-E2-1hr.L4 contains 76,163 ChIPSeq peaks (146-nt peak length)
- These 76,163 peaks contain 605,166 0-nt to 6-nt variant 15-nt EREs
- Listed by variant position

Table S70. Categorize 0-nt to 6-nt Variant 15-nt EREs into 924 Half-Site Groups

- Categorize the 605,166 0-nt to 6-nt variant 15-nt EREs into the 924 half-site groups
- For example, the half-site group 1-2-3-4-5-6 contains all 1-nt variant ERE DNA elements that vary positions 7 through 12 (i.e., 7A, 7C, 7G, 8A, 8C, 8T, 9C, 9G, 9T, 10A, 10G, 10T, 11A, 11G, 11T, 12A, 12C, 12G), totaling 1,320
- To convert this count to a (S/N) value, multiply the 76,163 peaks by the 146-nt peak length (=11,119,798 nucleotides), and divide 11,119,798 nucleotides by 4^{12} (i.e., the probability that any 12-nt DNA element that has a maximum possibility of 4 nucleotides in each position will occur in the genome is once every 16,777,216 nucleotides (4^{12}) at random occurrence), which yields an expected noise signal of 0.66
- There are 18 (1-nt variant DNA elements) in each 252 half-site group, thus 0.66 x 18 =11.93 expected noise signal
- The DNA element occurrence (1,320), divided by the expected noise (11.93), gives a (S/N) value of 110.64, which is displayed on the 924 half-site plots

Table S71. ER DNA-Binding at Non-NRFE Sites is Independent of NRFE-Containing ChIPSeq Peaks (15-nt ERE)

- The DNA-binding structure observed at non-NRFE DNA elements in the genome is independent of the binding peaks that contain an NRFE

- (S/N) value for all 605,166 0-nt to 6-nt variant EREs:
  - ER DNA-binding at the 0-nt variant consensus palindromic ERE =460.17
  - ER DNA-binding at 1-nt variant EREs =113.91
  - ER DNA-binding at 2-nt variant EREs =20.59
  - ER DNA-binding at 3-nt variant EREs =4.28
  - ER DNA-binding at 4-nt variant EREs =1.56
  - ER DNA-binding at 5-nt variant EREs =1.03
  - ER DNA-binding at 6-nt variant EREs =0.91

- (S/N) value for all 605,166 0-nt to 6-nt variant EREs (excluding peaks that have already been assigned to an ERE DNA element with less variants):
  - 304 peaks contain a 0-nt variant consensus palindromic ERE =115290.44
  - 2,705 peaks contain a 1-nt variant ERE =3204.99
  - 7,840 peaks contain a 2-nt variant ERE =198.09
  - 13,922 peaks contain a 3-nt variant ERE =21.35
  - 21,585 peaks contain a 4-nt variant ERE =3.78
  - 24,479 peaks contain a 5-nt variant ERE =1.25
  - 5,308 peaks contain a 6-nt variant ERE =0.96

- (S/N) value for all other ERE DNA elements found within these groups
  - Of the 304 peaks that contain a 0-nt variant consensus palindromic ERE =115290.44
    - 2 of them also contain a 1-nt variant ERE =21.00
    - 2 of them also contain a 2-nt variant ERE =1.27
    - 28 of them also contain a 3-nt variant ERE =1.78
    - 120 of them also contain a 4-nt variant ERE =1.43
    - 241 of them also contain a 5-nt variant ERE =0.97
    - 304 of them also contain a 6-nt variant ERE =0.92

Table S72-S74. Genome Assembly of the Mouse and Human Genome

Table S72. Genome Assembly (Chromosome Size) of the Mouse and Human Genome

- The mouse and human genome assemblies were obtained from UCSC Genome Browser
    - mouse genome build 38 (mm10)
    - human genome build 37 (hg19)
- The genome size of the mouse (mm10) genome is 2,730,871,774 nucleotides
- The genome size of the human (hg19) genome is 3,137,161,264 nucleotides

Table S73. Genome Assembly (Effective Size) of the Mouse and Human Genome

- The effective genome size of the mouse (mm10) genome is 2,647,537,730 nucleotides when excluding unreadable nucleotides, random regions, hap regions, and chromosome Un (i.e., clone contigs that cannot be confidently placed on a specific chromosome) from the reference
- The effective genome size of the human (hg19) genome is 2,861,343,702 nucleotides when excluding unreadable nucleotides, random regions, hap regions, and chromosome Un (i.e., clone contigs that cannot be confidently placed on a specific chromosome) from the reference

Table S74. Genome Assembly (N Islands) of the Mouse and Human Genome

- The unreadable nucleotides account for 2.9% [77,999,939] of the mouse (mm10) genome
- The unreadable nucleotides account for 7.5% [234,350,281] of the human (hg19) of the genome
- These unreadable nucleotides are contained within 501 unreadable regions in the mouse (mm10) genome
- These unreadable nucleotides are contained within 339 unreadable regions in the human (hg19) genome

Table S75. Script Codes for All Data Analyses

- Script codes for data analysis of sNR DNA-binding at 0-nt to 5-nt variant DNA elements in the genome
  - motif_positions
  - 258058.ERE.txt
  - 258058.HRE.txt
- Script codes for genome assembly of the mouse (mm10) and human (hg19) genomes
  - linreg.awk
  - get_error_terms_new

Datafile S1-S5 Descriptions: Compressed Datafiles

Datafile S1. ChIPSeq and ChIPExo Experiments

- 157 ER experiments
- 194 KR experiments
- 8 GR and GR-Dim experiments
- Peak selection criteria =L4, L8, L10, L15, L20
- doi:10.17632/8ks7pm2jw6.1 (Mendeley)

Datafile S2. Population Count of 1-nt to 20-nt DNA Elements in the Single-Stranded Mouse Genome [1.4 T]

- The population count of every 1-nt to 20-nt DNA elements in the single-stranded mouse (mm10) genome, including its reverse-complement DNA element, reverse DNA element, and complement DNA element
- Total =20 files (1-nt to 20-nt DNA elements)
- tar -xvzf *filename*.tar.gz to unzip files

Datafile S3. Population Count of 1-nt to 20-nt DNA Elements in the Single-Stranded Human Genome [1.4 T]

- The population count of every 1-nt to 20-nt DNA elements in the single-stranded human (hg19) genome, including its reverse-complement DNA element, reverse DNA element, and complement DNA element
- Total =20 files (1-nt to 20-nt DNA elements)
- tar -xvzf *filename*.tar.gz to unzip files

Datafile S4. Population Count of 1-nt to 20-nt DNA Elements in the Single-Stranded Mouse Genome (Chromosome)

- The population count of every 1-nt to 20-nt DNA elements (by chromosome) in the single-stranded mouse (mm10) genome, including its reverse-complement DNA element, reverse DNA element, and complement DNA element
- Total =440 files (1-nt to 20-nt DNA elements x 22)
    - 19 chromosomes
    - chromosome M
    - chromosome X
    - chromosome Y
- tar -xvzf *filename*.tar.gz to unzip files

Datafile S5. Population Count of 1-nt to 20-nt DNA Elements in the Single-Stranded Human Genome (Chromosome)

- The population count of every 1-nt to 20-nt DNA elements (by chromosome) in the single-stranded human (hg19) genome, including its reverse-complement DNA element, reverse DNA element, and complement DNA element
- Total =500 files (1-nt to 20-nt DNA elements x 25)
    - 22 chromosomes
    - chromosome M
    - chromosome X
    - chromosome Y
- tar -xvzf *filename*.tar.gz to unzip files

Transparent Methods

Quality Control and Peak Selection Criteria

In total, 381 ChIPSeq and ChIPExo experiments were obtained from Gene Expression Omnibus (GEO) and converted to fastq format using fastq-dump v2.4.5; original experiment names were retained.

Reads were first selected using a cross-correlation analysis (trim_and_filter_SE.pl) (5).

The sequencing reads were then mapped uniquely, allowing for no more than two mismatches, to the reference genome [mouse genome build 38 (mm10) or human genome build 37 (hg19)] using Bowtie v1.1.2 (6).

Mapped reads were deduplicated using MarkDuplicates.jar from the Picard tools package v1.96.

Peak selection was performed using Hypergeometric Optimization of Motif Enrichment (HOMER) v4.7.2. Multiple peak selection criteria were used (L4, L8, L10, L15, L20), where Lx represents an x-fold greater tag density at peaks than in the surrounding 10-kb region. This performs a low-to-high stringency analysis of the data. By studying the data with respect to a multiple spectrum of peak selection criteria, we adjust for both the risk of excess background noise and the risk of filtering out any low-amplitude information.

See our previously published study for the associated script codes and data analyses for these experiments (2).

Number and Location Coordinates of DNA Elements (ERE, HRE, p53RE) in the Mouse and Human Genome

Number and Location Coordinates of 0-nt to 5-nt Variant 13-nt ERE and HRE DNA Elements in the Genome
The number and location coordinates of the 81,922 0-nt to 5-nt variants of the 13-nt ERE and HRE consensus palindromic DNA element on the positive/sense strand were identified in the mouse (mm10) and human (hg19) genome using OligoMatch (University of California, Santa Cruz). Here the 13-nt ERE is the estrogen response element (5′-GGTCAnnnTGACC-3′) and the 13-nt HRE is the hormone response element (5′-GAACAnnnTGTTC-3′). The 0-nt to 5-nt variant 13-nt ERE and HRE DNA elements include the 1 0-nt variant consensus palindromic DNA element, 30 1-nt variant DNA elements (10 variant positions), 405 2-nt variant DNA elements (45 variant positions), 3,240 3-nt variant DNA elements (120 variant positions), 17,010 4-nt variant DNA elements (210 variant positions), and 61,236 5-nt variant DNA elements (252 variant positions), for a total of 81,922 DNA elements. See our previously published study for the associated script codes (2). The population count of each of the 81,922 0-nt to 5-nt variant 13-nt ERE and HRE DNA elements in the mouse (mm10) and human (hg19) genome can be found in **Table S55-S58**.

Four nucleotide possibilities in a DNA element with 10 primary positions:

n = number of primary positions in the DNA element = **10**

L = number of nucleotide possibilities = **4**

k = number of variants

Binomial Equation:

$$(x+y)^n = \sum_{k=0}^{n} \binom{n}{k} x^k y^{n-k}$$

Combinations:

$$L^n = [(L-1)+1]^n = \sum_{k=0}^{n} \binom{n}{k} (L-1)^k \, (1)^{n-k}$$

$$= \sum_{k=0}^{n} \binom{n}{k} (L-1)^k = 2^{20} = 4^{10}$$

	DNA Element	Combinatorial Counts				DNA Element Frequency in Genome			
**A**	**B**	**C**	**D**	**E**	**F**	**Mouse Genome (mm10)**		**Human Genome (hg19)**	
k	**13-nt ERE** **GGTCAnnnTGACC**	**Combinations** **C =** $\binom{n}{k}$	**4-nts** **D = C x 4^k**	**Unique** **E = C x 3^k**	**Total Unique** **F = ΣE**	**Unique**	**Total Unique**	**Unique**	**Total Unique**
**0**	0-nt Variant ERE	1	1	1	1	2,367		2,194	
**1**	1-nt Variant ERE	10	40	30	31	71,428	73,795	60,313	62,507
**2**	2-nt Variant ERE	45	720	405	436	898,155	971,950	914,726	977,233
**3**	3-nt Variant ERE	120	7,680	3,240	3,676	6,750,607	7,722,557	7,516,184	8,493,417
**4**	4-nt Variant ERE	210	53,760	17,010	20,686	35,508,190	43,230,747	38,222,674	46,716,091
**5**	5-nt Variant ERE	252	258,048	61,236	81,922	134,732,965	177,963,712	141,280,093	187,996,184

**A**	**B**	**C**	**D**	**E**	**F**	**Mouse Genome (mm10)**		**Human Genome (hg19)**	
k	**13-nt HRE** **GAACAnnnTGTTC**	**Combinations** **C =** $\binom{n}{k}$	**4-nts** **D = C x 4^k**	**Unique** **E = C x 3^k**	**Total Unique** **F = ΣE**	**Unique**	**Total Unique**	**Unique**	**Total Unique**
**0**	0-nt Variant HRE	1	1	1	1	3,444		3,535	
**1**	1-nt Variant HRE	10	40	30	31	97,039	100,483	104,767	108,302
**2**	2-nt Variant HRE	45	720	405	436	1,337,516	1,437,999	1,339,543	1,447,845
**3**	3-nt Variant HRE	120	7,680	3,240	3,676	10,461,197	11,899,196	10,771,159	12,219,004
**4**	4-nt Variant HRE	210	53,760	17,010	20,686	49,391,434	61,290,630	54,136,564	66,355,568
**5**	5-nt Variant HRE	252	258,048	61,236	81,922	169,440,498	230,731,128	187,469,898	253,825,466

**A**	**B**	**C**	**D**	**E**	**F**
k		**Combinations** **C =** $\binom{n}{k}$	**4-nts** **D = C x 4^k**	**Unique** **E = C x 3^k**	**Total Unique** **F = ΣE**
**0**	0-nt Variant	10!/(0! x 10!)	1 x 1	1 x 1	1 + 0
**1**	1-nt Variant	10!/(1! x 9!)	10 x 4	10 x 3	30 + 1
**2**	2-nt Variant	10!/(2! x 8!)	45 x 16	45 x 9	405 + 31
**3**	3-nt Variant	10!/(3! x 7!)	120 x 64	120 x 27	3,240 + 436
**4**	4-nt Variant	10!/(4! x 6!)	210 x 256	210 x 81	17,010 +3,676
**5**	5-nt Variant	10!/(5! x 5!)	252 x 1,024	252 x 243	61,236 + 20,686

Number and Location Coordinates of 0-nt to 6-nt Variant 15-nt ERE and HRE DNA Elements in the Genome

The number and location coordinates of the 912,718 0-nt to 6-nt variants of the 15-nt ERE and HRE consensus palindromic DNA element on the positive/sense strand were identified in the mouse (mm10) and human (hg19) genome using OligoMatch (University of California, Santa Cruz). Here the 15-nt ERE is the estrogen response element (5′-AGGTCAnnnTGACCT-3′) and the 13-nt HRE is the hormone response element (5′-AGAACAnnnTGTTCT-3′). The 0-nt to 6-nt variant 15-nt ERE and HRE DNA elements include the 1 0-nt variant consensus palindromic DNA element, 36 1-nt variant DNA elements (12 variant positions), 594 2-nt variant DNA elements (66 variant positions), 5,940 3-nt variant DNA elements (220 variant positions), 40,095 4-nt variant DNA elements (495 variant positions), 192,456 5-nt variant DNA elements (792 variant positions), and 673,596 6-nt variant DNA elements (924 variant positions), for a total of 912,718 DNA elements. See our previously published study for the associated script codes (2). The population count of each of the 912,718 0-nt to 6-nt variant 15-nt ERE and HRE DNA elements in the mouse (mm10) and human (hg19) genome can be found in **Table S59-S62**.

Four nucleotide possibilities in a DNA element with 12 primary positions:

n = number of primary positions in the DNA element = **12**

L = number of nucleotide possibilities = **4**

k = number of variants

Binomial Equation:

$$(x+y)^n = \sum_{k=0}^{n} \binom{n}{k} x^k y^{n-k}$$

Combinations:

$$L^n = [(L-1)+1]^n = \sum_{k=0}^{n} \binom{n}{k} (L-1)^k \, (1)^{n-k}$$

$$= \sum_{k=0}^{n} \binom{n}{k} (L-1)^k = 2^{24} = 4^{12}$$

	DNA Element	Combinatorial Counts				DNA Element Frequency in Genome			
A	B	C	D	E	F	Mouse Genome (mm10)		Human Genome (hg19)	
k	15-nt ERE AGGTCAnnnTGACCT	Combinations $C = \binom{n}{k}$	4-nts $D = C \times 4^k$	Unique $E = C \times 3^k$	Total Unique $F = \Sigma E$	Unique	Total Unique	Unique	Total Unique
0	0-nt Variant ERE	1	1	1	1	404		342	
1	1-nt Variant ERE	12	48	36	37	10,964	11,368	9,223	9,565
2	2-nt Variant ERE	66	1056	594	631	152,140	163,508	118,354	127,919
3	3-nt Variant ERE	220	14,080	5,940	6,571	1,256,647	1,420,155	1,213,963	1,341,882
4	4-nt Variant ERE	495	126,720	40,095	46,666	6,916,002	8,336,157	7,349,740	8,691,622
5	5-nt Variant ERE	792	811,008	192,456	239,122	30,575,777	38,911,934	33,052,166	41,743,788
6	6-nt Variant ERE	924	3,784,704	673,596	912,718	104,588,671	143,500,605	113,051,144	154,794,932

A	B	C	D	E	F	Mouse Genome (mm10)		Human Genome (hg19)	
k	15-nt HRE AGAACAnnnTGTTCT	Combinations $C = \binom{n}{k}$	4-nts $D = C \times 4^k$	Unique $E = C \times 3^k$	Total Unique $F = \Sigma E$	Unique	Total Unique	Unique	Total Unique
0	0-nt Variant HRE	1	1	1	1	639		565	
1	1-nt Variant HRE	12	48	36	37	17,847	18,486	14,639	15,204
2	2-nt Variant HRE	66	1056	594	631	256,540	275,026	206,983	222,187
3	3-nt Variant HRE	220	14,080	5,940	6,571	2,188,277	2,463,303	1,978,470	2,200,657
4	4-nt Variant HRE	495	126,720	40,095	46,666	11,128,133	13,591,436	11,661,975	13,862,632
5	5-nt Variant HRE	792	811,008	192,456	239,122	43,400,607	56,992,043	48,017,520	61,880,152
6	6-nt Variant HRE	924	3,784,704	673,596	912,718	134,064,927	191,056,970	151,083,618	212,963,770

A	B	C	D	E	F
k		Combinations $C = \binom{n}{k}$	4-nts $D = C \times 4^k$	Unique $E = C \times 3^k$	Total Unique $F = \Sigma E$
0	0-nt Variant	12!/(0! x 12!)	1 x 1	1 x 1	1 + 0
1	1-nt Variant	12!/(1! x 11!)	12 x 4	12 x 3	36 + 1
2	2-nt Variant	12!/(2! x 10!)	66 x 16	66 x 9	594 + 37
3	3-nt Variant	12!/(3! x 9!)	220 x 64	220 x 27	5,940 + 631
4	4-nt Variant	12!/(4! x 8!)	495 x 256	495 x 81	40,095 + 6,571
5	5-nt Variant	12!/(5! x 7!)	792 x 1,024	792 x 243	192,456 + 46,666
6	6-nt Variant	12!/(6! x 6!)	924 x 4,096	924 x 729	673,596 + 239,122

Number and Location Coordinates of 0-nt to 5-nt Variant 10-nt p53RE DNA Elements in the Genome

The number and location coordinates of the 81,922 0-nt to 5-nt variant of the 10-nt p53RE consensus palindromic DNA element on the positive/sense strand were identified in the mouse (mm10) genome using OligoMatch (University of California, Santa Cruz). Here the 10-nt p53RE is the p53 response element (5′-TGCCCGGGCA-3′). The 0-nt to 5-nt variant 10-nt p53RE DNA elements include the 1 0-nt variant consensus palindromic DNA element, 30 1-nt variant DNA elements (10 variant positions), 405 2-nt variant DNA elements (45 variant positions), 3,240 3-nt variant DNA elements (120 variant positions), 17,010 4-nt variant DNA elements (210 variant positions), and 61,236 5-nt variant DNA elements (252 variant positions), for a total of 81,922 DNA elements. See our previously published study for the associated script codes (2). The population count of each of the 81,922 0-nt to 5-nt variant 10-nt p53RE DNA elements in the mouse (mm10) genome can be found in **Table S63**.

Four nucleotide possibilities in a DNA element with 10 primary positions:

n = number of primary positions in the DNA element = **10**

L = number of nucleotide possibilities = **4**

k = number of variants

Binomial Equation:

$$(x + y)^n = \sum_{k=0}^{n} \binom{n}{k} x^k y^{n-k}$$

Combinations:

$$L^n = [(L-1)+1]^n = \sum_{k=0}^{n} \binom{n}{k} (L-1)^k \, (1)^{n-k}$$

$$= \sum_{k=0}^{n} \binom{n}{k} (L-1)^k = 2^{20} = 4^{10}$$

	DNA Element	Combinatorial Counts				DNA Element Frequency in Genome	
**A**	**B**	**C**	**D**	**E**	**F**	**Mouse Genome (mm10)**	
k	**P53RE** TGCCCGGGCA	**Combinations** C = $\binom{n}{k}$	**4-nts** D = C x 4^k	**Unique** E = C x 3^k	**Total Unique** F = ΣE	**Unique**	**Total Unique**
**0**	0-nt Variant p53RE	1	1	1	1	1,078	
**1**	1-nt Variant p53RE	10	40	30	31	33,992	35,070
**2**	2-nt Variant p53RE	45	720	405	436	641,422	676,492
**3**	3-nt Variant p53RE	120	7,680	3,240	3,676	5,380,457	6,056,949
**4**	4-nt Variant p53RE	210	53,760	17,010	20,686	29,257,379	35,314,328
**5**	5-nt Variant p53RE	252	258,048	61,236	81,922	113,933,936	149,248,264

**A**	**B**	**C**	**D**	**E**	**F**
k		**Combinations** C = $\binom{n}{k}$	**4-nts** D = C x 4^k	**Unique** E = C x 3^k	**Total Unique** F = ΣE
**0**	0-nt Variant	10!/(0! x 10!)	1 x 1	1 x 1	1 + 0
**1**	1-nt Variant	10!/(1! x 9!)	10 x 4	10 x 3	30 + 1
**2**	2-nt Variant	10!/(2! x 8!)	45 x 16	45 x 9	405 + 31
**3**	3-nt Variant	10!/(3! x 7!)	120 x 64	120 x 27	3,240 + 436
**4**	4-nt Variant	10!/(4! x 6!)	210 x 256	210 x 81	17,010 +3,676
**5**	5-nt Variant	10!/(5! x 5!)	252 x 1,024	252 x 243	61,236 + 20,686

Overlap DNA Elements (ERE, HRE, p53RE) in the Genome with ChIPSeq and ChIPExo Experiments

A customized C++ program, cppmatch_custom_full.cpp, was used to overlap location coordinates of each 0-nt to 5-nt/6-nt variant ERE, HRE, or p53RE DNA element in the genome and the location coordinates of the ChIPSeq or ChIPExo peaks in an experiment to determine the absolute number of times each 0-nt to 5-nt/6-nt variant ERE, HRE, or p53RE DNA element occurred within an experiment (the entire 10-nt, 13-nt or 15-nt DNA element was required to be within the peak boundaries).

See our previously published study for the associated script codes (2).

The complete experiments, including individual peaks and 0-nt to 5-nt variant overlaps, have also been provided (tar -xvzf *filename*.tar.gz to unzip files) **(Datafile S1)**.

Convert 0-nt to 5-nt/6-nt Variant DNA Elements to Variant Positions

Convert 0-nt to 5-nt Variant 13-nt ERE and HRE DNA Elements to Variant Positions

To evaluate the DNA-binding of sNRs at every DNA element in the 0-nt to 5-nt variant groups, the absolute number of times each of the 81,922 0-nt to 5-nt variant 13-nt ERE or HRE DNA elements occurred in an experiment was counted.

The 0-nt variant consensus palindromic 13-nt ERE DNA element (5′-GGTCAnnnTGACC-3′) and 0-nt variant consensus palindromic 13-nt HRE DNA element (5′-GAACAnnnTGTTC-3′) have ten (10) primary positions.

The 0-nt to 5-nt variant ERE or HRE DNA elements include the 1 0-nt variant consensus palindromic DNA element, 30 1-nt variant DNA elements (10 variant positions), 405 2-nt variant DNA elements (45 variant positions), 3,240 3-nt variant DNA elements (120 variant positions), 17,010 4-nt variant DNA elements (210 variant positions), and 61,236 5-nt variant DNA elements (252 variant positions), for a total of 81,922 DNA elements.

See **Table S75** for script codes to convert the counts of all overlapped 0-nt to 5-nt DNA elements to variant positions.

13-nt ERE = GGTCAnnnTGACC

1 2 3 4 5 6 7 8 9 10

13-nt HRE = GAACAnnnTGTTC

1 2 3 4 5 6 7 8 9 10

13-nt ERE and HRE DNA Elements						
A	B	C	D	E	F	G
	DNA Element	Variant Position Combinations		Palindromic Position Pairs (PP)	Same-Side Variants	Crossover Variants
k	13-nt ERE or HRE (***n***=10)	$C = \binom{n}{k}$	$D = \binom{n}{k}$			
**0**	0-nt Variant ERE or HRE	10!/(0! x 10!)	1			
**1**	1-nt Variant ERE or HRE	10!/(1! x 9!)	10			
**2**	2-nt Variant ERE or HRE	10!/(2! x 8!)	45	5	20	20
**3**	3-nt Variant ERE or HRE	10!/(3! x 7!)	120		20	100
**4**	4-nt Variant ERE or HRE	10!/(4! x 6!)	210	10	10	190
**5**	5-nt Variant ERE or HRE	10!/(5! x 5!)	252		2	250

	Palindromic Position Pairs (PP)			
	13-nt ERE and HRE DNA Elements			
	**2-nt ERE**	**2-nt HRE**	**4-nt ERE**	**4-nt HRE**
**1**	3-8	2-9	1-3-8-10	2-5-6-9
**2**	1-10	5-6	3-5-6-8	1-2-9-10
**3**	5-6	1-10	3-4-7-8	2-4-7-9
**4**	4-7	4-7	2-3-8-9	2-3-8-9
**5**	2-9	3-8	1-5-6-10	1-5-6-10
**6**			1-4-7-10	4-5-6-7
**7**			1-2-9-10	3-5-6-8
**8**			4-5-6-7	1-4-7-10
**9**			2-5-6-9	1-3-8-10
**10**			2-4-7-9	3-4-7-8

Convert 0-nt to 6-nt Variant 15-nt ERE and HRE DNA Elements to Variant Positions

To evaluate the DNA-binding of sNRs at every DNA element in the 0-nt to 6-nt variant groups, the absolute number of times each of the 912,718 0-nt to 6-nt variant 15-nt ERE or HRE DNA elements occurred in an experiment was counted.

The 0-nt variant consensus palindromic 15-nt ERE DNA element (5′-AGGTCAnnnTGACCT-3′) and 0-nt variant consensus palindromic 15-nt HRE DNA element (5′-AGAACAnnnTGTTCT-3′) have twelve (12) primary positions.

The 0-nt to 6-nt variant 15-nt ERE or HRE DNA elements include the 1 0-nt variant consensus palindromic DNA element, 36 1-nt variant DNA elements (12 variant positions), 594 2-nt variant DNA elements (66 variant positions), 5,940 3-nt variant DNA elements (220 variant positions), 40,095 4-nt variant DNA elements (495 variant positions), 192,456 5-nt variant DNA elements (792 variant positions), and 673,596 6-nt variant DNA elements (924 variant positions), for a total of 912,718 DNA elements.

See **Table S75** for script codes to convert the counts of all overlapped 0-nt to 6-nt DNA elements to variant positions.

15-nt ERE = AGGTCAnnnTGACCT

1 2 3 4 5 6 7 8 9 10 11 12

15-nt HRE = AGAACAnnnTGTTCT

1 2 3 4 5 6 7 8 9 10 11 12

15-nt ERE and HRE DNA Elements						
A	B	C	D	E	F	G
	DNA Element	Variant Position Combinations		Palindromic Position Pairs (PP)	Same-Side Variants	Crossover Variants
k	15-nt ERE or HRE (***n***=12)	C = $\binom{n}{k}$	D = $\binom{n}{k}$			
**0**	0-nt Variant ERE or HRE	12!/(0! x 12!)	1			
**1**	1-nt Variant ERE or HRE	12!/(1! x 11!)	12			
**2**	2-nt Variant ERE or HRE	12!/(2! x 10!)	66	6	30	30
**3**	3-nt Variant ERE or HRE	12!/(3! x 9!)	220		40	180
**4**	4-nt Variant ERE or HRE	12!/(4! x 8!)	495	15	30	450
**5**	5-nt Variant ERE or HRE	12!/(5! x 7!)	792		12	780
**6**	6-nt Variant ERE or HRE	12!/(6! x 6!)	924	20	2	902

	Palindromic Position Pairs (PP)					
	15-nt ERE and HRE DNA Elements					
	**2-nt ERE**	**2-nt HRE**	**4-nt ERE**	**4-nt HRE**	**6-nt ERE**	**6-nt HRE**
**1**	4-9	3-10	2-4-9-11	3-6-7-10	1-4-6-7-9-12	1-2-3-10-11-12
**2**	2-11	6-7	4-6-7-9	2-3-10-11	2-3-4-9-10-11	3-4-5-8-9-10
**3**	6-7	2-11	4-5-8-9	3-5-8-10	1-3-4-9-10-12	1-3-4-9-10-12
**4**	5-8	5-8	3-4-9-10	3-4-9-10	1-2-4-9-11-12	1-2-4-9-11-12
**5**	3-10	4-9	2-6-7-11	2-6-7-11	2-4-6-7-9-11	2-3-6-7-10-11
**6**	1-12	1-12	2-5-8-11	5-6-7-8	1-4-5-8-9-12	1-3-5-8-10-12
**7**			2-3-10-11	4-6-7-9	2-4-5-8-9-11	3-5-6-7-8-10
**8**			5-6-7-8	2-5-8-11	3-4-6-7-9-10	2-5-6-7-8-11
**9**			3-6-7-10	2-4-9-11	1-2-6-7-11-12	1-2-6-7-11-12
**10**			3-5-8-10	4-5-8-9	4-5-6-7-8-9	4-5-6-7-8-9
**11**			1-4-9-12	1-3-10-12	1-2-5-8-11-12	1-5-6-7-8-12
**12**			1-2-11-12	1-6-7-12	1-3-6-7-10-12	2-3-5-8-10-11
**13**			1-6-7-12	1-2-11-12	1-2-3-10-11-12	1-4-6-7-9-12
**14**			1-5-8-12	1-5-8-12	3-4-5-8-9-10	2-3-4-9-10-11
**15**			1-3-10-12	1-4-9-12	1-5-6-7-8-12	1-2-5-8-11-12
**16**					2-3-5-8-10-11	1-3-6-7-10-12
**17**					2-3-6-7-10-11	2-4-6-7-9-11
**18**					1-3-5-8-10-12	1-4-5-8-9-12
**19**					3-5-6-7-8-10	2-4-5-8-9-11
**20**					2-5-6-7-8-11	3-4-6-7-9-10

Convert 0-nt to 5-nt Variant 10-nt p53RE DNA Elements to Variant Positions

To evaluate the DNA-binding of p53 at every DNA element in the 0-nt to 5-nt variant groups, the absolute number of times each of the 81,922 0-nt to 5-nt variant 10-nt p53RE DNA elements occurred in an experiment was counted.

The 0-nt variant consensus palindromic 10-nt p53RE DNA element (5′-TGCCCGGGCA-3′) has ten (10) primary positions.

The 0-nt to 5-nt variant p53RE DNA elements include the 1 0-nt variant consensus palindromic DNA element, 30 1-nt variant DNA elements (10 variant positions), 405 2-nt variant DNA elements (45 variant positions), 3,240 3-nt variant DNA elements (120 variant positions), 17,010 4-nt variant DNA elements (210 variant positions), and 61,236 5-nt variant DNA elements (252 variant positions), for a total of 81,922 DNA elements.

See **Table S75** for script codes to convert the counts of all overlapped 0-nt to 5-nt DNA elements to variant positions.

10-nt p53RE = TGCCCGGGCA

1 2 3 4 5 6 7 8 9 10

10-nt p53RE DNA Elements						
**A**	**B**	**C**	**D**	**E**	**F**	**G**
	**DNA Element**	**Variant Position Combinations**		**Palindromic Position Pairs (PP)**	**Same-Side Variants**	**Crossover Variants**
k	**P53RE (*n*=10)**	C = $\binom{n}{k}$	D = $\binom{n}{k}$			
**0**	0-nt Variant p53RE	10!/(0! x 10!)	1			
**1**	1-nt Variant p53RE	10!/(1! x 9!)	10			
**2**	2-nt Variant p53RE	10!/(2! x 8!)	45	5	20	20
**3**	3-nt Variant p53RE	10!/(3! x 7!)	120		20	100
**4**	4-nt Variant p53RE	10!/(4! x 6!)	210	10	10	190
**5**	5-nt Variant p53RE	10!/(5! x 5!)	252		2	250

	**Palindromic Position Pairs (PP)**	
	**10-nt p53RE DNA Elements**	
	**2-nt p53RE**	**4-nt p53RE**
**1**	5-6	1-5-6-10
**2**	1-10	3-5-6-8
**3**	3-8	4-5-6-7
**4**	4-7	2-5-6-9
**5**	2-9	1-3-8-10
**6**		1-4-7-10
**7**		1-2-9-10
**8**		3-4-7-8
**9**		2-3-8-9
**10**		2-4-7-9

Categorize 0-nt to 5-nt/6-nt Variant DNA Elements into Half-Site Groups

Categorize 0-nt to 5-nt Variant 13-nt ERE and HRE DNA Elements Into 252 Half-Site Groups

To evaluate the DNA-binding of sNRs at every DNA element in the 0-nt to 5-nt variant groups, the absolute number of times each of the 81,922 0-nt to 5-nt variant DNA elements occurred in an experiment were counted. For display purposes, we have defined a 5-nt variant ERE and HRE DNA element by its five (5) fixed positions, resulting in 252 half-site groups (i.e., categorizing the 61,236 5-nt variant DNA elements into 252 half-site groups, defined by the 5 positions that are fixed/not varied). The remaining 0-nt to 4-nt variant DNA elements (1 0-nt variant consensus palindromic DNA element, 30 1-nt variant DNA elements, 405 2-nt variant DNA elements, 3,240 3-nt variant DNA elements, and 17,010 4-nt variant DNA elements, for a total of 20,686 DNA elements) can be categorized into these same 252 half-site groups (i.e., 5 positions are fixed, allowing for *up to* 5 positions to be varied). For example, the half-site group 1-2-3-4-5 contains all 1-nt variant DNA elements that vary positions 6 through 10. In the case of the ERE, this includes the following 1-nt variant ERE DNA elements: 6A, 6C, 6G, 7A, 7C, 7T, 8C, 8G, 8T, 9A, 9G, 9T, 10A, 10G, 10T. Thus, each of the 252 half-site groups contain 1 (0-nt variant consensus palindromic DNA element), 15 (1-nt variant DNA elements), 90 (2-nt variant DNA elements), 270 (3-nt variant DNA elements), 405 (4-nt variant DNA elements), and 243 (5-nt variant DNA elements), for a total of 1,024 DNA elements per half-site group. This allows all 81,922 0-nt to 5-nt variant DNA elements to be categorized into the 252 half-site groups, and thus providing the ability to sequentially track sNR DNA-binding at all 81,922 0-nt to 5-nt variant ERE or HRE DNA elements in the genome **(Table S1-S4)**.

Of these 252 half-site groups, thirty-two (32) have zero vacancies, one hundred and sixty (160) have one vacancy, and sixty (60) have two vacancies.

See **Table S75** for script codes to convert the counts of all overlapped 0-nt to 5-nt DNA elements into the half-site groups.

Four nucleotide possibilities in a DNA element with 5 primary positions:

n = number of primary positions in the DNA element = **5**

L = number of nucleotide possibilities = **4**

k = number of variants

Binomial Equation:

$$(x+y)^n = \sum_{k=0}^{n} \binom{n}{k} x^k y^{n-k}$$

Combinations:

$$L^n = [(L-1)+1]^n = \sum_{k=0}^{n} \binom{n}{k} (L-1)^k \, (1)^{n-k}$$

$$= \sum_{k=0}^{n} \binom{n}{k} (L-1)^k = 4^5 = 2^{10}$$

	DNA Element	Combinatorial Counts			
A	B	C	D	E	F
k	5-nt Half-Site	Combinations $C = \binom{n}{k}$	4-nts $D = C \times 4^k$	Unique $E = C \times 3^k$	Total Unique $F = \Sigma E$
**0**	0-nt Variant Half-Site	1	1	**1**	1
**1**	1-nt Variant Half-Site	5	20	**15**	16
**2**	2-nt Variant Half-Site	10	160	**90**	106
**3**	3-nt Variant Half-Site	10	640	**270**	376
**4**	4-nt Variant Half-Site	5	1,280	**405**	781
**5**	5-nt Variant Half-Site	1	1,024	**243**	1,024

A	B	C	D	E	F
k	GGTCAnnnTGACC<br>GAACAnnnTGTTC	Combinations $C = \binom{n}{k}$	4-nts $D = C \times 4^k$	Unique $E = C \times 3^k$	Total Unique $F = \Sigma E$
**0**	0-nt Variant	5!/(0! x 5!)	1 x 1	1 x 1	1 + 0
**1**	1-nt Variant	5!/(1! x 4!)	5 x 4	5 x 3	15 + 1
**2**	2-nt Variant	5!/(2! x 3!)	10 x 16	10 x 9	90 + 16
**3**	3-nt Variant	5!/(3! x 2!)	10 x 64	10 x 27	270 + 106
**4**	4-nt Variant	5!/(4! x 1!)	5 x 256	5 x 81	405 + 376
**5**	5-nt Variant	5!/(5! x 0!)	1 x 1,024	1 x 243	243 + 781

Categorize 0-nt to 6-nt Variant 15-nt ERE and HRE DNA Elements Into 924 Half-Site Groups

To evaluate the DNA-binding of sNRs at every DNA element in the 0-nt to 6-nt variant groups, the absolute number of times each of the 912,718 0-nt to 6-nt variant DNA elements occurred in an experiment were counted. For display purposes, we have defined a 6-nt variant ERE and HRE DNA element by its six (6) fixed positions, resulting in 924 half-site groups (i.e., categorizing the 673,596 6-nt variant DNA elements into 924 half-site groups, defined by the 6 positions that are fixed/not varied). The remaining 0-nt to 5-nt variant DNA elements (1 0-nt variant consensus palindromic DNA element, 36 1-nt variant DNA elements, 594 2-nt variant DNA elements, 5,940 3-nt variant DNA elements, and 40,095 4-nt variant DNA elements, 192,456 5-nt variant DNA elements, for a total of 239,122 elements) can be categorized into these same 924 half-site groups (i.e., 6 positions are fixed, allowing for *up to* 6 positions to be varied). For example, the half-site group 1-2-3-4-5-6 contains all 1-nt variant DNA elements that vary positions 7 through 12. In the case of the ERE, this includes the following 1-nt variant ERE DNA elements: 7A, 7C, 7G, 8A, 8C, 8T, 9C, 9G, 9T, 10A, 10G, 10T, 11A, 11G, 11T, 12A, 12C, 12G. Thus, each of the 924 half-site groups contain 1 (0-nt variant consensus palindromic DNA element), 18 (1-nt variant DNA elements), 135 (2-nt variant DNA elements), 540 (3-nt variant DNA elements), 1,215 (4-nt variant DNA elements), 1,458 (5-nt variant DNA elements), and 729 (6-nt variant DNA elements), for a total of 4,096 DNA elements per half-site group. This allows all 912,718 0-nt to 6-nt variant DNA elements to be categorized into the 924 half-site groups, and thus providing the ability to sequentially track sNR DNA-binding at 0-nt to 6-nt variant ERE or HRE DNA elements in the genome **(Table S21-S24)**.

Of these 924 half-site groups, sixty-four (64) have zero vacancies, four hundred and eighty (480) have one vacancy, three hundred and sixty (360) have two vacancies, and twenty (20) have three vacancies.

See **Table S75** for script codes to convert the counts of all overlapped 0-nt to 6-nt DNA elements into the half-site groups.

Four nucleotide possibilities in a DNA element with 6 primary positions:

n = number of primary positions in the DNA element = **6**

L = number of nucleotide possibilities = **4**

k = number of variants

Binomial Equation:

$$(x+y)^n = \sum_{k=0}^{n} \binom{n}{k} x^k y^{n-k}$$

Combinations:

$$L^n = [(L-1)+1]^n = \sum_{k=0}^{n} \binom{n}{k} (L-1)^k \, (1)^{n-k}$$

$$= \sum_{k=0}^{n} \binom{n}{k} (L-1)^k = 4^6 = 2^{12}$$

	DNA Element	Combinatorial Counts			
A	B	C	D	E	F
k	**6-nt Half-Site**	Combinations $C = \binom{n}{k}$	4-nts $D = C \times 4^k$	Unique $E = C \times 3^k$	Total Unique $F = \Sigma E$
**0**	0-nt Variant Half-Site	1	1	**1**	1
**1**	1-nt Variant Half-Site	6	24	**18**	19
**2**	2-nt Variant Half-Site	15	240	**135**	154
**3**	3-nt Variant Half-Site	20	1,280	**540**	694
**4**	4-nt Variant Half-Site	15	3,840	**1,215**	1,909
**5**	5-nt Variant Half-Site	6	6,144	**1,458**	3,367
**6**	6-nt Variant Half-Site	1	4,096	**729**	4,096

A	B	C	D	E	F
k	**AGGTCAnnnTGACCT AGAACAnnnTGTTCT**	Combinations $C = \binom{n}{k}$	4-nts $D = C \times 4^k$	Unique $E = C \times 3^k$	Total Unique $F = \Sigma E$
**0**	0-nt Variant	6!/(0! x 6!)	1 x 1	1 x 1	1 + 0
**1**	1-nt Variant	6!/(1! x 5!)	6 x 4	6 x 3	18 + 1
**2**	2-nt Variant	6!/(2! x 4!)	15 x 16	15 x 9	135 + 19
**3**	3-nt Variant	6!/(3! x 3!)	20 x 64	20 x 27	540 + 154
**4**	4-nt Variant	6!/(4! x 2!)	15 x 256	15 x 81	1,215 + 694
**5**	5-nt Variant	6!/(5! x 1!)	6 x 1,024	6 x 243	1,458 + 1,909
**6**	6-nt Variant	6!/(6! x 0!)	1 x 4,096	1 x 729	729 + 3,367

Categorize 0-nt to 5-nt Variant 10-nt p53RE DNA Elements Into 252 Half-Site Groups

To evaluate the DNA-binding of p53 at every DNA element in the 0-nt to 5-nt variant groups, the absolute number of times each of the 81,922 0-nt to 5-nt variant DNA elements occurred in an experiment were counted. For display purposes, we have defined a 5-nt variant p53RE DNA element by its five (5) fixed positions, resulting in 252 half-site groups (i.e., categorizing the 61,236 5-nt variant DNA elements into 252 half-site groups, defined by the 5 positions that are fixed/not varied). The remaining 0-nt to 4-nt variant DNA elements (1 0-nt variant consensus palindromic DNA element, 30 1-nt variant DNA elements, 405 2-nt variant DNA elements, 3,240 3-nt variant DNA elements, and 17,010 4-nt variant DNA elements, for a total of 20,686 DNA elements) can be categorized into these same 252 half-site groups (i.e., 5 positions are fixed, allowing for *up to* 5 positions to be varied). For example, the half-site group 1-2-3-4-5 contains all 1-nt variant DNA elements that vary positions 6 through 10. In the case of the p53RE, this includes the following 1-nt variant p53RE DNA elements: 6A, 6C, 6T, 7A, 7C, 7T, 8A, 8C, 8T, 9A, 9G, 9T, 10C, 10G, 10T. Thus, each of the 252 half-site groups contain 1 (0-nt variant consensus palindromic DNA element), 15 (1-nt variant DNA elements), 90 (2-nt variant DNA elements), 270 (3-nt variant DNA elements), 405 (4-nt variant DNA elements), and 243 (5-nt variant DNA elements), for a total of 1,024 DNA elements per half-site group. This allows all 81,922 0-nt to 5-nt variant DNA elements to be categorized into the 252 half-site groups, and thus providing the ability to sequentially track p53 DNA-binding at all 81,922 0-nt to 5-nt variant p53RE DNA elements in the genome **(Table S51-S52)**.

Of these 252 half-site groups, thirty-two (32) have zero vacancies, one hundred and sixty (160) have one vacancy, and sixty (60) have two vacancies.

See **Table S75** for script codes to convert the counts of all overlapped 0-nt to 5-nt DNA elements into the half-site groups.

Four nucleotide possibilities in a DNA element with 5 primary positions:

n = number of primary positions in the DNA element = **5**

L = number of nucleotide possibilities = **4**

k = number of variants

Binomial Equation:

$$(x+y)^n = \sum_{k=0}^{n} \binom{n}{k} x^k y^{n-k}$$

Combinations:

$$L^n = [(L-1)+1]^n = \sum_{k=0}^{n} \binom{n}{k} (L-1)^k \, (1)^{n-k}$$

$$= \sum_{k=0}^{n} \binom{n}{k} (L-1)^k = 4^5 = 2^{10}$$

	DNA Element	Combinatorial Counts			
A	B	C	D	E	F
k	**5-nt Half-Site**	**Combinations** $C = \binom{n}{k}$	**4-nts** $D = C \times 4^k$	**Unique** $E = C \times 3^k$	**Total Unique** $F = \Sigma E$
**0**	0-nt Variant Half-Site	1	1	**1**	1
**1**	1-nt Variant Half-Site	5	20	**15**	16
**2**	2-nt Variant Half-Site	10	160	**90**	106
**3**	3-nt Variant Half-Site	10	640	**270**	376
**4**	4-nt Variant Half-Site	5	1,280	**405**	781
**5**	5-nt Variant Half-Site	1	1,024	**243**	1,024

A	B	C	D	E	F
k	**TGCCCGGGCA**	**Combinations** $C = \binom{n}{k}$	**4-nts** $D = C \times 4^k$	**Unique** $E = C \times 3^k$	**Total Unique** $F = \Sigma E$
**0**	0-nt Variant	5!/(0! x 5!)	1 x 1	1 x 1	1 + 0
**1**	1-nt Variant	5!/(1! x 4!)	5 x 4	5 x 3	15 + 1
**2**	2-nt Variant	5!/(2! x 3!)	10 x 16	10 x 9	90 + 16
**3**	3-nt Variant	5!/(3! x 2!)	10 x 64	10 x 27	270 + 106
**4**	4-nt Variant	5!/(4! x 1!)	5 x 256	5 x 81	405 + 376
**5**	5-nt Variant	5!/(5! x 0!)	1 x 1,024	1 x 243	243 + 781

Signal-To-Noise (S/N) Analysis

sNR DNA-Binding at 0-nt to 5-nt Variant 13-nt ERE and HRE DNA Elements in the Genome

The signal-to-noise ratio (S/N) is the absolute number of times a 0-nt to 5-nt variant 13-nt ERE or HRE DNA element occurs in an experiment [defined by the total number of peaks in the experiment and the peak length] compared to the random frequency expectation of that DNA element occurring in the genome (i.e., the probability that any 10-nt DNA element that has a maximum possibility of 4 nucleotides in each position will occur in the genome is once every 1,048,576 nucleotides (4^{10}) at random occurrence). Thus, the expected noise is calculated by multiplying the total number of peaks in the experiment by the length of the peaks over 4^{10}.

For (S/N) analysis of sNR DNA-binding at 0-nt to 5-nt variant 13-nt ERE or HRE DNA elements in the genome (displayed by the number of variants in the DNA element), the expected noise is multiplied by the number of DNA elements in each group: 1 0-nt variant consensus palindromic DNA element, 30 1-nt variant DNA elements, 405 2-nt variant DNA elements, 3,240 3-nt variant DNA elements, 17,010 4-nt variant DNA elements, and 61,236 5-nt variant DNA elements, for a total of 81,922 0-nt to 5-nt variant DNA elements.

For (S/N) analysis of sNR DNA-binding at 0-nt to 5-nt variant 13-nt ERE or HRE DNA elements in the genome (displayed by the variant position in the DNA element) (i.e., 10 variant positions in 1-nt variant DNA elements, 45 variant positions in 2-nt variant DNA elements, 120 variant positions in 3-nt variant DNA elements, 210 variant positions in 4-nt variant DNA elements, 252 variant positions in 5-nt variant DNA elements), the expected noise is multiplied by the number of DNA elements in each group: 1 (0-nt variant consensus palindromic DNA element), 3 (1-nt variant DNA elements), 9 (2-nt variant DNA elements), 27 (3-nt variant DNA elements), 81 (4-nt variant DNA elements), and 243 (5-nt variant DNA elements), for a total of 81,922 0-nt to 5-nt variant DNA elements.

	**DNA Element**	**Combinatorial Counts**			
**A**	**B**	**C**	**D**	**E**	**F**
k	**13-nt ERE or HRE (*n*=10)**	**Combinations** C = $\binom{n}{k}$	**4-nts** D = C x 4^k	**Unique** E = C x 3^k	**Total Unique** F = ΣE
**0**	0-nt Variant ERE or HRE	**1**	1	**1**	1
**1**	1-nt Variant ERE or HRE	**10**	40	**30**	31
**2**	2-nt Variant ERE or HRE	**45**	720	**405**	436
**3**	3-nt Variant ERE or HRE	**120**	7,680	**3,240**	3,676
**4**	4-nt Variant ERE or HRE	**210**	53,760	**17,010**	20,686
**5**	5-nt Variant ERE or HRE	**252**	258,048	**61,236**	**81,922**

**A**	**B**	**C**	**D**	**E**	**F**
k		**Combinations** C = $\binom{n}{k}$	**4-nts** D = C x 4^k	**Unique** E = C x 3^k	**Total Unique** F = ΣE
**0**	0-nt Variant	10!/(0! x 10!)	1 x 1	1 x **1**	1 + 0
**1**	1-nt Variant	10!/(1! x 9!)	10 x 4	10 x **3**	30 + 1
**2**	2-nt Variant	10!/(2! x 8!)	45 x 16	45 x **9**	405 + 31
**3**	3-nt Variant	10!/(3! x 7!)	120 x 64	120 x **27**	3,240 + 436
**4**	4-nt Variant	10!/(4! x 6!)	210 x 256	210 x **81**	17,010 +3,676
**5**	5-nt Variant	10!/(5! x 5!)	252 x 1,024	252 x **243**	61,236 + 20,686

For (S/N) analysis of sNR DNA-binding at 0-nt to 5-nt variant 13-nt ERE or HRE DNA elements in the genome (displayed by the 252 half-site groups), the expected noise is multiplied by the number of DNA elements in each group: 1 (0-nt variant consensus palindromic DNA element), 15 (1-nt variant DNA elements), 90 (2-nt variant DNA elements), 270 (3-nt variant DNA elements), 405 (4-nt variant DNA elements), and 243 (5-nt variant DNA elements), for a total of 1,024 0-nt to 5-nt variant DNA elements per half-site group.

	**DNA Element**	**Combinatorial Counts**			
**A**	**B**	**C**	**D**	**E**	**F**
k	**13-nt Half-Site (*n*=5)**	**Combinations** C = $\binom{n}{k}$	**4-nts** D = C x 4^k	**Unique** E = C x 3^k	**Total Unique** F = ΣE
**0**	0-nt Variant Half-Site	1	1	**1**	1
**1**	1-nt Variant Half-Site	5	20	**15**	16
**2**	2-nt Variant Half-Site	10	160	**90**	106
**3**	3-nt Variant Half-Site	10	640	**270**	376
**4**	4-nt Variant Half-Site	5	1,280	**405**	781
**5**	5-nt Variant Half-Site	1	1,024	**243**	1,024

**A**	**B**	**C**	**D**	**E**	**F**

k		Combinations $C = \binom{n}{k}$	4-nts $D = C \times 4^k$	Unique $E = C \times 3^k$	Total Unique $F = \Sigma E$
**0**	0-nt Variant	5!/(0! x 5!)	1 x 1	1 x 1	1 + 0
**1**	1-nt Variant	5!/(1! x 4!)	5 x 4	5 x 3	15 + 1
**2**	2-nt Variant	5!/(2! x 3!)	10 x 16	10 x 9	90 + 16
**3**	3-nt Variant	5!/(3! x 2!)	10 x 64	10 x 27	270 + 106
**4**	4-nt Variant	5!/(4! x 1!)	5 x 256	5 x 81	405 + 376
**5**	5-nt Variant	5!/(5! x 0!)	1 x 1,024	1 x 243	243 + 781

The upper and lower one-tailed Poisson significance thresholds at p<0.001 were calculated using R: qpois(c(0.001,0.999),((total number of peaks in the experiment x peak length x number of DNA elements)/4^{10})).

sNR DNA-Binding at 0-nt to 6-nt Variant 15-nt ERE and HRE DNA Elements in the Genome

The signal-to-noise ratio (S/N) is the absolute number of times a 0-nt to 6-nt variant 15-nt ERE or HRE DNA element occurs in an experiment [defined by the total number of peaks in the experiment and the peak length] compared to the random frequency expectation of that DNA element occurring in the genome (i.e., the probability that any 12-nt DNA element that has a maximum possibility of 4 nucleotides in each position will occur in the genome is once every 16,777,216 nucleotides (4^{12}) at random occurrence). Thus, the expected noise is calculated by multiplying the total number of peaks in the experiment by the length of the peaks over 4^{12}.

For (S/N) analysis of sNR DNA-binding at 0-nt to 6-nt variant 15-nt ERE or HRE DNA elements in the genome (displayed by the number of variants in the DNA element), the expected noise is multiplied by the number of DNA elements in each group: 1 0-nt variant consensus palindromic DNA element, 36 1-nt variant DNA elements, 594 2-nt variant DNA elements, 5,940 3-nt variant DNA elements, 40,095 4-nt variant DNA elements, 192,456 5-nt variant DNA elements, and 673,596 6-nt variant DNA elements, for a total of 912,718 0-nt to 6-nt variant DNA elements.

For (S/N) analysis of sNR DNA-binding at 0-nt to 6-nt variant 15-nt ERE or HRE DNA elements in the genome (displayed by the variant position in the DNA element) (i.e., 12 variant positions in 1-nt variant DNA elements, 66 variant positions in 2-nt variant DNA elements, 220 variant positions in 3-nt variant DNA elements, 495 variant positions in 4-nt variant DNA elements, 792 variant positions in 5-nt variant DNA elements, 924 variant positions in 6-nt variant DNA elements), the expected noise is multiplied by the number of DNA elements in each group: 1 (0-nt variant consensus palindromic DNA element), 3 (1-nt variant DNA elements), 9 (2-nt variant DNA elements), 27 (3-nt variant DNA elements), 81 (4-nt variant DNA elements), 243 (5-nt variant DNA elements), and 729 (6-nt variant DNA elements), for a total of 912,718 0-nt to 6-nt variant DNA elements.

	**DNA Element**	**Combinatorial Counts**			
**A**	**B**	**C**	**D**	**E**	**F**
k	**15-nt ERE or HRE (*n*=12)**	**Combinations** C = $\binom{n}{k}$	**4-nts** D = C x 4^k	**Unique** E = C x 3^k	**Total Unique** F = ΣE
**0**	0-nt Variant ERE or HRE	**1**	1	**1**	1
**1**	1-nt Variant ERE or HRE	**12**	48	**36**	37
**2**	2-nt Variant ERE or HRE	**66**	1056	**594**	631
**3**	3-nt Variant ERE or HRE	**220**	14,080	**5,940**	6,571
**4**	4-nt Variant ERE or HRE	**495**	126,720	**40,095**	46,666
**5**	5-nt Variant ERE or HRE	**792**	811,008	**192,456**	239,122
**6**	6-nt Variant ERE or HRE	**924**	3,784,704	**673,596**	**912,718**

**A**	**B**	**C**	**D**	**E**	**F**
k		**Combinations** C = $\binom{n}{k}$	**4-nts** D = C x 4^k	**Unique** E = C x 3^k	**Total Unique** F = ΣE
**0**	0-nt Variant	12!/(0! x 12!)	1 x 1	1 x **1**	1 + 0
**1**	1-nt Variant	12!/(1! x 11!)	12 x 4	12 x **3**	36 + 1
**2**	2-nt Variant	12!/(2! x 10!)	66 x 16	66 x **9**	594 + 37
**3**	3-nt Variant	12!/(3! x 9!)	220 x 64	220 x **27**	5,940 + 631
**4**	4-nt Variant	12!/(4! x 8!)	495 x 256	495 x **81**	40,095 + 6,571
**5**	5-nt Variant	12!/(5! x 7!)	792 x 1,024	792 x **243**	192,456 + 46,666
**6**	6-nt Variant	12!/(6! x 6!)	924 x 4,096	924 x **729**	673,596 + 239,122

For (S/N) analysis of sNR DNA-binding at 0-nt to 6-nt variant 15-nt ERE or HRE DNA elements in the genome (displayed by the 924 half-site groups), the expected noise is multiplied by the number of DNA elements in each group: 1 (0-nt variant consensus palindromic DNA element), 18 (1-nt variant DNA elements), 135 (2-nt variant DNA elements), 540 (3-nt variant DNA elements), 1,215 (4-nt variant DNA elements), 1,458 (5-nt variant DNA elements), and 729 (6-nt variant DNA elements), for a total of 4,096 0-nt to 6-nt variant DNA elements per half-site group.

	**DNA Element**	**Combinatorial Counts**			
**A**	**B**	**C**	**D**	**E**	**F**
k	**15-nt Half-Site (*n*=6)**	**Combinations** C = $\binom{n}{k}$	**4-nts** D = C x 4^k	**Unique** E = C x 3^k	**Total Unique** F = ΣE
**0**	0-nt Variant Half-Site	1	1	**1**	1
**1**	1-nt Variant Half-Site	6	24	**18**	19
**2**	2-nt Variant Half-Site	15	240	**135**	154
**3**	3-nt Variant Half-Site	20	1,280	**540**	694
**4**	4-nt Variant Half-Site	15	3,840	**1,215**	1,909
**5**	5-nt Variant Half-Site	6	6,144	**1,458**	3,367
**6**	6-nt Variant Half-Site	1	4,096	**729**	4,096

**A**	**B**	**C**	**D**	**E**	**F**

k		Combinations<br>C = $\binom{n}{k}$	4-nts<br>D = C x 4^k	Unique<br>E = C x 3^k	Total Unique<br>F = ΣE
**0**	0-nt Variant	6!/(0! x 6!)	1 x 1	1 x 1	1 + 0
**1**	1-nt Variant	6!/(1! x 5!)	6 x 4	6 x 3	18 + 1
**2**	2-nt Variant	6!/(2! x 4!)	15 x 16	15 x 9	135 + 19
**3**	3-nt Variant	6!/(3! x 3!)	20 x 64	20 x 27	540 + 154
**4**	4-nt Variant	6!/(4! x 2!)	15 x 256	15 x 81	1,215 + 694
**5**	5-nt Variant	6!/(5! x 1!)	6 x 1,024	6 x 243	1,458 + 1,909
**6**	6-nt Variant	6!/(6! x 0!)	1 x 4,096	1 x 729	729 + 3,367

The upper and lower one-tailed Poisson significance thresholds at $p<0.001$ were calculated using R: qpois(c(0.001,0.999),((total number of peaks in the experiment x peak length x number of DNA elements)/4^{12})).

p53 DNA-Binding at 0-nt to 5-nt Variant 10-nt p53RE DNA Elements in the Genome

The signal-to-noise ratio (S/N) is the absolute number of times a 0-nt to 5-nt variant 10-nt p53RE DNA element occurs in an experiment [defined by the total number of peaks in the experiment and the peak length] compared to the random frequency expectation of that DNA element occurring in the genome (i.e., the probability that any 10-nt DNA element that has a maximum possibility of 4 nucleotides in each position will occur in the genome is once every 1,048,576 nucleotides (4^{10}) at random occurrence). Thus, the expected noise is calculated by multiplying the total number of peaks in the experiment by the length of the peaks over 4^{10}.

For (S/N) analysis of p53 DNA-binding at 0-nt to 5-nt variant 10-nt p53RE DNA elements in the genome (displayed by the number of variants in the DNA element), the expected noise is multiplied by the number of DNA elements in each group: 1 0-nt variant consensus palindromic DNA element, 30 1-nt variant DNA elements, 405 2-nt variant DNA elements, 3,240 3-nt variant DNA elements, 17,010 4-nt variant DNA elements, and 61,236 5-nt variant DNA elements, for a total of 81,922 0-nt to 5-nt variant DNA elements.

For (S/N) analysis of p53 DNA-binding at 0-nt to 5-nt variant 10-nt p53RE DNA elements in the genome (displayed by the variant position in the DNA element) (i.e., 10 variant positions in 1-nt variant DNA elements, 45 variant positions in 2-nt variant DNA elements, 120 variant positions in 3-nt variant DNA elements, 210 variant positions in 4-nt variant DNA elements, 252 variant positions in 5-nt variant DNA elements), the expected noise is multiplied by the number of DNA elements in each group: 1 (0-nt variant consensus palindromic DNA element), 3 (1-nt variant DNA elements), 9 (2-nt variant DNA elements), 27 (3-nt variant DNA elements), 81 (4-nt variant DNA elements), and 243 (5-nt variant DNA elements), for a total of 81,922 0-nt to 5-nt variant DNA elements.

	DNA Element	Combinatorial Counts			
A	B	C	D	E	F
k	**p53RE (*n*=10)**	**Combinations C = $\binom{n}{k}$**	**4-nts D = C x 4^k**	**Unique E = C x 3^k**	**Total Unique F = ΣE**
**0**	0-nt Variant p53RE	**1**	1	**1**	1
**1**	1-nt Variant p53RE	**10**	40	**30**	31
**2**	2-nt Variant p53RE	**45**	720	**405**	436
**3**	3-nt Variant p53RE	**120**	7,680	**3,240**	3,676
**4**	4-nt Variant p53RE	**210**	53,760	**17,010**	20,686
**5**	5-nt Variant p53RE	**252**	258,048	**61,236**	**81,922**

A	B	C	D	E	F
k		**Combinations C = $\binom{n}{k}$**	**4-nts D = C x 4^k**	**Unique E = C x 3^k**	**Total Unique F = ΣE**
**0**	0-nt Variant	10!/(0! x 10!)	1 x 1	1 x **1**	1 + 0
**1**	1-nt Variant	10!/(1! x 9!)	10 x 4	10 x **3**	30 + 1
**2**	2-nt Variant	10!/(2! x 8!)	45 x 16	45 x **9**	405 + 31
**3**	3-nt Variant	10!/(3! x 7!)	120 x 64	120 x **27**	3,240 + 436
**4**	4-nt Variant	10!/(4! x 6!)	210 x 256	210 x **81**	17,010 +3,676
**5**	5-nt Variant	10!/(5! x 5!)	252 x 1,024	252 x **243**	61,236 + 20,686

For (S/N) analysis of p53 DNA-binding at 0-nt to 5-nt variant 10-nt p53RE DNA elements in the genome (displayed by the 252 half-site groups), the expected noise is multiplied by the number of DNA elements in each group: 1 (0-nt variant consensus palindromic DNA element), 15 (1-nt variant DNA elements), 90 (2-nt variant DNA elements), 270 (3-nt variant DNA elements), 405 (4-nt variant DNA elements), and 243 (5-nt variant DNA elements), for a total of 1,024 0-nt to 5-nt variant DNA elements per half-site group.

	DNA Element	Combinatorial Counts			
A	B	C	D	E	F
k	**10-nt Half-Site (*n*=5)**	**Combinations C = $\binom{n}{k}$**	**4-nts D = C x 4^k**	**Unique E = C x 3^k**	**Total Unique F = ΣE**
**0**	0-nt Variant Half-Site	1	1	**1**	1
**1**	1-nt Variant Half-Site	5	20	**15**	16
**2**	2-nt Variant Half-Site	10	160	**90**	106
**3**	3-nt Variant Half-Site	10	640	**270**	376
**4**	4-nt Variant Half-Site	5	1,280	**405**	781
**5**	5-nt Variant Half-Site	1	1,024	**243**	1,024

A	B	C	D	E	F
k		**Combinations C = $\binom{n}{k}$**	**4-nts D = C x 4^k**	**Unique E = C x 3^k**	**Total Unique F = ΣE**
**0**	0-nt Variant	5!/(0! x 5!)	1 x 1	1 x 1	1 + 0
**1**	1-nt Variant	5!/(1! x 4!)	5 x 4	5 x 3	15 + 1
**2**	2-nt Variant	5!/(2! x 3!)	10 x 16	10 x 9	90 + 16

**3**	3-nt Variant	5!/(3! x 2!)	10 x 64	10 x 27	270 + 106
**4**	4-nt Variant	5!/(4! x 1!)	5 x 256	5 x 81	405 + 376
**5**	5-nt Variant	5!/(5! x 0!)	1 x 1,024	1 x 243	243 + 781

The upper and lower one-tailed Poisson significance thresholds at $p<0.001$ were calculated using R: qpois(c(0.001,0.999),((total number of peaks in the experiment x peak length x number of DNA elements)/4^{10})).

Data Analysis Example (WT-E2-1hr.L4)

ER DNA-Binding at 0-nt to 5-nt Variant 13-nt ERE DNA Elements in the Genome

Using WT-E2-1hr.L4 [76,163 peaks, 146-nt peak length] as an example experiment, we provide step-by-step instructions of the data analysis from peak selection to (S/N) analysis of ER DNA-binding at 81,922 0-nt to 5-nt variant 13-nt ERE DNA elements in the genome, displayed by the 252 half-site groups. The WT-E2-1hr.L4 experiment contains 76,163 ChIPSeq peaks **(Table S64)**. These 76,163 peaks contain 827,919 0-nt to 5-nt variant 13-nt ERE DNA elements **(Table S64, Column E)**. The 0-nt to 5-nt variant DNA elements include the 1 0-nt variant consensus palindromic ERE DNA element, 30 1-nt variant DNA elements (10 variant positions), 405 2-nt variant DNA elements (45 variant positions), 3,240 3-nt variant DNA elements (120 variant positions), 17,010 4-nt variant DNA elements (210 variant positions), and 61,236 5-nt variant DNA elements (252 variant positions), for a total of 81,922 DNA elements **(Table S65)**. First, we categorize these 827,919 0-nt to 5-nt variant ERE DNA elements by variant position **(Table S65)**. Then we categorize them into the 252 half-site groups, defined by the 5 positions that are fixed/not varied **(Table S66)**. For example, the half-site group 1-2-3-4-5 contains all 1-nt variant ERE DNA elements that vary positions 6 through 10 (i.e., 6A, 6C, 6G, 7A, 7C, 7T, 8C, 8G, 8T, 9A, 9G, 9T, 10A, 10G, 10T) **(Table S1)**, totaling 3,724 in this experiment **(Table S65-S66, Yellow)**. To convert this count to a (S/N) value, multiply the 76,163 peaks by the 146-nt peak length (=11,119,798 nucleotides), and divide 11,119,798 nucleotides by 4^{10} (i.e., the probability that any 10-nt DNA element that has a maximum possibility of 4 nucleotides in each position will occur in the genome is once every 1,048,576 nucleotides (4^{10}) at random occurrence), which yields an expected noise signal of 10.60 **(Table S66, Orange)**. There are 15 (1-nt variant DNA elements) in each 252 half-site group, thus 10.60 x 15 =159.07 expected noise signal **(Table S66, Orange)**. The DNA element occurrence (3,724), divided by the expected noise (159.07), gives a (S/N) value of 23.41, which is displayed on the 252 half-site plots **(Figure 2; Table S66, Yellow)**.

Next, to ensure that the sNR DNA-binding signal (i.e., (S/N) value) at non-NRFE DNA elements (i.e., 3-nt to 5-nt variant ERE DNA elements) is not due to non-NRFE DNA elements existing within peaks that also contain an NRFE, we first determined the (S/N) value for all 827,919 0-nt to 5-nt variant ERE DNA elements: ER DNA-binding at the 0-nt variant consensus palindromic ERE DNA element =113.35, ER DNA-binding at 1-nt variant ERE DNA elements =23.47, ER DNA-binding at 2-nt variant ERE DNA elements =4.20, ER DNA-binding at 3-nt variant ERE DNA elements =1.36, ER DNA-binding at 4-nt variant ERE DNA elements =0.96, ER DNA-binding at 5-nt variant ERE DNA elements =0.90 **(Table S67)**. Next, we calculated the (S/N) value for all 827,919 0-nt to 5-nt variant ERE DNA elements while excluding peaks that have already been assigned to an ERE DNA element with less variants. Thus, peak assignment is given to the 0-nt variant consensus palindromic ERE DNA element or the ERE DNA element with the least number of variants relative to the 0-nt variant consensus palindromic ERE DNA element (i.e., defining each peak by a single DNA element with the total number of peaks in an experiment =100%). Of 76,163 Peaks, 1,201 peaks contain a 0-nt variant consensus palindromic ERE DNA element ((S/N) value =7188.01), 7,332 peaks contain a 1-nt variant ERE DNA element ((S/N) value =243.22), 15,490 peaks contain a 2-nt variant ERE DNA element ((S/N) value =19.53), 24,374 peaks contain a 3-nt variant ERE DNA element ((S/N) value =3.02), 25,194 peaks contain a 4-nt variant ERE DNA element ((S/N) value =1.08), 2,599 peaks contain a 5-nt variant ERE DNA element ((S/N) value =0.96) **(Table S67)**. Next, we calculated the (S/N) value for all the other ERE DNA elements found within these groups **(Table S67)**. For example, of the 1,201 peaks that contain a 0-nt variant consensus palindromic ERE DNA element ((S/N) value =7188.01), 17 of them also contain a 1-nt variant ERE DNA element ((S/N) value =3.59), 88 of them also contain a 2-nt variant ERE DNA element ((S/N) value =1.34), 419 of them also contain a 3-nt variant ERE DNA element ((S/N) value =0.98), 1,055 of them also contain a 4-nt variant ERE DNA element ((S/N) value =0.83), and all 1,201 of them also contain a 5-nt variant ERE DNA element ((S/N) value =0.85) **(Table S67)**. The large (S/N) value for the ERE DNA element with the least number of variants, while the presence of other ERE DNA elements within those peaks are not enriched over what would be expected at random occurrence, was also observed in the 1-nt to 5-nt variant DNA element groups **(Table S67)**. This confirms that the DNA-binding structure observed at non-NRFE DNA elements in the genome is independent of the binding peaks that contain an NRFE DNA element.

(S/N) Value 13-nt ERE	Peaks Assigned to the ERE DNA Element with the Least Number of Variants					
	0-nt Variant ERE	1-nt Variant ERE	2-nt Variant ERE	3-nt Variant ERE	4-nt Variant ERE	5-nt Variant ERE
Other ERE DNA Elements Also Within Peaks	1,201 Peaks	7,332 Peaks	15,490 Peaks	24,347 Peaks	25,194 Peaks	2,599 Peaks
0-nt Variant ERE	**7188.01**					
1-nt Variant ERE	3.59	**243.22**				
2-nt Variant ERE	1.34	2.09	**19.53**			
3-nt Variant ERE	0.98	1.11	1.32	**3.02**		
4-nt Variant ERE	0.83	0.89	0.93	0.98	**1.08**	
5-nt Variant ERE	0.85	0.85	0.87	0.89	0.92	**0.96**

ER DNA-Binding at 0-nt to 6-nt Variant 15-nt ERE DNA Elements in the Genome

Using this same experiment, WT-E2-1hr.L4, we provide step-by-step instructions of the data analysis from peak selection to (S/N) analysis of ER DNA-binding at 912,718 0-nt to 6-nt variant 15-nt ERE DNA elements in the genome, displayed by the 924 half-site groups. The WT-E2-1hr.L4 experiment contains 76,163 ChIPSeq peaks **(Table S68)**. These 76,163 peaks contain 605,166 0-nt to 6-nt variant 15-nt ERE DNA elements **(Table S68, Column E)**. The 0-nt to 6-nt variant DNA elements include the 1 0-nt variant consensus palindromic DNA element, 36 1-nt variant DNA elements (12 variant positions), 594 2-nt variant DNA elements (66 variant positions), 5,940 3-nt variant DNA elements (220 variant positions), 40,095 4-nt variant DNA elements (495 variant positions), 192,456 5-nt variant DNA elements (792 variant positions), and 673,596 6-nt variant DNA elements (924 variant positions), for a total of 912,718 DNA elements **(Table S69)**. First, we categorize these 605,166 0-nt to 6-nt variant ERE DNA elements by variant position **(Table S69)**. Then we categorize them into the 924 half-site groups, defined by the 6 positions that are fixed/not varied **(Table S70)**. For example, the half-site group 1-2-3-4-5-6 contains all 1-nt variant ERE DNA elements that vary positions 7 through 12 (i.e., 7A, 7C, 7G, 8A, 8C, 8T, 9C, 9G, 9T, 10A, 10G, 10T, 11A, 11G, 11T, 12A, 12C, 12G) **(Table S21)**, totaling 1,320 in this experiment **(Table S69-S70, Yellow)**. To convert this count to a (S/N) value, multiply the 76,163 peaks by the 146-nt peak length (=11,119,798 nucleotides), and divide 11,119,798 nucleotides by 4^{12} (i.e., the probability that any 12-nt DNA element that has a maximum possibility of 4 nucleotides in each position will occur in the genome is once every 16,777,216 nucleotides (4^{12}) at random occurrence), which yields an expected noise signal of 0.66 **(Table S70, Orange)**. There are 18 (1-nt variant DNA elements) in each 924 half-site group, thus 0.66 x 18 =11.93 expected noise signal **(Table S70, Orange)**. The DNA element occurrence (1,320), divided by the expected noise (11.93), gives a (S/N) value of 110.64, which is displayed on the 924 half-site plots **(Figure S40; Table S70, Yellow)**.

Next, to ensure that the sNR DNA-binding signal (i.e., (S/N) value) at non-NRFE DNA elements is not due to non-NRFE DNA elements existing within peaks that also contain an NRFE, we first determined the (S/N) value for all 605,166 0-nt to 6-nt variant ERE DNA elements: ER DNA-binding at the 0-nt variant consensus palindromic ERE DNA element =460.17, ER DNA-binding at 1-nt variant ERE DNA elements =113.91, ER DNA-binding at 2-nt variant ERE DNA elements =20.59, ER DNA-binding at 3-nt variant ERE DNA elements =4.28, ER DNA-binding at 4-nt variant ERE DNA elements =1.56, ER DNA-binding at 5-nt variant ERE DNA elements =1.03, ER DNA-binding at 6-nt variant ERE DNA elements =0.91 **(Table S71)**. Next, we calculated the (S/N) value for all 605,166 0-nt to 6-nt variant ERE DNA elements, while excluding peaks that have already been assigned to an ERE DNA element with less variants. Thus, peak assignment is given to the 0-nt variant consensus palindromic ERE DNA element or the ERE DNA element with the least number of variants relative to the 0-nt variant consensus palindromic ERE DNA element (i.e., defining each peak by a single DNA element with the total number of peaks in an experiment =100%). Of 76,163 Peaks, 304 peaks contain a 0-nt variant consensus palindromic ERE DNA element ((S/N) value =115290.44), 2,705 peaks contain a 1-nt variant ERE DNA element ((S/N) value =3204.99), 7,840 peaks contain a 2-nt variant ERE DNA element ((S/N) value =198.09), 13,922 peaks contain a 3-nt variant ERE DNA element ((S/N) value =21.35), 21,585 peaks contain a 4-nt variant ERE DNA element ((S/N) value =3.78), 24,479 peaks contain a 5-nt variant ERE DNA element ((S/N) value =1.25), 5,308 peaks contain a 6-nt variant ERE DNA element ((S/N) value =0.96) **(Table S71)**. Next, we calculated the (S/N) value for all the other ERE DNA elements found within these groups **(Table S71)**. For example, of the 304 peaks that contain a 0-nt variant consensus palindromic ERE DNA element ((S/N) value =115290.44), 2 of them also contain a 1-nt variant ERE DNA element ((S/N) value =21.00), 2 of them also contain a 2-nt variant ERE DNA element ((S/N) value =1.27), 28 of them also contain a 3-nt variant ERE DNA element ((S/N) value =1.78), 120 of them also contain a 4-nt variant ERE DNA element ((S/N) value =1.43), 241 of them also contain a 5-nt variant ERE DNA element ((S/N) value =0.97), and all 304 of them also contain a 6-nt variant ERE DNA element ((S/N) value =0.92) **(Table S71)**. The large (S/N) value for the ERE DNA element with the least number of variants, while the presence of other ERE DNA elements within those peaks are not enriched over what would be expected at random occurrence, was also observed in the 1-nt to 6-nt variant DNA element groups **(Table S71)**. This confirms that the DNA-binding structure observed at non-NRFE DNA elements in the genome is independent of the binding peaks that contain an NRFE DNA element.

(S/N) Value 15-nt ERE	Peaks Assigned to the ERE DNA Element with the Least Number of Variants						
	0-nt Variant ERE	1-nt Variant ERE	2-nt Variant ERE	3-nt Variant ERE	4-nt Variant ERE	5-nt Variant ERE	6-nt Variant ERE
Other ERE DNA Elements Also Within Peaks	304 Peaks	2,705 Peaks	7,840 Peaks	13,922 Peaks	21,585 Peaks	24,479 Peaks	5,308 Peaks
0-nt Variant ERE	**115290.44**						
1-nt Variant ERE	21.00	**3204.99**					
2-nt Variant ERE	1.27	5.51	**198.09**				
3-nt Variant ERE	1.78	2.29	2.86	**21.35**			
4-nt Variant ERE	1.43	1.30	1.40	1.58	**3.78**		
5-nt Variant ERE	0.97	0.99	1.00	1.04	1.05	**1.25**	
6-nt Variant ERE	0.92	0.88	0.89	0.89	0.90	0.92	**0.96**

Mouse (mm10) and Human (hg19) Genome Assembly

The mouse and human genome assemblies were obtained from UCSC Genome Browser: mouse genome build 38 (mm10) and human genome build 37 (hg19). The genome size of the mouse (mm10) genome is 2,730,871,774 nucleotides and the genome size of the human (hg19) genome is 3,137,161,264 nucleotides **(Table S72)**.

Their effective sizes are 2,647,537,730 nucleotides (mm10) and 2,861,343,702 nucleotides (hg19) when excluding unreadable nucleotides, random regions, hap regions, and chromosome Un (i.e., clone contigs that cannot be confidently placed on a specific chromosome) from the reference **(Table S73)**.

Unreadable nucleotides account for 2.9% [77,999,939] (mm10) and 7.5% [234,350,281] (hg19) of the genome **(Table S73)**. These unreadable nucleotides are contained within 501 (mm10) and 339 (hg19) unreadable regions in the genome **(Table S74)**.

See **Table S75** for associated script codes.

Population Count of 1-nt to 20-nt DNA Elements in the Single-Stranded Mouse and Human Genome

Perl was used to obtain the counts of all 1-nt to 20-nt DNA elements in the single-stranded mouse and human genome **(Table S47-S50)**.

The equivalence was determined using the correlation coefficient, slope and y-intercept. Secondly, we took the measure of the variance of the points normalized by the overall length of the vector (i.e., sets of points), named the general error fraction (GEF). That is, the distance between the points normalized with respect to the overall length of the vector.

The population count of every 1-nt to 20-nt DNA elements in the single-stranded mouse and human genome, including its reverse-complement DNA element, reverse DNA element, and complement DNA element, have been provided (tar -xvzf *filename*.tar.gz to unzip files) **(Datafiles S2-S5)**.

See **Table S75** for associated script codes.

Global Error Fraction

Define:

$$E_K = \left|\frac{1}{2}(X_K - Y_K)\right|$$

$$A_K = \left|\frac{1}{2}(X_K + Y_K)\right|$$

$$Where\ X_K = DNA\ Element\ Frequency\ of\ X$$

$$Where\ Y_K = DNA\ Element\ Frequency\ of\ Y$$

$$\langle {E_K}^2 \rangle = \frac{1}{M}\sum_{K=1}^{M} {E_K}^2$$

$$\langle {A_K}^2 \rangle = \frac{1}{M}\sum_{K=1}^{M} {A_K}^2$$

$$\text{Global Error Fraction (GEF)} = \left[\frac{\langle {E_K}^2 \rangle}{\langle {A_K}^2 \rangle}\right]^{1/2}$$

Position Weight Matrices (PWMs) for DNA Motif Identification

Finding enriched DNA motifs in genomic regions (findMotifsGenome.pl)

The findMotifsGenome.pl program is a wrapper that helps set up the data for analysis using the HOMER motif discovery algorithm.

See **Table S75** for associated script codes.

Supplemental References

1. Aerts S. Chapter five - Computational Strategies for the Genome-Wide Identification of cis-Regulatory Elements and Transcriptional Targets. In: Plaza S, Payre F, editors. Current Topics in Developmental Biology: Academic Press; 2012. p. 121-45.

2. Coons LA, Hewitt SC, Burkholder AB, McDonnell DP, Korach KS. DNA Sequence Constraints Define Functionally Active Steroid Nuclear Receptor Binding Sites in Chromatin. Endocrinology. 2017;158(10):3212-34. Epub 2017/10/05. doi: 10.1210/en.2017-00468. PubMed PMID: 28977594; PMCID: Pmc5659708.

3. Tonelli C, Morelli MJ, Sabo A, Verrecchia A, Rotta L, Capra T, Bianchi S, Campaner S, Amati B. Genome-wide analysis of p53-regulated transcription in Myc-driven lymphomas. Oncogene. 2017;36(21):2921-9. Epub 2017/01/17. doi: 10.1038/onc.2016.443. PubMed PMID: 28092679; PMCID: PMC5454316.

4. Sahu B, Pihlajamaa P, Dubois V, Kerkhofs S, Claessens F, Janne OA. Androgen receptor uses relaxed response element stringency for selective chromatin binding and transcriptional regulation in vivo. Nucleic acids research. 2014;42(7):4230-40. Epub 2014/01/25. doi: 10.1093/nar/gkt1401. PubMed PMID: 24459135; PMCID: Pmc3985627.

5. Kharchenko PV, Tolstorukov MY, Park PJ. Design and analysis of ChIP-seq experiments for DNA-binding proteins. Nat Biotech. 2008;26(12):1351-9.

6. Langmead B, Salzberg SL. Fast gapped-read alignment with Bowtie 2. Nature methods. 2012;9(4):357-9. Epub 2012/03/06. doi: 10.1038/nmeth.1923. PubMed PMID: 22388286; PMCID: Pmc3322381.
